# Modular One‐Pot Access to π‐Expanded Tetrakis(Phenothiazinyl)‐Silanes With Broadly Tunable Redox and Emission Properties

**DOI:** 10.1002/chem.71368

**Published:** 2026-07-08

**Authors:** Thomas P. M. Merke, Lysander Presser, Leonard Karl, Christian Ganter, Thomas J. J. Müller

**Affiliations:** ^1^ Institut für Organische Chemie und Makromolekulare Chemie Heinrich‐Heine‐Universität Düsseldorf Düsseldorf Germany; ^2^ Institut für Anorganische Chemie und Strukturchemie I Heinrich‐Heine‐Universität Düsseldorf Düsseldorf Germany

**Keywords:** cross‐coupling, cyclic voltammetry, DFT calculations, fluorescence, phenothiazines, silanes, substituent effects

## Abstract

The development of multichromophoric materials with independently tunable redox and emissive properties remains a central challenge in organic materials chemistry. Herein, we report a library of 21 predominantly C3‐connected tetrakis(phenothiazinyl)silanes, accessed principally via a modular one‐pot BLEBS (bromine–lithium exchange–borylation–Suzuki) strategy in moderate to very good yields (15%–87%), featuring systematic variation at the 10‐, 7‐ and 2‐positions of the phenothiazine scaffold, a C1‐connected regioisomeric reference, as well as four distinct linker architectures. Cyclic voltammetry reveals fully reversible first oxidations spanning +0.17 to +0.42 V versus Fc/Fc^+^, with a linear Hammett correlation (*σ*_p_, *R* ^2^ = 0.985) for the 7‐substituted subset confirming predictable electronic fine‐tuning. Photophysically, the tetramers exhibit bright fluorescence with quantum yields up to *Φ*_F_ = 0.90 and emission tunable from deep blue to yellow–green (441–533 nm) through linker engineering. (TD‐)DFT calculations confirm locally excited, phenothiazine‐centered emissive states and quantitatively reproduce the experimental variation in radiative rates via computed oscillator strengths. Notably, 7‐substitution selectively modulates the redox potentials while leaving the optical gap essentially invariant, whereas linker modification primarily governs the emission energy, establishing the tetrakis(phenothiazinyl)silane scaffold as a versatile platform for the largely independent control of electrochemical and photophysical properties.

## Graphical abstract

A library of 21 tetrakis(phenothiazinyl)‐substituted silanes with broadly variable substituent decoration, which are fully reversible multistage redox systems with intense tunable emission from deep blue to yellow–green (441–533 nm), was rapidly synthesized by bromine–lithium exchange/borylation–Suzuki coupling of tetrakis(4‐bromophenyl)silane or by direct fourfold nucleophilic substitution of tetrachlorosilane.

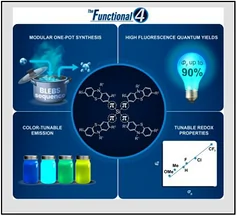

## 1 Introduction

Organic electronics have emerged as a transformative field at the interface of chemistry, physics, and materials science, driving the development of flexible, scalable, and cost‐effective technologies [1, 2, 3]. Devices such as organic light‐emitting diodes (OLEDs), organic field‐effect transistors (OFETs), and organic photovoltaics (OPVs) rely on functional π‐conjugated materials with precisely defined electronic structure and solid‐state organization [4, 5]. Beyond traditional polymers, π‐extended small molecules are increasingly employed to achieve high charge carrier mobilities, tunable emission, and improved morphological control [6]. Among the broad class of redox‐active heterocycles, phenothiazines have gained particular attention as electron‐rich, structurally versatile chromophores [7]. Phenothiazines are characterized by an inherent electron excess (16π‐electrons distributed over 14 skeletal atoms); to circumvent antiaromaticity, the thiazine core adopts a characteristic butterfly conformation folded along the *S*,*N*‐axis [8, 9]. This conformational feature has direct functional implications: the pronounced geometric reorganization toward a planarized S_1_ excited state gives rise to large Stokes shifts that reduce the spectral overlap between absorption and emission [9], thereby minimizing reabsorption losses in emissive applications. Furthermore, the preferential *intra*‐folded orientation of *N*‐substituents in the ground state maintains effective overlap of the nitrogen lone pair with the anellated π‐system, enhancing the intrinsic donor strength [10]. Combined with low reversible oxidation potentials and widely tunable emission, these properties render phenothiazines attractive building blocks for photoredox catalysis, light‐emitting materials, and hole‐transporting layers [11, 12].

Tetraphenylsilane is a well‐established platform in organic materials chemistry. Its rigid tetrahedral geometry and electronic tunability have motivated incorporation into diverse functional architectures, including acridine‐based TADF emitters [13], diketopyrrolopyrrole‐based photovoltaic materials [14], polycyanurate networks [15], metal–organic frameworks [16], porous organometallic networks [17], and tetraphosphane ligands [18]. In this context, tetrakis(phenothiazinyl)silanes constitute a still underexplored class of multichromophores in which four phenothiazine units are arranged on a rigid tetrahedral silane scaffold. The tetrahedral silicon center enforces a three‐dimensional, nonplanar topology that reduces π‐stacking and mitigates aggregation‐related quenching pathways often observed in planar π‐systems, while contributing high chemical and thermal stability [13]. To date, only *N*‐linked tetrakis(phenothiazinyl)silanes have been reported, disclosed in a patent as potential host and exciton‐blocking materials for OLED emissive layers [19], but their structure–property relationships have not been systematically investigated. In contrast, C‐linked analogues connected through the 3‐position remain unexplored (Figure 1), despite offering a distinct advantage over *N*‐linked systems: in C3‐connected phenothiazines, the conjugation pathway between the donor and the appended framework passes exclusively through the aromatic C3‐position and the pendant phenylene linker, with the inter‐ring torsion limited to a moderate biphenyl‐type distortion. The nitrogen atom is not part of the linkage pathway and therefore retains its role within the phenothiazine π‐system regardless of its conformational orientation, establishing a conformationally uniform linkage motif across the series. In *N*‐linked systems, by contrast, the nitrogen atom itself serves as the point of attachment; in the commonly adopted extra‐folded orientation, the nitrogen lone pair is poorly aligned with the anellated π‐system, rendering it conformationally disjunct from the conjugated framework [10]. The C3‐position furthermore coincides with a region of significant HOMO electron density [20], providing favorable conditions for effective conjugative coupling between the phenothiazine donor and the silicon‐centered scaffold. Beyond their structural novelty, C3‐connected tetrakis(phenothiazinyl)silanes are conceptually attractive because they provide two structurally separate handles for property modulation: peripheral substitution on the phenothiazine donor and variation of the linker bridging the donor to the silicon core. This separation is functionally relevant, because the same frontier orbital – the HOMO – determines both the first oxidation potential and, together with the LUMO, the optical gap, so that in many π‐conjugated chromophores a structural change tuning one property also shifts the other, making their independent adjustment within a single scaffold difficult.

**FIGURE 1:**
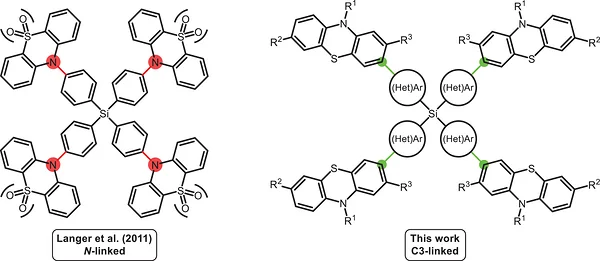
Comparison of *N*‐linked tetrakis(phenothiazinyl)silanes (Langer et al. [19]) and C3‐linked tetrakis(phenothiazinyl)silanes with systematic structural variation (this work).

However, the limited synthetic accessibility of selectively C3‐connected tetrakis(phenothiazinyl)silanes has so far impeded systematic investigation. Here, we report a modular one‐pot strategy based on the BLEBS (bromine–lithium exchange–borylation–Suzuki) sequence [21], complemented by direct lithiation and LiBS (lithiation–borylation–Suzuki) protocols [22], to access a structurally diverse library of C3‐connected tetrakis(phenothiazinyl)silanes. The electrochemical and photophysical properties are systematically investigated and rationalized by (TD‐)DFT calculations to establish comprehensive structure–property relationships across this previously unexplored compound class.

## 2 Results and Discussion

### 2.1 Synthesis

A rapid and modular access to a systematically varied tetramer library can be pursued by a telescoped one‐pot strategy that minimizes intermediate handling and is amenable to structural diversification (Scheme 1). The BLEBS sequence provides the pivotal synthetic platform to reach this goal [21]. The protocol concatenates metalation, borylation, and Pd‐catalyzed cross‐coupling in a single reaction vessel without isolation of reactive intermediates, thereby meeting key criteria of step economy and operational simplicity [23, 24, 25, 26]. Originally developed for the rapid construction of arylated heteroaromatics, the BLEBS sequence has been successfully applied to phenothiazines [21], dithienothiazines [27], and triarylamines [28]. In the present study, the method has been chosen to access both the functionalized phenothiazine coupling partners and the silicon‐centered target tetramers.

**SCHEME 1:**
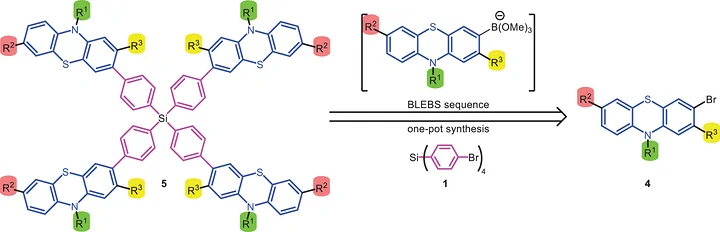
Retrosynthetic approach to phenylene‐expanded tetrakis(phenothiazinyl)silanes **5** via a modular one‐pot BLEBS sequence from tetrakis(4‐bromophenyl)silane (**1**) and substituted 3‐bromophenothiazines **4**.

Beyond the tetrafunctional core tetrakis(4‐bromophenyl)silane (**1**) [14], the synthesis of the target tetramers requires a set of mono‐brominated phenothiazines as coupling partners for the final one‐pot sequence. Substrates bearing substituents at the 10‐ or 2‐position (**4a**–**f**, **4o**–**q**) as well as a regioisomeric 1‐brominated precursor (**4r**) are synthesized following reported or adapted procedures, including nucleophilic substitution and Buchwald–Hartwig amination [29, 30, 31, 32, 33, 34, 35, 36]. In addition, a set of eight novel, asymmetrically substituted phenothiazines **4g**–**n** can be prepared by a BLEBS sequence starting from 3‐bromo‐10‐hexyl‐7‐iodo‐10*H*‐phenothiazine (**3**) (Scheme 2) [37]. A diverse set of aryl and heteroaryl bromides serves as coupling partners for the initiating bromine–lithium exchange–borylation, enabling the introduction of electron‐withdrawing (CF_3_, Cl, and F), electron‐neutral (H), electron‐donating (Me, OMe), and heteroaromatic (5‐methylthiophen‐2‐yl) substituents as well as a biphenothiazinyl unit. All intermediates **4g**–**n** can be obtained in good to excellent yields (74%–88%) in this one‐pot fashion.

**SCHEME 2:**
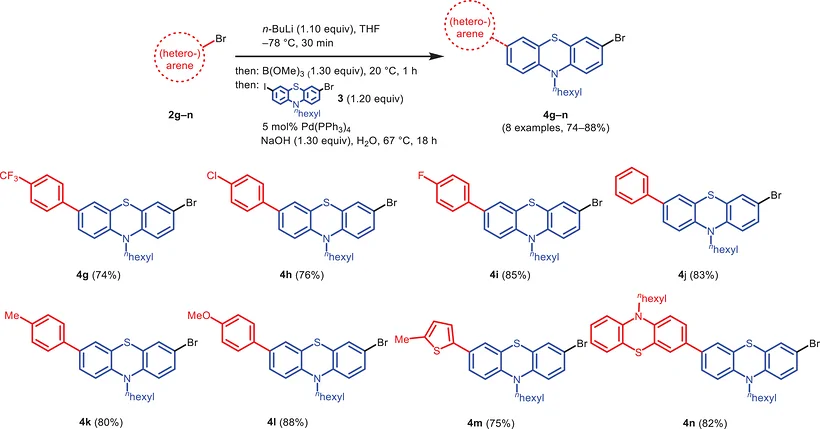
One‐pot BLEBS synthesis of 7‐substituted 3‐bromophenothiazines **4g**–**n** as cross‐coupling precursors.

With the complete set of coupling partners in hand, the silicon‐centered target tetramers are assembled in a one‐pot fashion via a fourfold BLEBS sequence starting from tetrakis(4‐bromophenyl)silane (**1**) (Scheme 3). This convergent strategy enables the formation of four C─C bonds from the tetravalent silicon core in a single operation. Substituent variation is achieved across multiple positions of the phenothiazine scaffold: aliphatic, benzylic, and aromatic groups at the 10‐position (**5a**–**f**), electron‐donating and electron‐withdrawing aryl and heteroaryl groups at the 7‐position (**5g**–**n**), donor‐functionalized groups at the 2‐position (**5o**–**q**), and a regioisomeric 1‐connected derivative (**5r**) as a structural reference. The target tetramers can be obtained after chromatographic purification in moderate to very good yields (49%–87%). Notably, the fourfold BLEBS protocol tolerates a broad range of substitution patterns without significant variation in efficiency.

**SCHEME 3:**
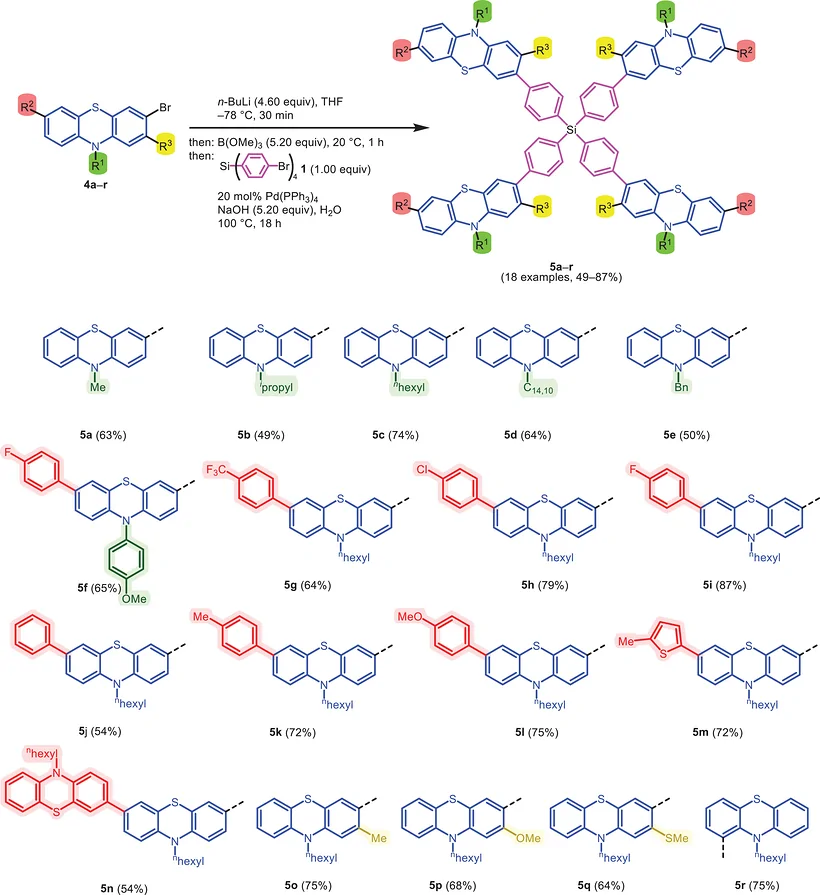
Fourfold one‐pot BLEBS protocol for the synthesis of phenylene‐expanded tetrakis(phenothiazinyl)silanes **5** from tetrakis(4‐bromophenyl)silane (**1**) and bromophenothiazines **4**.

In addition to variation at the phenothiazine periphery, structural diversity can be introduced at the central linker motif connecting the silicon core to the phenothiazine donors (Scheme 4). However, replacement of the phenylene spacer present in **5** requires alternative synthetic strategies adapted to the connectivity of each target. The directly connected tetramer **6**, lacking any π‐conjugated linker, is synthesized via bromine–lithium exchange on 3‐bromo‐10‐hexyl‐10*H*‐phenothiazine (**4c**) followed by electrophilic trapping with silicon tetrachloride in the sense of a nucleophilic substitution on silicon. The comparatively low yield of 15% reflects the increasing steric congestion at the silicon center during the successive substitution steps – particularly pronounced for this spacer‐free, direct Si–C connection – and the competitive formation of partially substituted silanes. The thienylene‐linked tetramer **7** is prepared analogously, exploiting the regioselective deprotonation of the α‐position of the thienyl substituent in 10‐hexyl‐3‐(thiophen‐2‐yl)‐10*H*‐phenothiazine (**4s**) with *n*‐butyllithium, followed by nucleophilic substitution at silicon tetrachloride, affording after isolation and purification the product in 31% yield. The higher yield compared to **6** is consistent with the diminished steric demand at the reaction center imparted by the thienylene spacer. The tetramer **8**, bearing a combined phenylene–thienylene linker, is accessed via a LiBS sequence [22] starting from the same thienyl phenothiazine precursor and tetrakis(4‐bromophenyl)silane (**1**). This variant proceeds via direct deprotonation of the thiophene unit, but is otherwise operationally identical to the standard BLEBS protocol. The yield of 61% is comparable to those obtained for the phenylene‐linked tetramers **5**, consistent with the analogous reaction mechanism and the reduced steric constraints relative to **6** and **7**.

**SCHEME 4:**
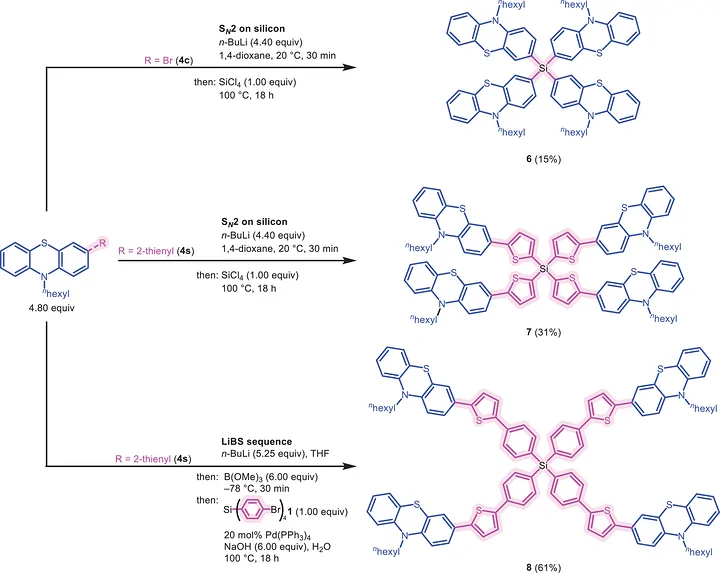
Metalation routes to linker‐modified tetrakis(phenothiazinyl)silanes **6**–**8** (direct, thienylene, and phenylene–thienylene linkers).

### 2.2 Structure

The chemical structures of all target compounds are confirmed by ^1^H and ^13^C NMR spectroscopy, IR spectroscopy, and MALDI mass spectrometry, and their elemental compositions are supported by elemental analysis. In addition, the molecular structure of compound **5a** was later unambiguously corroborated by single‐crystal X‐ray diffraction [38], providing insight into the solid‐state arrangement and conformational aspects for a representative member of the series. Among the compounds investigated, compound **5a** proves to be the only derivative amenable to single‐crystal growth, which can be attributed to the comparatively low conformational flexibility imparted by the compact *N*‐methyl substituent relative to the longer alkyl groups present in the remaining members of the library.

Since a formally planar 8π‐electron thiazine core is electronically disfavored, phenothiazines adopt the characteristic butterfly conformation folded along the *S*,*N*‐axis. This conformational folding positions substituents on the nitrogen atom in either an *intra*‐folded (quasi‐equatorial) or *extra*‐folded (quasi‐axial) orientation, with interconversion between the two orientations occurring via nitrogen inversion [8, 39].

Yellow needle‐shaped crystals of **5a** crystallize in the tetragonal space group I4_1_/a, revealing a conformationally twisted arrangement (Figure 2A). The pendant phenylene linker is distorted by 26.05(8)° relative to the benzene ring anellated to the 1,4‐thiazine fragment, indicating modest inter‐ring torsion. The phenothiazine core adopts the typical butterfly conformation in an *extra*‐folded orientation with a fold angle of 145.67(9)°, closely matching the value reported for *N*‐methylphenothiazine (143.7°) [40]. The *extra*‐folded orientation is associated with only slight pyramidalization at nitrogen, as evidenced by the near‐trigonal‐planar sum of bond angles (Σ∠N = 353.95(8)°, cf. ideal sp^2^ value of 360°), consistent with significant π‐delocalization across the phenothiazine scaffold. Overall, the bond metrics and conformational features of the phenothiazine moiety closely parallel those of unsubstituted *N*‐methylphenothiazine, indicating that incorporation into the tetrahedral silane framework does not perturb the intrinsic electronic structure of the donor unit. The silicon center adopts an essentially undistorted tetrahedral geometry, as evidenced by an average C–Si–C angle of 110.6(9)°. This near‐ideal tetrahedral arrangement permits a sterically unhindered spatial disposition of the four 4‐(10‐methyl‐10*H*‐phenothiazin‐3‐yl)phenyl substituents. Crystal packing does not reveal pronounced π‐stacking or other directional short contacts, suggesting that steric shielding by the tetrahedrally arranged substituents effectively prevents cofacial chromophore interactions. Hirshfeld surface analysis [41, 42, 43, 44] confirms this assessment quantitatively: H⋯H contacts dominate the intermolecular contact contributions, with no evidence of π⋯π, S⋯π, or other directional interactions (Figure 2B and Supporting Information). The absence of significant intermolecular chromophore coupling in the solid state is consistent with the retention of appreciable fluorescence quantum yields in the solid state discussed below.

**FIGURE 2:**
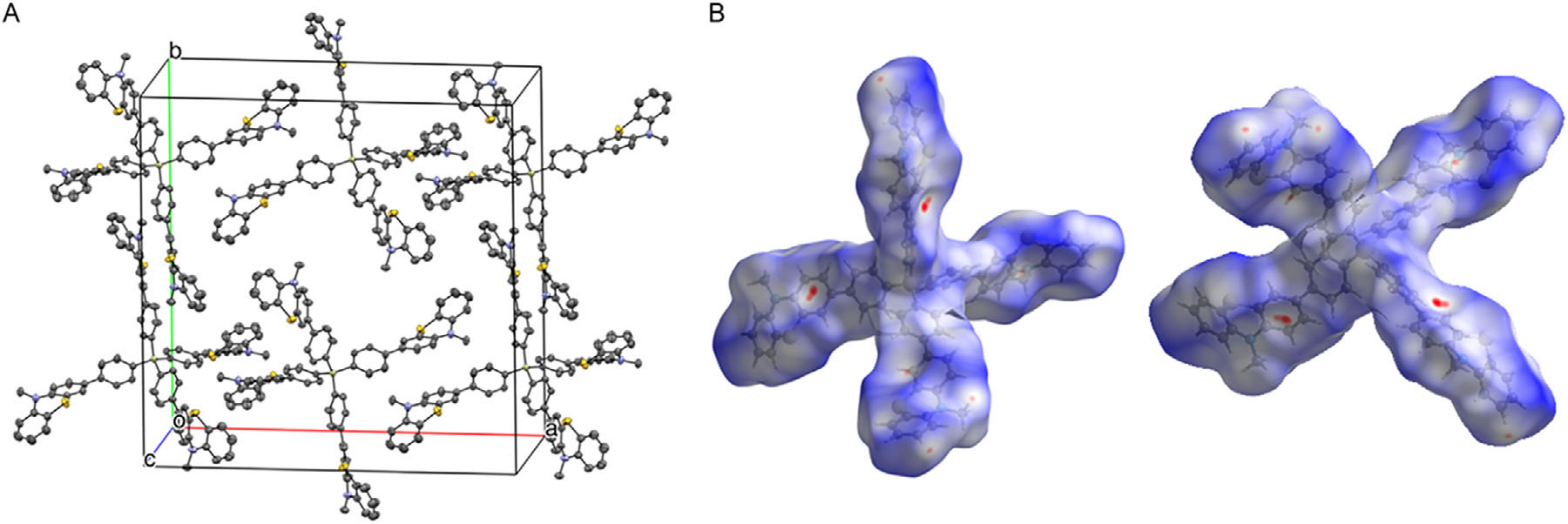
A: The packing of **5a** in the single crystal is depicted with thermal ellipsoids at 50% probability. Hydrogen atoms have been omitted for clarity. B: Hirshfeld surface of **5a** mapped with the d_norm_ property. The red spots represent the closest contacts and blue the most distant contacts. A breakdown of contact contributions is provided in the Supporting Information. The Hirshfeld analysis was performed according to the procedures described in the literature [41, 42, 43, 44].

### 2.3 Electrochemical Properties

Cyclic voltammetry was employed to assess the redox properties of the tetrakis(phenothiazinyl)silanes (Table 1). For all compounds, the first oxidation appears as a single, well‐defined, reversible wave. Each tetramer carries four structurally equivalent phenothiazine donors, each of which is an established one‐electron redox unit forming a persistent radical cation; the wave is accordingly assigned to four phenothiazine‐centered one‐electron oxidations. For four equivalent, noninteracting donors, the successive one‐electron steps are separated only by small statistical factors and therefore merge into a single voltammetric wave, so that a single reversible wave is the behavior expected for negligibly coupled, near‐degenerate redox sites. Several independent observations substantiate this assignment. The tetrahedral silicon center is a *σ*‐bonded, electronically insulating spacer that offers no π‐conjugation pathway between the arms, and the single‐crystal structure of **5a** together with the Hirshfeld analysis reveals no π⋯π or other directional interchromophore contacts, thus precluding both through‐bond and through‐space communication. Consistently, the (TD‐)DFT analysis shows the occupied frontier orbitals to be localized on the phenothiazine arms (see DFT section); the oxidation is therefore phenothiazine‐centered and distributed over four structurally equivalent, spatially separated donor units rather than a single delocalized π‐system, and the equivalence of these units implies near‐degenerate one‐electron oxidations. The single observed wave is therefore assigned to four superimposed, near‐degenerate one‐electron oxidations, and the apparent formal potential $E_{0}^{0/+4}$ approximates the mean one‐electron oxidation energy of the individual phenothiazine subunits; the nonresolution of the individual events is itself a consequence of the negligible interdonor coupling rather than a limitation of the measurement. Reversibility is confirmed for all compounds by scan‐rate‐dependent measurements (100–1000 mV s^−1^), which show linear dependence of peak currents on the square root of the scan rate. The first oxidation potentials span the range of +0.17 to +0.42 V, depending on the substitution pattern and electronic nature of the phenothiazine scaffold. The cyclic voltammograms of compounds **5g** and **5n** are shown in Figure 3A as representative examples spanning the full range of observed redox behavior within the 7‐substituted series. Cyclic voltammograms of all remaining compounds are provided in the Supporting Information.

**TABLE 1:** Electrochemical properties of tetrakis(phenothiazinyl)silanes **5**–**8**.^a^

compound ^b^		$E_{0}^{0/+4}$ / V ^a^	$E_{0}^{+4/+8}$ / V ^a^	*K*_c_ ^b^
10‐position	**5a**	0.29	—^c^	—
	**5b**	0.32	—^c^	—
	**5c**	0.28	—^c^	—
	**5d**	0.32	—^c^	—
	**5e**	0.32	—^c^	—
	**5f**	0.23	—^c^	—
7‐position	**5g**	0.30	—^c^	—
	**5h**	0.26	—^c^	—
	**5i**	0.25	—^c^	—
	**5j**	0.24	—^c^	—
	**5k**	0.23	0.88	8.97 × 10^10^
	**5l**	0.22	0.83	2.42 × 10^10^
	**5m**	0.21	0.80	9.99 × 10^9^
	**5n**	0.17	0.35	1.08 × 10^3^
2‐/1‐position	**5o**	0.22	—^c^	—
	**5p**	0.18	—^c^	—
	**5q**	0.21	—^c^	—
	**5r**	0.42	—^c^	—
linker	**6**	0.32	—^c^	—
	**7**	0.28	—^c^	—
	**8**	0.19	—^c^	—

^a^ Recorded in CH_2_Cl_2_, *T* = 293 K, *v* = 100 mV s^−1^; electrolyte: [*^n^* Bu_4_N][PF_6_], *c* = 0.1 m; Pt working electrode, Pt counter electrode, Ag/AgCl reference electrode; *E*_0_ values (formal potentials) were approximated as $E_{1/2}=\frac{\left(E_{\mathrm{pa}}+E_{\mathrm{pc}}\right)}{2}$ for reversible waves. Potentials are reported vs. Fc/Fc^+^ and were internally calibrated using Fc*/Fc*^+^ ($E_{1/2}$ = −0.54 V vs. Fc/Fc^+^).

^b^ Apparent comproportionation constant $K_{c}=10^{\frac{E_{0}^{+4/+8\;}-\;\;E_{0}^{0/+4}}{0.058\;V}}.$

^c^ Outside the measurement range.

**FIGURE 3:**
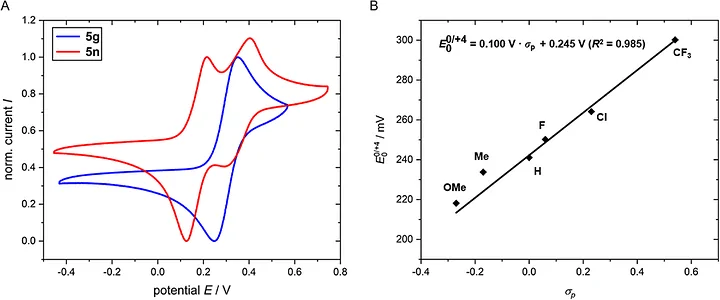
(A) Representative cyclic voltammograms of compounds **5g** (blue) and **5n** (red), recorded in CH_2_Cl_2_, *T* = 293 K, *v* = 100 mV s^−1^, electrolyte [^n^Bu_4_N][PF_6_], *c* = 0.1 m; Pt working electrode, Pt counter electrode, Ag/AgCl reference electrode, Fc*/Fc*^+^ ($E_{0}^{0/+1}$ = −0.54 V) as a redox standard (vs. Fc/Fc^+^: $E_{0}^{0/+1}$= 0.00 V). (B) Linear correlation between the first oxidation potentials $E_{0}^{0/+4}$ and the Hammett substituent constant *σ*_p_ [45] for 7‐substituted compounds **5g**–**l** (variation of substituent *R* ^2^). The regression follows the equation $E_{0}^{0/+4}$ = 0.100 V. *σ*_p_ + 0.245 V (*R* ^2^ = 0.985).

Variation at the 10‐position of the phenothiazine unit is explored to probe the influence of *N*‐substituents on the oxidation potential (**5a**–**f**). Modification of the alkyl chain (**5a**–**d**) results in minimal changes in $E_{0}^{0/+4}$ (0.28–0.32 V), consistent with the similar inductive character of saturated substituents. A similarly high oxidation potential is observed for the *N*‐benzyl derivative **5e** (0.32 V), where the methylene spacer effectively decouples the aromatic ring from the nitrogen lone pair. In contrast, compound **5f**, which bears a 4‐methoxyphenyl (anisyl) group at the 10‐position and a 4‐fluorophenyl substituent at the 7‐position, exhibits the lowest oxidation potential within this series (+0.23 V). The observed oxidation potential of **5f** thus reflects a combined contribution of both the 7‐ and 10‐substituent. A direct comparison with the 7‐fluorophenyl‐substituted reference compound **5i** (+0.25 V), which carries a hexyl group at the 10‐position, reveals that the additional replacement of the *N*‐hexyl by an *N*‐anisyl group results in a further cathodic shift of 20 mV.

To investigate the electronic fine‐tuning capability of 7‐substitution, a series of derivatives bearing electronically diverse aryl and heteroaryl groups at the 7‐position is evaluated (**5g**–**n**). The oxidation potentials range from +0.17 to +0.30 V, revealing a clear structure–property relationship: electron‐donating substituents such as methyl (**5k**, +0.23 V) or methoxy (**5l**, +0.22 V) lower the oxidation potential, while electron‐withdrawing groups such as chloro (**5h**, +0.26 V) or trifluoromethyl (**5g**, +0.30 V) shift the potential anodically. To identify the most appropriate electronic descriptor, $E_{0}^{0/+4}$ can be correlated with several established Hammett substituent constants, including *σ*_p_, *σ*_p_^+^, and *σ*_p_^−^, as well as the single‐component Swain–Lupton field (*F*) and resonance (*R*) parameters [45]. The best correlation is obtained with *σ*_p_ (*R* ^2^ = 0.985) (Figure 3B), while *σ*_p_^−^ (*R* ^2^ = 0.972) and *σ*_p_^+^ (*R* ^2^ = 0.908) provide progressively poorer fits. The purely inductive (*F*) and purely resonance‐based (*R*) descriptors fail to produce satisfactory correlations, indicating that neither field nor resonance effects alone account for the observed trend. The superior performance of *σ*_p_ is consistent with the structural arrangement of the 7‐substituted series, in which the electronically varied substituents occupy the *para*‐position of a pendant phenyl ring attached to *C*7 of the phenothiazine. In this topology, the substituent modulates the electron density at the redox‐active phenothiazine unit indirectly through the intervening phenylene spacer, a scenario for which *σ*_p_ provides the physically appropriate descriptor. The poorer correlation with *σ*_p_^+^ confirms that the substituent does not directly stabilize the phenothiazine radical cation through resonance [10]. Compounds **5m** and **5n** have to be excluded from the linear regression, as their 7‐substituents, 5‐methylthiophen‐2‐yl, and 10‐hexyl‐10*H*‐phenothiazin‐3‐yl, respectively, lack established *σ*_p_ parameters. Their oxidation potentials nevertheless fall below the extrapolated regression line, consistent with the strongly electron‐donating character of both heterocyclic substituents and confirming the qualitative trend. For the most electron‐rich members of this series (**5k**–**n**), a second reversible oxidation process ($E_{0}^{+4/+8}$) can be observed within the accessible potential window. For compounds **5k**–**m**, this second wave appears in a narrow range between +0.80 and +0.88 V and corresponds to the formation of the phenothiazine dication at the same redox‐active units. The electron‐donating substituents stabilize the initially formed radical cation sufficiently to render this second oxidation accessible under the measurement conditions. The associated comproportionation constants are large (*K*_c_ ≈ 10^10^), indicating a high thermodynamic stability of the intermediate radical cation state and a pronounced energetic separation between the two successive oxidation events. Compound **5n** exhibits a distinctly different behavior: the second oxidation appears at a markedly lower potential (+0.35 V), resulting in a dramatically reduced comproportionation constant (*K*_c_ = 1.08 · 10^3^). This reflects the unique architecture of compound **5n**, which features four biphenothiazine‐based arms comprising a total of eight redox‐active phenothiazine units. Here, the second oxidation event does not correspond to dication formation on the same phenothiazine core but rather to the oxidation of a further electronically coupled phenothiazine subunit within each arm. The low *K*_c_ value is consistent with the relatively strong electronic coupling between two adjacent phenothiazine units within a single biphenothiazine arm [30, 35, 46].

Donor‐functionalized derivatives bearing substituents at the 2‐position (**5o**–**q**) were examined to assess the effect of substitution at a site in direct conjugation with the 1,4‐thiazine π‐system. Compared to the unsubstituted reference compound **5c** (+0.28 V), all 2‐substituted derivatives exhibit cathodically shifted oxidation potentials (+0.18 to +0.22 V), reflecting the electronic activation of the phenothiazine unit by electron‐donating groups at a conjugated position. Notably, the 2‐methoxy derivative **5p** (+0.18 V) shows the strongest cathodic shift within this subset, consistent with the pronounced +*M*‐effect of the methoxy group. In contrast, the regioisomeric compound **5r**, in which the phenothiazine is connected through the 1‐position rather than the 3‐position, exhibits the most anodically shifted oxidation potential among all target tetramers (+0.42 V). This pronounced shift of +0.14 V relative to the 3‐connected analogue **5c** reflects the diminished conjugative coupling between the phenothiazine unit and the pendant linker at C1 compared to C3, resulting in a more localized HOMO and a correspondingly higher ionization energy. A detailed rationalization based on the computed torsion angles and NTO topologies is provided in the DFT section below.

To investigate the influence of the linker motif on the redox behavior, a series of derivatives was examined in which the phenothiazine units are connected to the central silicon atom either directly (**6**, +0.32 V), through a phenylene spacer (**5c**, +0.28 V), a thienylene spacer (**7**, +0.28 V), or a combined phenylene–thienylene linker (**8**, +0.19 V). The oxidation potential decreases systematically with increasing π‐conjugation between the donor units and the silicon core. Although the directly connected system **6** exhibits the highest oxidation potential, reflecting the absence of any conjugation pathway beyond the phenothiazine itself, the introduction of a phenylene (**5c**, +0.28 V) or thienylene spacer (**7**, +0.28 V) leads to an identical cathodic shift of 40 mV. The combination of both linker units in compound **8** results in a further substantial decrease to +0.19 V, reflecting the cumulative effect of the extended conjugation pathway on the HOMO energy. These data demonstrate that structural modification at the linker site provides an effective and complementary means of tuning the redox properties of the tetrameric framework, in addition to the substituent‐based modulation at the 7‐ or 2‐position.

### 2.4 Photophysical Properties

Photophysical properties were evaluated in CH_2_Cl_2_ solution and in the solid state to elucidate structure–property relationships within the tetrakis(phenothiazinyl)silane series. UV–vis absorption and fluorescence emission spectra were recorded under standardized conditions, and absolute fluorescence quantum yields (integrating sphere) as well as excited‐state lifetimes (TCSPC) were determined (Table 2).

**TABLE 2:** Photophysical properties of tetrakis(phenothiazinyl)silanes **5**–**8**.

compound		*λ*_max, abs_ / nm (*ε* / L ^.^ mol*^−^* ^1.^ cm*^−^* ^1^)^a^	*λ*_max, em_ / nm (*Φ*_F_)^b^	Δ*ν̃* / cm*^−^* ^1^ ^c^	*τ*_F_ */* ns ^d^	*k*_r_ / 10^7^ s*^−^* ^1^ ^e^	*k*_nr_ / 10^7^ s*^−^* ^1^ ^f^	*λ*_max, em_ / nm (*Φ_F_*)^g^
10‐position	**5a**	238 (79,900), 268 (137,100), 318 (45,700)	477 (0.61)	10,500	5.97	10.22	6.53	479 (0.55)
	**5b**	241 (86,000), 271 (138,400), 321 (40,700)	478 (0.62)	10,200	5.95	10.42	6.39	471 (0.66)
	**5c**	241 (117,600), 272 (190,300), 323 (50,500)	476 (0.50)	10,000	5.92	8.45	8.45	470 (0.47)
	**5d**	242 (112,600), 272 (171,000), 324 (59,400)	482 (0.69)	10,100	5.70	12.11	5.44	—^h^
	**5e**	238 (94,600), 270 (149,200), 327 (43,400)	473 (0.79)	9400	5.78	13.67	3.63	468 (0.19)
	**5f**	285 (305,300), 351 (62,800)	473 (0.82)	7300	5.36	15.30	3.36	505 (0.18)
7‐position	**5g**	241 (127,200), 287 (205,500), 342 (108,800)	493 (0.90)	9000	5.66	15.90	1.77	492 (0.77)
	**5h**	245 (151,900), 285 (264,000), 341 (77,500)	483 (0.76)	8600	5.49	13.84	4.37	487 (0.30)
	**5i**	242 (127,900), 281 (229,500), 337 (65,500)	480 (0.72)	8800	5.41	13.31	5.18	476 (0.12)
	**5j**	241 (109,700), 282 (193,700), 338 (55,800)	482 (0.79)	8800	5.40	14.63	3.89	490 (0.15)
	**5k**	248 (129,300), 283 (237,600), 339 (65,500)	484 (0.86)	8800	5.22	16.48	2.68	490 (0.77)
	**5l**	282 (211,300), 339 (54,900)	485 (0.82)	8900	5.06	16.21	3.56	514 (0.61)
	**5m**	291 (267,800), 352 (96,100)	486 (0.53)	7800	4.45	11.91	10.56	521 (0.17)
	**5n**	236 (182,600), 284 (314,000), 361 (45,600)	486 (0.58)	7100	3.33	17.42	12.61	513 (0.36)
2‐/1‐position	**5o**	239 (92,400), 264 (135,200), 316 (33,900)	491 (0.38)	11,300	4.95	7.68	12.53	468 (0.33)
	**5p**	263 (143,800), 328 (43,500)	477 (0.85)	9500	5.46	15.57	2.75	475 (0.67)
	**5q**	272 (182,000), 324 (34,300)	485 (0.41)	10,200	5.17	7.93	11.41	473 (0.34)
	**5r**	259 (147,000), 316 (27,100)	472 (0.08)	10,500	2.18	3.67	42.20	461 (0.06)
linker	**6**	266 (201,300), 316 (35,800)	441 (0.11)	9000	4.91	2.24	18.13	442 (0.16)
	**7**	246 (79,200), 300 (89,700), 366 (59,200)	507 (0.71)	7600	5.82	12.20	4.98	501 (0.40)
	**8**	241 (102,400), 332 (104,900), 385 (111,100)	533 (0.78)	7200	4.00	19.50	5.50	529 (0.29)

^a^ Recorded in CH_2_Cl_2_, *T* = 293 K, *c* = 10*^−^* ^5^ m.

^b^ Recorded in CH_2_Cl_2_, *T* = 293 K, *c* = 10*^−^* ^7^ m; absolute quantum yields determined using an integrating sphere, $\lambda_{ex}=\lambda_{\max,abs}$.

^c^ $\Delta \;\tilde{\nu \;}=\frac{1}{\lambda_{\max,\;abs}}\;-\;\frac{1}{\lambda_{\max,\;em}}$, (solution).

^d^ Recorded in CH_2_Cl_2_, *T* = 293 K, *c* = 10*^−^* ^7^ m.

^e^ $k_{r}=\frac{\Phi_{F}}{\tau_{F}}$.

^f^ $k_{\mathrm{nr}}=\frac{1-\Phi_{F}}{\tau_{F}}$.

^g^ Recorded in the solid state, *T* = 293 K; absolute quantum yields determined using an integrating sphere, $\lambda_{\mathrm{ex}}=\lambda_{\max,\mathrm{abs}}$.

^h^ Compound **5d** is a resin.

All compounds exhibit fluorescence in both media, with emission maxima spanning the blue to yellow–green region and quantum yields ranging from *Φ*_F_ = 0.08–0.90 in solution and 0.06–0.77 in the solid state (Figure 4A). Across the series, solid‐state quantum yields are generally lower than in solution, consistent with enhanced nonradiative decay through intermolecular contacts in the condensed phase. This range of quantum yields has two distinct origins. Part of it reflects intrinsically weak emitters: **5r** and **6** are only weakly fluorescent in solution as well (*Φ*_F_ = 0.08 and 0.11), their low efficiencies arising from molecular factors – the C1‐induced inter‐ring torsion in **5r** and the low radiative rate and elevated nonradiative decay of the directly linked π‐system in **6** – rather than from the solid‐state environment. The remaining, solid‐state‐specific reductions affect compounds that are strongly emissive in solution, and their solid‐state efficiency does not track the solution values: **5** **g** and **5k** (*Φ*_F_ = 0.90 and 0.86 in solution) retain *Φ*_F_ = 0.77 in the solid state, whereas the similarly emissive **5e**, **5f** and **5j** (*Φ*_F_ = 0.79–0.82) drop to 0.15–0.19. This decorrelation indicates that the solid‐state variation is governed by intermolecular packing and morphology rather than by the intrinsic electronic structure of the chromophores. A compound‐specific structural rationalization is not accessible, since only **5a** afforded single crystals suitable for diffraction, the remaining derivatives being isolated as powders for which no single‐crystal structure could be obtained. For **5a**, the Hirshfeld analysis reveals no π⋯π or other directional interchromophore contacts, in line with its essentially retained solid‐state quantum yield (*Φ*_F_ = 0.55, vs. 0.61 in solution).

**FIGURE 4:**
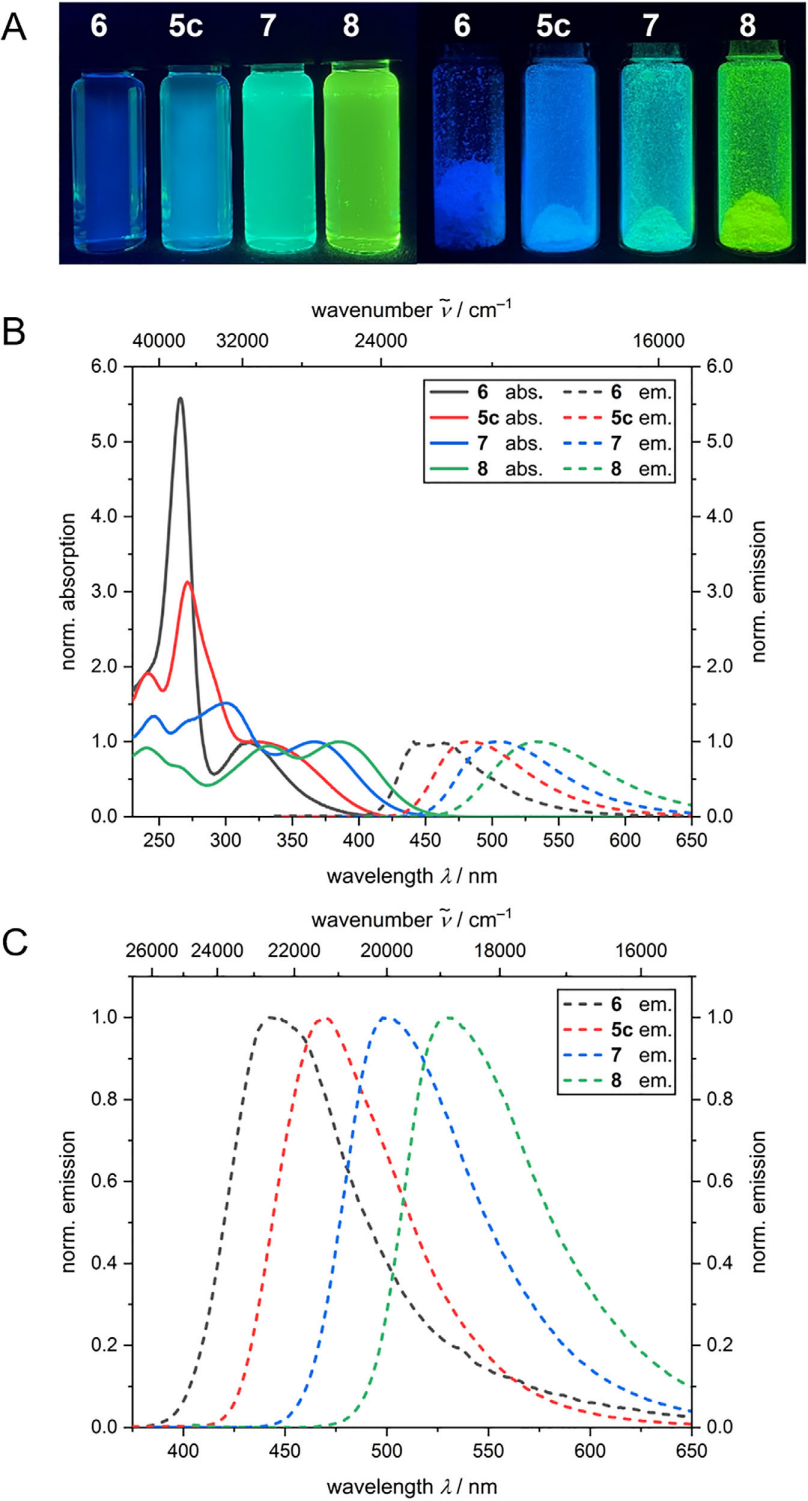
A) Emission of selected compounds **6**, **5c**, **7**, and **8** in solution (left; in CH_2_Cl_2_ (*c* = 10^−5^ m)) and in the solid state (right) under the hand‐held UV lamp (*λ*_exc_ = 365 nm). (B) Normalized UV–vis absorption (solid lines; recorded in CH_2_Cl_2_, *T* = 293 K, *c* = 10^−5^ m) and emission spectra (dashed lines; recorded in CH_2_Cl_2_, *T* = 293 K, *c* = 10^−7^ m) of the linker series: compounds **6** (no linker), **5c** (phenylene), **7** (thienylene), and **8** (phenylene–thienylene). C: Normalized emission spectra (dashed lines; recorded in the solid state, *T*  =  293 K) of the linker series: compounds **6** (no linker), **5c** (phenylene), **7** (thienylene), and **8** (phenylene–thienylene).

Variation of the *N*‐substituent at position 10 (**5a**–**f**) exerts only a minor influence on the photophysical properties, consistent with the weak electronic coupling of this position to the phenothiazine π‐system. The lowest‐energy absorption maxima fall in a narrow range (318–327 nm; *ε* ≈ 4–6 · 10^4^ L mol^−1^ cm^−1^) for the alkyl‐ and benzyl‐substituted derivatives **5a**–**e**, and the emission maxima vary only slightly in both solution (473–482 nm) and solid state (468–479 nm). Stokes shifts within this subset are large (9400–10500 cm^−1^), characteristic of phenothiazine‐based chromophores in which a pronounced geometric reorganization from the butterfly‐folded ground‐state geometry toward a more planarized *S*_1_ minimum occurs upon photoexcitation [47, 48, 49], as confirmed by the (TD‐)DFT analysis discussed below. Fluorescence quantum yields range from 0.50 to 0.82 in solution and exceed those reported for representative monomeric phenothiazines under comparable conditions (*Φ*_F_ ≈ 0.15–0.25) [10, 35, 50]. This enhancement was verified directly by comparison with the corresponding monomeric chromophore, 10‐hexyl‐3‐phenyl‐10*H*‐phenothiazine (**9**), which constitutes a single arm of **5c** (Figure 5). Under identical conditions, the monomer **9** exhibits a solution quantum yield of only *Φ*_F_ = 0.24 (*τ*_F_ = 4.88 ns), compared with *Φ*_F_ = 0.50 (*τ*_F_ = 5.92 ns) for **5c**. The rate‐constant analysis assigns this twofold increase to both a reduced nonradiative rate (*k*_nr_ decreasing from 15.57 to 8.45 · 10^7^ s^−1^) and an increased radiative rate (*k*_r_ rising from 4.92 to 8.45 · 10^7^ s^−1^): the rigid tetrahedral scaffold restricts the conformational and torsional motion of the phenothiazine subunits and thereby suppresses nonradiative decay, while the increased radiative rate accompanies the somewhat more extended π‐system of the silane‐bound arm, reflected in the small bathochromic shifts of absorption and emission (319 → 323 and 464 → 476 nm). Consistently, the absorption bands of **5c** are approximately four times as intense as those of the monomer, indicating that the four arms behave as essentially independent chromophores.

**FIGURE 5:**
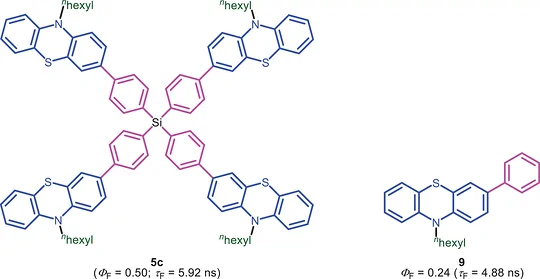
Comparison of fluorescence quantum yield *Φ*_F_ and fluorescence lifetime *τ*_F_ between tetrakis(phenothiazinyl)silane **5c** and the constituting chromophore monomer **9** (recorded in CH_2_Cl_2_, *T* = 293 K, *c* = 10*^−^* ^7^ m; absolute quantum yields determined using an integrating sphere).

The 7‐substituted derivatives (**5g**–**n**) show modest shifts of the lowest‐energy absorption bands (337–361 nm) and a confined solution‐emission window (480–493 nm), indicating that C7‐substitution modulates the lowest‐energy transition energy only weakly. This is consistent with largely phenothiazine‐centered, locally excited states in which the 7‐substituent perturbs the electronic structure indirectly through the pendant phenylene spacer. No monotonic dependence of *λ*_max, abs_ or *λ*_max, em_ on the Hammett parameter *σ*_p_ is apparent, in contrast to the clear correlation observed for the oxidation potentials. Fluorescence quantum yields are high across this subset (*Φ*_F_ = 0.53–0.90 in solution), with **5g** (CF_3_, 0.90) achieving the highest value. In the solid state, larger variations are observed: while **5g** and **5k** retain high quantum yields (0.77), other members such as **5i** (0.12) and **5j** (0.15) show substantially reduced values, indicating that solid‐state packing effects rather than intrinsic electronic properties dominate the fluorescence efficiency in the condensed phase. The excited‐state lifetimes decrease systematically from 5.66 ns (**5g**) to 3.33 ns (**5n**). A quantitative analysis of the rate constants reveals that *k*_r_ remains on a comparable scale across the series (11.9–17.4 · 10^7^ s^−1^), while *k*_nr_ increases markedly from 1.77 · 10^7^ s^−1^ (**5g**) to 12.61 · 10^7^ s^−1^ (**5n**). The reduced quantum yields and shortened lifetimes observed for the most extended systems **5m** and **5n** are thus primarily attributable to enhanced non‐radiative deactivation rather than a decrease in radiative rates.

Derivatives bearing donor substituents at the 2‐position (**5o**–**q**) show only moderate changes in spectral positions relative to the reference compound **5c**: the lowest‐energy absorption maxima remain in the range of 316–328 nm, and the solution emission maxima span 477–491 nm. Compound **5o** exhibits the most red‐shifted solution emission (491 nm) and the largest Stokes shift within the entire series (11300 cm^−1^). Fluorescence quantum yields within this subset vary considerably (*Φ*_F_ = 0.38–0.85), with **5p** (2‐OMe, 0.85) achieving the highest value and **5o** (2‐Me, 0.38) the lowest. The rate constant analysis shows that the low quantum yield of **5o** is associated with elevated non‐radiative decay (*k*_nr_ = 12.53 · 10^7^ s^−1^), while **5p** combines a high radiative rate (*k*_r_ = 15.57 · 10^7^ s^−1^) with efficient suppression of non‐radiative pathways (*k*_nr_ = 2.75 · 10^7^ s^−1^).

Changing the point of attachment from C3 to C1 has a pronounced impact on fluorescence efficiency. The regioisomeric compound **5r** exhibits absorption and emission maxima close to those of **5c** (*λ*_max, abs_ = 316 nm; *λ*_max, em_ = 472 nm), yet its fluorescence is strongly attenuated (*Φ*_F_ = 0.08 in solution, 0.06 in solid state) and its lifetime is markedly shortened (*τ*_F_ = 2.18 ns). The combination of largely unchanged spectral positions but drastically reduced *Φ*_F_ and *τ*_F_ is reflected in the rate constants: *k*_r_ drops to 3.67 · 10^7^ s^−1^ while *k*_nr_ rises to 42.20 · 10^7^ s^−1^, indicating that C1‐linkage accelerates nonradiative decay by approximately fivefold relative to **5c** while simultaneously reducing the radiative rate. In line with the electrochemical data, which showed the most anodic oxidation potential for **5r**, C1‐linkage diminishes effective electronic communication between the phenothiazine subunit and the conjugated framework, thereby promoting nonradiative decay and identifying attachment topology as a critical and independently tunable parameter for excited‐state deactivation.

Varying the linker architecture exerts the strongest influence on the optical response within this tetramer series (Figure 4B,C). Compared with peripheral substitution on the phenothiazine scaffold, changes at the linker translate into pronounced shifts in both absorption and emission, consistent with an effective extension of the π‐conjugation pathway connecting the phenothiazine donors to the silicon core. The directly connected tetramer **6** (no π‐linker) displays the most hypsochromically shifted spectra, with a lowest‐energy absorption maximum at 316 nm, and emits in the deep‐blue region. Notably, compound **6** exhibits a vibronically structured emission with two nearly equally intense maxima at 441 and 464 nm, assignable to the 0–0 and 0–1 transitions. This vibronic fine structure reflects the localized nature of the emitting state, in which the electronic transitions couple to well‐defined vibrational modes of the unperturbed phenothiazine chromophore in the absence of any conjugation‐extending linker. In parallel, **6** displays the lowest fluorescence quantum yield in solution (*Φ*_F_ = 0.11), and the rate constant analysis confirms that this is dominated by a dramatically elevated non‐radiative rate (*k*_nr_ = 18.13 · 10^7^ s^−1^) combined with a low radiative rate (*k*_r_ = 2.24 · 10^7^ s^−1^). The absence of π‐extension preserves conformational flexibility in the excited state, opening efficient non‐radiative relaxation pathways. Introducing an aromatic spacer progressively red‐shifts the spectra: the phenylene‐linked reference **5c** emits at 476 nm and the thienylene‐linked analogue **7** at 507 nm in solution, while combining both linker units in **8** affords the most red‐shifted member, with absorption extending to 385 nm and emission at 533 nm (solution) and 529 nm (solid state), corresponding to yellow–green luminescence (Figure 4A). Importantly, substantial π‐extension does not compromise solution‐phase fluorescence efficiency: **7** (*Φ*_F_ = 0.71) and **8** (*Φ*_F_ = 0.78) retain high quantum yields despite their strongly red‐shifted emission. Compound **8** achieves the highest radiative rate within the entire series (*k*_r_ = 19.50 · 10^7^ s^−1^), demonstrating that extended conjugation enhances the oscillator strength of the emissive transition. The vibronic structure observed for **6** vanishes upon introduction of any linker, yielding single, broad emission bands for **5c**, **7**, and **8**, consistent with the transition from a localized to a delocalized emitting state.

### 2.5 Calculated Electronic Structure

For a deeper understanding of the underlying electronic structure, (TD‐)DFT calculations were performed with Gaussian 16 [51], using the CAM‐B3LYP functional and the def2‐SVP basis set. Dispersion effects were accounted for using Grimme's D3 correction with Becke–Johnson damping (GD3BJ). Frequency calculations confirmed all optimized structures as true minima (no imaginary frequencies). Solvent effects (CH_2_Cl_2_) were included via the SMD model. To reduce computational cost, alkyl chains longer than ethyl were truncated to ethyl substituents; this approximation is justified by the negligible influence of the remote alkyl chain on the electronic structure of the phenothiazine chromophore, as confirmed by the minimal variation in photophysical and electrochemical properties across the 10‐alkyl series (**5a**–**e**). Vertical emission energies were computed at the relaxed *S*_1_ geometry using a nonequilibrium solvation treatment.

The calculated excitation and emission energies are in good agreement with the experimental data, although the emission energies are generally slightly overestimated. The corrected emission energies, obtained from the relaxed *S*_1_ geometries, reproduce the experimental values more closely (Table 3).

**TABLE 3:** (TD‐)DFT calculations on the UV–vis absorption maxima and emission maxima of compounds **5**–**8** (CAM‐B3LYP/def2‐SVP, SMD CH_2_Cl_2_, and GD3BJ).

compound		*λ*_max, abs_ / nm (*ε* / L ^.^ mol−^1^. cm−^1^)^a^	*λ*_max, calcd, comb._/ nm	*λ*_max, calcd_ / nm (oscillator strength)	*λ_max, em_*/ nm^b^	*λ*_max, em, calcd_ / nm (oscillator strength)	Most dominant contribution
10‐position	**5a**	318 (45,700)	314	315 (0.49) 315 (0.34) 314 (0.50) 311 (0.01)	477	462 (0.21)	HOMO→LUMO (76%)
	**5b**	321 (40,700)	315	316 (0.53) 315 (0.46) 315 (0.40) 312 (0.04)	478	458 (0.23)	HOMO→LUMO (76%)
	**5c**	323 (50,500)	334	336 (0.34) 336 (0.34) 335 (0.42) 332 (0.01)	476	465 (0.20)	HOMO→LUMO (76%)
	**5d**	324 (59,400)	334	336 (0.34) 336 (0.34) 335 (0.42) 332 (0.01)	482	465 (0.20)	HOMO→LUMO (76%)
	**5e**	327 (43,400)	326	327 (0.31) 327 (0.41) 326 (0.36) 323 (0.02)	473	456 (0.20)	HOMO→LUMO (77%)
	**5f**	351 (62,800)	338	338 (0.70) 338 (0.32) 337 (0.56) 335 (0.06)	473	465 (0.21)	HOMO→LUMO (76%)
7‐position	**5g**	342 (108,800)	330	331 (0.31) 330 (0.35) 330 (0.34) 328 (<0.01)	493	454 (0.1597)	HOMO→LUMO (73%)
	**5h**	341 (77,500)	349	349 (0.43) 349 (0.54) 348 (0.93) 346 (0.03)	483	467 (0.32)	HOMO→LUMO (86%)
	**5i**	337 (65,500)	346	347 (0.42) 347 (0.50) 346 (0.83) 343 (0.02)	480	464 (0.29)	HOMO→LUMO (83%)
	**5j**	338 (55,800)	347	348 (0.65) 347 (0.61) 347 (0.38) 344 (0.14)	482	465 (0.32)	HOMO→LUMO (83%)
	**5k**	339 (65,500)	346	348 (0.45) 347 (0.53) 346 (0.90) 344 (0.03)	484	468 (0.34)	HOMO→LUMO (82%)
	**5l**	339 (54,900)	346	347 (0.46) 346 (0.56) 346 (0.94) 343 (0.02)	485	468 (0.36)	HOMO→LUMO (80%)
	**5m**	352 (96,100)	354	356 (0.44) 355 (0.73) 354 (1.13) 351 (0.04)	486	469 (0.38)	HOMO→LUMO (86%)
	**5n**	361 (45,600)	353	354 (0.97) 353 (0.64) 353 (1.01) 350 (0.25)	486	466 (0.49)	HOMO→LUMO (80%)
2‐/1‐position	**5o**	316 (33,900)	328	329 (0.18) 329 (0.16) 327 (0.21) 326 (0.05)	491	445 (0.09)	HOMO→LUMO (71%)
	**5p**	328 (43,500)	308	310 (0.54) 308 (0.46) 308 (0.64) 305 (0.05)	477	449 (0.1870)	HOMO→LUMO (77%)
	**5q**	324 (34,300)	333	334 (0.12) 333 (0.12) 333 (0.10) 332 (0.01)	485	467 (0.0535)	HOMO→LUMO (80%)
	**5r**	316 (27,100)	295	300 (0.11) 298 (0.11) 298 (0.09) 297 (0.06)	472	477 (0.03)	HOMO→LUMO (71%)
linker	**6**	316 (35,800)	326	328 (0.06) 327 (0.10) 325 (0.11) 323 (0.01)	441	446 (0.01)	HOMO→LUMO (87%)
	**7**	366 (59,200)	348	349 (0.46) 349 (0.66) 348 (0.75) 341 (0.01)	507	489 (0.34)	HOMO→LUMO (83%)
	**8**	385 (111,100)	360	365 (1.30) 361 (1.83) 360 (1.00) 354 (0.06)	533	508 (1.19)	HOMO→LUMO (86%)

^a^ Recorded in CH_2_Cl_2_, *T* = 293 K, *c* = 10*^−^* ^5^ m.

^b^ Recorded in CH_2_Cl_2_, *T* = 293 K, *c* = 10*^−^* ^7^ m.

The calculations further show that, for all structures, the longest‐wavelength absorption band consists of four closely spaced electronic transitions ‐ three of comparable oscillator strength together with a markedly weaker fourth ‐ , which collectively account for the broad, featureless absorption envelope observed experimentally. A representative comparison of the simulated [52] and measured absorption spectra of **5c** illustrates this agreement (Figure 6).

**FIGURE 6:**
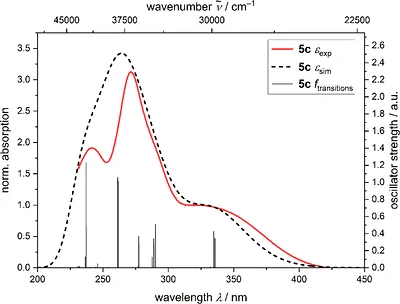
Comparison of the simulated (CAM‐B3LYP/def2‐SVP, SMD CH_2_Cl_2_, and GD3BJ) and measured (recorded in CH_2_Cl_2_, *T* = 293 K) absorption spectra of compound **5c** as a representative example. Simulated spectra data were generated from Gaussian 16 output using Multiwfn [52].

Because of the high number of electronic contributions involved, natural transition orbitals (NTOs) [52] were used to analyze these transitions. The analysis reveals all four excitations to be localized on the phenothiazine units, with the transitions either confined to a single phenothiazine or shared between two. In one limiting case, two transitions are each localized on a single phenothiazine unit, while the remaining two are distributed across the other two phenothiazines. In the other limiting case, three transitions are localized on separate phenothiazine units, while the fourth is shared between the remaining phenothiazine and an adjacent unit. These two limiting cases are exemplified by compounds **6** and **8**, respectively (Figure 7). Most structures fall between these two extremes, with a gradual shift toward the latter limiting case as the size of the π‐system increases. Across all compounds, the electron density of each transition extends over the entire π‐system of the respective phenothiazine arm, and all transitions exhibit a pronounced locally excited character. This explains the minor influence of the 7‐substitution pattern on the absorption and emission energies, as the substituent modulates the electronic structure of the phenothiazine unit only indirectly through the pendant phenylene spacer.

**FIGURE 7:**
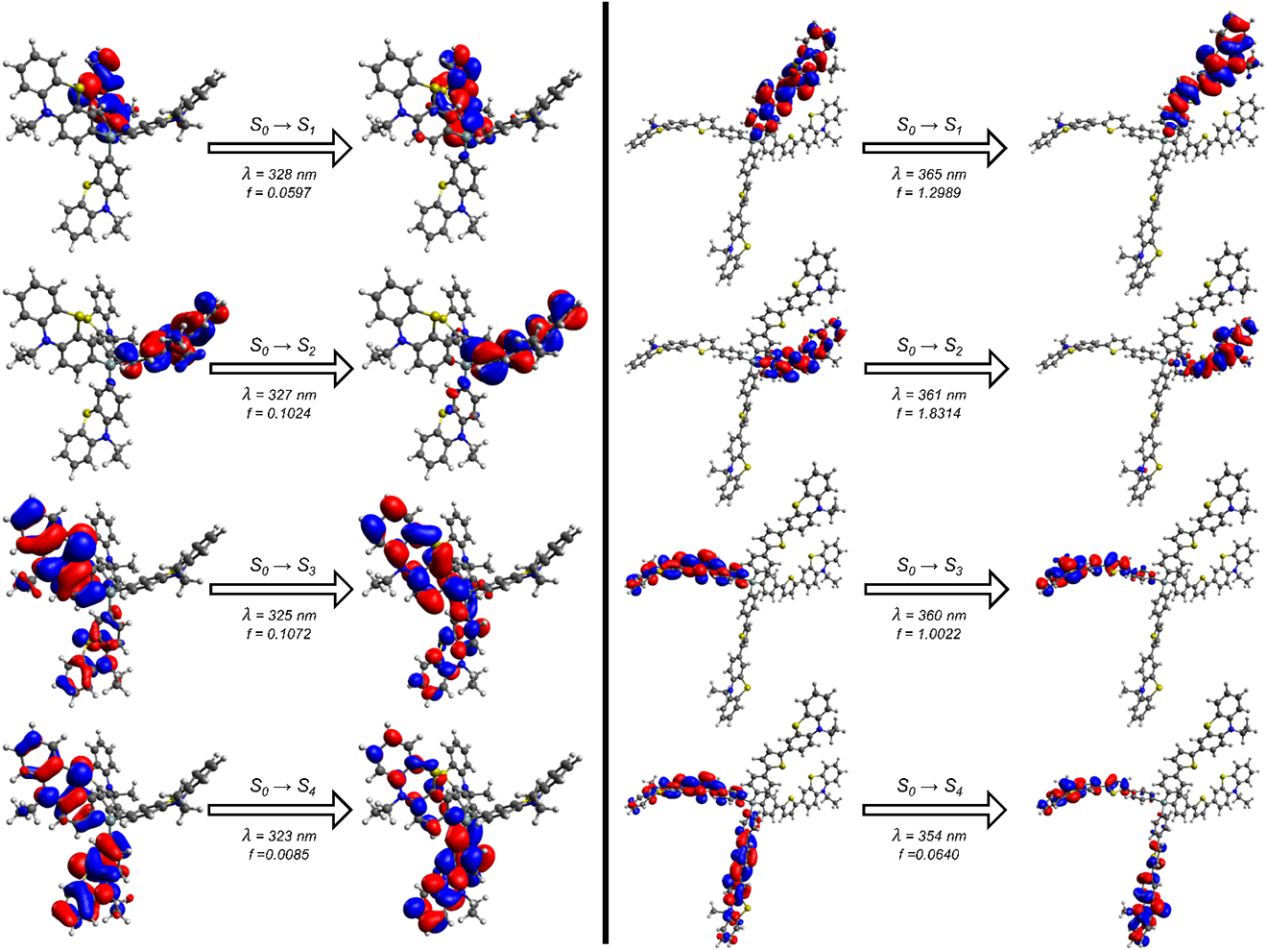
Natural transition orbitals (NTOs) for the first four transitions of compound **6** (left) and **8** (right). The wavelength and oscillator strength are listed for each transition (CAM‐B3LYP/def2‐SVP, SMD CH_2_Cl_2_, and GD3BJ). NTOs were generated from Gaussian 16 using Multiwfn [52].

The NTO analysis of the C1‐connected regioisomer **5r** provides direct insight into the role of topology. In contrast to the C3‐linked derivatives, in which the hole NTO extends from the phenothiazine into the conjugated linker with moderate inter‐ring torsion (32°–33°), the C1‐linkage in **5r** induces a substantially larger torsion angle between the phenothiazine and the phenylene linker (48°–55°). This enhanced torsion diminishes the orbital overlap between the two π‐systems, consistent with the experimentally observed higher oxidation potential and the dramatically accelerated non‐radiative decay for this compound.

After the vertical Franck–Condon transition to a vibrationally excited *S*_1_ state, relaxation occurs to the vibrationally relaxed *S*_1_ minimum under planarization of one of the four phenothiazine units, while the folding angles along the *S*–*N* axis of the remaining three phenothiazines remain virtually unchanged. The planarization toward an sp^2^ nitrogen geometry is a well‐established feature of phenothiazine photophysics and is virtually independent of the substitution pattern [9]. The large geometric change relative to the ground‐state geometry accounts for the pronounced Stokes shifts of 7100–11300 cm^−1^ observed across the series, characteristic of phenothiazine‐based chromophores [9]. The emission for all compounds is localized on the planarized phenothiazine unit and has a substantial HOMO→LUMO contribution between 71% and 87%.

The calculated oscillator strengths of the *S*_1_→*S*_0_ emission span nearly two orders of magnitude across the series, from 0.01 (**6**) to 1.19 (**8**). According to the Strickler–Berg relation, the radiative rate constant *k*_r_ is directly proportional to the oscillator strength of the emissive transition [53]. The extremely low calculated oscillator strength for the directly connected tetramer **6** is thus fully consistent with the experimentally determined low radiative rate (*k*_r_ = 2.24 · 10^7^ s^−1^) and the correspondingly low fluorescence quantum yield (*Φ*_F_ = 0.11). Conversely, compound **8**, which combines the largest π‐system with the highest calculated oscillator strength, exhibits the highest experimental radiative rate within the series (*k*_r_ = 19.50 · 10^7^ s^−1^). This quantitative agreement between the computed oscillator strengths and the experimental radiative rates across the linker series validates the (TD‐)DFT approach and confirms that the variation in fluorescence efficiency is rooted in the electronic structure of the emissive state.

The computational results provide a physical rationale for the different sensitivity of electrochemical and photophysical properties to 7‐substitution. Although the ground‐state oxidation potentials exhibit a clear Hammett correlation, reflecting the susceptibility of the formally charged species to electronic stabilization through the pendant aryl substituent, the excited‐state transition energies vary only weakly across the 7‐substituted series (480–493 nm). This difference is consistent with the predominantly locally excited character of the emissive states, in which the transition density is largely confined to the phenothiazine core with limited extension into the peripheral 7‐substituents. Consequently, 7‐substitution primarily serves as a tool for electronic fine‐tuning of redox potentials, while linker engineering acts as the dominant structural handle to modulate the optical gap. This largely independent tunability identifies the tetrakis(phenothiazinyl)silane scaffold as a versatile platform for the modulation of electrochemical and photophysical properties through complementary structural parameters.

## 3 Conclusion

In summary, a modular synthetic platform based on the BLEBS sequence, complemented by LiBS and direct lithiation–nucleophilic substitution protocols, provided rapid access to a library of 21 tetrakis(phenothiazinyl)silanes with systematic variation at the 10‐, 7‐ and 2‐positions of the phenothiazine scaffold, a C1‐connected regioisomeric reference, as well as four distinct linker architectures, ranging from direct Si–C connectivity to phenylene, thienylene, and combined phenylene–thienylene spacers. Single‐crystal X‐ray diffraction confirmed the undistorted tetrahedral geometry at silicon and the absence of significant intermolecular π‐stacking. Electrochemically, all compounds display fully reversible first oxidations spanning +0.17 to +0.42 V versus Fc/Fc^+^. Within the 7‐substituted subset, the oxidation potentials correlate linearly with the Hammett parameter *σ*_p_ (*R* ^2^ = 0.985), establishing quantitative electronic fine‐tuning through the pendant aryl substituent. Photophysically, the tetramers exhibit bright fluorescence with quantum yields up to *Φ*_F_ = 0.90 in solution and emission tunable from deep blue to yellow–green (441–533 nm). Linker engineering emerges as the dominant structural handle to modulate the optical gap, while C1‐linkage is identified as a critical topology that dramatically accelerates non‐radiative decay (*k*_nr_ increased fivefold) without altering the optical gap. (TD‐)DFT calculations confirm the predominantly locally excited, phenothiazine‐centered character of all emissive states, with emission arising from a single planarized phenothiazine arm. The computed oscillator strengths quantitatively reproduce the experimental variation in radiative rates across the linker series. Notably, 7‐substitution selectively modulates the redox potentials while leaving the optical gap essentially invariant, whereas linker engineering primarily governs the optical gap, identifying the tetrakis(phenothiazinyl)silane scaffold as a versatile platform for the largely independent control of electrochemical and photophysical properties. Future work will extend this approach to tetrakis(triarylamino)silanes as complementary multidonor reference systems to delineate the role of the donor motif within this modular framework.

## Conflicts of Interest

The authors declare no conflict of interest.

## Supporting information




**Supporting file 1**: chem71368‐sup‐0001‐SuppMat.pdf.
The authors have cited additional references within the Supporting Information [54, 55, 56, 57, 58, 59, 60, 61, 62, 63].

## Data Availability

The data that support the findings of this study are available from the corresponding author upon reasonable request.
