# Supplementary material for: Modular One‐Pot Access to π‐Expanded Tetrakis(Phenothiazinyl)‐Silanes With Broadly Tunable Redox and Emission Properties
Source: Chemistry. 2026 Jul 8;32(39):e71368. doi: 10.1002/chem.71368 (PMC13715450; doi:10.1002/chem.71368)
Supplement: Supplementary file 1 [file CHEM-32-e71368-s001.pdf]

Table of contents

1. General considerations – overview of experimental procedures ... 8

2. Syntheses ... 11

2.1 Synthesis of starting materials according to literature ... 11

2.2 Synthesis of non-commercial and unpublished starting materials ... 11

2.2.1 General procedure (GPI) of the BLEBS sequence for the synthesis of (hetero)arylated phenothiazine bromides **4g**-**n** ... 11

2.2.2 3-Bromo-10-hexyl-7-(4-(trifluoromethyl)phenyl)-10*H*-phenothiazine (**4g**) ... 13

2.2.3 3-Bromo-7-(4-chlorophenyl)-10-hexyl-10*H*-phenothiazine (**4h**) ... 14

2.2.4 3-Bromo-7-(4-fluorophenyl)-10-hexyl-10*H*-phenothiazine (**4i**) ... 15

2.2.5 3-Bromo-10-hexyl-7-phenyl-10*H*-phenothiazine (**4j**) ... 16

2.2.6 3-Bromo-10-hexyl-7-(*p*-tolyl)-10*H*-phenothiazine (**4k**) ... 17

2.2.7 3-Bromo-10-hexyl-7-(4-methoxyphenyl)-10*H*-phenothiazine (**4l**) ... 18

2.2.8 3-Bromo-10-hexyl-7-(5-methylthiophen-2-yl)-10*H*-phenothiazine (**4m**) ... 19

2.2.9 7-Bromo-10,10'-dihexyl-10*H*,10'*H*-3,3'-biphenothiazine (**4n**) ... 20

2.2.10 3-Bromo-7-(4-fluorophenyl)-10-(4-methoxyphenyl)-10*H*-phenothiazine (**4f**) ... 21

2.3 Synthesis of target compounds ... 22

2.3.1 General procedure (GPII) of the BLEBS sequence for the synthesis of phenyl-expanded tetrakis(phenothiazinyl)silanes 5 ... 22

2.3.2 Tetrakis(4-(10-methyl-10*H*-phenothiazin-3-yl)phenyl)silane (**5a**) ... 24

2.3.3 Tetrakis(4-(10-isopropyl-10*H*-phenothiazin-3-yl)phenyl)silane (**5b**) ... 25

2.3.4 Tetrakis(4-(10-hexyl-10*H*-phenothiazin-3-yl)phenyl)silane (**5c**) ... 26

2.3.5 Tetrakis(4-(10-(2-decyltetradecyl)-10*H*-phenothiazin-3-yl)phenyl)silane (**5d**) ... 27

2.3.6 Tetrakis(4-(10-benzyl-10*H*-phenothiazin-3-yl)phenyl)silane (**5e**) ... 28

2.3.7 Tetrakis(4-(7-(4-fluorophenyl)-10-(4-methoxyphenyl)-10*H*-phenothiazin-3-yl)phenyl)silane (**5f**) ... 29

2.3.8 Tetrakis(4-(10-hexyl-7-(4-(trifluoromethyl)phenyl)-10*H*-phenothiazin-3-yl)phenyl)silane (**5g**) ... 30

2.3.9 Tetrakis(4-(7-(4-chlorophenyl)-10-hexyl-10*H*-phenothiazin-3-yl)phenyl)silane (**5h**) . 31

2.3.10 Tetrakis(4-(7-(4-fluorophenyl)-10-hexyl-10*H*-phenothiazin-3-yl)phenyl)silane (**5i**) ... 32

2.3.11 Tetrakis(4-(10-hexyl-7-phenyl-10*H*-phenothiazin-3-yl)phenyl)silane (**5j**) ... 33

2.3.12 Tetrakis(4-(10-hexyl-7-(*p*-tolyl)-10*H*-phenothiazin-3-yl)phenyl)silane (**5k**) ... 34

2.3.13 Tetrakis(4-(10-hexyl-7-(4-methoxyphenyl)-10*H*-phenothiazin-3-yl)phenyl)silane (**5l**) ... 35

2.3.14 Tetrakis(4-(10-hexyl-7-(5-methylthiophen-2-yl)-10*H*-phenothiazin-3-yl)phenyl)silane (**5m**) 36
2.3.15 Tetrakis(4-(10,10'-dihexyl-10*H*,10'*H*-[3,3'-biphenothiazin]-7-yl)phenyl)silane (**5n**) ...37
2.3.16 Tetrakis(4-(10-hexyl-2-methyl-10*H*-phenothiazin-3-yl)phenyl)silane (**5o**) 38
2.3.17 Tetrakis(4-(10-hexyl-2-methoxy-10*H*-phenothiazin-3-yl)phenyl)silane (**5p**) 39
2.3.18 Tetrakis(4-(10-hexyl-2-(methylthio)-10*H*-phenothiazin-3-yl)phenyl)silane (**5q**) 40
2.3.19 Tetrakis(4-(10-hexyl-10*H*-phenothiazin-1-yl)phenyl)silane (**5r**)....41
2.3.20 Tetrakis(10-hexyl-10*H*-phenothiazin-3-yl)silane (**6**) 42
2.3.21 Tetrakis(5-(10-hexyl-10*H*-phenothiazin-3-yl)thiophen-2-yl)silane (**7**) 43
2.3.22 Tetrakis(4-(5-(10-hexyl-10*H*-phenothiazin-3-yl)thiophen-2-yl)phenyl)silane (**8**) 44

3. ^{1}H and ^{13}C NMR spectra 46

4. Cyclic voltammograms 76

5. Absorption and emission spectra 87

6. Data of quantum chemical calculations 98

6.1 Quantum chemical calculation data of tetrakis(4-(10-methyl-10*H*-phenothiazin-3-yl)phenyl)silane (**5a**) 98
6.1.1 Computed xyz-coordinates of compound **5a** (cam-B3LYP/def2svp SMD CH_2Cl_2 GD3BJ)....98
6.1.2 Computed excitation energies of compound **5a** (cam-B3LYP/def2svp SMD CH_2Cl_2 GD3BJ) 103
6.1.3 Computed emission energies of compound **5a** (cam-B3LYP/def2svp SMD CH_2Cl_2 GD3BJ) 107
6.2 Quantum chemical calculation data of tetrakis(4-(10-isopropyl-10*H*-phenothiazin-3-yl)phenyl)silane (**5b**) 110
6.2.1 Computed xyz-coordinates of compound **5b** (cam-B3LYP/def2svp SMD CH_2Cl_2 GD3BJ)....110
6.2.2 Computed excitation energies of compound **5b** (cam-B3LYP/def2svp SMD CH_2Cl_2 GD3BJ) 116
6.2.3 Computed emission energies of compound **5b** (cam-B3LYP/def2svp SMD CH_2Cl_2 GD3BJ) 120
6.3 Quantum chemical calculation data of tetrakis(4-(10-ethyl-10*H*-phenothiazin-3-yl)phenyl)silane (**5c/5d**) 122

6.3.1 Computed xyz-coordinates of compounds **5c**/**5d** (cam-B3LYP/def2svp SMD CH_2Cl_2 GD3BJ)....122
6.3.2 Computed excitation energies of compounds **5c**/**5d** (cam-B3LYP/def2svp SMD CH_2Cl_2 GD3BJ).... 128
6.3.3 Computed emission energies of compounds **5c**/**5d** (cam-B3LYP/def2svp SMD CH_2Cl_2 GD3BJ).... 132
6.4 Quantum chemical calculation data of tetrakis(4-(10-benzyl-10*H*-phenothiazin-3-yl)phenyl)silane (**5e**).... 135
6.4.1 Computed xyz-coordinates of compound **5e** (cam-B3LYP/def2svp SMD CH_2Cl_2 GD3BJ)....135
6.4.2 Computed excitation energies of compound **5e** (cam-B3LYP/def2svp SMD CH_2Cl_2 GD3BJ).... 142
6.4.3 Computed emission energies of compound **5e** (cam-B3LYP/def2svp SMD CH_2Cl_2 GD3BJ).... 146
6.5 Quantum chemical calculation data of tetrakis(4-(7-(4-fluorophenyl)-10-(4-methoxyphenyl)-10*H*-phenothiazin-3-yl)phenyl)silane (**5f**).... 148
6.5.1 Computed xyz-coordinates of compound **5f** (cam-B3LYP/def2svp SMD CH_2Cl_2 GD3BJ)....148
6.5.2 Computed excitation energies of compound **5f** (cam-B3LYP/def2svp SMD CH_2Cl_2 GD3BJ).... 156
6.5.3 Computed emission energies of compound **5f** (cam-B3LYP/def2svp SMD CH_2Cl_2 GD3BJ).... 160
6.6 Quantum chemical calculation data of tetrakis(4-(10-hexyl-7-(4-(trifluoromethyl)phenyl)-10*H*-phenothiazin-3-yl)phenyl)silane (**5g**).... 163
6.6.1 Computed xyz-coordinates of compound **5g** (cam-B3LYP/def2svp SMD CH_2Cl_2 GD3BJ)....163
6.6.2 Computed excitation energies of compound **5g** (cam-B3LYP/def2svp SMD CH_2Cl_2 GD3BJ).... 169
6.6.3 Computed emission energies of compound **5g** (cam-B3LYP/def2svp SMD CH_2Cl_2 GD3BJ).... 174
6.7 Quantum chemical calculation data of tetrakis(4-(7-(4-chlorophenyl)-10-hexyl-10*H*-phenothiazin-3-yl)phenyl)silane (**5h**).... 177

6.7.1 Computed xyz-coordinates of compound **5h** (cam-B3LYP/def2svp SMD CH_2Cl_2 GD3BJ)........177
6.7.2 Computed excitation energies of compound **5h** (cam-B3LYP/def2svp SMD CH_2Cl_2 GD3BJ)........184
6.7.3 Computed emission energies of compound **5h** (cam-B3LYP/def2svp SMD CH_2Cl_2 GD3BJ)........189
6.8 Quantum chemical calculation data of tetrakis(4-(7-(4-fluorophenyl)-10-hexyl-10*H*-phenothiazin-3-yl)phenyl)silane (**5i**)........191
6.8.1 Computed xyz-coordinates of compound **5i** (cam-B3LYP/def2svp SMD CH_2Cl_2 GD3BJ)........191
6.8.2 Computed excitation energies of compound **5i** (cam-B3LYP/def2svp SMD CH_2Cl_2 GD3BJ)........198
6.8.3 Computed emission energies of compound **5i** (cam-B3LYP/def2svp SMD CH_2Cl_2 GD3BJ)........203
6.9 Quantum chemical calculation data of tetrakis(4-(10-hexyl-7-phenyl-10*H*-phenothiazin-3-yl)phenyl)silane (**5j**)........205
6.9.1 Computed xyz-coordinates of compound **5j** (cam-B3LYP/def2svp SMD CH_2Cl_2 GD3BJ)........205
6.9.2 Computed excitation energies of compound **5j** (cam-B3LYP/def2svp SMD CH_2Cl_2 GD3BJ)........212
6.9.3 Computed emission energies of compound **5j** (cam-B3LYP/def2svp SMD CH_2Cl_2 GD3BJ)........216
6.10 Quantum chemical calculation data of tetrakis(4-(10-hexyl-7-(p-tolyl)-10*H*-phenothiazin-3-yl)phenyl)silane (**5k**)........218
6.10.1 Computed xyz-coordinates of compound **5k** (cam-B3LYP/def2svp SMD CH_2Cl_2 GD3BJ)........218
6.10.2 Computed excitation energies of compound **5k** (cam-B3LYP/def2svp SMD CH_2Cl_2 GD3BJ)........225
6.10.3 Computed emission energies of compound **5k** (cam-B3LYP/def2svp SMD CH_2Cl_2 GD3BJ)........230
6.11 Quantum chemical calculation data of tetrakis(4-(10-hexyl-7-(4-methoxyphenyl)-10*H*-phenothiazin-3-yl)phenyl)silane (**5l**)........233

6.11.1 Computed xyz-coordinates of compound **5l** (cam-B3LYP/def2svp SMD CH_2Cl_2 GD3BJ)....233
6.11.2 Computed excitation energies of compound **5l** (cam-B3LYP/def2svp SMD CH_2Cl_2 GD3BJ).... 241
6.11.3 Computed emission energies of compound **5l** (cam-B3LYP/def2svp SMD CH_2Cl_2 GD3BJ).... 246
6.12 Quantum chemical calculation data of tetrakis(4-(10-hexyl-7-(5-methylthiophen-2-yl)-10*H*-phenothiazin-3-yl)phenyl)silane (**5m**) 249
6.12.1 Computed xyz-coordinates of compound **5m** (cam-B3LYP/def2svp SMD CH_2Cl_2 GD3BJ)....249
6.12.2 Computed excitation energies of compound **5m** (cam-B3LYP/def2svp SMD CH_2Cl_2 GD3BJ).... 256
6.12.3 Computed emission energies of compound **5m** (cam-B3LYP/def2svp SMD CH_2Cl_2 GD3BJ).... 261
6.13 Quantum chemical calculation data of tetrakis(4-(10,10'-dihexyl-10*H*,10'*H*-[3,3'-biphenothiazin]-7-yl)phenyl)silane (**5n**) 263
6.13.1 Computed xyz-coordinates of compound **5n** (cam-B3LYP/def2svp SMD CH_2Cl_2 GD3BJ)....263
6.13.2 Computed excitation energies of compound **5n** (cam-B3LYP/def2svp SMD CH_2Cl_2 GD3BJ).... 272
6.13.3 Computed emission energies of compound **5n** (cam-B3LYP/def2svp SMD CH_2Cl_2 GD3BJ).... 277
6.14 Quantum chemical calculation data of tetrakis(4-(10-hexyl-2-methyl-10*H*-phenothiazin-3-yl)phenyl)silane (**5o**).... 280
6.14.1 Computed xyz-coordinates of compound **5o** (cam-B3LYP/def2svp SMD CH_2Cl_2 GD3BJ)....280
6.14.2 Computed excitation energies of compound **5o** (cam-B3LYP/def2svp SMD CH_2Cl_2 GD3BJ).... 286
6.14.3 Computed emission energies of compound **5o** (cam-B3LYP/def2svp SMD CH_2Cl_2 GD3BJ).... 291
6.15 Quantum chemical calculation data of tetrakis(4-(10-hexyl-2-methoxy-10*H*-phenothiazin-3-yl)phenyl)silane (**5p**).... 293

6.15.1 Computed xyz-coordinates of compound **5p** (cam-B3LYP/def2svp SMD CH_2Cl_2 GD3BJ)....293
6.15.2 Computed excitation energies of compound **5p** (cam-B3LYP/def2svp SMD CH_2Cl_2 GD3BJ)....299
6.15.3 Computed emission energies of compound **5p** (cam-B3LYP/def2svp SMD CH_2Cl_2 GD3BJ)....303
6.16 Tetrakis(4-(10-hexyl-2-(methylthio)-10*H*-phenothiazin-3-yl)phenyl)silane (**5q**)....306
6.16.1 Computed xyz-coordinates of compound **5q** (cam-B3LYP/def2svp SMD CH_2Cl_2 GD3BJ)....306
6.16.2 Computed excitation energies of compound **5q** (cam-B3LYP/def2svp SMD CH_2Cl_2 GD3BJ)....312
6.16.3 Computed emission energies of compound **5q** (cam-B3LYP/def2svp SMD CH_2Cl_2 GD3BJ)....317
6.17 Quantum chemical calculation data of tetrakis(4-(10-hexyl-10*H*-phenothiazin-1-yl)phenyl)silane (**5r**)....320
6.17.1 Computed xyz-coordinates of compound **5r** (cam-B3LYP/def2svp SMD CH_2Cl_2 GD3BJ)....320
6.17.2 Computed excitation energies of compound **5r** (cam-B3LYP/def2svp SMD CH_2Cl_2 GD3BJ)....326
6.17.3 Computed emission energies of compound **5r** (cam-B3LYP/def2svp SMD CH_2Cl_2 GD3BJ)....330
6.18 Quantum chemical calculation data of tetrakis(10-hexyl-10*H*-phenothiazin-3-yl)silane (**6**)....332
6.18.1 Computed xyz-coordinates of compound **6** (cam-B3LYP/def2svp SMD CH_2Cl_2 GD3BJ)....332
6.18.2 Computed excitation energies of compound **6** (cam-B3LYP/def2svp SMD CH_2Cl_2 GD3BJ)....337
6.18.3 Computed emission energies of compound **6** (cam-B3LYP/def2svp SMD CH_2Cl_2 GD3BJ)....342
6.19 Quantum chemical calculation data of tetrakis(5-(10-hexyl-10*H*-phenothiazin-3-yl)thiophen-2-yl)silane (**7**)....344

6.19.1 Computed xyz-coordinates of compound **7** (cam-B3LYP/def2svp SMD CH_2Cl_2 GD3BJ)....344

6.19.2 Computed excitation energies of compound **7** (cam-B3LYP/def2svp SMD CH_2Cl_2 GD3BJ).... 349

6.19.3 Computed emission energies of compound **7** (cam-B3LYP/def2svp SMD CH_2Cl_2 GD3BJ).... 353

6.20 .... Quantum chemical calculation data of tetrakis(4-(5-(10-hexyl-10*H*-phenothiazin-3-yl)thiophen-2-yl)phenyl)silane (**8**).... 355

6.20.1 Computed xyz-coordinates of compound **8** (cam-B3LYP/def2svp SMD CH_2Cl_2 GD3BJ)....355

6.20.2 Computed excitation energies of compound **8** (cam-B3LYP/def2svp SMD CH_2Cl_2 GD3BJ).... 362

6.20.3 Computed emission energies of compound **8** (cam-B3LYP/def2svp SMD CH_2Cl_2 GD3BJ).... 367

7. Crystallographic data....369

7.1 X-ray structure determinations.... 369

7.2 Hirshfeld analysis.... 371

8. Literature....375

1. General considerations – overview of experimental procedures

Unless otherwise stated, reactions were carried out in oven-dried Schlenk tubes or multi-neck flasks under an atmosphere of dry nitrogen or argon using standard septum/syringe techniques. Anhydrous solvents were obtained from an MB-SPS 800 solvent purification system (M. Braun) and used as received. Commercial reagents were purchased from ABCR, Acros, Alfa Aesar, Fluorochem, Macherey-Nagel, Merck, Roth, Sigma-Aldrich, and VWR and used without further purification.

For non-ambient temperatures, cooling baths (ice/water, 0 °C; salt/ice, −10 °C; acetone/dry ice, −78 °C) or silicone-oil baths were used. Thin-layer chromatography (TLC) was performed on aluminum sheets pre-coated with silica gel 60 F254 (Merck). Visualization was carried out under UV light at 254 and 365 nm. Flash column chromatography was conducted using a Büchi Pure C-810 FlashPrep system equipped with Büchi FlashPure EcoFlex silica cartridges (50 µm irregular), employing cartridge sizes between 25 g and 120 g depending on the scale. Solvent removal under reduced pressure was performed using a Heidolph Laborota 4000 rotary evaporator.

Melting points were determined using a Büchi B-545 melting point apparatus and are reported uncorrected.

All NMR spectra (^{1}H, ^{13}C, ^{29}Si, DEPT-135) were recorded on Bruker Avance III 300 (300 MHz), Bruker Avance III 600 (600 MHz) and Bruker Avance NEO Evo (600 MHz) spectrometers at 298 K unless otherwise noted. Deuterated solvents used included acetone-d_6, $CDCl_3$, CD_2Cl_2, DMSO-d_6, and THF-d_8. Chemical shifts are referenced to the residual solvent signals (acetone-d_6: δ_H 2.05, δ_C 29.84, 206.26; $CDCl_3$: δ_H 7.26, δ_C 77.16; CD_2Cl_2: δ_H 5.32, δ_C 54.00; DMSO-d_6: δ_H 2.50, δ_C 39.52; THF-d_8: δ_H 1.73, 3.58, δ_C 25.37, 67.57). Multiplicities are abbreviated as follows: s = singlet, d = doublet, dd = doublet of doublets, ddd = doublet of doublets of doublets, dt = doublet of triplets, t = triplet, td = triplet of doublets, quint = quintet, sept = septet, m = multiplet. Carbon multiplicities (CH, CH_2, CH_3, and quaternary carbons) were assigned based on DEPT-135 experiments where applicable.

Electron ionization (EI) mass spectra were recorded on a TSQ 7000 triple quadrupole mass spectrometer (Finnigan MAT). All signals above 10% of the base peak and the molecular ion peak are reported. Electrospray ionization (ESI) mass spectra were acquired using a Finnigan LCQ Deca ion trap instrument (Thermo Quest). High-resolution ESI (HR-ESI) and MALDI-TOF spectra were recorded on a UHR-QTOF maXis 4G and an UltrafleXtreme spectrometer (Bruker Daltonics), respectively.

Infrared (IR) spectra were recorded using a Shimadzu IRAffinity-1 instrument equipped with a single-reflection ATR unit (diamond ATR crystal, range: 4000–600 cm^{-1}). Absorption band intensities are denoted as s = strong, m = medium, and w = weak.

Elemental analyses were performed at the Institute of Pharmaceutical Chemistry, Heinrich Heine University Düsseldorf, using a PerkinElmer 2400 Series II CHNS/O Analyzer.

Crystals suitable for X-ray analysis were selected using a polarization microscope and fixed with NVH oil on a loop glass fiber. Crystal structures were determined using a Rigaku XtaLAB-Synergy S diffractometer equipped with a HyPix 6000 detector and a Photon Jet X-ray source with monochromated Cu-K_α radiation (λ = 1.54184 Å) with a generator setting of 50 kV and 1 mA. Data acquisition was performed, and the raw data were processed using CrysAlisPro v43.98a (Rigaku Corporation). Crystal structures were solved by direct methods and subsequent ΔF syntheses using SHELXT[54] and OLEX2[55]. Approximate positions of the H atoms were found in different stages of converging refinements by full-matrix least-squares calculations on F^2 using SHELXL[56] and OLEX2[55]. Anisotropic displacement parameters were refined for all atoms heavier than hydrogens. The riding model was applied to the corresponding H atoms, assuming idealized bond lengths and angles for all the CH, CH_2 and CH_3 groups. The isotropic displacement parameters of these H atoms were constrained to 120, 120 and 150% of the equivalent isotropic displacement parameters of the parent carbon atoms and H atoms of CH_3 groups were allowed to rotate around the adjacent C–C bonds. All graphics were produced using Mercury v.2021.2.0 (CCDC).[38]

Cyclic voltammetry was performed at 293 K using a Princeton Applied Research 263A potentiostat. Measurements were conducted in a 3.0 mL single-compartment cell using a three-electrode configuration: platinum disk (2 mm) as the working electrode, Ag/AgCl (3.0 M NaCl, aqueous) as the reference electrode, and a platinum wire as the counter electrode. The electrolyte was a degassed 0.1 M solution of tetrabutylammonium hexafluorophosphate in anhydrous dichloromethane. Analyte concentrations were typically ~1 mM. Decamethylferrocene (Fc*) was used as an internal standard.[57] Potentials are reported vs. Fc/Fc^+ and were internally calibrated using Fc^*/Fc^{*+} ($E_{1/2}$ = –0.54 V vs. Fc/Fc^+).[58-60] For reversible waves, $E_{1/2}$ was taken as $\frac{E_{pa} + E_{pc}}{2}$. Scan rates of 100, 250, 500, and 1000 mV/s were applied. Data were evaluated using VersaStudio software; if peak assignment was ambiguous, additional convolution–deconvolution analysis was performed using Condecon.

UV/Vis spectra were recorded at 293 K using a PerkinElmer Lambda 19 UV/Vis/NIR spectrometer. Unless otherwise stated, spectra were measured in HPLC-grade dichloromethane. Molar extinction coefficients were obtained from Beer–Lambert plots using at least five concentrations in the range of $1–10 \cdot 10^{-5}$ M. Fluorescence spectra were recorded

at 293 K using an FS5 spectrofluorometer (Edinburgh Instruments). For measurements in solution, the SC-05 module was used at a concentration of 10^{-7} M. For solid-state measurements, the SC-10 module was employed. Absolute fluorescence quantum yields were determined using an integrating sphere (Edinburgh Instruments, SC-30). Solution measurements were performed at an absorbance of ~0.10 at the excitation wavelength; solid-state values were obtained in the integrating sphere in the SC-10 module with appropriate blank correction.

Time-resolved fluorescence measurements were performed on an FLS 920 fluorometer (Edinburgh Instruments) equipped with a pulsed EPLED light source (λ_{ex} = 375 ± 10 nm) and a multichannel plate photomultiplier (MCP-PMT). Fluorescence decays were evaluated by reconvolution fitting (FAST, Edinburgh Instruments) using a monoexponential model. Reduced χ^2 values were typically 1.0–1.3. Instrument response functions were recorded using a non-emissive LUDOX solution (silica particle dispersion) and included in the analysis to account for temporal broadening.

All quantum-chemical calculations were carried out on the ZIM high-performance computing cluster Ivybridge at Heinrich Heine University Düsseldorf using Gaussian 09[61] and Gaussian 16[51]. Density-functional theory methods were employed. Frequency analyses of the optimized geometries were used to identify the nature of the stationary points. Minima exhibited no imaginary vibrational frequencies, transition states exactly one. The methods, functionals, and basis sets employed are reported together with the corresponding results.

2. Syntheses

2.1 Synthesis of starting materials according to literature

The following compounds were obtained through synthesis protocols adapted from established literature methods: tetrakis(4-bromophenyl)silane (**1**),[14] 3-bromo-10-hexyl-7-iodo-10*H*-phenothiazine (**3**),[29] 3-bromo-10-methyl-10*H*-phenothiazine (**4a**),[35] 3-bromo-10-isopropyl-10*H*-phenothiazine (**4b**),[62] 3-bromo-10-hexyl-10*H*-phenothiazine (**4c**),[30] 3-bromo-10-(2-decyltetradecyl)-10*H*-phenothiazine (**4d**),[37] 10-benzyl-3-bromo-10*H*-phenothiazine (**4e**),[36] 3-bromo-10-hexyl-2-methyl-10*H*-phenothiazine (**4o**),[32] 3-bromo-10-hexyl-2-methoxy-10*H*-phenothiazine (**4p**),[32] 3-bromo-10-hexyl-2-(methylthio)-10*H*-phenothiazine (**4q**),[32] 1-bromo-10-hexyl-10*H*-phenothiazine (**4r**),[33] 10-hexyl-3-(thiophen-2-yl)-10*H*-phenothiazine (**4s**),[34] 10-hexyl-3-phenyl-10*H*-phenothiazine (**9**).[63]

2.2 Synthesis of non-commercial and unpublished starting materials

2.2.1 General procedure (GPI) of the BLEBS sequence for the synthesis of (hetero)arylated phenothiazine bromides 4g-n

In a dry Schlenk tube under nitrogen, the aryl bromide (1.00 equiv.) was dissolved in anhydrous THF (6.00 mL/mmol) and cooled to −78 °C. *n*-Butyllithium (1.10 equiv., 1.6 M in *n*-hexane) was added dropwise. After 30 min of stirring at –78 °C, trimethyl borate (1.30 equiv.) was added. The cooling bath was then removed, and the reaction mixture was stirred at 20 °C for 1 h. Subsequently, 3-bromo-10-hexyl-7-iodo-10*H*-phenothiazine (**3**) (1.20 equiv.), tetrakis(triphenylphosphane)palladium(0) (5 mol%), sodium hydroxide (1.30 equiv.), and distilled water (2.00 mL/mmol) were added. The mixture was then refluxed at 67 °C for 18 h. After completion of the reaction, the organic phase was separated, and the aqueous phase was extracted three times with dichloromethane. The combined organic phases were concentrated. The crude product was adsorbed onto Celite® and purified by flash column chromatography on silica gel.

Table S1. Experimental details for the synthesis of (hetero)arylated phenothiazine bromides **4g-n**.

entry	aryl bromide **2** [g] (mmol)	phenothiazine **3** [g] (mmol)	yield of product **4** [g] (%)
1	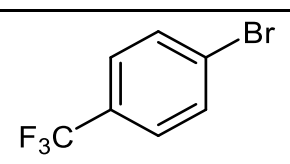 2.25 (10.0) of **2a**	5.86 (12.0)	3.76 (74) of **4g**
2	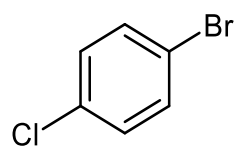 1.91 (10.0) of **2b**	5.86 (12.0)	3.59 (76) of **4h**

3	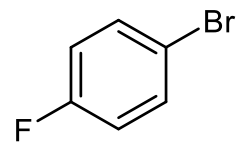 1.75 (10.0) of **2c**	5.86 (12.0)	3.86 (85) of **4i**
4	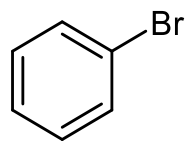 1.57 (10.0) of **2d**	5.86 (12.0)	3.62 (83) of **4j**
5	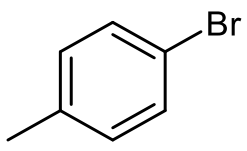 0.342 (2.00) of **2e**	1.17 (2.40)	0.719 (80) of **4k**
6	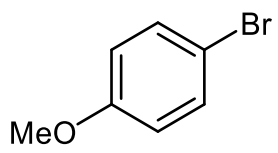 1.87 (10.0) of **2f**	5.86 (12.0)	4.11 (88) of **4l**
7	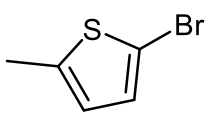 1.24 (7.00) of **2g**	4.10 (8.40)	2.42 (75) of **4m**
8	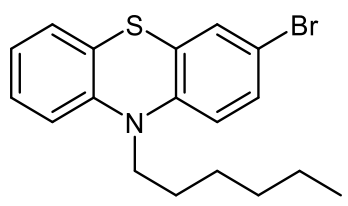 1.88 (5.20) of **2h**	3.05 (6.24)	2.74 (82) of **4n**

2.2.2 3-Bromo-10-hexyl-7-(4-(trifluoromethyl)phenyl)-10*H*-phenothiazine (4g)

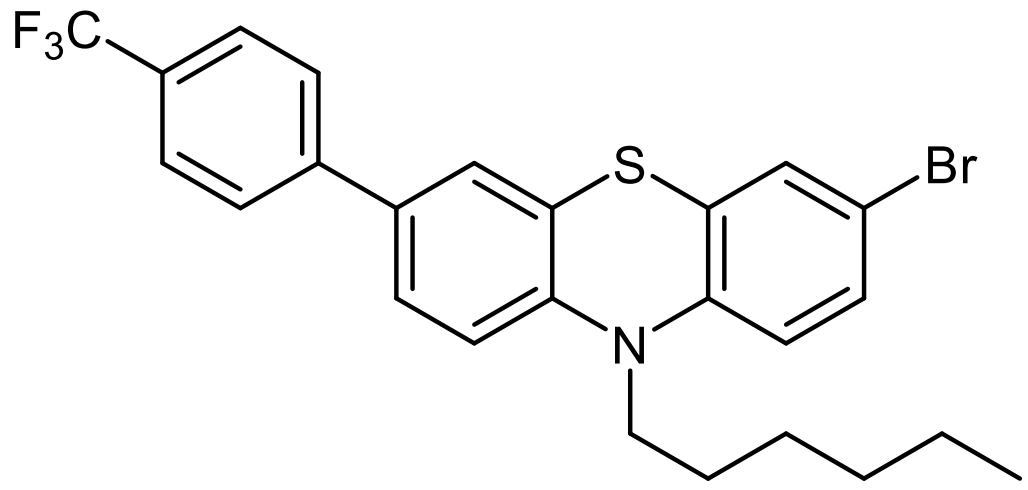

$C_{25}H_{23}BrF_3NS$

[506.43]

The crude product was synthesized according to **GPI** and purified by column chromatography on silica gel using gradient elution (petroleum ether → petroleum ether/acetone 100:1). Compound **4g** was obtained as a pale-yellow oil in a yield of 3.76 g (7.42 mmol, 74%), R_f (petroleum ether) = 0.22.

^{1}H NMR (600 MHz, acetone-d$_6$): δ 7.82 – 7.87 (m, 2H), 7.73 – 7.78 (m, 2H), 7.58 (dd, $^3J_{HH}$ = 8.5 Hz, $^4J_{HH}$ = 2.2 Hz, 1H), 7.50 (d, $^4J_{HH}$ = 2.2 Hz, 1H), 7.34 (dd, $^3J_{HH}$ = 8.7 Hz, $^4J_{HH}$ = 2.3 Hz, 1H), 7.29 (d, $^4J_{HH}$ = 2.3 Hz, 1H), 7.13 (d, $^3J_{HH}$ = 8.5 Hz, 1H), 6.98 (d, $^3J_{HH}$ = 8.7 Hz, 1H), 3.96 (t, $^3J_{HH}$ = 7.1 Hz, 2H), 1.76 – 1.84 (m, 2H), 1.42 – 1.50 (m, 2H), 1.24 – 1.34 (m, 4H), 0.81 – 0.88 (m, 3H). ^{13}C NMR (150 MHz, acetone-d$_6$): δ 146.1 (C_{quat}), 145.3 (C_{quat}), 144.4 (C_{quat}), 134.7 (C_{quat}), 131.2 (CH), 130.2 (CH), 129.3 (q, $^2J_{CF}$ = 32.2 Hz, C_{quat}), 127.8 (CH), 127.5 (C_{quat}), 127.5 (CH), 126.6 (q, $^3J_{CF}$ = 3.8 Hz, CH), 126.5 (CH), 125.5 (q, $^1J_{CF}$ = 270.9 Hz, C_{quat}), 124.7 (C_{quat}), 118.4 (CH), 117.3 (CH), 115.2 (C_{quat}), 48.1 (CH_2), 32.3 (CH_2), 27.5 (CH_2), 27.2 (CH_2), 23.4 (CH_2), 14.4 (CH_3). MS (EI) *m/z* (%): 508 ([$M^{13}C^{81}Br]^+$, 20), 507 ([$M^{12}C^{81}Br]^+$, 81), 506 ([$M^{13}C^{79}Br]^+$, 13), 505 ([$M^{12}C^{79}Br]^+$, 77), 436 ([$C_{20}H_{12}{}^{81}BrF_3NS]^+$, 43), 434 ([$C_{20}H_{12}{}^{79}BrF_3NS]^+$, 39), 423 ([$^{13}C_{19}H_{10}{}^{81}BrF_3NS]^+$, 20), 422 ([$^{12}C_{19}H_{10}{}^{81}BrF_3NS]^+$, 100), 421 ([$^{13}C_{19}H_{10}{}^{79}BrF_3NS]^+$, 17), 420 ([$^{12}C_{19}H_{10}{}^{79}BrF_3NS]^+$, 97), 404 ([$C_{20}H_{12}{}^{81}BrF_3N]^+$, 14), 402 ([$C_{20}H_{12}{}^{79}BrF_3N]^+$, 17), 355 (26), 342 (11), 341 (29), 340 (13), 323 ([$C_{20}H_{12}F_3N]^+$, 10), 272 (13). IR: $\tilde{\nu}$ [cm^{-1}] = 2955 (w), 2928 (w), 2857 (w), 1616 (w), 1599 (w), 1491 (w), 1456 (s), 1420 (w), 1391 (w), 1321 (s), 1263 (m), 1252 (m), 1190 (w), 1161 (m), 1109 (s), 1070 (s), 1015 (m), 868 (w), 843 (m), 808 (m), 775 (w), 756 (w), 692 (w), 606 (w). Anal. calcd. for $C_{25}H_{23}BrF_3NS$ [506.4]: C 59.29, H 4.58, N 2.77, S 6.33; Found: C 59.32, H 4.58, N 2.88, S 6.65.

2.2.3 3-Bromo-7-(4-chlorophenyl)-10-hexyl-10*H*-phenothiazine (4h)

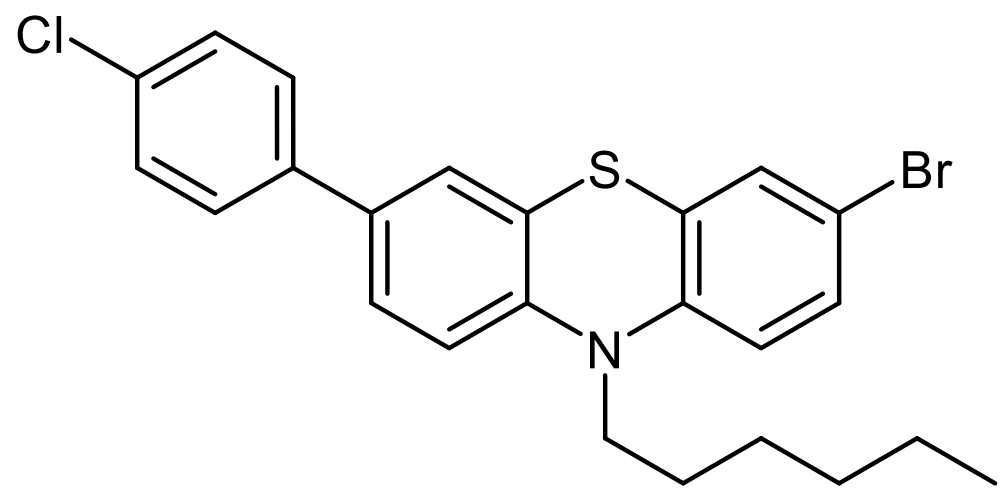

$C_{24}H_{23}BrClNS$

[472.87]

The crude product was synthesized according to **GPI** and purified by column chromatography on silica gel (petroleum ether). Compound **4h** was obtained as a pale yellow oil in a yield of 3.59 g (7.59 mmol, 76%), R_f (petroleum ether) = 0.17.

^{1}H NMR (600 MHz, acetone-d$_6$): δ 7.61 – 7.66 (m, 2H), 7.50 (dd, $^3J_{HH}$ = 8.5 Hz, $^4J_{HH}$ = 2.2 Hz, 1H), 7.40 – 7.47 (m, 3H), 7.33 (dd, $^3J_{HH}$ = 8.7 Hz, $^4J_{HH}$ = 2.3 Hz, 1H), 7.29 (d, $^4J_{HH}$ = 2.3 Hz, 1H), 7.10 (d, $^3J_{HH}$ = 8.5 Hz, 1H), 6.97 (d, $^3J_{HH}$ = 8.7 Hz, 1H), 3.95 (t, $^3J_{HH}$ = 7.0 Hz, 2H), 1.75 – 1.83 (m, 2H), 1.42 – 1.50 (m, 2H), 1.24 – 1.34 (m, 4H), 0.82 – 0.89 (m, 3H). ^{13}C NMR (150 MHz, acetone-d$_6$): δ 145.6 (C_{quat}), 145.5 (C_{quat}), 139.4 (C_{quat}), 135.2 (C_{quat}), 133.6 (C_{quat}), 131.2 (CH), 130.2 (CH), 129.9 (CH), 128.9 (CH), 127.7 (C_{quat}), 127.1 (CH), 126.3 (CH), 125.4 (C_{quat}), 118.3 (CH), 117.3 (CH), 115.0 (C_{quat}), 48.1 (CH_2), 32.3 (CH_2), 27.5 (CH_2), 27.2 (CH_2), 23.4 (CH_2), 14.4 (CH_3). MS (EI) *m/z* (%): 475 (28), 474 ($[M^{13}C^{81}Br]^+$, 29), 473 ($[M^{12}C^{81}Br]^+$, 100), 472 ($[M^{13}C^{79}Br]^+$, 31), 471 ($[M^{12}C^{79}Br]^+$, 100), 402 ($[C_{19}H_{12}{}^{81}BrClNS]^+$, 32), 400 ($[C_{19}H_{12}{}^{79}BrClNS]^+$, 24), 390 (28), 389 ($[^{13}C_{18}H_{10}{}^{81}BrClNS]^+$, 23), 388 ($[^{12}C_{18}H_{10}{}^{81}BrClNS]^+$, 97), 387 ($[^{13}C_{18}H_{10}{}^{79}BrClNS]^+$, 20), 386 ($[^{12}C_{18}H_{10}{}^{79}BrClNS]^+$, 75), 321 (10), 307 (11). IR: $\tilde{\nu}$ [cm^{-1}] = 2953 (w), 2928 (w), 2868 (w), 2851 (w), 1908 (w), 1840 (w), 1601 (w), 1460 (s), 1416 (w), 1389 (m), 1350 (m), 1269 (m), 1242 (m), 1144 (w), 1109 (m), 1094 (m), 1011 (w), 887 (w), 870 (w), 835 (m), 795 (s), 762 (m), 727 (w), 652 (w). Anal. calcd. for $C_{24}H_{23}BrClNS$ [472.9]: C 60.96, H 4.90, N 2.96, S 6.78; Found: C 61.16, H 4.71, N 2.95, S 7.14.

2.2.4 3-Bromo-7-(4-fluorophenyl)-10-hexyl-10*H*-phenothiazine (4i)

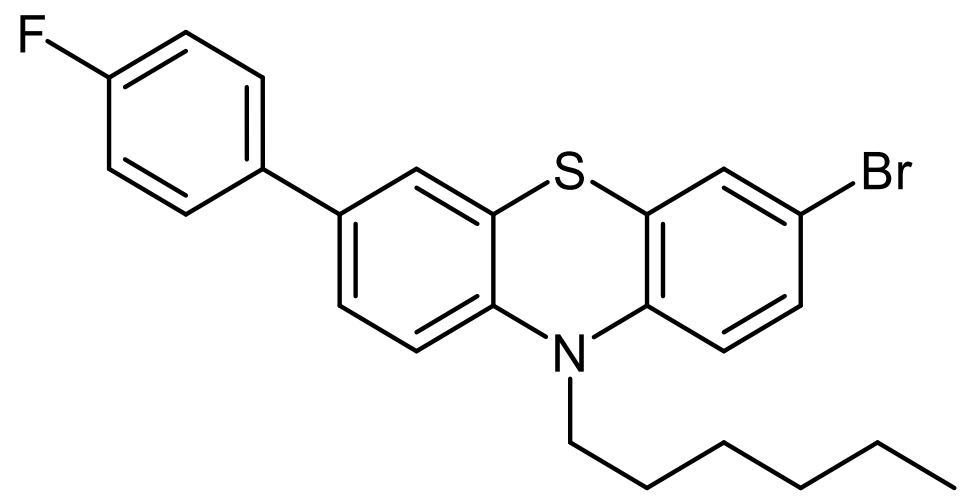

$C_{24}H_{23}BrFNS$

[456.42]

The crude product was synthesized according to **GPI** and purified by column chromatography on silica gel (petroleum ether). Compound **4i** was obtained as a yellow oil in a yield of 3.86 g (8.46 mmol, 85%), R_f (petroleum ether) = 0.23.

^{1}H NMR (600 MHz, acetone-d$_6$): δ 7.61 – 7.68 (m, 2H), 7.47 (dd, $^3J_{HH}$ = 8.4 Hz, $^4J_{HH}$ = 2.2 Hz, 1H), 7.40 (d, $^4J_{HH}$ = 2.2 Hz, 1H), 7.33 (dd, $^3J_{HH}$ = 8.7 Hz, $^4J_{HH}$ = 2.3 Hz, 1H), 7.29 (d, $^4J_{HH}$ = 2.3 Hz, 1H), 7.15 – 7.22 (m, 2H), 7.10 (d, $^3J_{HH}$ = 8.5 Hz, 1H), 6.97 (d, $^3J_{HH}$ = 8.7 Hz, 1H), 3.96 (t, $^3J_{HH}$ = 7.0 Hz, 2H), 1.76 – 1.84 (m, 2H), 1.43 – 1.51 (m, 2H), 1.24 – 1.34 (m, 4H), 0.81 – 0.90 (m, 3H). ^{13}C NMR (150 MHz, acetone-d$_6$): δ 163.3 (d, $^1J_{CF}$ = 244.4 Hz, C_{quat}), 145.6 (C_{quat}), 145.2 (C_{quat}), 137.0 (d, $^4J_{CF}$ = 3.3 Hz, C_{quat}), 135.6 (C_{quat}), 131.2 (CH), 130.2 (CH), 129.2 (d, $^3J_{CF}$ = 7.8 Hz, CH), 127.7 (C_{quat}), 127.1 (CH), 126.3 (CH), 125.4 (C_{quat}), 118.3 (CH), 117.2 (CH), 116.5 (d, $^2J_{CF}$ = 21.6 Hz, CH), 115.0 (C_{quat}), 48.1 (CH_2), 32.3 (CH_2), 27.5 (CH_2), 27.2 (CH_2), 23.4 (CH_2), 14.4 (CH_3). MS (EI) *m/z* (%): 458 ([$M^{13}C^{81}Br$]$^+$, 24), 457 ([$M^{12}C^{81}Br$]$^+$, 79), 456 ([$M^{13}C^{79}Br$]$^+$, 32), 455 ([$M^{12}C^{79}Br$]$^+$, 100), 386 ([$C_{19}H_{12}{}^{81}BrFNS$]$^+$, 26), 384 ([$C_{19}H_{12}{}^{79}BrFNS$]$^+$, 25), 373 ([$^{13}C_{18}H_{10}{}^{81}BrFNS$]$^+$, 18), 372 ([$^{12}C_{18}H_{10}{}^{81}BrFNS$]$^+$, 75), 371 ([$^{13}C_{18}H_{10}{}^{79}BrFNS$]$^+$, 20), 370 ([$^{12}C_{18}H_{10}{}^{79}BrFNS$]$^+$, 74), 305 (11), 291 (15), 290 (11). IR: $\tilde{\nu}$ [cm^{-1}] = 2953 (w), 2926 (w), 2853 (w), 1599 (w), 1516 (w), 1489 (m), 1456 (s), 1420 (w), 1393 (m), 1331 (m), 1300 (w), 1250 (m), 1223 (s), 1194 (m), 1157 (m), 1107 (m), 1013 (w), 868 (w), 837 (m), 806 (s), 758 (m), 727 (w), 685 (w), 667 (w). Anal. calcd. for $C_{24}H_{23}BrFNS$ [456.4]: C 63.16, H 5.08, N 3.07, S 7.02; Found: C 63.34, H 5.08, N 3.06, S 7.23.

2.2.5 3-Bromo-10-hexyl-7-phenyl-10*H*-phenothiazine (4j)

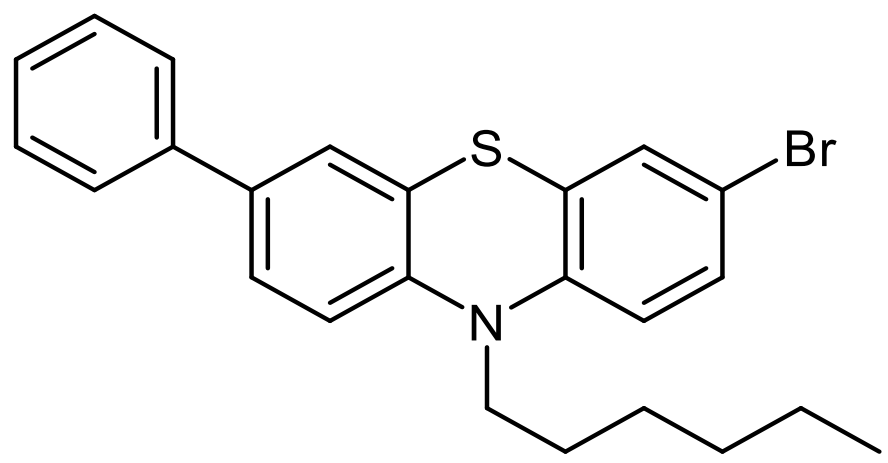

$C_{24}H_{24}BrNS$

[438.43]

The crude product was synthesized according to **GPI** and purified by column chromatography on silica gel (petroleum ether). Compound **4j** was obtained as a yellow oil in a yield of 3.62 g (8.26 mmol, 83%), R_f (petroleum ether) = 0.20.

^{1}H NMR (600 MHz, acetone-d$_6$): δ 7.59 – 7.64 (m, 2H), 7.50 (dd, $^3J_{HH}$ = 8.4 Hz, $^4J_{HH}$ = 2.2 Hz, 1H), 7.39 – 7.46 (m, 3H), 7.28 – 7.35 (m, 3H), 7.11 (d, $^3J_{HH}$ = 8.5 Hz, 1H), 6.97 (d, $^3J_{HH}$ = 8.7 Hz, 1H), 3.96 (t, $^3J_{HH}$ = 7.0 Hz, 2H), 1.76 – 1.84 (m, 2H), 1.43 – 1.51 (m, 2H), 1.25 – 1.35 (m, 4H), 0.81 – 0.90 (m, 3H). ^{13}C NMR (150 MHz, acetone-d$_6$): δ 145.6 (C_{quat}), 145.2 (C_{quat}), 140.6 (C_{quat}), 136.6 (C_{quat}), 131.1 (CH), 130.1 (CH), 129.8 (CH), 128.1 (CH), 127.7 (C_{quat}), 127.2 (CH), 127.1 (CH), 126.3 (CH), 125.3 (C_{quat}), 118.2 (CH), 117.2 (CH), 114.9 (C_{quat}), 48.1 (CH_2), 32.2 (CH_2), 27.5 (CH_2), 27.2 (CH_2), 23.4 (CH_2), 14.3 (CH_3). MS (EI) *m*/*z* (%): 440 ([M^{13}C^{81}Br]$^+$, 25), 439 ([M^{12}C^{81}Br]$^+$, 75), 438 ([M^{13}C^{79}Br]$^+$, 38), 437 ([M^{12}C^{79}Br]$^+$, 100), 368 ([$C_{19}H_{13}{}^{81}BrNS$]$^+$, 28), 366 ([$C_{19}H_{13}{}^{79}BrNS$]$^+$, 27), 355 ([$^{13}C_{18}H_{11}{}^{81}BrNS$]$^+$, 22), 354 ([$^{12}C_{18}H_{11}{}^{81}BrNS$]$^+$, 90), 353 ([$^{13}C_{18}H_{11}{}^{79}BrNS$]$^+$, 25), 352 ([$^{12}C_{18}H_{11}{}^{79}BrNS$]$^+$, 89), 334 ([$C_{19}H_{13}BrN$]$^+$, 10), 287 (11), 273 (18), 272 (16). IR: $\tilde{\nu}$ [cm^{-1}] = 3057 (w), 2953 (w), 2926 (w), 2853 (w), 1601 (w), 1456 (s), 1391 (w), 1329 (w), 1294 (w), 1261 (m), 1248 (m), 1192 (w), 1146 (w), 1107 (w), 1080 (w), 1051 (w), 868 (w), 802 (m), 758 (s), 733 (w), 696 (m). Anal. calcd. for $C_{24}H_{24}BrNS$ [438.4]: C 65.75, H 5.52, N 3.19, S 7.31; Found: C 65.88, H 5.50, N 3.07, S 7.37.

2.2.6 3-Bromo-10-hexyl-7-(*p*-tolyl)-10*H*-phenothiazine (4k)

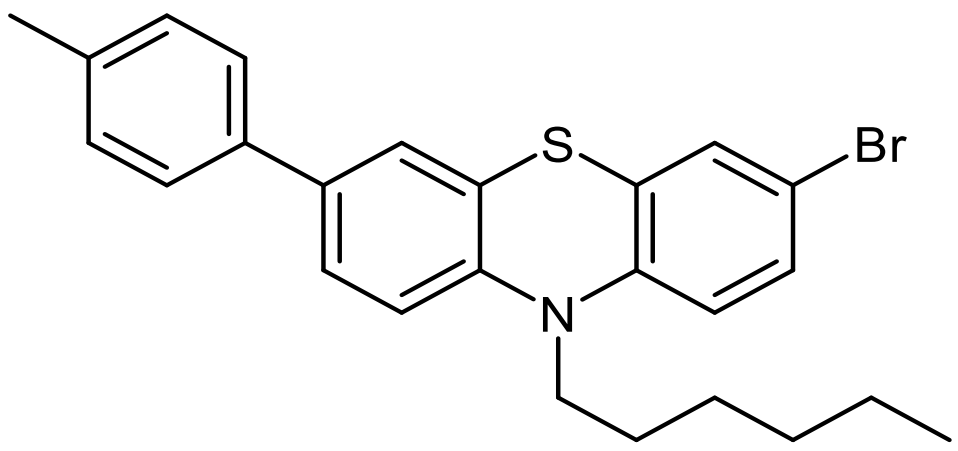

$C_{25}H_{26}BrNS$

[452.45]

The crude product was synthesized according to **GPI** and purified by column chromatography on silica gel (petroleum ether). Compound **4k** was obtained as a pale yellow oil in a yield of 0.719 g (1.59 mmol, 80%), R_f (petroleum ether) = 0.24.

^{1}H NMR (600 MHz, acetone-d$_6$): δ 7.45 – 7.53 (m, 3H), 7.40 (d, $^{4}J_{HH}$ = 2.0 Hz, 1H), 7.33 (dd, $^{3}J_{HH}$ = 8.6 Hz, $^{4}J_{HH}$ = 2.1 Hz, 1H), 7.29 (s, 1H), 7.24 (d, $^{3}J_{HH}$ = 7.7 Hz, 2H), 7.09 (dd, $^{3}J_{HH}$ = 8.4 Hz, $^{4}J_{HH}$ = 1.8 Hz, 1H), 6.97 (dd, $^{3}J_{HH}$ = 8.7 Hz, $^{4}J_{HH}$ = 1.8 Hz, 1H), 3.95 (t, $^{3}J_{HH}$ = 7.1 Hz, 2H), 2.34 (s, 3H), 1.80 (pent, $^{3}J_{HH}$ = 7.3 Hz, 2H), 1.43 – 1.51 (m, 2H), 1.26 – 1.35 (m, 4H), 0.82 – 0.88 (m, 3H). ^{13}C NMR (150 MHz, acetone-d$_6$): δ 145.7 (C_{quat}), 145.0 (C_{quat}), 137.7 (C_{quat}), 136.6 (C_{quat}), 131.1 (CH), 130.5 (CH), 130.1 (CH), 127.8 (C_{quat}), 127.1 (CH), 126.9 (CH), 126.1 (CH), 125.2 (C_{quat}), 118.2 (CH), 117.2 (CH), 114.9 (C_{quat}), 48.1 (CH_2), 32.3 (CH_2), 27.5 (CH_2), 27.2 (CH_2), 23.4 (CH_2), 21.2 (CH_3), 14.4 (CH_3); one C_{quat} is not visible, likely due to signal overlap caused by the high number of signals. MS (EI) *m*/*z* (%): 454 ([$M^{13}C^{81}Br]^+$, 95), 453 ([$M^{12}C^{81}Br]^+$, 80), 452 ([$M^{13}C^{79}Br]^+$, 100), 451 ([$M^{12}C^{79}Br]^+$, 58), 382 ([$C_{20}H_{15}{}^{81}BrNS]^+$, 13), 380 ([$C_{20}H_{15}{}^{79}BrNS]^+$, 12), 369 ([$^{13}C_{19}H_{13}{}^{81}BrNS]^+$, 12), 368 ([$^{12}C_{19}H_{13}{}^{81}BrNS]^+$, 37), 367 ([$^{13}C_{19}H_{13}{}^{79}BrNS]^+$, 14), 366 ([$^{12}C_{19}H_{13}{}^{79}BrNS]^+$, 34). IR: $\tilde{\nu}$ [cm^{-1}] = 3051 (w), 3022 (w), 2953 (w), 2922 (w), 2855 (w), 1601 (w), 1489 (m), 1456 (s), 1416 (w), 1391 (m), 1377 (w), 1331 (m), 1294 (w), 1250 (m), 1192 (w), 1159 (w), 1146 (w), 1107 (m), 1082 (w), 1038 (w), 1016 (w), 881 (w), 866 (w), 802 (s), 754 (m), 725 (w), 684 (w). Anal. calcd. for $C_{25}H_{26}BrNS$ [452.5]: C 66.37, H 5.79, N 3.10, S 7.09; Found: C 66.62, H 5.77, N 2.88, S 6.92.

2.2.7 3-Bromo-10-hexyl-7-(4-methoxyphenyl)-10*H*-phenothiazine (4l)

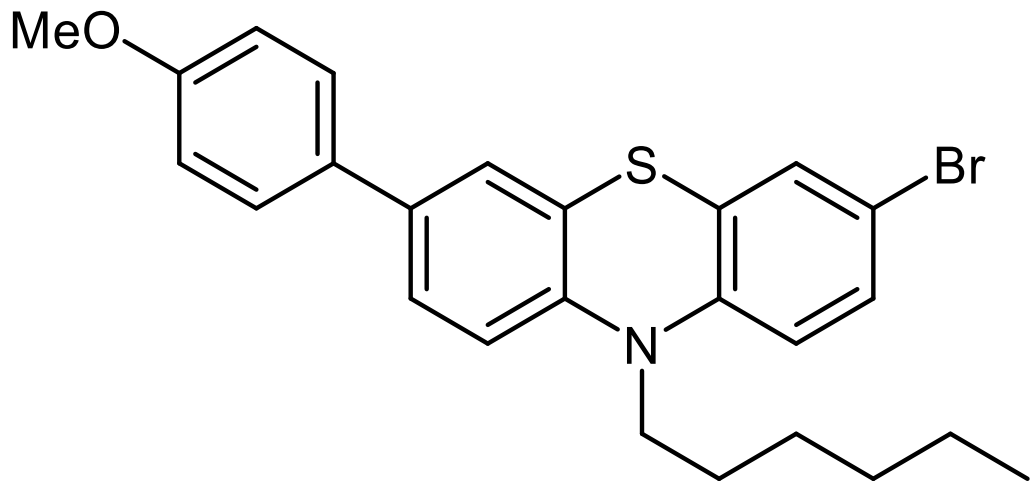

$C_{25}H_{26}BrNOS$

[468.45]

The crude product was synthesized according to **GPI** and purified by column chromatography on silica gel (petroleum ether/acetone 25:1). Compound **4l** was obtained as a pale yellow oil in a yield of 4.11 g (8.77 mmol, 88%), R_f (petroleum ether) = 0.13.

^{1}H NMR (300 MHz, DMSO-d$_6$): δ 7.50 – 7.59 (m, 2H), 7.44 (dd, $^3J_{HH}$ = 8.5 Hz, $^4J_{HH}$ = 2.2 Hz, 1H), 7.29 – 7.40 (m, 3H), 6.89 – 7.08 (m, 4H), 3.84 (t, $^3J_{HH}$ = 6.9 Hz, 2H), 3.77 (s, 3H), 1.66 (pent, $^3J_{HH}$ = 6.9 Hz, 2H), 1.30 – 1.44 (m, 2H), 1.23 (m, 4H), 0.76 – 0.87 (m, 3H). ^{13}C NMR (75 MHz, DMSO-d$_6$): δ 158.7 (C_{quat}), 144.0 (C_{quat}), 143.0 (C_{quat}), 134.5 (C_{quat}), 131.2 (C_{quat}), 130.1 (C_{quat}), 128.9 (CH), 127.2 (CH), 125.8 (C_{quat}), 125.5 (CH), 124.6 (CH), 123.3 (CH), 117.3 (CH), 116.2 (CH), 114.3 (CH), 113.6 (C_{quat}), 55.1 (CH_3), 46.6 (CH_2), 30.8 (CH_2), 26.1 (CH_2), 25.8 (CH_2), 22.1 (CH_2), 13.8 (CH_3). MS (MALDI-TOF) *m*/*z*: 467.108 ([M]$^+$). IR: $\tilde{\nu}$ [cm^{-1}] = 3053 (w), 2953 (w), 2926 (w), 2853 (w), 1607 (w), 1518 (w), 1489 (m), 1456 (s), 1441 (m), 1391 (m), 1377 (w), 1352 (w), 1331 (w), 1290 (m), 1279 (m), 1261 (m), 1238 (s), 1179 (m), 1148 (w), 1107 (m), 1082 (w), 1047 (m), 1026 (w), 866 (w), 833 (m), 804 (s), 785 (m), 754 (m), 725 (w), 685 (w), 667 (w), 638 (w). Anal. calcd. for $C_{25}H_{26}BrNOS$ [468.5]: C 64.10, H 5.59, N 2.99, S 6.84; Found: C 64.37, H 5.58, N 2.91, S 6.99.

2.2.8 3-Bromo-10-hexyl-7-(5-methylthiophen-2-yl)-10*H*-phenothiazine (4m)

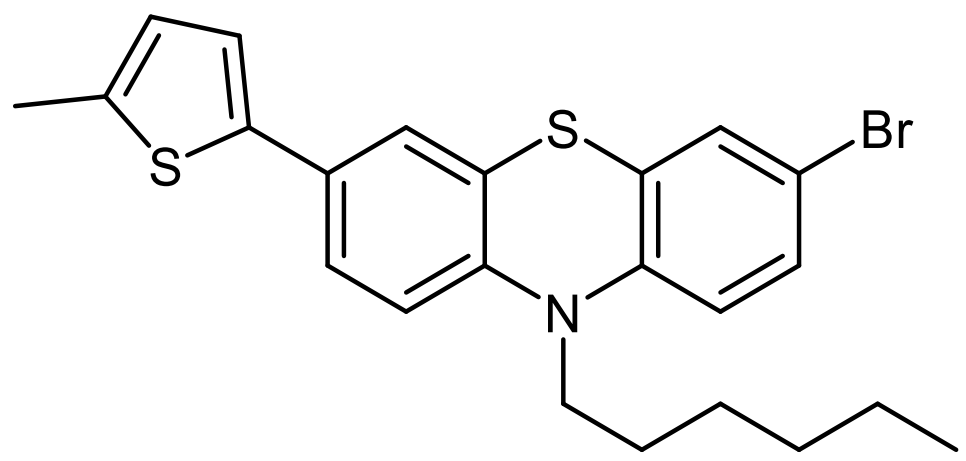

$C_{23}H_{24}BrNS_2$

[458.48]

The crude product was synthesized according to **GPI** and purified by column chromatography on silica gel (petroleum ether). Compound **4m** was obtained as a yellow oil in a yield of 2.42 g (5.28 mmol, 75%), R_f (petroleum ether) = 0.29.

^{1}H NMR (600 MHz, acetone-d$_6$): δ 7.39 (dd, $^3J_{HH}$ = 8.5 Hz, $^4J_{HH}$ = 2.2 Hz, 1H), 7.34 (d, $^4J_{HH}$ = 2.2 Hz, 1H), 7.32 (dd, $^3J_{HH}$ = 8.7 Hz, $^4J_{HH}$ = 2.3 Hz, 1H), 7.28 (d, $^4J_{HH}$ = 2.4 Hz, 1H), 7.14 (d, $^3J_{HH}$ = 3.6 Hz, 1H), 7.01 (d, $^3J_{HH}$ = 8.5 Hz, 1H), 6.94 (d, $^3J_{HH}$ = 8.7 Hz, 1H), 6.72 – 6.77 (m, 1H), 3.92 (t, $^3J_{HH}$ = 7.1 Hz, 2H), 2.47 (s, 3H), 1.73 – 1.81 (m, 2H), 1.41 – 1.49 (m, 2H), 1.24 – 1.34 (m, 4H), 0.80 – 0.89 (m, 3H). ^{13}C NMR (150 MHz, acetone-d$_6$): δ 145.5 (C_{quat}), 144.8 (C_{quat}), 141.5 (C_{quat}), 139.8 (C_{quat}), 131.2 (CH), 130.6 (C_{quat}), 130.2 (CH), 127.5 (CH), 127.5 (C_{quat}), 125.6 (CH), 125.4 (C_{quat}), 124.6 (CH), 123.6 (CH), 118.2 (CH), 117.2 (CH), 114.9 (C_{quat}), 48.1 (CH_2), 32.3 (CH_2), 27.5 (CH_2), 27.2 (CH_2), 23.4 (CH_2), 15.4 (CH_3), 14.4 (CH_3). MS (EI) *m*/*z* (%): 460 ([$M^{13}C^{81}Br]^+$, 26), 459 ([$M^{12}C^{81}Br]^+$, 82), 458 ([$M^{13}C^{79}Br]^+$, 34), 457 ([$M^{12}C^{79}Br]^+$, 100), 388 ([$C_{18}H_{13}{}^{81}BrNS_2]^+$, 16), 386 ([$C_{18}H_{13}{}^{79}BrNS_2]^+$, 15), 375 ([$^{13}C_{18}H_{13}{}^{81}BrNS_2]^+$, 23), 374 ([$^{12}C_{18}H_{13}{}^{81}BrNS_2]^+$, 99), 373 ([$^{13}C_{18}H_{13}{}^{79}BrNS_2]^+$, 27), 372 ([$^{12}C_{18}H_{13}{}^{79}BrNS_2]^+$, 97), 307 (11), 293 (11). IR: $\tilde{\nu}$ [cm^{-1}] = 3065 (w), 2953 (w), 2920 (w), 2853 (w), 1599 (w), 1503 (m), 1454 (s), 1393 (m), 1377 (w), 1329 (m), 1294 (w), 1263 (m), 1248 (m), 1238 (m), 1209 (m), 1194 (w), 1159 (w), 1146 (w), 1107 (w), 1082 (w), 1051 (w), 993 (w), 961 (w), 932 (w), 868 (m), 791 (s), 752 (m), 737 (w), 687 (w), 654 (w). Anal. calcd. for $C_{23}H_{24}BrNS_2$ [458.5]: C 60.25, H 5.28, N 3.06, S 13.99; Found: C 60.46, H 5.08, N 2.90, S 14.05.

2.2.9 7-Bromo-10,10'-dihexyl-10*H*,10'*H*-3,3'-biphenothiazine (4n)

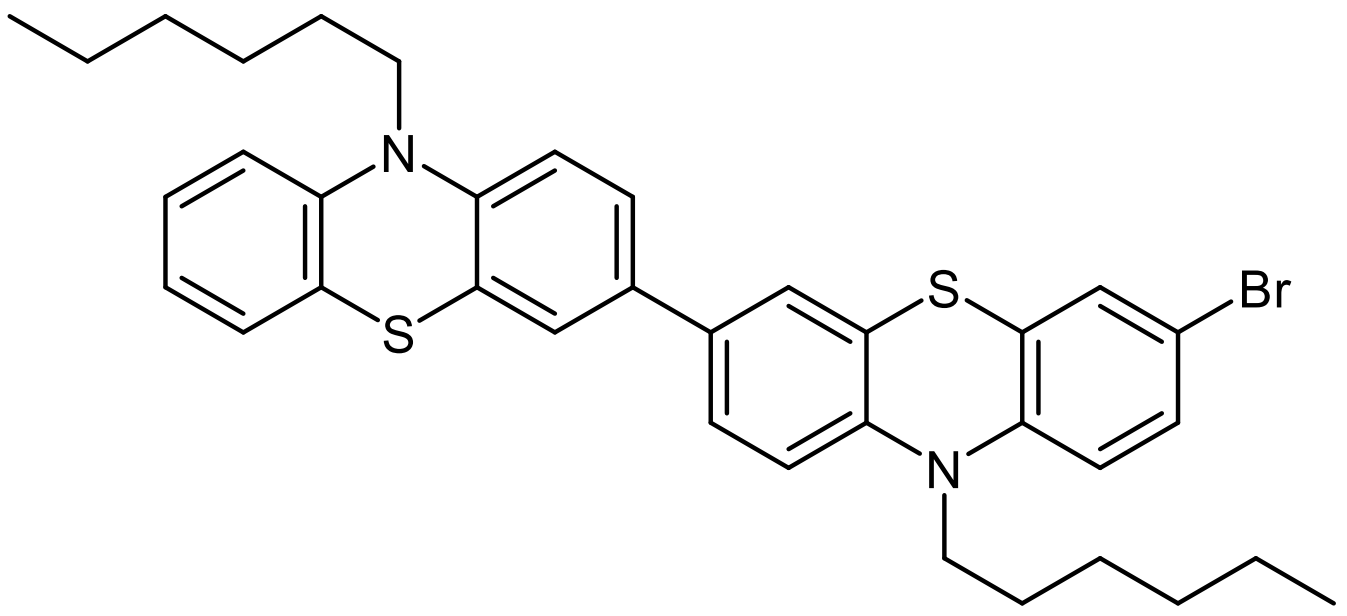

$C_{36}H_{39}BrN_2S_2$
[643.75]

The crude product was synthesized according to **GPI** and purified by column chromatography on silica gel using gradient elution (petroleum ether → petroleum ether/acetone 100:1). Compound **4n** was obtained as a yellow resin in a yield of 2.74 g (4.26 mmol, 82%), R_f (petroleum ether) = 0.17.

^{1}H NMR (600 MHz, acetone-d$_6$): δ 7.38 – 7.45 (m, 2H), 7.35 (d, $^4J_{HH}$ = 2.2 Hz, 2H), 7.30 (dd, $^3J_{HH}$ = 8.7 Hz, $^4J_{HH}$ = 2.3 Hz, 1H), 7.26 (d, $^4J_{HH}$ = 2.3 Hz, 1H), 7.16 – 7.22 (m, 1H), 7.14 (dd, $^3J_{HH}$ = 7.6 Hz, $^4J_{HH}$ = 1.6 Hz, 1H), 6.96 – 7.07 (m, 3H), 6.86 – 6.96 (m, 2H), 3.76 – 4.06 (m, 4H), 1.66 – 1.86 (m, 4H), 1.37 – 1.54 (m, 4H), 1.14 – 1.37 (m, 8H), 0.72 – 0.94 (m, 6H). ^{13}C NMR (150 MHz, acetone-d$_6$): δ 146.2 (C$_{quat}$), 145.6 (C$_{quat}$), 145.4 (C$_{quat}$), 144.8 (C$_{quat}$), 135.4 (C$_{quat}$), 134.8 (C$_{quat}$), 131.1 (CH), 130.1 (CH), 128.5 (CH), 128.2 (CH), 127.8 (C$_{quat}$), 126.5 (CH), 126.3 (CH), 126.1 (C$_{quat}$), 125.8 (CH), 125.7 (CH), 125.2 (C$_{quat}$), 123.4 (CH), 118.2 (CH), 117.2 (CH), 116.9 (CH), 116.7 (CH), 114.9 (C$_{quat}$), 48.1 (CH$_2$), 48.0 (CH$_2$), 32.3 (CH$_2$), 32.3 (CH$_2$), 27.7 (CH$_2$), 27.5 (CH$_2$), 27.3 (CH$_2$), 27.3 (CH$_2$), 23.4 (CH$_2$), 23.4 (CH$_2$), 14.4 (CH$_3$); one C$_{quat}$ and one CH$_3$ are not visible due to signal overlap. MS (EI) *m/z* (%): 646 ([M^{13}C^{81}Br]$^+$, 37), 645 ([M^{12}C^{81}Br]$^+$, 99), 644 ([M^{13}C^{79}Br]$^+$, 53), 643 ([M^{12}C^{79}Br]$^+$, 100), 642 (19), 560 ([$^{13}C_{30}H_{26}{}^{81}BrN_2S_2$]$^+$, 16), 559 ([$^{12}C_{30}H_{26}{}^{81}BrN_2S_2$]$^+$, 35), 558 ([$^{13}C_{30}H_{26}{}^{79}BrN_2S_2$]$^+$, 16), 557 ([$^{12}C_{30}H_{26}{}^{79}BrN_2S_2$]$^+$, 32), 475 ([$^{13}C_{24}H_{13}{}^{81}BrN_2S_2$]$^+$, 16), 474 ([$^{12}C_{24}H_{13}{}^{81}BrN_2S_2$]$^+$, 47), 473 ([$^{13}C_{24}H_{13}{}^{79}BrN_2S_2$]$^+$, 17), 472 ([$^{12}C_{24}H_{13}{}^{79}BrN_2S_2$]$^+$, 43), 393 ([$C_{24}H_{13}N_2S_2$]$^+$, 10). IR: $\tilde{\nu}$ [cm^{-1}] = 2953 (w), 2924 (w), 2853 (w), 1713 (w), 1599 (w), 1576 (w), 1485 (w), 1452 (s), 1416 (w), 1375 (w), 1331 (m), 1250 (m), 1238 (m), 1192 (w), 1159 (w), 1136 (w), 1105 (w), 1082 (w), 1063 (w), 1040 (w), 926 (w), 872 (w), 804 (m), 745 (m), 727 (m), 706 (w), 693 (w). Anal. calcd. for $C_{36}H_{39}BrN_2S_2$ [643.8]: C 67.17, H 6.11, N 4.35, S 9.96; Found: C 67.36, H 6.16, N 4.29, S 9.81.

2.2.10 3-Bromo-7-(4-fluorophenyl)-10-(4-methoxyphenyl)-10*H*-phenothiazine (4f)

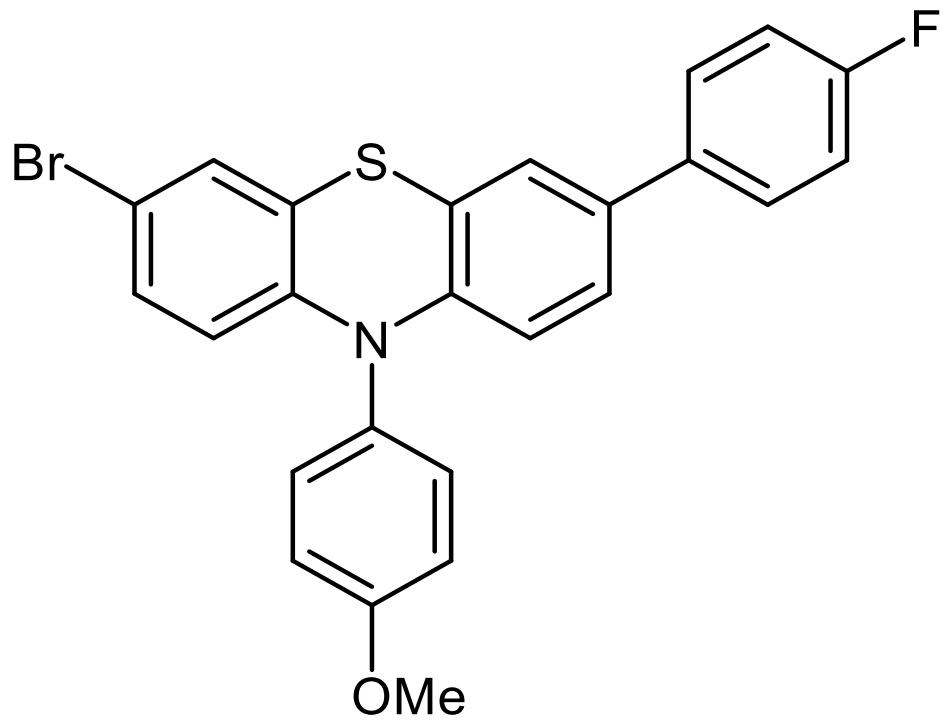

$C_{25}H_{17}BrFNOS$

[478.38]

In a dry Schlenk tube under a nitrogen atmosphere, 7-(4-fluorophenyl)-10-(4-methoxyphenyl)-10*H*-phenothiazine (2.00 g, 5.00 mmol, 1.00 equiv.) was dissolved in anhydrous THF (6.00 mL). The solution was then degassed under a stream of nitrogen for 30 min. *N*-Bromosuccinimide (0.890 g, 5.00 mmol, 1.00 equiv.) was added, and the reaction mixture was stirred for 18 h at 20 °C. Upon completion, the solvent was removed under reduced pressure. The crude product was adsorbed onto Celite® and purified by flash column chromatography on silica gel (petroleum ether/dichloromethane 100:1). Compound **4f** was obtained as a yellow solid in a yield of 2.33 g (4.87 mmol, 97%), Mp 75 – 77 °C, R_f (petroleum ether/acetone 100:1) = 0.23.

^{1}H NMR (300 MHz, acetone-d$_6$): δ 7.54 – 7.66 (m, 2H), 7.33 – 7.42 (m, 2H), 7.10 – 7.32 (m, 7H), 7.05 (dd, $^{3}J_{HH}$ = 8.8 Hz, $^{4}J_{HH}$ = 2.3 Hz, 1H), 6.25 (d, $^{3}J_{HH}$ = 8.6 Hz, 1H), 6.12 (d, $^{3}J_{HH}$ = 8.8 Hz, 1H), 3.92 (s, 3H). ^{13}C NMR (75 MHz, acetone-d$_6$): δ 163.2 (d, $^{1}J_{CF}$ = 245 Hz, C_{quat}), 160.8 (C_{quat}), 144.7 (C_{quat}), 144.3 (C_{quat}), 136.7 (d, $^{4}J_{CF}$ = 3.0 Hz, C_{quat}), 135.4 (C_{quat}), 133.5 (C_{quat}), 132.9 (CH), 130.6 (CH), 129.3 (CH), 129.0 (d, $^{3}J_{CF}$ = 7.5 Hz, CH), 126.6 (CH), 125.5 (CH), 122.6 (C_{quat}), 120.2 (C_{quat}), 118.1 (C_{quat}), 117.2 (CH), 117.1 (CH), 116.5 (d, $^{2}J_{CF}$ = 22.5 Hz, CH), 114.8 (C_{quat}), 56.1 (CH_3). MS (EI) *m/z* (%): 480 ([$M^{13}C^{81}Br$]$^+$, 27), 479 ([$M^{12}C^{81}Br$]$^+$, 100), 478 ([$M^{13}C^{79}Br$]$^+$, 35), 477 ([$M^{12}C^{79}Br$]$^+$, 83), 476 (12), 399 ([$^{13}C_{25}H_{17}FNOS$]$^+$, 10), 398 ([$^{12}C_{25}H_{17}FNOS$]$^+$, 25). IR: $\tilde{\nu}$ [cm^{-1}] = 2835 (w), 1603 (w), 1508 (m), 1487 (m), 1456 (s), 1420 (w), 1379 (w), 1290 (m), 1242 (s), 1229 (s), 1180 (w), 1157 (m), 1101 (w), 1074 (w), 1030 (m), 1013 (w), 868 (w), 835 (m), 800 (s), 766 (w), 746 (w), 723 (w), 679 (w), 662 (w), 642 (w), 610 (m). Anal. calcd. for $C_{25}H_{17}BrFNOS$ [478.4]: C 62.77, H 3.58, N 2.93, S 6.70; Found: C 63.10, H 3.80, N 2.65, S 6.79.

2.3 Synthesis of target compounds

2.3.1 General procedure (GPII) of the BLEBS sequence for the synthesis of phenyl-expanded tetrakis(phenothiazinyl)silanes 5

In a dry Schlenk tube under a nitrogen atmosphere, phenothiazine bromide **4** (4.20 equiv.) was dissolved in anhydrous THF (6.00 mL/mmol) and cooled to –78 °C. *n*-Butyllithium (4.60 equiv., 1.6 M in *n*-hexane) was added dropwise, and the reaction mixture was stirred for 30 min at –78 °C. Subsequently, trimethyl borate (5.20 equiv.) was added. The cooling bath was removed, and the mixture was stirred at 20 °C for 1 h. Afterward, tetrakis(4-bromophenyl)silane **1** (1.00 equiv.), tetrakis(triphenylphosphane)palladium(0) (20 mol%), sodium hydroxide (5.20 equiv.), and distilled water (2.00 mL/mmol) were added. The reaction was then heated at 100 °C and stirred for 18 h. Upon completion, the organic phase was separated, and the aqueous phase was extracted three times with dichloromethane. The combined organic phases were dried (anhydrous magnesium sulfate) and the solvents were removed in vacuo. The crude product was adsorbed onto Celite® and purified by flash column chromatography on silica gel. Recrystallization was performed where necessary to afford the pure product.

Table S2. Experimental details for the synthesis of phenyl-expanded tetrakis(phenothiazinyl)silanes **5**.

entry	phenothiazine bromide **4** [mg] (mmol)	silane **1** [mg] (mmol)	yield of product **5** [mg] (%)
1	368 (1.26) of **4a**	196 (0.30)	222 (63) of **5a**
2	403 (1.26) of **4b**	196 (0.30)	191 (49) of **5b**
3	761 (2.10) of **4c**	326 (0.50)	538 (74) of **5c**
4	516 (0.84) of **4d**	130 (0.20)	315 (64) of **5d**
5	464 (1.26) of **4e**	196 (0.30)	222 (50) of **5e**
6	502 (1.05) of **4f**	163 (0.25)	314 (65) of **5f**
7	638 (1.26) of **4g**	196 (0.30)	392 (64) of **5g**
8	596 (1.26) of **4h**	196 (0.30)	449 (79) of **5h**
9	575 (1.26) of **4i**	196 (0.30)	478 (87) of **5i**

10	552 (1.26) of **4j**	196 (0.30)	286 (54) of **5j**
11	380 (0.84) of **4k**	130 (0.20)	263 (72) of **5k**
12	590 (1.26) of **4l**	196 (0.30)	423 (75) of **5l**
13	578 (1.26) of **4m**	196 (0.30)	399 (72) of **5m**
14	541 (0.84) of **4n**	130 (0.20)	279 (54) of **5n**
15	474 (1.26) of **4o**	196 (0.30)	343 (75) of **5o**
16	510 (1.30) of **4p**	203 (0.31)	335 (68) of **5p**
17	515 (1.26) of **4q**	196 (0.30)	314 (64) of **5q**
18	380 (1.05) of **4r**	163 (0.25)	275 (75) of **5r**

2.3.2 Tetrakis(4-(10-methyl-10*H*-phenothiazin-3-yl)phenyl)silane (5a)

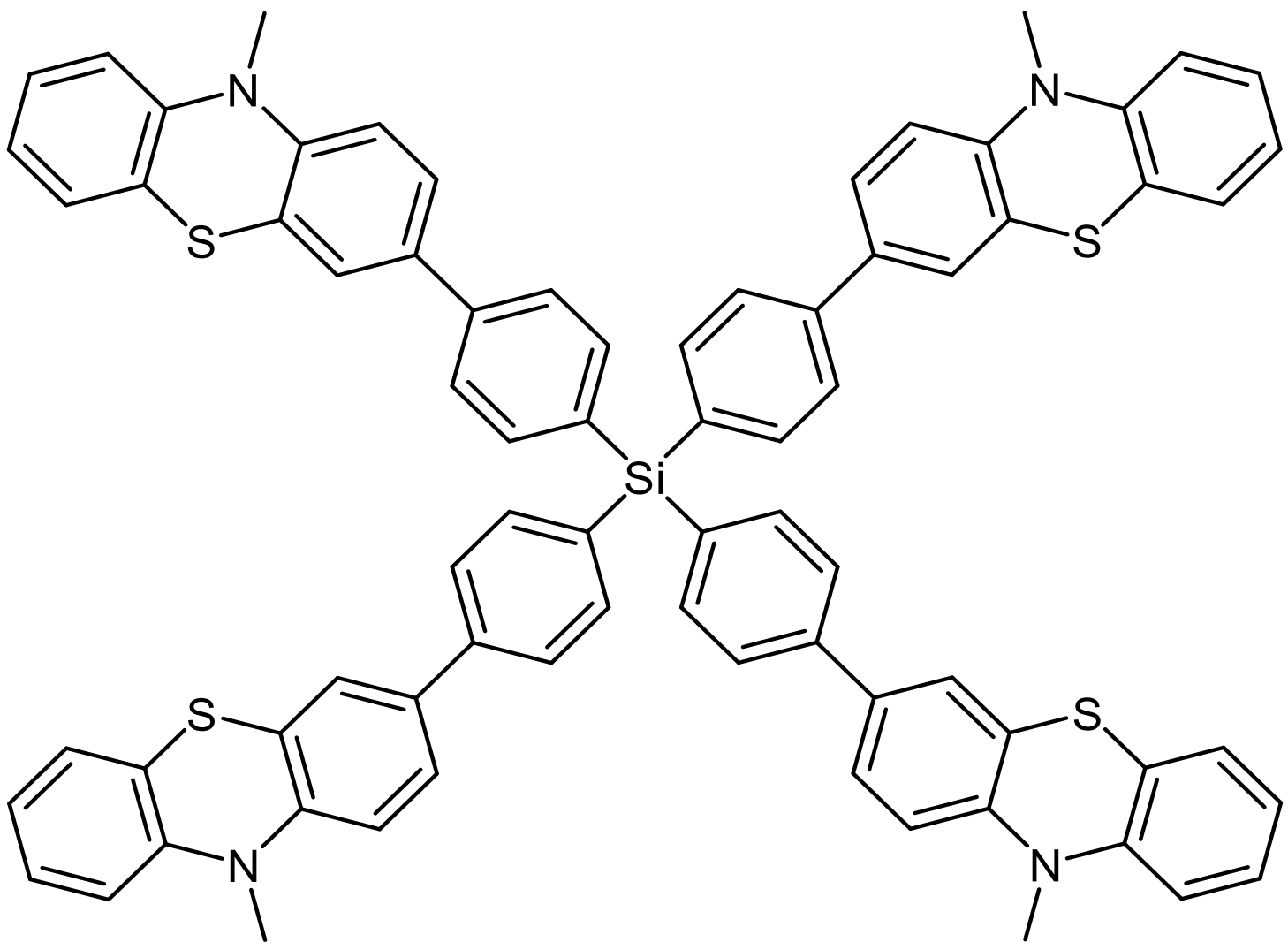

$C_{76}H_{56}N_4S_4Si$

[1181.64]

The crude product was synthesized according to **GPII** and purified by column chromatography on silica gel using gradient elution (petroleum ether/dichloromethane 3:1 → 1:1), followed by recrystallization from a mixture of *n*-pentane and dichloromethane. Compound **5a** was obtained as a pale-yellow solid in a yield of 222 mg (0.188 mmol, 63%), Mp 330 – 333 °C (decomposition), R_f (petroleum ether/dichloromethane 2:1) = 0.10.

^{1}H NMR (600 MHz, THF-d_8): δ 7.59 – 7.70 (m, 16H), 7.47 (dd, $^3J_{HH}$ = 8.4 Hz, $^4J_{HH}$ = 2.2 Hz, 4H), 7.43 (d, $^4J_{HH}$ = 2.2 Hz, 4H), 7.14 (td, $^3J_{HH}$ = 7.7 Hz, $^4J_{HH}$ = 1.5 Hz, 4H), 7.07 – 7.12 (m, 4H), 6.95 (d, $^3J_{HH}$ = 8.5 Hz, 4H), 6.89 (t, $^3J_{HH}$ = 7.4 Hz, 8H), 3.38 (s, 12H). ^{13}C NMR (150 MHz, THF-d_8): δ 146.8 (C_{quat}), 146.6 (C_{quat}), 142.3 (C_{quat}), 137.9 (CH), 136.1 (C_{quat}), 133.8 (C_{quat}), 128.4 (CH), 127.8 (CH), 127.0 (CH), 126.8 (CH), 126.1 (CH), 125.0 (C_{quat}), 124.2 (C_{quat}), 123.3 (CH), 115.4 (CH), 115.2 (CH), 35.7 (CH_3). MS (MALDI-TOF) *m/z*: 1180.317 ($[M]^+$). IR: $\tilde{\nu}$ [cm^{-1}] = 3063 (w), 3013 (w), 2963 (w), 2872 (w), 2818 (w), 1595 (w), 1576 (w), 1512 (w), 1487 (w), 1460 (s), 1404 (w), 1383 (w), 1333 (m), 1292 (w), 1271 (w), 1258 (m), 1196 (w), 1163 (w), 1142 (m), 1107 (m), 1040 (w), 1015 (w), 926 (w), 876 (w), 827 (w), 804 (s), 741 (s), 719 (m), 706 (m), 677 (w), 640 (w), 631 (w), 608 (w). Anal. calcd. for $C_{76}H_{56}N_4S_4Si$ [1181.6]: C 77.25, H 4.78, N 4.74, S 10.85; Found: C 77.04, H 4.91, N 4.46, S 10.79.

2.3.3 Tetrakis(4-(10-isopropyl-10*H*-phenothiazin-3-yl)phenyl)silane (5b)

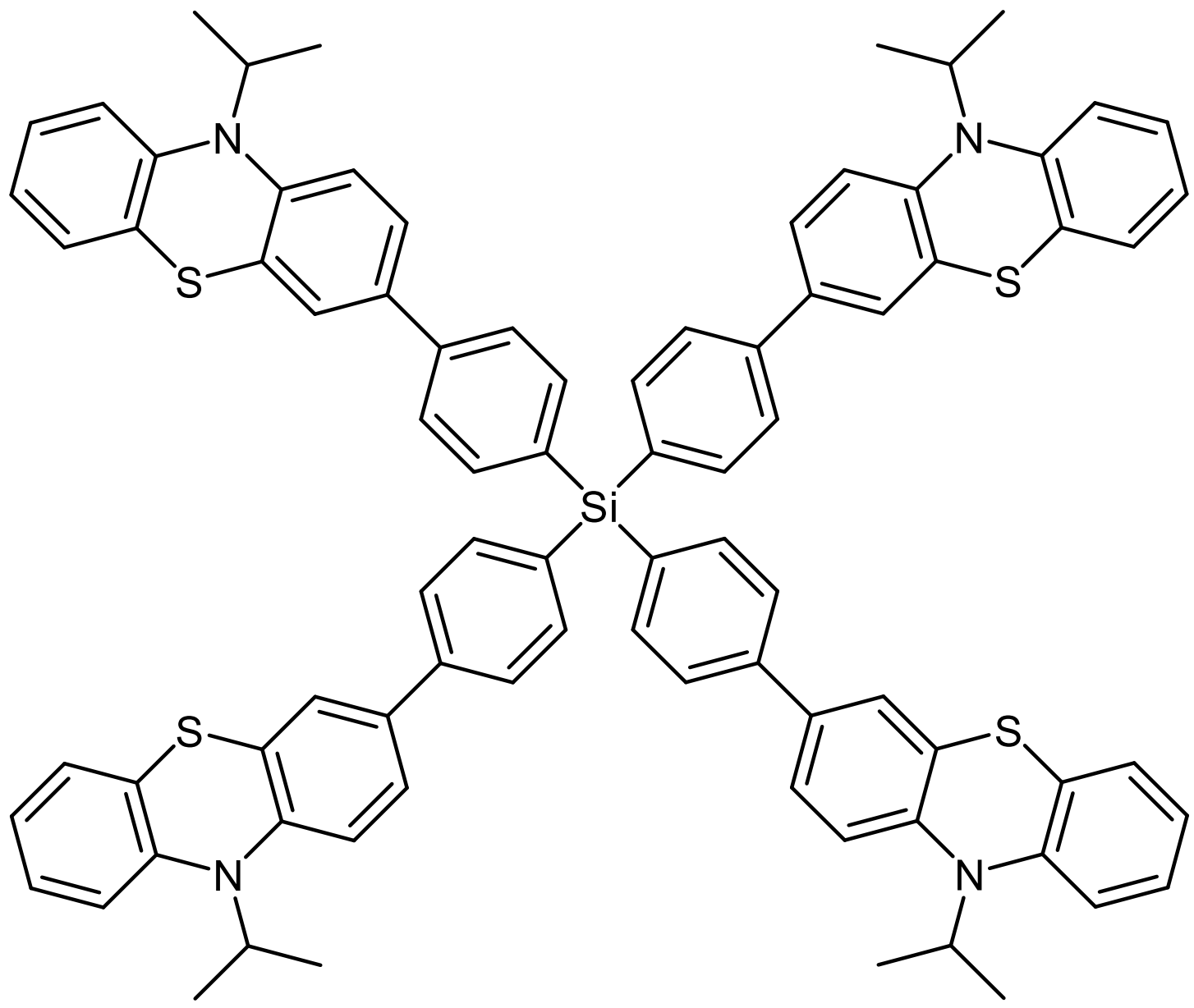

$C_{84}H_{72}N_4S_4Si$

[1293.85]

The crude product was synthesized according to **GPII** and purified by column chromatography on silica gel using gradient elution (petroleum ether/dichloromethane 5:1 → 2:1), followed by recrystallization from a mixture of *n*-pentane and dichloromethane. Compound **5b** was obtained as a pale-yellow solid in a yield of 191 mg (0.148 mmol, 49%), Mp 258 – 260 °C, R_f (petroleum ether/dichloromethane 2:1) = 0.43.

^{1}H NMR (300 MHz, THF-d$_8$): δ 7.57 – 7.74 (m, 16H), 7.38 – 7.50 (m, 8H), 7.18 (d, $^3J_{HH}$ = 8.4 Hz, 4H), 7.05 – 7.15 (m, 12H), 6.86 – 6.93 (m, 4H), 4.34 (hept, $^3J_{HH}$ = 6.8 Hz, 4H), 1.64 (d, $^3J_{HH}$ = 6.9 Hz, 24H). ^{13}C NMR (75 MHz, THF-d$_8$): δ 146.2 (C_{quat}), 145.9 (C_{quat}), 142.2 (C_{quat}), 137.8 (CH), 136.2 (C_{quat}), 133.7 (C_{quat}), 128.3 (C_{quat}), 128.0 (CH), 127.9 (CH), 127.4 (C_{quat}), 126.7 (CH), 126.4 (CH), 126.2 (CH), 123.5 (CH), 119.1 (CH), 118.9 (CH), 54.6 (CH), 22.5 (CH_3). MS (MALDI-TOF) *m/z*: 1292.550 ([M]$^+$). IR: $\tilde{\nu}$ [cm^{-1}] = 3057 (w), 3013 (w), 2972 (w), 2932 (w), 2874 (w), 1595 (w), 1576 (w), 1508 (w), 1487 (w), 1462 (s), 1441 (m), 1408 (w), 1391 (w), 1368 (w), 1335 (w), 1298 (m), 1269 (m), 1236 (m), 1196 (w), 1171 (w), 1115 (s), 1084 (w), 1042 (w), 1015 (w), 995 (w), 928 (w), 903 (w), 885 (w), 829 (w), 806 (s), 746 (s), 737 (m), 718 (m), 704 (m), 673 (m), 648 (w), 606 (m). Anal. calcd. for $C_{84}H_{72}N_4S_4Si$ [1293.9]: C 77.98, H 5.61, N 4.33, S 9.91; Found: C 78.08, H 5.58, N 4.11, S 9.82.

2.3.4 Tetrakis(4-(10-hexyl-10*H*-phenothiazin-3-yl)phenyl)silane (5c)

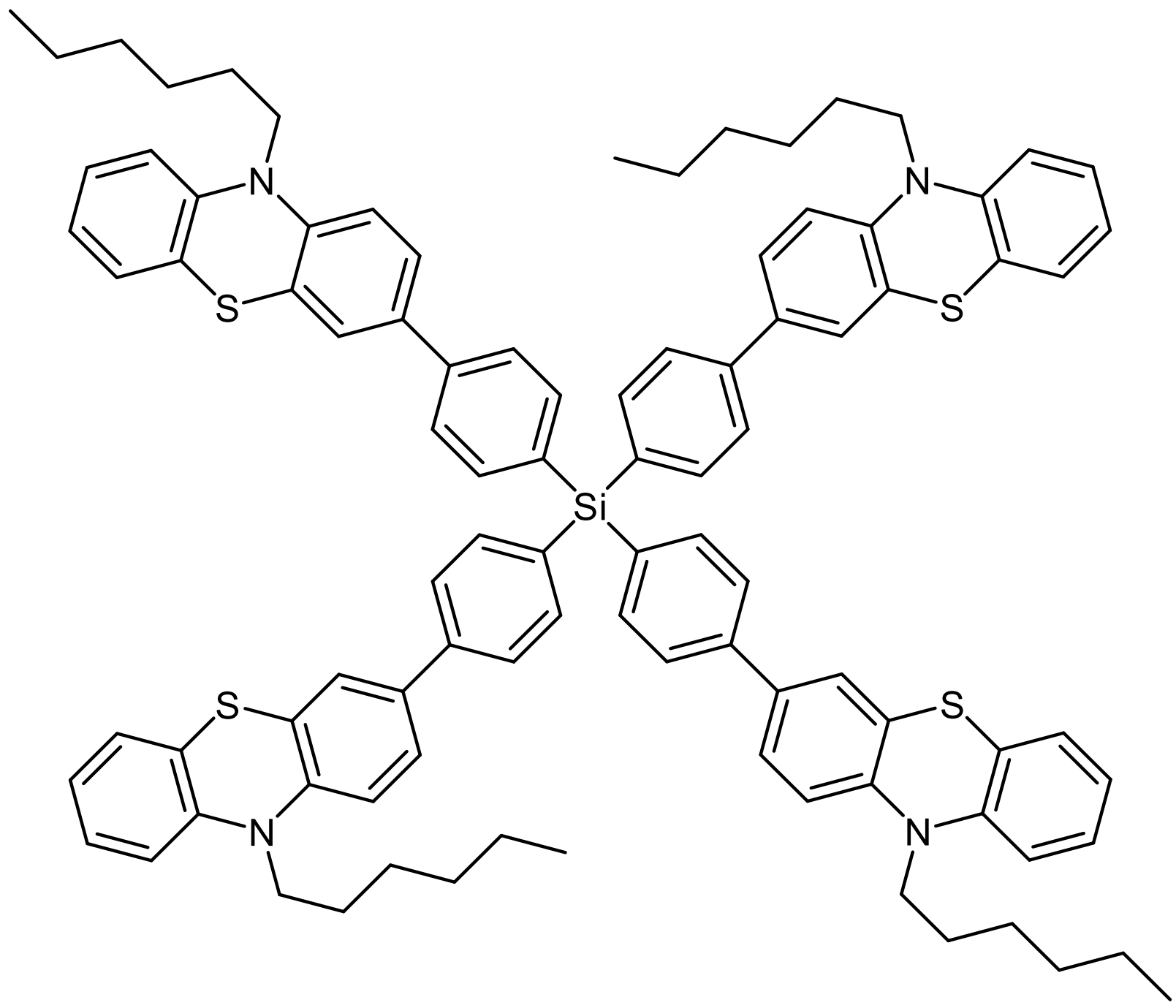

$C_{96}H_{96}N_4S_4Si$

[1462.18]

The crude product was synthesized according to **GPII** and purified by column chromatography on silica gel using gradient elution (petroleum ether/dichloromethane 5:1 → 4:1). Compound **5c** was obtained as a pale-yellow solid in a yield of 538 mg (0.368 mmol, 74%), Mp 169 – 171 °C, R_f (petroleum ether/dichloromethane 4:1) = 0.20.

^{1}H NMR (600 MHz, THF-d$_8$): δ 7.68 (d, $^3J_{HH}$ = 6.6 Hz, 8H), 7.62 (d, $^3J_{HH}$ = 6.7 Hz, 8H), 7.46 (dd, $^3J_{HH}$ = 8.4 Hz, $^4J_{HH}$ = 1.8 Hz, 4H), 7.42 (s, 4H), 7.10 – 7.15 (m, 4H), 7.09 (d, $^3J_{HH}$ = 7.6 Hz, 4H), 7.01 (d, $^3J_{HH}$ = 7.3 Hz, 4H), 6.95 (d, $^3J_{HH}$ = 8.2 Hz, 4H), 6.87 (t, $^3J_{HH}$ = 7.5 Hz, 4H), 3.93 (t, $^3J_{HH}$ = 7.0 Hz, 8H), 1.82 (pent, $^3J_{HH}$ = 7.1 Hz, 8H), 1.47 (pent, $^3J_{HH}$ = 6.7 Hz, 8H), 1.27 – 1.36 (m, 16H), 0.84 – 0.91 (m, 12H). ^{13}C NMR (150 MHz, THF-d$_8$): δ 146.2 (C_{quat}), 146.1 (C_{quat}), 142.3 (C_{quat}), 137.9 (CH), 136.1 (C_{quat}), 133.7 (C_{quat}), 128.2 (CH), 126.8 (CH), 126.5 (C_{quat}), 126.5 (CH), 125.7 (C_{quat}), 123.3 (CH), 116.7 (CH), 116.5 (CH), 48.2 (CH_2), 32.6 (CH_2), 28.0 (CH_2), 27.6 (CH_2), 23.7 (CH_2), 14.5 (CH_3); two CH are not visible, likely due to signal overlap caused by the high number of signals. ^{29}Si NMR (119 MHz, THF-d$_8$): δ 14.4. MS (MALDI-TOF) *m*/*z*: 1460.701 ([M]$^+$). IR: $\tilde{\nu}$ [cm^{-1}] = 2957 (w), 2924 (w), 2853 (w), 1595 (m), 1576 (w), 1510 (w), 1464 (s), 1443 (m), 1406 (m), 1381 (w), 1362 (m), 1290 (m), 1269 (m), 1246 (m), 1240 (m), 1188 (m), 1163 (w), 1140 (w), 1107 (m), 1034 (w), 1015 (w), 891 (w), 804 (s), 743 (s), 718 (m), 710 (m), 679 (w), 644 (m), 625 (m). Anal. calcd. for $C_{96}H_{96}N_4S_4Si$ [1462.2]: C 78.86, H 6.62, N 3.83, S 8.77; Found: C 78.85, H 6.62, N 3.77, S 8.70.

2.3.5 Tetrakis(4-(10-(2-decyltetradecyl)-10*H*-phenothiazin-3-yl)phenyl)silane (5d)

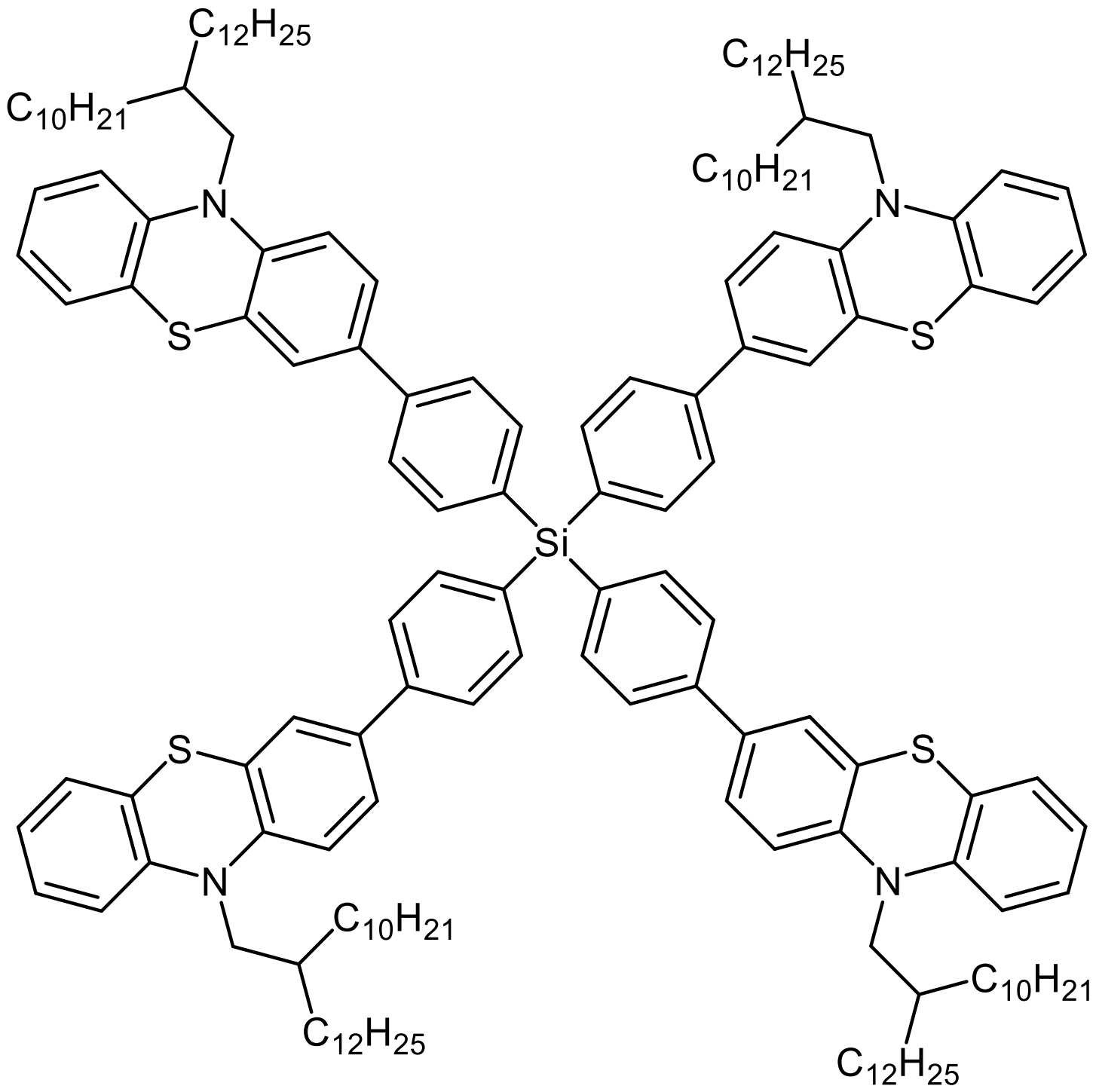

$C_{168}H_{240}N_4S_4Si$

[2472.12]

The crude product was synthesized according to **GPII** and purified by column chromatography on silica gel using gradient elution (petroleum ether/dichloromethane 100:0 → 20:1). Compound **5d** was obtained as a pale-yellow resin in a yield of 315 mg (0.127 mmol, 64%), R_f (petroleum ether/dichloromethane 10:1) = 0.34.

^{1}H NMR (300 MHz, THF-d$_8$): δ 7.65 – 7.72 (m, 8H), 7.56 – 7.65 (m, 8H), 7.42 – 7.51 (m, 8H), 7.08 – 7.19 (m, 8H), 7.04 (d, $^3J_{HH}$ = 8.4 Hz, 4H), 6.97 (d, $^3J_{HH}$ = 7.9 Hz, 4H), 6.89 (t, $^3J_{HH}$ = 7.4 Hz, 4H), 3.83 (t, $^3J_{HH}$ = 7.1 Hz, 8H), 1.95 – 2.11 (m, 4H), 1.22 – 1.56 (m, 160H), 0.82 – 0.93 (m, 24H). ^{13}C NMR (75 MHz, THF-d$_8$): δ 146.7 (C_{quat}), 146.6 (C_{quat}), 142.4 (C_{quat}), 137.9 (CH), 136.2 (C_{quat}), 133.7 (C_{quat}), 128.4 (CH), 128.1 (CH), 127.4 (C_{quat}), 126.8 (CH), 126.7 (C_{quat}), 126.6 (CH), 123.4 (CH), 117.2 (CH), 117.1 (CH), 52.4 (CH_2), 35.8 (CH), 33.0 (CH_2), 32.7 (CH_2), 31.1 (CH_2), 30.8 (CH_2), 30.8 (CH_2), 30.6 (CH_2), 30.5 (CH_2), 27.3 (CH_2), 23.7 (CH_2), 14.6 (CH_3); one CH and one CH_2 are not visible due to signal overlap. MS (MALDI-TOF) *m*/*z*: 2469.967 ([M]$^+$). IR: $\tilde{\nu}$ [cm^{-1}] = 3063 (w), 3015 (w), 2953 (w), 2920 (s), 2851 (m), 1597 (w), 1576 (w), 1537 (w), 1510 (w), 1487 (w), 1458 (s), 1443 (m), 1406 (w), 1377 (w), 1337 (w), 1290 (w), 1267 (w), 1250 (m), 1196 (w), 1161 (w), 1132 (w), 1105 (m), 1038 (w), 1016 (w), 926 (w), 885 (w), 831 (w), 806 (m), 741 (s), 721 (m), 704 (m), 681 (w), 644 (w), 629 (w), 617 (w). Anal. calcd. for $C_{168}H_{240}N_4S_4Si$ [2472.1]: C 81.62, H 9.79, N 2.27, S 5.19; Found: C 81.73, H 9.68, N 2.38, S 5.11.

2.3.6 Tetrakis(4-(10-benzyl-10*H*-phenothiazin-3-yl)phenyl)silane (5e)

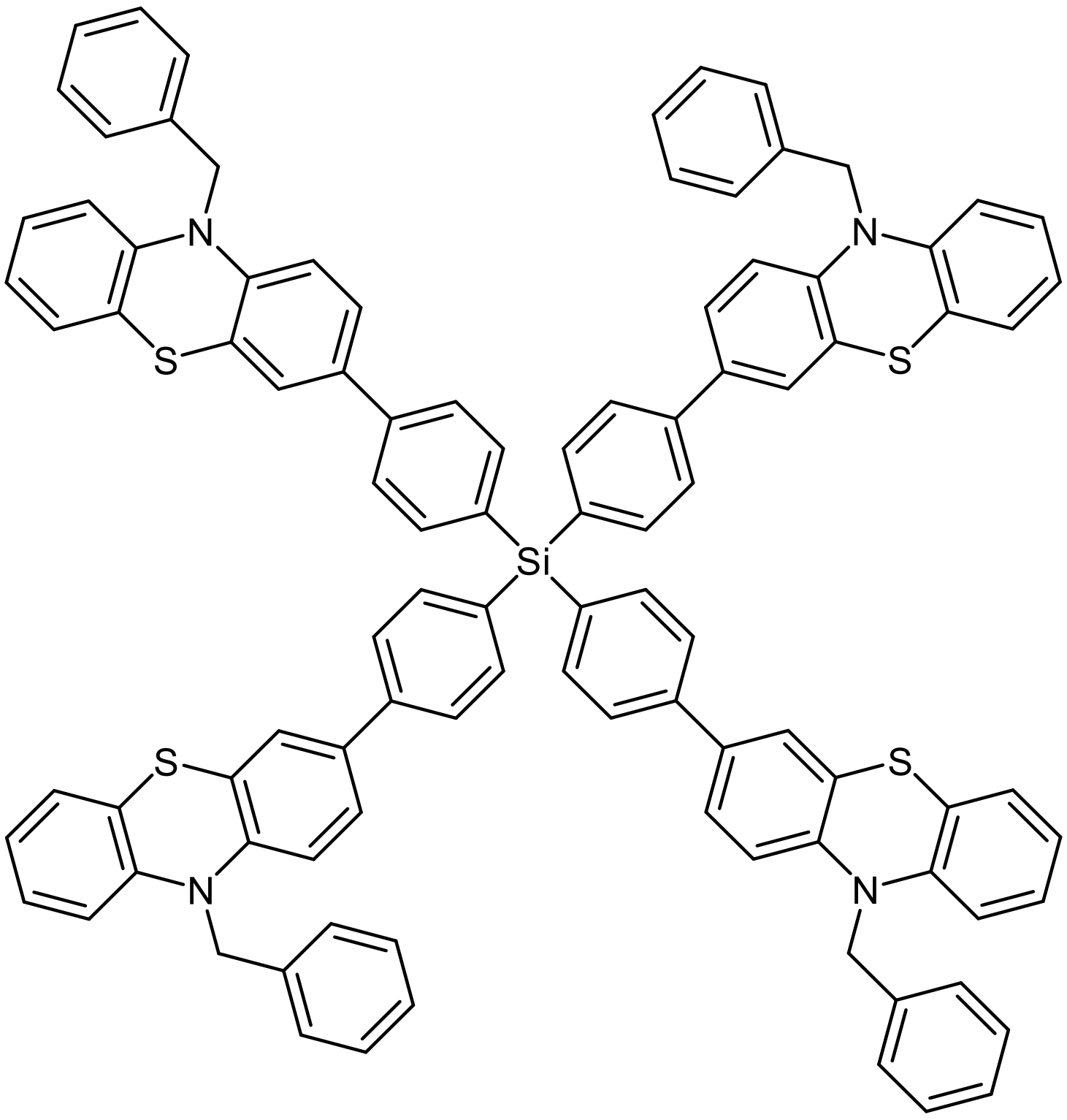

$C_{100}H_{72}N_4S_4Si$
[1486.03]

The crude product was synthesized according to **GPII** and purified by column chromatography on silica gel using gradient elution (petroleum ether/dichloromethane 6:1 → 1:1), followed by recrystallization from a mixture of *n*-pentane and dichloromethane. Compound **5e** was obtained as a pale-yellow solid in a yield of 222 mg (0.149 mmol, 50%), Mp 240 – 242 °C (decomposition), R_f (petroleum ether/dichloromethane 2:1) = 0.31.

^{1}H NMR (600 MHz, THF-d$_8$): δ 7.58 – 7.65 (m, 8H), 7.52 – 7.58 (m, 8H), 7.36 – 7.41 (m, 4H), 7.31 – 7.36 (m, 8H), 7.23 – 7.31 (m, 12H), 7.16 – 7.23 (m, 4H), 7.05 (dt, $^3J_{HH}$ = 7.6 Hz, $^4J_{HH}$ = 1.4 Hz, 4H), 6.91 – 6.98 (m, 4H), 6.79 – 6.86 (m, 4H), 6.76 (dd, $^3J_{HH}$ = 8.6 Hz, $^4J_{HH}$ = 1.8 Hz, 4H), 6.70 (dd, $^3J_{HH}$ = 8.3 Hz, $^4J_{HH}$ = 1.2 Hz, 4H), 5.14 (s, 8H). ^{13}C NMR (150 MHz, THF-d$_8$): δ 145.5 (C_{quat}), 145.3 (C_{quat}), 142.1 (C_{quat}), 138.1 (C_{quat}), 137.9 (CH), 136.2 (C_{quat}), 133.7 (C_{quat}), 129.6 (CH), 128.2 (CH), 127.8 (CH), 127.7 (CH), 127.7 (CH), 126.7 (CH), 126.7 (CH), 125.9 (CH), 124.9 (C_{quat}), 124.0 (C_{quat}), 123.5 (CH), 116.8 (CH), 116.6 (CH), 53.1 (CH_2). MS (MALDI-TOF) *m*/*z*: 1483.757 ([M]$^+$). IR: $\tilde{\nu}$ [cm^{-1}] = 3021 (w), 1597 (w), 1576 (w), 1535 (w), 1510 (w), 1489 (w), 1466 (s), 1443 (m), 1406 (w), 1364 (m), 1337 (w), 1269 (w), 1252 (m), 1219 (m), 1196 (w), 1163 (w), 1134 (w), 1109 (m), 1076 (w), 1049 (w), 1005 (w), 970 (w), 926 (w), 883 (w), 833 (w), 804 (m), 743 (m), 727 (s), 694 (m), 683 (w), 658 (m), 638 (w), 627 (m). Anal.

calcd. for $C_{100}H_{72}N_4S_4Si$ [1486.0]: C 80.83, H 4.88, N 3.77, S 8.63; Found: C 80.49, H 4.93, N 3.44, S 8.23.

2.3.7 Tetrakis(4-(7-(4-fluorophenyl)-10-(4-methoxyphenyl)-10*H*-phenothiazin-3-yl)phenyl)silane (5f)

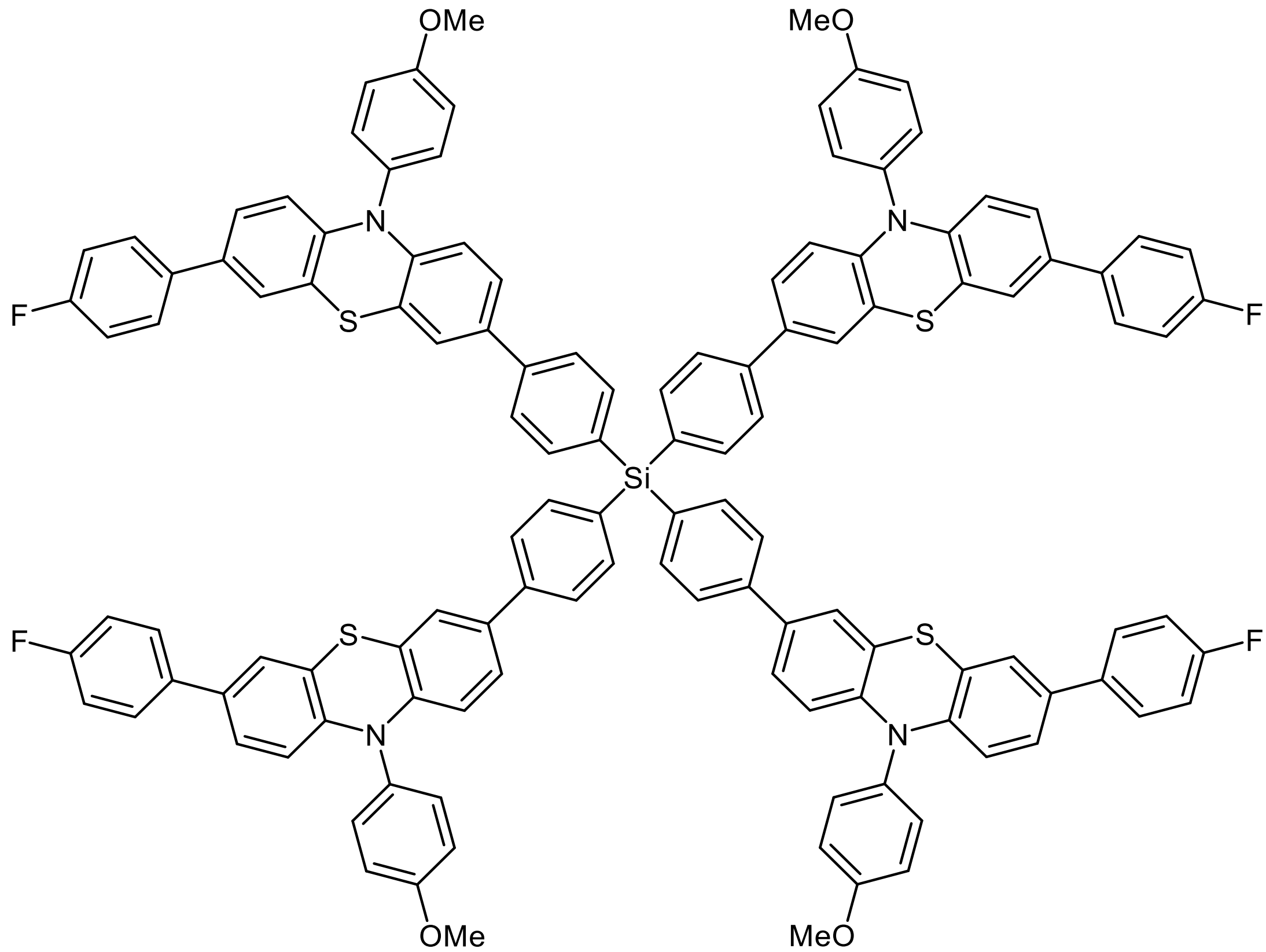

$C_{124}H_{84}F_4N_4O_4S_4Si$
[1926.38]

The crude product was synthesized according to **GPII** and purified by column chromatography on silica gel using gradient elution (petroleum ether/dichloromethane 5:1 → 1:1), followed by recrystallization from a mixture of *n*-pentane and dichloromethane. Compound **5f** was obtained as a pale-yellow solid in a yield of 314 mg (0.163 mmol, 65%), Mp 208 – 210 °C (decomposition), R_f (petroleum ether/dichloromethane 2:1) = 0.60.

^{1}H NMR (600 MHz, THF-d$_8$): δ 7.46 – 7.68 (m, 28H), 7.16 – 7.44 (m, 24H), 7.00 – 7.15 (m, 12H), 6.24 (t, $^3J_{HH}$ = 8.5 Hz, 8H), 3.88 (s, 12H). ^{13}C NMR (150 MHz, THF-d$_8$): δ 163.4 (d, $^1J_{CF}$ = 245.1 Hz, C_{quat}), 160.9 (C_{quat}), 145.0 (C_{quat}), 144.7 (C_{quat}), 142.0 (C_{quat}), 137.9 (CH), 137.2 (d, $^4J_{CF}$ = 3.2 Hz, C_{quat}), 136.0 (C_{quat}), 135.4 (C_{quat}), 134.2 (C_{quat}), 133.7 (C_{quat}), 133.1 (CH), 128.8 (d, $^3J_{CF}$ = 8.0 Hz, CH), 126.6 (CH), 126.3 (CH), 126.1 (CH), 125.7 (CH), 125.6 (CH), 121.0 (C_{quat}), 120.9 (C_{quat}), 117.1 (CH), 117.0 (CH), 116.9 (CH), 116.4 (d, $^2J_{CF}$ = 21.5 Hz, CH), 56.0

(CH_3). MS (MALDI-TOF) *m/z*: 1924.959 ($[M]^+$). IR: $\tilde{\nu}$ [cm^{-1}] = 3022 (w), 2833 (w), 1595 (w), 1508 (m), 1487 (m), 1462 (s), 1381 (w), 1308 (m), 1287 (m), 1240 (s), 1231 (s), 1179 (w), 1157 (m), 1111 (m), 1076 (w), 1032 (m), 1015 (w), 914 (w), 881 (w), 835 (m), 804 (s), 791 (m), 773 (w), 735 (w), 708 (m), 681 (w), 664 (w), 640 (w), 613 (m). Anal. calcd. for $C_{124}H_{84}F_4N_4O_4S_4Si$ [1926.4]: C 77.31, H 4.40, N 2.91, S 6.66; Found: C 77.43, H 4.61, N 2.60, S 6.35.

2.3.8 Tetrakis(4-(10-hexyl-7-(4-(trifluoromethyl)phenyl)-10*H*-phenothiazin-3-yl)phenyl)silane (5g)

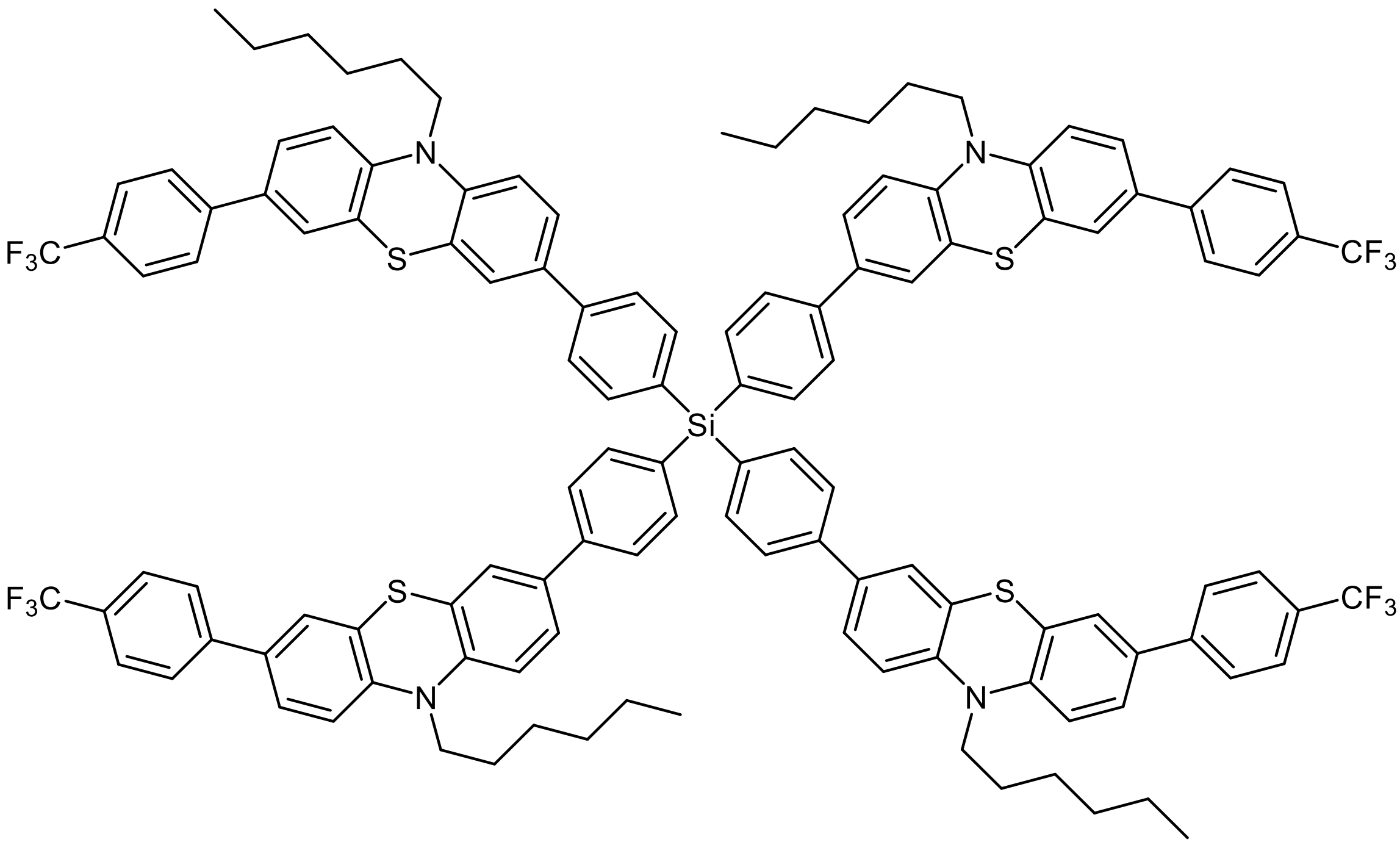

$C_{124}H_{108}F_{12}N_4S_4Si$

[2038.56]

The crude product was synthesized according to **GPII** and purified by column chromatography on silica gel using gradient elution (petroleum ether/dichloromethane 4:1 → 1:1), followed by recrystallization from a mixture of *n*-pentane and dichloromethane. Compound **5g** was obtained as a pale-yellow solid in a yield of 392 mg (0.192 mmol, 64%), Mp 168 – 170 °C, R_f (petroleum ether/dichloromethane 5:1) = 0.10.

^{1}H NMR (300 MHz, THF-d$_8$): δ 7.58 – 7.83 (m, 32H), 7.43 – 7.55 (m, 16H), 7.06 (d, $^3J_{HH}$ = 8.7 Hz, 8H), 3.97 (t, $^3J_{HH}$ = 7.1 Hz, 8H), 1.85 (pent, $^3J_{HH}$ = 7.4 Hz, 8H), 1.43 – 1.57 (m, 8H), 1.27 – 1.39 (m, 16H), 0.83 – 0.96 (m, 12H). ^{13}C NMR (75 MHz, THF-d$_8$): δ 146.2 (C_{quat}), 145.4 (C_{quat}), 144.6 (C_{quat}), 142.1 (C_{quat}), 137.8 (CH), 136.2 (C_{quat}), 134.5 (C_{quat}), 133.7 (C_{quat}), 129.6 (q, $^2J_{CF}$ = 32.1 Hz, C_{quat}), 129.2 (C_{quat}), 127.5 (CH), 127.4 (CH), 127.0 (CH), 126.9 (q, $^3J_{CF}$ = 3.9 Hz, CH), 126.7 (CH), 126.5 (CH), 126.1 (CH), 125.8 (q, $^1J_{CF}$ = 270.7 Hz, C_{quat}), 123.8 (C_{quat}), 116.7

(CH), 116.7 (CH), 48.2 (CH_2), 32.5 (CH_2), 27.8 (CH_2), 27.5 (CH_2), 23.6 (CH_2), 14.4 (CH_3). ^{29}Si NMR (119 MHz, THF-d_8): δ 14.4. MS (MALDI-TOF) *m/z*: 2036.940 ([M]$^+$). IR: $\tilde{\nu}$ [cm^{-1}] = 2955 (w), 2930 (w), 2855 (w), 1616 (w), 1597 (w), 1585 (w), 1464 (s), 1420 (w), 1406 (w), 1389 (w), 1321 (s), 1285 (m), 1261 (m), 1250 (m), 1192 (w), 1163 (m), 1109 (s), 1070 (s), 1035 (w), 1015 (m), 883 (w), 843 (m), 804 (s), 762 (w), 723 (m), 711 (m), 692 (w), 648 (w). Anal. calcd. for $C_{124}H_{108}F_{12}N_4S_4Si$ [2038.6]: C 73.06, H 5.34, N 2.75, S 6.29; Found: C 73.48, H 5.61, N 2.65, S 6.26.

2.3.9 Tetrakis(4-(7-(4-chlorophenyl)-10-hexyl-10*H*-phenothiazin-3-yl)phenyl)silane (5h)

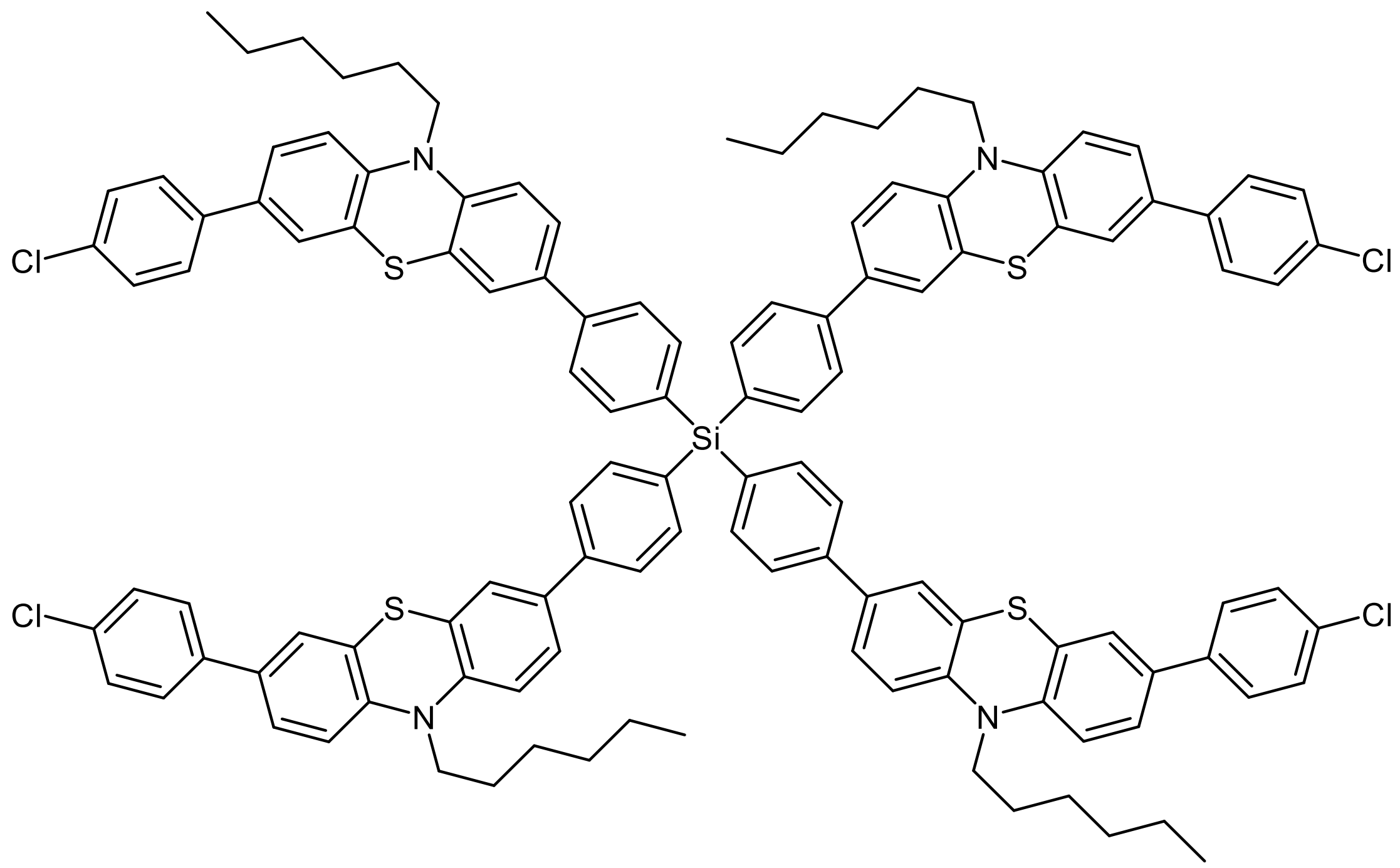

$C_{120}H_{108}Cl_4N_4S_4Si$

[1904.34]

The crude product was synthesized according to **GPII** and purified by column chromatography on silica gel using gradient elution (petroleum ether/dichloromethane 5:1 → 1:1), followed by recrystallization from a mixture of *n*-pentane and dichloromethane. Compound **5h** was obtained as a pale-yellow solid in a yield of 449 mg (0.236 mmol, 79%), Mp 200 – 202 °C. R_f (petroleum ether/dichloromethane 2:1) = 0.56.

^{1}H NMR (600 MHz, THF-d_8): δ 7.67 – 7.71 (m, 8H), 7.61 – 7.65 (m, 8H), 7.54 – 7.59 (m, 8H), 7.47 (dd, $^3J_{HH}$ = 8.5 Hz, $^4J_{HH}$ = 2.2 Hz, 4H), 7.45 (d, $^4J_{HH}$ = 2.2 Hz, 4H), 7.41 (d, $^3J_{HH}$ = 7.9 Hz, 8H), 7.36 – 7.40 (m, 8H), 6.99 – 7.06 (m, 8H), 3.95 (t, $^3J_{HH}$ = 7.1 Hz, 8H), 1.79 – 1.87 (m, 8H), 1.43 – 1.53 (m, 8H), 1.27 – 1.38 (m, 16H), 0.85 – 0.92 (m, 12H). ^{13}C NMR (150 MHz, THF-d_8): δ 145.7 (C_{quat}), 142.3 (C_{quat}), 139.7 (C_{quat}), 137.9 (CH), 136.2 (C_{quat}), 135.1 (C_{quat}), 133.8 (C_{quat}),

133.7 (C_{quat}), 129.8 (CH), 128.7 (CH), 126.9 (CH), 126.8 (CH), 126.7 (CH), 126.5 (CH), 126.3 (CH), 126.2 (C_{quat}), 126.0 (C_{quat}), 116.8 (CH), 116.7 (CH), 48.3 (CH_2), 32.6 (CH_2), 27.9 (CH_2), 27.6 (CH_2), 23.7 (CH_2), 14.5 (CH_3); one C_{quat} is not visible, likely due to signal overlap caused by the high number of signals. MS (MALDI-TOF) *m*/*z*: 1902.857 ($[M]^+$). IR: $\tilde{\nu}$ [cm^{-1}] = 2951 (w), 2922 (w), 2853 (w), 1595 (w), 1464 (s), 1406 (m), 1385 (m), 1358 (m), 1269 (m), 1246 (m), 1192 (w), 1109 (m), 1094 (m), 1034 (w), 1011 (m), 883 (w), 831 (m), 802 (s), 721 (m), 652 (m), 617 (w). Anal. calcd. for $C_{120}H_{108}Cl_4N_4S_4Si$ [1904.3]: C 75.69, H 5.72, N 2.94, S 6.73; Found: C 75.42, H 5.55, N 2.84, S 7.05.

2.3.10 Tetrakis(4-(7-(4-fluorophenyl)-10-hexyl-10*H*-phenothiazin-3-yl)phenyl)silane (5i)

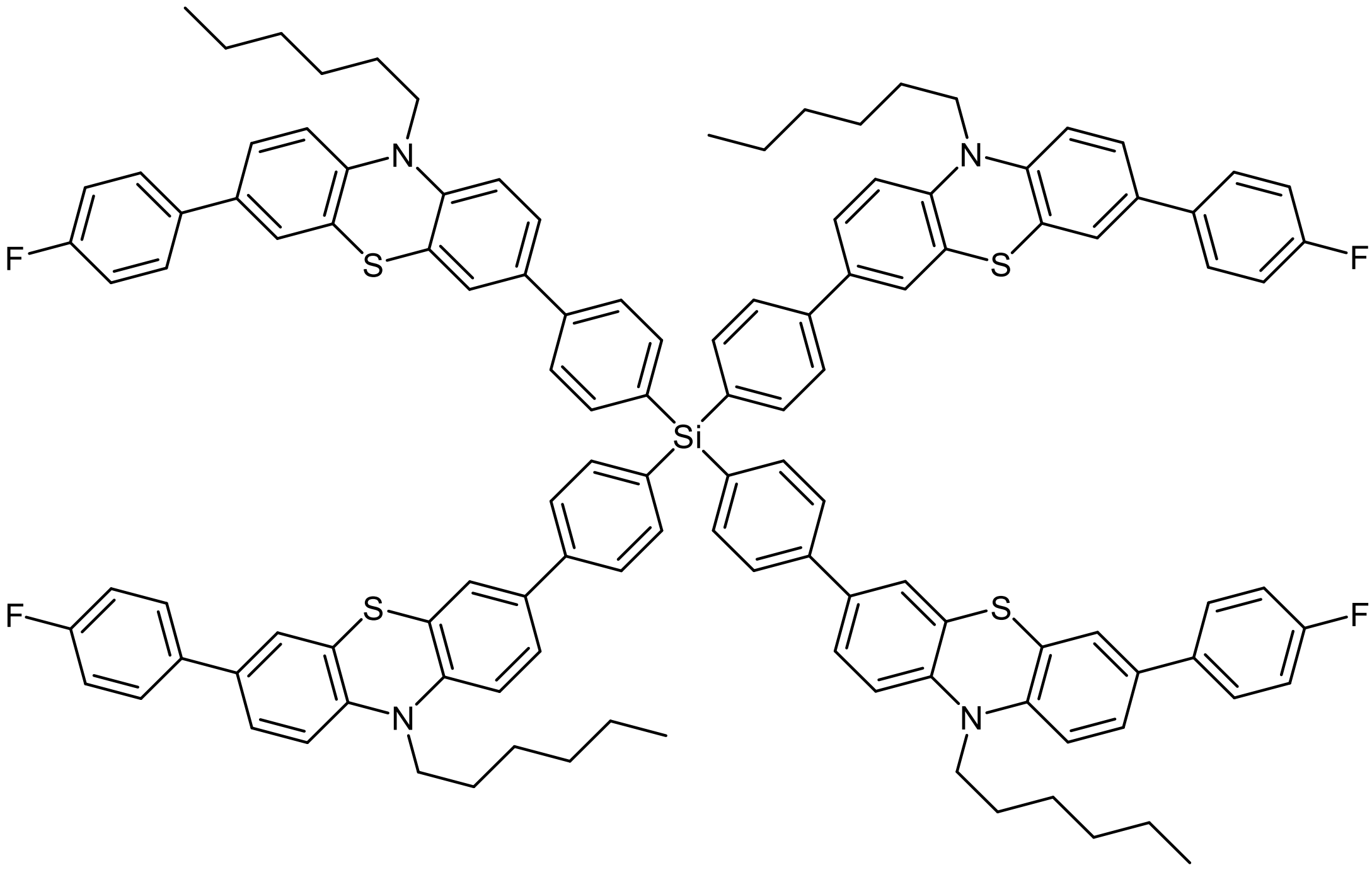

$C_{120}H_{108}F_4N_4S_4Si$
[1838.53]

The crude product was synthesized according to **GPII** and purified by column chromatography on silica gel using gradient elution (petroleum ether/dichloromethane 5:1 → 1:1), followed by recrystallization from a mixture of *n*-pentane and dichloromethane. Compound **5i** was obtained as a pale-yellow solid in a yield of 478 mg (0.260 mmol, 87%), Mp 163 – 165 °C, R_f (petroleum ether/dichloromethane 2:1) = 0.60.

^{1}H NMR (300 MHz, THF-d$_8$): δ 7.61 – 7.74 (m, 16H), 7.53 – 7.61 (m, 8H), 7.43 – 7.52 (m, 8H), 7.35 – 7.43 (m, 8H), 7.07 – 7.17 (m, 8H), 7.03 (t, $^3J_{HH}$ = 8.4 Hz, 8H), 3.96 (t, $^3J_{HH}$ = 7.0 Hz, 8H), 1.84 (pent, $^3J_{HH}$ = 7.2 Hz, 8H), 1.42 – 1.55 (m, 8H), 1.24 – 1.42 (m, 16H), 0.83 – 0.94 (m, 12H). ^{13}C NMR (75 MHz, THF-d$_8$): δ 145.7 (d, $^1J_{CF}$ = 244.2 Hz, C_{quat}), 145.3 (C_{quat}), 142.2 (C_{quat}),

137.8 (CH), 137.3 (d, $^4J_{CF}$ = 3.4 Hz, C_{quat}), 136.0 (C_{quat}), 135.4 (C_{quat}), 133.7 (C_{quat}), 129.0 (d, $^3J_{CF}$ = 7.9 Hz, CH), 128.8 (CH), 126.8 (CH), 126.7 (CH), 126.5 (CH), 126.4 (CH), 126.3 (CH), 126.0 (C_{quat}), 125.9 (C_{quat}), 116.6 (d, $^2J_{CF}$ = 21.4 Hz, CH), 116.4 (C_{quat}), 116.1 (CH), 48.1 (CH_2), 32.5 (CH_2), 27.8 (CH_2), 27.5 (CH_2), 23.6 (CH_2), 14.4 (CH_3). MS (MALDI-TOF) *m*/*z*: 1836.979 ($[M]^+$). IR: $\tilde{\nu}$ [cm^{-1}] = 2953 (w), 2924 (w), 2851 (w), 1595 (w), 1468 (s), 1423 (w), 1406 (w), 1387 (w), 1362 (w), 1263 (w), 1246 (m), 1231 (s), 1190 (w), 1159 (w), 1109 (m), 1016 (w), 889 (w), 835 (w), 802 (s), 721 (m), 652 (m). Anal. calcd. for $C_{120}H_{108}F_4N_4S_4Si$ [1838.5]: C 78.40, H 5.92, N 3.05, S 6.98; Found: C 78.64, H 5.92, N 2.90, S 6.79.

2.3.11 Tetrakis(4-(10-hexyl-7-phenyl-10*H*-phenothiazin-3-yl)phenyl)silane (5j)

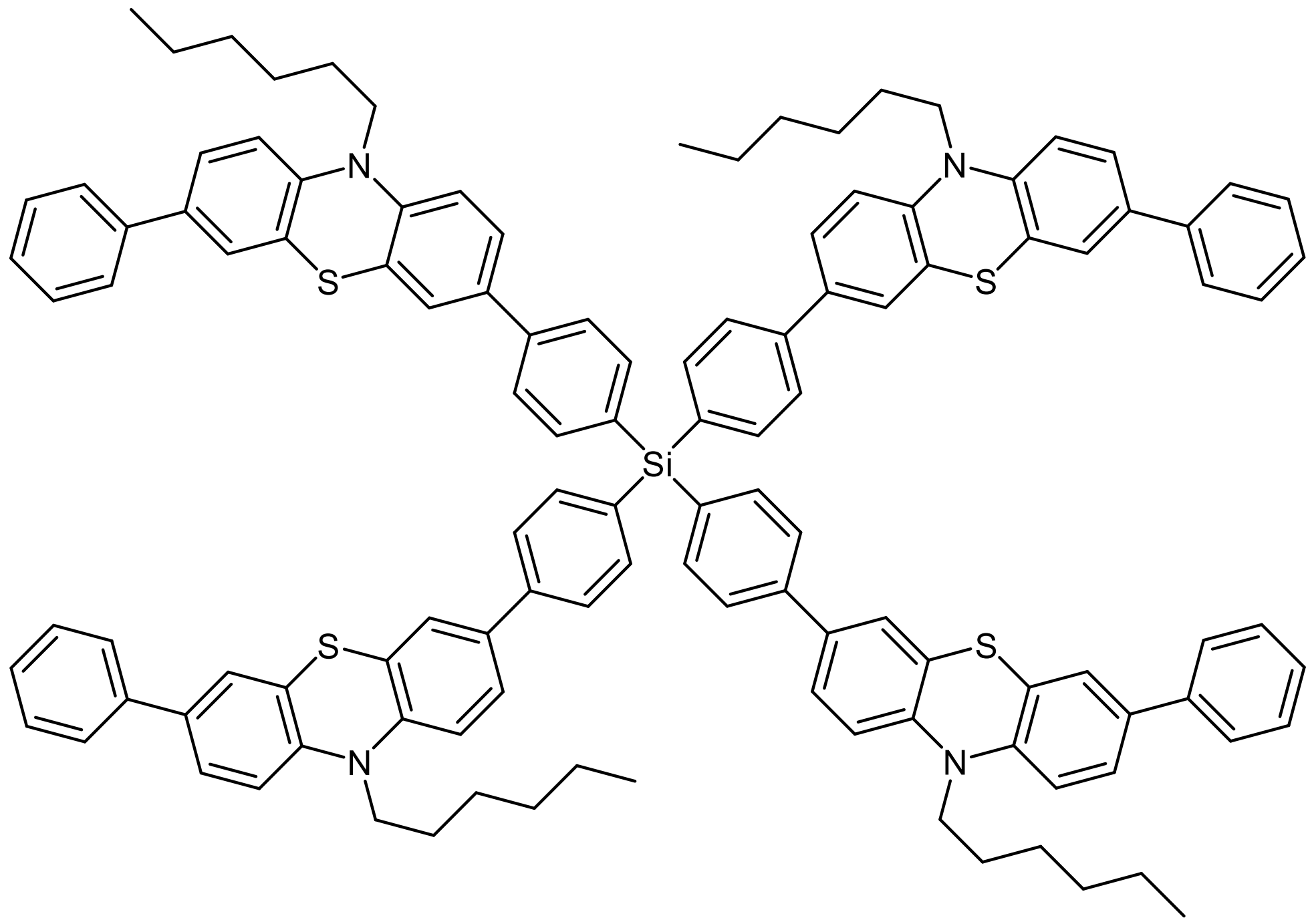

$C_{120}H_{112}N_4S_4Si$
[1766.57]

The crude product was synthesized according to **GPII** and purified by column chromatography on silica gel using gradient elution (petroleum ether/dichloromethane 5:1 → 1:1), followed by recrystallization from a mixture of *n*-pentane and dichloromethane. Compound **5j** was obtained as a pale-yellow solid in a yield of 286 mg (0.162 mmol, 54%), Mp 210 – 213 °C, R_f (petroleum ether/dichloromethane 2:1) = 0.53.

^{1}H NMR (600 MHz, THF-d_8): δ 7.66 (d, $^3J_{HH}$ = 7.8 Hz, 8H), 7.59 (d, $^3J_{HH}$ = 7.9 Hz, 8H), 7.51 (d, $^3J_{HH}$ = 7.6 Hz, 8H), 7.32 – 7.46 (m, 24H), 7.23 (t, $^3J_{HH}$ = 7.3 Hz, 4H), 6.93 – 7.00 (m, 8H), 3.93 (t, $^3J_{HH}$ = 7.1 Hz, 8H), 1.85 (pent, $^3J_{HH}$ = 7.3 Hz, 8H), 1.47 – 1.53 (m, 8H), 1.31 – 1.40 (m, 16H), 0.86 – 0.94 (m, 12H). ^{13}C NMR (150 MHz, THF-d_8): δ 145.5 (C_{quat}), 145.1 (C_{quat}), 142.1 (C_{quat}),

140.9 (C_{quat}), 137.9 (CH), 136.4 (C_{quat}), 136.0 (C_{quat}), 133.6 (C_{quat}), 129.6 (CH), 127.8 (CH), 127.2 (CH), 126.8 (CH), 126.8 (CH), 126.7 (CH), 126.5 (CH), 126.5 (CH), 126.1 (C_{quat}), 126.0 (C_{quat}), 116.5 (CH), 116.5 (CH), 48.3 (CH_2), 32.7 (CH_2), 28.0 (CH_2), 27.7 (CH_2), 23.9 (CH_2), 14.8 (CH_3). ^{29}Si NMR (119 MHz, THF-d_8): δ 14.3. MS (MALDI-TOF) *m*/*z*: 1765.087 ($[M]^+$). IR: $\tilde{\nu}$ [cm^{-1}] = 2990 (s), 2972 (s), 2901 (s), 1595 (w), 1506 (w), 1470 (s), 1406 (m), 1366 (m), 1321 (w), 1306 (w), 1246 (m), 1229 (m), 1173 (w), 1152 (w), 1107 (m), 1074 (s), 1053 (s), 1028 (m), 804 (m), 760 (m), 719 (m), 692 (m). Anal. calcd. for $C_{120}H_{112}N_4S_4Si$ [1766.6]: C 81.59, H 6.39, N 3.17, S 7.26; Found: C 81.40, H 6.37, N 3.04, S 7.05.

2.3.12 Tetrakis(4-(10-hexyl-7-(*p*-tolyl)-10*H*-phenothiazin-3-yl)phenyl)silane (5k)

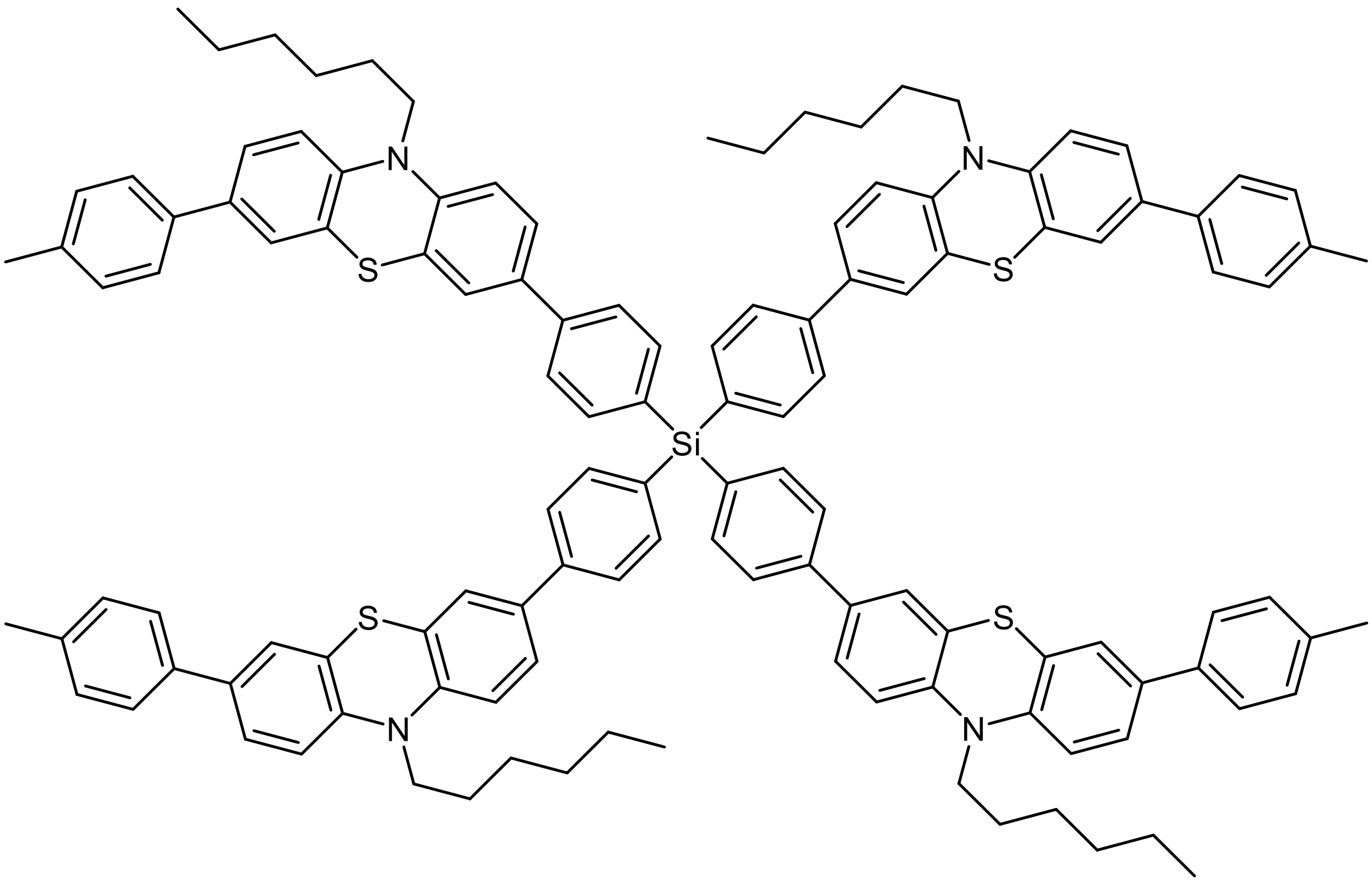

$C_{124}H_{120}N_4S_4Si$
[1822.68]

The crude product was synthesized according to **GPII** and purified by column chromatography on silica gel using gradient elution (petroleum ether/dichloromethane 5:1 → 1:1), followed by recrystallization from a mixture of *n*-pentane and dichloromethane. Compound **5k** was obtained as a pale-yellow solid in a yield of 263 mg (0.144 mmol, 72%), Mp 151 – 154 °C, R_f (petroleum ether/dichloromethane 10:1) = 0.30.

^{1}H NMR (300 MHz, THF-d_8): δ 7.58 – 7.73 (m, 16H), 7.29 – 7.54 (m, 24H), 7.18 (d, $^3J_{HH}$ = 7.9 Hz, 8H), 7.00 (t, $^3J_{HH}$ = 9.1 Hz, 8H), 3.93 (t, $^3J_{HH}$ = 7.1 Hz, 8H), 2.32 (s, 12H), 1.76 – 1.91 (m, 8H), 1.41 – 1.56 (m, 8H), 1.25 – 1.41 (m, 16H), 0.81 – 0.97 (m, 12H). ^{13}C NMR (75 MHz, THF-d_8): δ 145.9 (C_{quat}), 145.1 (C_{quat}), 142.3 (C_{quat}), 138.2 (C_{quat}), 137.9 (CH), 137.4 (C_{quat}), 136.5

(C_{quat}), 136.1 (C_{quat}), 133.8 (C_{quat}), 130.3 (CH), 128.2 (CH), 127.1 (CH), 126.9 (CH), 126.8 (CH), 126.5 (CH), 126.5 (CH), 126.2 (CH), 126.1 (C_{quat}), 125.9 (C_{quat}), 116.6 (CH), 48.2 (CH_2), 32.6 (CH_2), 27.9 (CH_2), 27.6 (CH_2), 23.7 (CH_2), 21.3 (CH_3), 14.5 (CH_3). MS (MALDI-TOF) *m*/*z*: 1820.932 ($[M]^+$). IR: $\tilde{\nu}$ [cm^{-1}] = 3022 (w), 2953 (w), 2922 (w), 2853 (w), 1595 (w), 1537 (w), 1462 (s), 1408 (w), 1389 (m), 1377 (m), 1333 (m), 1310 (w), 1288 (m), 1248 (m), 1192 (m), 1142 (w), 1111 (m), 1034 (w), 1016 (w), 883 (w), 802 (s), 772 (w), 710 (m), 689 (w), 664 (w). Anal. calcd. for $C_{124}H_{120}N_4S_4Si$ [1822.7]: C 81.71, H 6.64, N 3.07, S 7.04; Found: C 81.91, H 6.89, N 2.95, S 6.89.

2.3.13 Tetrakis(4-(10-hexyl-7-(4-methoxyphenyl)-10*H*-phenothiazin-3-yl)phenyl)silane (5l)

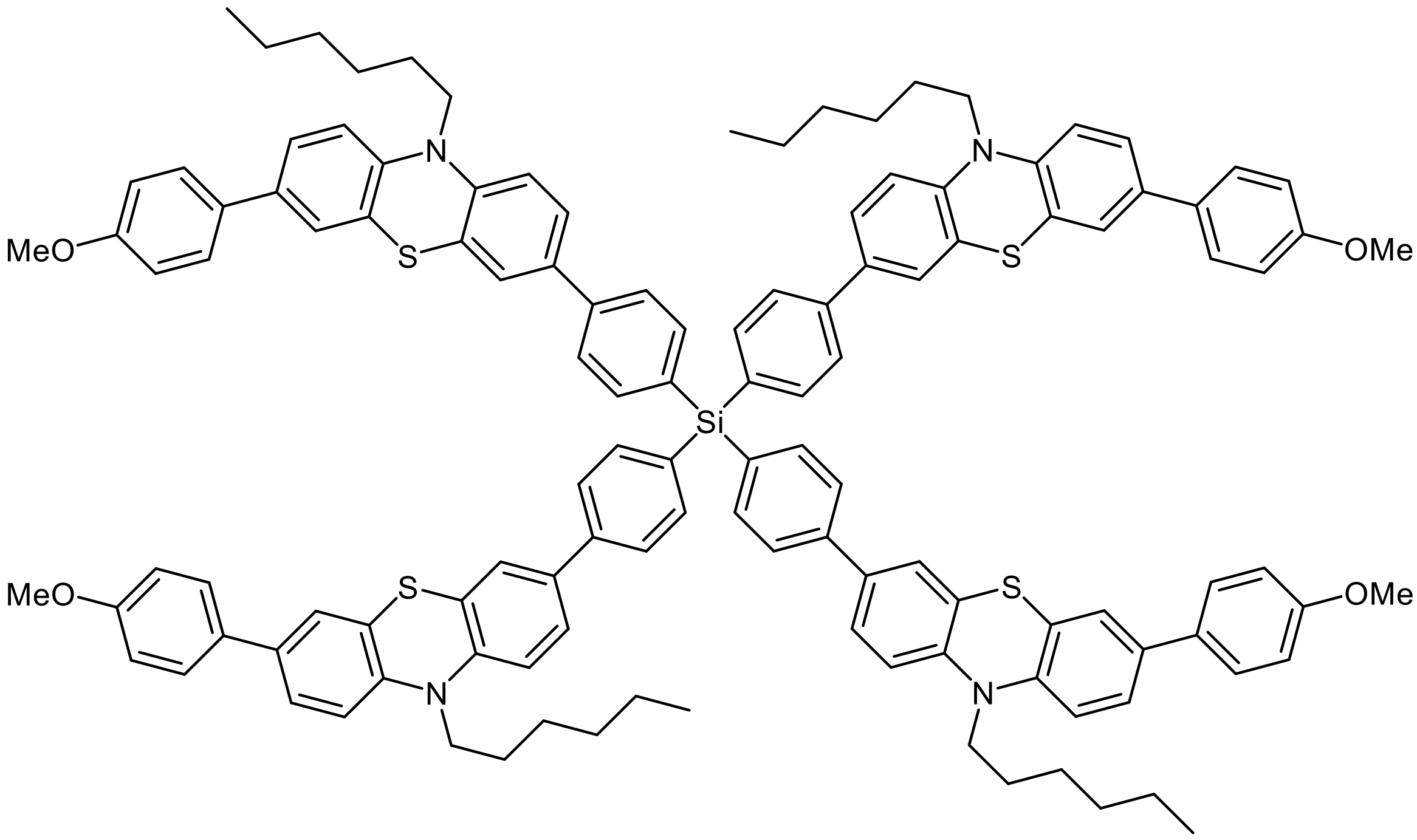

$C_{124}H_{120}N_4O_4S_4Si$

[1886.67]

The crude product was synthesized according to **GPII** and purified by column chromatography on silica gel using gradient elution (petroleum ether/dichloromethane 5:1 → 1:1), followed by recrystallization from a mixture of *n*-pentane and dichloromethane. Compound **5l** was obtained as a pale-yellow solid in a yield of 423 mg (0.224 mmol, 75%), Mp 156 – 158 °C, R_f (petroleum ether/dichloromethane 2:1) = 0.32.

^1H NMR (300 MHz, THF-d_8): δ 7.58 – 7.74 (m, 16H), 7.40 – 7.57 (m, 16H), 7.31 – 7.40 (m, 8H), 6.86 – 7.09 (m, 16H), 3.94 (t, $^3J_{HH}$ = 7.0 Hz, 8H), 3.78 (s, 12H), 1.84 (pent, $^3J_{HH}$ = 7.3 Hz, 8H), 1.42 – 1.58 (m, 8H), 1.27 – 1.39 (m, 16H), 0.82 – 0.94 (m, 12H). ^{13}C NMR (75 MHz, THF-d_8):

δ 160.2 (C_{quat}), 145.8 (C_{quat}), 144.6 (C_{quat}), 142.2 (C_{quat}), 137.8 (CH), 136.3 (C_{quat}), 135.9 (C_{quat}), 133.6 (C_{quat}), 133.3 (C_{quat}), 128.1 (CH), 126.7 (CH), 126.4 (CH), 126.1 (CH), 126.0 (CH), 125.9 (C_{quat}), 125.8 (C_{quat}), 116.5 (CH), 115.0 (CH), 55.5 (CH_3), 48.1 (CH_2), 32.5 (CH_2), 27.8 (CH_2), 27.5 (CH_2), 23.6 (CH_2), 14.4 (CH_3); two CH are not visible due to signal overlap. ^{29}Si NMR (119 MHz, THF-d_8): δ 14.4. MS (MALDI-TOF) *m/z*: 1885.157 ($[M]^+$). IR: $\tilde{\nu}$ [cm^{-1}] = 2957 (m), 2913 (m), 1607 (w), 1464 (s), 1393 (m), 1335 (w), 1242 (s), 1179 (m), 1148 (w), 1109 (m), 1049 (m), 1028 (m), 880 (w), 831 (m), 802 (s), 719 (m), 712 (m), 654 (w). Anal. calcd. for $C_{124}H_{120}N_4O_4S_4Si$ [1886.7]: C 78.94, H 6.41, N 2.97, S 6.80; Found: C 79.16, H 6.24, N 2.86, S 6.84.

2.3.14 Tetrakis(4-(10-hexyl-7-(5-methylthiophen-2-yl)-10*H*-phenothiazin-3-yl)phenyl)silane (5m)

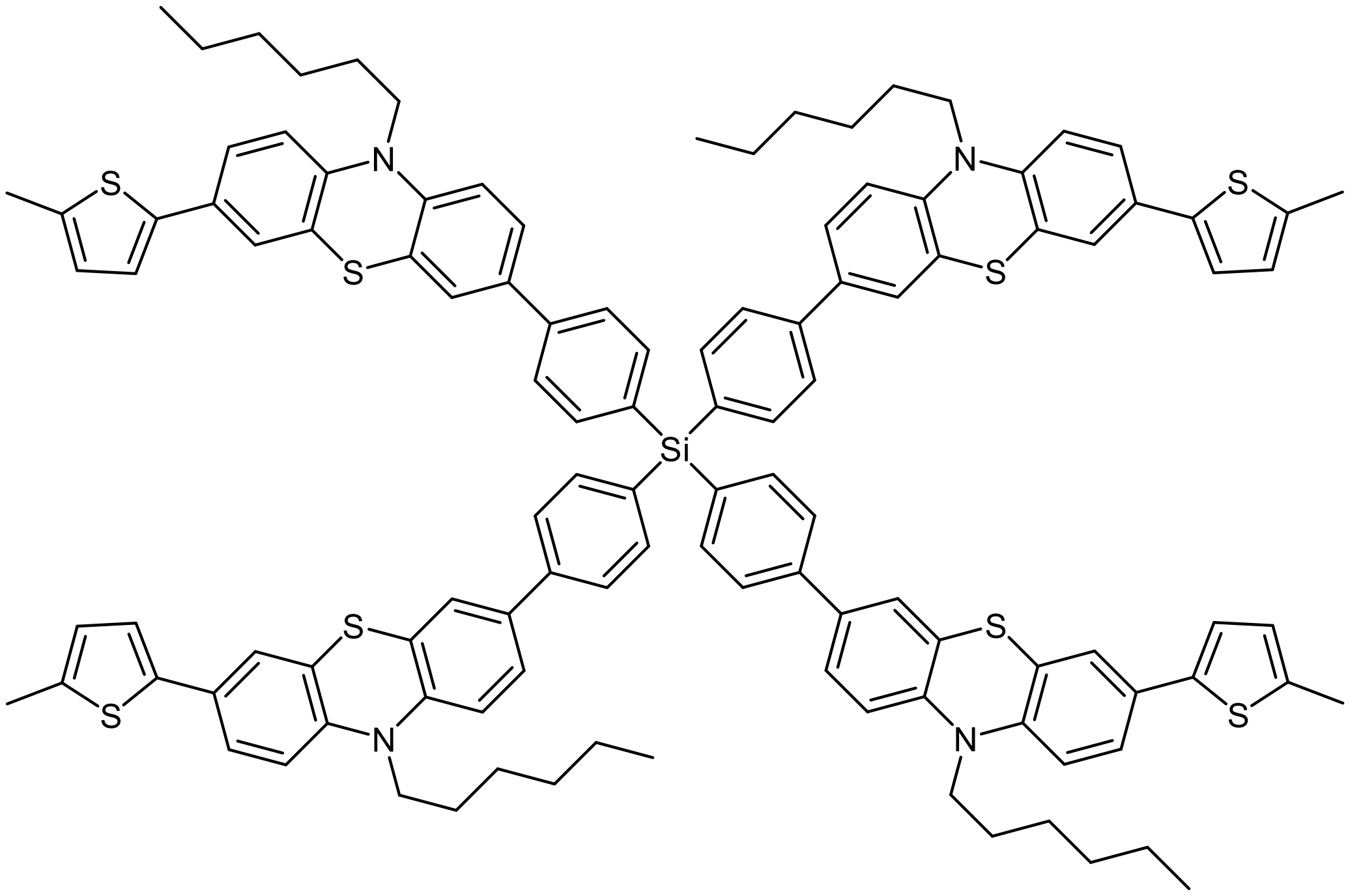

$C_{116}H_{112}N_4S_8Si$

[1846.77]

The crude product was synthesized according to **GPII** and purified by column chromatography on silica gel using gradient elution (petroleum ether/dichloromethane 5:1 → 1:1), followed by recrystallization from a mixture of *n*-pentane and dichloromethane. Compound **5m** was obtained as a pale-yellow solid in a yield of 399 mg (0.216 mmol, 72%), Mp 153 – 155 °C, R_f (petroleum ether/dichloromethane 2:1) = 0.43.

^{1}H NMR (600 MHz, THF-d_8): δ 7.67 – 7.71 (m, 8H), 7.61 – 7.64 (m, 8H), 7.41 – 7.48 (m, 8H), 7.29 – 7.34 (m, 8H), 7.04 (d, $^3J_{HH}$ = 3.5 Hz, 4H), 7.01 (d, $^3J_{HH}$ = 8.6 Hz, 4H), 6.89 – 6.94 (m,

4H), 6.65 – 6.69 (m, 4H), 3.92 (t, $^{3}J_{HH}$ = 7.1 Hz, 8H), 2.44 (s, 12H), 1.77 – 1.85 (m, 8H), 1.43 – 1.50 (m, 8H), 1.27 – 1.37 (m, 16H), 0.84 – 0.92 (m, 12H). ^{13}C NMR (150 MHz, THF-d$_8$): δ 145.7 (C_{quat}), 144.9 (C_{quat}), 142.3 (C_{quat}), 142.1 (C_{quat}), 139.4 (C_{quat}), 137.9 (CH), 136.1 (C_{quat}), 133.8 (C_{quat}), 130.6 (C_{quat}), 127.2 (CH), 126.9 (CH), 126.8 (CH), 126.5 (CH), 126.1 (C_{quat}), 125.8 (C_{quat}), 125.3 (CH), 124.7 (CH), 123.1 (CH), 116.7 (CH), 116.6 (CH), 48.3 (CH_2), 32.6 (CH_2), 27.9 (CH_2), 27.6 (CH_2), 23.7 (CH_2), 15.4 (CH_3), 14.5 (CH_3). MS (MALDI-TOF) *m/z*: 1844.880 ([M]$^+$). IR: $\tilde{\nu}$ [cm^{-1}] = 2953 (w), 2918 (w), 2853 (w), 1595 (w), 1499 (w), 1458 (s), 1402 (w), 1377 (w), 1358 (w), 1333 (w), 1300 (w), 1261 (m), 1248 (m), 1209 (w), 1192 (w), 1161 (w), 1107 (m), 1051 (w), 1016 (m), 878 (w), 793 (s), 721 (m), 710 (m), 691 (w), 675 (w), 656 (m). Anal. calcd. for $C_{116}H_{112}N_4S_8Si$ [1846.8]: C 75.44, H 6.11, N 3.03, S 13.89; Found: C 75.18, H 5.96, N 2.75, S 13.69.

2.3.15 Tetrakis(4-(10,10'-dihexyl-10*H*,10'*H*-[3,3'-biphenothiazin]-7-yl)phenyl)silane (5n)

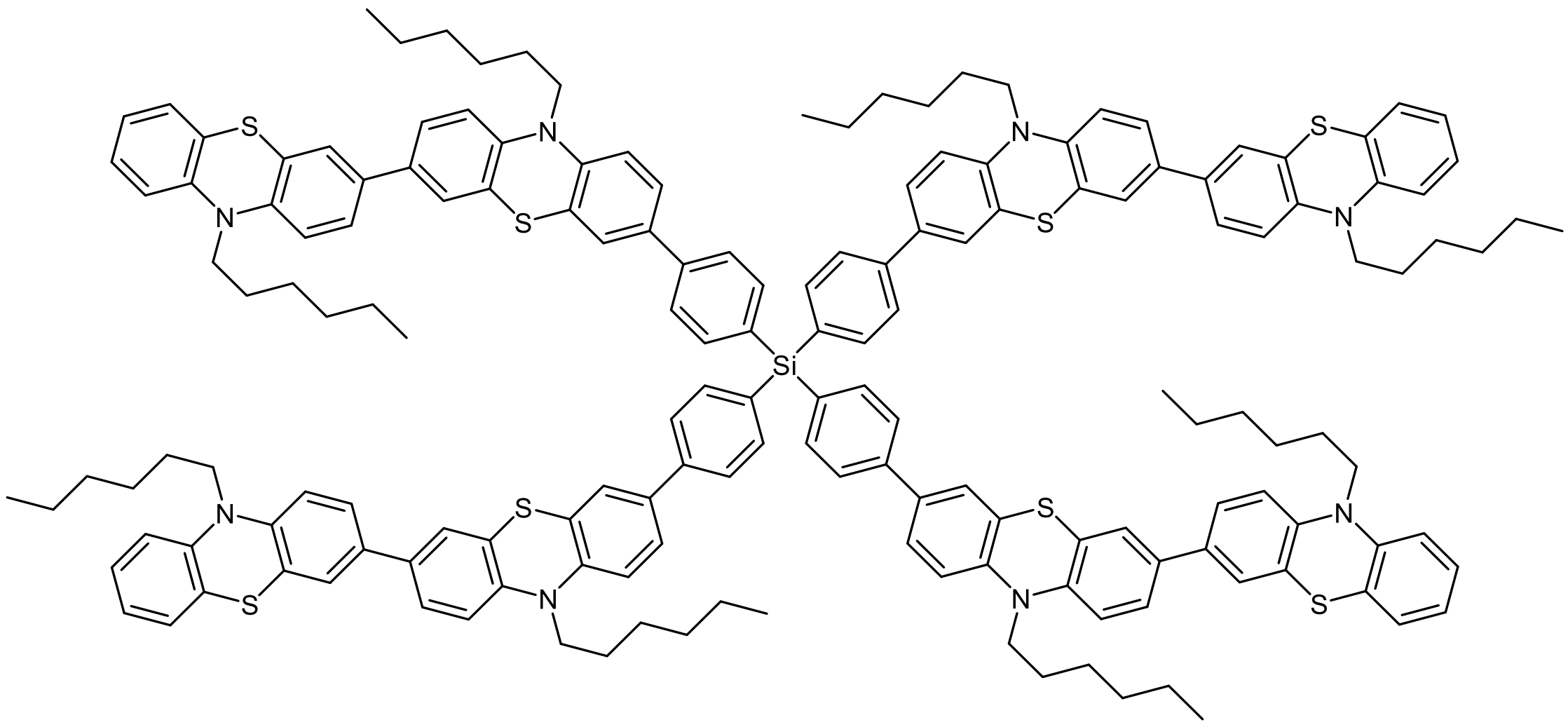

$C_{168}H_{172}N_8S_8Si$
[2587.85]

The crude product was synthesized according to **GPII** and purified by column chromatography on silica gel using gradient elution (petroleum ether/dichloromethane 5:1 → 2:1), followed by recrystallization from a mixture of *n*-pentane and dichloromethane. Compound **5n** was obtained as a pale-yellow solid in a yield of 279 mg (0.108 mmol, 54%), Mp 175 – 176 °C, R_f (petroleum ether/dichloromethane 2:1) = 0.47.

^{1}H NMR (600 MHz, THF-d$_8$): δ 7.67 – 7.71 (m, 8H), 7.61 – 7.65 (m, 8H), 7.43 – 7.48 (m, 8H), 7.31 – 7.39 (m, 16H), 7.11 (ddd, $^{3}J_{HH}$ = 8.1 Hz, $^{3}J_{HH}$ = 7.3 Hz, $^{4}J_{HH}$ = 1.6 Hz, 4H), 7.09 (dd, $^{3}J_{HH}$ = 7.6 Hz, $^{4}J_{HH}$ = 1.5 Hz, 4H), 7.01 (d, $^{3}J_{HH}$ = 8.6 Hz, 4H), 6.94 – 6.99 (m, 8H), 6.93 (dd, $^{3}J_{HH}$ = 8.2 Hz, $^{4}J_{HH}$ = 1.1 Hz, 4H), 6.84 – 6.88 (m, 4H), 3.85 – 3.98 (m, 16H), 1.75 – 1.87 (m,

16H), 1.42 – 1.52 (m, 16H), 1.25 – 1.37 (m, 32H), 0.83 – 0.92 (m, 24H). ^{13}C NMR (150 MHz, THF-d$_8$): δ 146.4 (C$_{quat}$), 145.8 (C$_{quat}$), 145.4 (C$_{quat}$), 145.0 (C$_{quat}$), 142.3 (C$_{quat}$), 137.9 (CH), 136.1 (C$_{quat}$), 135.4 (C$_{quat}$), 135.3 (C$_{quat}$), 133.8 (C$_{quat}$), 128.2 (CH), 128.1 (CH), 126.9 (CH), 126.8 (CH), 126.5 (CH), 126.1 (CH), 126.0 (CH), 126.0 (C$_{quat}$), 125.8 (CH), 125.8 (CH), 123.2 (CH), 116.6 (CH), 116.5 (CH), 48.2 (CH$_2$), 48.1 (CH$_2$), 32.6 (CH$_2$), 32.6 (CH$_2$), 28.0 (CH$_2$), 27.9 (CH$_2$), 27.6 (CH$_2$), 27.6 (CH$_2$), 23.7 (CH$_2$), 23.7 (CH$_2$), 14.5 (CH$_3$), 14.5 (CH$_3$); three C$_{quat}$ and two CH are not visible due to signal overlap. ^{29}Si NMR (119 MHz, THF-d$_8$): δ 14.3. MS (MALDI-TOF) *m*/*z*: 2586.462 ([M]$^+$). IR: $\tilde{\nu}$ [cm^{-1}] = 2953 (w), 2926 (w), 2853 (w), 1597 (w), 1576 (w), 1456 (s), 1408 (w), 1377 (w), 1331 (w), 1240 (m), 1192 (w), 1134 (w), 1107 (m), 1057 (w), 1040 (w), 1016 (w), 872 (w), 802 (m), 745 (m), 721 (m), 710 (m), 658 (w), 617 (w). Anal. calcd. for $C_{168}H_{172}N_8S_8Si$ [2587.9]: C 77.97, H 6.70, N 4.33, S 9.91; Found: C 78.25, H 6.45, N 3.98, S 9.86.

2.3.16 Tetrakis(4-(10-hexyl-2-methyl-10*H*-phenothiazin-3-yl)phenyl)silane (5o)

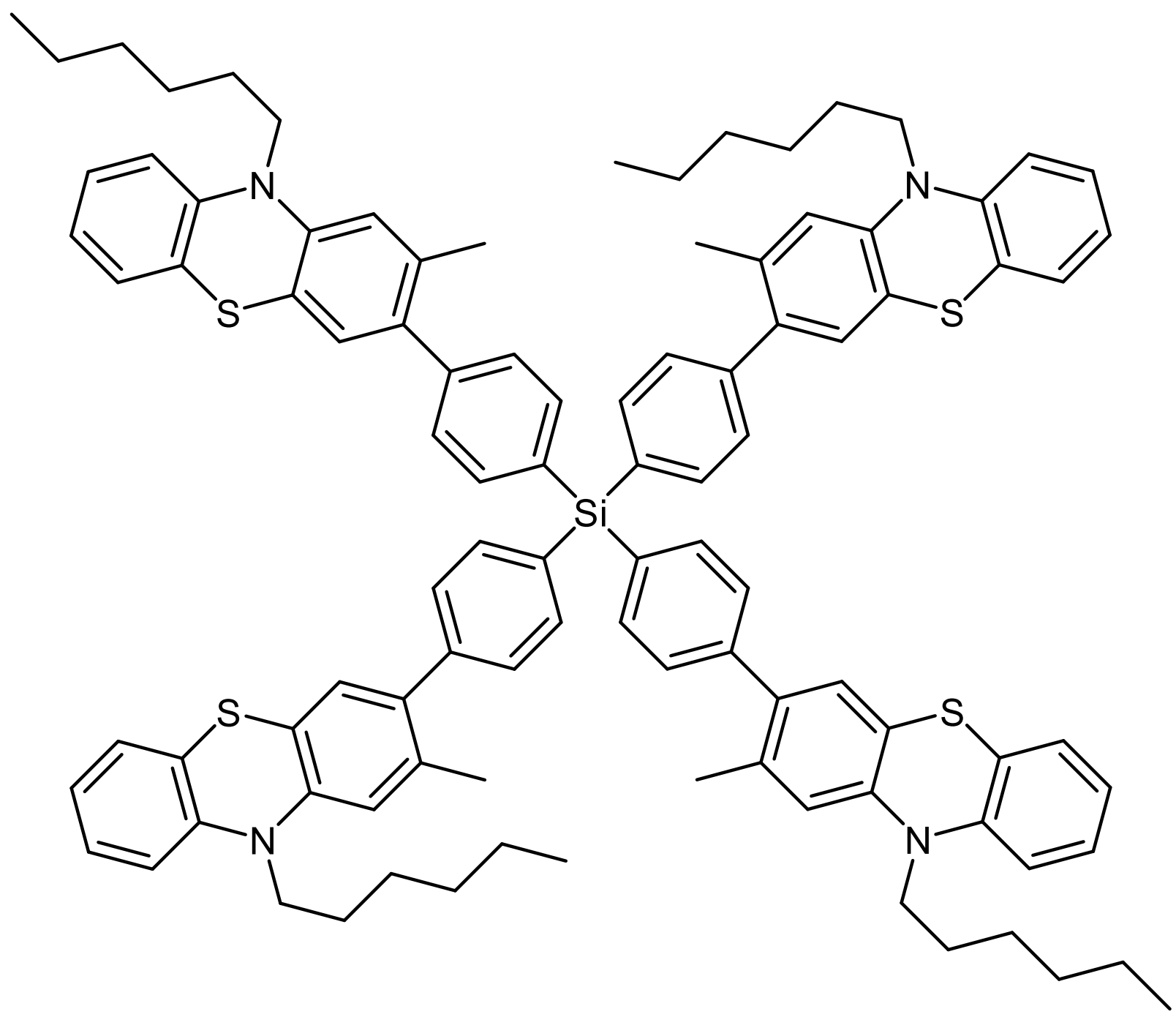

$C_{100}H_{104}N_4S_4Si$
[1518.29]

The crude product was synthesized according to **GPII** and purified by column chromatography on silica gel (petroleum ether/dichloromethane 2:1), followed by recrystallization from a mixture of *n*-pentane and dichloromethane. Compound **5o** was obtained as a pale-yellow solid in a yield of 343 mg (0.226 mmol, 75%), Mp 156 – 157 °C, R$_f$ (petroleum ether/dichloromethane 5:1) = 0.18.

^{1}H NMR (300 MHz, THF-d$_8$): δ 7.69 – 7.73 (m, 8H), 7.36 – 7.38 (m, 8H), 7.07 – 7.14 (m, 8H), 7.02 (s, 4H), 6.94 (dd, $^3J_{HH}$ = 8.3 Hz, $^4J_{HH}$ = 1.2 Hz, 4H), 6.84 – 6.87 (m, 8H), 3.93 (t, $^3J_{HH}$ = 7.0 Hz, 8H), 2.27 (s, 12H), 1.77 – 1.86 (m, 8H), 1.43 – 1.52 (m, 8H), 1.27 – 1.38 (m, 16H), 0.84 – 0.92 (m, 12H). ^{13}C NMR (75 MHz, THF-d$_8$): δ 146.2 (C$_{quat}$), 145.7 (C$_{quat}$), 143.5 (C$_{quat}$), 137.2 (CH), 136.9 (C$_{quat}$), 135.1 (C$_{quat}$), 133.5 (C$_{quat}$), 129.7 (CH), 129.1 (CH), 128.1 (CH), 127.9 (CH), 126.1 (C$_{quat}$), 123.0 (C$_{quat}$), 123.0 (CH), 118.2 (CH), 116.4 (CH), 48.0 (CH$_2$), 32.5 (CH$_2$), 27.9 (CH$_2$), 27.5 (CH$_2$), 23.6 (CH$_2$), 20.8 (CH$_3$), 14.4 (CH$_3$). MS (MALDI-TOF) *m/z*: 1516.775 ([M]$^+$). IR: $\tilde{\nu}$ [cm^{-1}] = 3057 (w), 3013 (w), 2953 (w), 2924 (w), 2855 (w), 1597 (w), 1574 (w), 1504 (w), 1487 (w), 1460 (s), 1443 (m), 1400 (m), 1377 (m), 1325 (m), 1294 (m), 1254 (m), 1236 (m), 1192 (m), 1134 (w), 1103 (m), 1040 (w), 1018 (w), 926 (w), 887 (w), 849 (w), 827 (m), 795 (w), 746 (s), 712 (m), 687 (s), 656 (m), 602 (m). Anal. calcd. for $C_{100}H_{104}N_4S_4Si$ [1518.3]: C 79.11, H 6.90, N 3.69, S 8.45; Found: C 79.15, H 7.02, N 3.51, S 8.47.

2.3.17 Tetrakis(4-(10-hexyl-2-methoxy-10*H*-phenothiazin-3-yl)phenyl)silane (5p)

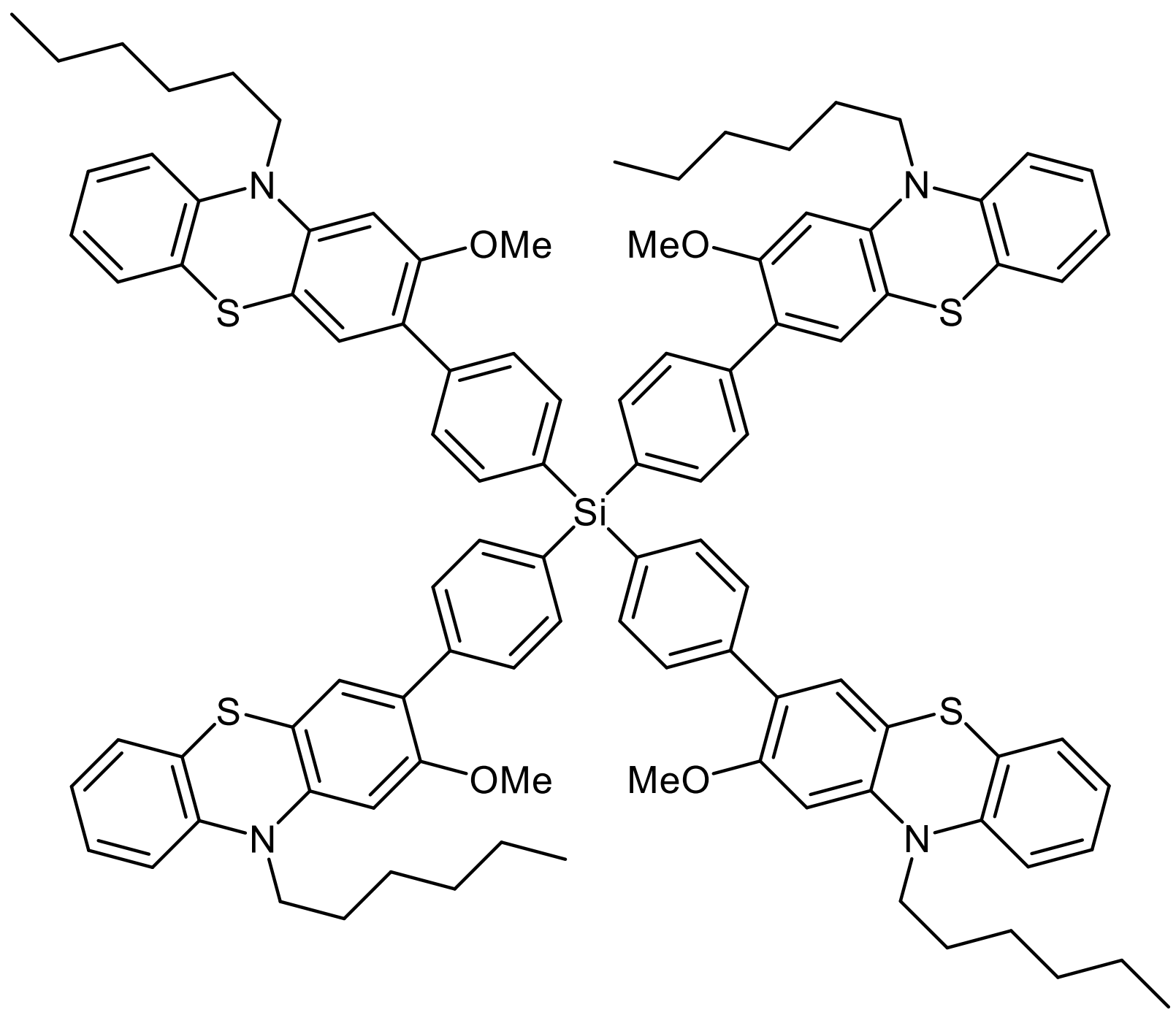

$C_{100}H_{104}N_4O_4S_4Si$

[1582.28]

The crude product was synthesized according to **GPII** and purified by column chromatography on silica gel (petroleum ether/dichloromethane 2:1), followed by recrystallization from a mixture of *n*-pentane and dichloromethane. Compound **5p** was obtained as a pale-yellow solid in a yield of 335 mg (0.212 mmol, 68%), Mp 126 – 128 °C, R$_f$ (petroleum ether/dichloromethane 5:1) = 0.28.

^{1}H NMR (600 MHz, THF-d$_8$): δ 7.64 (d, $^{3}J_{HH}$ = 7.8 Hz, 8H), 7.53 (d, $^{3}J_{HH}$ = 7.5 Hz, 8H), 7.07 – 7.14 (m, 12H), 6.95 (d, $^{3}J_{HH}$ = 8.1 Hz, 4H), 6.87 (t, $^{3}J_{HH}$ = 7.5 Hz, 4H), 6.66 (s, 4H), 3.96 (t, $^{3}J_{HH}$ = 7.0 Hz, 8H), 3.79 (s, 12H), 1.84 (pent, $^{3}J_{HH}$ = 7.3 Hz, 8H), 1.49 (pent, $^{3}J_{HH}$ = 7.2 Hz, 8H), 1.27 – 1.38 (m, 16H), 0.85 – 0.93 (m, 12H). ^{13}C NMR (150 MHz, THF-d$_8$): δ 157.8 (C$_{quat}$), 147.3 (C$_{quat}$), 146.3 (C$_{quat}$), 140.3 (C$_{quat}$), 137.0 (CH), 133.5 (C$_{quat}$), 129.6 (CH), 128.2 (CH), 127.9 (CH), 126.8 (CH), 126.8 (C$_{quat}$), 125.8 (C$_{quat}$), 123.3 (CH), 116.8 (C$_{quat}$), 116.6 (CH), 101.6 (CH), 56.2 (CH_3), 48.3 (CH_2), 32.6 (CH_2), 28.2 (CH_2), 27.7 (CH_2), 23.7 (CH_2), 14.5 (CH_3). MS (MALDI-TOF) *m*/*z*: 1580.706 ([M]$^+$). IR: $\tilde{\nu}$ [cm^{-1}] = 2988 (m), 2959 (m), 2924 (m), 2901 (m), 1599 (w), 1574 (w), 1443 (m), 1404 (m), 1383 (m), 1329 (w), 1273 (m), 1254 (m), 1234 (m), 1206 (m), 1192 (m), 1177 (m), 1134 (w), 1103 (m), 1055 (s), 1038 (s), 1018 (m), 968 (w), 891 (w), 827 (m), 745 (m), 692 (m), 669 (m), 611 (m). Anal. calcd. for $C_{100}H_{104}N_4O_4S_4Si$ [1582.3]: C 75.91, H 6.63, N 3.54, S 8.10; Found: C 76.21, H 6.57, N 3.27, S 8.08.

2.3.18 Tetrakis(4-(10-hexyl-2-(methylthio)-10*H*-phenothiazin-3-yl)phenyl)silane (5q)

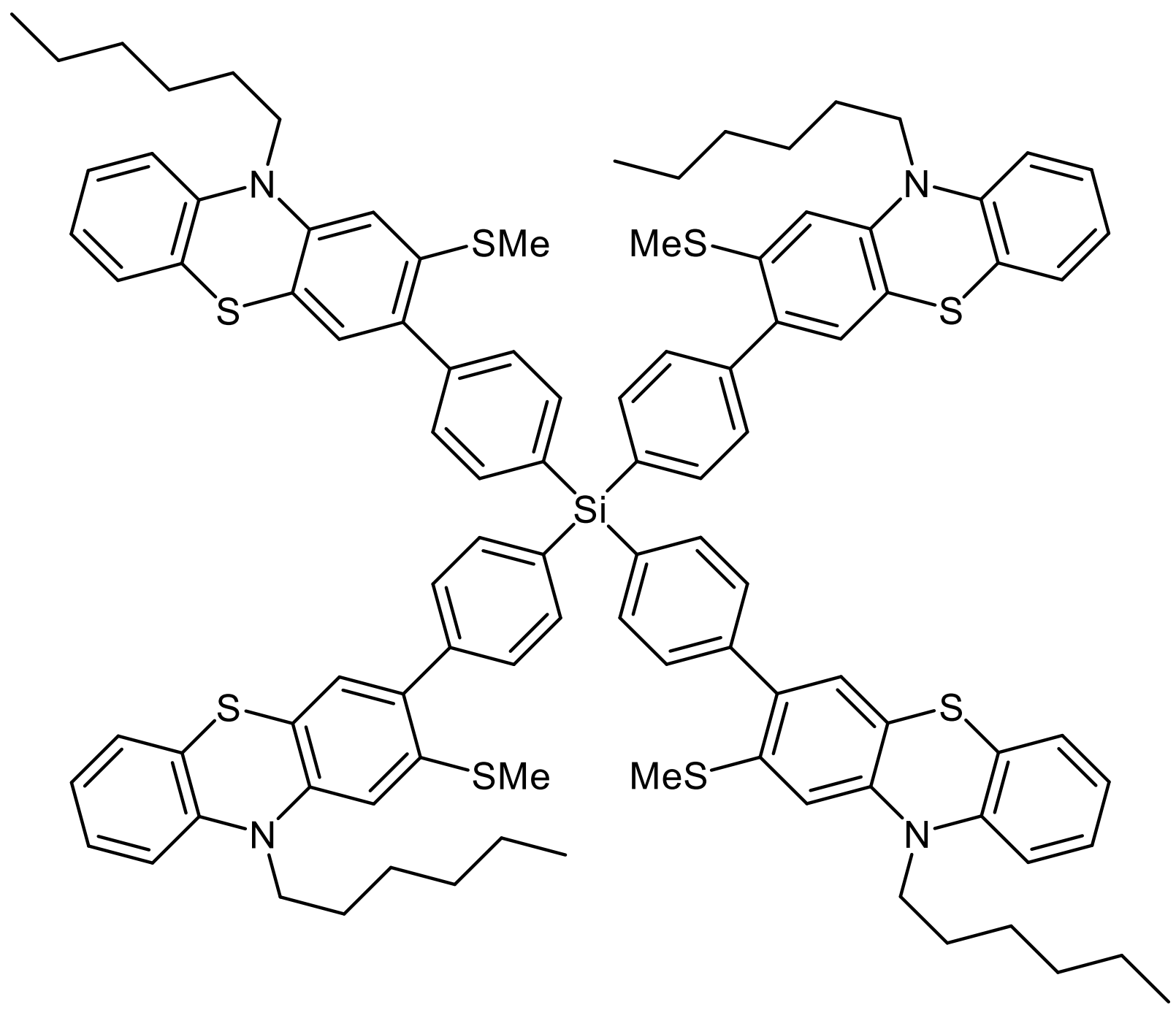

$C_{100}H_{104}N_4S_8Si$

[1646.53]

The crude product was synthesized according to **GPII** and purified by column chromatography on silica gel (petroleum ether/dichloromethane 2:1), followed by recrystallization from a mixture of *n*-pentane and dichloromethane. Compound **5q** was obtained as a pale-yellow solid in a yield of 314 mg (0.191 mmol, 64%), Mp 106 – 108 °C, R$_f$ (petroleum ether/dichloromethane 5:1) = 0.23.

^{1}H NMR (600 MHz, THF-d$_8$): δ 7.70 (d, $^3J_{HH}$ = 8.0 Hz, 8H), 7.46 (d, $^3J_{HH}$ = 8.0 Hz, 8H), 7.11 – 7.16 (m, 4H), 7.09 (dd, $^3J_{HH}$ = 7.7 Hz, $^4J_{HH}$ = 1.5 Hz, 4H), 7.03 (s, 4H), 6.94 – 6.98 (m, 4H), 6.85 – 6.91 (m, 8H), 3.96 (t, $^3J_{HH}$ = 7.1 Hz, 8H), 2.34 (s, 12H), 1.84 (pent, $^3J_{HH}$ = 7.6 Hz, 8H), 1.45 – 1.53 (m, 8H), 1.26 – 1.38 (m, 16H), 0.83 – 0.92 (m, 12H). ^{13}C NMR (150 MHz, THF-d$_8$): δ 146.3 (C$_{quat}$), 146.2 (C$_{quat}$), 142.4 (C$_{quat}$), 137.2 (C$_{quat}$), 137.1 (CH), 136.4 (C$_{quat}$), 134.1 (C$_{quat}$), 129.9 (CH), 129.4 (CH), 128.2 (CH), 128.1 (CH), 126.0 (C$_{quat}$), 123.4 (CH), 122.8 (C$_{quat}$), 116.7 (CH), 114.9 (CH), 48.3 (CH$_2$), 32.7 (CH$_2$), 28.0 (CH$_2$), 27.7 (CH$_2$), 23.7 (CH$_2$), 16.7 (CH$_3$), 14.5 (CH$_3$). MS (MALDI-TOF) *m/z*: 1644.618 ([M]$^+$). IR: $\tilde{\nu}$ [cm^{-1}] = 2953 (w), 2918 (w), 2870 (w), 1595 (w), 1570 (w), 1506 (w), 1458 (s), 1381 (w), 1323 (w), 1292 (w), 1250 (m), 1192 (w), 1134 (w), 1111 (m), 1086 (m), 1051 (w), 1038 (m), 1018 (w), 935 (w), 889 (w), 827 (m), 745 (s), 681 (m), 667 (w), 640 (m). Anal. calcd. for $C_{100}H_{104}N_4S_8Si$ [1646.5]: C 72.95, H 6.37, N 3.40, S 15.58; Found: C 73.25, H 6.42, N 3.33, S 15.46.

2.3.19 Tetrakis(4-(10-hexyl-10*H*-phenothiazin-1-yl)phenyl)silane (5r)

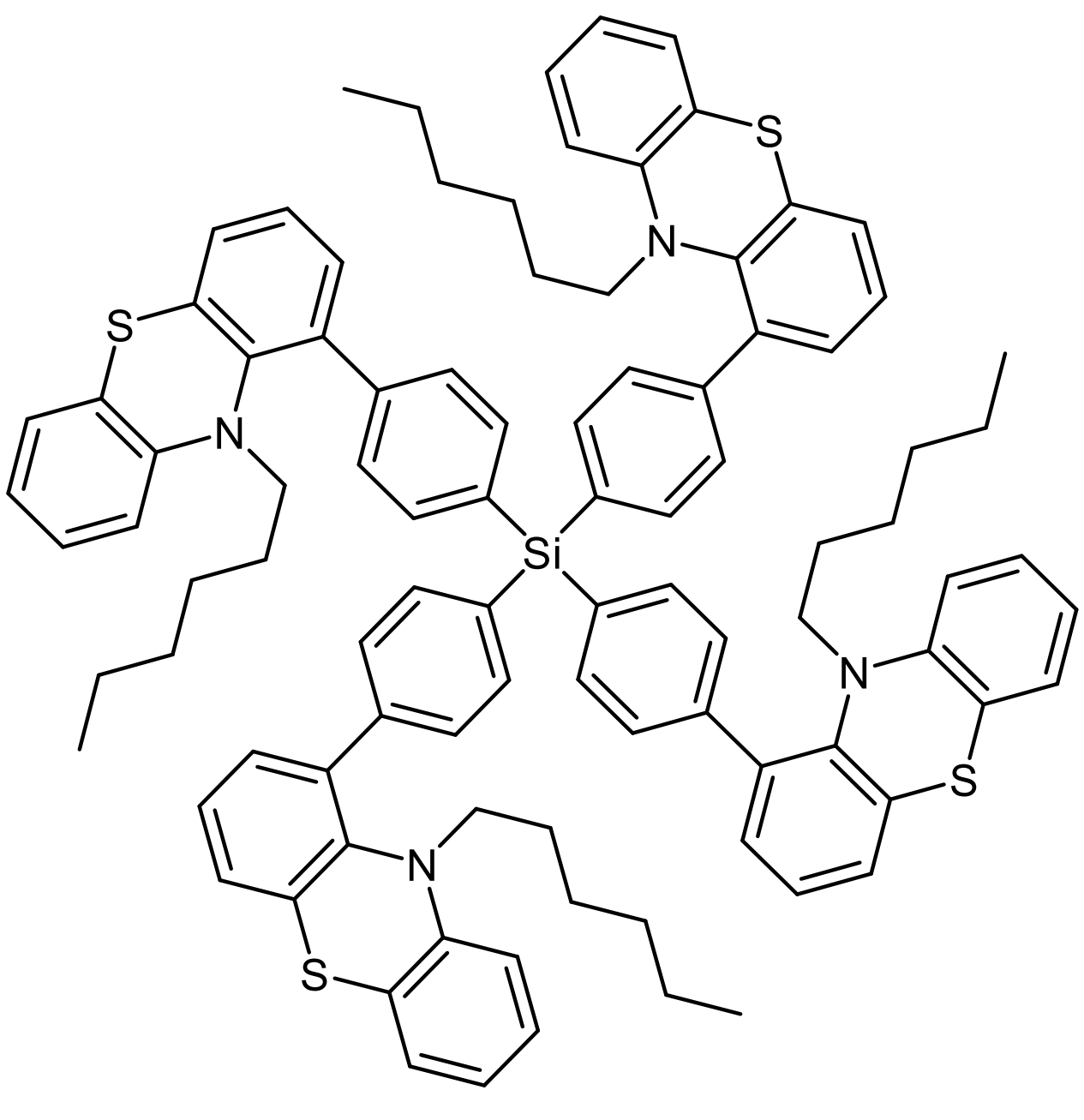

$C_{96}H_{96}N_4S_4Si$

[1462.18]

The crude product was synthesized according to **GPII** and purified by column chromatography on silica gel (petroleum ether/dichloromethane 10:1), followed by recrystallization from a mixture of *n*-pentane and dichloromethane. Compound **5r** was obtained as a pale yellow solid in a yield of 275 mg (0.188 mmol, 75%), Mp 120 – 121 °C, R$_f$ (petroleum ether/dichloromethane 5:1) = 0.20.

^{1}H NMR (300 MHz, THF-d_8): δ 7.87 (d, $^{3}J_{HH}$ = 7.9 Hz, 8H), 7.55 (d, $^{3}J_{HH}$ = 7.9 Hz, 8H), 7.02 – 7.29 (m, 20H), 6.88 – 7.02 (m, 8H), 3.17 – 3.31 (m, 8H), 1.34 – 1.49 (m, 8H), 1.18 – 1.29 (m, 8H), 0.98 – 1.16 (m, 16H), 0.74 (t, $^{3}J_{HH}$ = 6.9 Hz, 12H). ^{13}C NMR (75 MHz, THF-d_8): δ 148.1 (C_{quat}), 143.7 (C_{quat}), 143.7 (C_{quat}), 137.6 (CH), 134.6 (C_{quat}), 134.0 (C_{quat}), 133.9 (C_{quat}), 131.1 (CH), 130.4 (C_{quat}), 129.1 (CH), 128.1 (CH), 127.9 (CH), 127.3 (CH), 124.5 (CH), 123.9 (CH), 119.3 (CH), 54.1 (CH_2), 32.3 (CH_2), 29.6 (CH_2), 27.1 (CH_2), 23.4 (CH_2), 14.4 (CH_3). MS (MALDI-TOF) *m*/*z*: 1460.756 ($[M]^+$). IR: $\tilde{\nu}$ [cm^{-1}] = 3059 (w), 3013 (w), 2953 (w), 2926 (w), 2855 (w), 1595 (w), 1568 (w), 1537 (w), 1476 (m), 1466 (m), 1445 (m), 1427 (s), 1385 (m), 1331 (w), 1283 (w), 1261 (w), 1240 (w), 1223 (w), 1196 (w), 1148 (w), 1130 (w), 1111 (m), 1074 (w), 1040 (w), 1016 (w), 955 (w), 928 (w), 907 (w), 833 (w), 814 (w), 777 (m), 746 (s), 725 (m), 704 (s), 683 (m), 610 (w). Anal. calcd. for $C_{96}H_{96}N_4S_4Si$ [1462.2]: C 78.86, H 6.62, N 3.83, S 8.77; Found: C 79.08, H 6.95, N 3.60, S 8.41.

2.3.20 Tetrakis(10-hexyl-10*H*-phenothiazin-3-yl)silane (6)

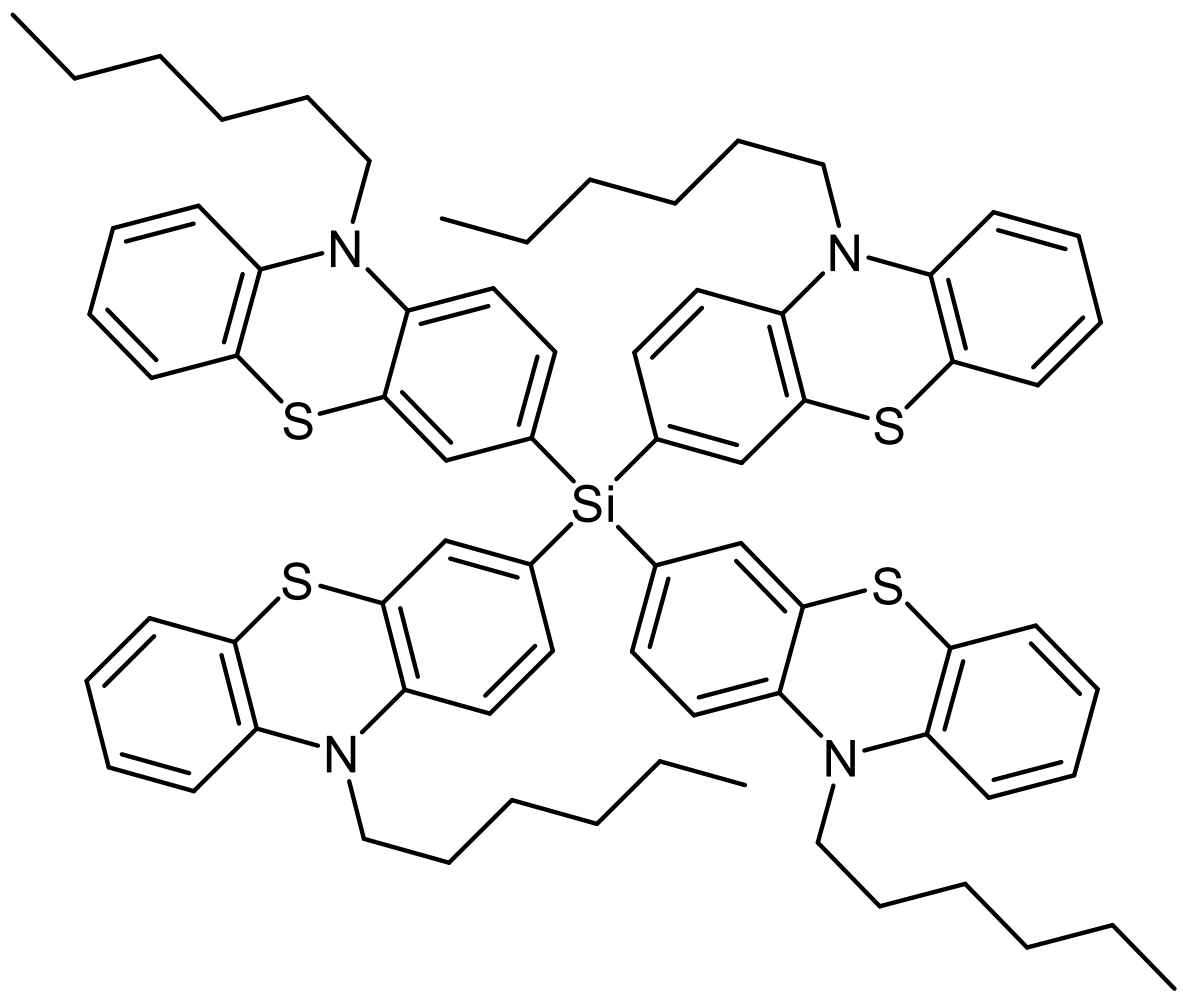

$C_{72}H_{80}N_4S_4Si$

[1157.78]

In a dry Schlenk tube under a nitrogen atmosphere, 870 mg (2.40 mmol, 4.80 equiv.) of 3-bromo-10-hexyl-10*H*-phenothiazine (**4c**) was dissolved in anhydrous 1,4-dioxane (12.0 mL, 5.00 mL/mmol). Subsequently, *n*-butyllithium (1.38 mL, 2.20 mmol, 4.40 equiv., 1.6 M in *n*-hexane) was added dropwise under vigorous stirring at 20 °C. The reaction mixture was stirred for an additional 30 min, after which silicon tetrachloride (0.057 mL, 0.500 mmol, 1.00 equiv.) was added, and the mixture was heated to 100 °C and stirred for 18 h. Upon completion of the reaction, the solvent was removed under reduced pressure, and the crude product was adsorbed onto Celite®. Purification by flash column chromatography on silica gel using gradient elution (petroleum ether/dichloromethane, 100:1 → 5:1), afforded compound **6** as a pale-

yellow solid (86.8 mg, 0.0750 mmol, 15%), Mp 102 – 104 °C, R_f (petroleum ether/dichloromethane 5:1) = 0.22.

^{1}H NMR (600 MHz, THF-d$_8$): δ 7.26 (dd, $^3J_{HH}$ = 8.1 Hz, $^4J_{HH}$ = 1.6 Hz, 4H), 7.20 (d, $^4J_{HH}$ = 1.5 Hz, 4H), 7.07 – 7.13 (m, 4H), 7.02 (dd, $^3J_{HH}$ = 7.6 Hz, $^4J_{HH}$ = 1.5 Hz, 4H), 6.93 (t, $^3J_{HH}$ = 8.3 Hz, 8H), 6.85 (td, $^3J_{HH}$ = 7.4 Hz, $^4J_{HH}$ = 1.1 Hz, 4H), 3.88 (t, $^3J_{HH}$ = 7.2 Hz, 8H), 1.75 – 1.83 (m, 8H), 1.40 – 1.49 (m, 8H), 1.26 – 1.36 (m, 16H), 0.83 – 0.92 (m, 12H). ^{13}C NMR (150 MHz, THF-d$_8$): δ 147.9 (C$_{quat}$), 146.0 (C$_{quat}$), 136.6 (CH), 135.4 (CH), 128.4 (C$_{quat}$), 128.2 (CH), 128.1 (CH), 125.8 (C$_{quat}$), 125.4 (C$_{quat}$), 123.4 (CH), 116.5 (CH), 116.0 (CH), 48.2 (CH$_2$), 32.6 (CH$_2$), 27.9 (CH$_2$), 27.6 (CH$_2$), 23.7 (CH$_2$), 14.5 (CH$_3$). ^{29}Si NMR (119 MHz, THF-d$_8$): δ 15.2. MS (MALDI-TOF) *m*/*z*: 1156.478 ([M]$^+$). IR: $\tilde{\nu}$ [cm^{-1}] = 2953 (w), 2928 (w), 2847 (w), 1591 (w), 1568 (m), 1487 (w), 1452 (s), 1371 (w), 1337 (m), 1315 (w), 1298 (w), 1281 (m), 1261 (m), 1248 (s), 1236 (m), 1221 (m), 1198 (m), 1171 (m), 1155 (m), 1125 (s), 1103 (s), 1038 (m), 901 (w), 870 (w), 854 (w), 808 (m), 799 (m), 741 (s), 685 (m), 656 (m), 604 (m). Anal. calcd. for $C_{72}H_{80}N_4S_4Si$ [1157.8]: C 74.69, H 6.97, N 4.84, S 11.08; Found: C 74.32, H 6.95, N 4.44, S 11.35.

2.3.21 Tetrakis(5-(10-hexyl-10*H*-phenothiazin-3-yl)thiophen-2-yl)silane (7)

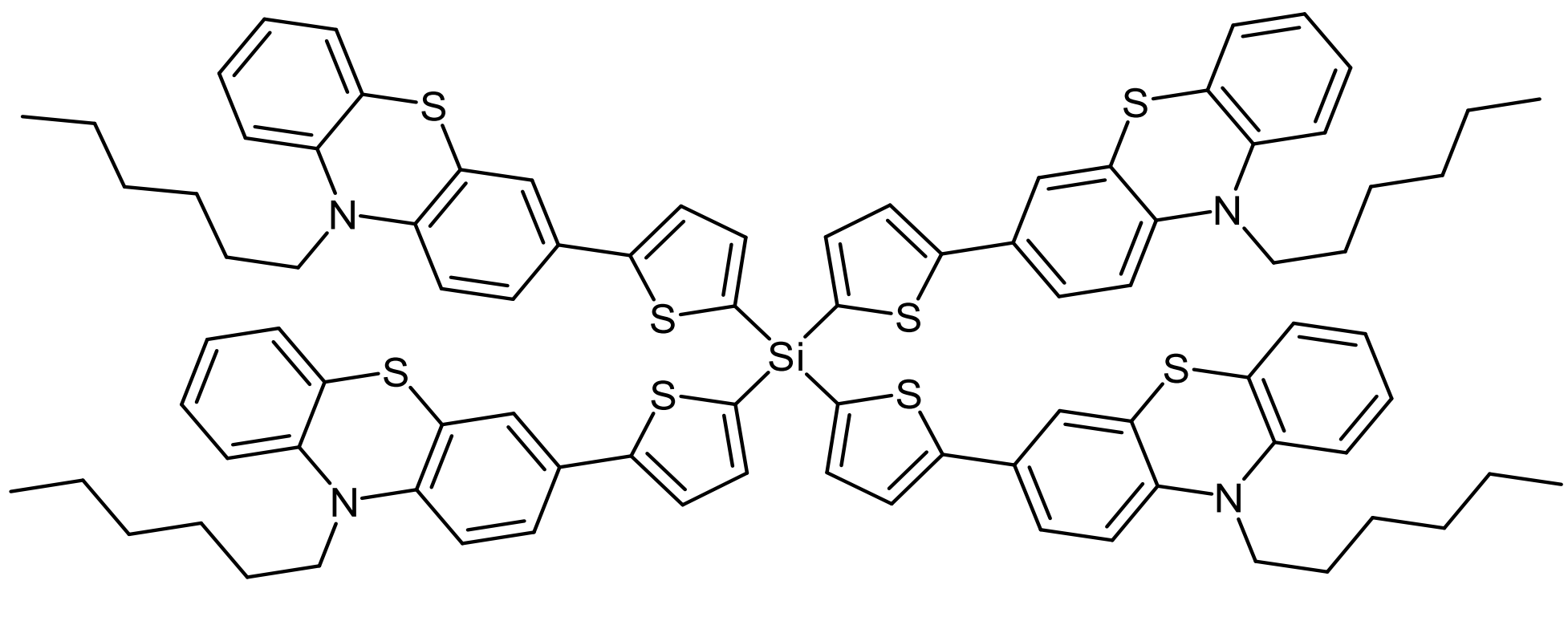

$C_{88}H_{88}N_4S_8Si$
[1486.27]

In a dry Schlenk tube under a nitrogen atmosphere, 439 mg (1.20 mmol, 4.80 equiv.) of 10-hexyl-3-(thiophen-2-yl)-10*H*-phenothiazine (**4s**) was dissolved in anhydrous 1,4-dioxane (6.00 mL, 5.00 mL/mmol). Subsequently, *n*-butyllithium (0.69 mL, 1.10 mmol, 4.40 equiv., 1.6 M in *n*-hexane) was added dropwise under vigorous stirring at 20 °C. The reaction mixture was stirred for an additional 30 min, after which silicon tetrachloride (0.029 mL, 0.250 mmol, 1.00 equiv.) was added, and the mixture was heated to 100 °C and stirred for 18 h. Upon completion of the reaction, the solvent was removed under reduced pressure, and the crude product was adsorbed onto Celite®. Purification by flash column chromatography on silica gel using gradient elution (petroleum ether/dichloromethane, 6:1 → 2:1), followed by recrystallization in a mixture

of *n*-pentane and dichloromethane, afforded compound **7** as a pale-yellow solid (114 mg, 0.0767 mmol, 31%), Mp 166 – 168 °C, R_f (petroleum ether/dichloromethane 5:1) = 0.17.

^{1}H NMR (600 MHz, THF-d$_8$): δ 7.38 – 7.55 (m, 16H), 7.09 – 7.13 (m, 4H), 7.07 (dd, $^3J_{HH}$ = 7.6 Hz, $^4J_{HH}$ = 1.5 Hz, 4H), 6.89 – 6.95 (m, 8H), 6.87 (t, $^3J_{HH}$ = 7.2 Hz, 4H), 3.88 (t, $^3J_{HH}$ = 7.1 Hz, 8H), 1.78 (pent, $^3J_{HH}$ = 7.5 Hz, 8H), 1.44 (pent, $^3J_{HH}$ = 7.6 Hz, 8H), 1.23 – 1.34 (m, 16H), 0.81 – 0.93 (m, 12H). ^{13}C NMR (150 MHz, THF-d$_8$): δ 152.8 (C$_{quat}$), 146.2 (C$_{quat}$), 146.1 (C$_{quat}$), 140.6 (CH), 132.8 (C$_{quat}$), 129.6 (C$_{quat}$), 128.2 (CH), 128.2 (CH), 126.7 (C$_{quat}$), 126.1 (CH), 125.4 (CH), 125.4 (C$_{quat}$), 124.9 (CH), 123.4 (CH), 116.6 (CH), 116.6 (CH), 48.2 (CH$_2$), 32.6 (CH$_2$), 27.9 (CH$_2$), 27.5 (CH$_2$), 23.7 (CH$_2$), 14.5 (CH$_3$). MS (MALDI-TOF) *m/z*: 1484.746 ([M]$^+$). IR: $\tilde{\nu}$ [cm^{-1}] = 2988 (s), 2943 (m), 2901 (s), 1485 (w), 1460 (m), 1443 (w), 1425 (m), 1406 (m), 1381 (m), 1335 (w), 1231 (m), 1103 (m), 1057 (s), 1028 (m), 1011 (m), 970 (w), 922 (w), 891 (w), 872 (m), 800 (m), 745 (m), 698 (m). Anal. calcd. for $C_{88}H_{88}N_4S_8Si$ [1486.3]: C 71.12, H 5.97, N 3.77, S 17.26; Found: C 71.16, H 6.34, N 3.54, S 17.22.

2.3.22 Tetrakis(4-(5-(10-hexyl-10*H*-phenothiazin-3-yl)thiophen-2-yl)phenyl)silane (8)

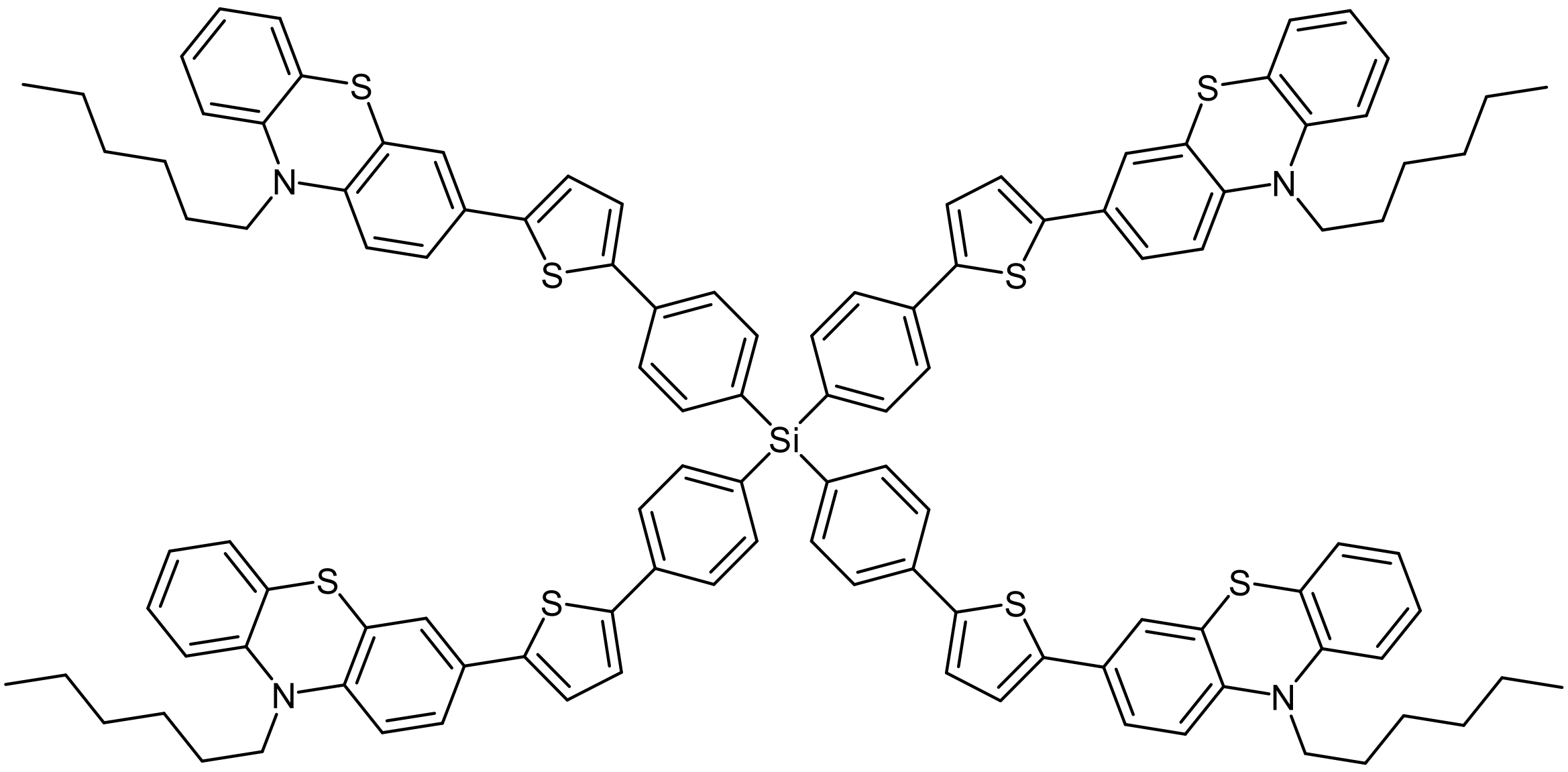

$C_{112}H_{104}N_4S_8Si$

[1790.66]

In a dry Schlenk tube under a nitrogen atmosphere, 384 mg (1.05 mmol, 4.20 equiv.) of 10-hexyl-3-(thiophen-2-yl)-10*H*-phenothiazine (**4s**) was dissolved in anhydrous THF (7.50 mL) and cooled to –78 °C. *n*-Butyllithium (0.72 mL, 1.15 mmol, 4.60 equiv., 1.6 M in *n*-hexane) was added dropwise, and the reaction mixture was stirred for 30 min at –78 °C. Trimethyl borate (0.145 mL, 1.30 mmol, 5.20 equiv.) was then added. The cooling bath was removed, and the reaction was stirred at 20 °C for 1 h. Subsequently, tetrakis(4-bromophenyl)silane (**1**, 163 mg,

0.250 mmol, 1.00 equiv.), tetrakis(triphenylphosphane)palladium(0) (58 mg, 0.050 mmol, 20 mol%), sodium hydroxide (52 mg, 1.30 mmol, 5.20 equiv.), and distilled water (2.50 mL) were added. The mixture was then heated at 100 °C and stirred for 18 h. Upon completion, the organic phase was separated, and the aqueous phase was extracted three times with dichloromethane. The combined organic phases were concentrated. The crude product was adsorbed onto Celite®. Purification by flash column chromatography on silica gel using gradient elution (petroleum ether/dichloromethane, 6:1 → 1:1), followed by recrystallization from a mixture of *n*-pentane and dichloromethane, afforded compound **8** as a pale-yellow solid (274 mg, 0.153 mmol, 61%), Mp 116 – 118 °C, R_f (petroleum ether/dichloromethane 2:1) = 0.42.

^{1}H NMR (600 MHz, THF-d_8): δ 7.67 – 7.73 (m, 8H), 7.61 – 7.67 (m, 8H), 7.40 – 7.46 (m, 12H), 7.29 (d, $^3J_{HH}$ = 3.9 Hz, 4H), 7.12 (ddd, $^3J_{HH}$ = 8.5 Hz, $^3J_{HH}$ = 7.4 Hz, $^4J_{HH}$ = 1.6 Hz, 4H), 7.09 (dd, $^3J_{HH}$ = 7.6 Hz, $^4J_{HH}$ = 1.5 Hz, 4H), 6.91 – 6.96 (m, 8H), 6.88 (td, $^3J_{HH}$ = 7.5 Hz, $^4J_{HH}$ = 1.1 Hz, 4H), 3.90 (t, $^3J_{HH}$ = 7.1 Hz, 8H), 1.79 (pent, $^3J_{HH}$ = 7.6 Hz, 8H), 1.45 (pent, $^3J_{HH}$ = 7.2 Hz, 8H), 1.25 – 1.35 (m, 16H), 0.83 – 0.91 (m, 12H). ^{13}C NMR (150 MHz, THF-d_8): δ 146.1 (C_{quat}), 145.9 (C_{quat}), 144.3 (C_{quat}), 143.3 (C_{quat}), 138.0 (CH), 136.8 (C_{quat}), 134.1 (C_{quat}), 129.8 (C_{quat}), 128.3 (CH), 128.2 (CH), 126.7 (C_{quat}), 125.8 (CH), 125.7 (CH), 125.5 (CH), 125.4 (C_{quat}), 124.9 (CH), 124.5 (CH), 123.4 (CH), 116.7 (CH), 116.6 (CH), 48.2 (CH_2), 32.6 (CH_2), 27.9 (CH_2), 27.6 (CH_2), 23.7 (CH_2), 14.5 (CH_3). MS (MALDI-TOF) *m/z*: 1788.928 ([M]$^+$). IR: $\tilde{\nu}$ [cm^{-1}] = 3061 (w), 3015 (w), 2947 (w), 2922 (w), 2851 (w), 1591 (m), 1578 (w), 1533 (w), 1508 (w), 1487 (w), 1468 (m), 1447 (s), 1402 (w), 1362 (w), 1331 (w), 1283 (w), 1269 (w), 1236 (m), 1225 (w), 1192 (w), 1165 (w), 1136 (w), 1111 (m), 1061 (w), 1038 (w), 1018 (w), 989 (w), 947 (w), 932 (w), 872 (w), 824 (w), 793 (s), 735 (s), 718 (m), 683 (w), 662 (m). Anal. calcd. for $C_{112}H_{104}N_4S_8Si$ [1790.7]: C 75.13, H 5.85, N 3.13, S 14.32; Found: C 74.97, H 6.06, N 2.97, S 14.13.

3. ^{1}H and ^{13}C NMR spectra

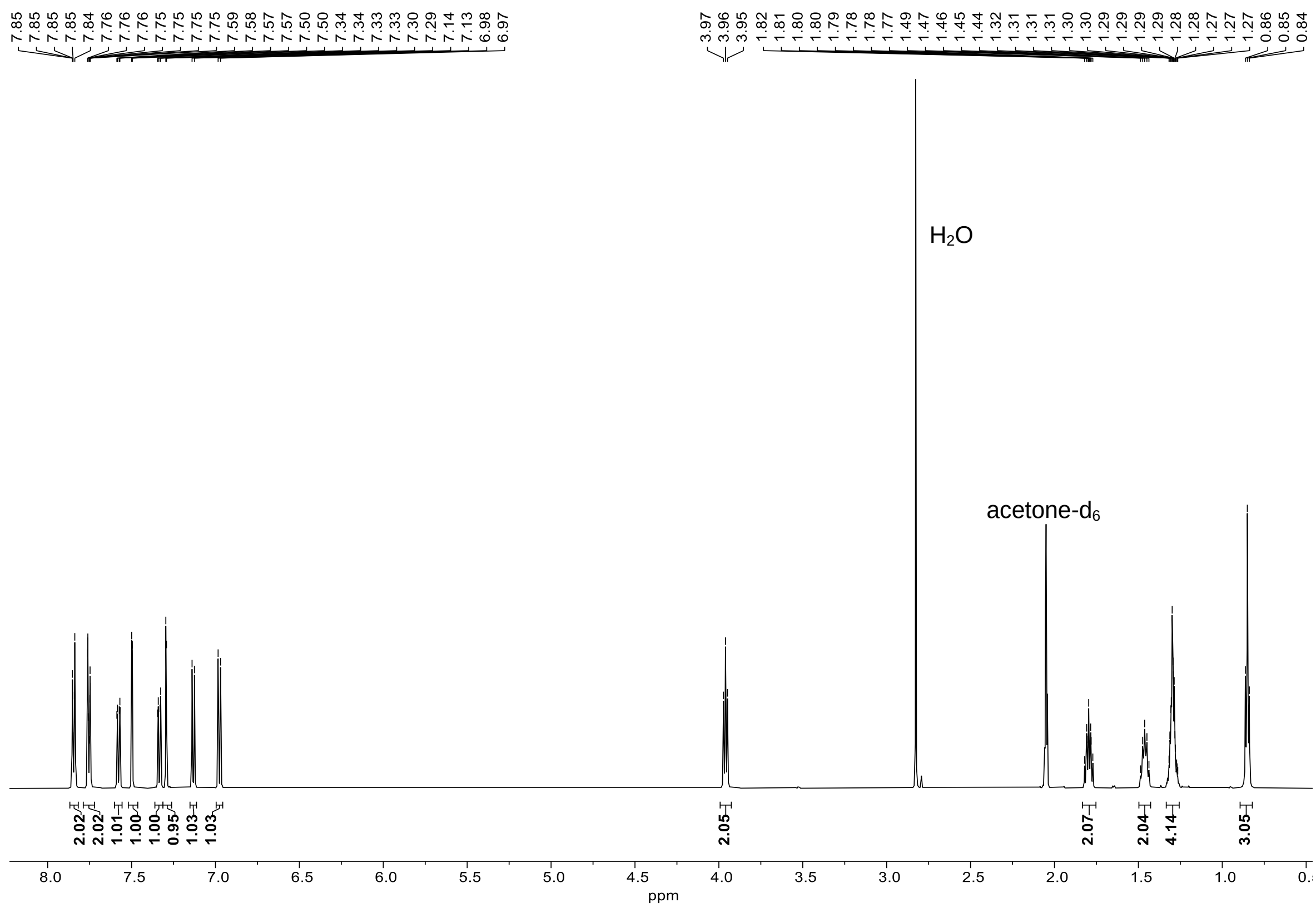

Figure S1. ^{1}H NMR spectrum (acetone-d$_6$, 600 MHz, *T* = 298 K) of compound **4g**.

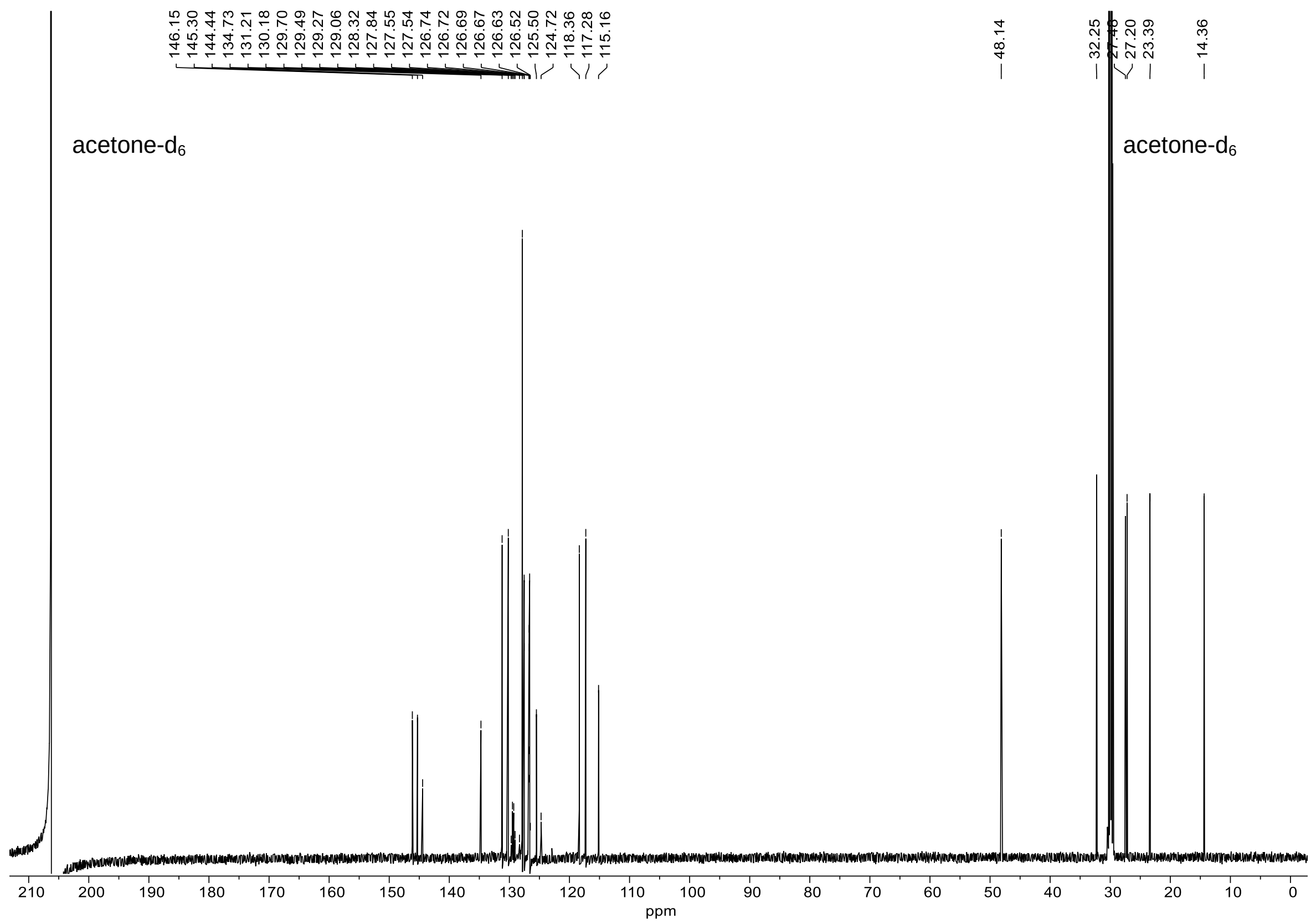

Figure S2. ^{13}C NMR spectrum (acetone-d$_6$, 150 MHz, *T* = 298 K) of compound **4g**.

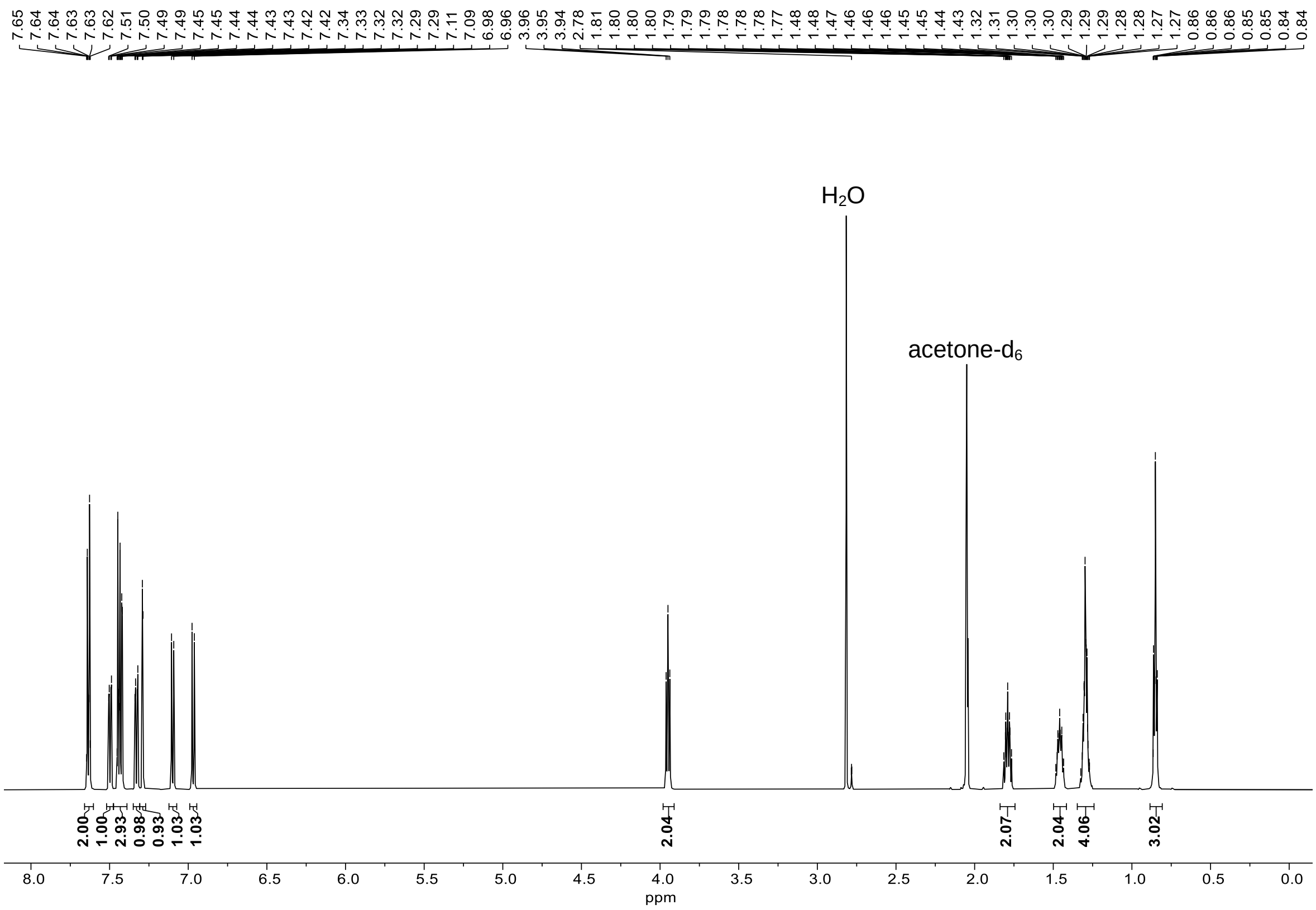

Figure S3. ^{1}H NMR spectrum (acetone-d$_6$, 600 MHz, T = 298 K) of compound **4h**.

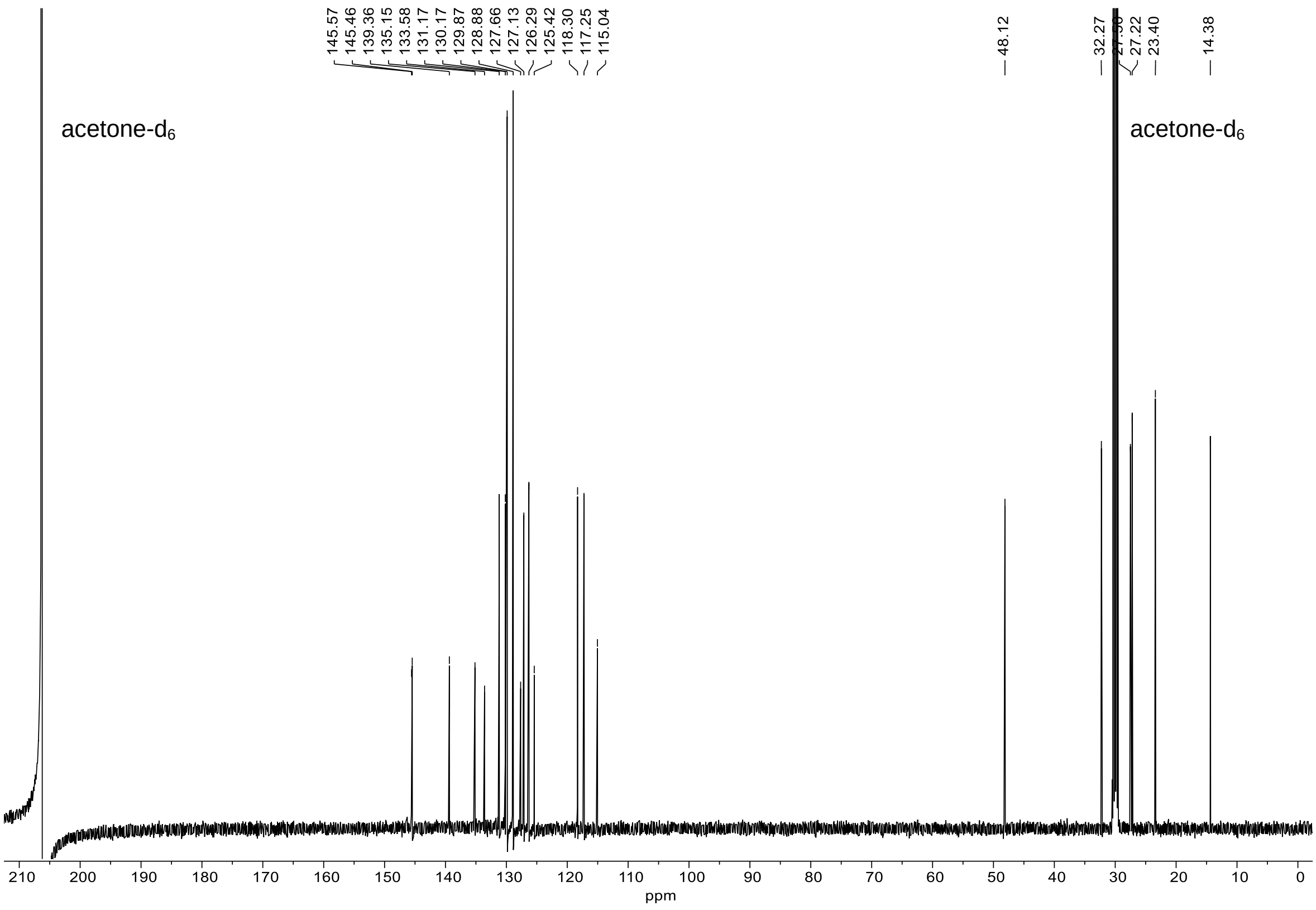

Figure S4. ^{13}C NMR spectrum (acetone-d$_6$, 150 MHz, T = 298 K) of compound **4h**.

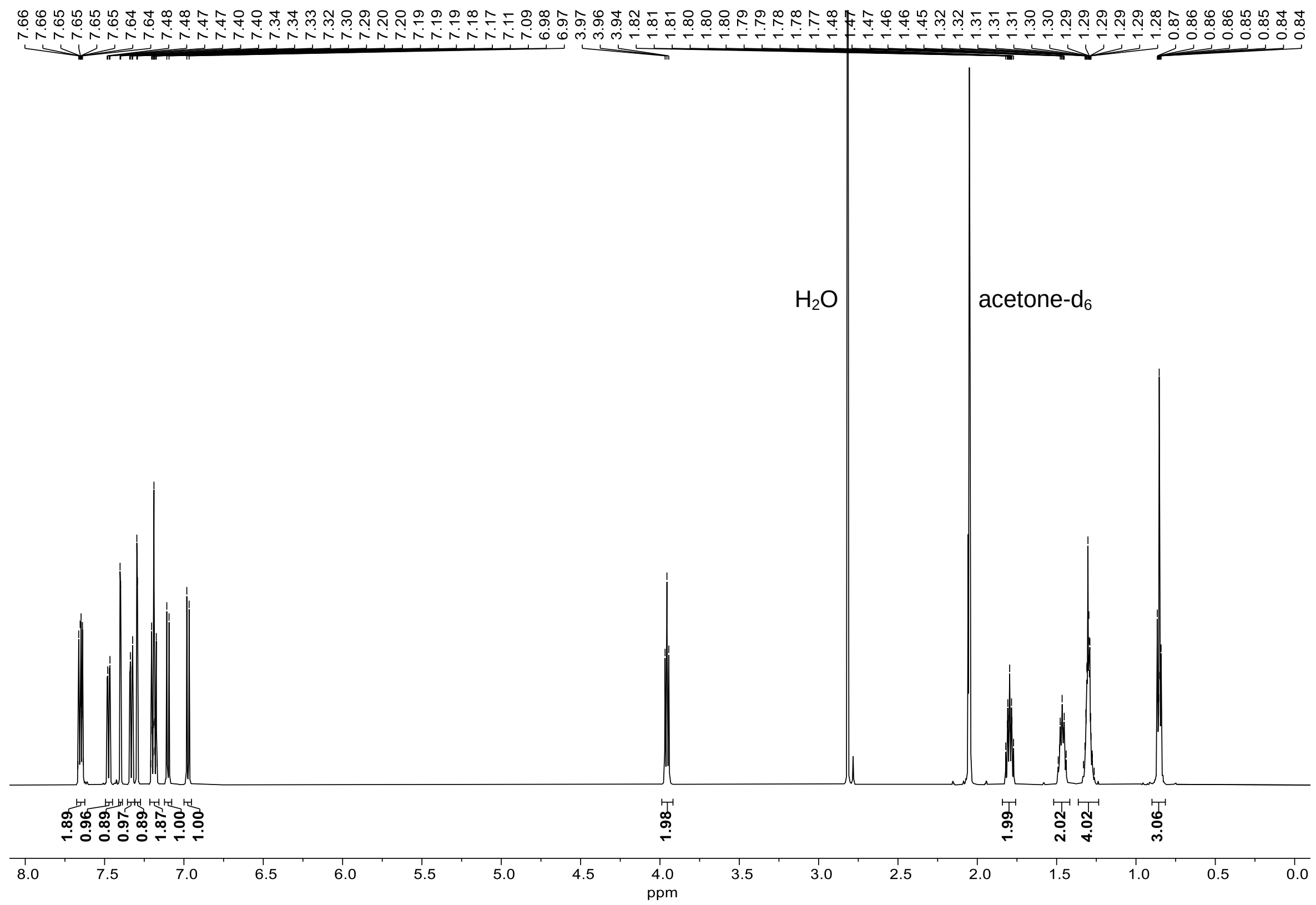

Figure S5. ^{1}H NMR spectrum (acetone-d$_6$, 600 MHz, T = 298 K) of compound **4i**.

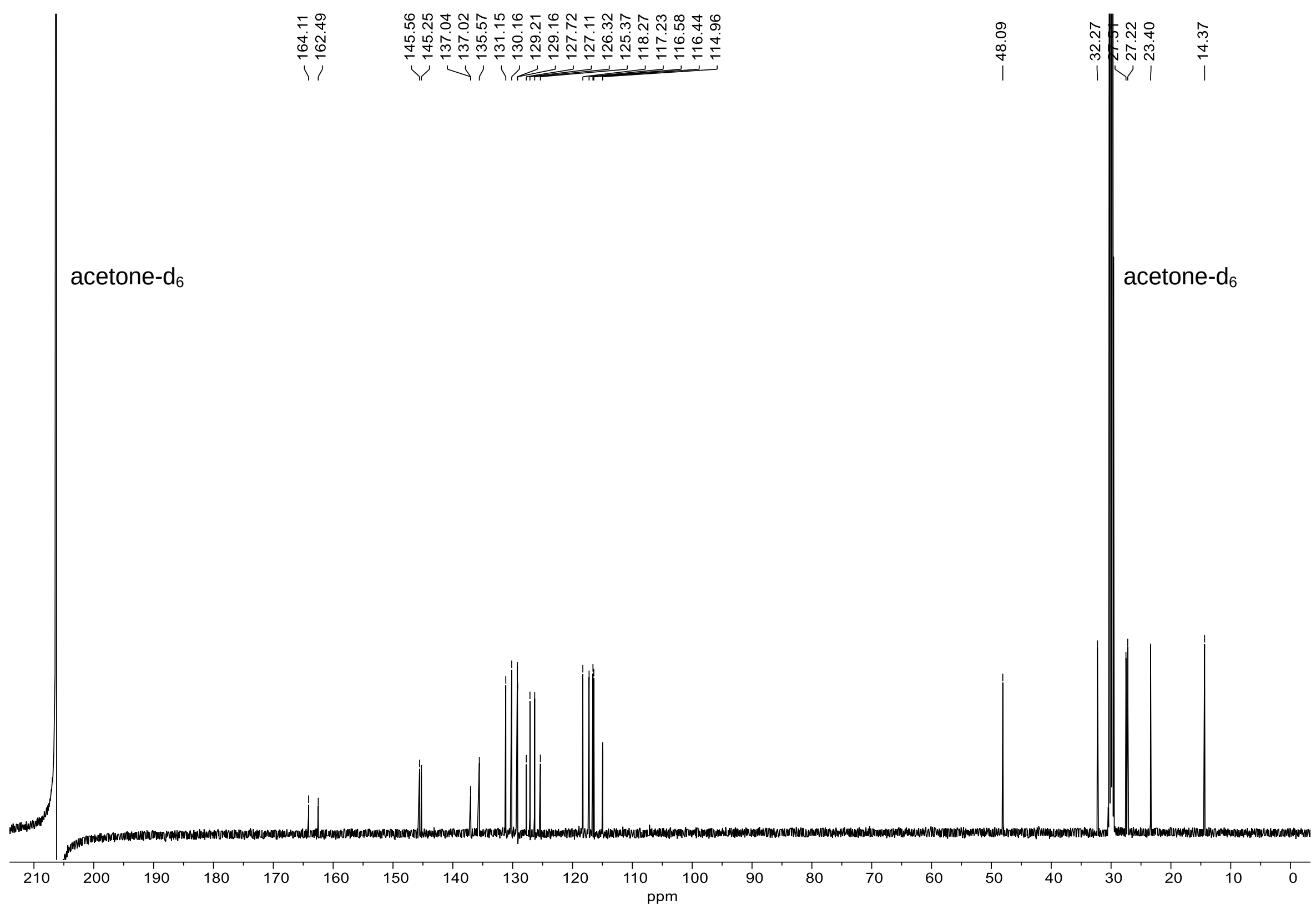

Figure S6. ^{13}C NMR spectrum (acetone-d$_6$, 150 MHz, T = 298 K) of compound **4i**.

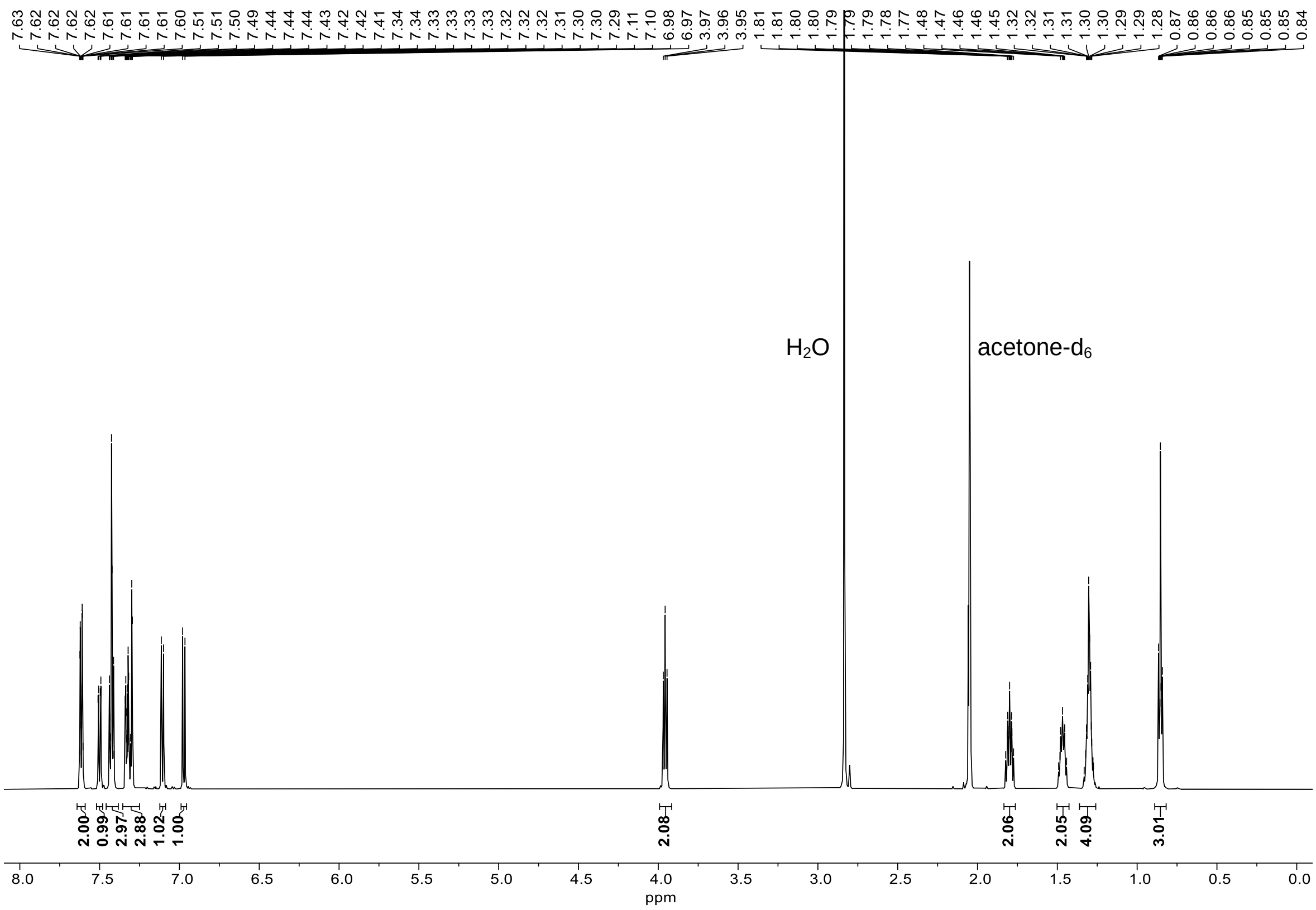

Figure S7. ^{1}H NMR spectrum (acetone-d6, 600 MHz, *T* = 298 K) of compound **4j**.

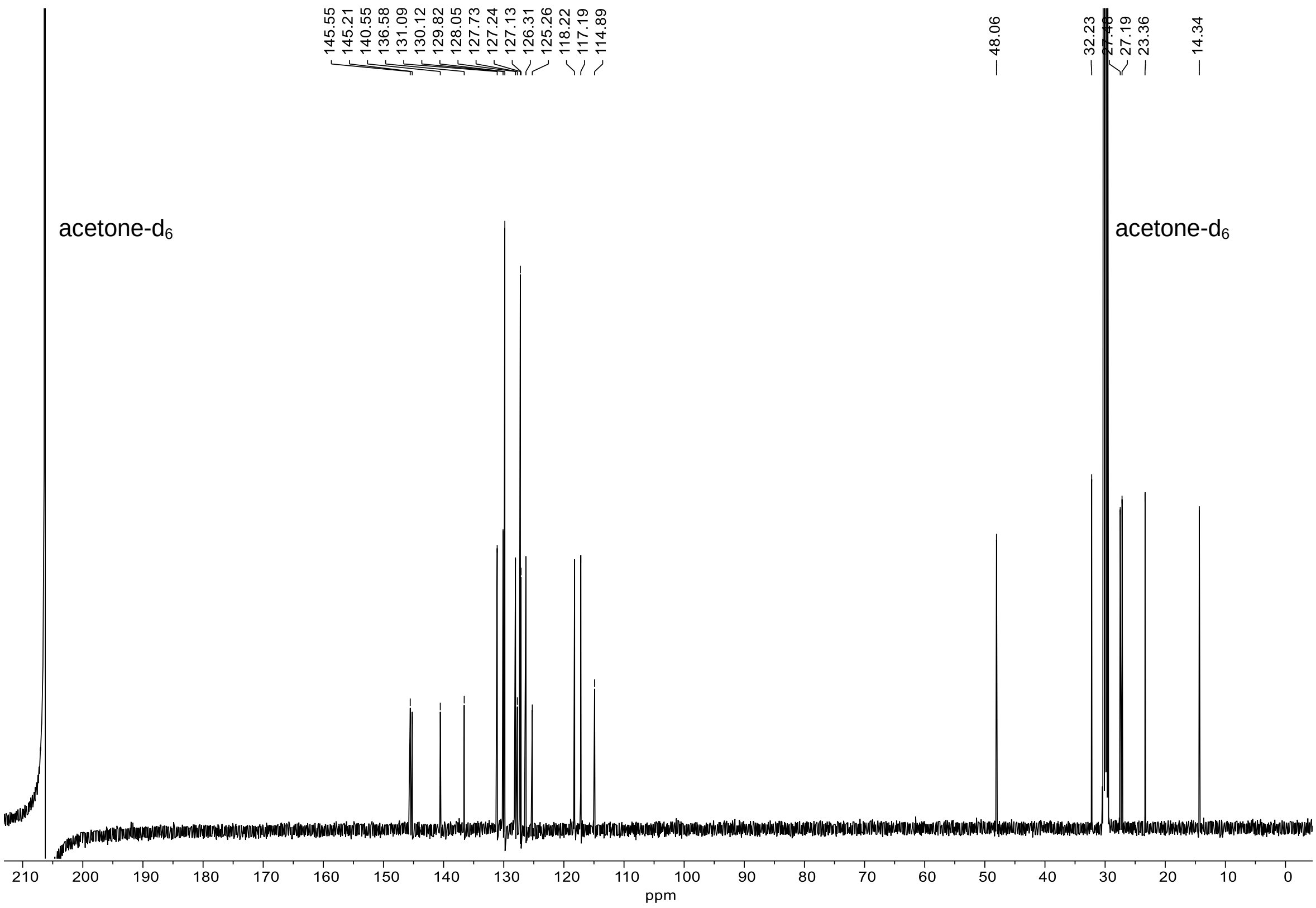

Figure S8. ^{13}C NMR spectrum (acetone-d6, 150 MHz, *T* = 298 K) of compound **4j**.

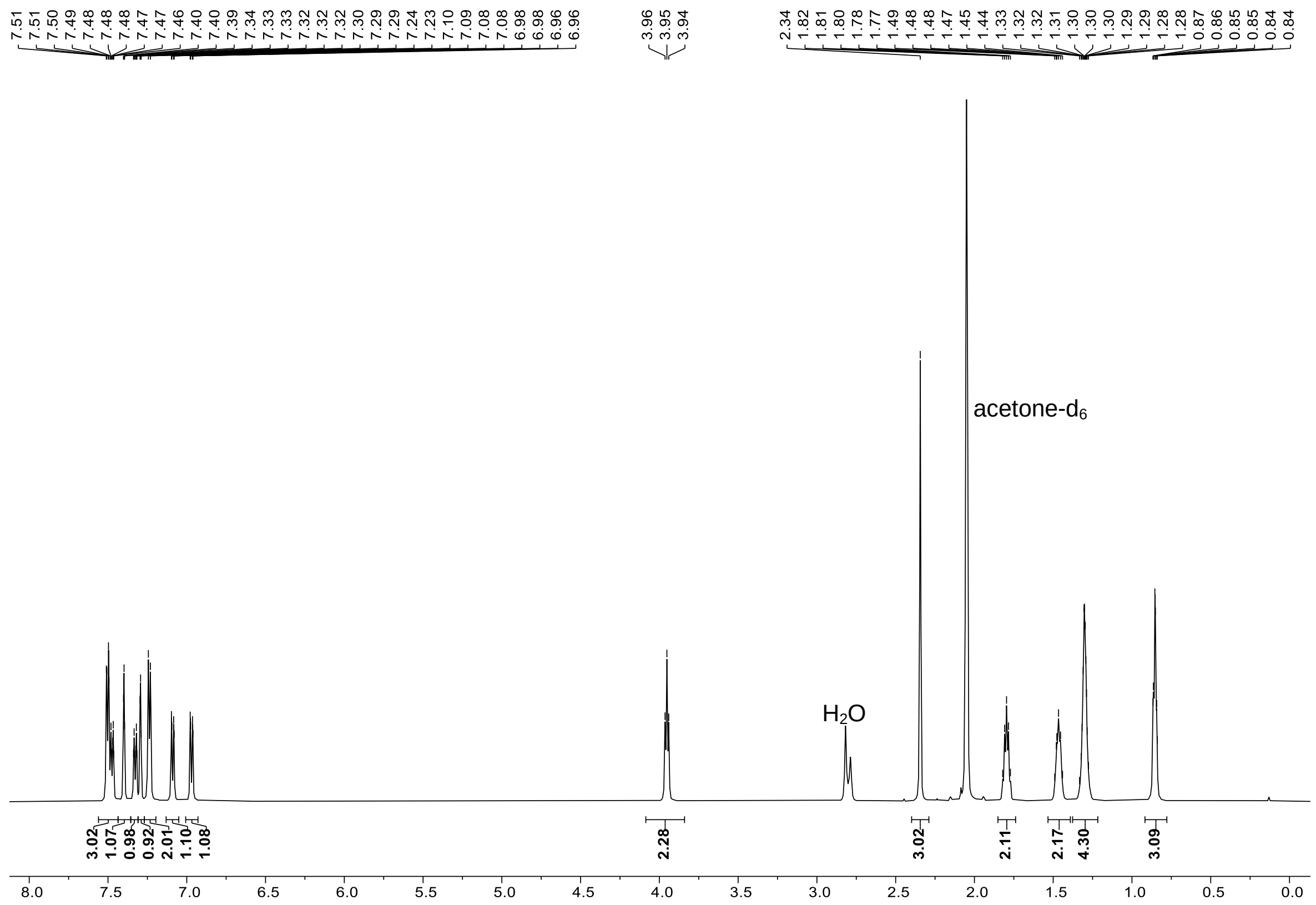

Figure S9. ^{1}H NMR spectrum (acetone-d$_6$, 600 MHz, T = 298 K) of compound **4k**.

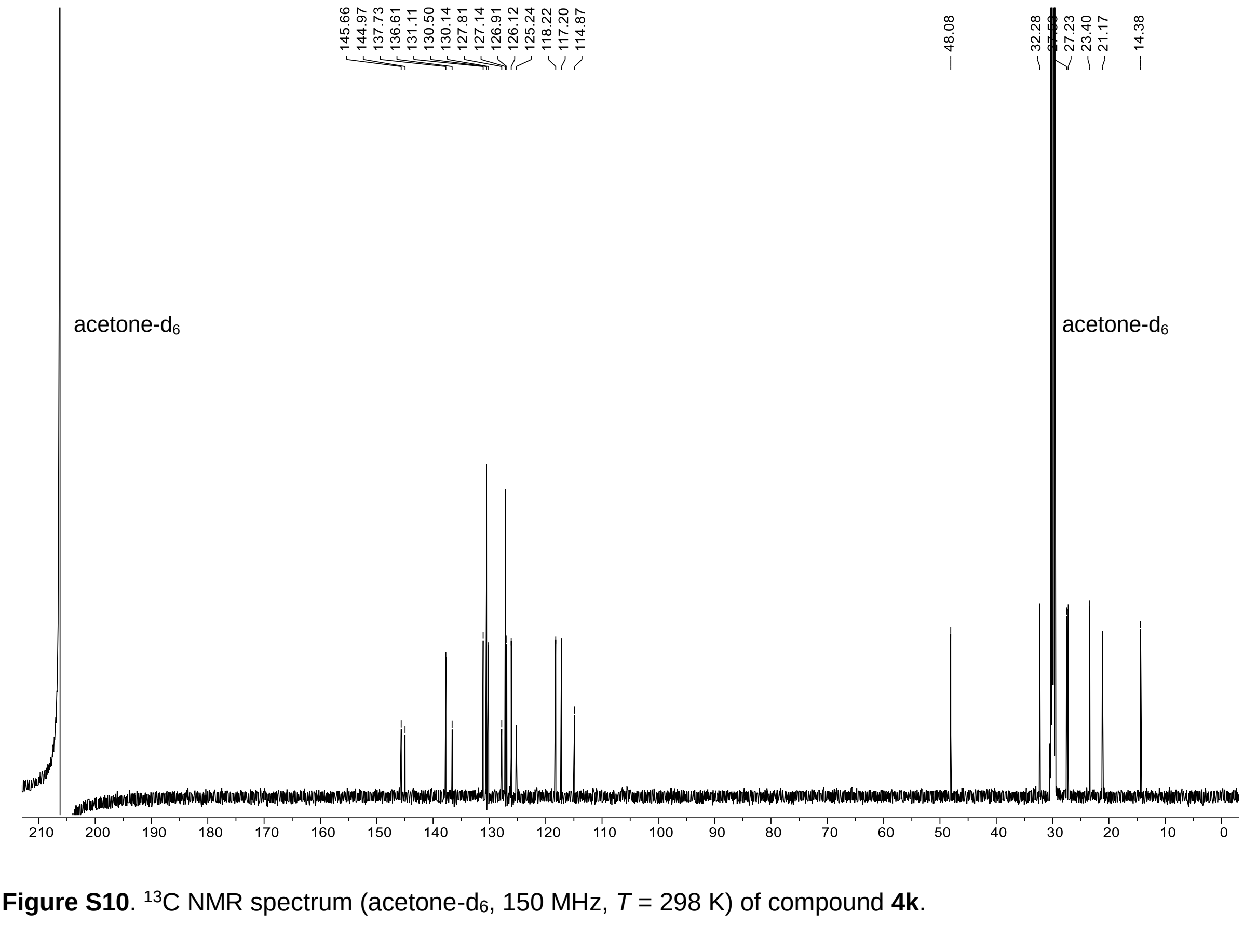

Figure S10. ^{13}C NMR spectrum (acetone-d$_6$, 150 MHz, T = 298 K) of compound **4k**.

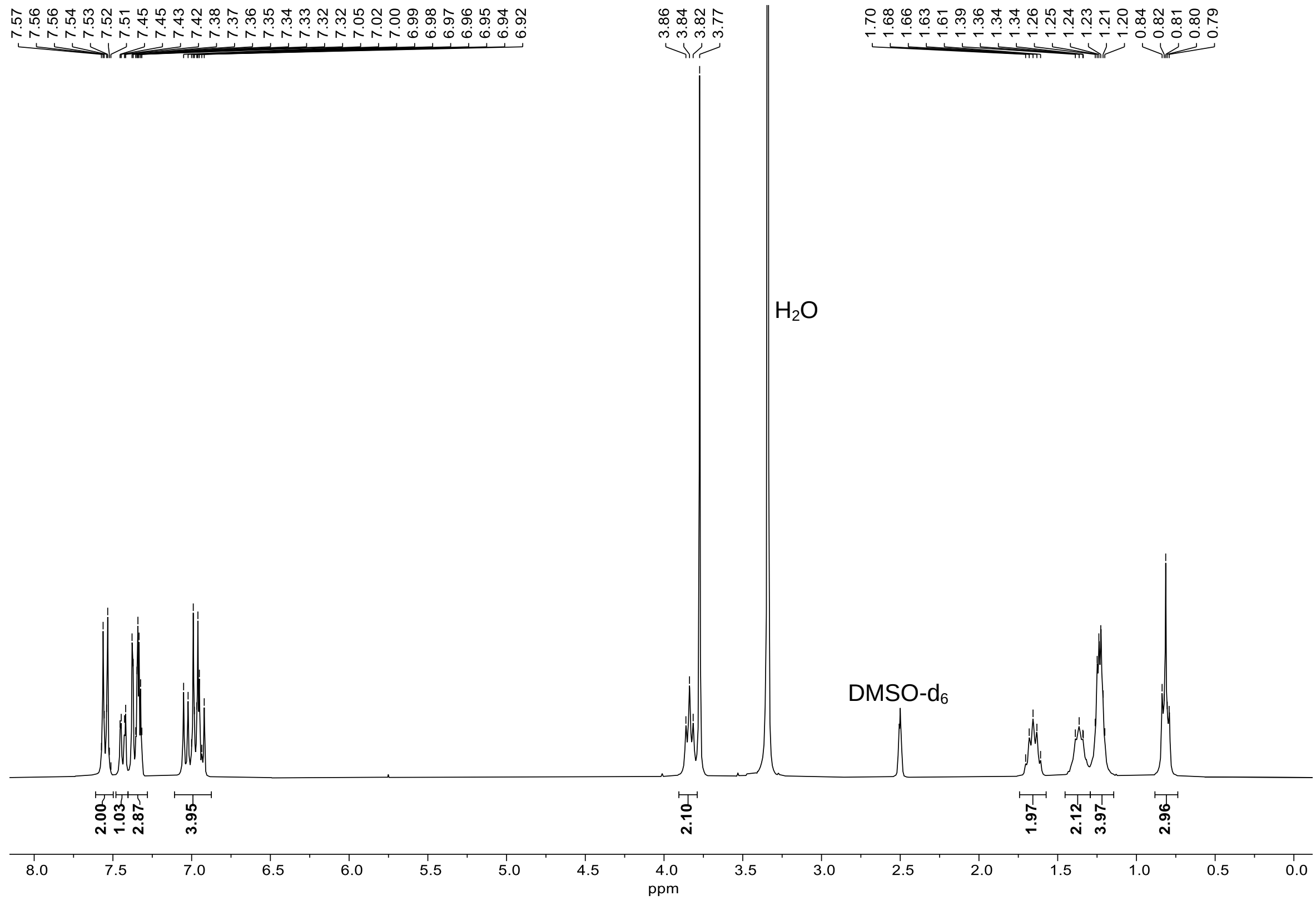

Figure S11. ^{1}H NMR spectrum (DMSO-d$_6$, 300 MHz, T = 298 K) of compound **4l**.

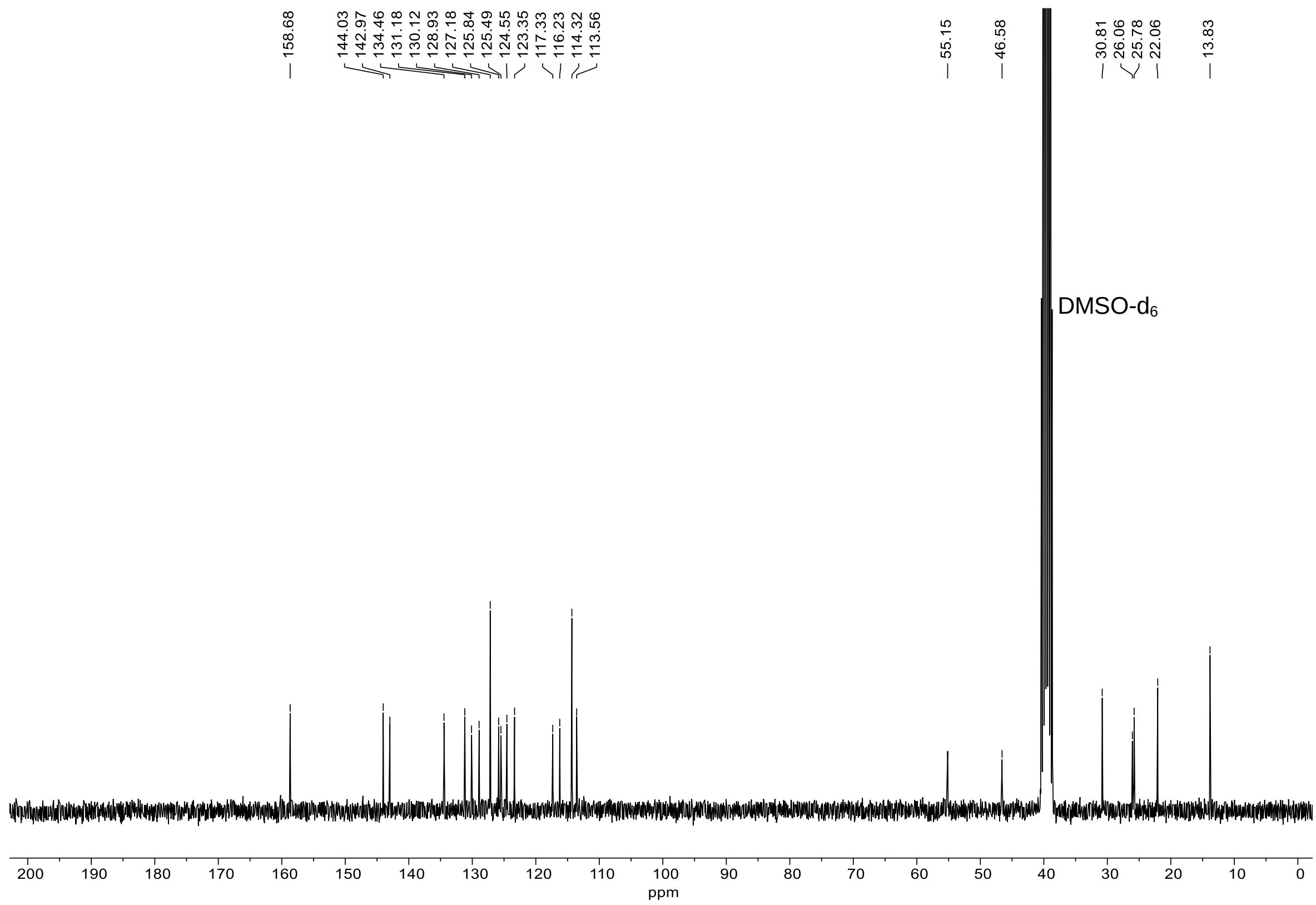

Figure S12. ^{13}C NMR spectrum (DMSO-d$_6$, 75 MHz, T = 298 K) of compound **4l**.

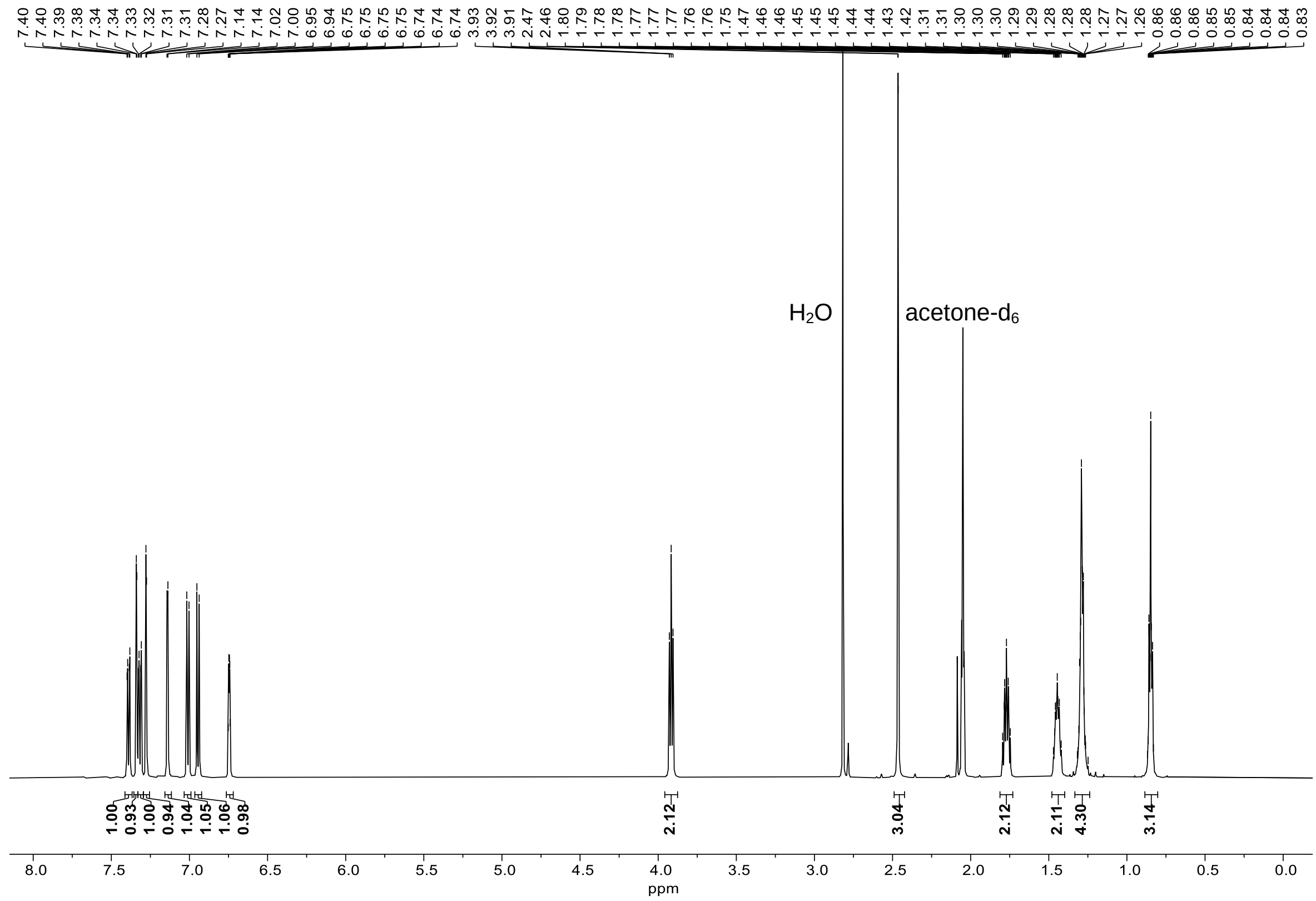

Figure S13. ^{1}H NMR spectrum (acetone-d6, 600 MHz, T = 298 K) of compound **4m**.

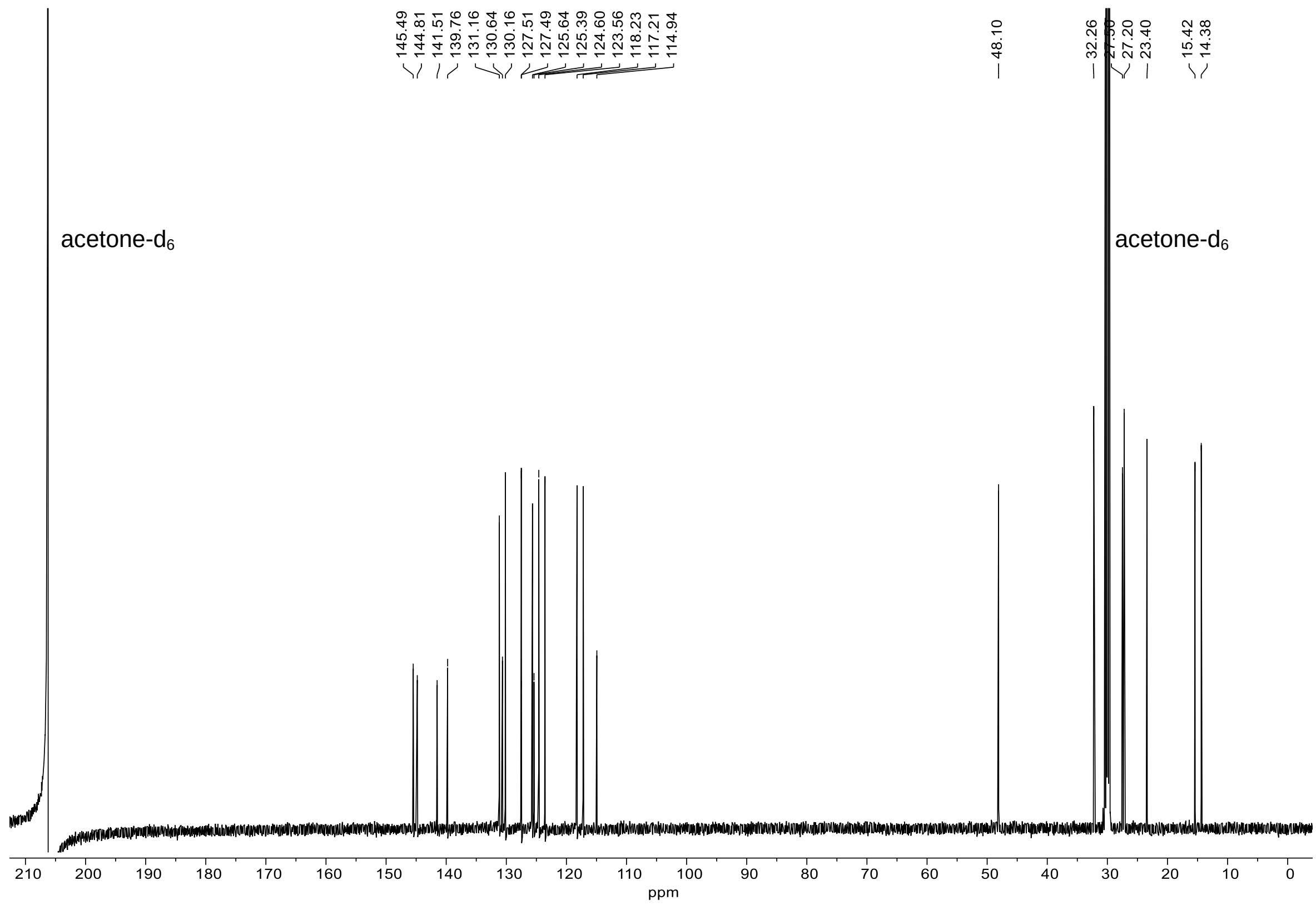

Figure S14. ^{13}C NMR spectrum (acetone-d6, 150 MHz, T = 298 K) of compound **4m**.

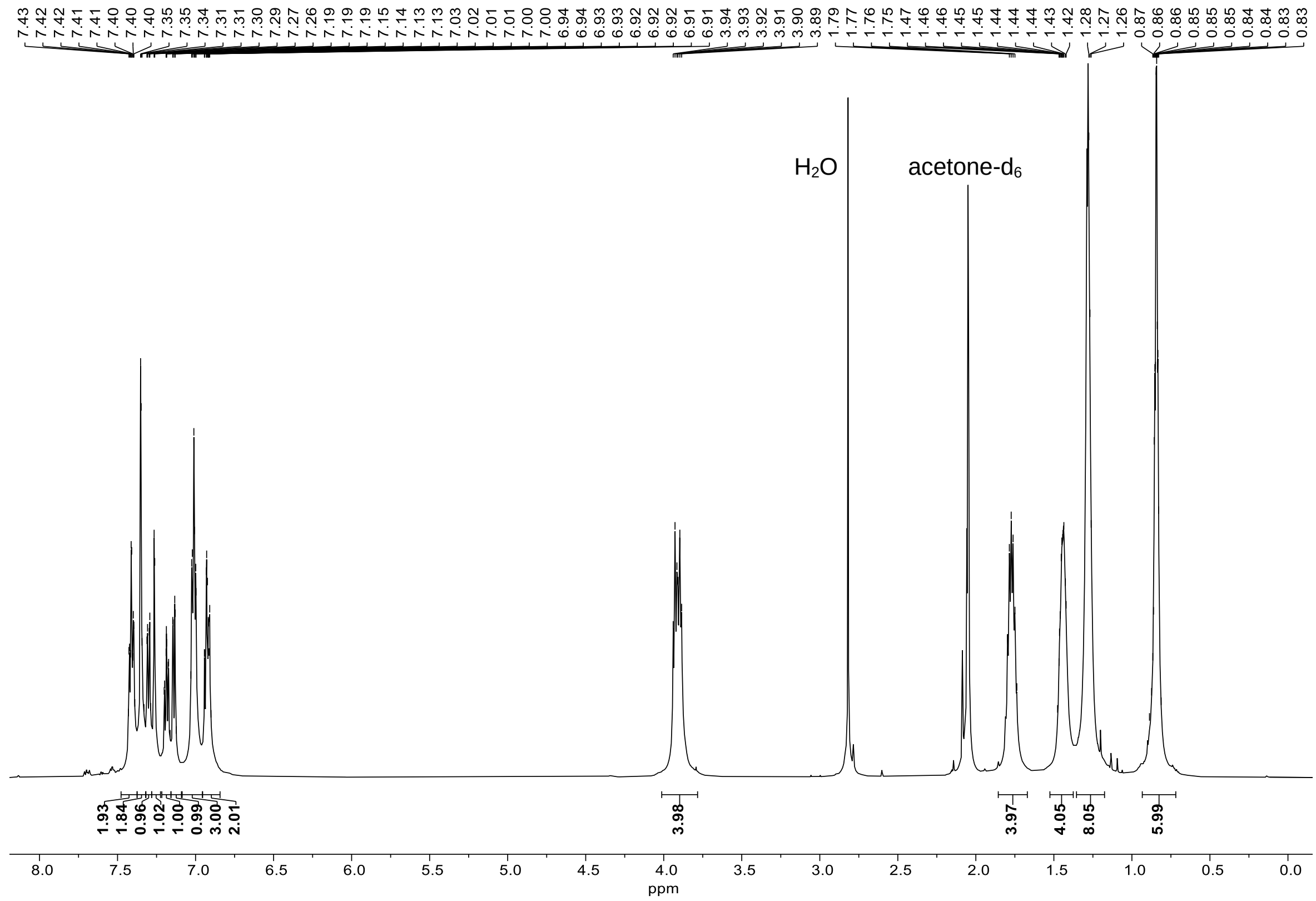

Figure S15. ^{1}H NMR spectrum (acetone-d_6, 600 MHz, T = 298 K) of compound **4n**.

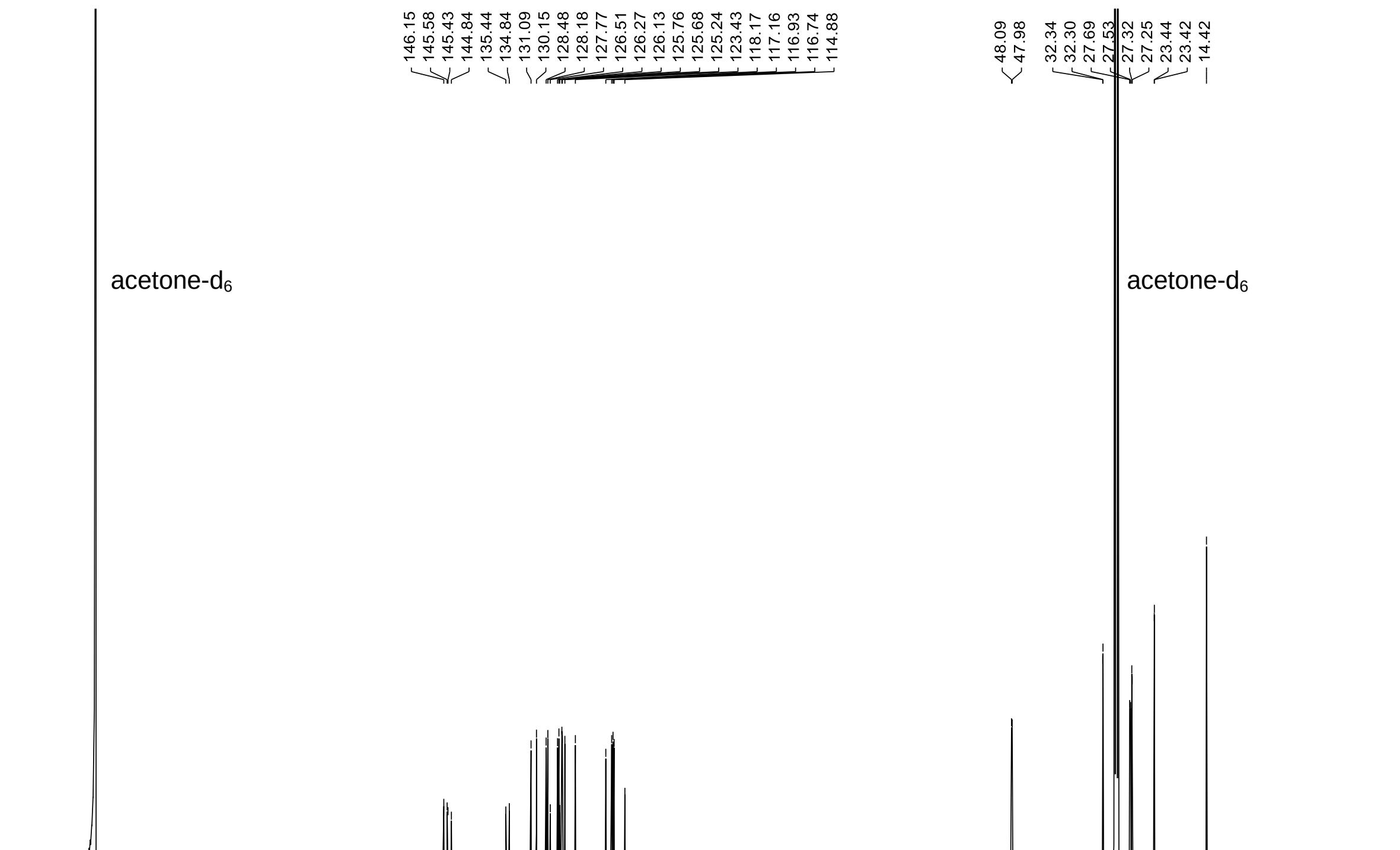

Figure S16. ^{13}C NMR spectrum (acetone-d_6, 150 MHz, T = 298 K) of compound **4n**.

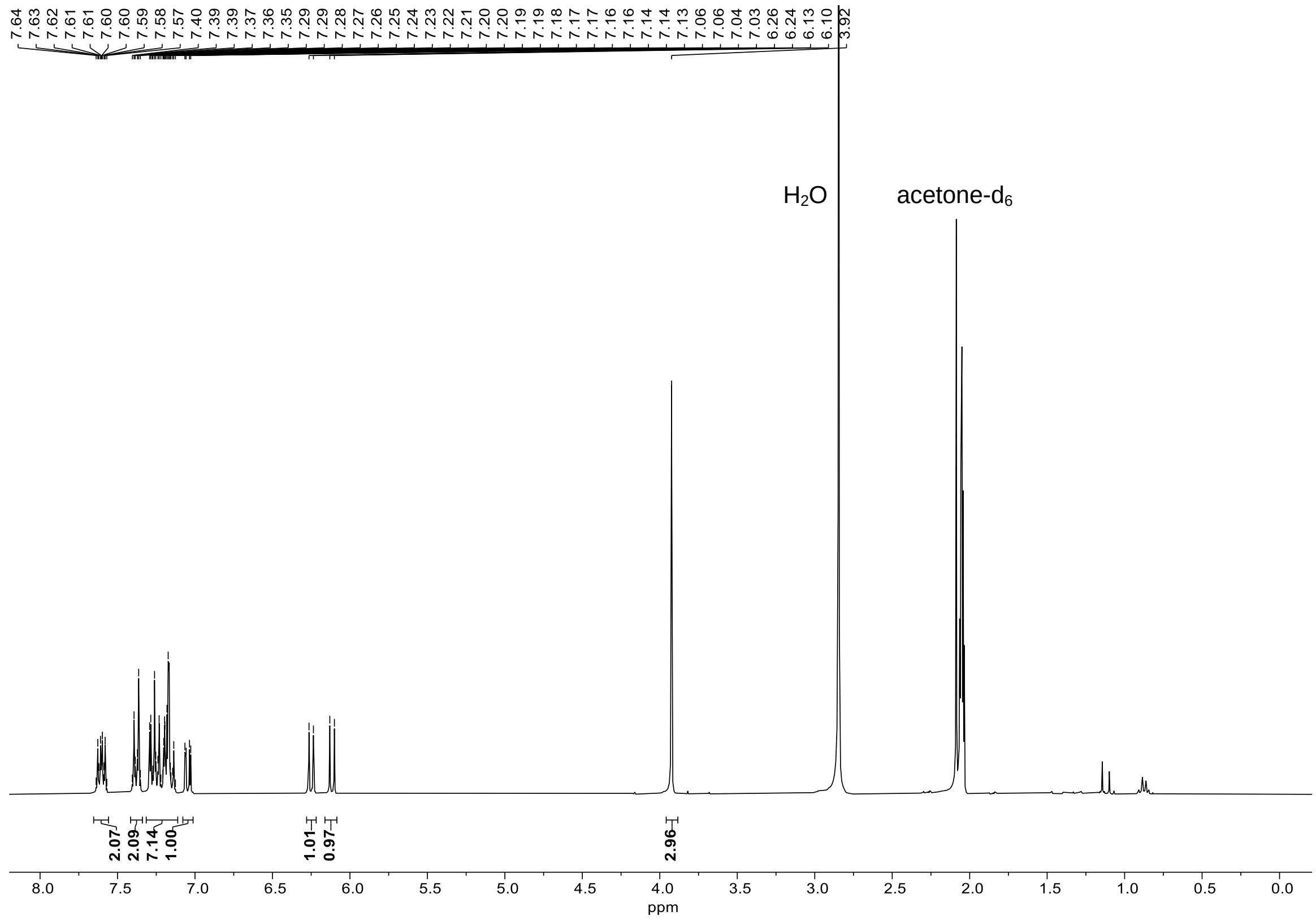

Figure S17. ^{1}H NMR spectrum (acetone-d$_6$, 300 MHz, T = 298 K) of compound **4f**.

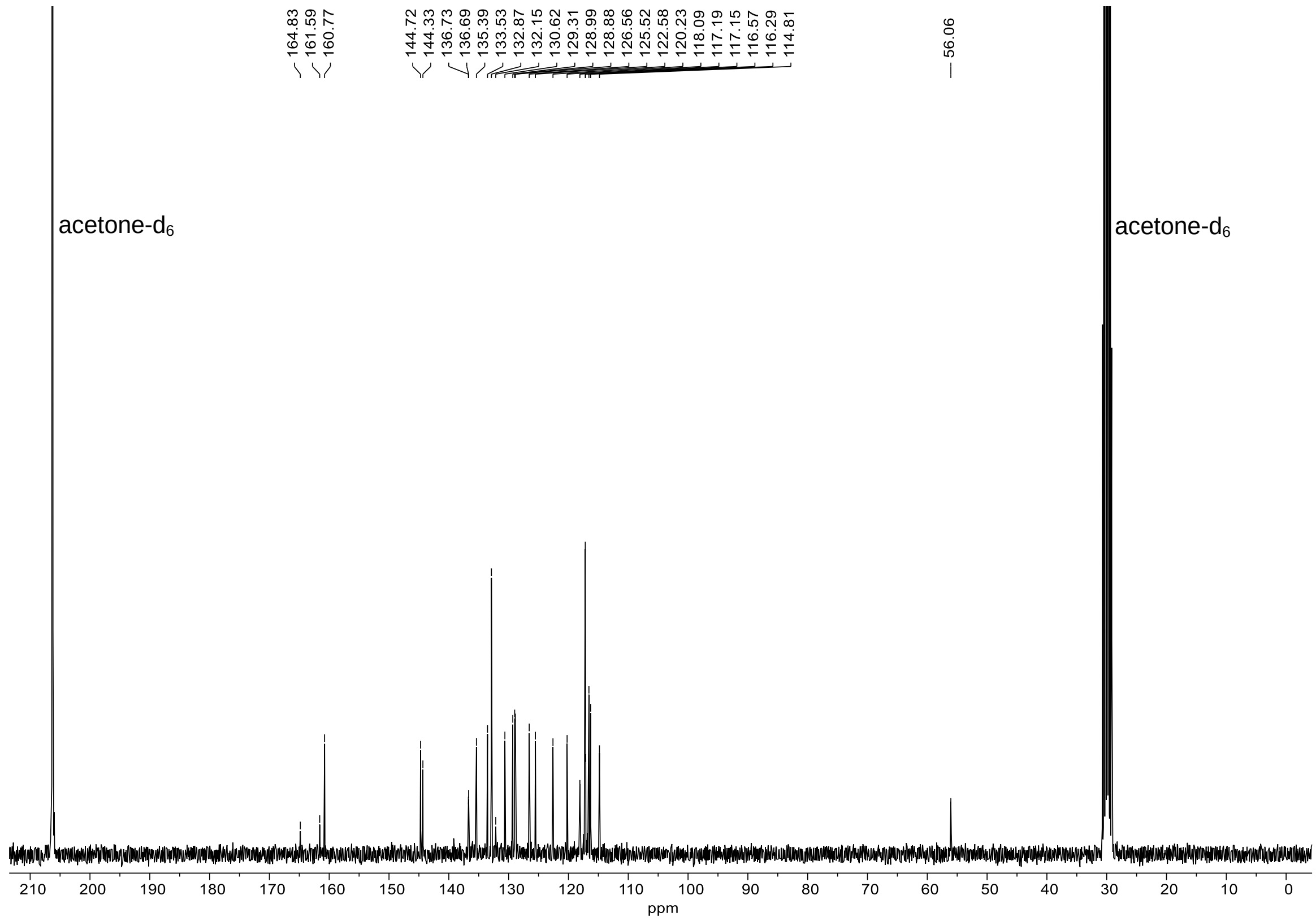

Figure S18. ^{13}C NMR spectrum (acetone-d$_6$, 75 MHz, T = 298 K) of compound **4f**.

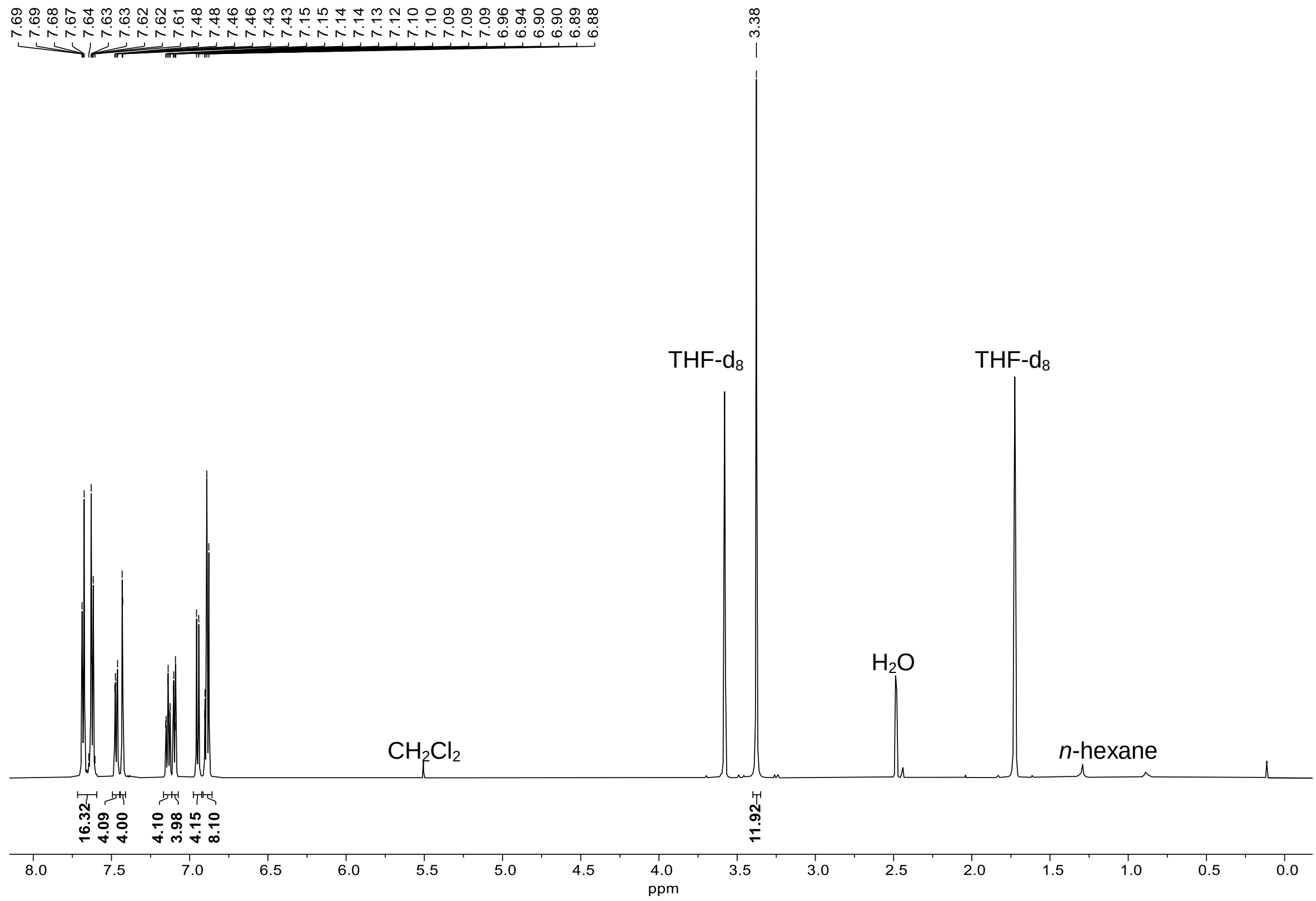

Figure S19. ^{1}H NMR spectrum (THF-d$_8$, 600 MHz, T = 298 K) of compound **5a**.

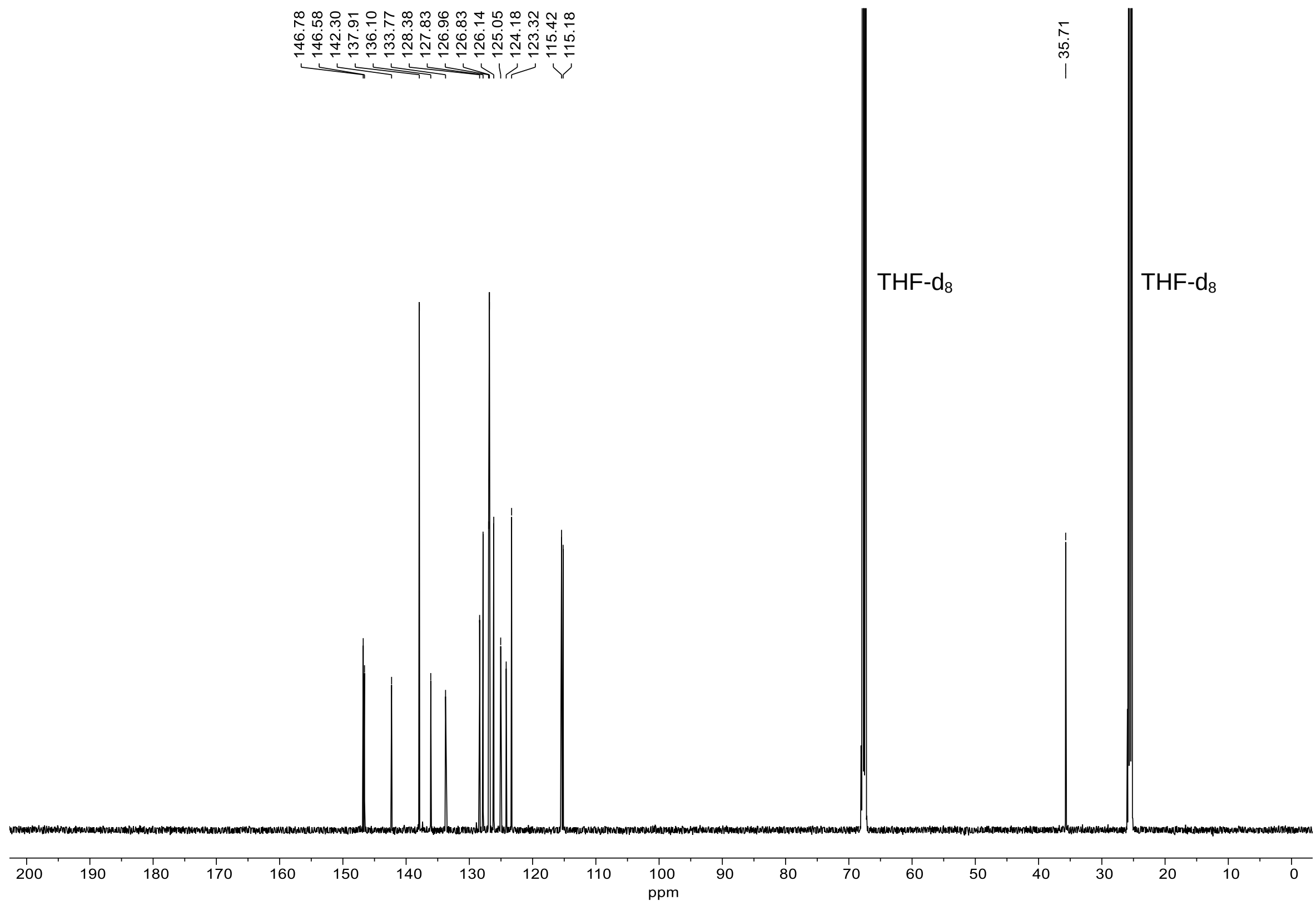

Figure S20. ^{13}C NMR spectrum (THF-d$_8$, 150 MHz, T = 298 K) of compound **5a**.

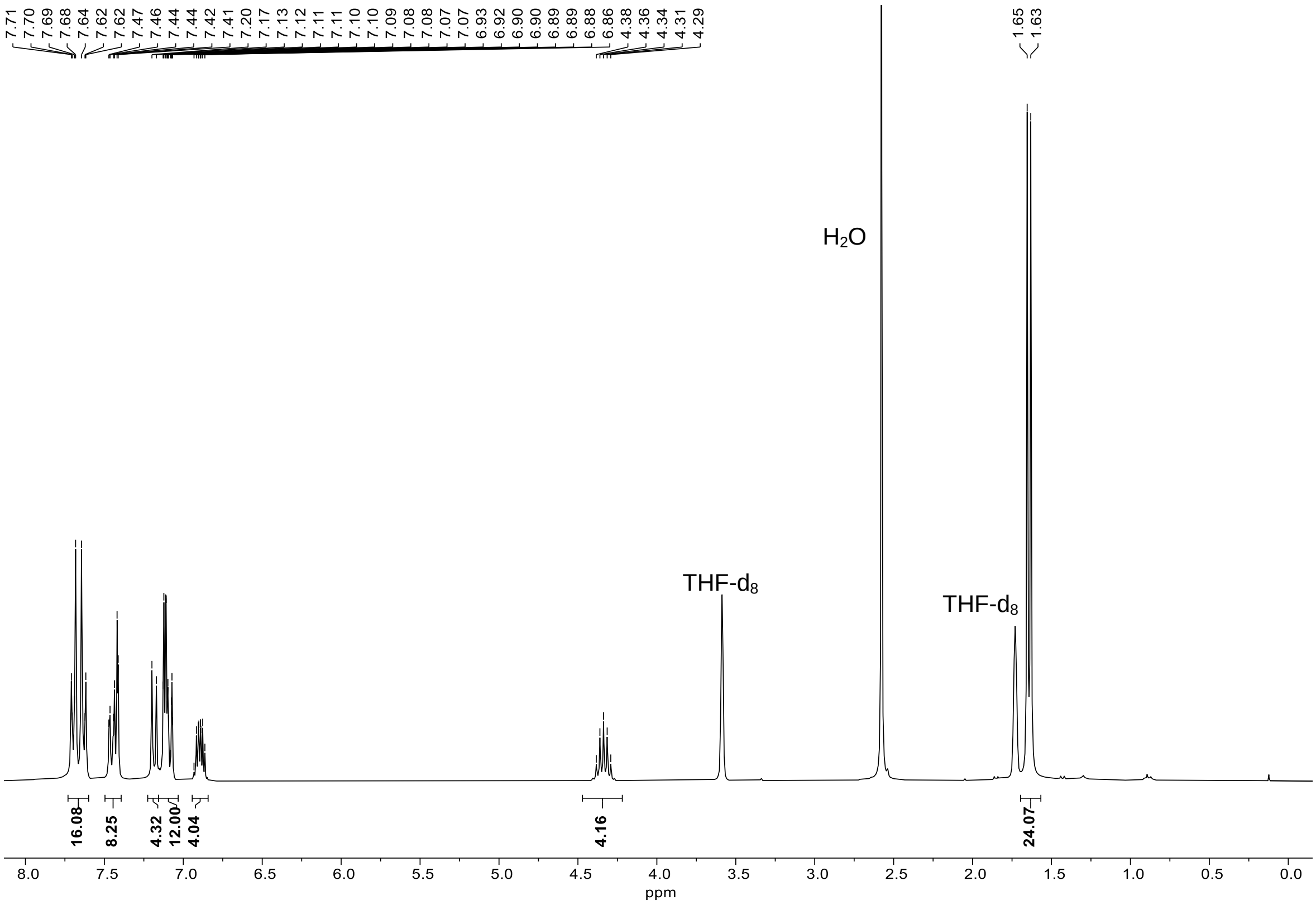

Figure S21. ^{1}H NMR spectrum (THF-d$_8$, 300 MHz, T = 298 K) of compound **5b**.

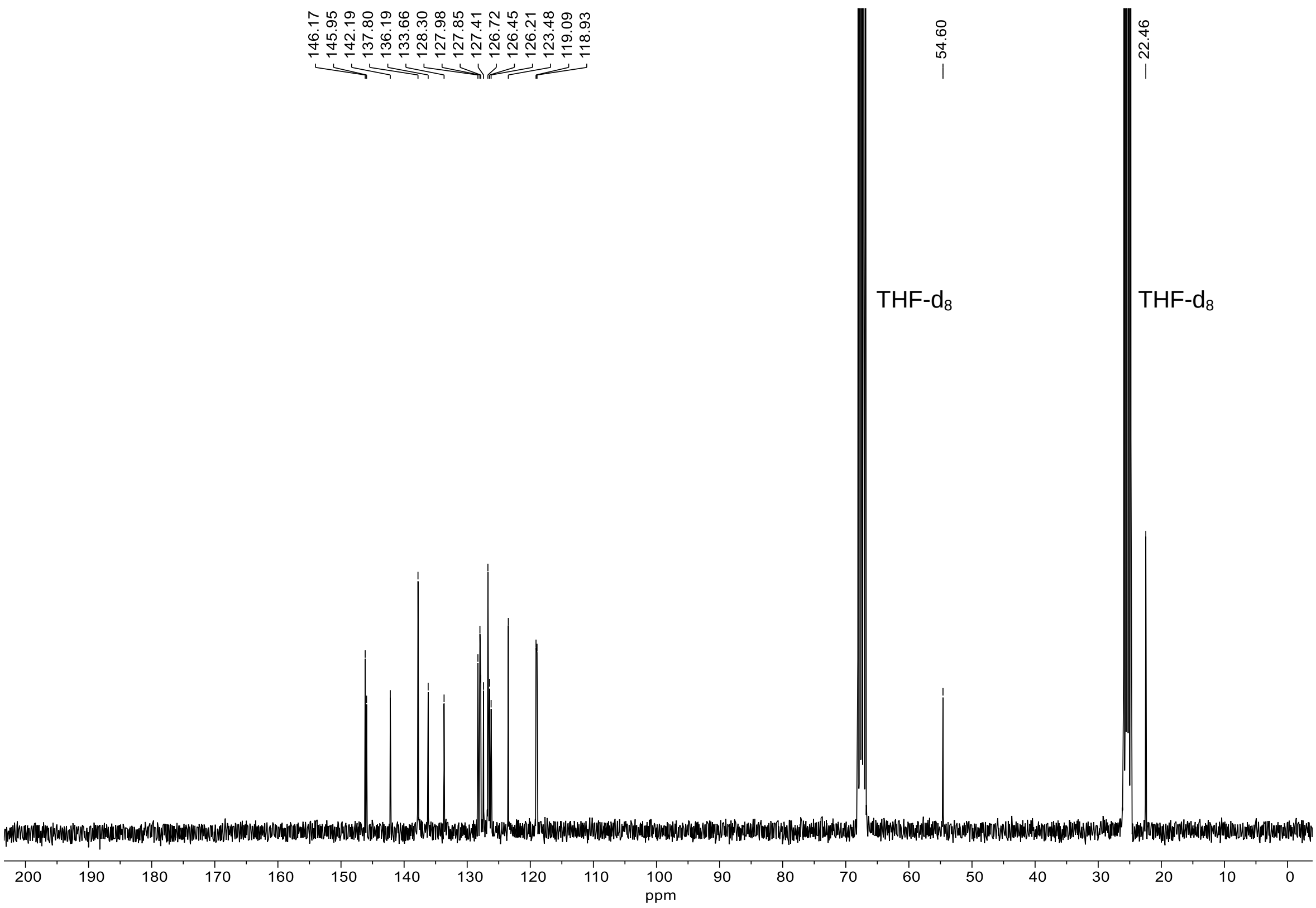

Figure S22. ^{13}C NMR spectrum (THF-d$_8$, 75 MHz, T = 298 K) of compound **5b**.

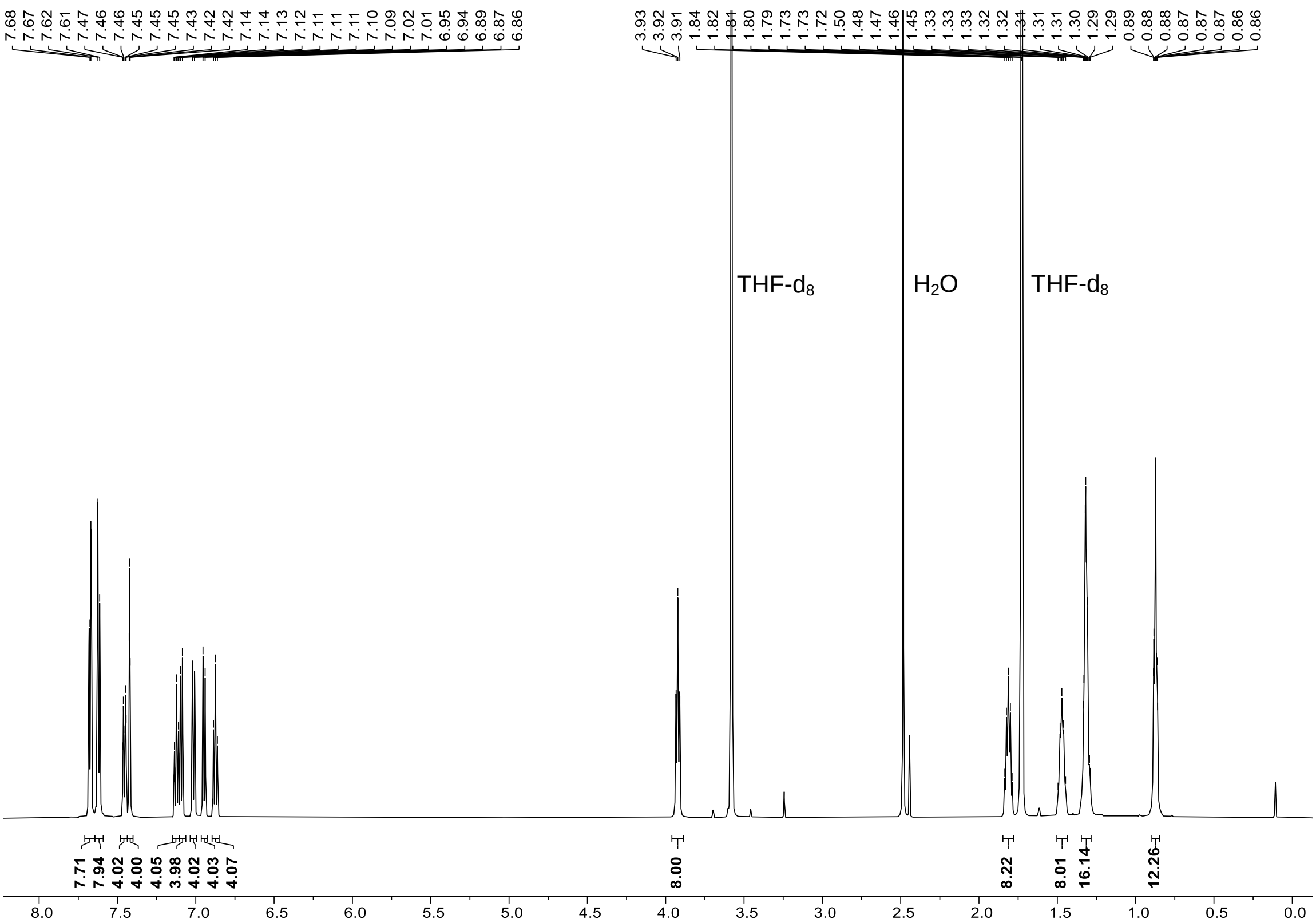

Figure S23. ^{1}H NMR spectrum (THF-d8, 600 MHz, T = 298 K) of compound **5c**.

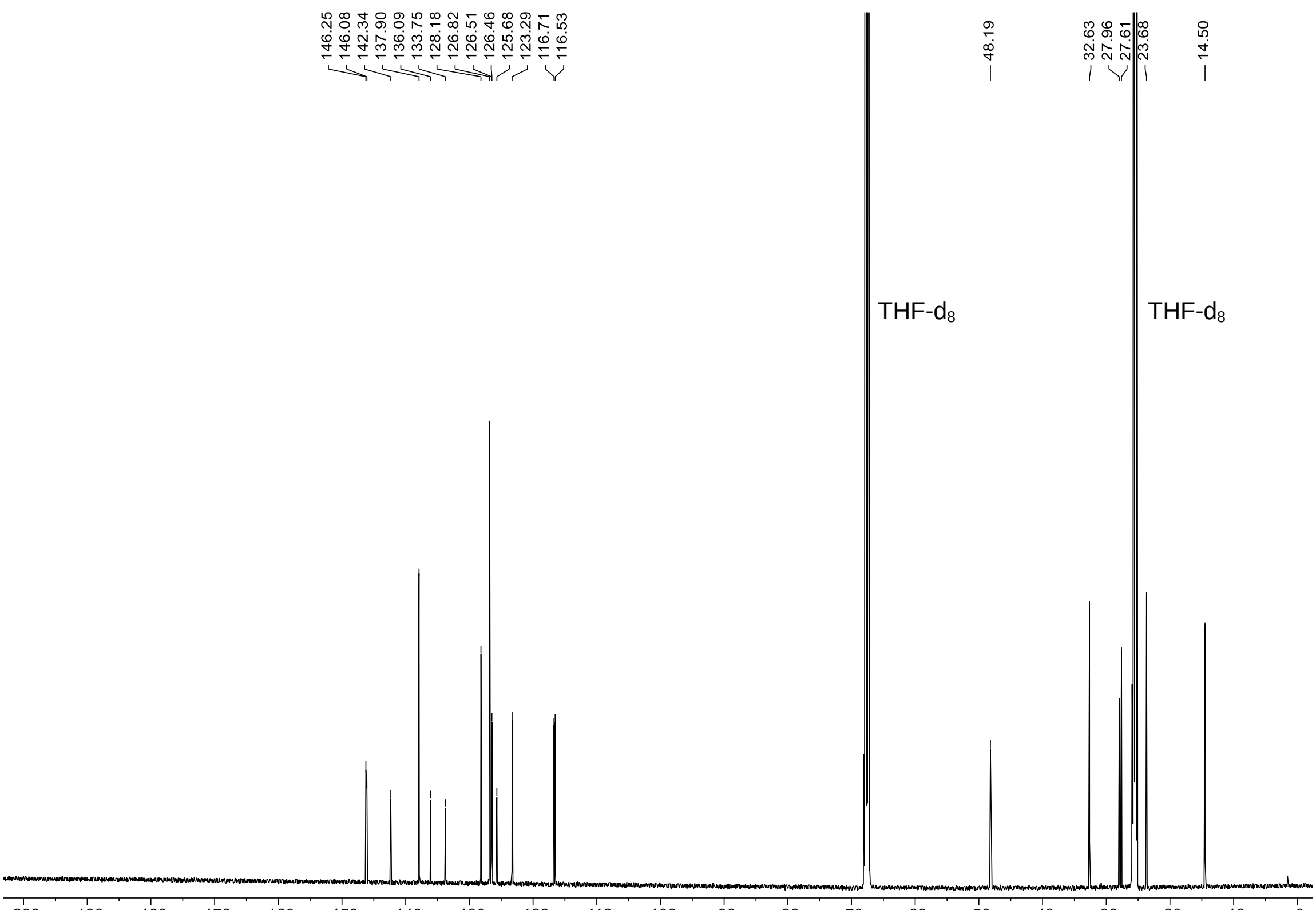

Figure S24. ^{13}C NMR spectrum (THF-d8, 150 MHz, T = 298 K) of compound **5c**.

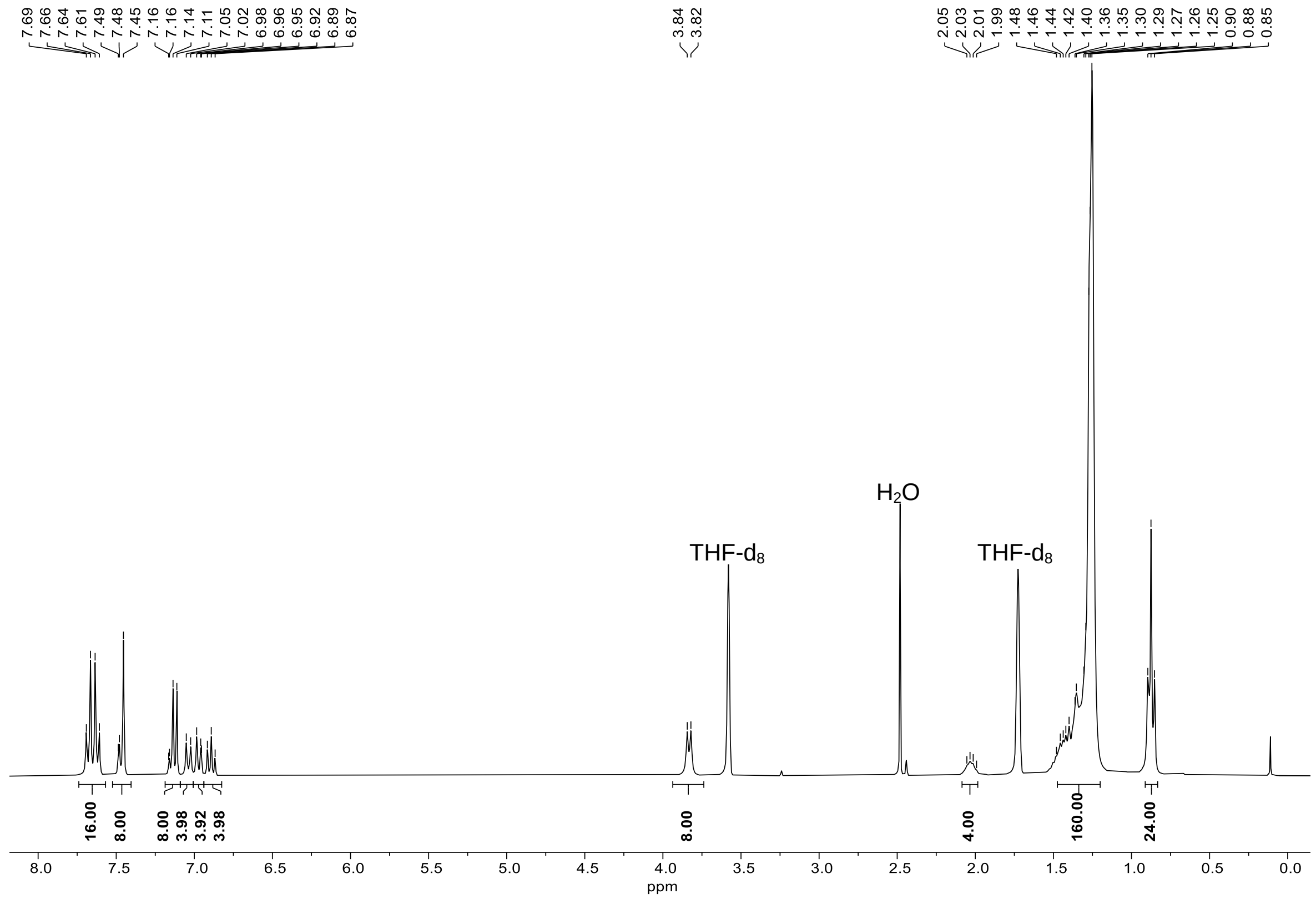

Figure S25. ^{1}H NMR spectrum (THF-d$_8$, 300 MHz, T = 298 K) of compound **5d**.

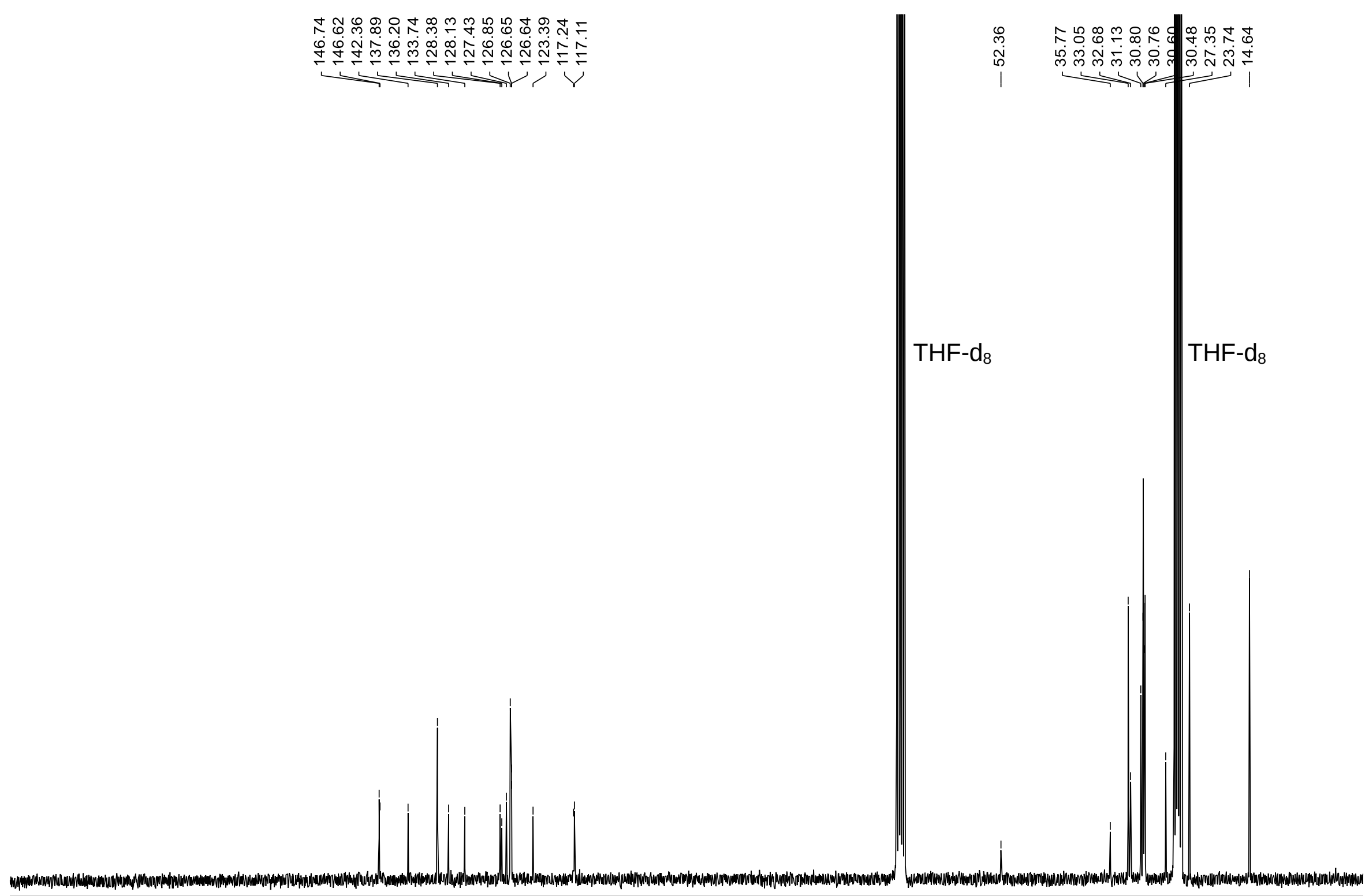

Figure S26. ^{13}C NMR spectrum (THF-d$_8$, 75 MHz, T = 298 K) of compound **5d**.

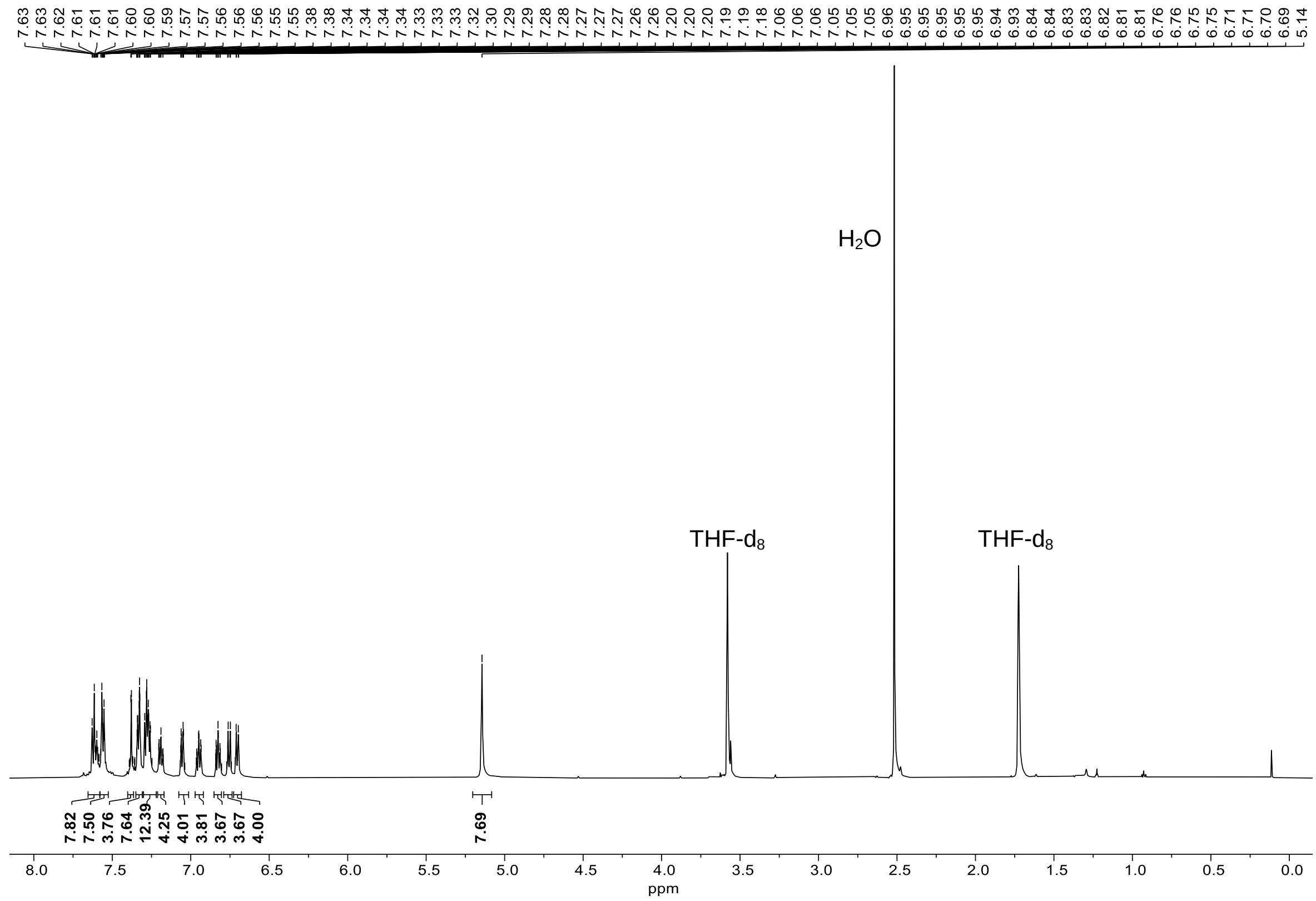

Figure S27. ^{1}H NMR spectrum (THF-d$_8$, 600 MHz, T = 298 K) of compound **5e**.

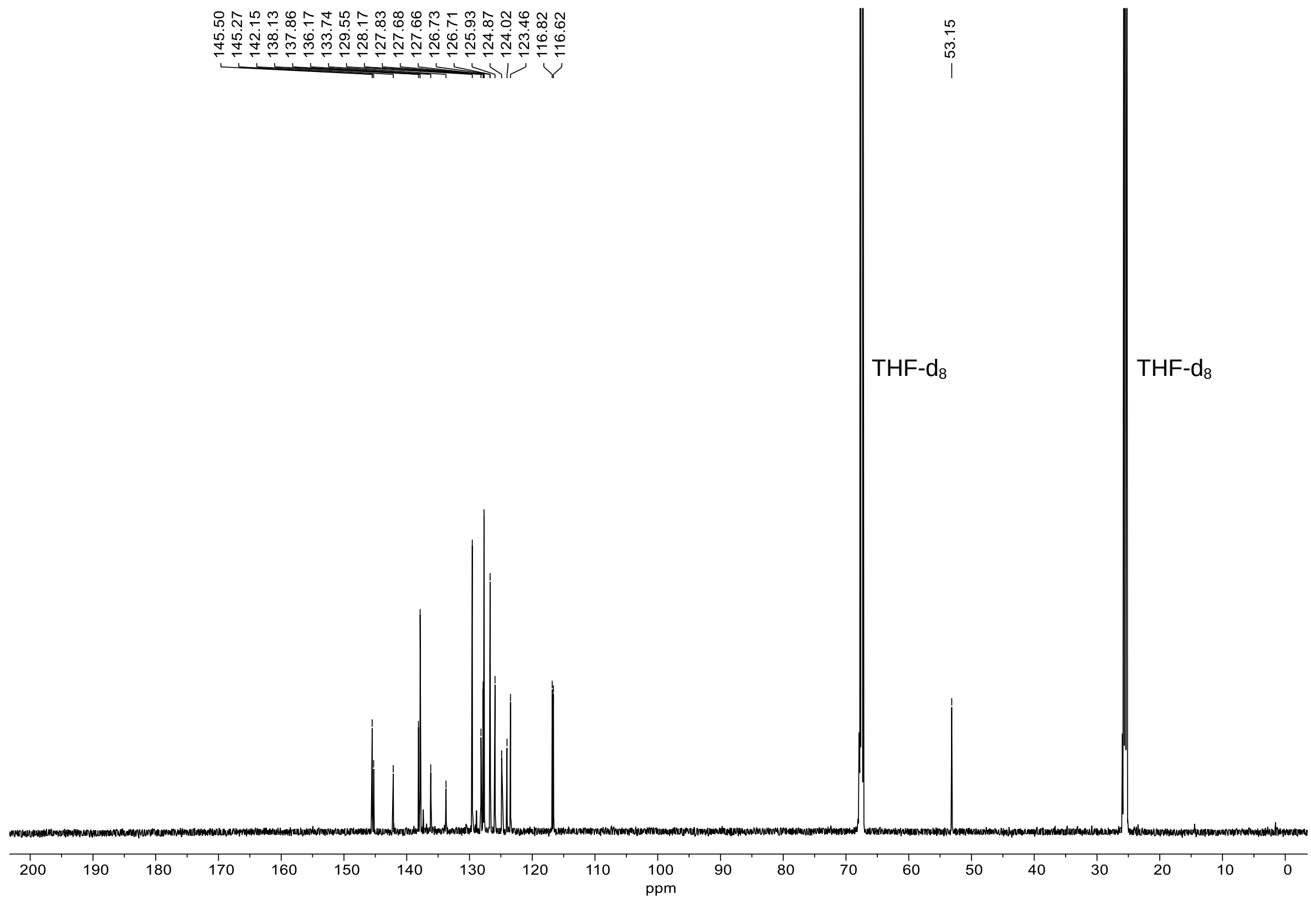

Figure S28. ^{13}C NMR spectrum (THF-d$_8$, 150 MHz, T = 298 K) of compound **5e**.

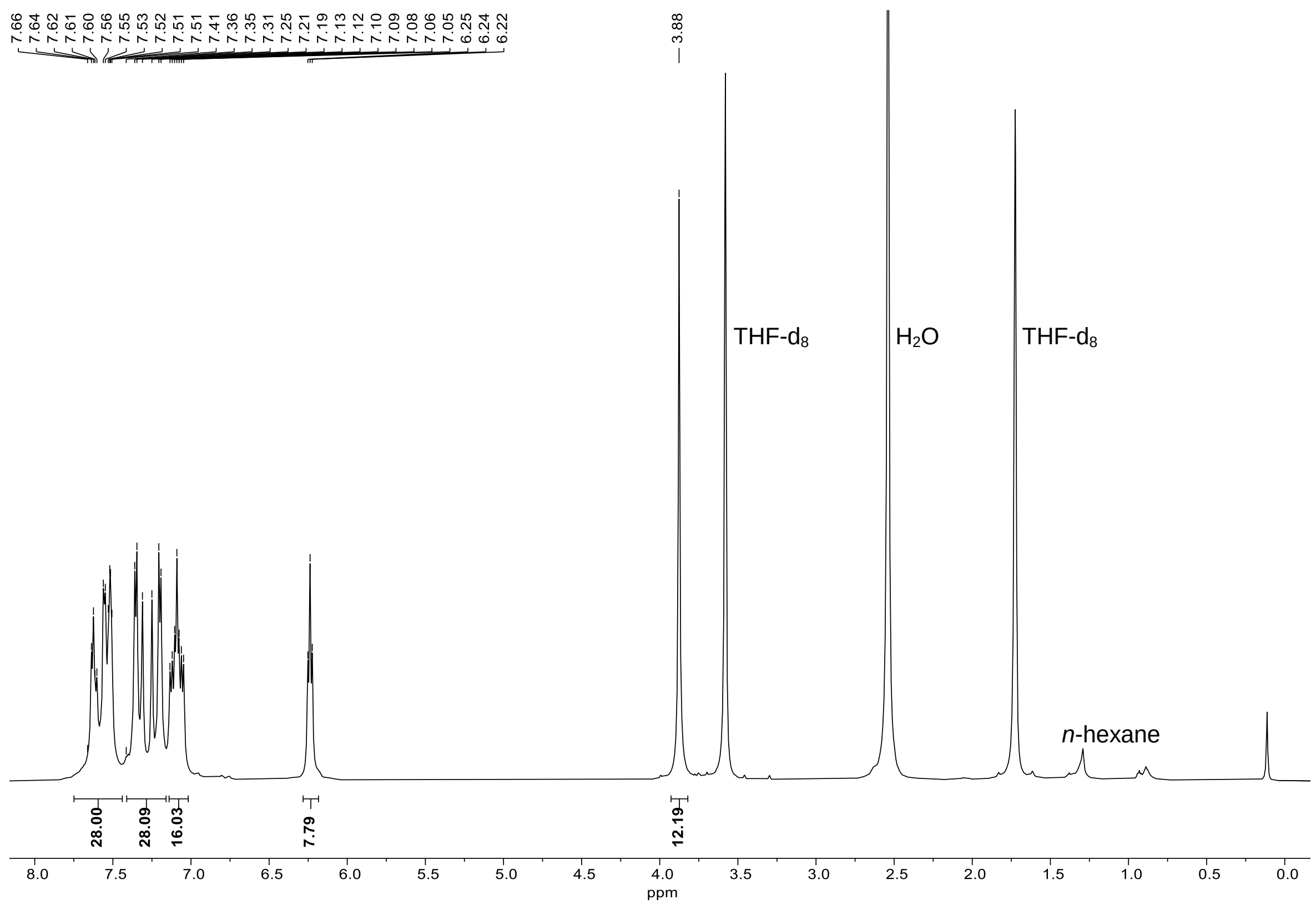

Figure S29. ^{1}H NMR spectrum (THF-d$_8$, 600 MHz, T = 298 K) of compound **5f**.

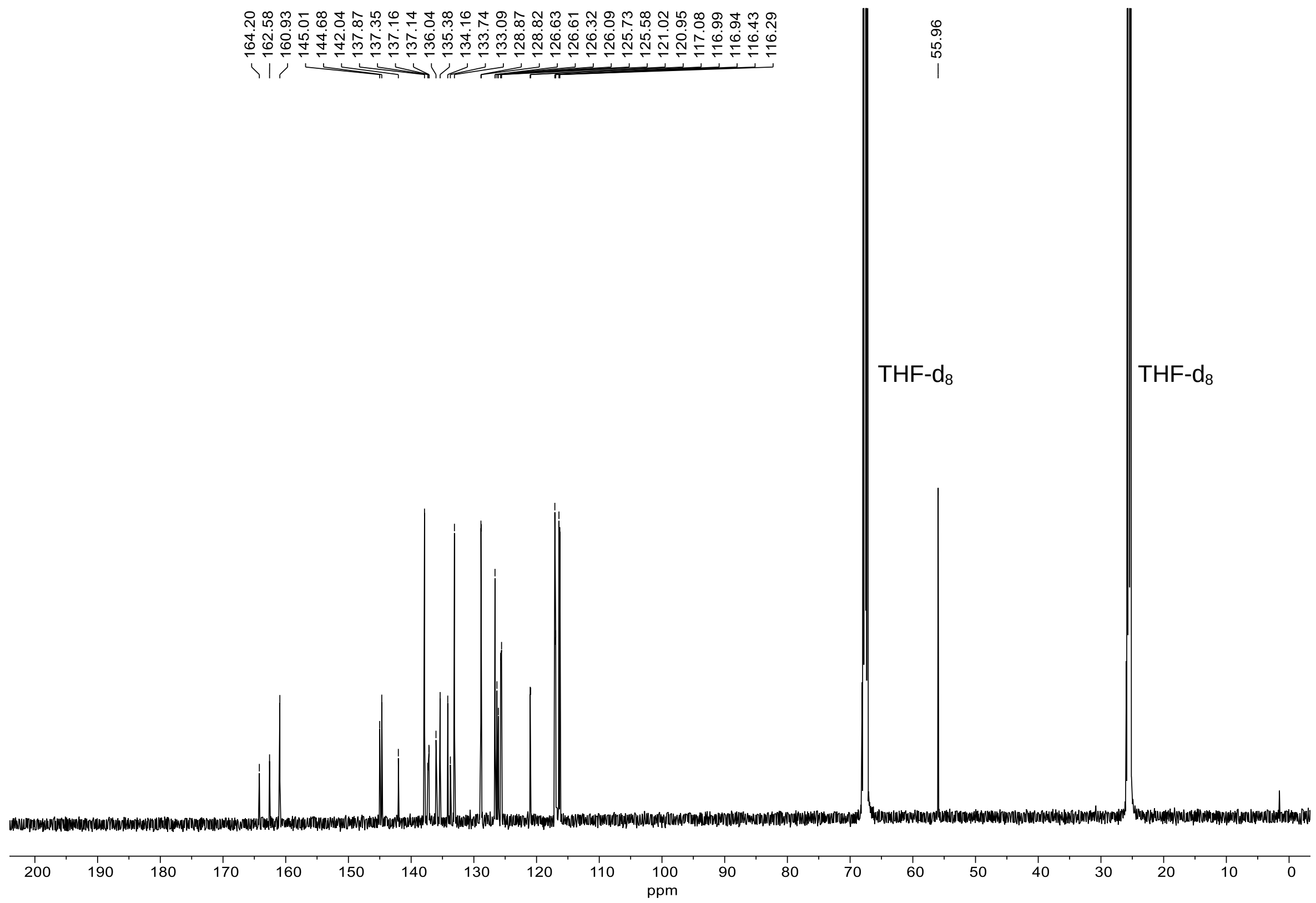

Figure S30. ^{13}C NMR spectrum (THF-d$_8$, 150 MHz, T = 298 K) of compound **5f**.

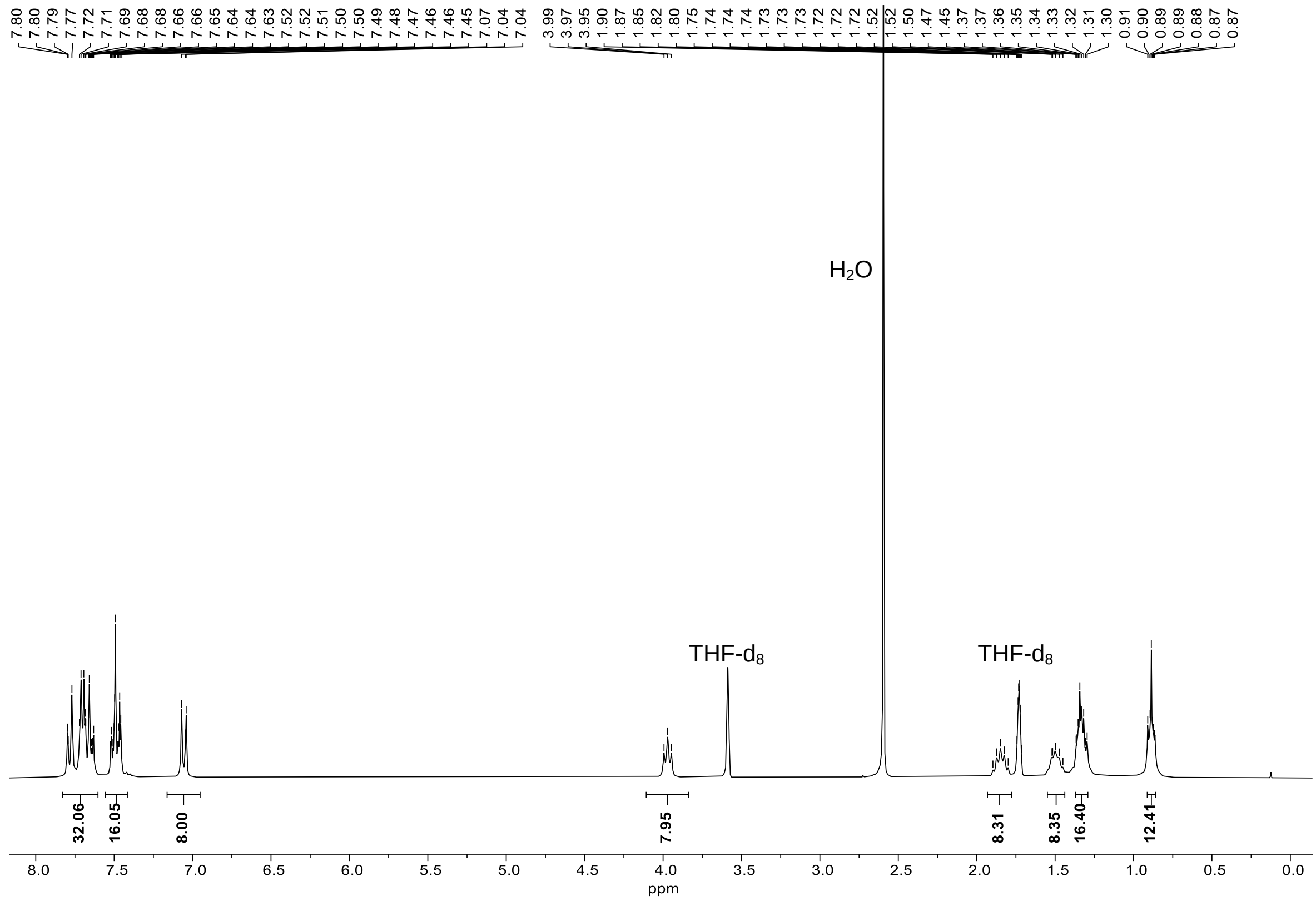

Figure S31. ^{1}H NMR spectrum (THF-d_8, 300 MHz, T = 298 K) of compound **5g**.

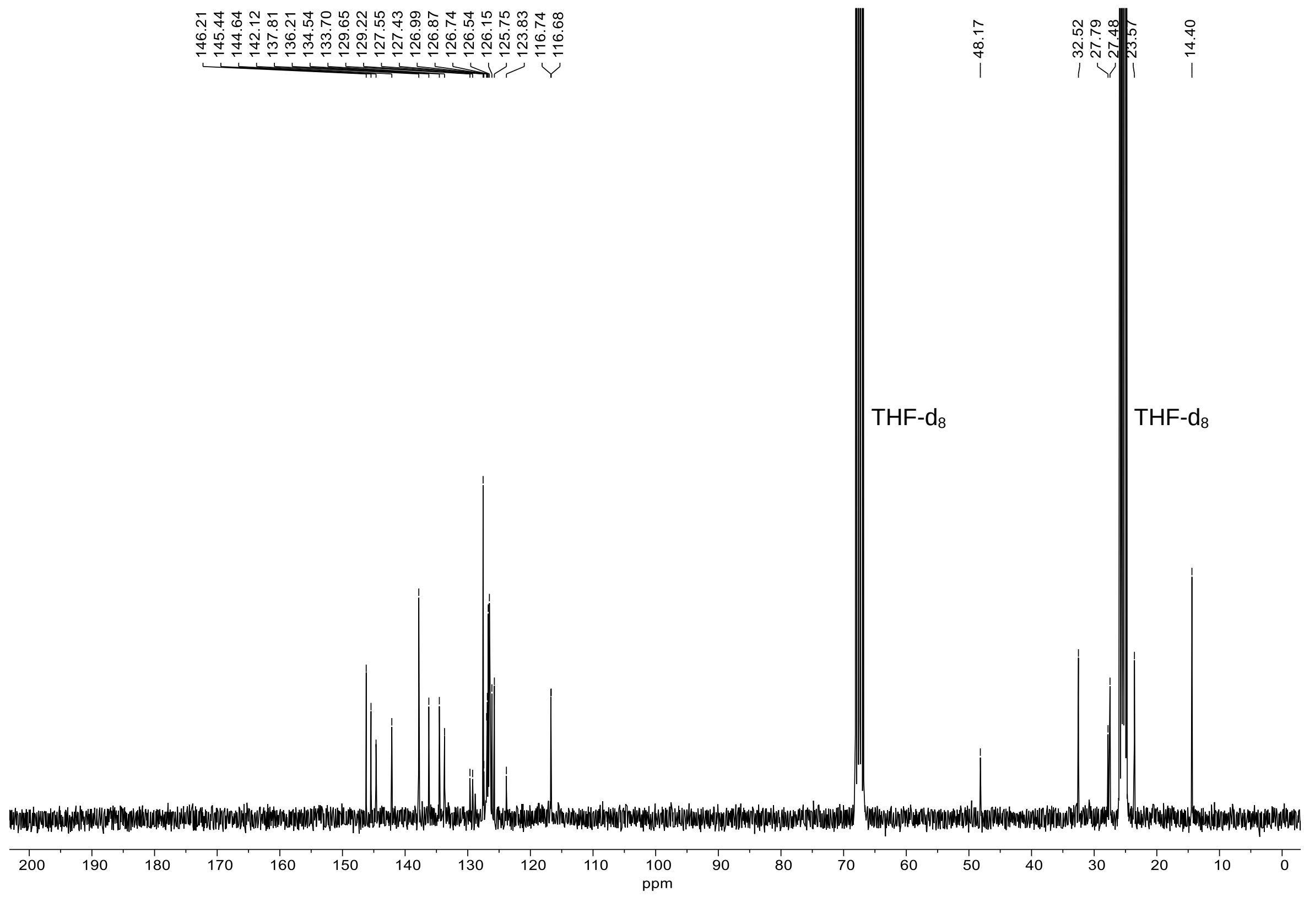

Figure S32. ^{13}C NMR spectrum (THF-d_8, 75 MHz, T = 298 K) of compound **5g**.

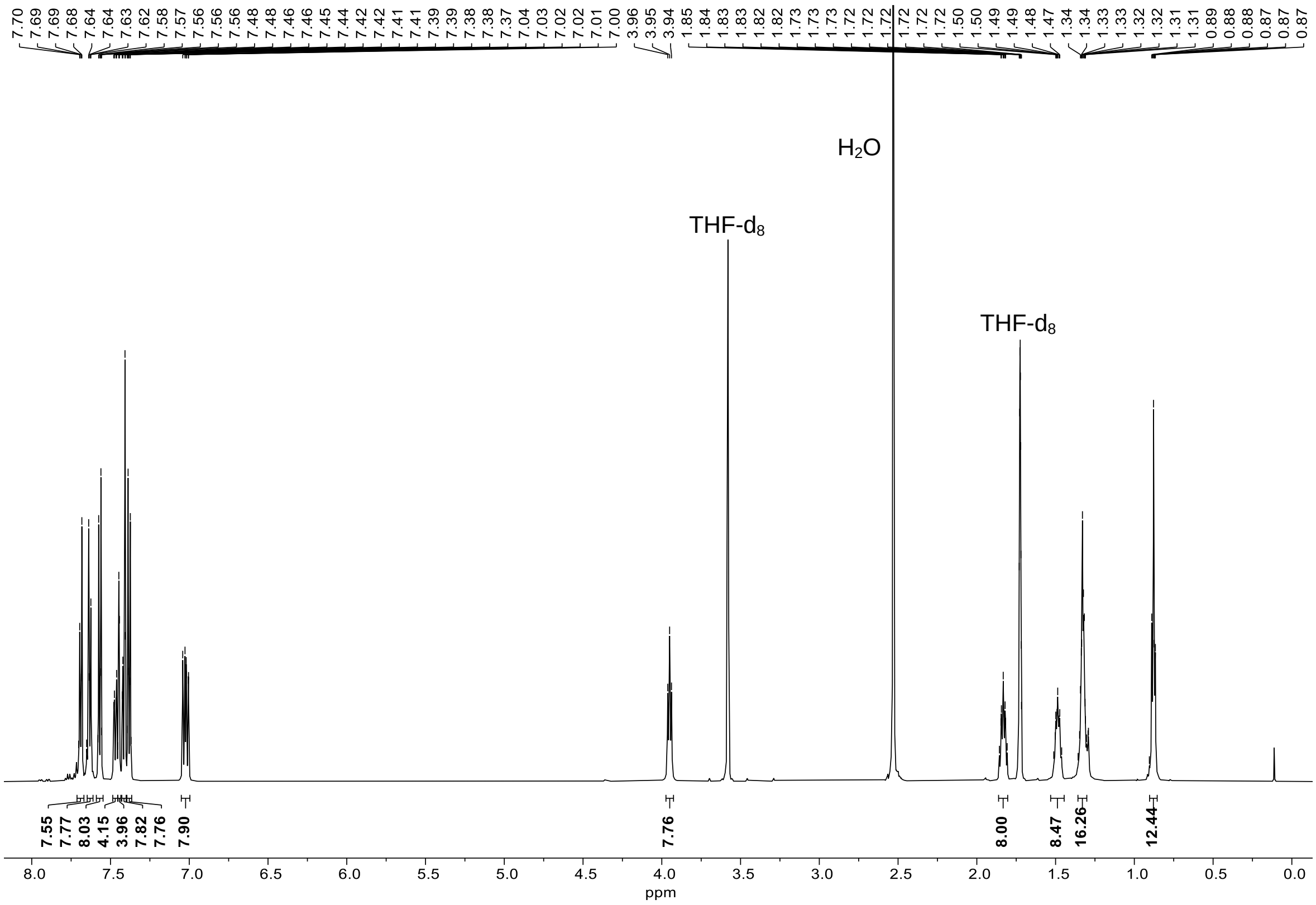

Figure S33. ^{1}H NMR spectrum (THF-d_8, 600 MHz, T = 298 K) of compound **5h**.

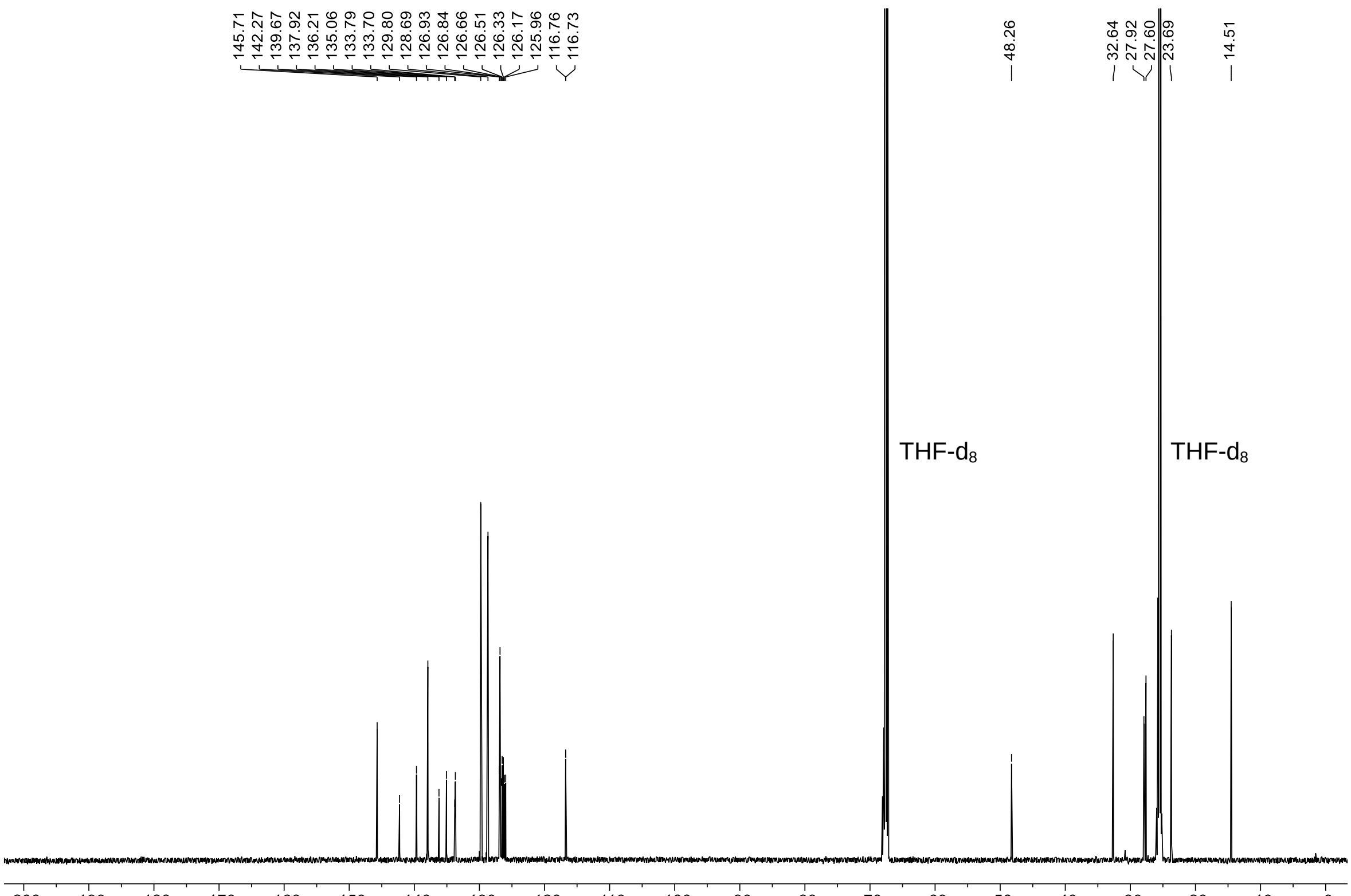

Figure S34. ^{13}C NMR spectrum (THF-d_8, 150 MHz, T = 298 K) of compound **5h**.

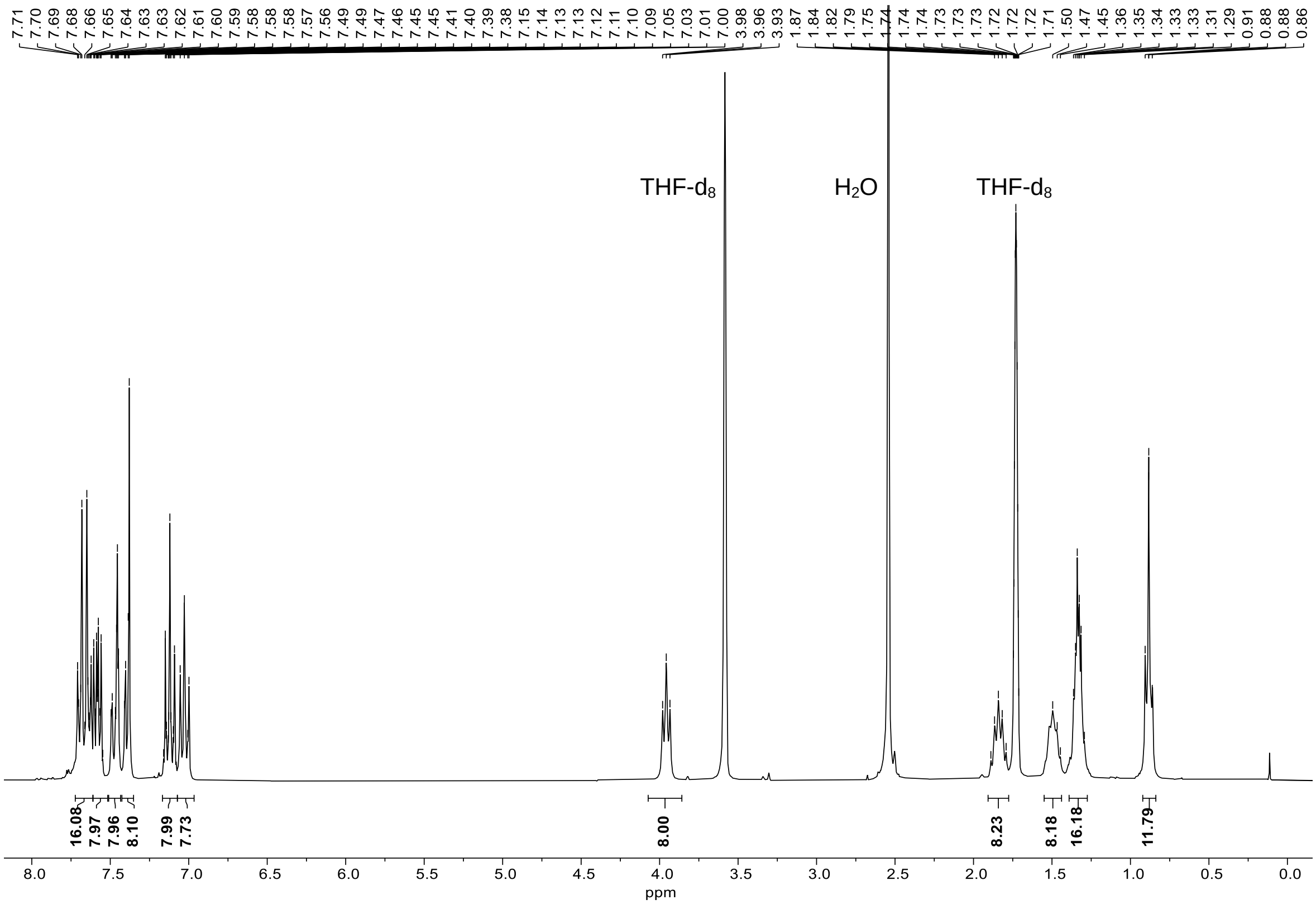

Figure S35. ^{1}H NMR spectrum (THF-d$_8$, 300 MHz, T = 298 K) of compound **5i**.

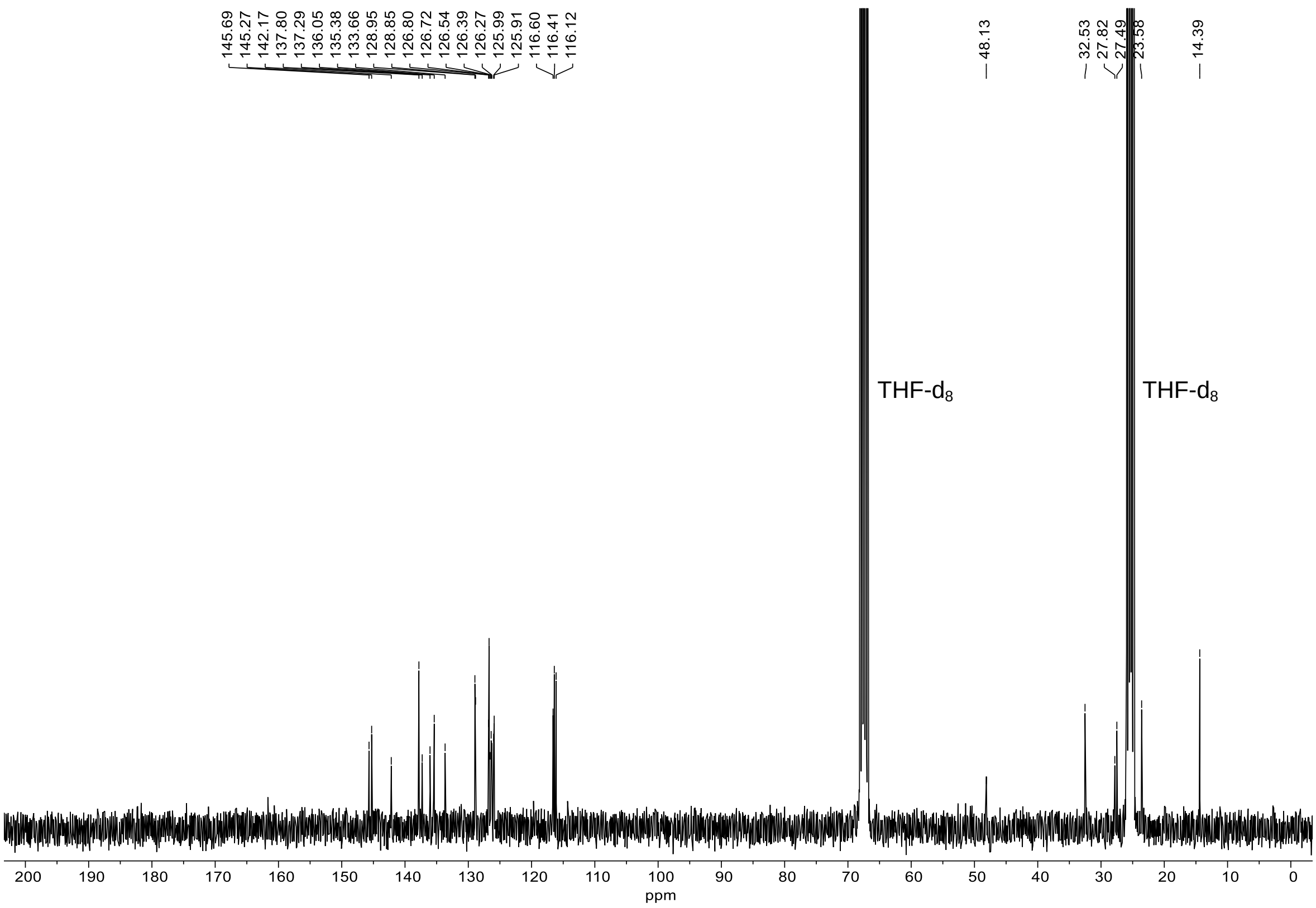

Figure S36. ^{13}C NMR spectrum (THF-d$_8$, 75 MHz, T = 298 K) of compound **5i**.

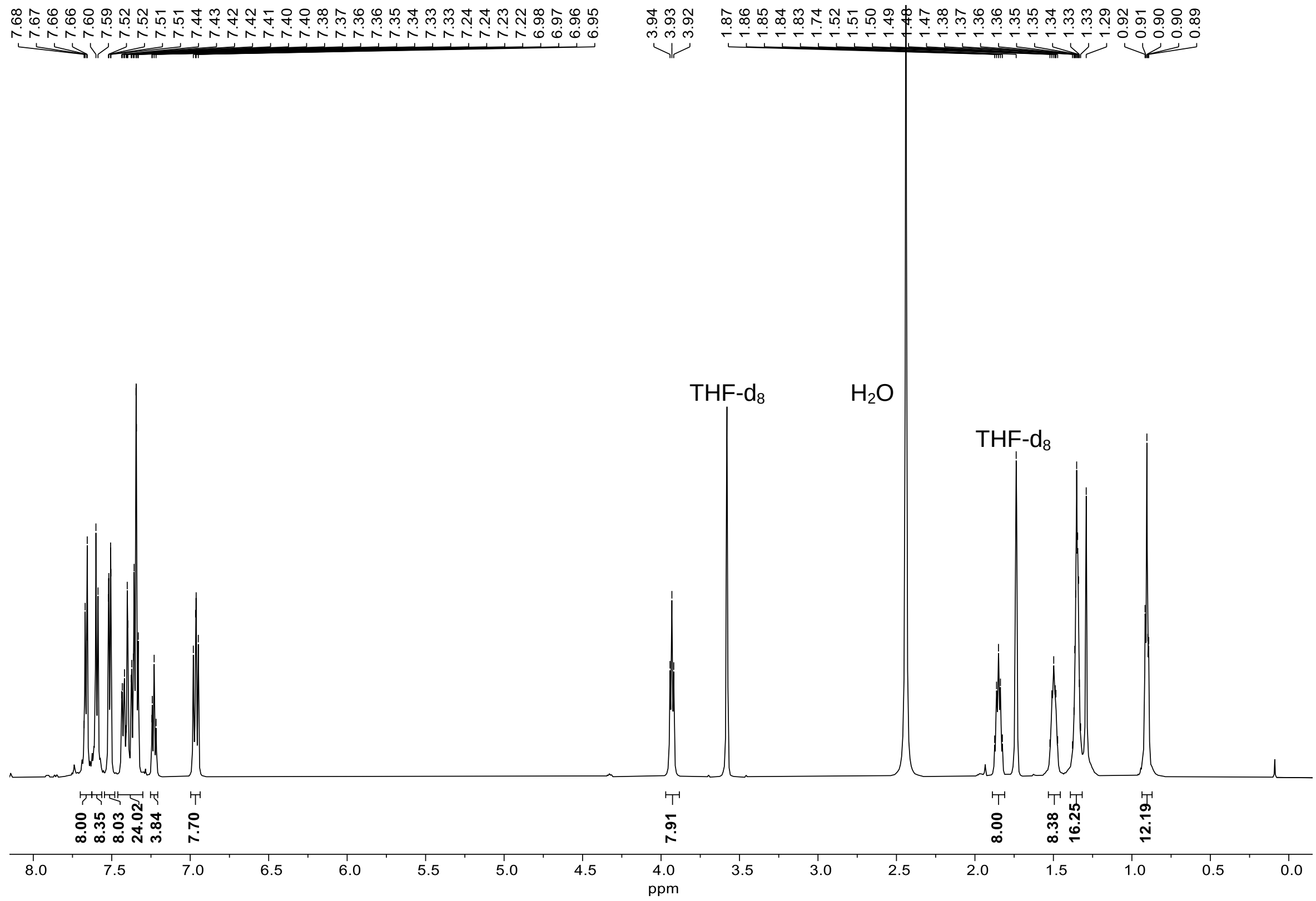

Figure S37. ^{1}H NMR spectrum (THF-d_8, 600 MHz, T = 298 K) of compound **5j**.

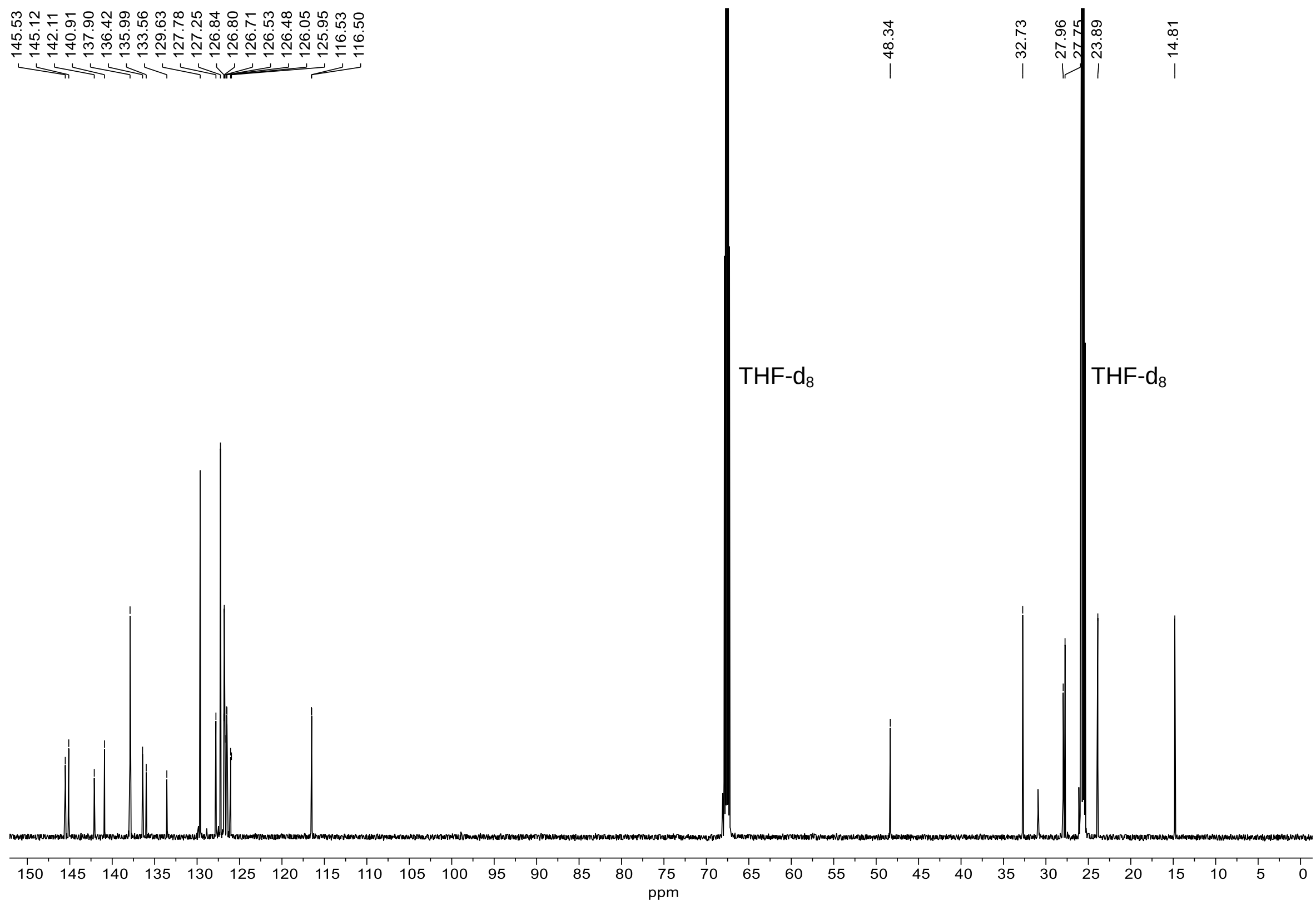

Figure S38. ^{13}C NMR spectrum (THF-d_8, 150 MHz, T = 298 K) of compound **5j**.

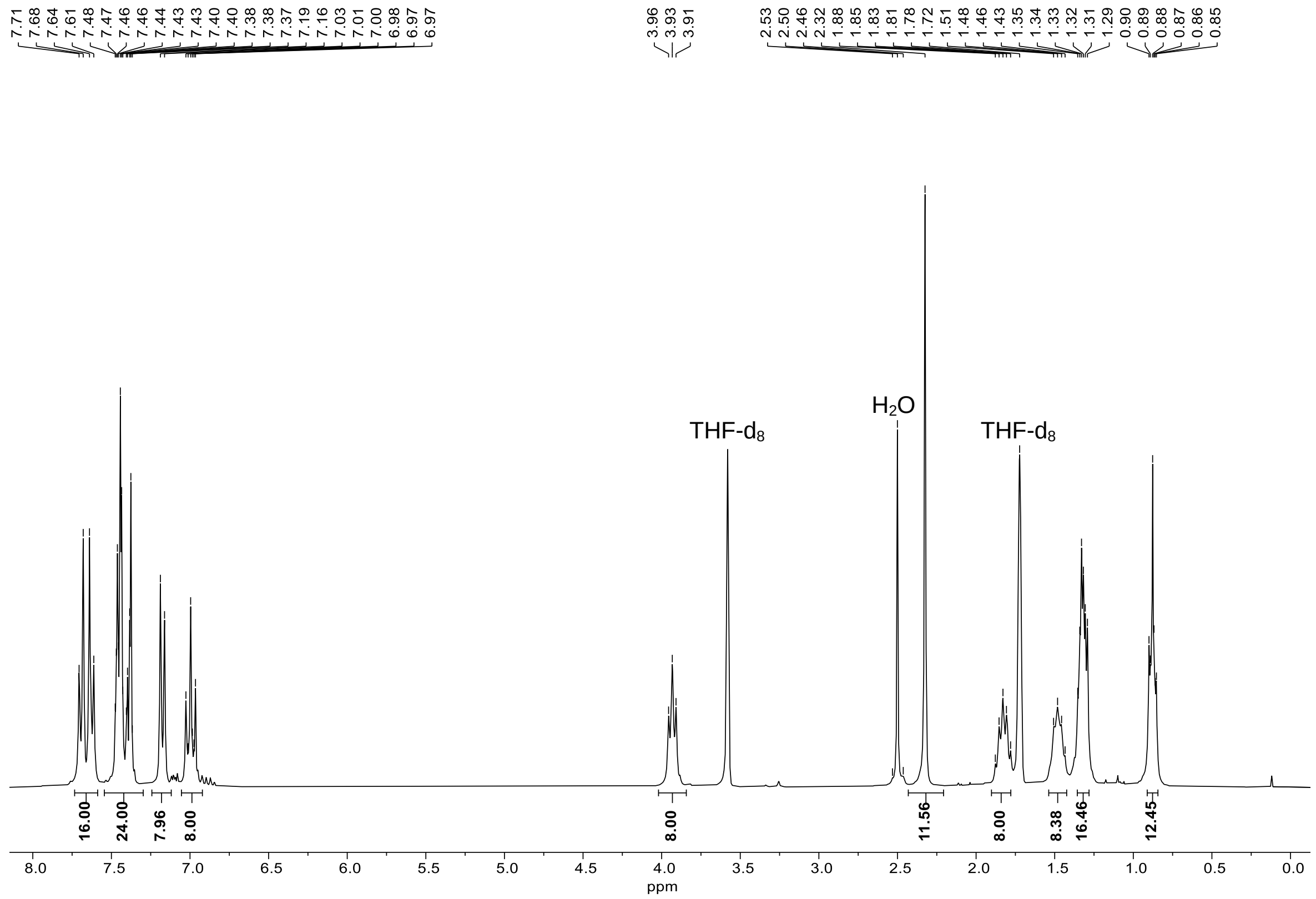

Figure S39. ^{1}H NMR spectrum (THF-d_8, 300 MHz, T = 298 K) of compound **5k**.

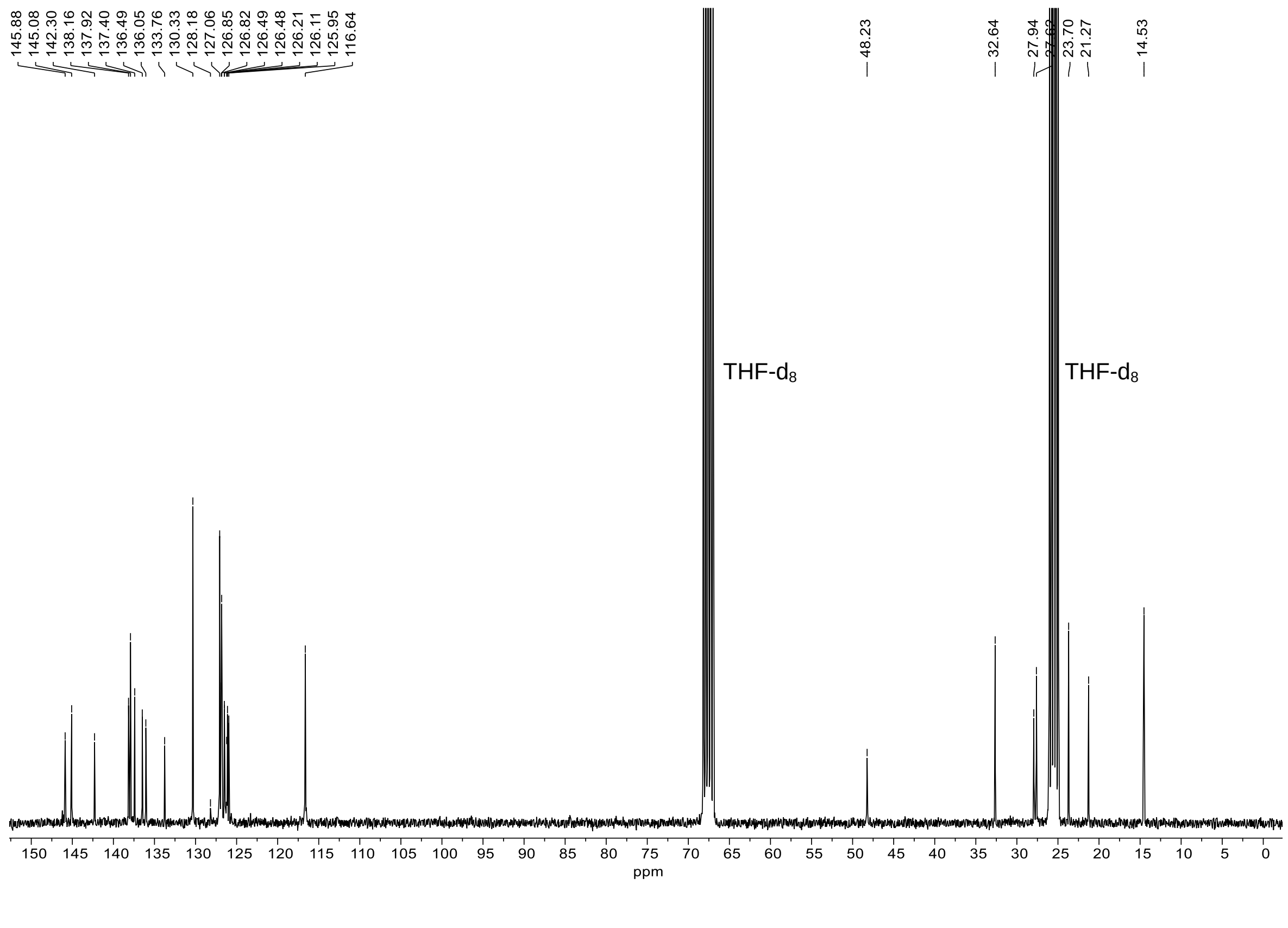

Figure S40. ^{13}C NMR spectrum (THF-d_8, 75 MHz, T = 298 K) of compound **5k**.

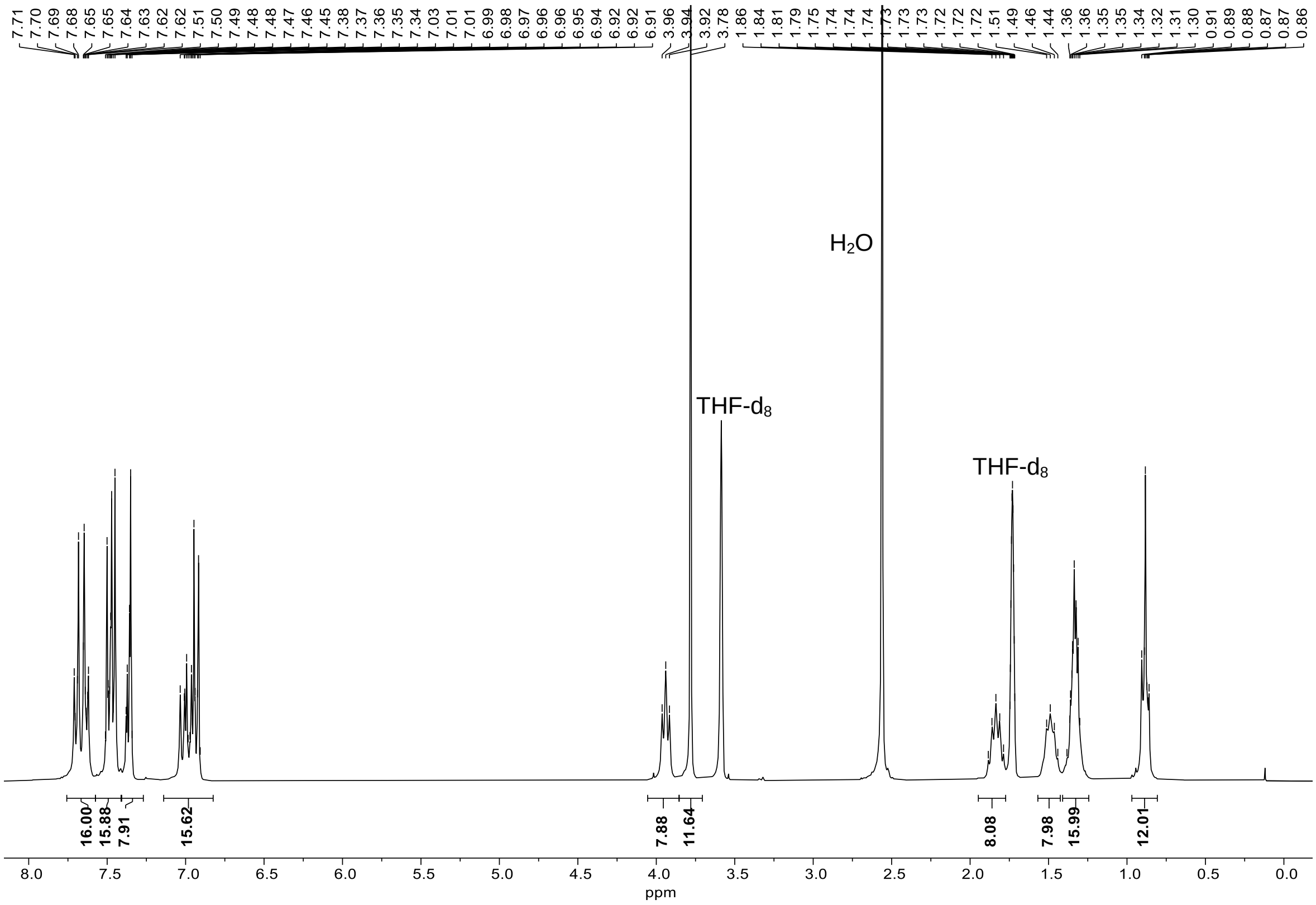

Figure S41. ^{1}H NMR spectrum (THF-d_8, 300 MHz, T = 298 K) of compound **5l**.

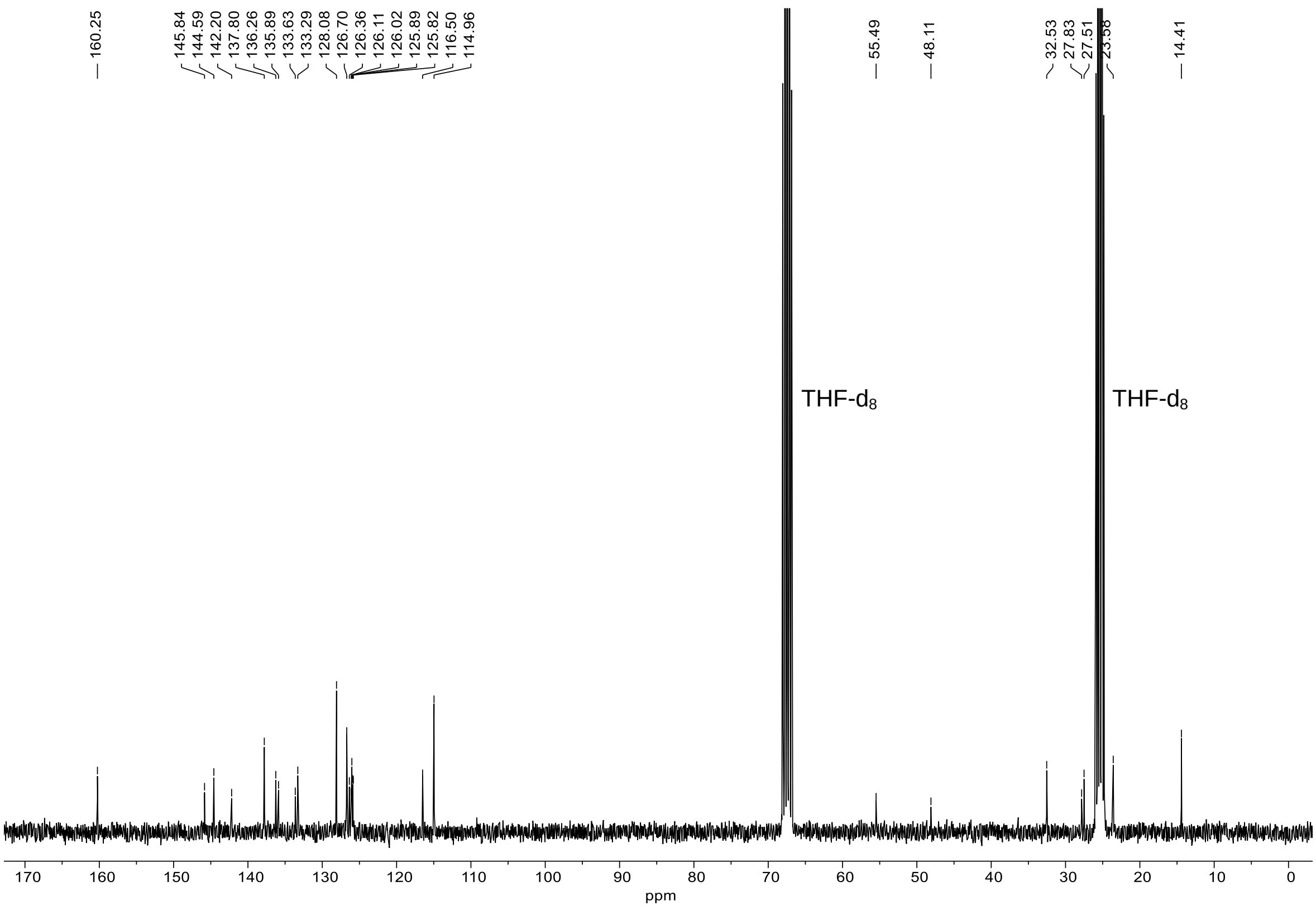

Figure S42. ^{13}C NMR spectrum (THF-d_8, 75 MHz, T = 298 K) of compound **5l**.

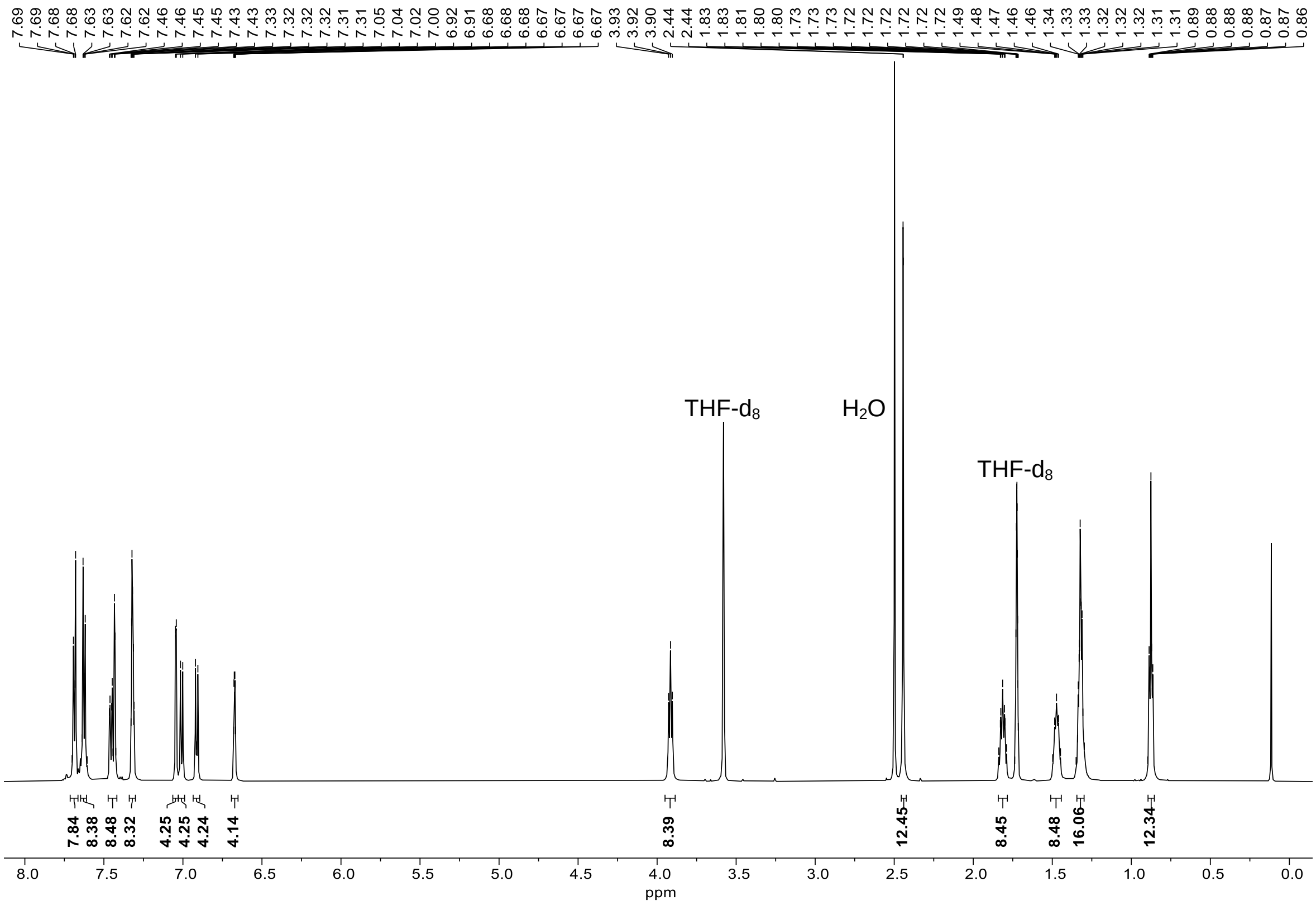

Figure S43. ^{1}H NMR spectrum (THF-d$_8$, 600 MHz, *T* = 298 K) of compound **5m**.

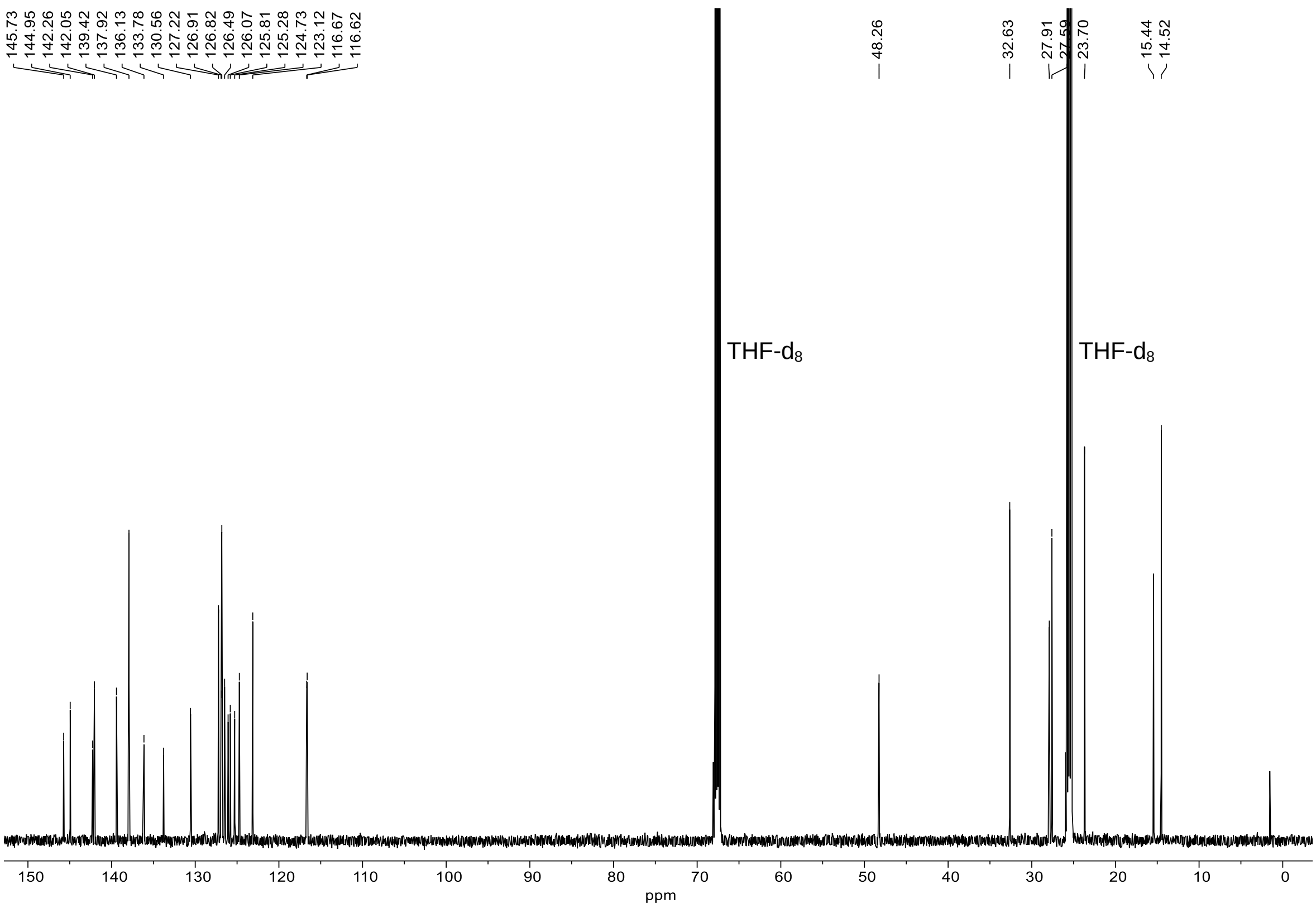

Figure S44. ^{13}C NMR spectrum (THF-d$_8$, 150 MHz, *T* = 298 K) of compound **5m**.

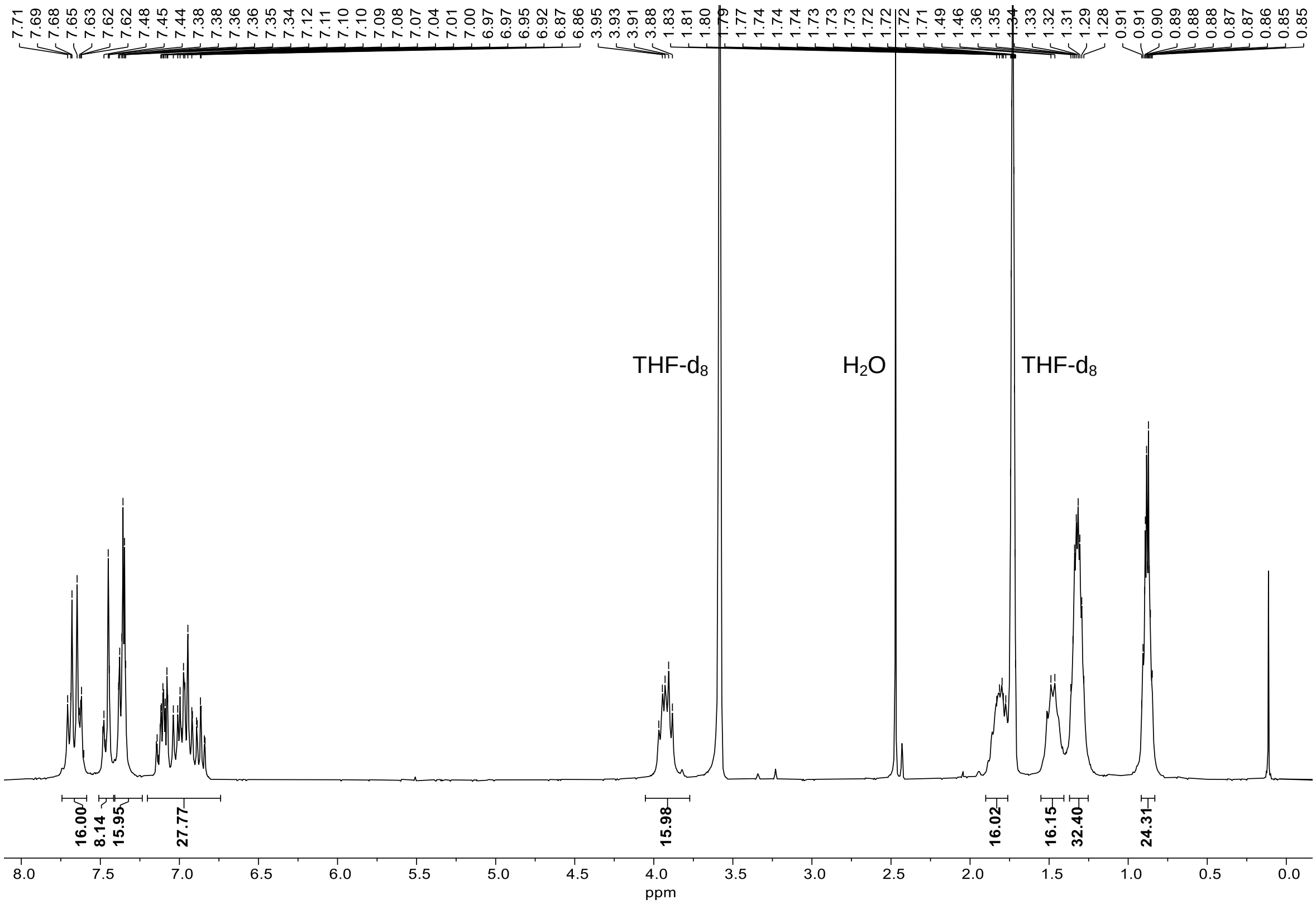

Figure S45. ^{1}H NMR spectrum (THF-d$_8$, 600 MHz, T = 298 K) of compound **5n**.

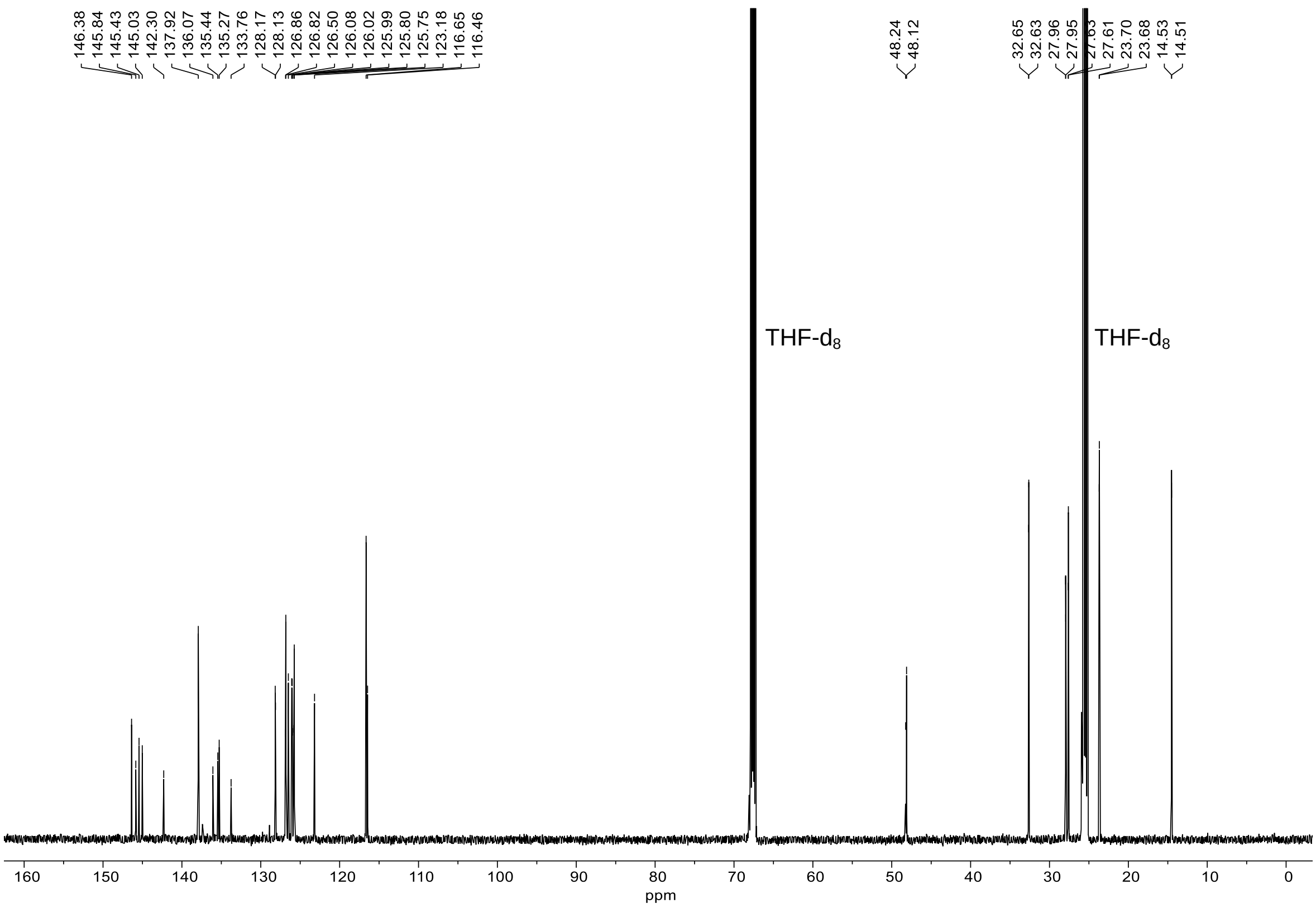

Figure S46. ^{13}C NMR spectrum (THF-d$_8$, 150 MHz, T = 298 K) of compound **5n**.

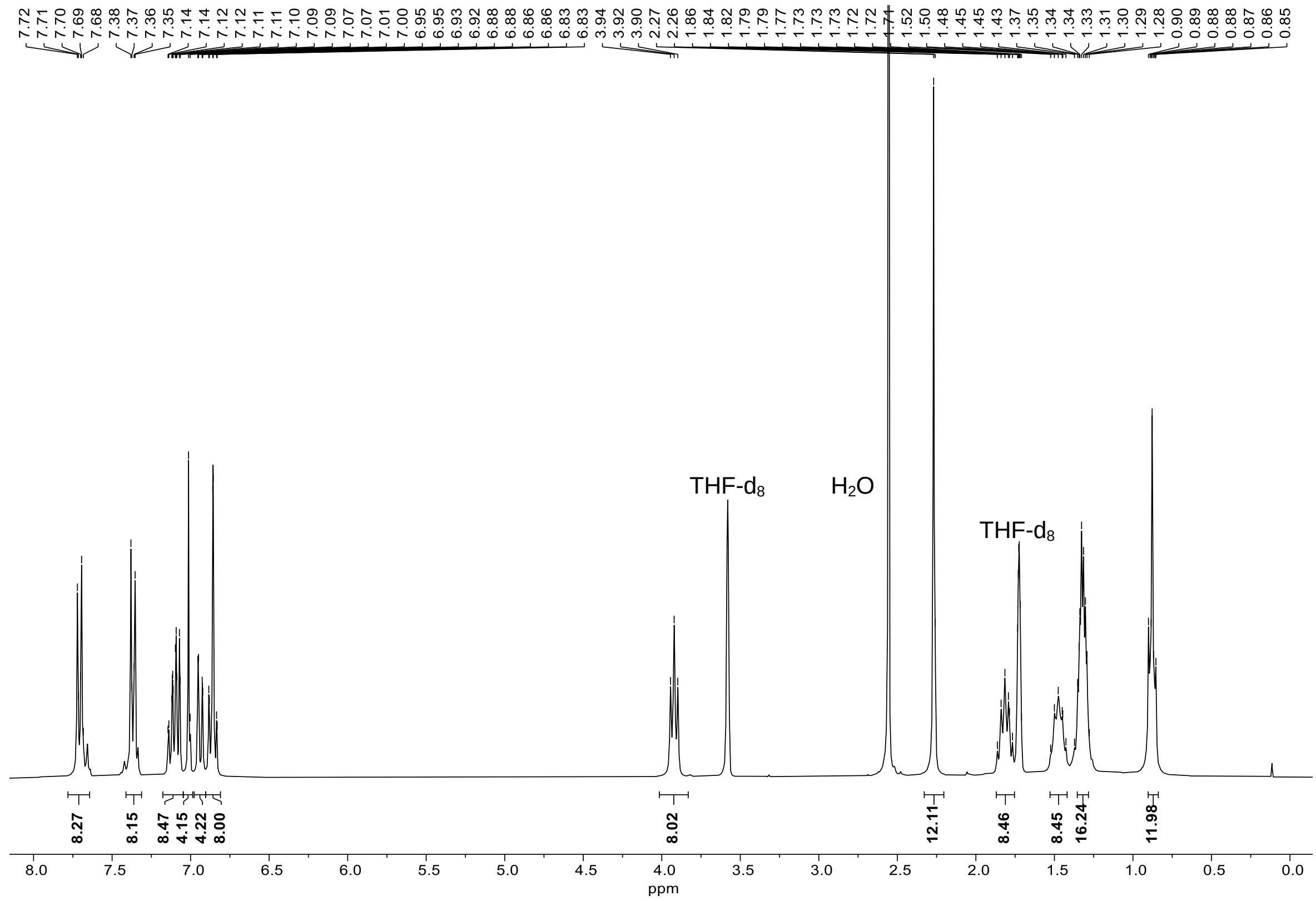

Figure S47. ^{1}H NMR spectrum (THF-d$_8$, 300 MHz, T = 298 K) of compound **5o**.

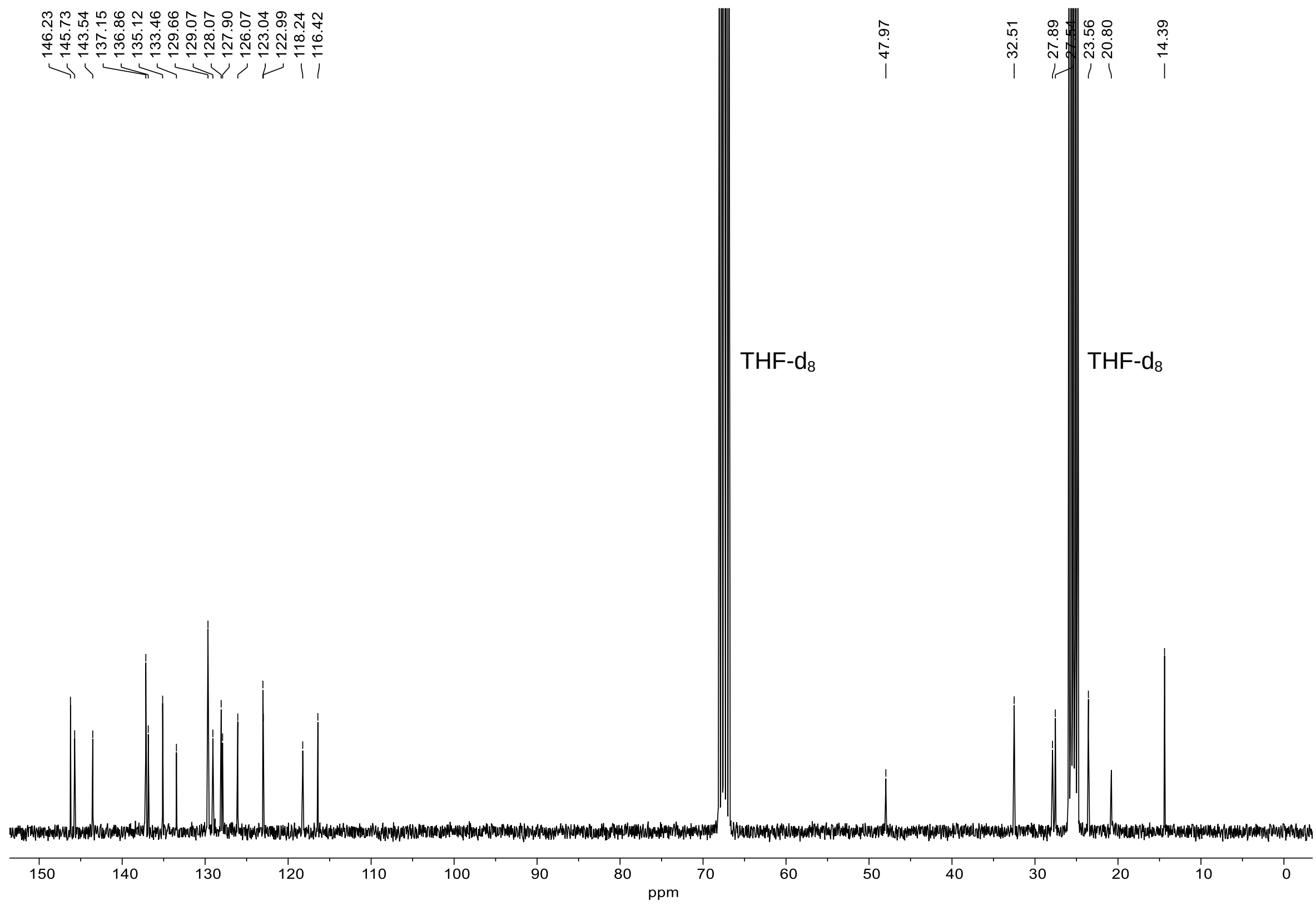

Figure S48. ^{13}C NMR spectrum (THF-d$_8$, 75 MHz, T = 298 K) of compound **5o**.

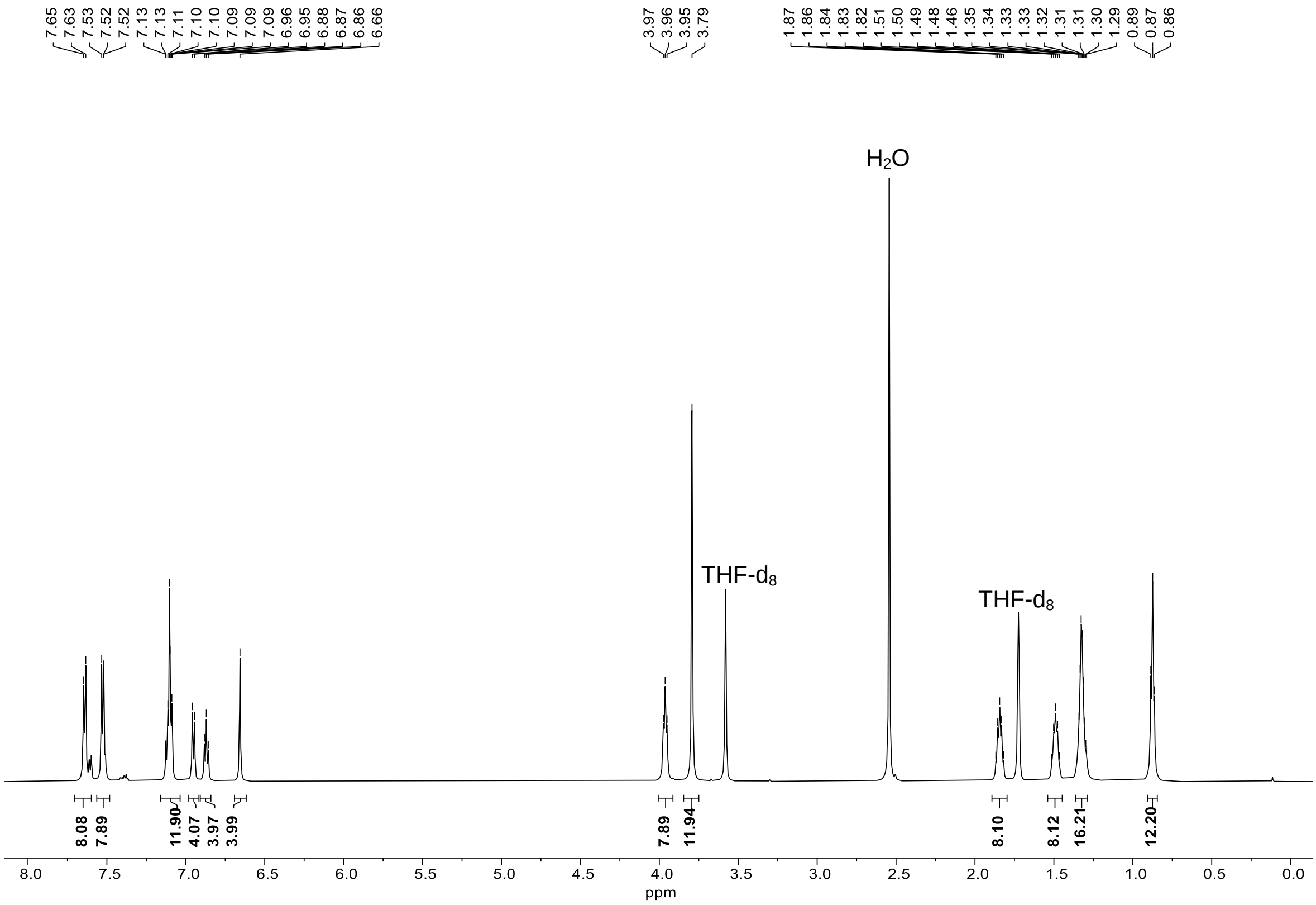

Figure S49. ^{1}H NMR spectrum (THF-d$_8$, 600 MHz, T = 298 K) of compound **5p**.

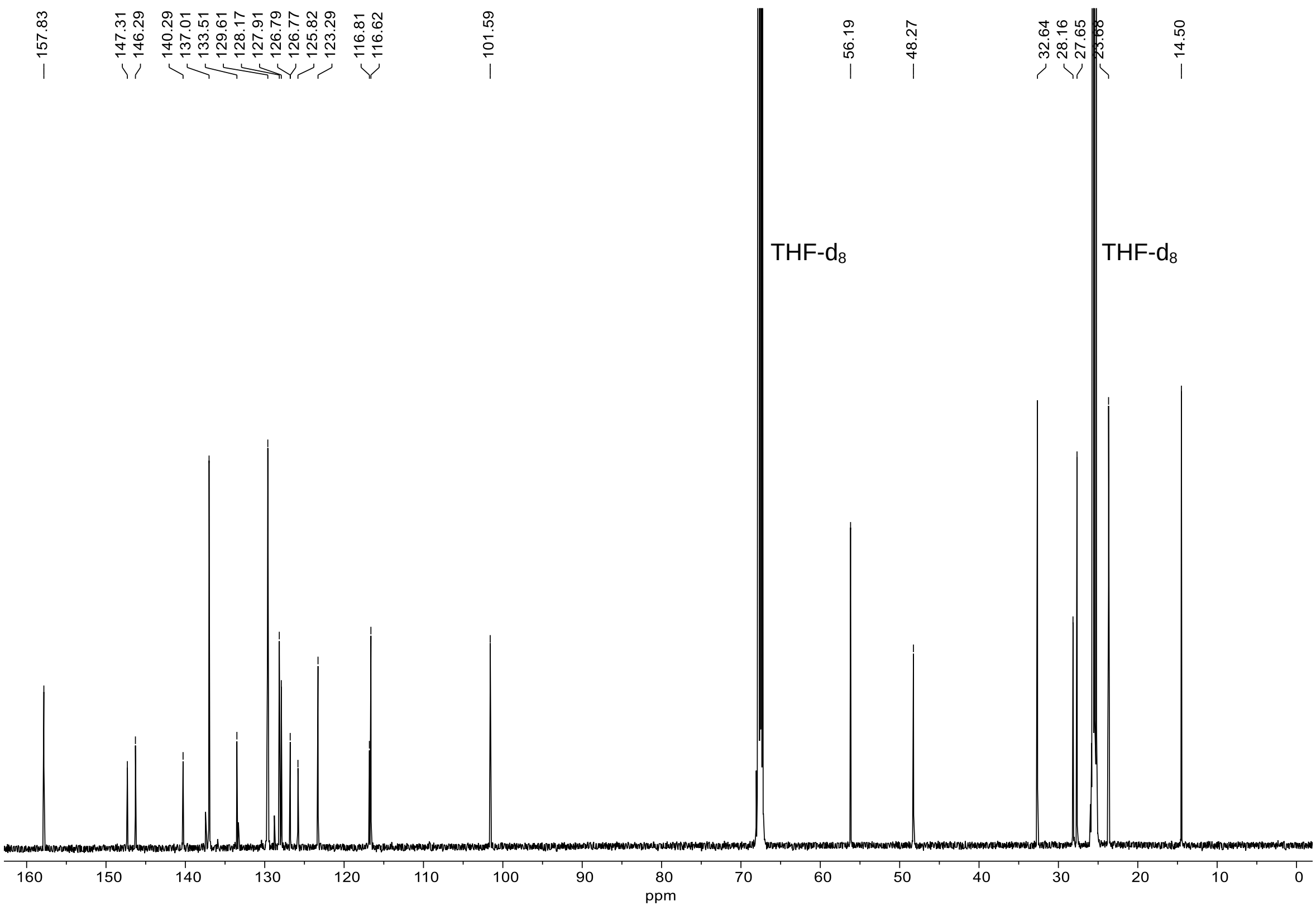

Figure S50. ^{13}C NMR spectrum (THF-d$_8$, 150 MHz, T = 298 K) of compound **5p**.

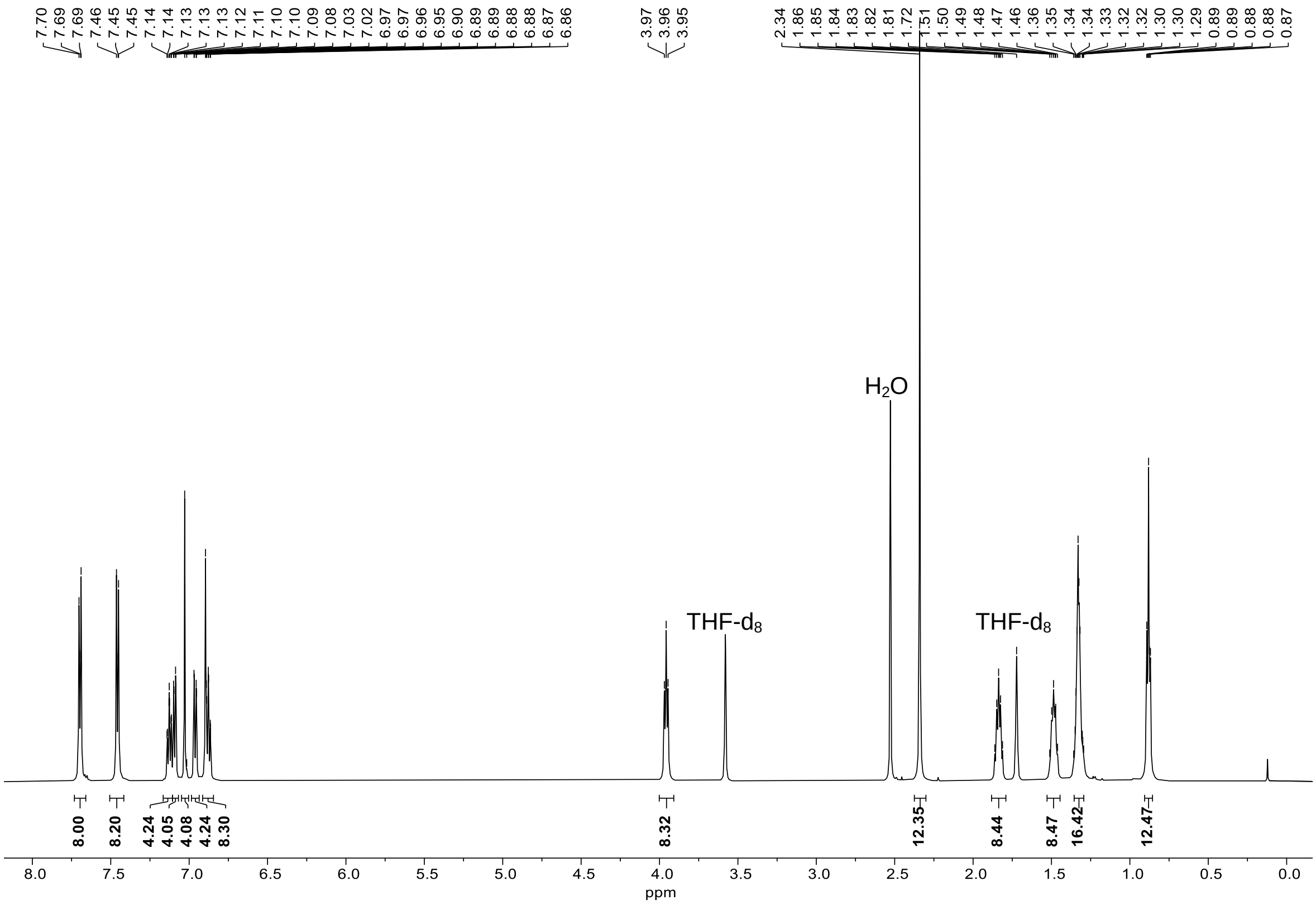

Figure S51. ^{1}H NMR spectrum (THF-d_8, 600 MHz, T = 298 K) of compound **5q**.

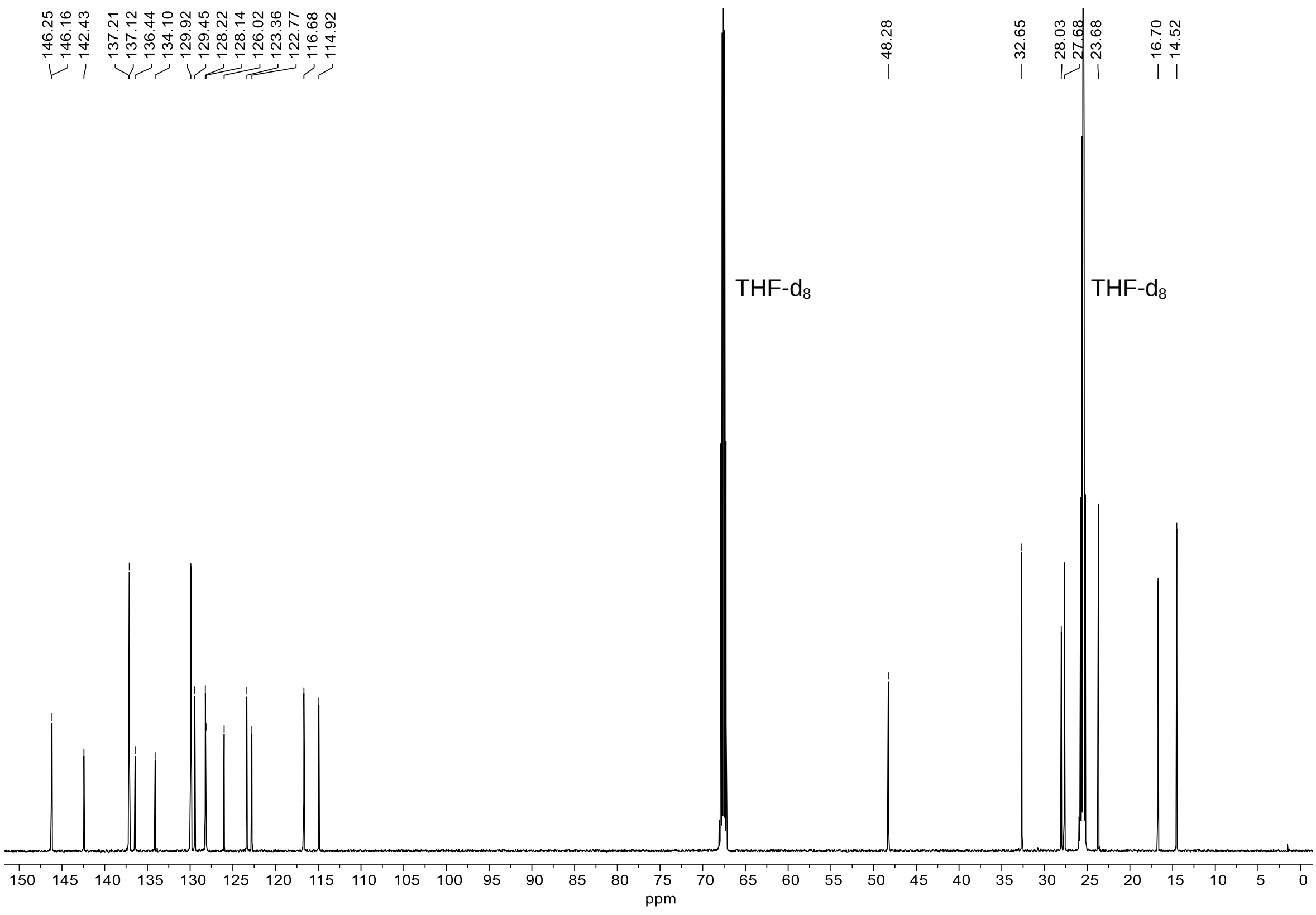

Figure S52. ^{13}C NMR spectrum (THF-d_8, 150 MHz, T = 298 K) of compound **5q**.

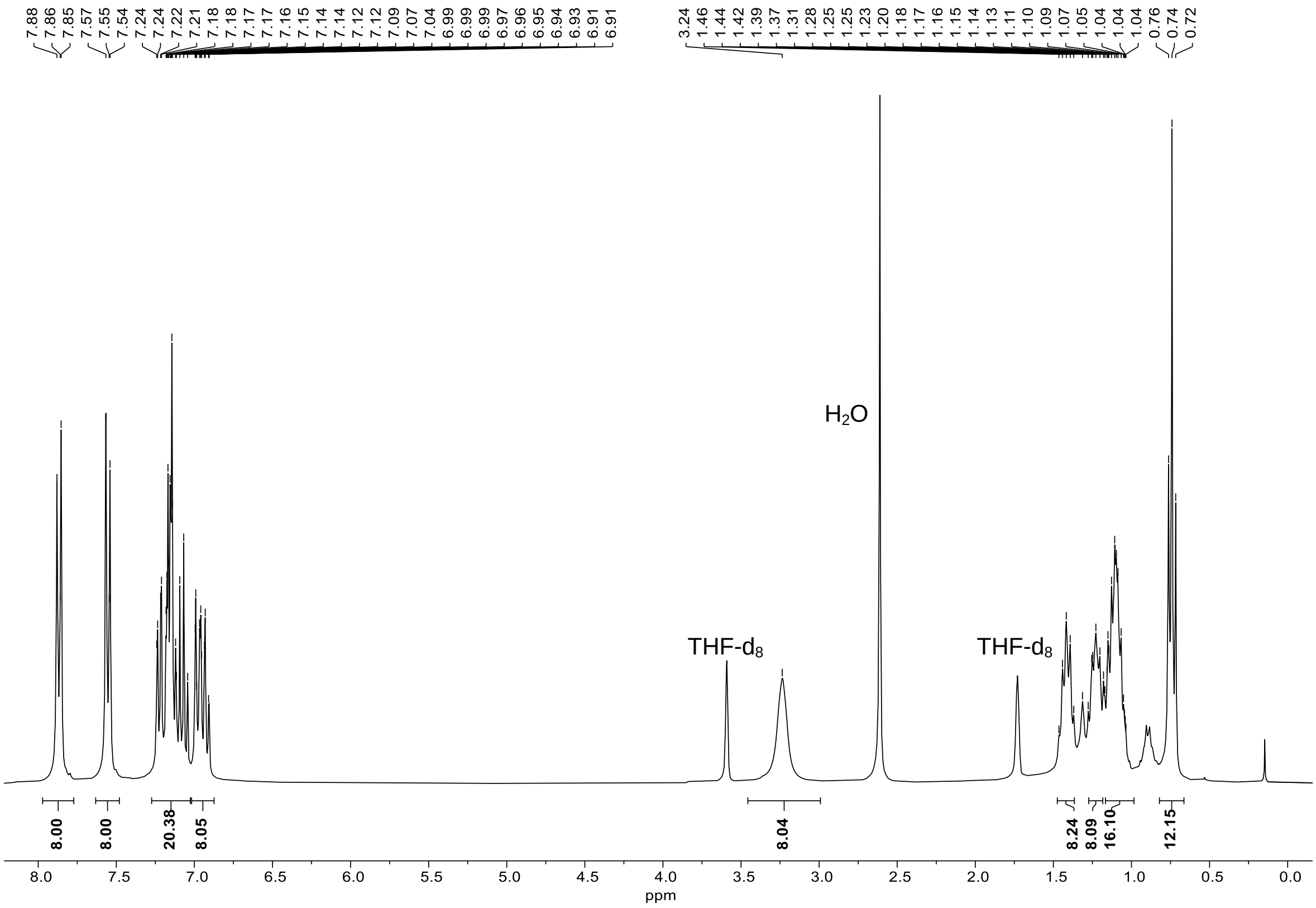

Figure S53. ^{1}H NMR spectrum (THF-d$_8$, 300 MHz, T = 298 K) of compound **5r**.

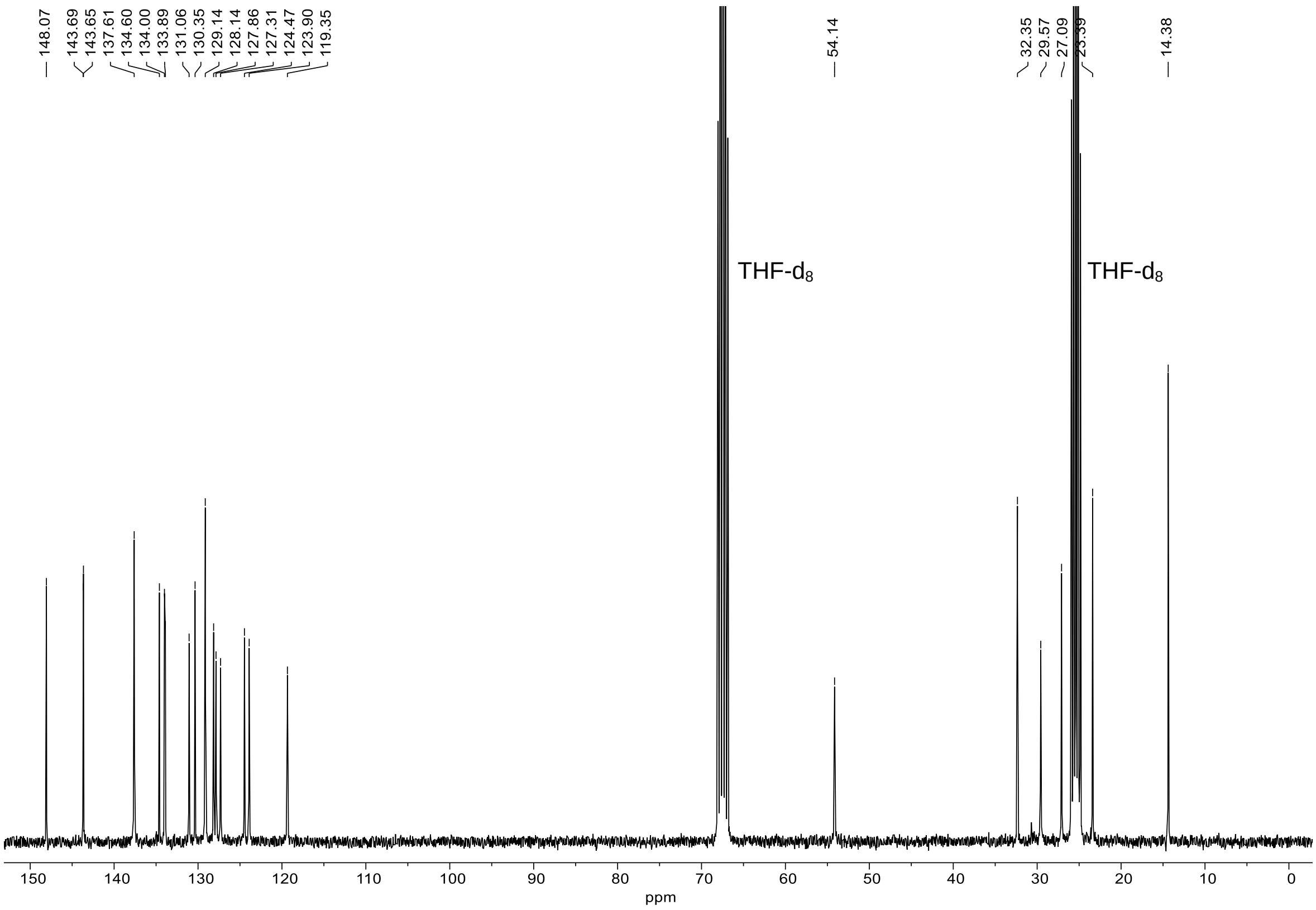

Figure S54. ^{13}C NMR spectrum (THF-d$_8$, 75 MHz, T = 298 K) of compound **5r**.

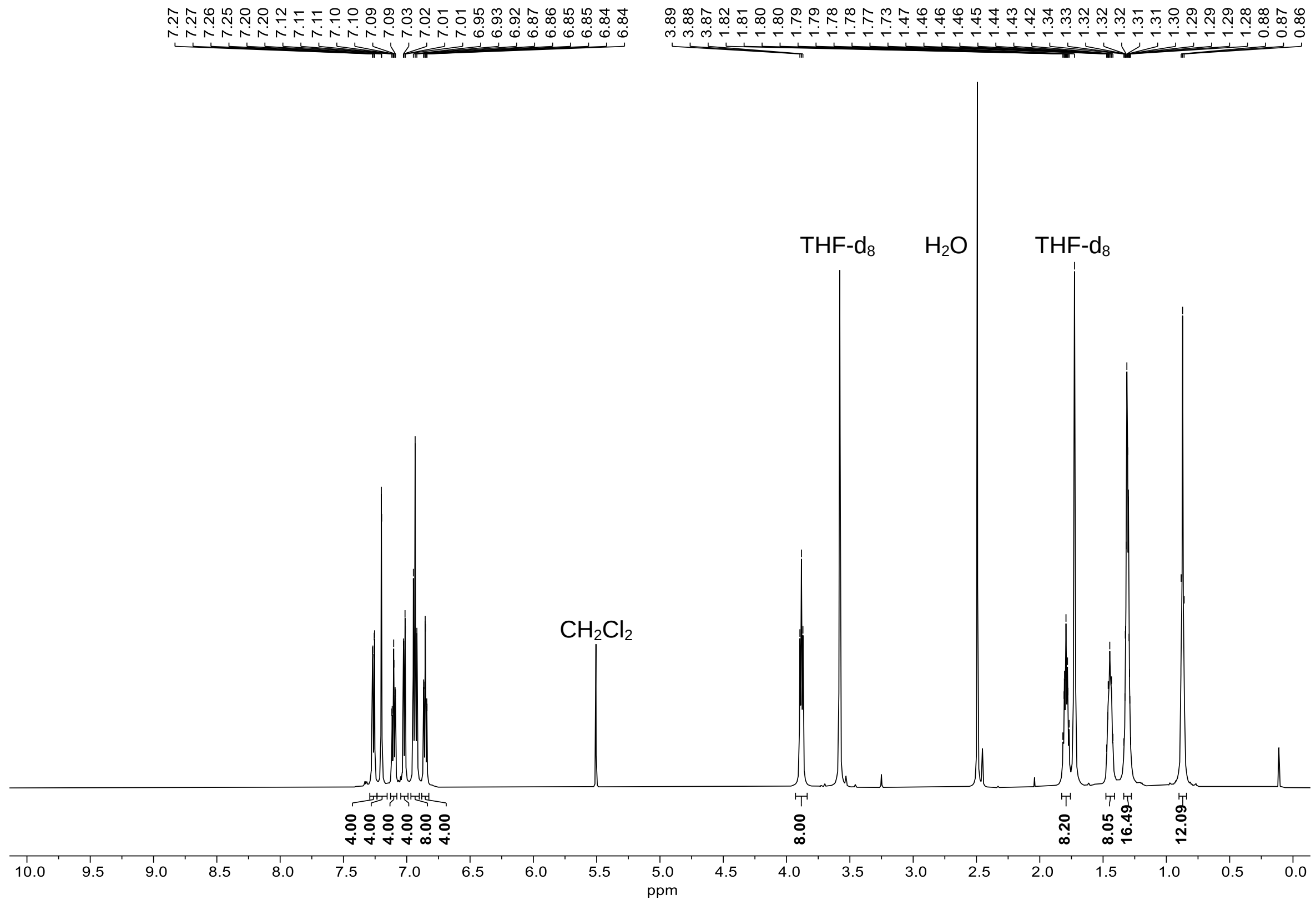

Figure S55. ^{1}H NMR spectrum (THF-d_8, 600 MHz, *T* = 298 K) of compound **6**.

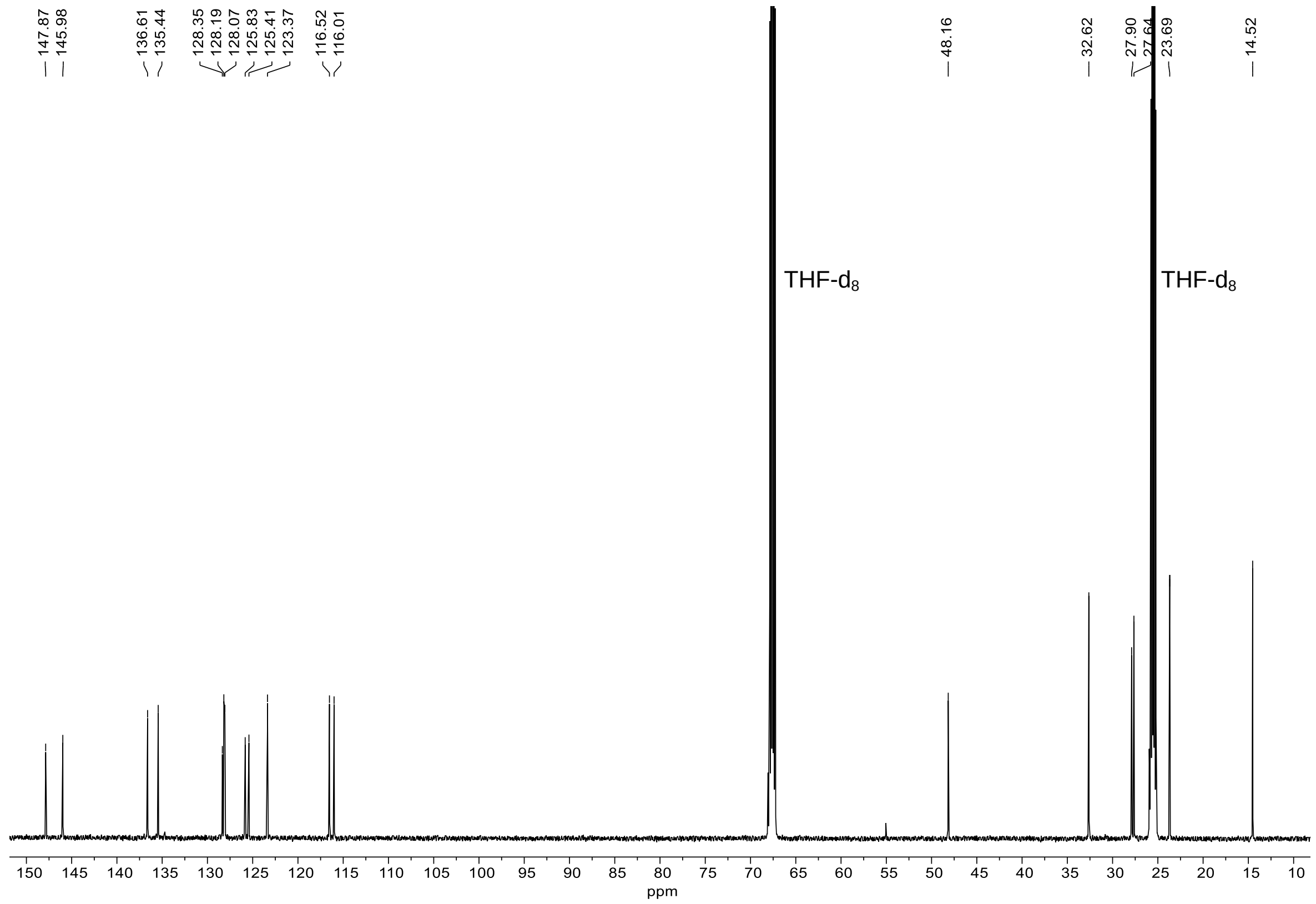

Figure S56. ^{13}C NMR spectrum (THF-d_8, 150 MHz, *T* = 298 K) of compound **6**.

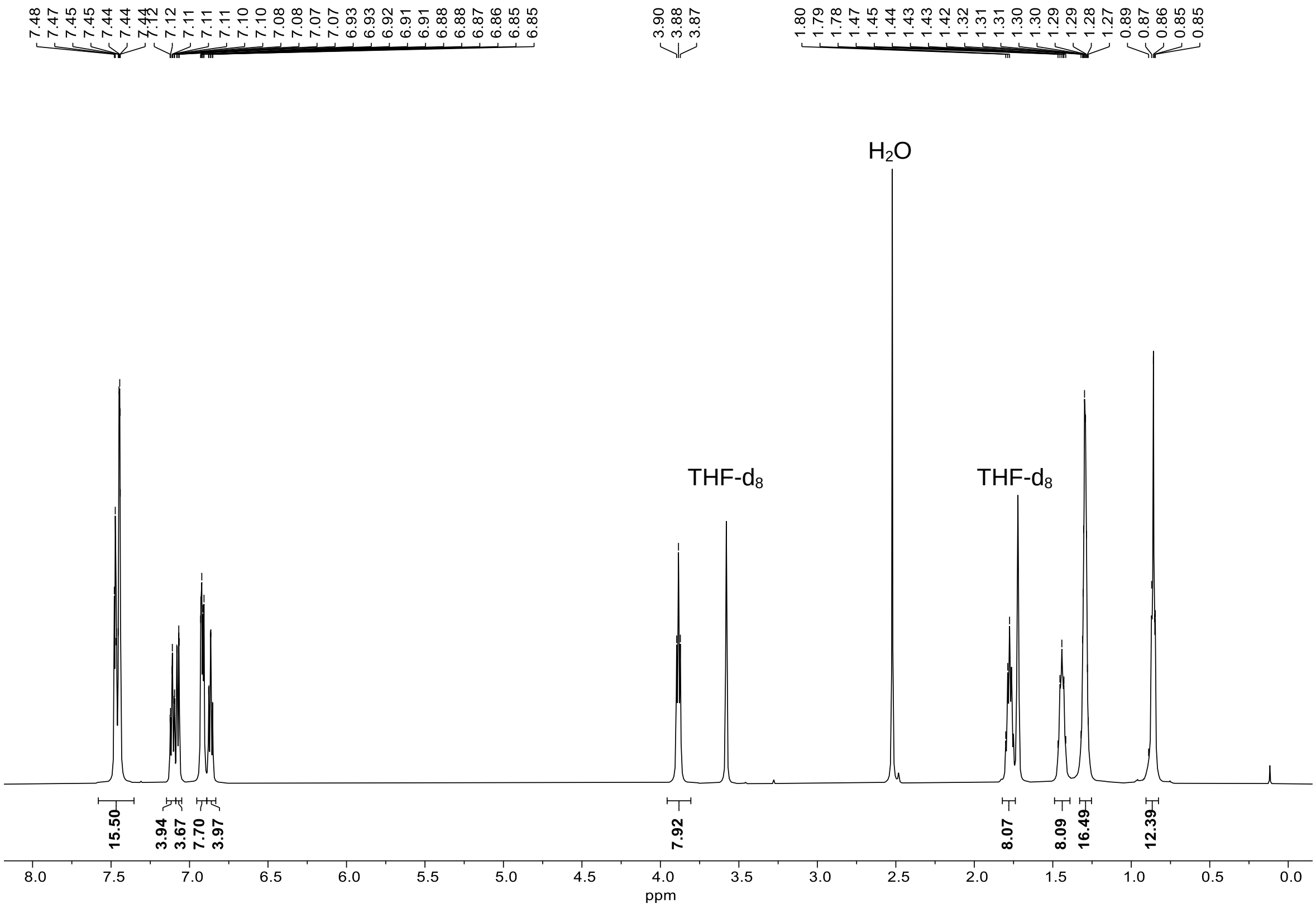

Figure S57. ^{1}H NMR spectrum (THF-d_8, 600 MHz, T = 298 K) of compound **7**.

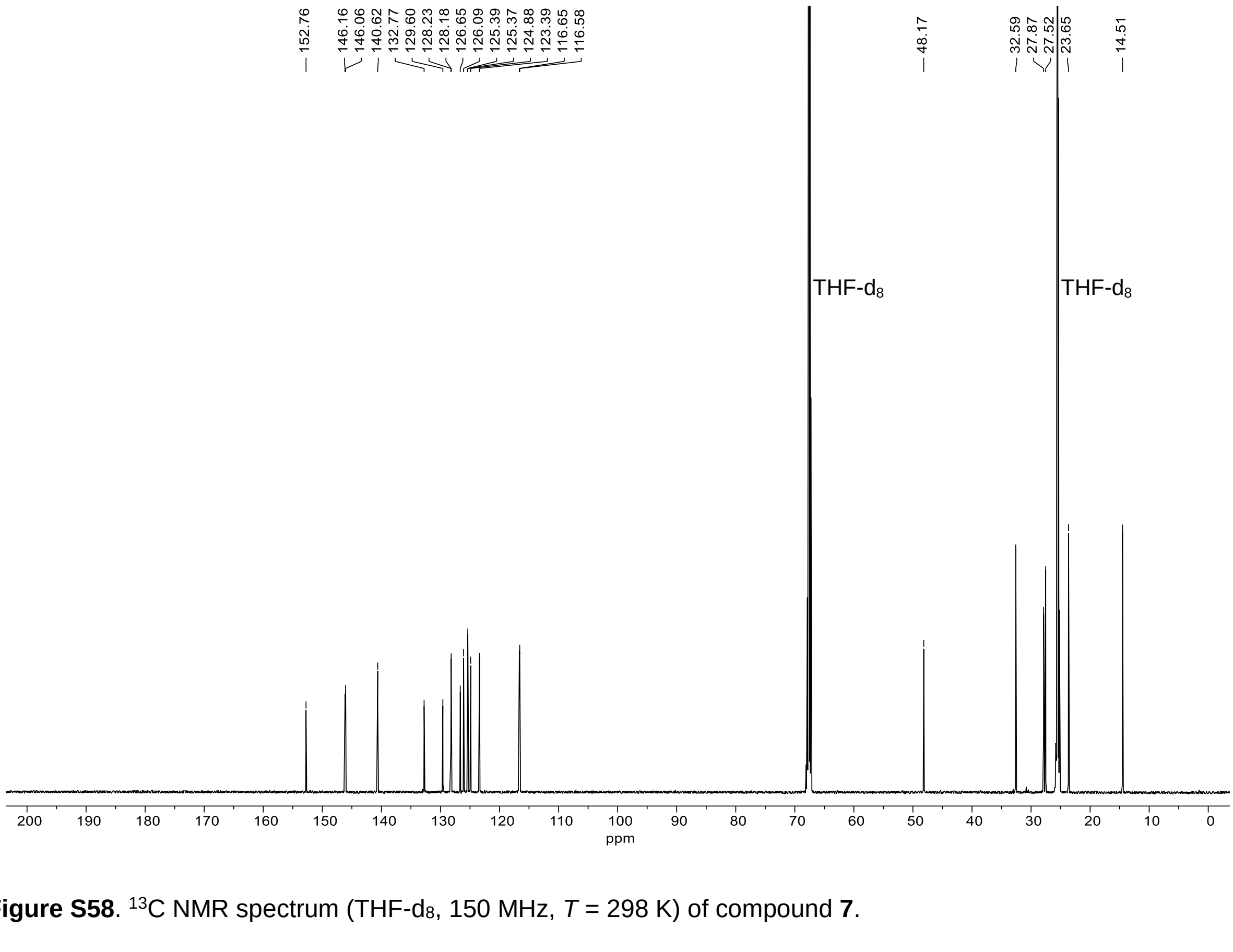

Figure S58. ^{13}C NMR spectrum (THF-d_8, 150 MHz, T = 298 K) of compound **7**.

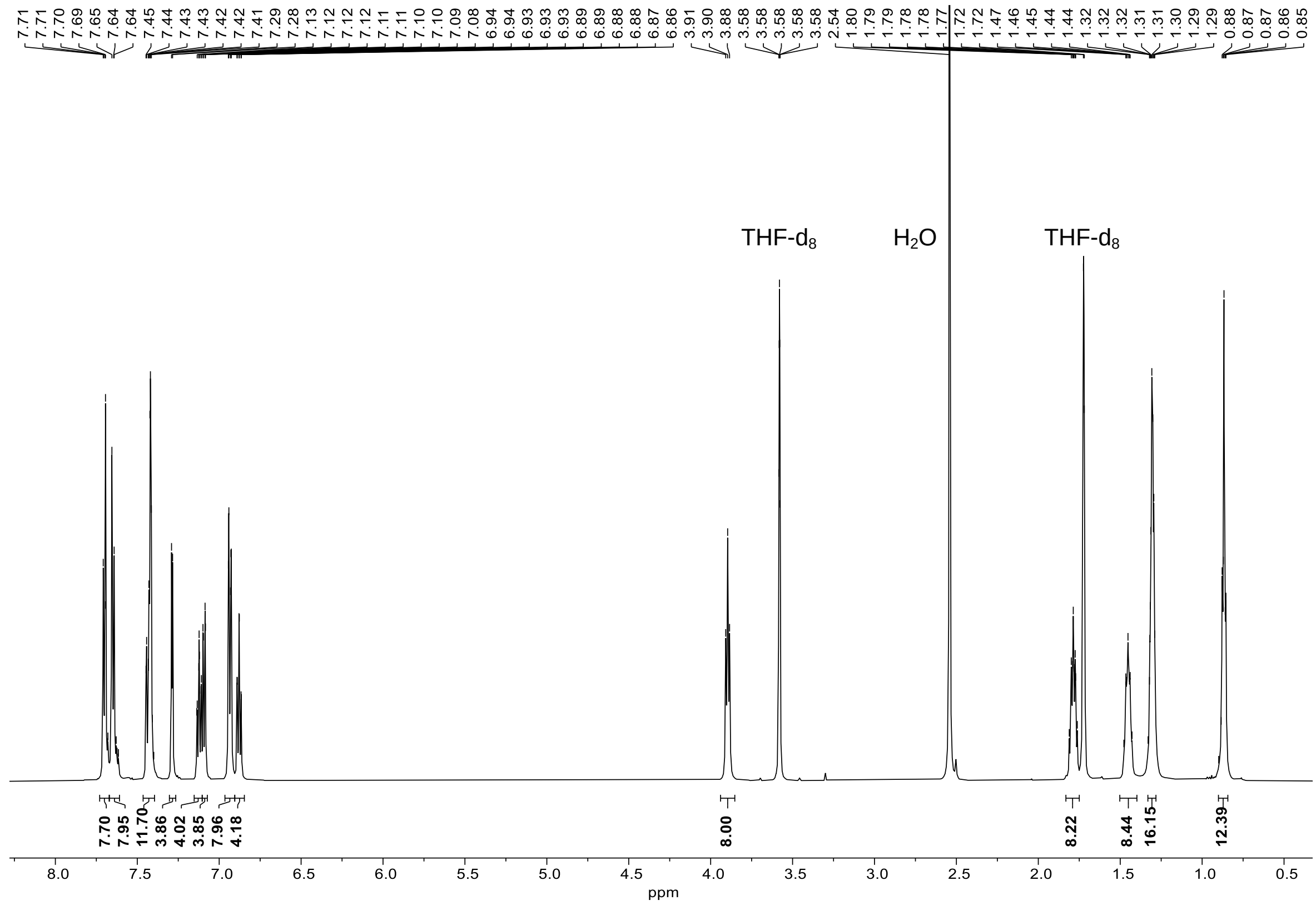

Figure S59. ^{1}H NMR spectrum (THF-d_8, 600 MHz, T = 298 K) of compound **8**.

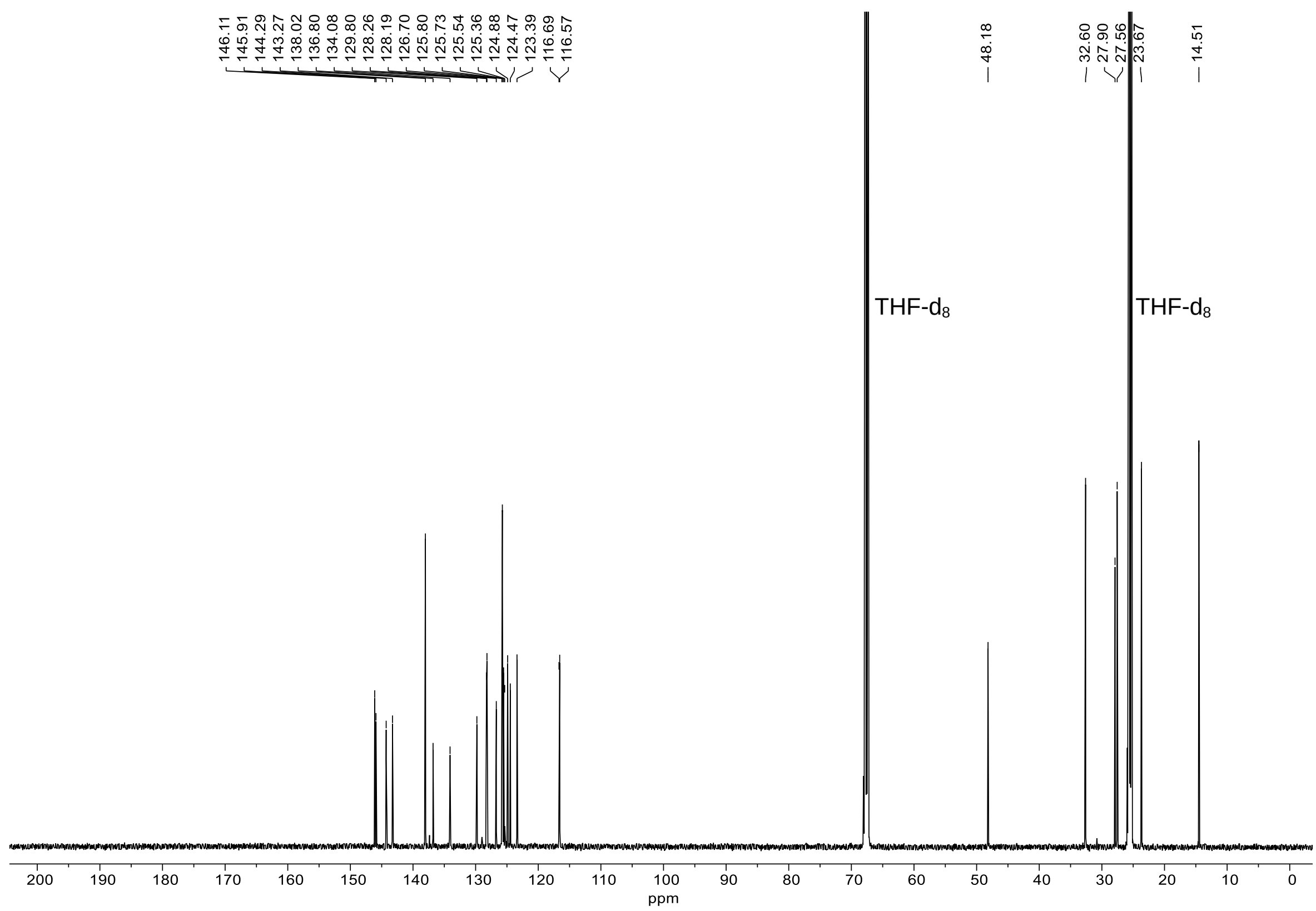

Figure S60. ^{13}C NMR spectrum (THF-d_8, 150 MHz, T = 298 K) of compound **8**.

4. Cyclic voltammograms

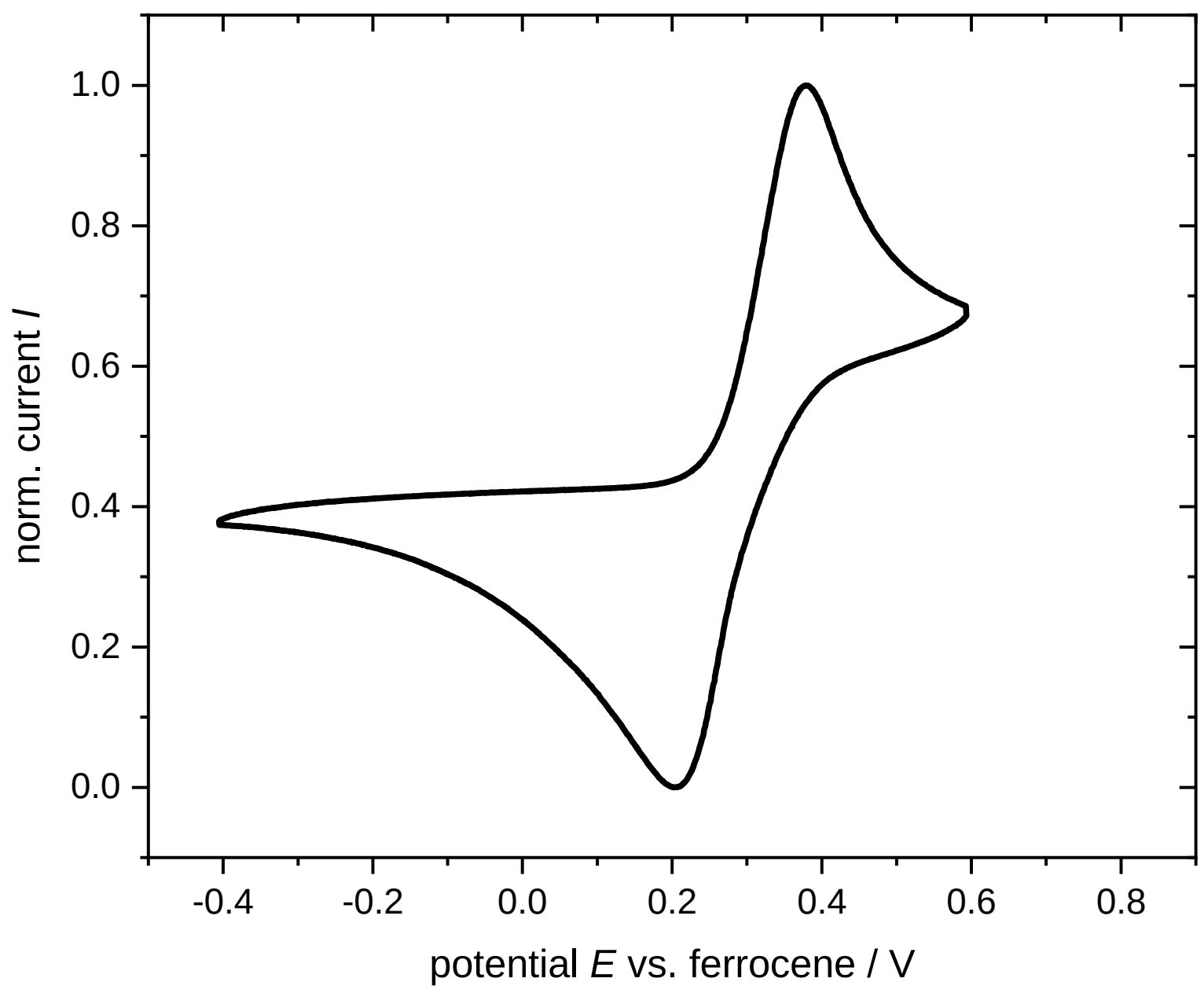

Figure S61. Cyclic voltammogram (recorded in CH_2Cl_2, T = 293 K; electrolyte: [^{n}Bu$_4$N][PF$_6$] (c = 0.1 M); working electrode: Pt disk; counter electrode: Pt wire; reference electrode: Ag/AgCl (c = 3.0 M NaCl); scan rate: v = 100 mV/s; potentials referenced to Fc/Fc^+) of compound **5a**.

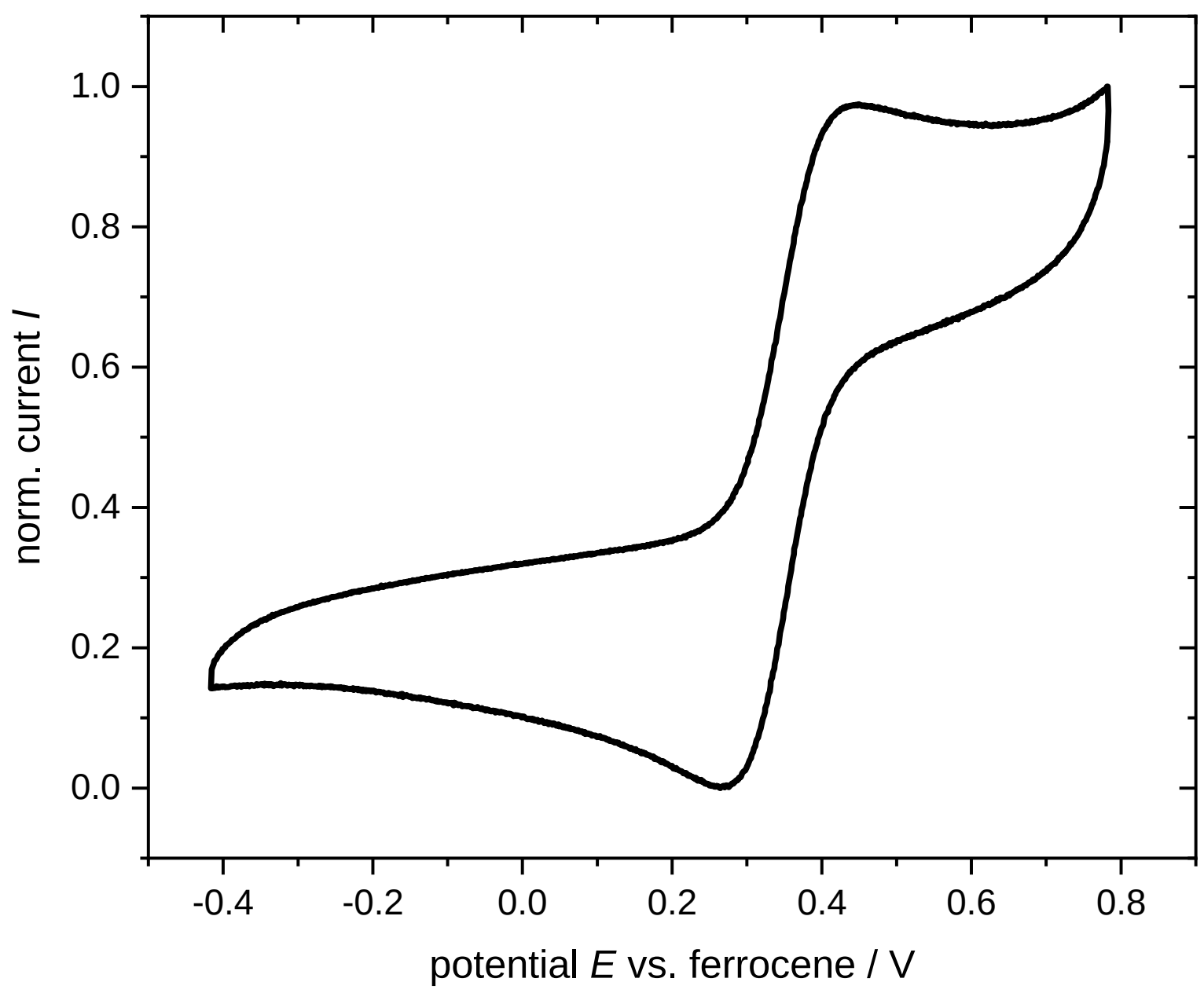

Figure S62. Cyclic voltammogram (recorded in CH_2Cl_2, T = 293 K; electrolyte: [^{n}Bu$_4$N][PF$_6$] (c = 0.1 M); working electrode: Pt disk; counter electrode: Pt wire; reference electrode: Ag/AgCl (c = 3.0 M NaCl); scan rate: v = 100 mV/s; potentials referenced to Fc/Fc^+) of compound **5b**.

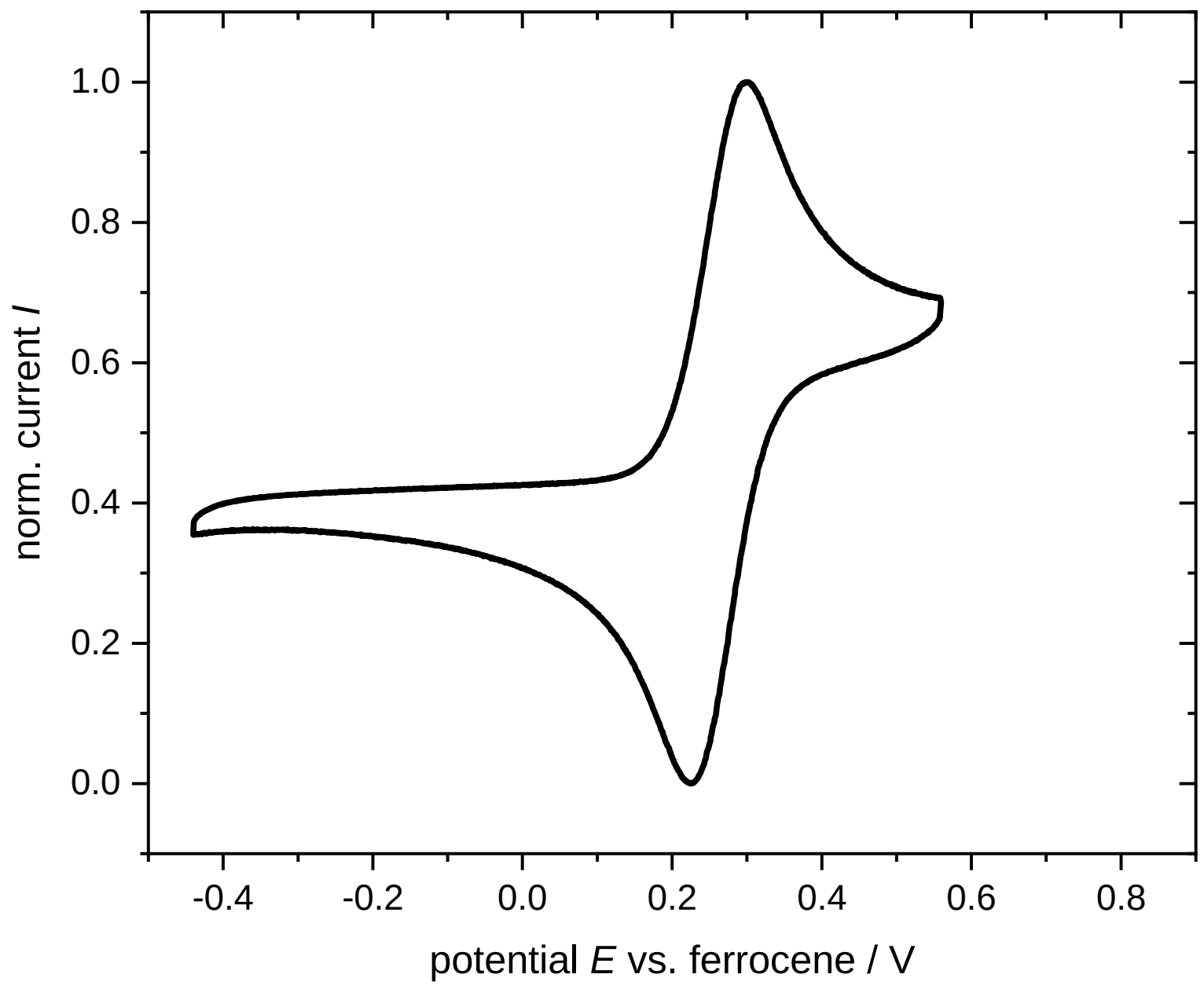

Figure S63. Cyclic voltammogram (recorded in CH_2Cl_2, T = 293 K; electrolyte: $[^nBu_4N][PF_6]$ (c = 0.1 M); working electrode: Pt disk; counter electrode: Pt wire; reference electrode: Ag/AgCl (c = 3.0 M NaCl); scan rate: v = 100 mV/s; potentials referenced to Fc/Fc^+) of compound **5c**.

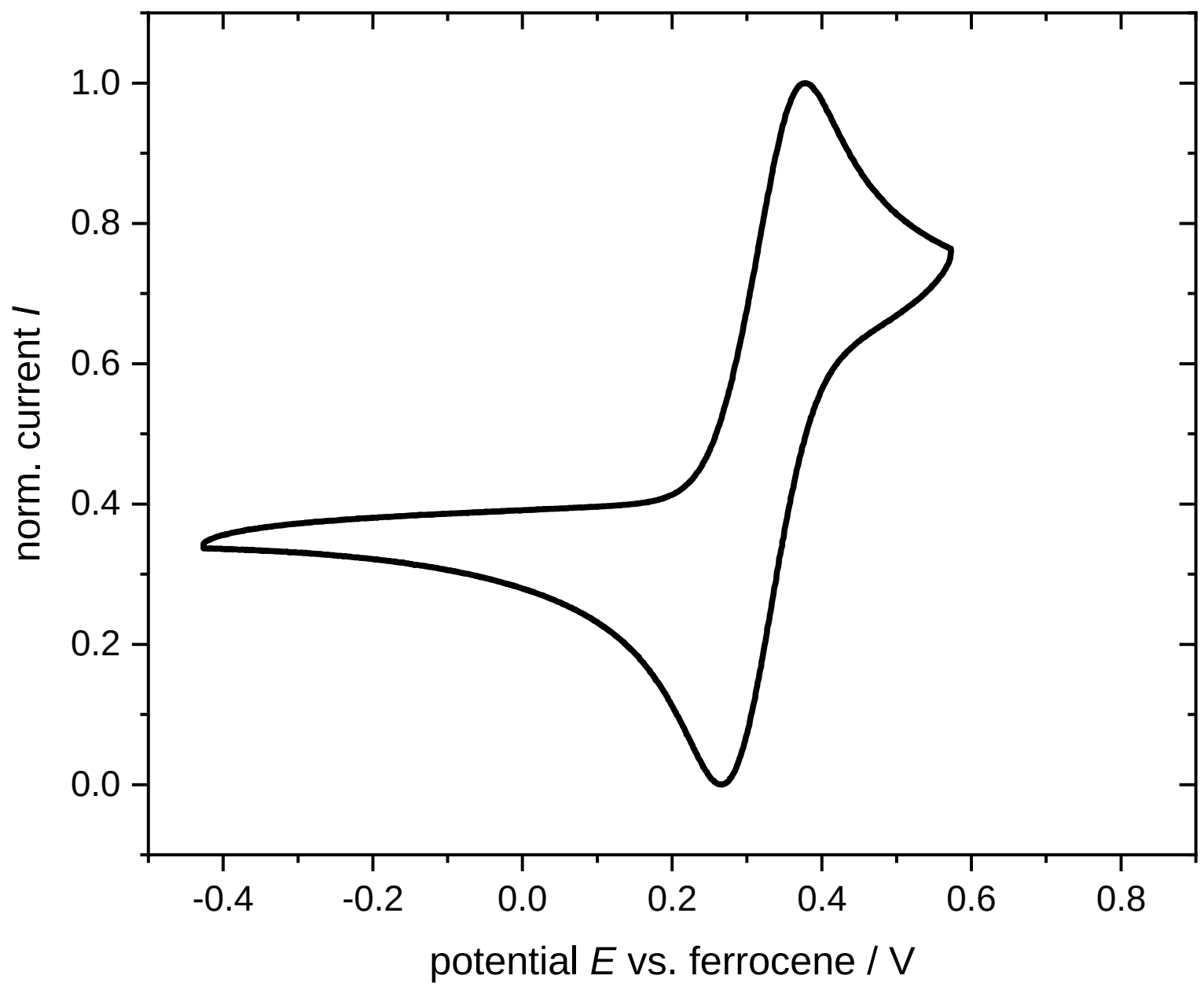

Figure S64. Cyclic voltammogram (recorded in CH_2Cl_2, T = 293 K; electrolyte: $[^nBu_4N][PF_6]$ (c = 0.1 M); working electrode: Pt disk; counter electrode: Pt wire; reference electrode: Ag/AgCl (c = 3.0 M NaCl); scan rate: v = 100 mV/s; potentials referenced to Fc/Fc^+) of compound **5d**.

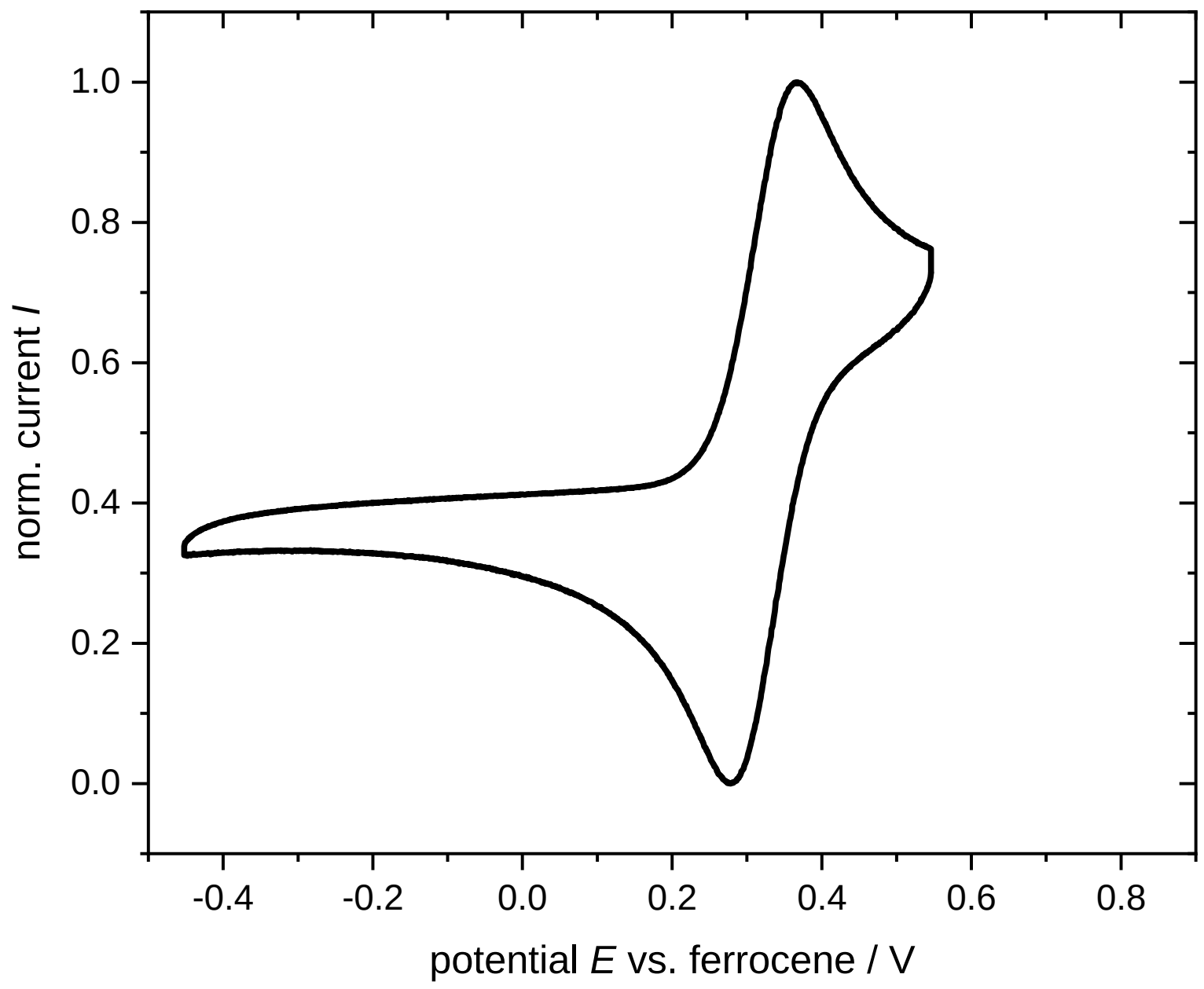

Figure S65. Cyclic voltammogram (recorded in CH_2Cl_2, T = 293 K; electrolyte: $[^nBu_4N][PF_6]$ (c = 0.1 M); working electrode: Pt disk; counter electrode: Pt wire; reference electrode: Ag/AgCl (c = 3.0 M NaCl); scan rate: ν = 100 mV/s; potentials referenced to Fc/Fc^+) of compound **5e**.

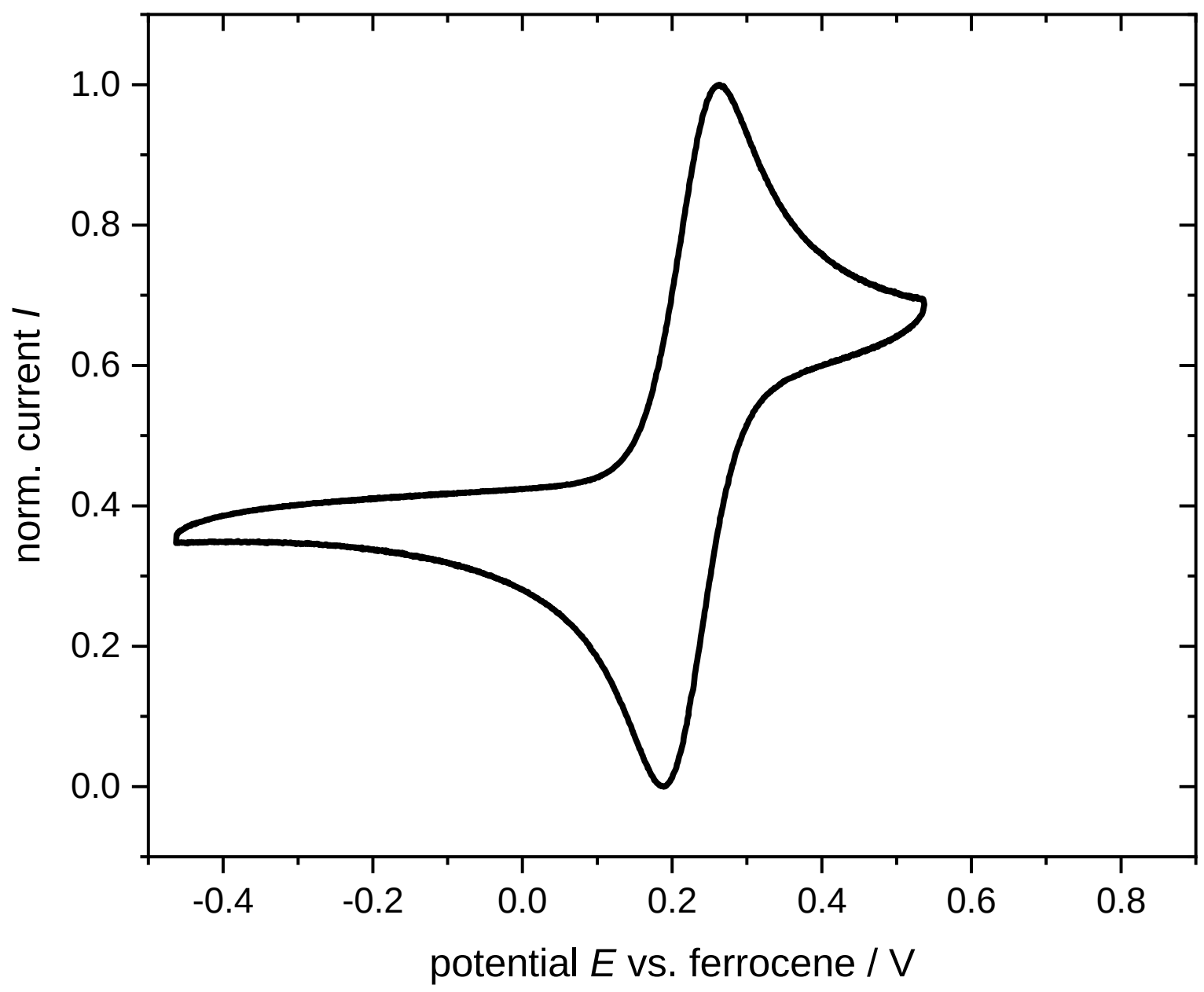

Figure S66. Cyclic voltammogram (recorded in CH_2Cl_2, T = 293 K; electrolyte: $[^nBu_4N][PF_6]$ (c = 0.1 M); working electrode: Pt disk; counter electrode: Pt wire; reference electrode: Ag/AgCl (c = 3.0 M NaCl); scan rate: ν = 100 mV/s; potentials referenced to Fc/Fc^+) of compound **5f**.

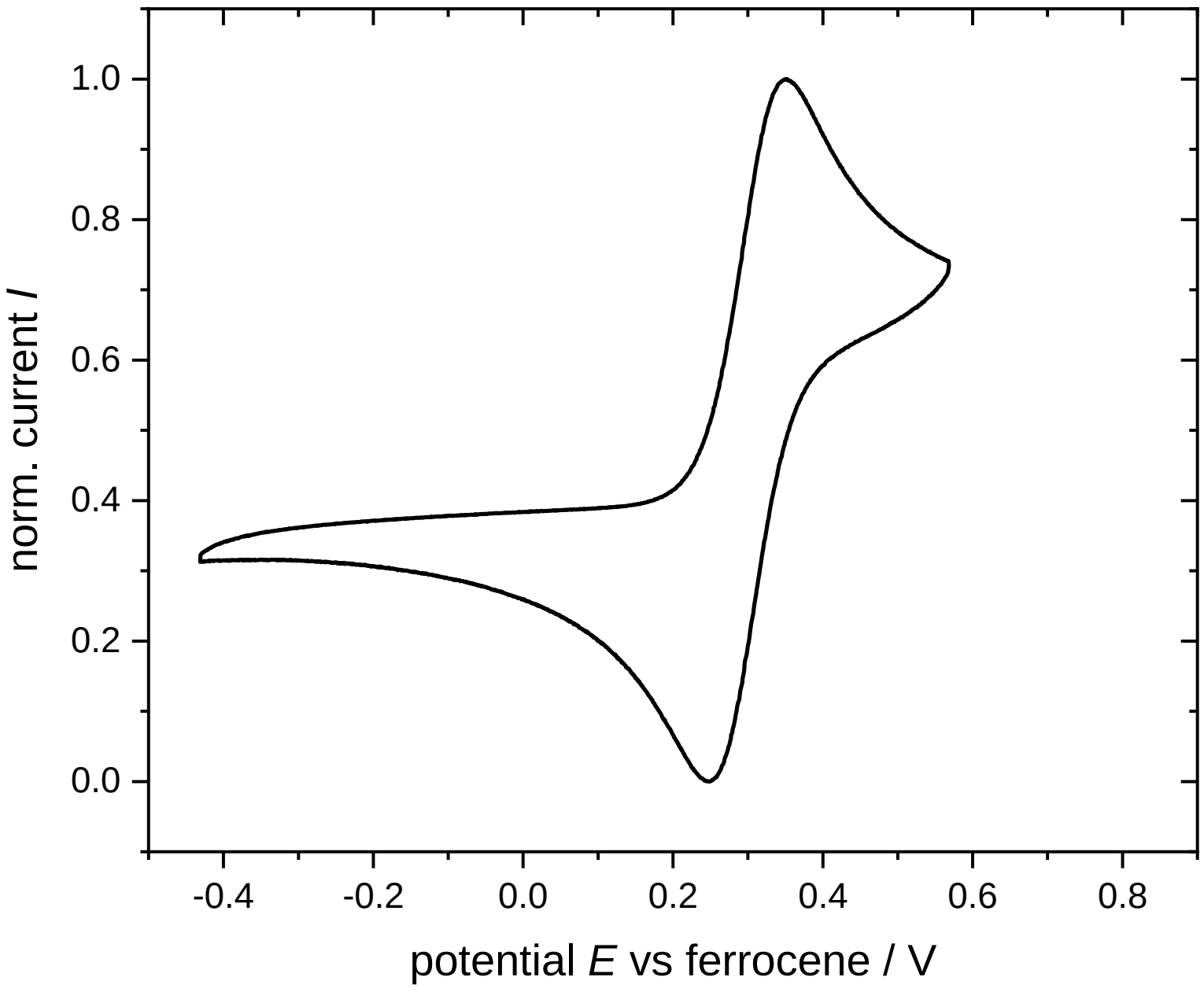

Figure S67. Cyclic voltammogram (recorded in CH_2Cl_2, T = 293 K; electrolyte: [^{n}Bu$_4$N][PF$_6$] (c = 0.1 M); working electrode: Pt disk; counter electrode: Pt wire; reference electrode: Ag/AgCl (c = 3.0 M NaCl); scan rate: v = 100 mV/s; potentials referenced to Fc/Fc$^+$) of compound **5g**.

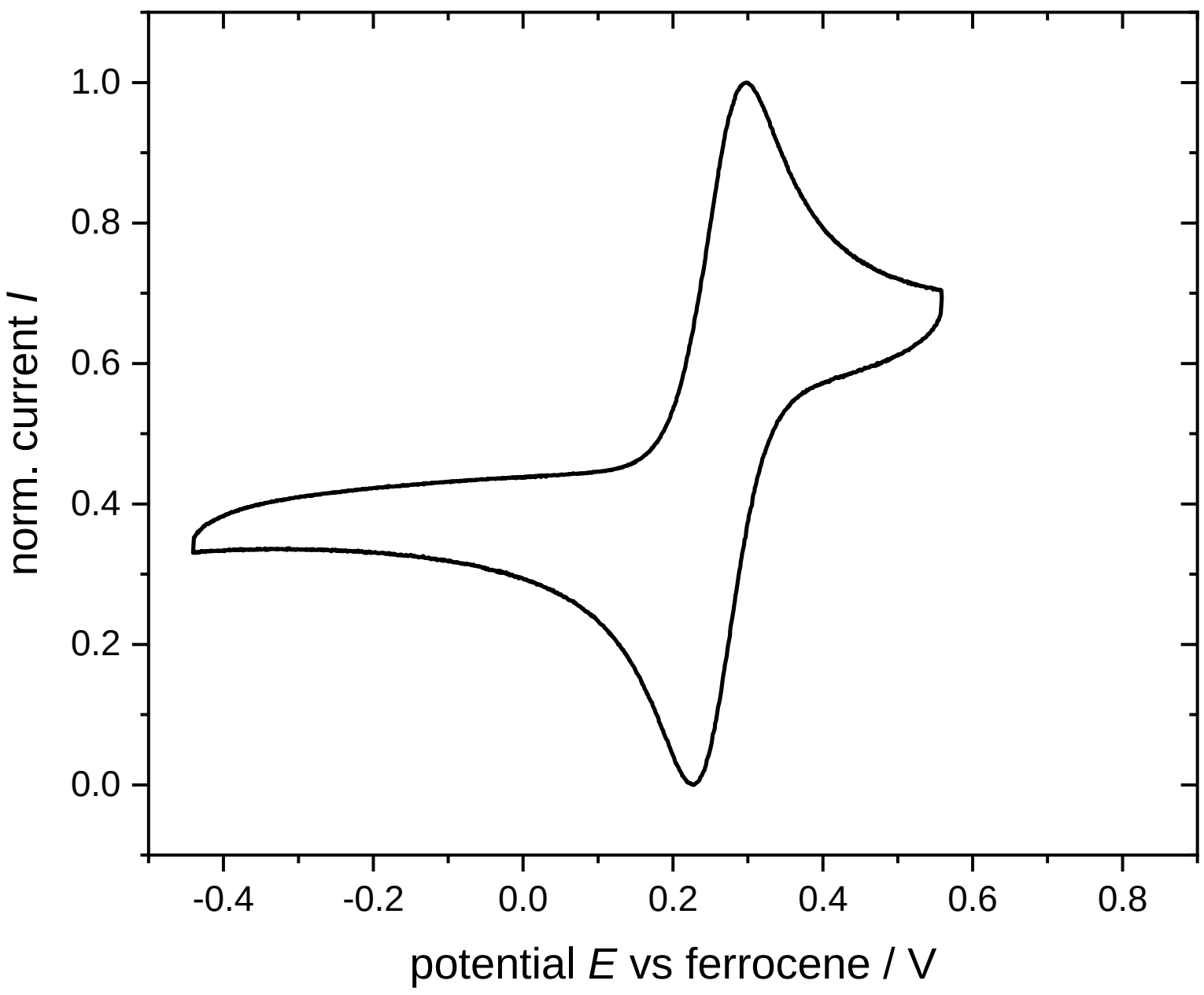

Figure S68. Cyclic voltammogram (recorded in CH_2Cl_2, T = 293 K; electrolyte: [^{n}Bu$_4$N][PF$_6$] (c = 0.1 M); working electrode: Pt disk; counter electrode: Pt wire; reference electrode: Ag/AgCl (c = 3.0 M NaCl); scan rate: v = 100 mV/s; potentials referenced to Fc/Fc$^+$) of compound **5h**.

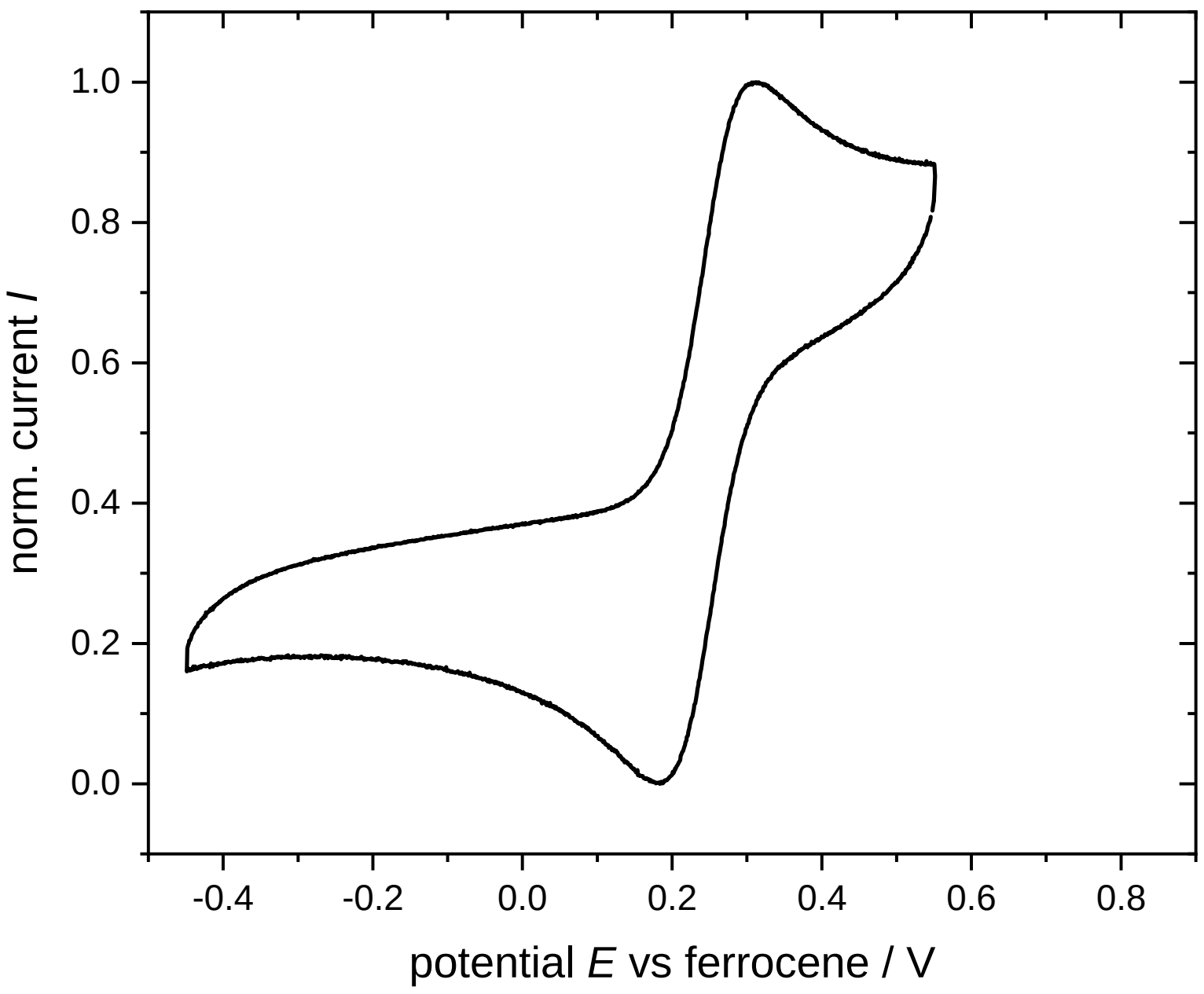

Figure S69. Cyclic voltammogram (recorded in CH_2Cl_2, T = 293 K; electrolyte: [^{n}Bu$_4$N][PF$_6$] (c = 0.1 M); working electrode: Pt disk; counter electrode: Pt wire; reference electrode: Ag/AgCl (c = 3.0 M NaCl); scan rate: v = 100 mV/s; potentials referenced to Fc/Fc$^+$) of compound **5i**.

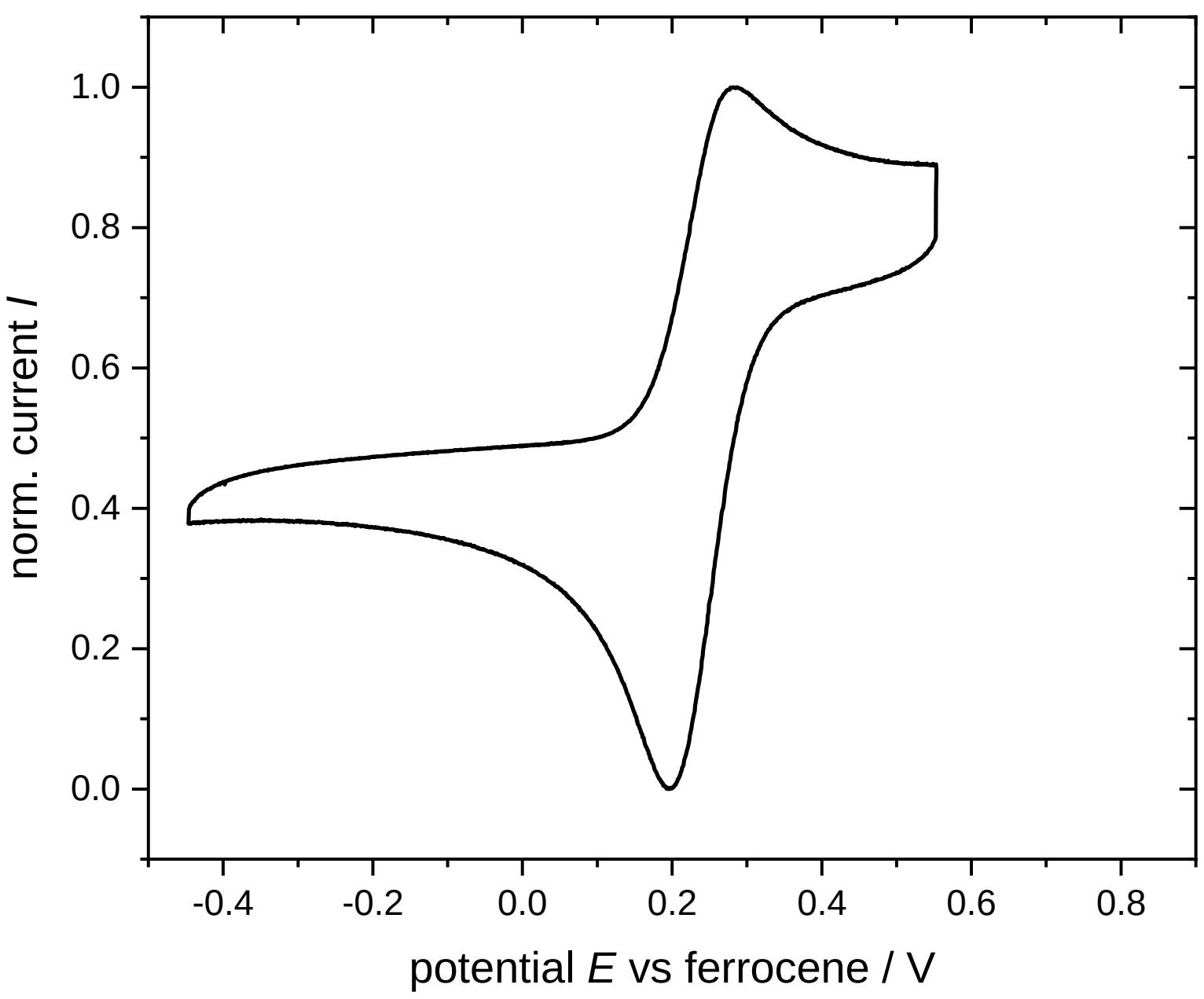

Figure S70. Cyclic voltammogram (recorded in CH_2Cl_2, T = 293 K; electrolyte: [^{n}Bu$_4$N][PF$_6$] (c = 0.1 M); working electrode: Pt disk; counter electrode: Pt wire; reference electrode: Ag/AgCl (c = 3.0 M NaCl); scan rate: v = 100 mV/s; potentials referenced to Fc/Fc$^+$) of compound **5j**.

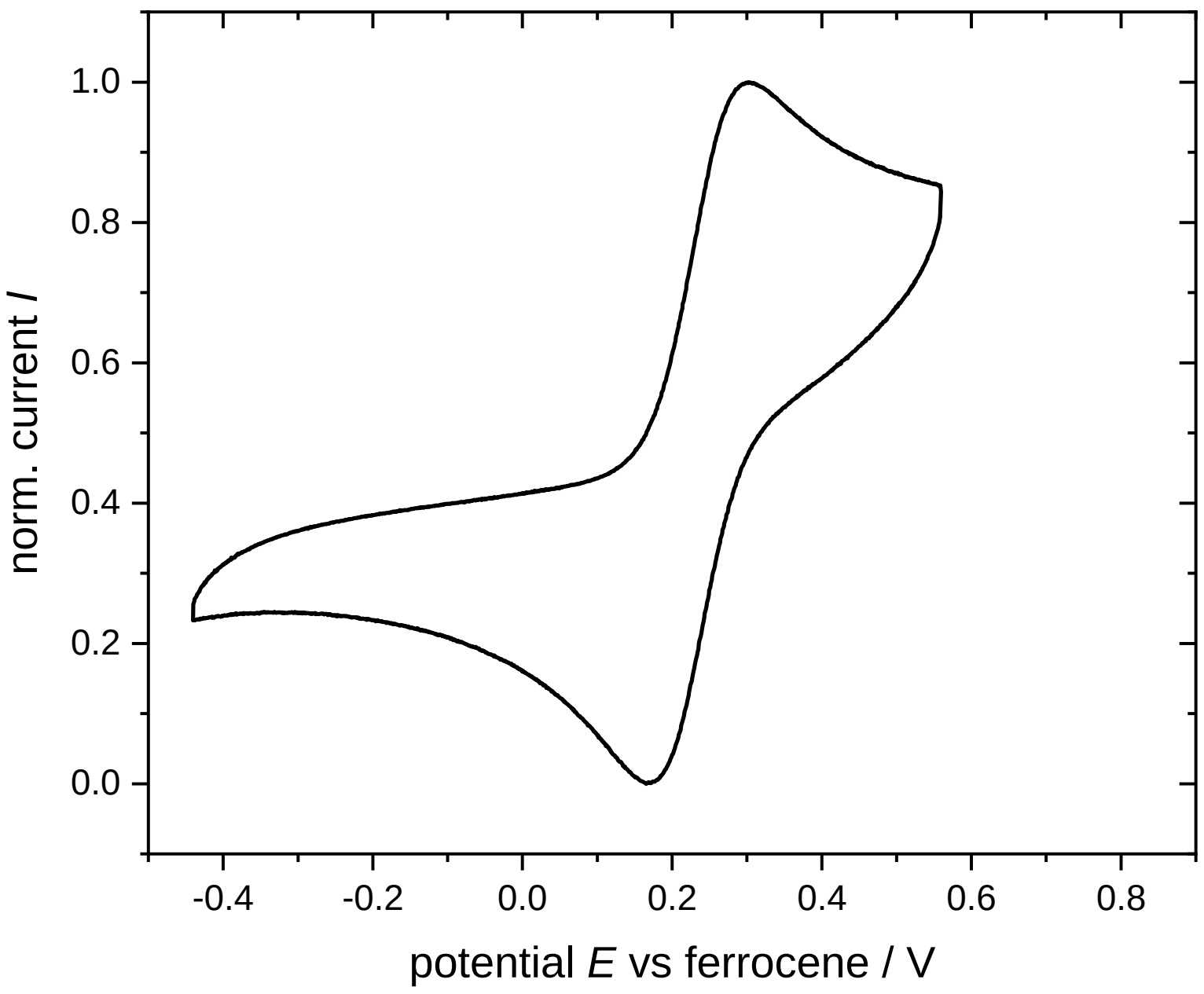

Figure S71. Cyclic voltammogram (recorded in CH_2Cl_2, T = 293 K; electrolyte: [^{n}Bu$_4$N][PF$_6$] (c = 0.1 M); working electrode: Pt disk; counter electrode: Pt wire; reference electrode: Ag/AgCl (c = 3.0 M NaCl); scan rate: v = 100 mV/s; potentials referenced to Fc/Fc$^+$) of compound **5k**.

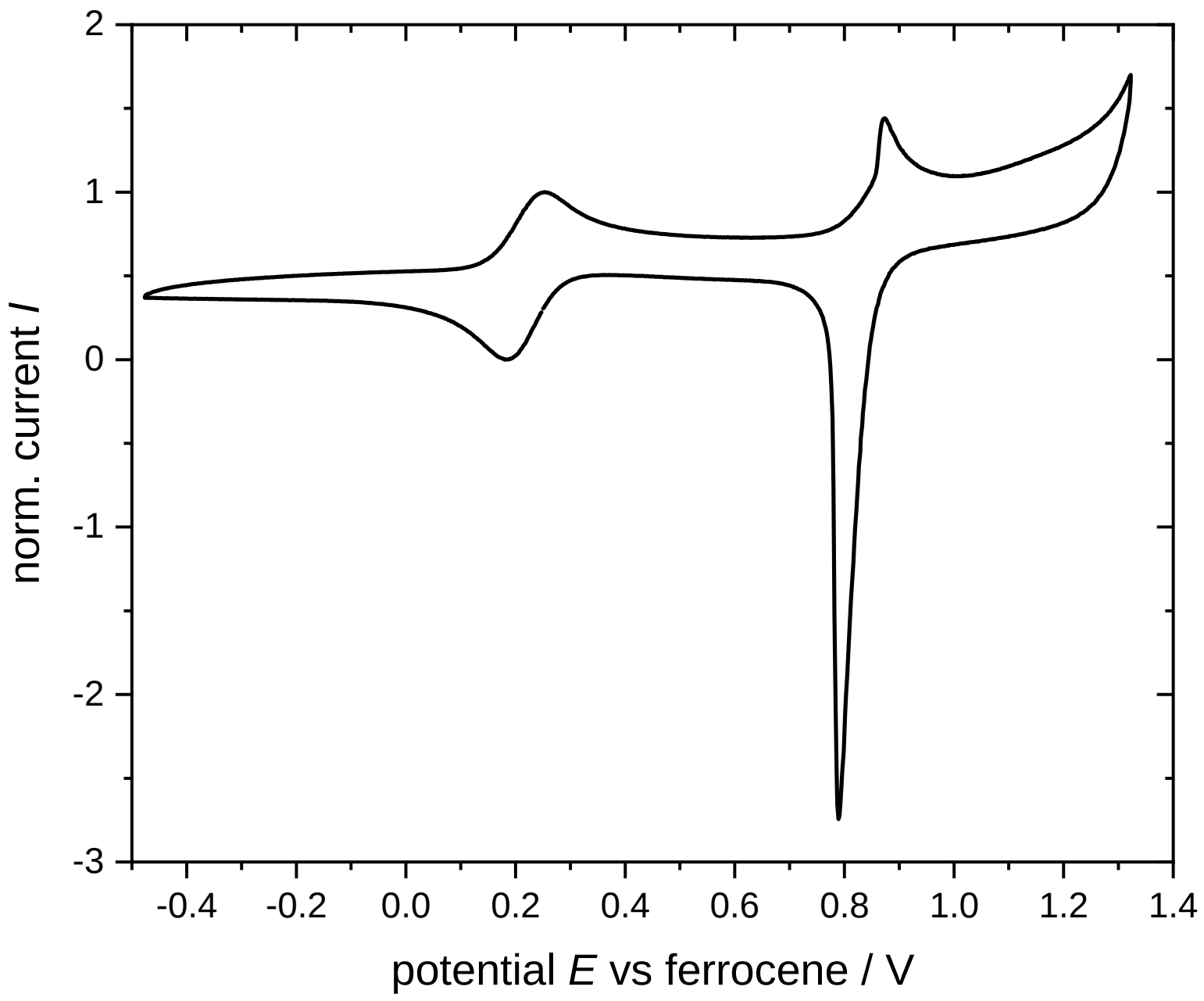

Figure S72. Cyclic voltammogram (recorded in CH_2Cl_2, T = 293 K; electrolyte: [^{n}Bu$_4$N][PF$_6$] (c = 0.1 M); working electrode: Pt disk; counter electrode: Pt wire; reference electrode: Ag/AgCl (c = 3.0 M NaCl); scan rate: v = 100 mV/s; potentials referenced to Fc/Fc$^+$) of compound **5l**.

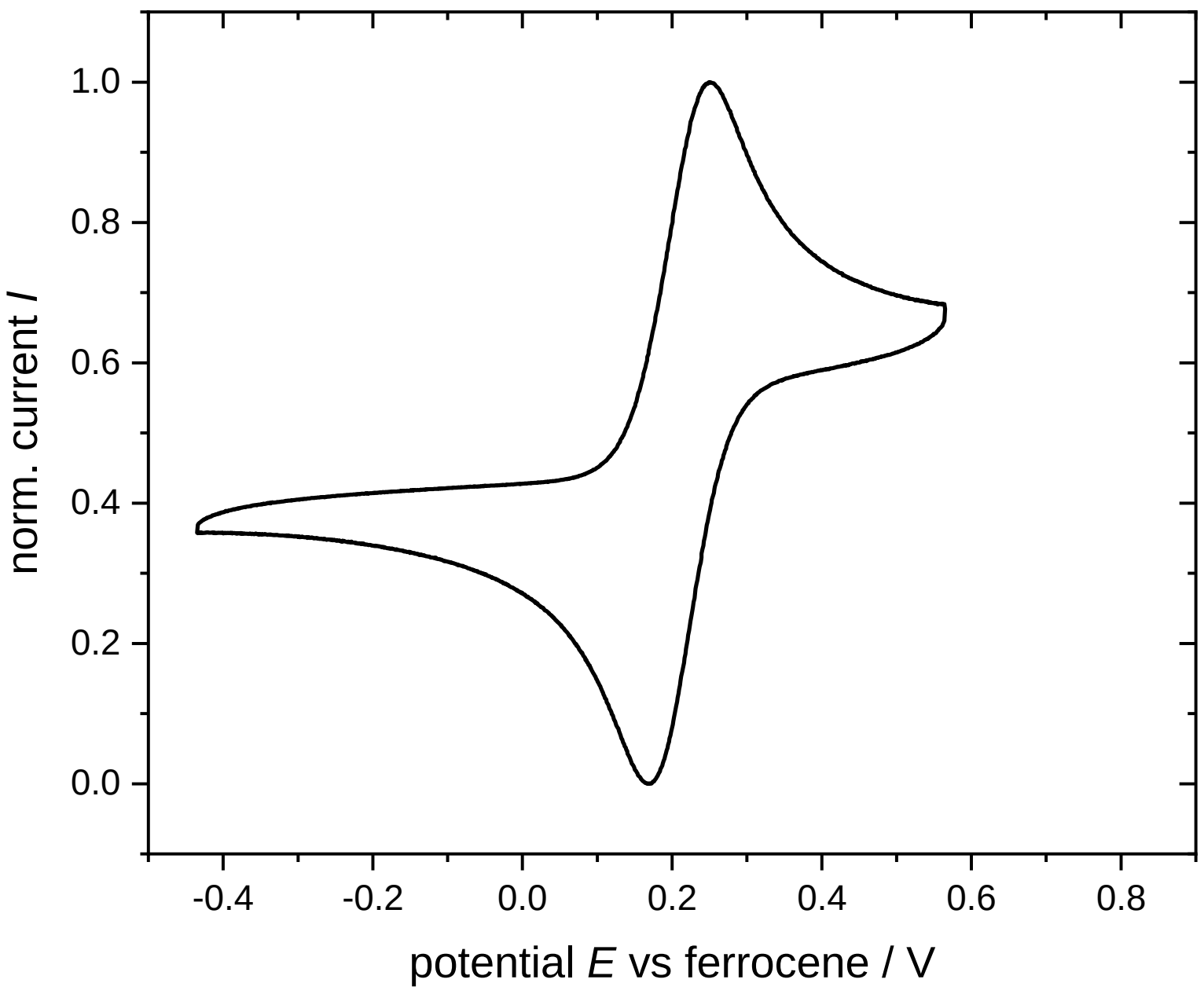

Figure S73. Cyclic voltammogram (recorded in CH_2Cl_2, T = 293 K; electrolyte: [^{n}Bu$_4$N][PF$_6$] (c = 0.1 M); working electrode: Pt disk; counter electrode: Pt wire; reference electrode: Ag/AgCl (c = 3.0 M NaCl); scan rate: v = 100 mV/s; potentials referenced to Fc/Fc$^+$) of compound **5m**.

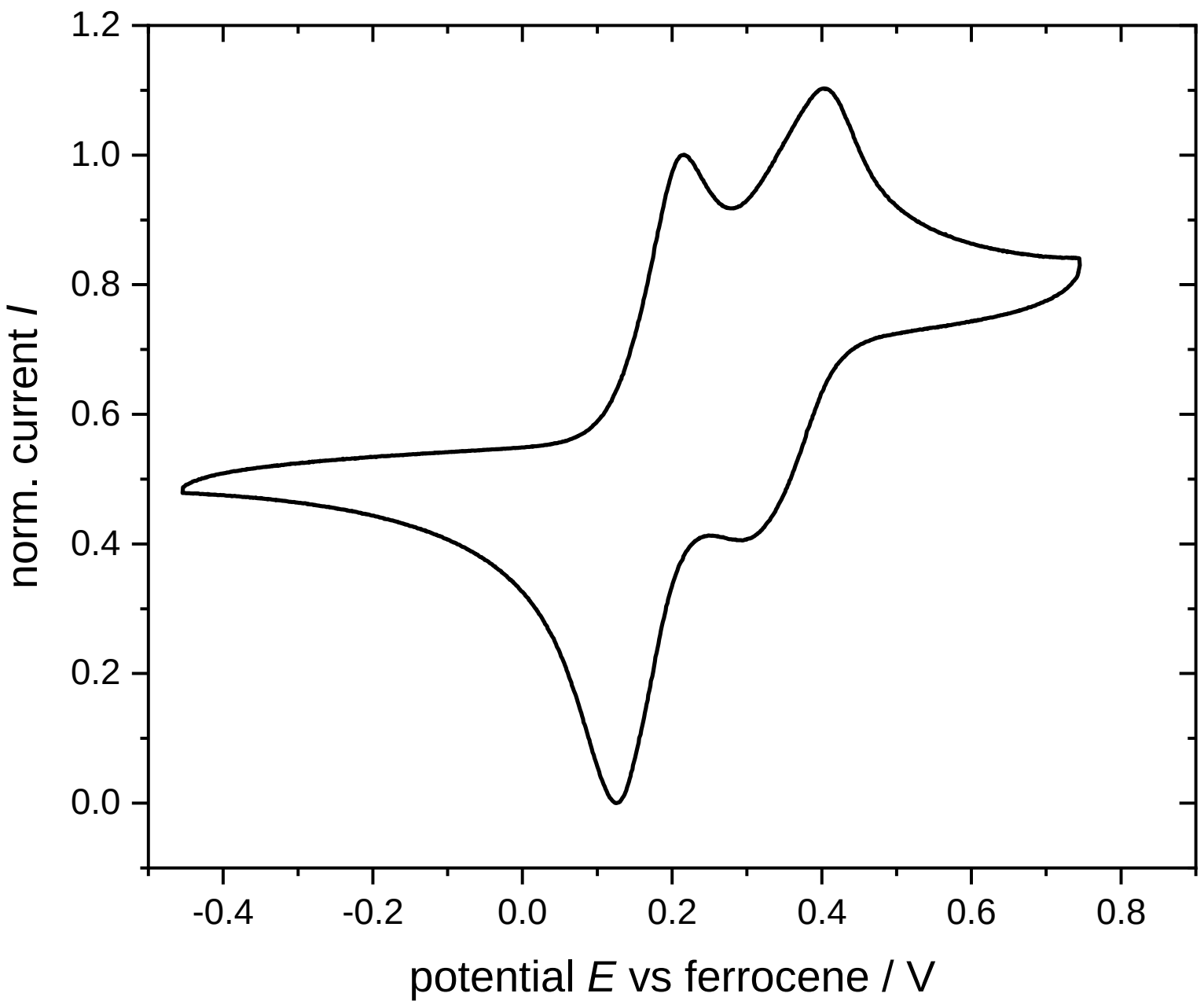

Figure S74. Cyclic voltammogram (recorded in CH_2Cl_2, T = 293 K; electrolyte: [^{n}Bu$_4$N][PF$_6$] (c = 0.1 M); working electrode: Pt disk; counter electrode: Pt wire; reference electrode: Ag/AgCl (c = 3.0 M NaCl); scan rate: v = 100 mV/s; potentials referenced to Fc/Fc$^+$) of compound **5n**.

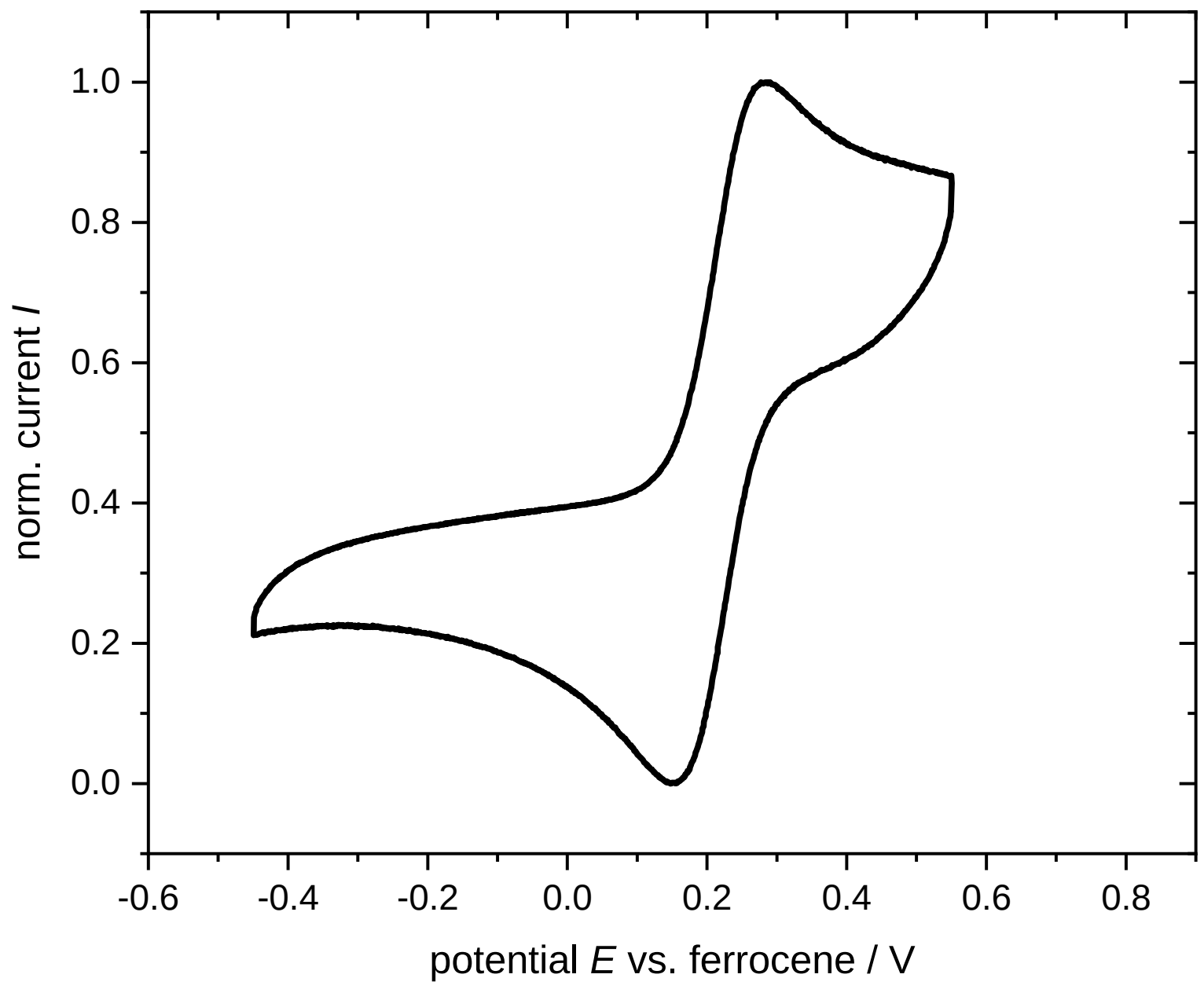

Figure S75. Cyclic voltammogram (recorded in CH_2Cl_2, T = 293 K; electrolyte: [^{n}Bu$_4$N][PF$_6$] (c = 0.1 M); working electrode: Pt disk; counter electrode: Pt wire; reference electrode: Ag/AgCl (c = 3.0 M NaCl); scan rate: v = 100 mV/s; potentials referenced to Fc/Fc$^+$) of compound **5o**.

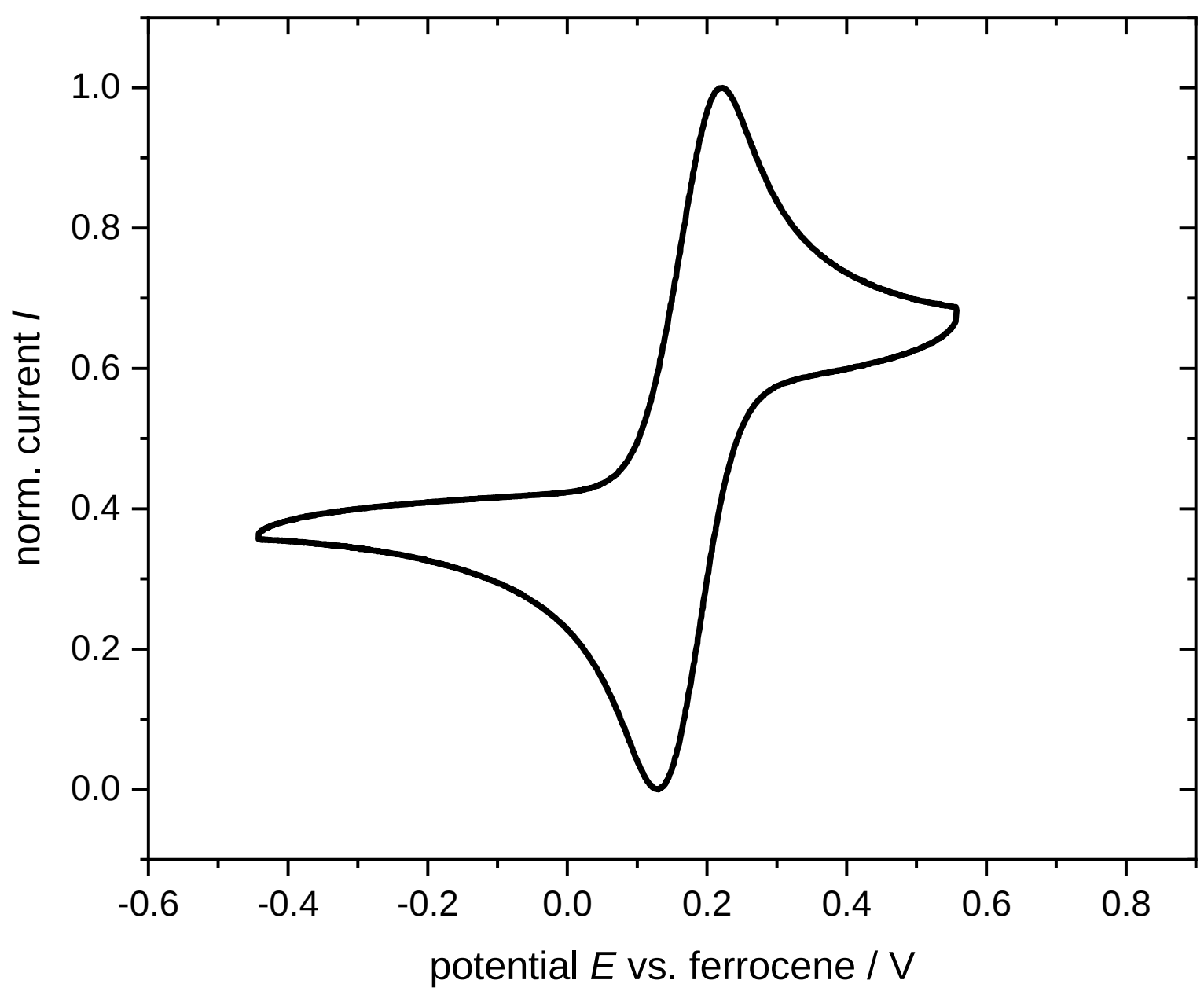

Figure S76. Cyclic voltammogram (recorded in CH_2Cl_2, T = 293 K; electrolyte: [^{n}Bu$_4$N][PF$_6$] (c = 0.1 M); working electrode: Pt disk; counter electrode: Pt wire; reference electrode: Ag/AgCl (c = 3.0 M NaCl); scan rate: v = 100 mV/s; potentials referenced to Fc/Fc$^+$) of compound **5p**.

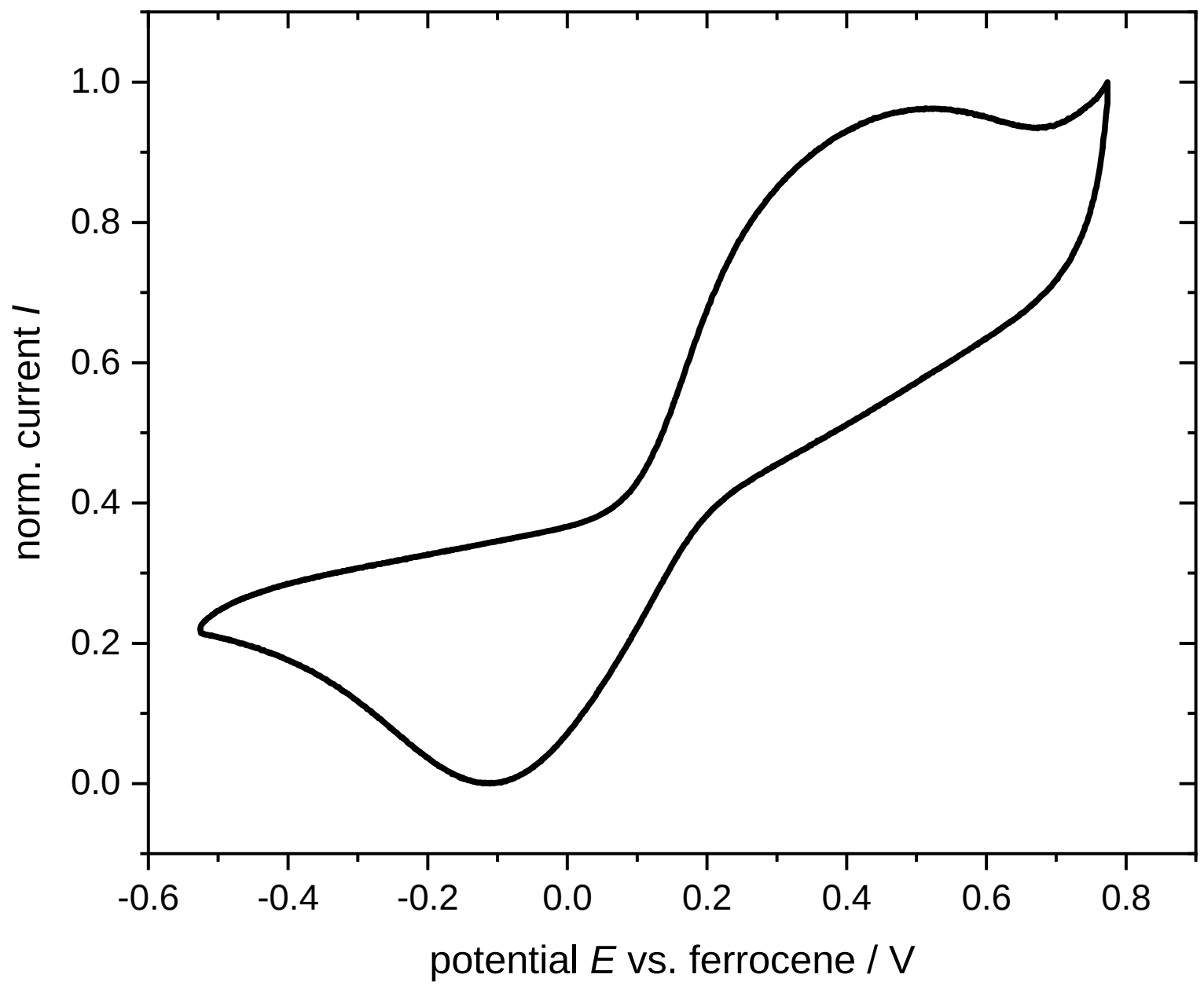

Figure S77. Cyclic voltammogram (recorded in CH_2Cl_2, T = 293 K; electrolyte: $[^{n}Bu_4N][PF_6]$ (c = 0.1 M); working electrode: Pt disk; counter electrode: Pt wire; reference electrode: Ag/AgCl (c = 3.0 M NaCl); scan rate: v = 100 mV/s; potentials referenced to Fc/Fc^+) of compound **5q**.

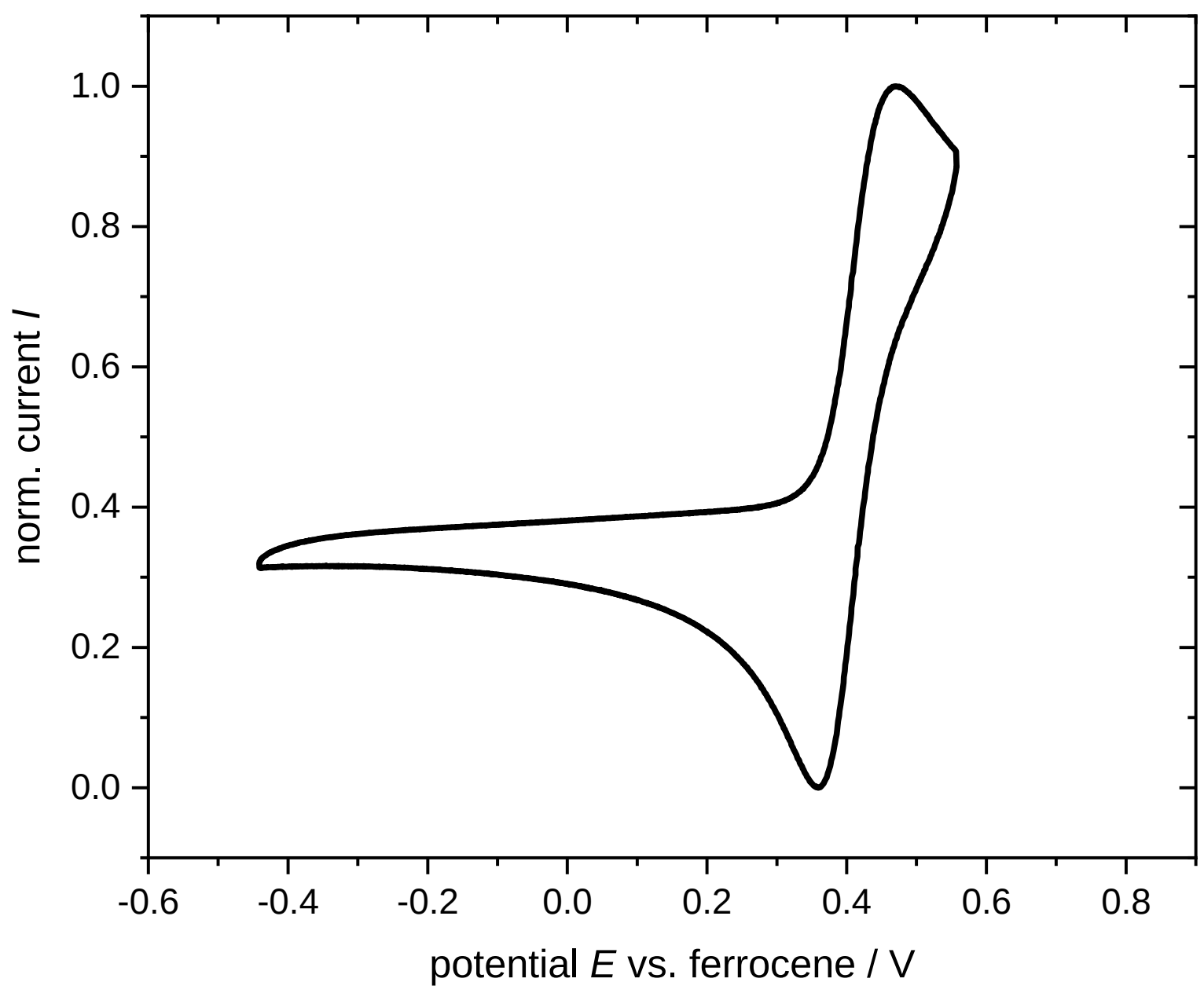

Figure S78. Cyclic voltammogram (recorded in CH_2Cl_2, T = 293 K; electrolyte: $[^{n}Bu_4N][PF_6]$ (c = 0.1 M); working electrode: Pt disk; counter electrode: Pt wire; reference electrode: Ag/AgCl (c = 3.0 M NaCl); scan rate: v = 100 mV/s; potentials referenced to Fc/Fc^+) of compound **5r**.

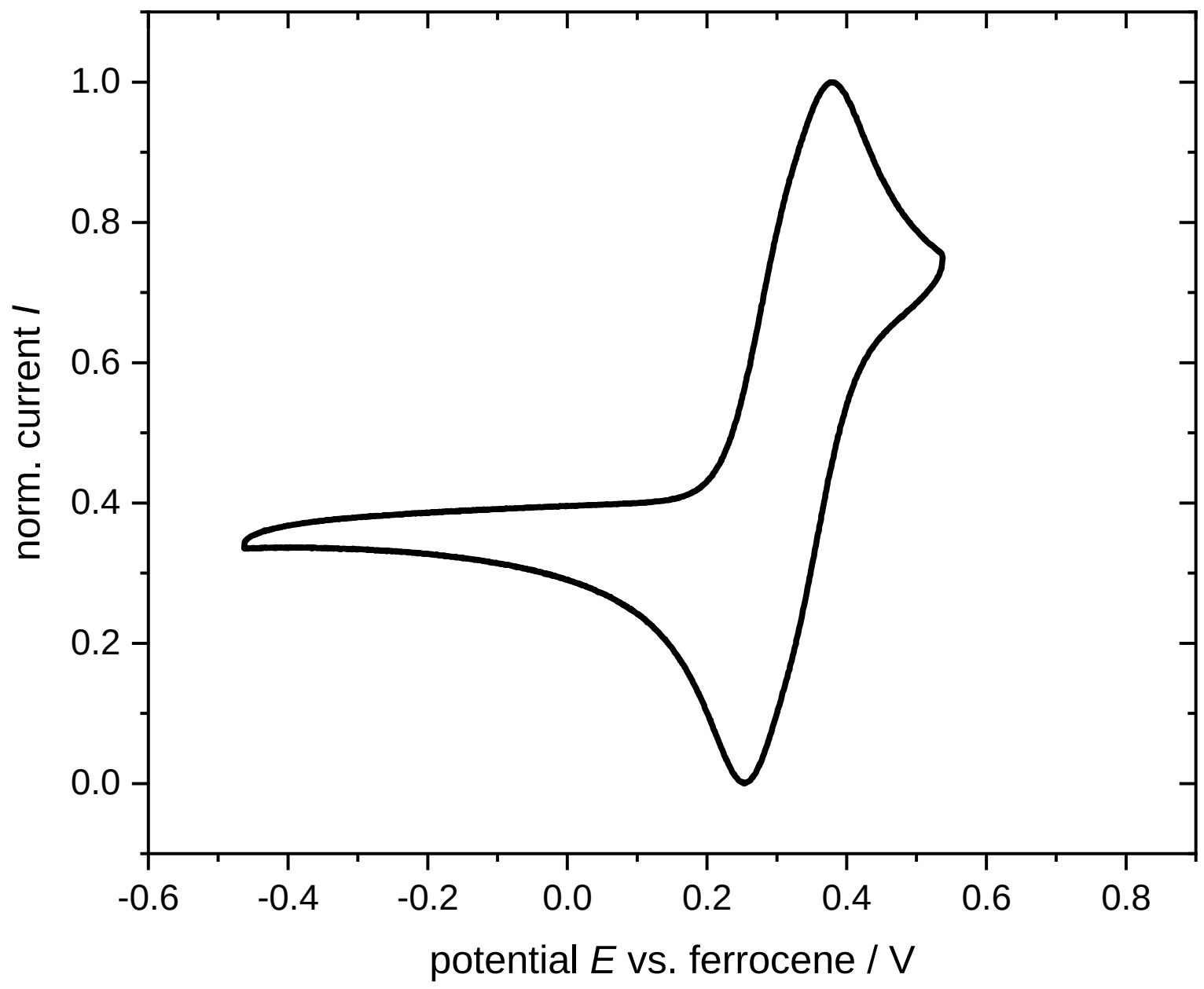

Figure S79. Cyclic voltammogram (recorded in CH_2Cl_2, T = 293 K; electrolyte: $[^{n}Bu_4N][PF_6]$ (c = 0.1 M); working electrode: Pt disk; counter electrode: Pt wire; reference electrode: Ag/AgCl (c = 3.0 M NaCl); scan rate: v = 100 mV/s; potentials referenced to Fc/Fc^+) of compound **6**.

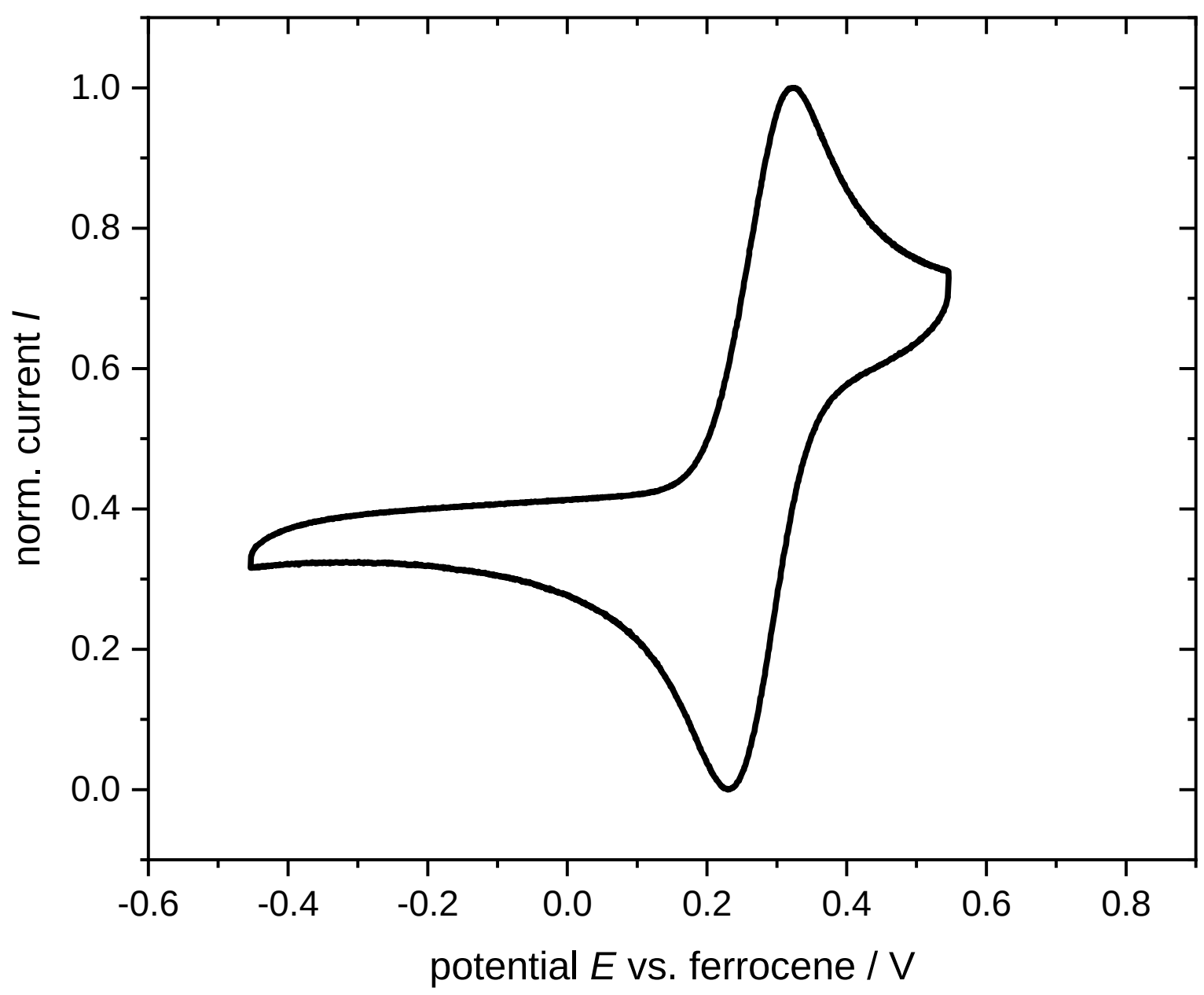

Figure S80. Cyclic voltammogram (recorded in CH_2Cl_2, T = 293 K; electrolyte: $[^{n}Bu_4N][PF_6]$ (c = 0.1 M); working electrode: Pt disk; counter electrode: Pt wire; reference electrode: Ag/AgCl (c = 3.0 M NaCl); scan rate: v = 100 mV/s; potentials referenced to Fc/Fc^+) of compound **7**.

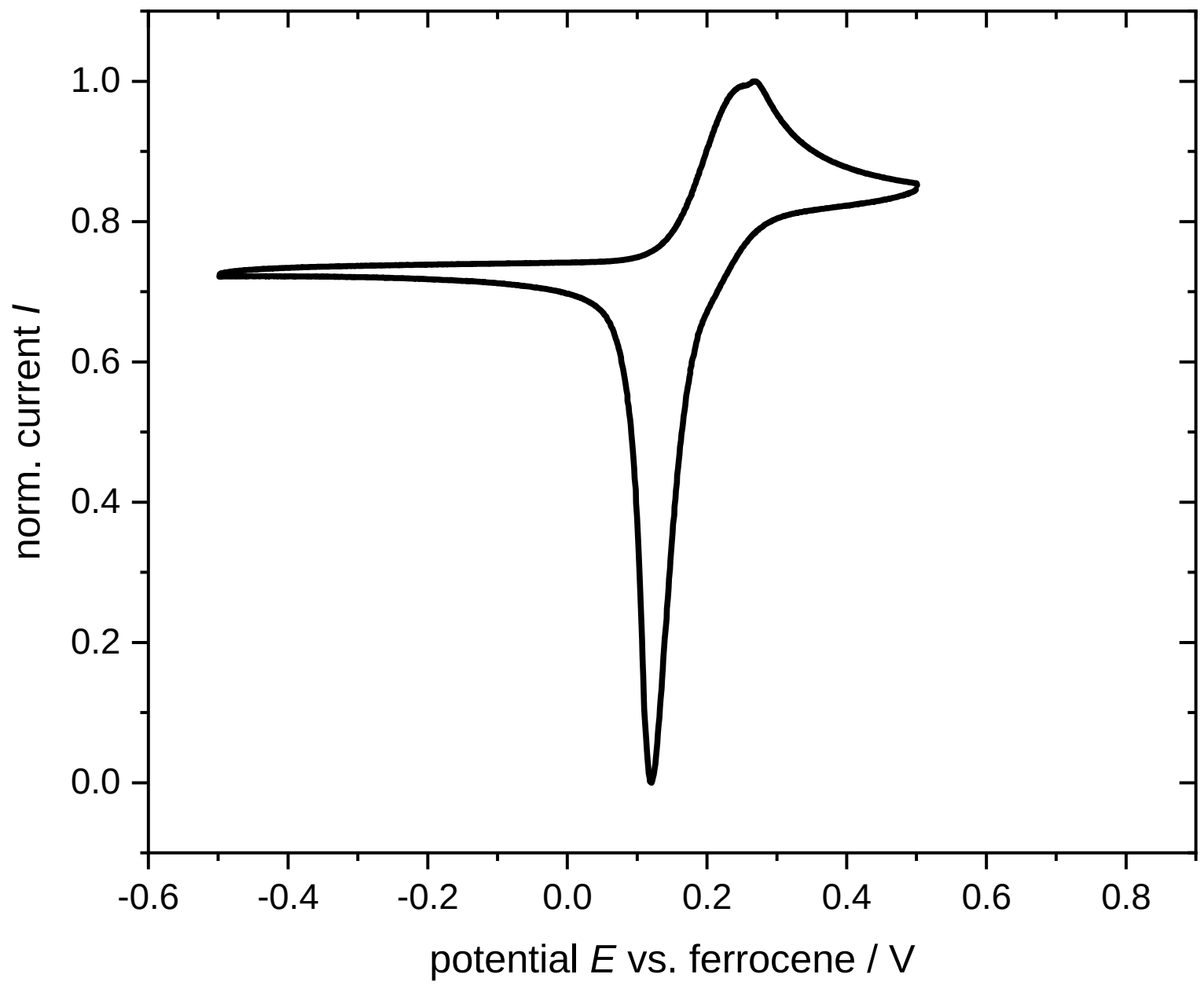

Figure S81. Cyclic voltammogram (recorded in CH_2Cl_2, T = 293 K; electrolyte: [$^nBu_4N$][PF_6] (c = 0.1 M); working electrode: Pt disk; counter electrode: Pt wire; reference electrode: Ag/AgCl (c = 3.0 M NaCl); scan rate: v = 100 mV/s; potentials referenced to Fc/Fc^+) of compound **8**.

5. Absorption and emission spectra

All solution-phase spectra were recorded in dichloromethane at T = 293 K. For absorption measurements, solutions with a concentration of $c = 10^{-5}$ M were used, whereas for emission measurements, a lower concentration of $c = 10^{-7}$ M was employed to minimize reabsorption and inner filter effects. For solid-state emission, λ_{exc} was chosen based on the corresponding excitation spectrum. All spectra were corrected for the instrumental response.

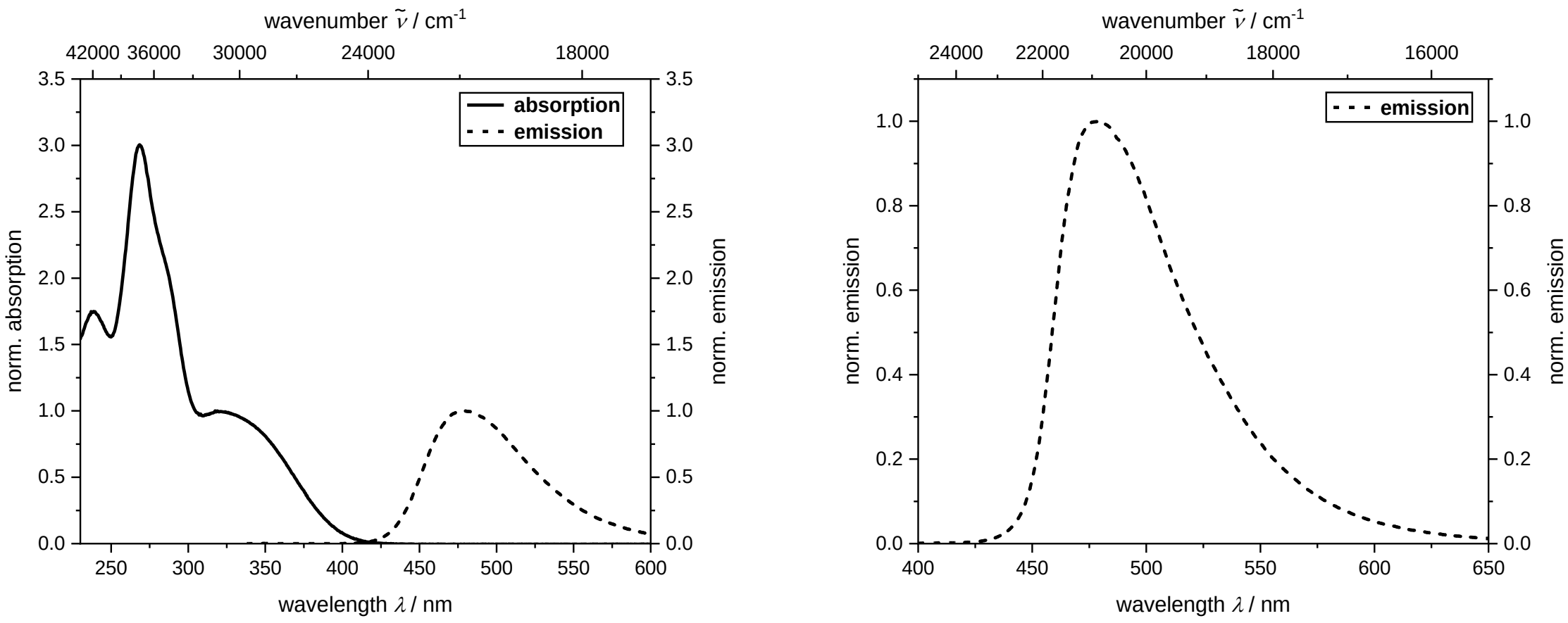

Figure S82. Left: normalized absorption (recorded in CH_2Cl_2, T = 293 K, $c = 10^{-5}$ M, solid line) and emission spectrum (recorded in CH_2Cl_2, T = 293 K, $c = 10^{-7}$ M, dashed line) of compound **5a**. Right: normalized emission spectrum (recorded in the solid state, T = 293 K, dashed line) of compound **5a**.

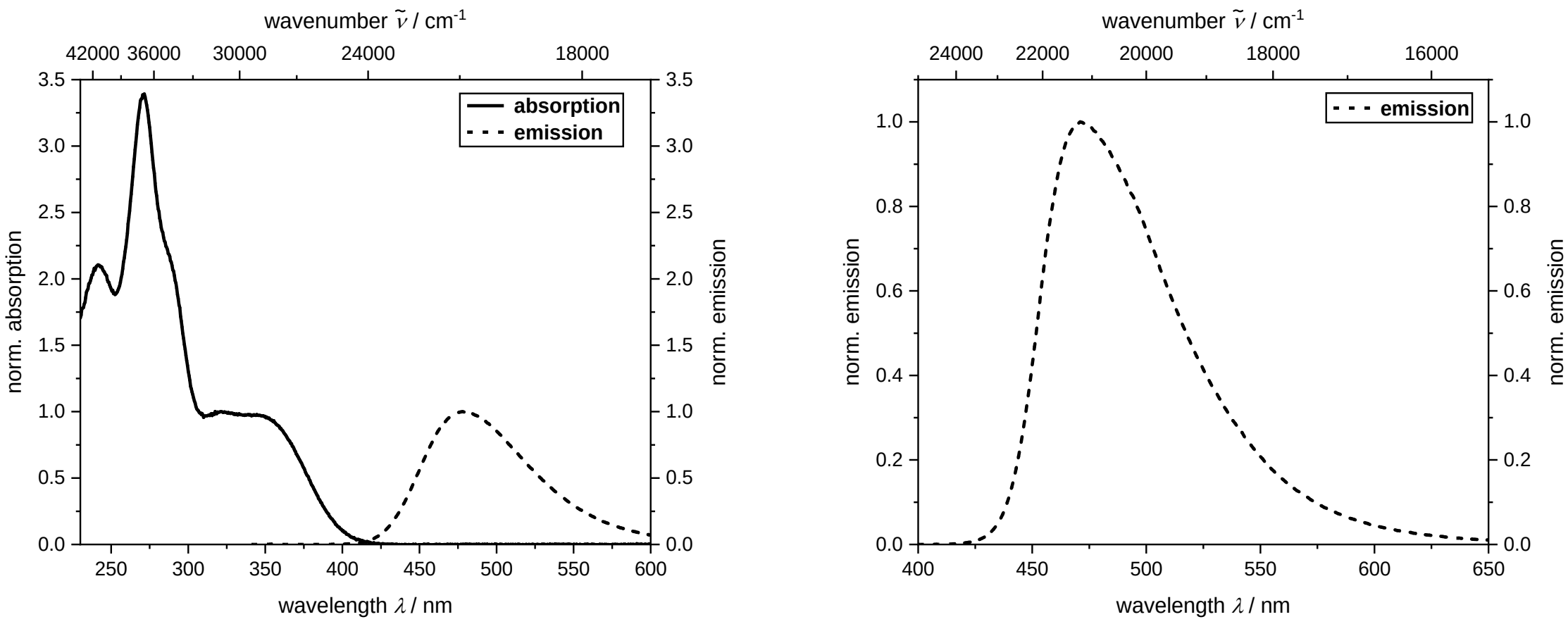

Figure S83. Left: normalized absorption (recorded in CH_2Cl_2, T = 293 K, $c = 10^{-5}$ M, solid line) and emission spectrum (recorded in CH_2Cl_2, T = 293 K, $c = 10^{-7}$ M, dashed line) of compound **5b**. Right: normalized emission spectrum (recorded in the solid state, T = 293 K, dashed line) of compound **5b**.

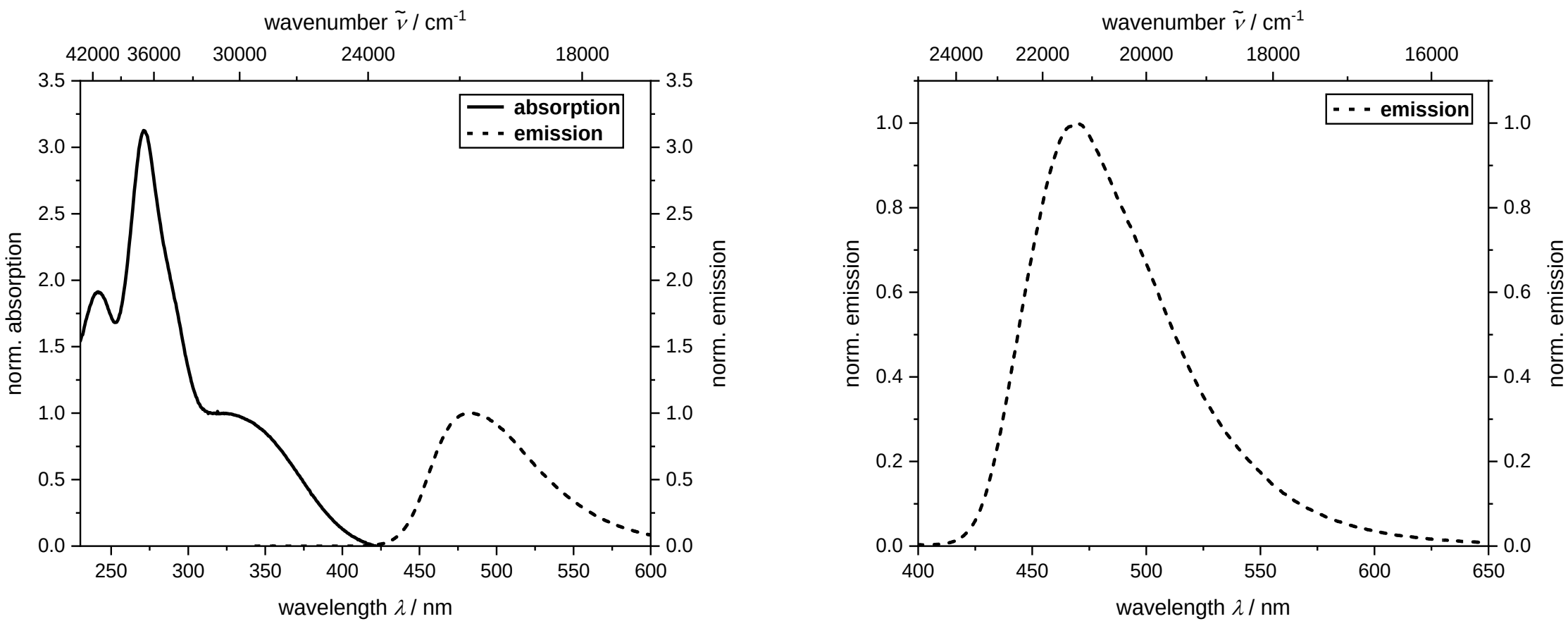

Figure S84. Left: normalized absorption (recorded in CH_2Cl_2, T = 293 K, c = 10^{-5} M, solid line) and emission spectrum (recorded in CH_2Cl_2, T = 293 K, c = 10^{-7} M, dashed line) of compound **5c**. Right: normalized emission spectrum (recorded in the solid state, T = 293 K, dashed line) of compound **5c**.

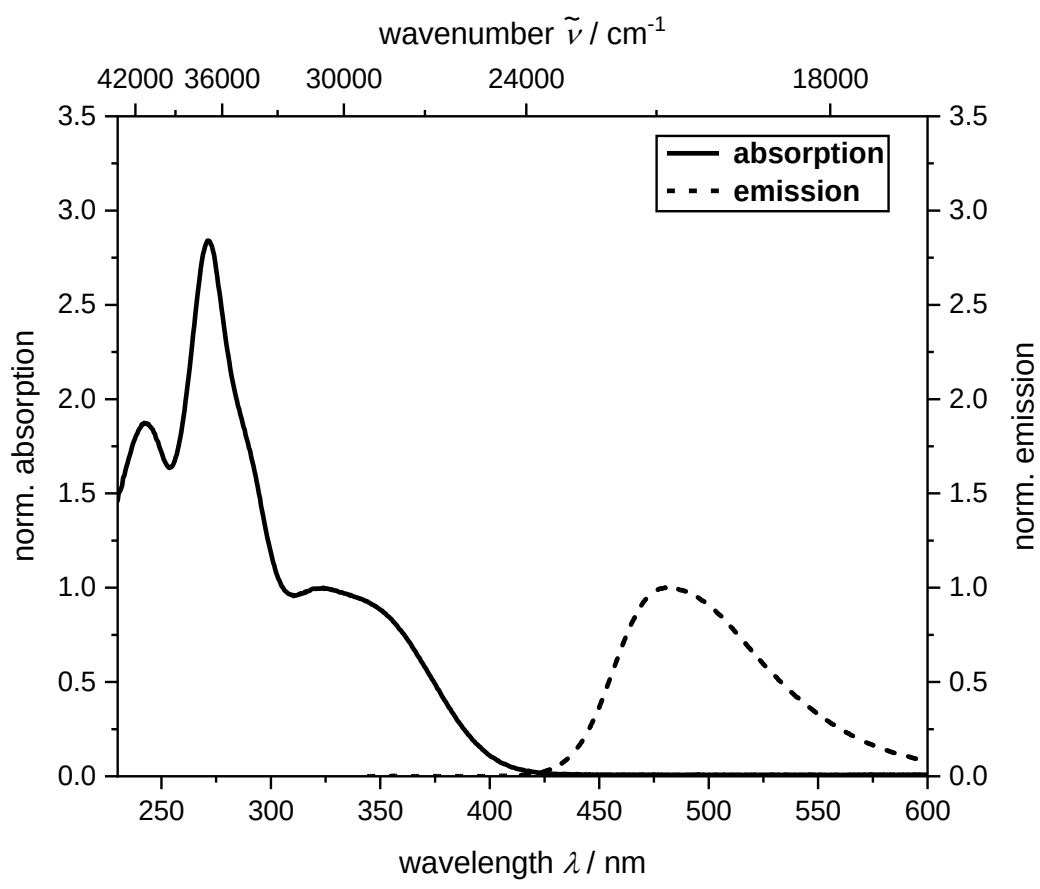

Figure S85. normalized absorption (recorded in CH_2Cl_2, T = 293 K, c = 10^{-5} M, solid line) and emission spectrum (recorded in CH_2Cl_2, T = 293 K, c = 10^{-7} M, dashed line) of compound **5d**.

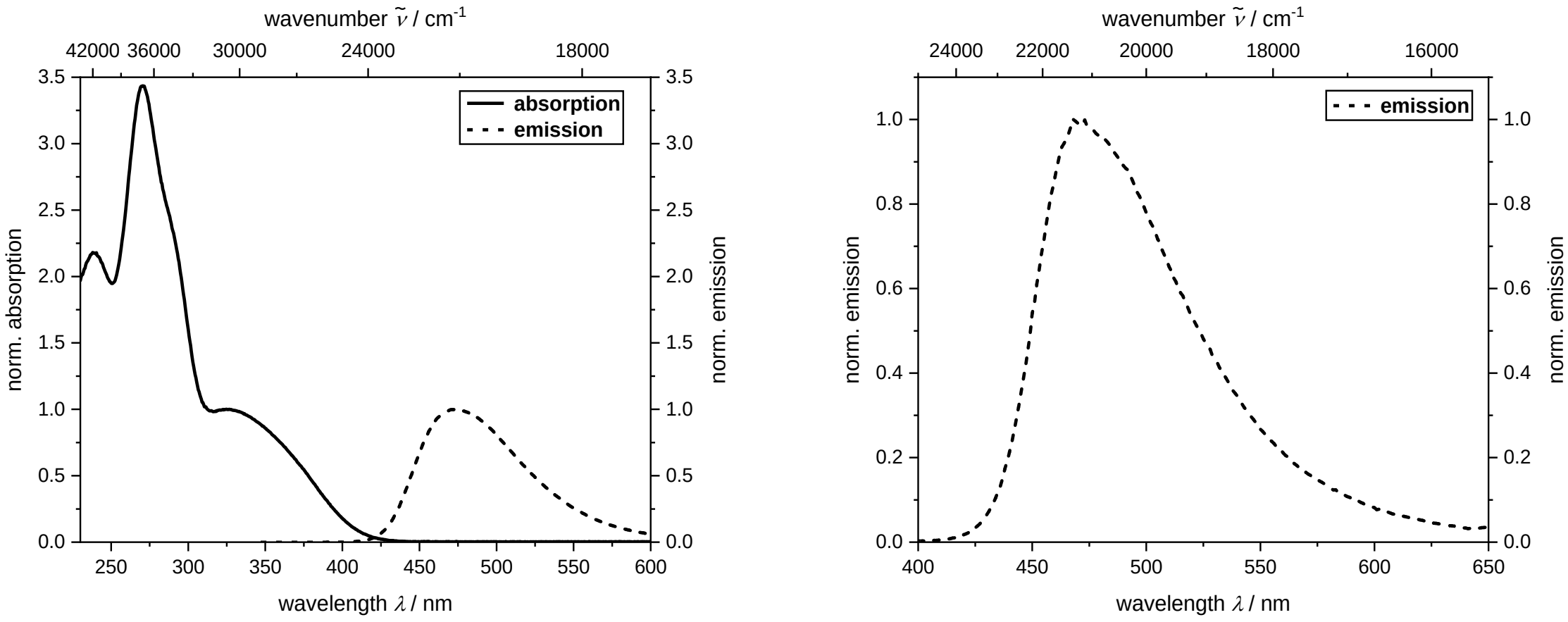

Figure S86. Left: normalized absorption (recorded in CH_2Cl_2, T = 293 K, c = 10^{-5} M, solid line) and emission spectrum (recorded in CH_2Cl_2, T = 293 K, c = 10^{-7} M, dashed line) of compound **5e**. Right: normalized emission spectrum (recorded in the solid state, T = 293 K, dashed line) of compound **5e**.

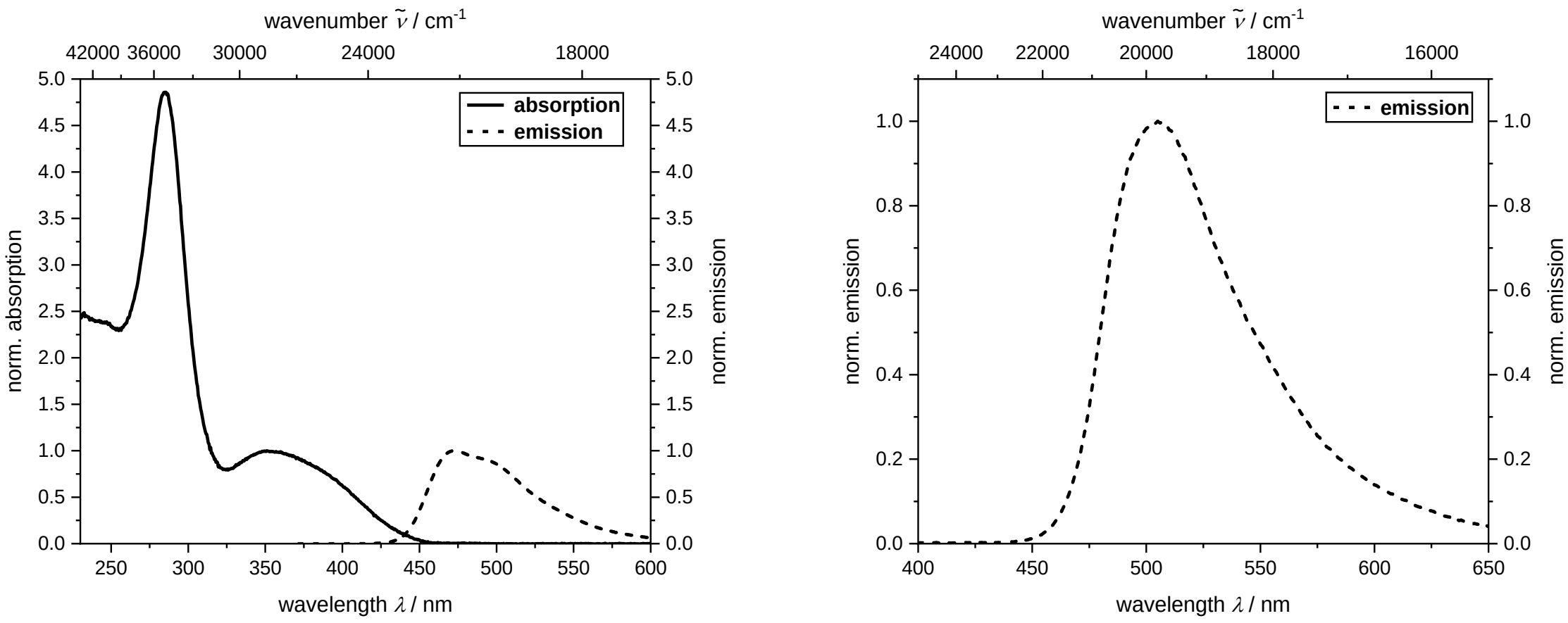

Figure S87. Left: normalized absorption (recorded in CH_2Cl_2, T = 293 K, c = 10^{-5} M, solid line) and emission spectrum (recorded in CH_2Cl_2, T = 293 K, c = 10^{-7} M, dashed line) of compound **5f**. Right: normalized emission spectrum (recorded in the solid state, T = 293 K, dashed line) of compound **5f**.

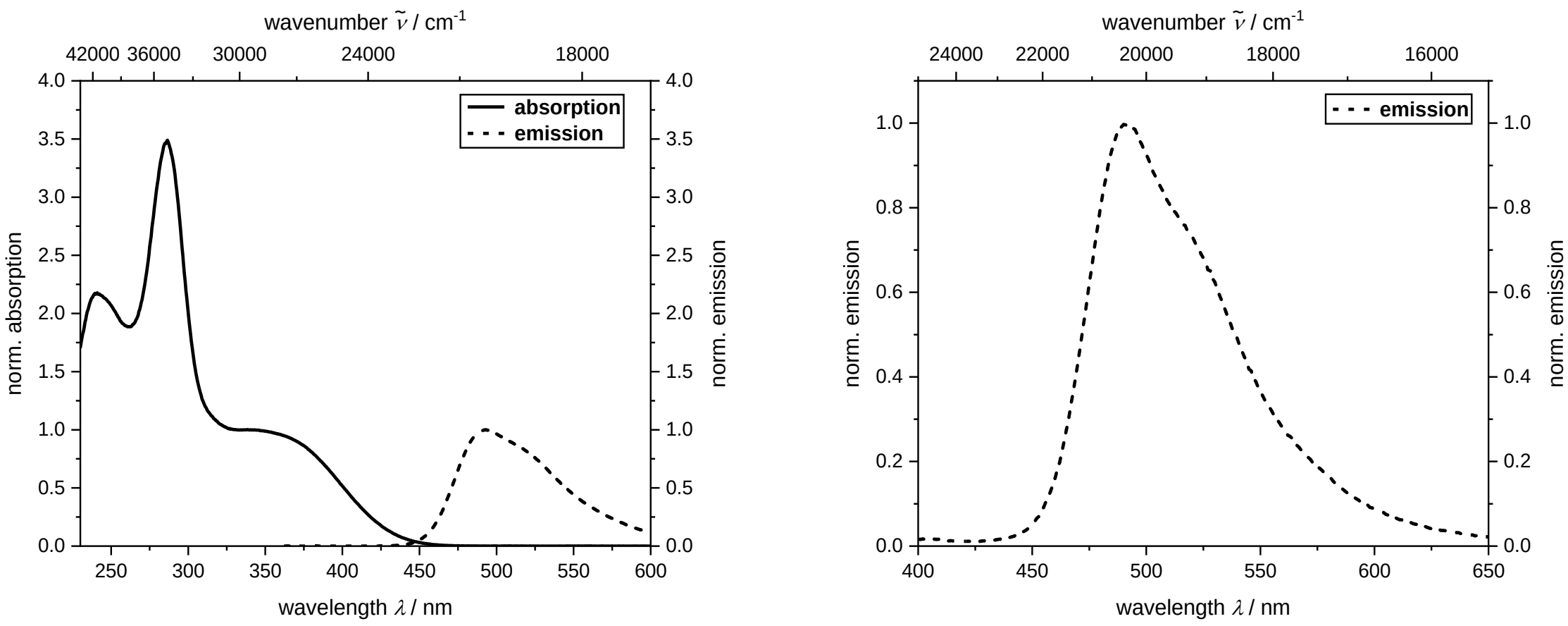

Figure S88. Left: normalized absorption (recorded in CH_2Cl_2, T = 293 K, c = 10^{-5} M, solid line) and emission spectrum (recorded in CH_2Cl_2, T = 293 K, c = 10^{-7} M, dashed line) of compound **5g**. Right: normalized emission spectrum (recorded in the solid state, T = 293 K, dashed line) of compound **5g**.

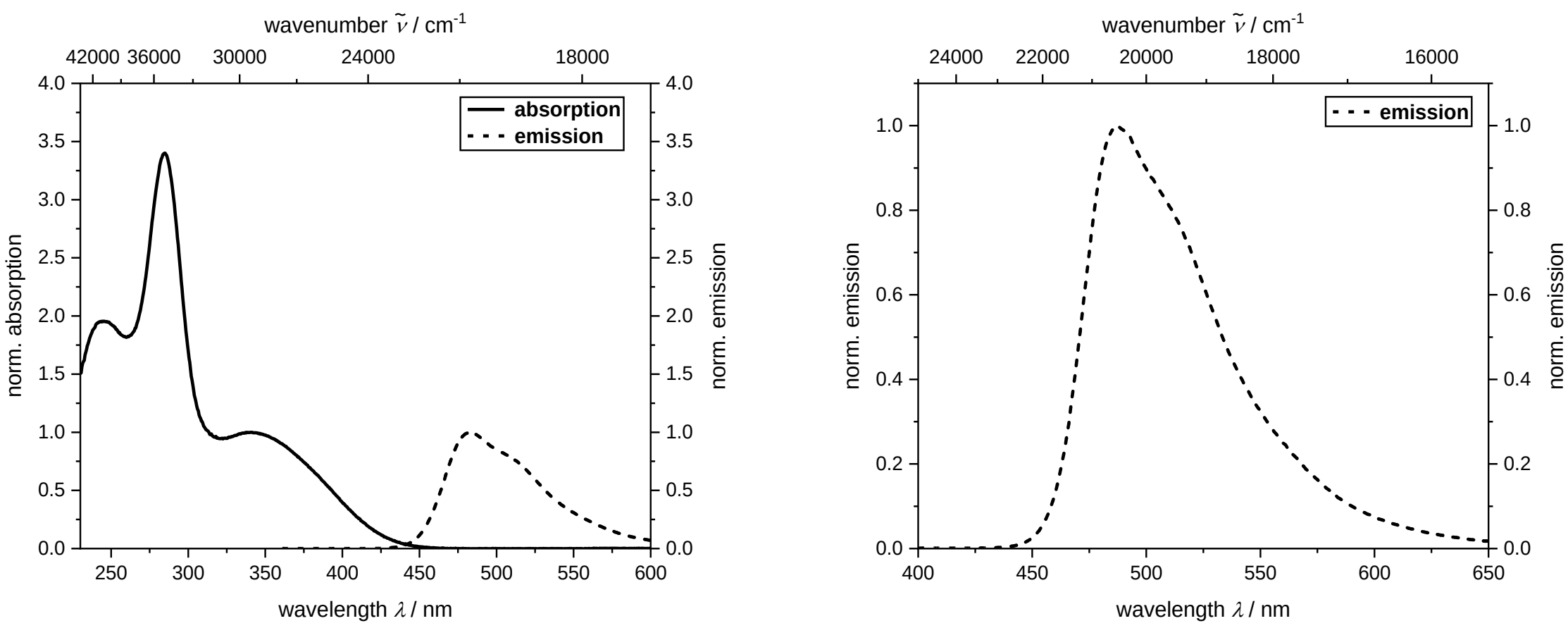

Figure S89. Left: normalized absorption (recorded in CH_2Cl_2, T = 293 K, c = 10^{-5} M, solid line) and emission spectrum (recorded in CH_2Cl_2, T = 293 K, c = 10^{-7} M, dashed line) of compound **5h**. Right: normalized emission spectrum (recorded in the solid state, T = 293 K, dashed line) of compound **5h**.

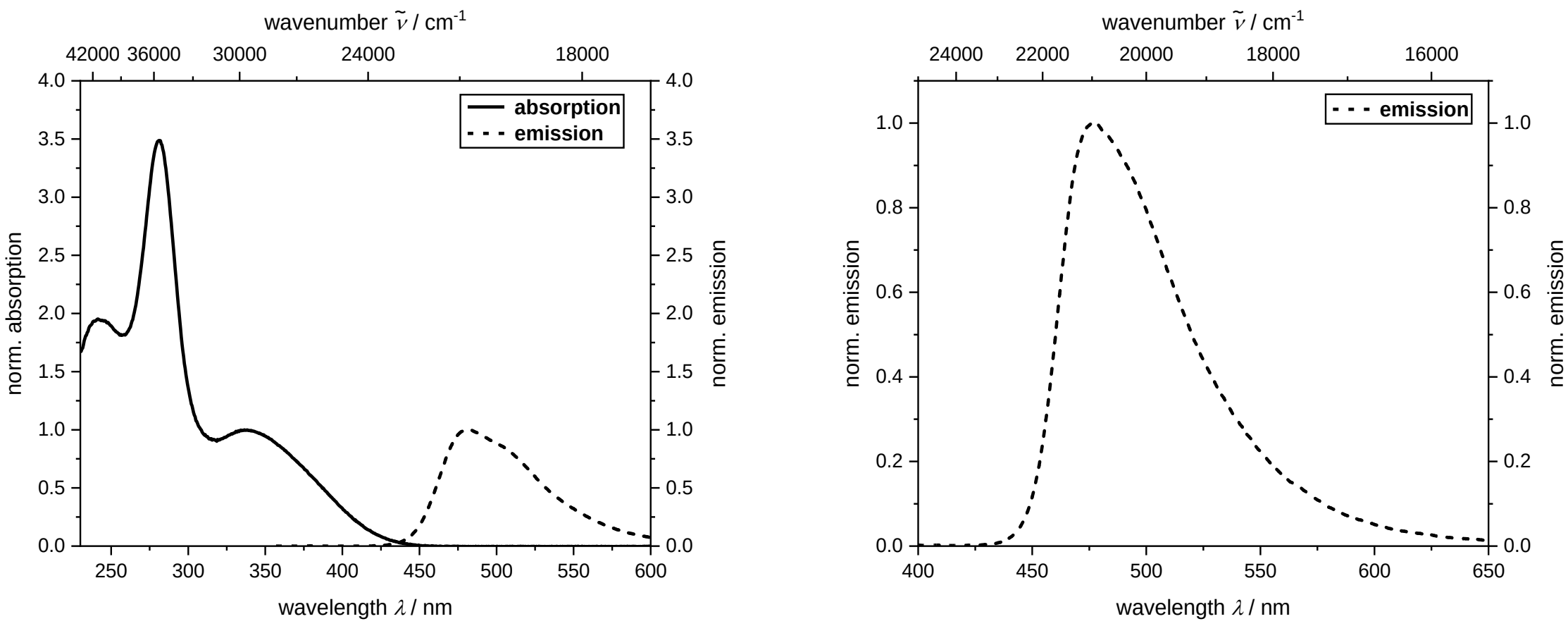

Figure S90. Left: normalized absorption (recorded in CH_2Cl_2, T = 293 K, c = 10^{-5} M, solid line) and emission spectrum (recorded in CH_2Cl_2, T = 293 K, c = 10^{-7} M, dashed line) of compound **5i**. Right: normalized emission spectrum (recorded in the solid state, T = 293 K, dashed line) of compound **5i**.

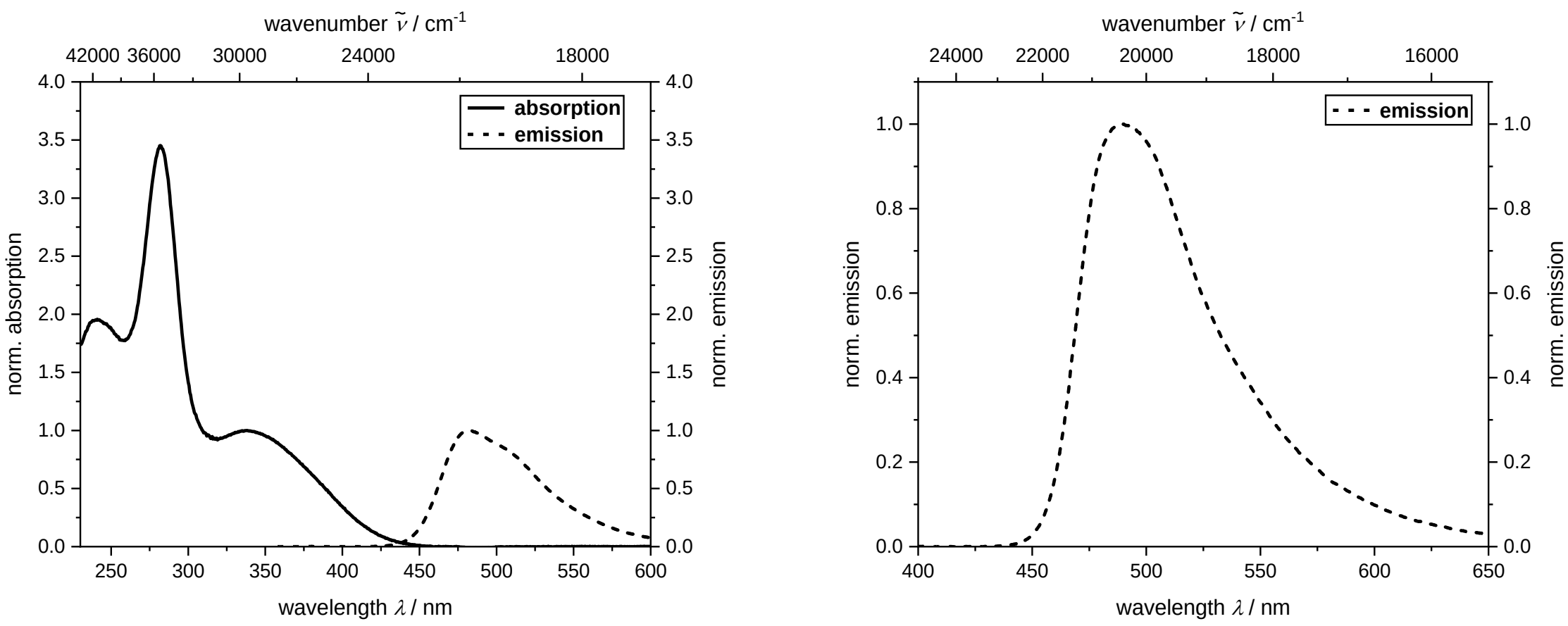

Figure S91. Left: normalized absorption (recorded in CH_2Cl_2, T = 293 K, c = 10^{-5} M, solid line) and emission spectrum (recorded in CH_2Cl_2, T = 293 K, c = 10^{-7} M, dashed line) of compound **5j**. Right: normalized emission spectrum (recorded in the solid state, T = 293 K, dashed line) of compound **5j**.

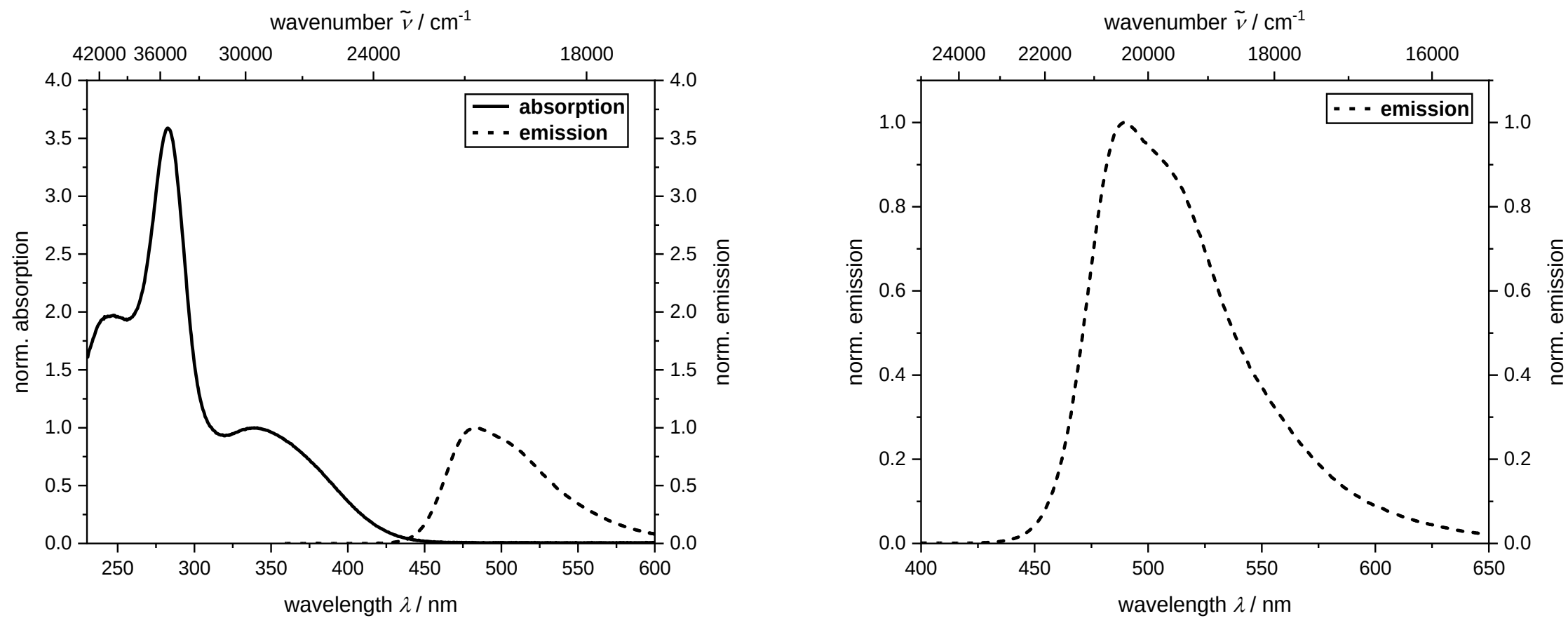

Figure S92. Left: normalized absorption (recorded in CH_2Cl_2, T = 293 K, c = 10^{-5} M, solid line) and emission spectrum (recorded in CH_2Cl_2, T = 293 K, c = 10^{-7} M, dashed line) of compound **5k**. Right: normalized emission spectrum (recorded in the solid state, T = 293 K, dashed line) of compound **5k**.

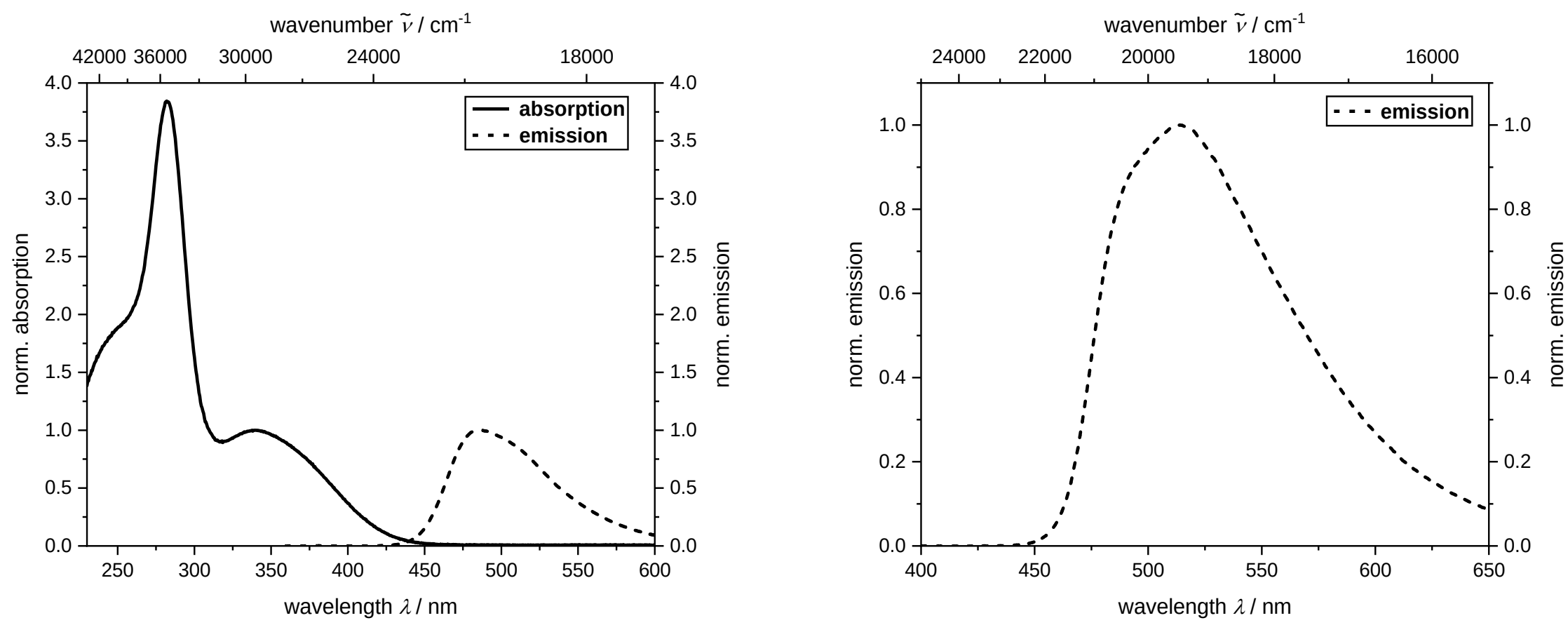

Figure S93. Left: normalized absorption (recorded in CH_2Cl_2, T = 293 K, c = 10^{-5} M, solid line) and emission spectrum (recorded in CH_2Cl_2, T = 293 K, c = 10^{-7} M, dashed line) of compound **5l**. Right: normalized emission spectrum (recorded in the solid state, T = 293 K, dashed line) of compound **5l**.

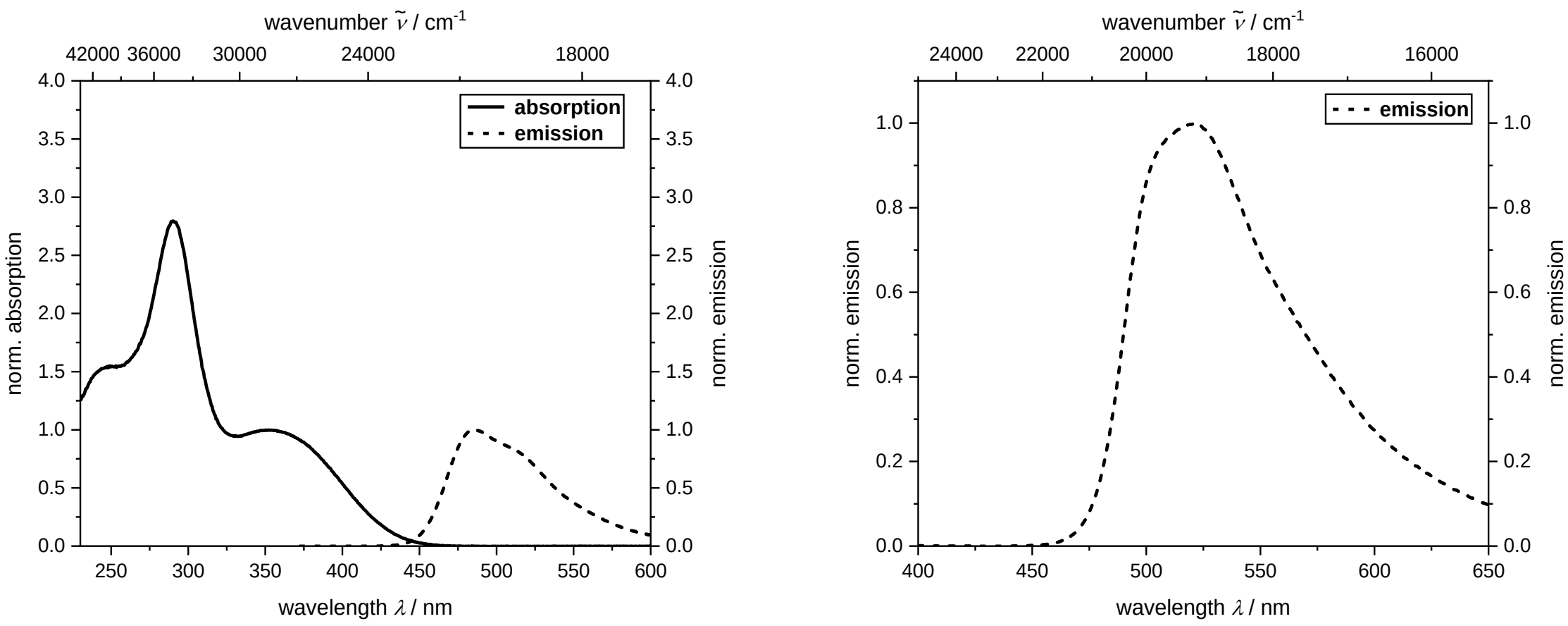

Figure S94. Left: normalized absorption (recorded in CH_2Cl_2, T = 293 K, c = 10^{-5} M, solid line) and emission spectrum (recorded in CH_2Cl_2, T = 293 K, c = 10^{-7} M, dashed line) of compound **5m**. Right: normalized emission spectrum (recorded in the solid state, T = 293 K, dashed line) of compound **5m**.

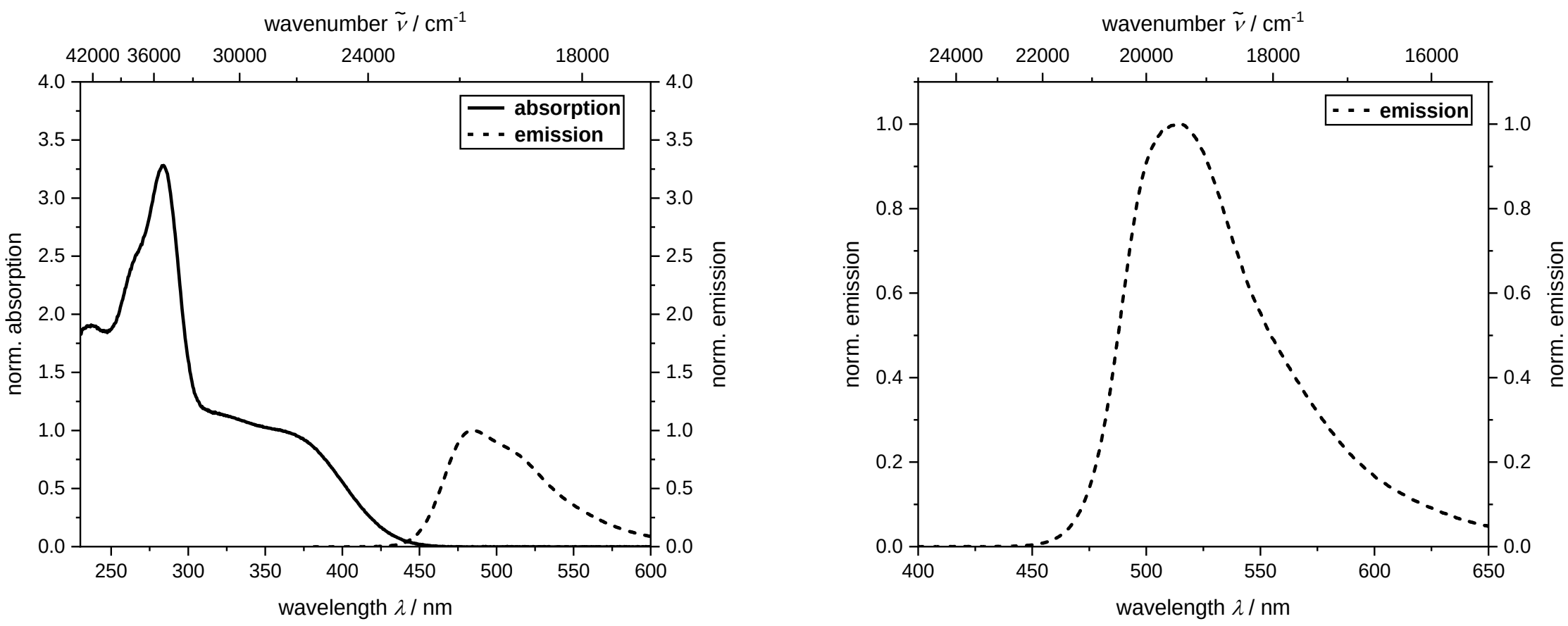

Figure S95. Left: normalized absorption (recorded in CH_2Cl_2, T = 293 K, c = 10^{-5} M, solid line) and emission spectrum (recorded in CH_2Cl_2, T = 293 K, c = 10^{-7} M, dashed line) of compound **5n**. Right: normalized emission spectrum (recorded in the solid state, T = 293 K, dashed line) of compound **5n**.

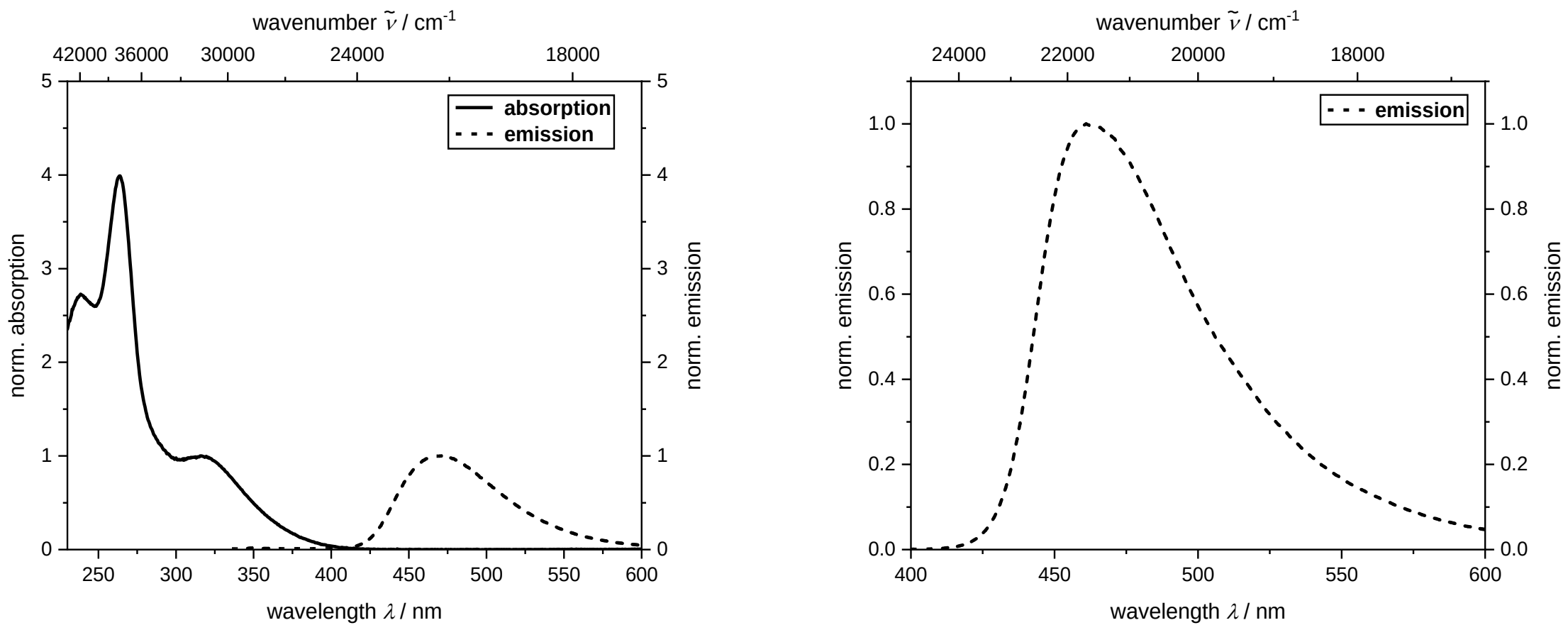

Figure S96. Left: normalized absorption (recorded in CH_2Cl_2, T = 293 K, c = 10^{-5} M, solid line) and emission spectrum (recorded in CH_2Cl_2, T = 293 K, c = 10^{-7} M, dashed line) of compound **5o**. Right: normalized emission spectrum (recorded in the solid state, T = 293 K, dashed line) of compound **5o**.

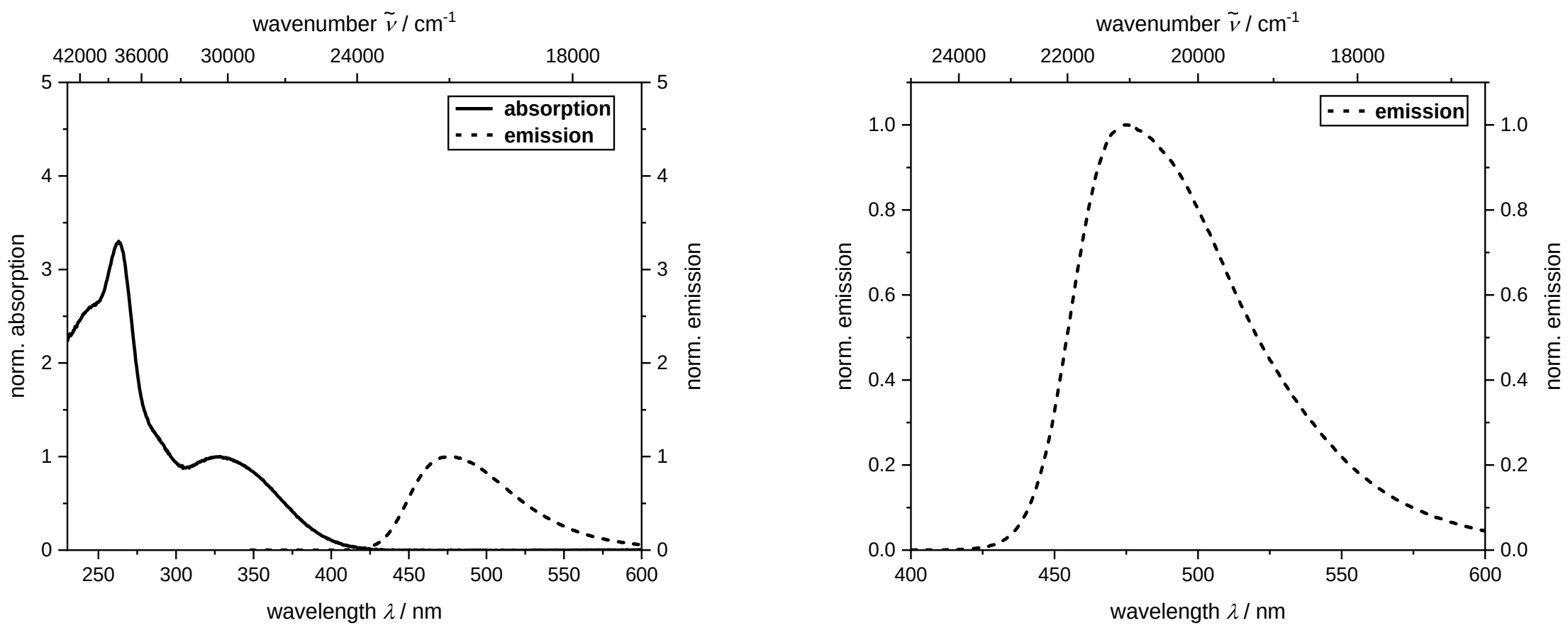

Figure S97. Left: normalized absorption (recorded in CH_2Cl_2, T = 293 K, c = 10^{-5} M, solid line) and emission spectrum (recorded in CH_2Cl_2, T = 293 K, c = 10^{-7} M, dashed line) of compound **5p**. Right: normalized emission spectrum (recorded in the solid state, T = 293 K, dashed line) of compound **5p**.

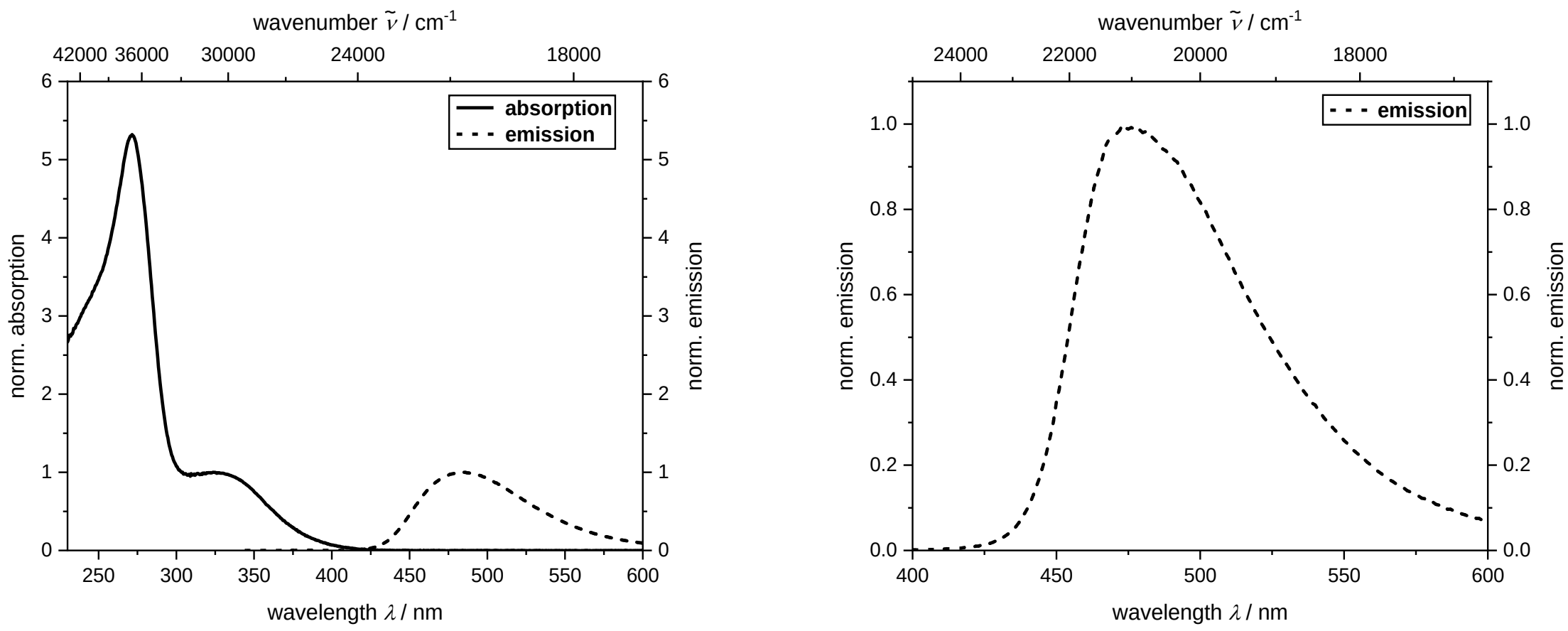

Figure S98. Left: normalized absorption (recorded in CH_2Cl_2, T = 293 K, c = 10^{-5} M, solid line) and emission spectrum (recorded in CH_2Cl_2, T = 293 K, c = 10^{-7} M, dashed line) of compound **5q**. Right: normalized emission spectrum (recorded in the solid state, T = 293 K, dashed line) of compound **5q**.

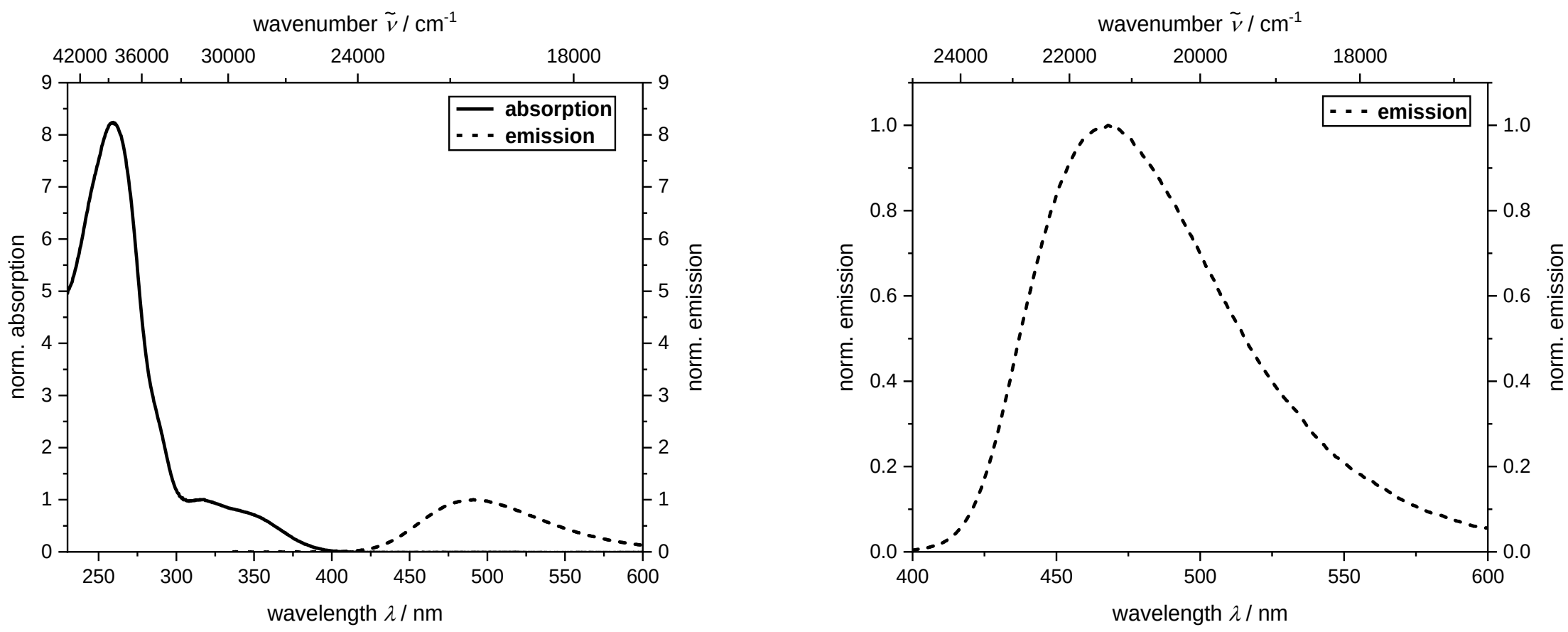

Figure S99. Left: normalized absorption (recorded in CH_2Cl_2, T = 293 K, c = 10^{-5} M, solid line) and emission spectrum (recorded in CH_2Cl_2, T = 293 K, c = 10^{-7} M, dashed line) of compound **5r**. Right: normalized emission spectrum (recorded in the solid state, T = 293 K, dashed line) of compound **5r**.

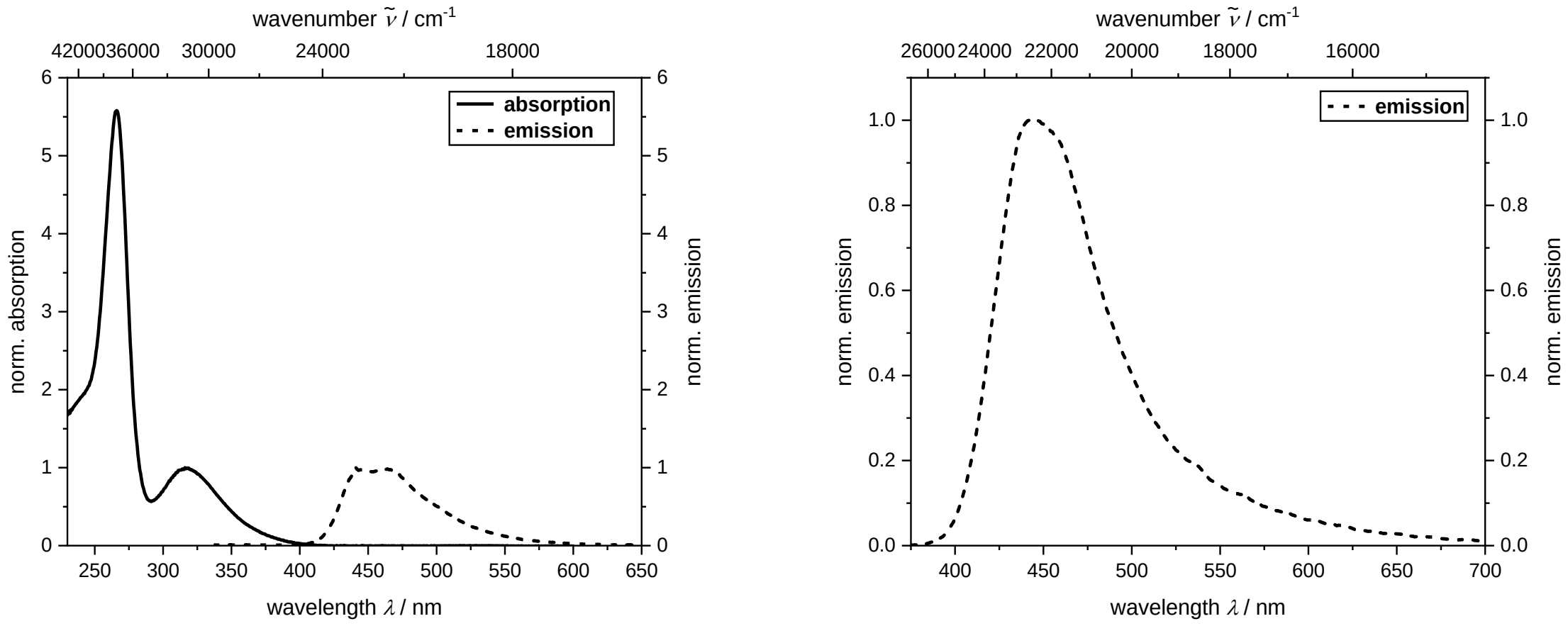

Figure S100. Left: normalized absorption (recorded in CH_2Cl_2, T = 293 K, c = 10^{-5} M, solid line) and emission spectrum (recorded in CH_2Cl_2, T = 293 K, c = 10^{-7} M, dashed line) of compound **6**. Right: normalized emission spectrum (recorded in the solid state, T = 293 K, dashed line) of compound **6**.

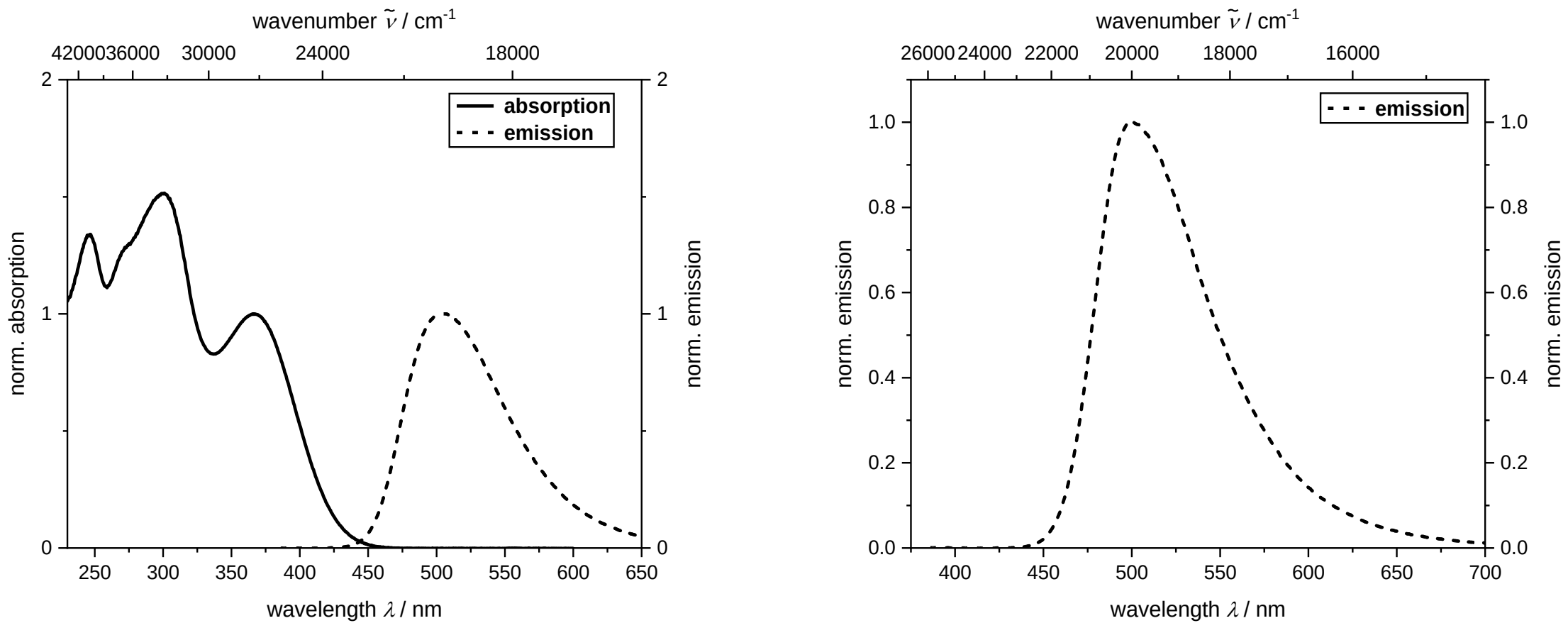

Figure S101. Left: normalized absorption (recorded in CH_2Cl_2, T = 293 K, c = 10^{-5} M, solid line) and emission spectrum (recorded in CH_2Cl_2, T = 293 K, c = 10^{-7} M, dashed line) of compound **7**. Right: normalized emission spectrum (recorded in the solid state, T = 293 K, dashed line) of compound **7**.

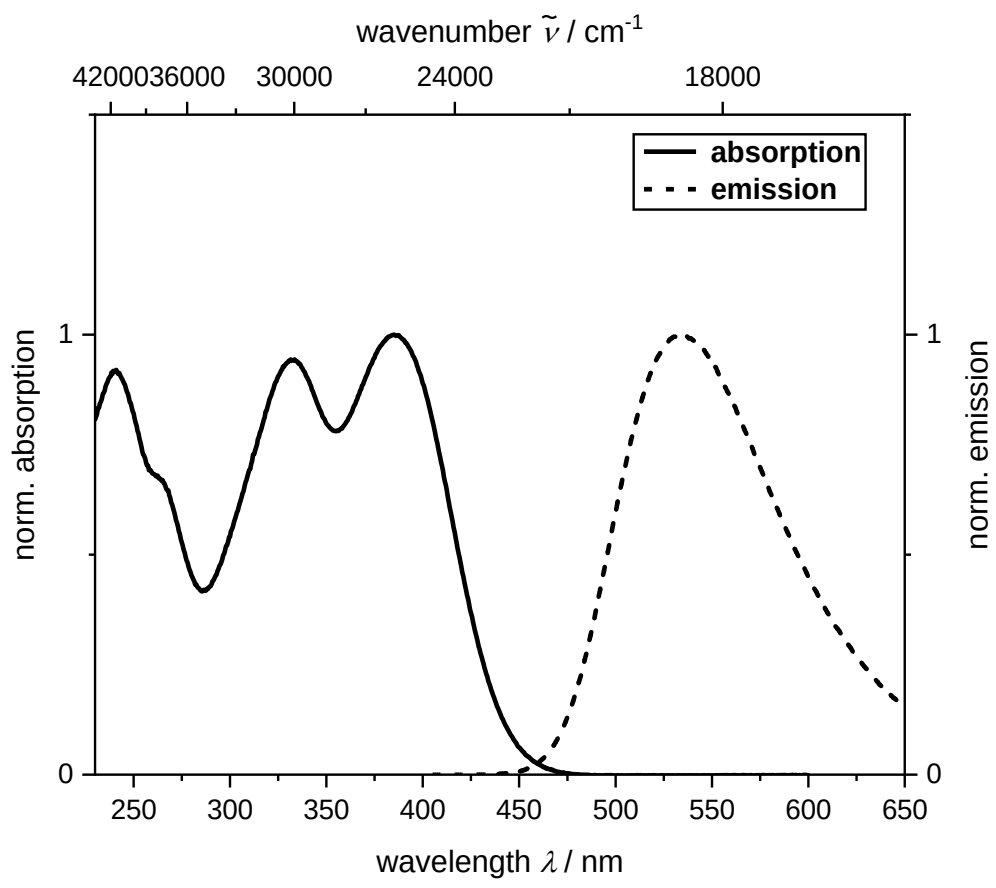

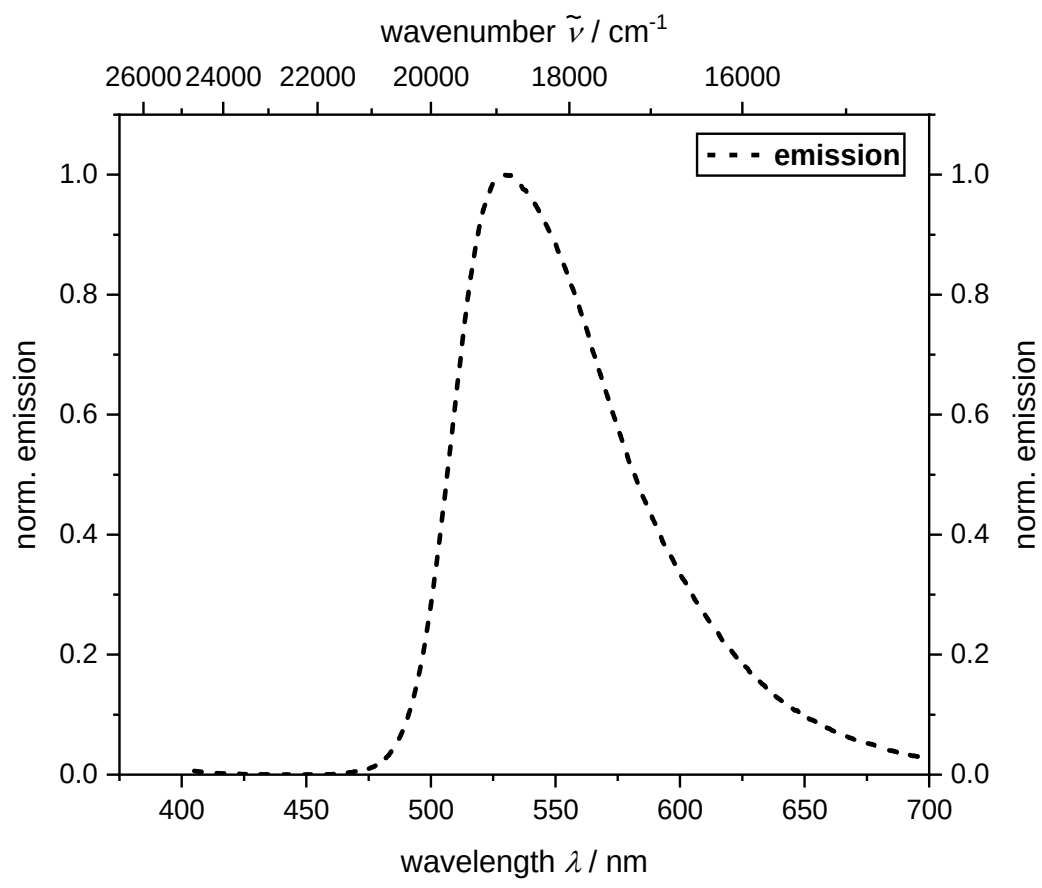

Figure S102. Left: normalized absorption (recorded in CH_2Cl_2, T = 293 K, c = 10^{-5} M, solid line) and emission spectrum (recorded in CH_2Cl_2, T = 293 K, c = 10^{-7} M, dashed line) of compound **8**. Right: normalized emission spectrum (recorded in the solid state, T = 293 K, dashed line) of compound **8**.

6. Data of quantum chemical calculations

6.1 Quantum chemical calculation data of tetrakis(4-(10-methyl-10*H*-phenothiazin-3-yl)phenyl)silane (5a)

6.1.1 Computed xyz-coordinates of compound 5a (cam-B3LYP/def2svp SMD CH_2Cl_2 GD3BJ)

0 1

C -3.23415 10.72737 -7.15012

C -4.41618 10.27984 -7.72913

C -4.67465 8.91612 -7.85222

C -3.73705 7.96786 -7.42442

C -2.54141 8.43212 -6.84673

C -2.30336 9.79479 -6.69700

N -3.96870 6.58675 -7.55268

C -3.55619 5.72241 -6.52697

C -2.34512 5.95111 -5.84978

S -1.28655 7.26162 -6.40176

C -4.31926 4.61613 -6.13513

C -3.87958 3.76312 -5.12857

C -2.68006 3.99221 -4.44825

C -1.92936 5.11466 -4.82245

C -2.21776 3.08821 -3.36621

C -2.50015 1.71667 -3.40063

C -2.07086 0.87059 -2.38303

C -1.34005 1.35435 -1.29038

C -1.05193 2.72759 -1.26586

C -1.48278 3.57795 -2.27730

Si -0.78817 0.23844 0.12143

C -5.02988 6.14698 -8.43413

C -10.25479 -1.37264 8.87802

C -10.40602 -0.03939 9.24195

C -9.35627 0.86322 9.08422

C -8.11729 0.44155 8.58566

C -7.97359 -0.91120 8.22757

C -9.03557 -1.80080 8.35701

N -7.03882 1.33088 8.43267

C -6.20247 1.20967 7.31150

C -5.85504 -0.06062 6.81911

S -6.38669 -1.50156 7.70350

C -5.69003 2.32678 6.64135

C -4.85201 2.18048 5.54094

C -4.51189 0.91911 5.04393

C -5.04287 -0.19869 5.70110

C -3.62604 0.76196 3.86360

C -3.63862 1.69615 2.82003

C -2.80950 1.54529 1.71286

C -1.93527 0.45699 1.59779

C -1.93110 -0.47876 2.64370

C -2.75376 -0.33028 3.75396

C -7.10648 2.61368 9.10107

C 2.21935 -11.72629 -4.02180

C 1.16550 -12.40200 -3.41714

C -0.01516 -11.73479 -3.09709

C -0.17751 -10.37839 -3.40525

C 0.89273 -9.70635 -4.02307

C 2.08120 -10.37028 -4.31090

N -1.36601 -9.68852 -3.10679

C -1.29520 -8.36375 -2.64941

C -0.33987 -7.47934 -3.18121

S 0.67619 -8.02117 -4.52907

C -2.15291 -7.87231 -1.65824

C -2.07473 -6.54979 -1.23485

C -1.12066 -5.67077 -1.75550

C -0.24530 -6.17000 -2.72909

C -1.03014 -4.26267 -1.29655

C -2.17588 -3.55636 -0.90431

C -2.08926 -2.23841 -0.47168

C -0.86005 -1.56353 -0.41870

C 0.28135 -2.27575 -0.80869

C 0.20088 -3.59678 -1.23786

C -2.54702 -10.46231 -2.78606

C 11.86015 3.10440 -0.59429

C 12.25297 2.51611 0.60256

C 11.34198 2.34716 1.64335

C 10.02056 2.79371 1.51806

C 9.63617 3.39401 0.30541

C 10.54188 3.53072 -0.74189

N 9.08780 2.64852 2.56041

C 7.75950 2.31621 2.25270

C 7.13708 2.85986 1.11484

S 8.00548 4.07313 0.15756

C 7.00883 1.44073 3.04633

C 5.68797 1.14136 2.73009

C 5.06976 1.67310 1.59479

C 5.82937 2.52880 0.78598

C 3.66518 1.34188 1.25030

C 2.71020 1.11433 2.24932

C 1.39428 0.79995 1.92451

C 0.97443 0.70169 0.59175

C 1.93376 0.93932 -0.40472

C 3.25059 1.24859 -0.08565

C 9.58210 2.34003 3.88587

H -3.03348 11.79529 -7.04590

H -5.16088 10.99626 -8.08269

H -5.61928 8.59199 -8.28659

H -1.37081 10.12472 -6.23378

H -5.27637 4.41455 -6.61383

H -4.50953 2.91689 -4.84930

H -0.97923 5.32592 -4.32858

H -3.04646 1.29729 -4.24757

H -2.30515 -0.19421 -2.45433

H -0.47978 3.15112 -0.43577

H -1.25798 4.64414 -2.21012

H -6.03699 6.24334 -7.99078

H -5.00130 6.73514 -9.35980

H -4.86803 5.09516 -8.70092

H -11.07861 -2.07940 8.99357

H -11.35716 0.31621 9.64408

H -9.50892 1.90732 9.35354

H -8.89650 -2.84251 8.05930

H -5.93955 3.33071 6.98133

H -4.45148 3.07812 5.06701

H -4.82407 -1.20315 5.33453

H -4.32384 2.54508 2.86118

H -2.85941 2.29154 0.91638

H -1.26930 -1.34805 2.59630

H -2.70648 -1.06892 4.55646

H -7.74533 3.34790 8.57859

H -7.49815 2.47441 10.11639

H -6.09587 3.03224 9.18678

H 3.14775 -12.24578 -4.26632

H 1.25920 -13.46297 -3.17546

H -0.81617 -12.28099 -2.60104

H 2.89908 -9.81938 -4.78065

H -2.89112 -8.52838 -1.19941

H -2.75746 -6.21044 -0.45405

H 0.50659 -5.51617 -3.17457

H -3.15354 -4.03955 -0.95402

H -3.00718 -1.72500 -0.17240

H 1.26304 -1.79779 -0.76903

H 1.11637 -4.12494 -1.51144

H -2.54863 -10.85270 -1.75280

H -3.43839 -9.83714 -2.92109

H -2.62507 -11.31063 -3.47750

H 12.57071 3.23032 -1.41331

H 13.28004 2.16829 0.73298

H 11.66851 1.86125 2.56172

H 10.21010 3.99038 -1.67555

H 7.45788 0.97513 3.92238

H 5.14163 0.44763 3.37144

H 5.38411 2.97903 -0.10311

H 2.99371 1.20576 3.29963

H 0.67947 0.63975 2.73510

H 1.65237 0.87955 -1.45972

H 3.97287 1.40378 -0.88938

H 9.85123 1.27685 4.01731

H 10.46933 2.95066 4.09493

H 8.81638 2.59757 4.62814

SCF Done: E(RCAM-B3LYP) = -5026.87374135 A.U. after 6 cycles

Zero-point correction= 1.126384 (Hartree/Particle)

Thermal correction to Energy= 1.195407

Thermal correction to Enthalpy= 1.196351

Thermal correction to Gibbs Free Energy= 1.006131

Sum of electronic and zero-point Energies= -5025.747357

Sum of electronic and thermal Energies= -5025.678334

Sum of electronic and thermal Enthalpies= -5025.677390

Sum of electronic and thermal Free Energies= -5025.867611

6.1.2 Computed excitation energies of compound 5a (cam-B3LYP/def2svp SMD CH_2Cl_2 GD3BJ)

Excitation energies and oscillator strengths:

Excited State 1: Singlet-A 3.9349 eV 315.08 nm f=0.4900 <S**2>=0.000

306 -> 311 0.24803

306 -> 313 -0.17983

307 -> 310 0.20936

307 -> 311 -0.19522

307 -> 312 0.14430

307 -> 316 -0.10442

308 -> 311 0.17205

308 -> 313 -0.18547

309 -> 310 0.20371

309 -> 311 -0.11218

309 -> 312 0.11433

This state for optimization and/or second-order correction.

Total Energy, E(TD-HF/TD-DFT) = -5026.72913466

Copying the excited state density for this state as the 1-particle RhoCI density.

Excited State 2: Singlet-A 3.9372 eV 314.90 nm f=0.3399 <S**2>=0.000

306 -> 311 0.16348

306 -> 312 -0.16517

306 -> 313 0.17044

307 -> 310 -0.18539

307 -> 311 -0.22523

307 -> 312 -0.12974

308 -> 310 -0.22872

308 -> 311 -0.11024

309 -> 311 0.10562

309 -> 312 -0.12431

309 -> 313 0.25172

Excited State 3: Singlet-A 3.9463 eV 314.17 nm f=0.4987 <S**2>=0.000

306 -> 312 0.22030

306 -> 313 0.14742

307 -> 310 -0.11356

307 -> 312 0.22512

307 -> 318 -0.10431

308 -> 310 0.16275

308 -> 311 -0.14198

308 -> 313 -0.21896

309 -> 310 0.20610

309 -> 311 0.16164

309 -> 312 -0.11749

309 -> 313 -0.12433

Excited State 4: Singlet-A 3.9808 eV 311.46 nm f=0.0104 <S**2>=0.000

306 -> 310 -0.27020

306 -> 316 0.11464

306 -> 318 -0.10647

307 -> 313 0.27028

308 -> 311 0.14588

308 -> 312 -0.24946

309 -> 311 -0.19844

309 -> 312 -0.19427

Excited State 5: Singlet-A 4.3971 eV 281.97 nm f=0.4796 <S**2>=0.000

306 -> 315 -0.24236

307 -> 314 0.11461

307 -> 315 -0.14622

307 -> 318 -0.10706

308 -> 315 0.28165

308 -> 316 -0.18212

309 -> 315 0.25644

309 -> 316 -0.13277

309 -> 317 -0.10014

Excited State 6: Singlet-A 4.4151 eV 280.82 nm f=0.4169 <S**2>=0.000

306 -> 314 -0.16102

306 -> 318 0.21288

307 -> 310 0.10412

307 -> 316 0.30938

308 -> 314 -0.12061

308 -> 317 0.14422

308 -> 318 0.15812

309 -> 316 0.21101

Excited State 7: Singlet-A 4.4174 eV 280.67 nm f=0.1840 <S**2>=0.000

306 -> 316 0.14143

306 -> 317 -0.25414

307 -> 317 0.28731

308 -> 315 0.15771

308 -> 316 0.18552

308 -> 317 0.10066

309 -> 314 -0.13422

309 -> 315 -0.15255

309 -> 316 -0.11229

309 -> 317 -0.13139

309 -> 318 0.15871

Excited State 8: Singlet-A 4.4346 eV 279.58 nm f=0.1239 <S**2>=0.000

306 -> 310 0.11009

306 -> 316 0.26048

307 -> 314 -0.14351

307 -> 315 -0.17697

307 -> 318 0.18565

308 -> 316 0.11453

308 -> 317 -0.22296

309 -> 317 0.26858

Excited State 9: Singlet-A 4.5824 eV 270.57 nm f=0.3082 <S**2>=0.000

306 -> 319 0.25973

307 -> 319 0.17117

307 -> 322 0.13498

308 -> 318 0.13127

308 -> 319 -0.25587

308 -> 320 0.10193

308 -> 321 0.15470

309 -> 318 0.12354

309 -> 319 -0.25533

309 -> 320 -0.10435

Excited State 10: Singlet-A 4.5879 eV 270.24 nm f=0.6451 <S**2>=0.000

306 -> 320 0.28356

307 -> 319 -0.17020

307 -> 320 -0.17640

307 -> 321 -0.18394

308 -> 320 0.19092

308 -> 321 -0.14877

308 -> 322 -0.14012

309 -> 322 0.20115

6.1.3 Computed emission energies of compound 5a (cam-B3LYP/def2svp SMD CH_2Cl_2 GD3BJ)

Excitation energies and oscillator strengths:

Excited State 1: Singlet-A 2.7911 eV 444.21 nm f=0.2075 <S**2>=0.000

309 -> 310 -0.61851

309 -> 311 0.12610

309 -> 315 -0.17712

309 -> 318 -0.11598

309 -> 323 0.10162

309 -> 324 0.10571

This state for optimization and/or second-order correction.

Total Energy, E(TD-HF/TD-DFT) = -5026.70489461

Copying the excited state density for this state as the 1-particle RhoCI density.

Excited State 2: Singlet-A 3.7656 eV 329.25 nm f=0.3906 <S**2>=0.000

309 -> 310 0.19153

309 -> 314 0.29728

309 -> 315 -0.44530

309 -> 317 -0.11471

309 -> 318 -0.19527

309 -> 329 0.20484

Excited State 3: Singlet-A 3.9492 eV 313.95 nm f=0.3233 <S**2>=0.000

306 -> 312 0.17385

306 -> 313 -0.18435

307 -> 311 0.10729

307 -> 312 0.15056

308 -> 311 0.35735

308 -> 313 0.32588

308 -> 317 -0.15341

308 -> 318 0.10292

308 -> 321 -0.14264

Excited State 4: Singlet-A 3.9635 eV 312.81 nm f=0.3730 <S**2>=0.000

306 -> 311 0.13920

306 -> 312 0.36798

306 -> 320 0.11474

307 -> 311 0.21890

307 -> 313 -0.26672

307 -> 316 0.13195

308 -> 311 -0.17039

308 -> 313 -0.13499

Excited State 5: Singlet-A 3.9960 eV 310.27 nm f=0.1193 <S**2>=0.000

306 -> 311 0.22552

306 -> 313 -0.19097

306 -> 316 0.13192

307 -> 311 -0.13197

307 -> 312 0.35682

307 -> 313 0.14045

307 -> 316 -0.13151

307 -> 318 -0.15549

307 -> 319 0.12001

307 -> 325 -0.10525

308 -> 313 -0.19544

Excited State 6: Singlet-A 4.2528 eV 291.54 nm f=0.2970 <S**2>=0.000

305 -> 310 -0.11900

309 -> 314 -0.13371

309 -> 323 0.20251

309 -> 324 0.22536

309 -> 327 -0.11196

309 -> 328 0.51306

corrected external emission excitation: -5026.756657 - -5026.855324 = 0.098667 = 2.684863 eV 461.79 nm

6.2 Quantum chemical calculation data of tetrakis(4-(10-isopropyl-10*H*-phenothiazin-3-yl)phenyl)silane (5b)

6.2.1 Computed xyz-coordinates of compound 5b (cam-B3LYP/def2svp SMD CH_2Cl_2 GD3BJ)

0 1

C -8.41204 -7.48816 -6.21049

C -8.95487 -6.53100 -7.06160

C -8.22518 -5.39795 -7.41223

C -6.91737 -5.20923 -6.94449

C -6.37469 -6.19464 -6.10069

C -7.12057 -7.30787 -5.72229

S -4.67481 -6.06489 -5.61592

N -6.15496 -4.08081 -7.29917

C -4.59887 -4.30362 -5.43563

C -5.31403 -3.49529 -6.33714

C -5.15949 -2.10887 -6.20944

C -4.30455 -1.56083 -5.26094

C -3.59725 -2.36631 -4.36258

C -3.77866 -3.75048 -4.45954

C -6.36845 -3.47867 -8.62787

C -7.36355 -2.31442 -8.68009

C -2.69552 -1.78199 -3.33976

C -1.97035 -0.61143 -3.60161

C -1.12640 -0.06430 -2.64184

C -0.96442 -0.66170 -1.38271

C -1.69495 -1.82942 -1.12634

C -2.54252 -2.37974 -2.08213

Si 0.23238 0.06218 -0.12232

C -5.04295 -3.13106 -9.29697

C 12.84675 -2.82472 0.88443

C 12.92076 -3.78299 -0.12125

C 11.99904 -3.78888 -1.16511

C 10.99557 -2.81342 -1.24623

C 10.94628 -1.83827 -0.23410

C 11.84666 -1.85759 0.82778

S 9.79310 -0.50111 -0.38938

N 10.06184 -2.78964 -2.29886

C 8.44825 -1.41179 -1.09818

C 8.73062 -2.43390 -2.02174

C 7.63591 -3.07362 -2.61709

C 6.33143 -2.68593 -2.33680

C 6.05212 -1.67089 -1.41590

C 7.14119 -1.05459 -0.78930

C 10.48429 -3.28177 -3.62263

C 10.17599 -4.75424 -3.91622

C 4.65866 -1.26662 -1.10597

C 3.65632 -1.32631 -2.08269

C 2.34879 -0.95125 -1.79158

C 1.98574 -0.49686 -0.51715

C 2.99319 -0.44145 0.45785

C 4.30090 -0.81693 0.17278

C 9.99967 -2.36043 -4.73689

C -4.43430 12.14584 -0.38169

C -3.41106 12.77459 0.32035

C -2.12975 12.22976 0.34702

C -1.83101 11.05507 -0.35714

C -2.87062 10.44146 -1.07846

C -4.15927 10.96865 -1.07251

S -2.49307 9.04402 -2.10105

N -0.54008 10.49421 -0.35877

C -1.29295 8.27952 -1.04474

C -0.40427 9.09570 -0.32232

C 0.58236 8.44874 0.43303

C 0.69797 7.06403 0.43719

C -0.19193 6.25372 -0.27533

C -1.20105 6.89345 -1.00381

C 0.61781 11.39978 -0.24732

C 1.15329 11.62478 1.17100

C -0.08356 4.77439 -0.25191

C 1.16279 4.14176 -0.16146

C 1.26163 2.75414 -0.13618

C 0.12403 1.93942 -0.20119

C -1.12131 2.57892 -0.29939

C -1.22589 3.96461 -0.32118

C 1.72260 11.00859 -1.22291

C -6.53376 -4.22106 10.07175

C -5.55347 -5.02064 10.65050

C -4.20382 -4.76974 10.41669

C -3.79854 -3.68912 9.62084

C -4.80022 -2.87753 9.05904

C -6.14935 -3.15360 9.26466

S -4.32357 -1.41486 8.17904

N -2.44081 -3.40611 9.38325

C -2.86444 -2.05101 7.40110

C -2.05154 -2.95278 8.11014

C -0.87528 -3.37933 7.48053

C -0.51092 -2.90118 6.22761

C -1.32134 -2.00528 5.52305

C -2.51593 -1.60419 6.13186

C -1.46164 -3.73108 10.43626

C -0.78579 -5.10162 10.31914

C -0.94020 -1.50195 4.17997

C -0.21631 -2.30253 3.28537

C 0.13990 -1.82694 2.02876

C -0.21407 -0.53656 1.60571

C -0.93314 0.25988 2.50632

C -1.28934 -0.21009 3.76622

C -0.44935 -2.60526 10.61833

H -8.98707 -8.37107 -5.92514

H -9.96799 -6.65272 -7.45097

H -8.68464 -4.65081 -8.05752

H -6.67259 -8.04769 -5.05508

H -5.71960 -1.43911 -6.85602

H -4.22019 -0.47457 -5.19925

H -3.24309 -4.42108 -3.78511

H -6.80639 -4.29668 -9.21509

H -7.73756 -2.20050 -9.70887

H -8.22769 -2.48650 -8.02334

H -6.90856 -1.35542 -8.39608

H -2.05371 -0.12998 -4.57791

H -0.57918 0.85000 -2.88672

H -1.61394 -2.32152 -0.15418

H -3.11193 -3.27824 -1.83640

H -5.22554 -2.83481 -10.34056

H -4.36945 -4.00040 -9.29596

H -4.52947 -2.29666 -8.79929

H 13.56113 -2.82607 1.70984

H 13.69621 -4.55156 -0.09209

H 12.06368 -4.56719 -1.92399

H 11.77146 -1.09251 1.60376

H 7.79921 -3.89496 -3.30901

H 5.51276 -3.21772 -2.82434

H 6.97651 -0.25151 -0.06880

H 11.57776 -3.18500 -3.59692

H 10.85050 -5.11763 -4.70628

H 10.32191 -5.38687 -3.02963

H 9.14902 -4.90825 -4.27563

H 3.90446 -1.65595 -3.09337

H 1.59807 -1.01299 -2.58317

H 2.75372 -0.10927 1.47163

H 5.05074 -0.78159 0.96540

H 10.46202 -2.66219 -5.68838

H 10.28380 -1.31870 -4.52848

H 8.90964 -2.40034 -4.86963

H -5.44212 12.56509 -0.39359

H -3.60864 13.69636 0.87186

H -1.35468 12.72778 0.92753

H -4.94583 10.45860 -1.63311

H 1.27205 9.03121 1.03752

H 1.48031 6.60745 1.04581

H -1.91501 6.30579 -1.58365

H 0.22593 12.36454 -0.59566

H 1.71694 12.56951 1.20241

H 0.34143 11.69054 1.90864

H 1.84014 10.83040 1.49444

H 2.07421 4.74205 -0.13270

H 2.25408 2.30110 -0.07488

H -2.03742 1.98483 -0.35784

H -2.21430 4.42493 -0.37601

H 2.49909 11.78782 -1.22865

H 1.32185 10.91103 -2.24235

H 2.20569 10.06002 -0.95032

H -7.59244 -4.42367 10.24430

H -5.83568 -5.86507 11.28307

H -3.45818 -5.42859 10.85913

H -6.90110 -2.50995 8.80231

H -0.22300 -4.09293 7.97602

H 0.43243 -3.23772 5.79469

H -3.19793 -0.93140 5.60912

H -2.06627 -3.75297 11.35260

H -0.40182 -5.40413 11.30520

H -1.48788 -5.87509 9.97802

H 0.07019 -5.09414 9.63016

H 0.05930 -3.31985 3.56954

H 0.70668 -2.48352 1.36296

H -1.21679 1.27786 2.22901

H -1.83035 0.44906 4.44782

H 0.16351 -2.80429 11.50993

H -0.96221 -1.64255 10.75799

H 0.23156 -2.51194 9.76100

SCF Done: E(RCAM-B3LYP) = -5340.97216078 A.U. after 1 cycles

Zero-point correction= 1.351163 (Hartree/Particle)

Thermal correction to Energy= 1.431921

Thermal correction to Enthalpy= 1.432865

Thermal correction to Gibbs Free Energy= 1.219420

Sum of electronic and zero-point Energies= -5339.620998

Sum of electronic and thermal Energies= -5339.540240

Sum of electronic and thermal Enthalpies= -5339.539296

Sum of electronic and thermal Free Energies= -5339.752740

6.2.2 Computed excitation energies of compound 5b (cam-B3LYP/def2svp SMD CH_2Cl_2 GD3BJ)

Excitation energies and oscillator strengths:

Excited State 1: Singlet-A 3.9295 eV 315.52 nm f=0.5313 <S**2>=0.000

338 -> 343 0.24969

338 -> 345 -0.24034

339 -> 342 0.26884

339 -> 343 0.24255

339 -> 344 -0.13012

339 -> 350 -0.11428

340 -> 342 0.14588

340 -> 343 0.15707

340 -> 344 0.17282

This state for optimization and/or second-order correction.

Total Energy, E(TD-HF/TD-DFT) = -5340.82775513

Copying the excited state density for this state as the 1-particle RhoCI density.

Excited State 2: Singlet-A 3.9347 eV 315.11 nm f=0.4570 <S**2>=0.000

338 -> 344 0.30219

339 -> 342 0.13860

339 -> 344 -0.23751

339 -> 345 0.10095

340 -> 342 -0.27000

340 -> 344 -0.10707

340 -> 345 -0.27872

340 -> 354 -0.10612

Excited State 3: Singlet-A 3.9403 eV 314.65 nm f=0.4012 <S**2>=0.000

338 -> 342 -0.13582

338 -> 343 -0.11605

338 -> 345 -0.12981

339 -> 343 -0.12930

339 -> 345 0.14778

340 -> 344 0.20263

341 -> 342 -0.26030

341 -> 343 0.26954

341 -> 345 0.23955

341 -> 346 -0.11844

341 -> 351 0.10431

Excited State 4: Singlet-A 3.9775 eV 311.72 nm f=0.0401 <S**2>=0.000

338 -> 342 0.25095

339 -> 343 0.16572

339 -> 345 -0.16721

340 -> 344 -0.24628

341 -> 342 -0.12181

341 -> 343 0.23524

341 -> 345 0.23321

341 -> 351 0.10914

Excited State 5: Singlet-A 4.3724 eV 283.56 nm f=0.2812 <S**2>=0.000

341 -> 342 -0.13512

341 -> 343 0.13210

341 -> 346 0.23202

341 -> 347 -0.14806

341 -> 348 0.40615

341 -> 349 -0.14311

341 -> 350 0.19406

341 -> 353 0.13812

Excited State 6: Singlet-A 4.3991 eV 281.84 nm f=0.3068 <S**2>=0.000

338 -> 346 -0.14298

338 -> 348 0.15357

338 -> 349 -0.16529

338 -> 350 -0.17860

339 -> 347 0.22102

339 -> 348 0.16829

340 -> 347 0.26459

340 -> 349 0.20999

340 -> 350 0.10365

340 -> 354 0.13101

Excited State 7: Singlet-A 4.4018 eV 281.67 nm f=0.2550 <S**2>=0.000

338 -> 347 -0.29418

339 -> 346 0.13986

339 -> 348 -0.24513

339 -> 349 -0.17215

339 -> 350 0.17664

340 -> 349 0.29691

340 -> 354 0.10110

Excited State 8: Singlet-A 4.4135 eV 280.92 nm f=0.0236 <S**2>=0.000

338 -> 342 -0.10325

338 -> 348 0.20905

338 -> 349 0.22274

339 -> 347 0.25592

339 -> 349 -0.24574

340 -> 344 0.10103

340 -> 346 -0.15367

340 -> 347 -0.12519

340 -> 348 0.14008

340 -> 350 -0.19070

Excited State 9: Singlet-A 4.5480 eV 272.61 nm f=0.2767 <S**2>=0.000

338 -> 351 -0.14493

340 -> 353 -0.10591

341 -> 348 0.11664

341 -> 350 0.22986

341 -> 351 0.43783

341 -> 352 0.11830

341 -> 353 -0.22180

Excited State 10: Singlet-A 4.5540 eV 272.25 nm f=0.5044 <S**2>=0.000

338 -> 351 0.14884

338 -> 352 -0.12169

338 -> 353 0.27135

338 -> 354 -0.11079

339 -> 351 0.20507

339 -> 353 0.24779

339 -> 354 0.12782

340 -> 350 -0.10312

340 -> 352 -0.23252

340 -> 354 0.15327

6.2.3 Computed emission energies of compound 5b (cam-B3LYP/def2svp SMD CH_2Cl_2 GD3BJ)

Excitation energies and oscillator strengths:

Excited State 1: Singlet-A 2.8132 eV 440.72 nm f=0.2300 <S**2>=0.000

341 -> 342 0.61781

341 -> 343 -0.12802

341 -> 346 -0.12691

341 -> 354 -0.12386

341 -> 355 0.14894

This state for optimization and/or second-order correction.

Total Energy, E(TD-HF/TD-DFT) = -5340.79934895

Copying the excited state density for this state as the 1-particle RhoCI density.

Excited State 2: Singlet-A 3.7713 eV 328.76 nm f=0.3235 <S**2>=0.000

341 -> 342 0.21350

341 -> 346 0.34117

341 -> 347 -0.10223

341 -> 348 0.32520

341 -> 350 0.24351

341 -> 352 0.12469

341 -> 354 0.15299

341 -> 355 -0.12748

341 -> 361 -0.19057

Excited State 3: Singlet-A 3.9384 eV 314.81 nm f=0.4045 <S**2>=0.000

338 -> 343 -0.14503

338 -> 344 0.14841

338 -> 345 -0.14067

339 -> 343 -0.13995

339 -> 344 -0.10463

339 -> 345 -0.15860

340 -> 343 0.21974

340 -> 344 0.37356

340 -> 345 -0.18845

340 -> 349 -0.10380

340 -> 350 0.10662

340 -> 353 -0.11468

Excited State 4: Singlet-A 3.9439 eV 314.37 nm f=0.4202 <S**2>=0.000

338 -> 344 0.17444

338 -> 345 0.34452

339 -> 343 0.35169

339 -> 344 -0.19925

339 -> 347 -0.11052

339 -> 351 -0.10876

339 -> 357 0.10076

340 -> 345 -0.13501

Excited State 5: Singlet-A 3.9780 eV 311.67 nm f=0.0726 <S**2>=0.000

338 -> 343 0.32491

338 -> 347 -0.10161

339 -> 345 0.33215

340 -> 344 0.27642

Excited State 6: Singlet-A 4.2376 eV 292.58 nm f=0.1840 <S**2>=0.000

341 -> 354 0.12003

341 -> 355 -0.19965

341 -> 356 -0.12120

341 -> 360 0.58949

corrected external emission excitation: -5340.853677 - -5340.953209 = 0.099532 = 2.708401 eV 457.78 nm

6.3 Quantum chemical calculation data of tetrakis(4-(10-ethyl-10*H*-phenothiazin-3-yl)phenyl)silane (5c/5d)

6.3.1 Computed xyz-coordinates of compounds 5c/5d (cam-B3LYP/def2svp SMD CH_2Cl_2 GD3BJ)

0 1

C -8.14689 -10.05157 -2.13684

C -7.45581 -10.70061 -3.15198

C -6.44416 -10.04880 -3.85310

C -6.10531 -8.71486 -3.57850

C -6.84197 -8.05992 -2.57229

C -7.82642 -8.72776 -1.84928

N -5.08350 -8.05372 -4.28442

C -4.36295 -6.99255 -3.71415

C -4.92571 -6.17281 -2.71654

S -6.63897 -6.32365 -2.30332

C -3.05492 -6.68336 -4.11669

C -2.34655 -5.63001 -3.54960

C -2.89234 -4.84513 -2.53169

C -4.19756 -5.14803 -2.12588

C -2.13246 -3.73510 -1.90733

C -1.20779 -2.98543 -2.64831

C -0.50039 -1.94248 -2.06213

C -0.68058 -1.60186 -0.71274

C -1.60782 -2.35271 0.02125

C -2.31841 -3.39779 -0.56045

Si 0.31828 -0.20884 0.06527

C -4.56607 -8.67077 -5.50339

C -3.49044 -9.73760 -5.31685

C 13.39525 -0.85234 1.24678

C 13.54038 -2.23277 1.29930

C 12.45276 -3.05169 1.59295

C 11.18549 -2.51506 1.86876

C 11.06479 -1.11177 1.84792

C 12.14636 -0.30006 1.51719

N 10.08551 -3.34010 2.16532

C 8.76745 -2.93125 1.90659

C 8.40847 -1.56976 1.88925

S 9.57103 -0.33437 2.38923

C 7.73915 -3.85521 1.66693

C 6.43649 -3.44615 1.40544

C 6.09248 -2.09396 1.34761

C 7.11225 -1.16694 1.59238

C 4.70880 -1.65278 1.04725

C 3.89511 -2.37840 0.16788

C 2.59670 -1.96503 -0.11291

C 2.05591 -0.81045 0.46734

C 2.87443 -0.08997 1.35085

C 4.17239 -0.49831 1.63478

C 10.33142 -4.75039 2.45595

C 10.46902 -5.67418 1.24887

C -2.85684 8.76359 -9.00628

C -1.97090 9.76567 -8.63165

C -0.86945 9.47775 -7.82928

C -0.60286 8.17059 -7.39363

C -1.49114 7.16080 -7.81244

C -2.61080 7.46020 -8.58370

N 0.50918 7.87617 -6.58378

C 0.50870 6.78234 -5.70427

C -0.27691 5.63973 -5.95088

S -1.12402 5.46001 -7.49338

C 1.29887 6.76326 -4.54525

C 1.29403 5.67796 -3.67649

C 0.48170 4.56458 -3.90145

C -0.30767 4.57636 -5.05770

C 0.45291 3.41669 -2.96284

C 1.59580 3.03942 -2.24599

C 1.56545 1.96554 -1.36208

C 0.39663 1.22118 -1.15632

C -0.74277 1.59830 -1.88370

C -0.71914 2.67323 -2.76449

C 1.55459 8.88652 -6.43890

C 1.31184 9.96728 -5.38852

C -7.26619 5.87700 8.93584

C -6.56285 5.54339 10.08623

C -5.64054 4.49975 10.07862

C -5.40739 3.73723 8.92374

C -6.15655 4.06481 7.77667

C -7.04964 5.13268 7.77956

N -4.47603 2.68290 8.91250

C -3.82703 2.28718 7.73240

C -4.41664 2.47944 6.46782

S -6.09264 3.03110 6.34286

C -2.56818 1.66787 7.75058

C -1.92976 1.27745 6.57881

C -2.49859 1.50729 5.32440

C -3.75516 2.12404 5.29926

C -1.81284 1.11312 4.06994

C -0.98804 -0.01946 4.02180

C -0.34764 -0.38741 2.84421

C -0.50617 0.35175 1.66192

C -1.32754 1.48521 1.71812

C -1.96730 1.86026 2.89506

C -3.95691 2.19645 10.18876

C -2.78737 2.97548 10.78444

H -8.93133 -10.56211 -1.57536

H -7.69040 -11.73688 -3.40455

H -5.91660 -10.60076 -4.62643

H -8.36241 -8.18829 -1.06493

H -2.56108 -7.27288 -4.88473

H -1.32821 -5.44468 -3.89498

H -4.68288 -4.54247 -1.35816

H -1.05692 -3.20500 -3.70694

H 0.19954 -1.37308 -2.67981

H -1.78370 -2.12314 1.07493

H -3.01891 -3.97111 0.04997

H -5.42274 -9.09494 -6.04447

H -4.18211 -7.86293 -6.14136

H -3.17241 -10.11071 -6.30228

H -2.60231 -9.34529 -4.80333

H -3.85107 -10.59514 -4.73321

H 14.24137 -0.20705 1.00398

H 14.50963 -2.69417 1.09839

H 12.60736 -4.12743 1.60371

H 12.00587 0.78314 1.49618

H 7.94273 -4.92251 1.68788

H 5.67279 -4.21030 1.25138

H 6.89625 -0.09752 1.55651

H 4.28807 -3.27262 -0.31977

H 1.99629 -2.55954 -0.80558

H 2.48968 0.80806 1.84203

H 4.77135 0.07763 2.34300

H 11.23932 -4.80393 3.07191

H 9.51304 -5.09884 3.10049

H 10.65936 -6.70123 1.59576

H 9.55987 -5.69083 0.63272

H 11.30035 -5.38053 0.59393

H -3.72864 8.98648 -9.62400

H -2.13516 10.79638 -8.95316

H -0.21390 10.29581 -7.54276

H -3.28376 6.64993 -8.87355

H 1.93175 7.61109 -4.29704

H 1.91957 5.72565 -2.78360

H -0.93444 3.71545 -5.29768

H 2.53219 3.58008 -2.39663

H 2.48242 1.69950 -0.83089

H -1.67733 1.04411 -1.76087

H -1.63290 2.94923 -3.29437

H 2.49220 8.35772 -6.21896

H 1.69894 9.35072 -7.42400

H 2.15862 10.67041 -5.38712

H 0.39772 10.54234 -5.58803

H 1.21983 9.55086 -4.37640

H -7.97998 6.70271 8.93309

H -6.71693 6.10531 11.00990

H -5.09831 4.28636 10.99594

H -7.59817 5.36426 6.86358

H -2.05789 1.48572 8.69261

H -0.94574 0.81176 6.65487

H -4.25840 2.29529 4.34572

H -0.85901 -0.63360 4.91504

H 0.28746 -1.27750 2.85065

H -1.46897 2.10160 0.82715

H -2.58159 2.76274 2.90378

H -4.79497 2.18108 10.89906

H -3.66947 1.14596 10.04541

H -2.48054 2.50496 11.73094

H -1.91498 2.98862 10.11735

H -3.04961 4.02050 10.99785

SCF Done: E(RCAM-B3LYP) = -5183.92442231   A.U. after   1 cycles

6.3.2 Computed excitation energies of compounds 5c/5d (cam-B3LYP/def2svp SMD CH_2Cl_2 GD3BJ)

Excitation energies and oscillator strengths:

Excited State 1: Singlet-A 3.6911 eV 335.90 nm f=0.3381 <S**2>=0.000

322 -> 327 -0.20811

322 -> 328 -0.11925

322 -> 329 -0.21946

323 -> 328 -0.14455

323 -> 329 -0.13763

324 -> 327 0.10927

324 -> 328 0.10185

324 -> 329 0.10695

325 -> 326 0.29952

325 -> 327 -0.22839

325 -> 328 0.18574

325 -> 330 0.14784

This state for optimization and/or second-order correction.

Total Energy, E(TD-HF/TD-DFT) = -5183.78877720

Copying the excited state density for this state as the 1-particle RhoCI density.

Excited State 2: Singlet-A 3.6938 eV 335.65 nm f=0.3441 <S**2>=0.000

322 -> 327 -0.22769

322 -> 328 0.15159

322 -> 329 0.10932

323 -> 326 0.30228

323 -> 327 0.25128

323 -> 329 -0.14451

323 -> 332 -0.13872

324 -> 327 0.10161

325 -> 328 -0.13234

325 -> 329 -0.22926

Excited State 3: Singlet-A 3.7037 eV 334.76 nm f=0.4242 <S**2>=0.000

322 -> 326 -0.10118

322 -> 328 -0.20534

322 -> 329 0.16784

323 -> 327 0.16273

323 -> 328 -0.15331

323 -> 329 -0.15285

324 -> 326 0.26659

324 -> 328 -0.20098

324 -> 329 0.19411

324 -> 331 -0.16786

325 -> 327 0.17751

325 -> 329 0.10247

Excited State 4: Singlet-A 3.7309 eV 332.31 nm f=0.0133 <S**2>=0.000

322 -> 326 -0.26580

322 -> 331 0.11665

323 -> 327 0.15677

323 -> 328 -0.10556

323 -> 329 -0.16268

324 -> 328 0.29856

324 -> 329 -0.22704

324 -> 331 0.16776

325 -> 327 0.18277

325 -> 329 0.12939

Excited State 5: Singlet-A 4.2723 eV 290.20 nm f=0.5073 <S**2>=0.000

322 -> 331 0.23400

324 -> 326 0.10172

324 -> 328 -0.12645

324 -> 331 0.49689

324 -> 343 0.10417

Excited State 6: Singlet-A 4.2884 eV 289.12 nm f=0.2765 <S**2>=0.000

322 -> 333 0.31231

323 -> 333 0.20905

324 -> 333 -0.18292

325 -> 326 -0.13585

325 -> 330 0.25096

325 -> 334 -0.29508

Excited State 7: Singlet-A 4.2928 eV 288.82 nm f=0.3406 <S**2>=0.000

322 -> 330 -0.15361

322 -> 332 -0.16658

322 -> 334 0.17600

323 -> 326 0.11707

323 -> 332 0.39553

323 -> 333 0.11126

323 -> 344 0.10534

325 -> 332 0.11760

325 -> 333 -0.25790

Excited State 8: Singlet-A 4.3103 eV 287.65 nm f=0.1239 <S**2>=0.000

322 -> 326 -0.11627

322 -> 331 -0.11965

322 -> 332 -0.20706

322 -> 334 -0.14241

323 -> 330 0.14257

323 -> 332 0.27926

323 -> 334 -0.14158

324 -> 332 0.11337

325 -> 333 0.29874

Excited State 9: Singlet-A 4.4654 eV 277.65 nm f=0.2835 <S**2>=0.000

322 -> 335 0.23700

322 -> 336 0.13180

322 -> 337 -0.10468

323 -> 338 0.10068

324 -> 334 -0.17661

324 -> 335 0.40230

324 -> 337 -0.24305

324 -> 338 0.14522

Excited State 10: Singlet-A 4.4702 eV 277.36 nm f=0.3060 <S**2>=0.000

322 -> 335 -0.11923

322 -> 337 -0.23210

322 -> 338 -0.16182

323 -> 335 -0.17635

323 -> 337 -0.22604

324 -> 337 0.17023

325 -> 336 0.31795

325 -> 337 0.20076

6.3.3 Computed emission energies of compounds 5c/5d (cam-B3LYP/def2svp SMD CH_2Cl_2 GD3BJ)

Excitation energies and oscillator strengths:

Excited State 1: Singlet-A 2.7564 eV 449.80 nm f=0.2046 <S**2>=0.000

325 -> 326 0.61575

325 -> 327 -0.13764

325 -> 330 -0.14713

325 -> 334 -0.10284

325 -> 338 0.12942

325 -> 339 -0.11998

This state for optimization and/or second-order correction.

Total Energy, E(TD-HF/TD-DFT) = -5183.75730649

Copying the excited state density for this state as the 1-particle RhoCI density.

Excited State 2: Singlet-A 3.7036 eV 334.77 nm f=0.2675 <S**2>=0.000

322 -> 327 0.13539

322 -> 328 0.10731

322 -> 329 -0.22810

323 -> 327 -0.17050

324 -> 327 0.25949

324 -> 328 -0.33040

324 -> 329 0.21260

324 -> 330 -0.11285

324 -> 333 -0.13642

324 -> 336 -0.14455

Excited State 3: Singlet-A 3.7109 eV 334.11 nm f=0.3354 <S**2>=0.000

322 -> 327 -0.25340

322 -> 329 0.26501

322 -> 332 0.13321

323 -> 327 0.22937

323 -> 328 0.14096

323 -> 329 -0.11471

323 -> 334 0.10068

324 -> 328 -0.12537

324 -> 329 0.16913

325 -> 330 -0.17596

325 -> 333 0.11320

325 -> 334 -0.13350

Excited State 4: Singlet-A 3.7205 eV 333.25 nm f=0.4386 <S**2>=0.000

322 -> 328 0.17714

322 -> 329 0.13373

322 -> 331 -0.10718

323 -> 327 0.14792

323 -> 328 0.26046

323 -> 329 0.18817

323 -> 331 -0.15940

325 -> 326 0.13659

325 -> 330 0.24705

325 -> 331 0.10723

325 -> 333 -0.15802

325 -> 334 0.18681

325 -> 345 -0.11610

Excited State 5: Singlet-A 3.7552 eV 330.17 nm f=0.0165 <S**2>=0.000

322 -> 327 -0.22647

323 -> 328 -0.14780

323 -> 329 -0.24849

323 -> 331 0.12832

324 -> 328 -0.20691

325 -> 326 0.13545

325 -> 330 0.23561

325 -> 331 0.10469

325 -> 333 -0.15013

325 -> 334 0.17425

325 -> 345 -0.12445

Excited State 6: Singlet-A 4.2163 eV 294.06 nm f=0.1499 <S**2>=0.000

325 -> 338 0.15790

325 -> 339 -0.19262

325 -> 340 0.10045

325 -> 344 0.58983

corrected external emission excitation: -5183.810535 - -5183.908447 = 0.097912 = 2.664322 eV 465.35 nm

6.4 Quantum chemical calculation data of tetrakis(4-(10-benzyl-10*H*-phenothiazin-3-yl)phenyl)silane (5e)

6.4.1 Computed xyz-coordinates of compound 5e (cam-B3LYP/def2svp SMD CH_2Cl_2 GD3BJ)

0 1

C -10.72549 -2.48920 -7.35670

C -10.99759 -3.76311 -6.87207

C -10.00757 -4.50541 -6.23332

C -8.70689 -4.00333 -6.08741

C -8.43791 -2.72352 -6.60532

C -9.44145 -1.97130 -7.21005

S -6.78082 -2.09997 -6.61808

N -7.70205 -4.75061 -5.44601

C -6.17929 -2.85890 -5.13614

C -6.65435 -4.12464 -4.75032

C -6.03749 -4.73221 -3.64856

C -4.99947 -4.10822 -2.96654

C -4.54419 -2.83892 -3.33291

C -5.16468 -2.22611 -4.42810

C -7.89590 -6.17813 -5.27612

C -3.44723 -2.16326 -2.59727

C -3.27937 -2.35255 -1.21843

C -2.25018 -1.72015 -0.53085

C -1.34773 -0.86730 -1.18463

C -1.51871 -0.68544 -2.56306

C -2.54502 -1.31866 -3.25672

Si -0.00051 0.02360 -0.21743

C -8.71008 -6.62430 -4.07487

C -8.92848 -7.99234 -3.88349

C -9.66720 -8.44908 -2.79673

C -10.20033 -7.54120 -1.88194

C -9.98763 -6.17807 -2.06569

C -9.24691 -5.72275 -3.15571

C -2.89084 9.09981 8.82227

C -4.13324 9.48389 8.33154

C -4.48843 9.21588 7.01205

C -3.59649 8.58008 6.13711

C -2.33366 8.21790 6.63957

C -1.99794 8.45620 7.96970

S -1.09585 7.57139 5.55100

N -3.94826 8.31188 4.80142

C -2.14056 6.70764 4.41221

C -3.41948 7.20840 4.11153

C -4.13776 6.56292 3.09616

C -3.60776 5.47157 2.41809

C -2.34921 4.95444 2.73626

C -1.63186 5.59416 3.75436

C -5.07020 9.02324 4.21918

C -1.79116 3.77793 2.02508

C -2.62685 2.75764 1.55304

C -2.10478 1.65757 0.88044

C -0.72879 1.52540 0.65326

C 0.10323 2.55144 1.12665

C -0.41321 3.65310 1.79880

C -6.45315 8.44936 4.46952

C -7.55861 9.09236 3.90356

C -8.84522 8.60255 4.10388

C -9.04593 7.45810 4.87542

C -7.95072 6.81251 5.44194

C -6.66217 7.30541 5.23989

C 4.72230 -10.74688 5.61520

C 5.54326 -10.12669 6.54970

C 5.34593 -8.79125 6.89160

C 4.30104 -8.04662 6.32716

C 3.46210 -8.69302 5.40157

C 3.68678 -10.01777 5.03670

S 2.02071 -7.87078 4.78373

N 4.09349 -6.69857 6.67314

C 2.62720 -6.20707 4.77585

C 3.53580 -5.78622 5.76315

C 3.85528 -4.42245 5.80096

C 3.29611 -3.52412 4.89965

C 2.41588 -3.94581 3.89960

C 2.10234 -5.30976 3.85416

C 4.69866 -6.18862 7.88889

C 1.83343 -2.99421 2.92200

C 1.55403 -1.66925 3.28020

C 1.00658 -0.77897 2.36192

C 0.71246 -1.17149 1.04980

C 0.98884 -2.50176 0.69852

C 1.54046 -3.39379 1.61047

C 6.13873 -5.71702 7.79662

C 6.86800 -5.76057 6.60834

C 8.18862 -5.31478 6.57026

C 8.79571 -4.81993 7.72078

C 8.07343 -4.77375 8.91308

C 6.75596 -5.21907 8.94858

C 10.82825 0.96241 -7.62608

C 10.86314 2.34616 -7.75132

C 9.72491 3.10957 -7.50456

C 8.50998 2.50525 -7.15276

C 8.48191 1.10245 -7.05340

C 9.63218 0.34730 -7.26605

S 6.94771 0.25978 -6.78719

N 7.35558 3.27193 -6.90841

C 6.12765 1.47251 -5.79126

C 6.36392 2.84102 -6.01261

C 5.56703 3.75158 -5.30597

C 4.58345 3.31858 -4.42438

C 4.36839 1.95947 -4.18112

C 5.17027 1.04770 -4.87840

C 7.31424 4.64127 -7.38495

C 3.33361 1.49505 -3.22514

C 2.13442 2.20215 -3.06113

C 1.16767 1.76955 -2.16104

C 1.34987 0.61126 -1.39002

C 2.55134 -0.08949 -1.55727

C 3.52368 0.34080 -2.45454

C 7.95038 5.69728 -6.49897

C 7.94001 7.02805 -6.92883

C 8.51036 8.03066 -6.15117

C 9.10160 7.71606 -4.92773

C 9.11602 6.39391 -4.49314

C 8.54375 5.39074 -5.27438

H -11.50206 -1.89913 -7.84675

H -11.99737 -4.19034 -6.97443

H -10.26185 -5.48614 -5.83650

H -9.20177 -0.97462 -7.58751

H -6.36742 -5.71119 -3.30697

H -4.53472 -4.63412 -2.13107

H -4.85267 -1.22907 -4.74457

H -8.36671 -6.56539 -6.19133

H -6.90345 -6.64955 -5.23122

H -3.97475 -2.99188 -0.67115

H -2.15419 -1.89617 0.54404

H -0.83103 -0.04304 -3.11830

H -2.63125 -1.16911 -4.33476

H -8.51261 -8.70830 -4.59798

H -9.82863 -9.52114 -2.66186

H -10.78055 -7.89771 -1.02795

H -10.40069 -5.45794 -1.35567

H -9.08689 -4.65180 -3.29059

H -2.61328 9.29723 9.85927

H -4.85133 9.98813 8.98165

H -5.48095 9.50088 6.66873

H -1.01374 8.14896 8.33072

H -5.13225 6.90909 2.82249

H -4.19617 5.01992 1.61786

H -0.64838 5.22185 4.04695

H -5.03940 10.05920 4.58660

H -4.90125 9.08810 3.13450

H -3.70302 2.81491 1.72747

H -2.79258 0.88492 0.52874

H 1.18237 2.50079 0.95788

H 0.26472 4.44108 2.13267

H -7.40634 9.98955 3.29697

H -9.69797 9.11645 3.65415

H -10.05523 7.07145 5.03302

H -8.09611 5.91505 6.04755

H -5.81039 6.79092 5.68781

H 4.88117 -11.78994 5.33558

H 6.36270 -10.67759 7.01613

H 6.02581 -8.32619 7.60267

H 3.02491 -10.48482 4.30384

H 4.56614 -4.04712 6.53421

H 3.58730 -2.47446 4.96348

H 1.39803 -5.68210 3.10798

H 4.07400 -5.36022 8.25372

H 4.62747 -6.97391 8.65550

H 1.74538 -1.33244 4.30081

H 0.79414 0.24252 2.68627

H 0.77160 -2.85594 -0.31295

H 1.76152 -4.41451 1.29245

H 6.40161 -6.14646 5.70040

H 8.74488 -5.35567 5.63100

H 9.83005 -4.47032 7.69070

H 8.54068 -4.38734 9.82186

H 6.19382 -5.18077 9.88590

H 11.72119 0.36136 -7.80707

H 11.78972 2.85184 -8.03115

H 9.79432 4.19278 -7.58070

H 9.57802 -0.73919 -7.16648

H 5.71981 4.82197 -5.42781

H 3.99685 4.06762 -3.88978

H 5.02066 -0.02502 -4.74206

H 7.79247 4.66859 -8.37468

H 6.26091 4.90559 -7.55683

H 1.94661 3.09602 -3.65901

H 0.24767 2.35219 -2.06270

H 2.74599 -0.98865 -0.96787

H 4.45671 -0.22000 -2.53880

H 7.47770 7.27962 -7.88751

H 8.49382 9.06525 -6.50166

H 9.55007 8.50175 -4.31567

H 9.57662 6.13601 -3.53676

H 8.56117 4.35725 -4.92458

SCF Done: E(RCAM-B3LYP) = -5949.96706618 A.U. after 1 cycles

Zero-point correction= 1.456097 (Hartree/Particle)

Thermal correction to Energy= 1.542852

Thermal correction to Enthalpy= 1.543797

Thermal correction to Gibbs Free Energy= 1.310659

Sum of electronic and zero-point Energies= -5948.510969

Sum of electronic and thermal Energies= -5948.424214

Sum of electronic and thermal Enthalpies= -5948.423270

Sum of electronic and thermal Free Energies= -5948.656407

6.4.2 Computed excitation energies of compound 5e (cam-B3LYP/def2svp SMD CH_2Cl_2 GD3BJ)

Excitation energies and oscillator strengths:

Excited State 1: Singlet-A 3.7901 eV 327.13 nm f=0.3137 <S**2>=0.000

386 -> 392 -0.24280

386 -> 393 0.21798

387 -> 390 0.30814

387 -> 391 -0.27485

387 -> 396 -0.13332

388 -> 392 -0.20139

388 -> 393 0.16986

This state for optimization and/or second-order correction.

Total Energy, E(TD-HF/TD-DFT) = -5949.82778385

Copying the excited state density for this state as the 1-particle RhoCI density.

Excited State 2: Singlet-A 3.7967 eV 326.56 nm f=0.4116 <S**2>=0.000

386 -> 390 -0.12481

386 -> 391 0.27877

386 -> 396 0.10617

387 -> 392 0.28187

387 -> 393 -0.20344

388 -> 390 -0.27484

389 -> 393 -0.19204

Excited State 3: Singlet-A 3.8057 eV 325.79 nm f=0.3579 <S**2>=0.000

386 -> 392 -0.17459

386 -> 393 -0.18333

388 -> 391 -0.11449

388 -> 392 0.18356

388 -> 393 0.24293

389 -> 390 0.27341

389 -> 391 0.28764

389 -> 394 0.12423

389 -> 395 -0.12559

389 -> 404 0.11587

Excited State 4: Singlet-A 3.8328 eV 323.49 nm f=0.0201 <S**2>=0.000

386 -> 390 -0.25887

387 -> 392 0.12827

387 -> 393 -0.17522

388 -> 391 0.28673

388 -> 395 -0.12074

389 -> 392 0.24619

389 -> 393 0.24736

389 -> 403 -0.10568

Excited State 5: Singlet-A 4.3077 eV 287.82 nm f=0.4987 <S**2>=0.000

386 -> 394 0.16154

386 -> 397 -0.14750

388 -> 394 -0.17510

388 -> 395 -0.14627

388 -> 397 0.14268

389 -> 390 0.12560

389 -> 391 0.11425

389 -> 394 -0.21382

389 -> 395 0.29432

389 -> 397 0.13414

389 -> 398 -0.12181

Excited State 6: Singlet-A 4.3175 eV 287.16 nm f=0.4355 <S**2>=0.000

386 -> 395 -0.20157

386 -> 396 0.13780

387 -> 395 0.12780

388 -> 390 0.11121

388 -> 395 0.31127

388 -> 416 0.10318

389 -> 394 -0.19959

389 -> 395 -0.15713

389 -> 397 0.19973

Excited State 7: Singlet-A 4.3350 eV 286.01 nm f=0.3475 <S**2>=0.000

386 -> 394 0.12900

386 -> 396 0.11755

386 -> 397 0.24586

387 -> 390 0.12346

387 -> 394 -0.10074

387 -> 396 0.35636

387 -> 398 -0.11161

387 -> 399 -0.10636

388 -> 394 0.11585

388 -> 397 0.21089

Excited State 8: Singlet-A 4.3517 eV 284.91 nm f=0.0991 <S**2>=0.000

386 -> 390 0.11708

386 -> 396 0.26642

387 -> 394 0.14579

387 -> 397 0.29688

388 -> 395 -0.11444

388 -> 396 0.20467

389 -> 394 0.10459

389 -> 396 0.10037

Excited State 9: Singlet-A 4.5247 eV 274.02 nm f=0.1975 <S**2>=0.000

386 -> 400 -0.15491

386 -> 401 0.11803

386 -> 402 0.14853

388 -> 398 -0.11521

388 -> 401 -0.21796

388 -> 402 -0.23889

389 -> 399 -0.13482

389 -> 400 0.21696

389 -> 401 0.29449

Excited State 10: Singlet-A 4.5263 eV 273.92 nm f=0.4232 <S**2>=0.000

386 -> 399 0.20883

386 -> 400 -0.14468

387 -> 399 -0.18738

387 -> 400 0.11390

387 -> 401 -0.10813

387 -> 402 0.16046

388 -> 401 0.28101

389 -> 398 -0.10131

389 -> 400 0.23352

389 -> 402 -0.19893

6.4.3 Computed emission energies of compound 5e (cam-B3LYP/def2svp SMD CH_2Cl_2 GD3BJ)

Excitation energies and oscillator strengths:

Excited State 1: Singlet-A 2.8075 eV 441.62 nm f=0.1978 <S**2>=0.000

389 -> 390 0.61961

389 -> 391 -0.12396

389 -> 394 -0.15002

389 -> 411 -0.15107

This state for optimization and/or second-order correction.

Total Energy, E(TD-HF/TD-DFT) = -5949.78461304

Copying the excited state density for this state as the 1-particle RhoCI density.

Excited State 2: Singlet-A 3.7513 eV 330.51 nm f=0.4212 <S**2>=0.000

389 -> 390 0.20385

389 -> 394 0.40665

389 -> 396 0.28938

389 -> 398 -0.28574

389 -> 401 0.11164

389 -> 417 -0.18226

Excited State 3: Singlet-A 3.8103 eV 325.40 nm f=0.3056 <S**2>=0.000

386 -> 392 -0.25412

386 -> 393 -0.15920

387 -> 391 0.22074

387 -> 392 0.30300

387 -> 397 -0.11711

387 -> 405 -0.11081

388 -> 391 0.18022

388 -> 392 0.12025

388 -> 393 0.24789

388 -> 397 -0.11685

Excited State 4: Singlet-A 3.8179 eV 324.74 nm f=0.3067 <S**2>=0.000

386 -> 392 -0.13577

386 -> 393 0.31733

387 -> 391 -0.26096

387 -> 393 0.12446

387 -> 395 0.13078

387 -> 403 0.10347

388 -> 391 0.22720

388 -> 392 -0.22797

388 -> 393 0.12183

388 -> 394 -0.10417

388 -> 403 -0.12127

Excited State 5: Singlet-A 3.8481 eV 322.19 nm f=0.0400 <S**2>=0.000

386 -> 391 -0.31627

386 -> 395 0.15488

386 -> 403 0.12284

387 -> 393 0.30515

388 -> 392 0.28288

388 -> 393 -0.13456

388 -> 396 -0.11946

Excited State 6: Singlet-A 4.2546 eV 291.41 nm f=0.1173 <S**2>=0.000

389 -> 402 0.23762

389 -> 404 -0.14230

389 -> 409 -0.11413

389 -> 410 -0.10157

389 -> 411 0.20891

389 -> 416 -0.47182

389 -> 417 0.14297

corrected external emission excitation: -5949.850165 - -5949.950146 = 0.099981 = 2.720634 eV 455.72 nm

6.5 Quantum chemical calculation data of tetrakis(4-(7-(4-fluorophenyl)-10-(4-methoxyphenyl)-10*H*-phenothiazin-3-yl)phenyl)silane (5f)

6.5.1 Computed xyz-coordinates of compound 5f (cam-B3LYP/def2svp SMD CH_2Cl_2 GD3BJ)

0 1

C -9.61877 3.80239 -7.65612

C -8.84674 4.35512 -8.68174

C -7.51069 4.68546 -8.48848

C -6.88817 4.49888 -7.24828

C -7.67189 3.97611 -6.20509

C -8.99750 3.61526 -6.41578

S -7.02434 3.90766 -4.55816

N -5.53267 4.82192 -7.04523

C -5.32472 3.58822 -4.94024

C -4.74654 4.14291 -6.09525

C -3.36351 3.99458 -6.25724

C -2.59587 3.31940 -5.31604

C -3.17299 2.73859 -4.18308

C -4.55617 2.88275 -4.02231

C -4.87005 5.62746 -8.02672

C -11.04011 3.43021 -7.86543

C -11.59939 2.32180 -7.21403

C -12.93038 1.96679 -7.40491

C -13.70830 2.73076 -8.26084

C -13.19423 3.83327 -8.92555

C -11.86207 4.17652 -8.72163

C -2.35994 2.00355 -3.18354

C -1.03806 2.38086 -2.90799

C -0.28213 1.69586 -1.96389

C -0.80777 0.60445 -1.25534

C -2.12927 0.23335 -1.53485

C -2.89015 0.91518 -2.47910

Si 0.24712 -0.32072 -0.00047

C -4.34858 5.06837 -9.19863

C -3.71627 5.86931 -10.13433

C -3.59102 7.24958 -9.91608

C -4.10650 7.81129 -8.74457

C -4.74243 6.99410 -7.81111

F -14.99211 2.39424 -8.45102

C 11.11555 -6.90783 -3.35164

C 10.77983 -7.17719 -4.68125

C 9.46619 -7.10223 -5.12851

C 8.41898 -6.77578 -4.25793

C 8.74979 -6.53658 -2.91285

C 10.07024 -6.57859 -2.48099

S 7.47092 -6.32517 -1.70646

N 7.08654 -6.69144 -4.70530

C 6.25617 -5.52594 -2.71681

C 6.14814 -5.84753 -4.08084

C 5.07059 -5.29842 -4.78710

C 4.15251 -4.46040 -4.16614

C 4.27560 -4.11305 -2.81788

C 5.35290 -4.66029 -2.11111

C 6.77035 -7.22008 -5.99846

C 12.51928 -6.97030 -2.87441

C 12.97934 -6.10652 -1.87049

C 14.29337 -6.15871 -1.41820

C 15.15612 -7.08624 -1.98110

C 14.74192 -7.95805 -2.97609

C 13.42392 -7.89478 -3.41526

C 3.30365 -3.21001 -2.15397

C 2.67932 -2.17376 -2.86006

C 1.76742 -1.32890 -2.23526

C 1.44099 -1.47913 -0.88132

C 2.06510 -2.52268 -0.18076

C 2.97732 -3.36894 -0.79983

C 6.98059 -6.47262 -7.16252

C 6.66868 -7.00565 -8.40150

C 6.13865 -8.30086 -8.50212

C 5.92707 -9.05077 -7.34169

C 6.24514 -8.50223 -6.10001

F 16.42502 -7.14184 -1.55168

C 6.12398 10.61804 4.34885

C 6.99873 10.01504 5.25690

C 6.78203 8.72561 5.72789

C 5.66291 7.98417 5.32923

C 4.76407 8.60065 4.44160

C 5.00634 9.87703 3.94760

S 3.22426 7.81846 4.05054

N 5.44042 6.67451 5.79630

C 3.74142 6.12635 4.11890

C 4.73390 5.72714 5.03053

C 4.98633 4.35440 5.14541

C 4.29307 3.42655 4.37776

C 3.33046 3.82306 3.44497

C 3.07583 5.19473 3.33063

C 6.22144 6.20760 6.90250

C 6.36110 11.98786 3.82945

C 5.99240 12.33769 2.52277

C 6.21049 13.61912 2.02845

C 6.80742 14.55963 2.85343

C 7.18786 14.25493 4.15110

C 6.96018 12.96971 4.63110

C 2.60073 2.83531 2.61249

C 3.22398 1.65959 2.17469

C 2.53939 0.73386 1.39388

C 1.20649 0.94056 1.01558

C 0.58961 2.12320 1.45134

C 1.26786 3.04972 2.23469

C 7.51086 5.69613 6.71875

C 8.24804 5.25239 7.80316

C 7.70942 5.31142 9.09742

C 6.42174 5.82169 9.28533

C 5.68952 6.26480 8.18460

F 7.02231 15.79698 2.38387

C -9.86726 -4.40961 7.33385

C -9.67963 -5.78439 7.16625

C -8.46329 -6.30546 6.74186

C -7.36485 -5.47503 6.48674

C -7.54040 -4.09524 6.68799

C -8.77079 -3.57827 7.07764

S -6.15871 -2.99416 6.57612

N -6.13194 -5.99612 6.04811

C -5.21830 -3.83697 5.33523

C -5.24853 -5.23990 5.25417

C -4.36488 -5.85042 4.35520

C -3.50377 -5.09809 3.56581

C -3.49556 -3.70138 3.61989

C -4.37705 -3.08985 4.51898

C -5.92233 -7.41014 6.13818

C -11.16986 -3.84978 7.77306

C -11.22967 -2.70808 8.58460

C -12.44687 -2.17911 9.00019

C -13.61747 -2.80385 8.59928

C -13.60295 -3.93478 7.79783

C -12.37755 -4.44949 7.38871

C -2.58909 -2.89464 2.76645

C -2.23670 -3.32556 1.47976

C -1.38626 -2.56988 0.68146

C -0.85583 -1.34916 1.12610

C -1.20894 -0.92592 2.41384

C -2.05628 -1.68080 3.21876

C -5.22529 -7.92820 7.23179

C -5.00949 -9.29215 7.34406

C -5.48941 -10.16673 6.35962

C -6.18784 -9.65259 5.26153

C -6.39788 -8.27912 5.16050

F -14.79496 -2.30086 8.99683

O -2.96556 7.95033 -10.88053

C -2.81425 9.34365 -10.72861

O 5.86549 -8.73680 -9.74619

C 5.32855 -10.02867 -9.91880

O 8.49765 4.85845 10.09026

C 8.02119 4.89347 11.41635

O -5.23569 -11.47527 6.54883

C -5.69464 -12.40888 5.59797

H -9.28300 4.50770 -9.67039

H -6.94359 5.09533 -9.32192

H -9.56710 3.21549 -5.57460

H -2.87418 4.41406 -7.13421

H -1.52330 3.22166 -5.49188

H -5.04824 2.47172 -3.13884

H -10.97932 1.70862 -6.55772

H -13.36809 1.10077 -6.90542

H -13.83992 4.41537 -9.58539

H -11.46045 5.05632 -9.22733

H -0.60120 3.23949 -3.42146

H 0.73903 2.03370 -1.76716

H -2.58296 -0.60903 -1.00739

H -3.91044 0.58364 -2.68147

H -4.44410 3.99469 -9.37290

H -3.30606 5.44826 -11.05382

H -4.02042 8.87903 -8.54810

H -5.14923 7.42642 -6.89486

H 11.56186 -7.42388 -5.40137

H 9.25515 -7.30192 -6.17724

H 10.27918 -6.38744 -1.42667

H 4.93470 -5.54002 -5.83943

H 3.31473 -4.08023 -4.75273

H 5.49719 -4.41090 -1.05829

H 12.30378 -5.36304 -1.44393

H 14.65801 -5.48349 -0.64217

H 15.44797 -8.68049 -3.38923

H 13.08943 -8.59420 -4.18328

H 2.92131 -2.01254 -3.91231

H 1.30482 -0.53232 -2.82289

H 1.82801 -2.69141 0.87328

H 3.42643 -4.18168 -0.22583

H 7.39296 -5.46428 -7.08864

H 6.82698 -6.43533 -9.31843

H 5.51704 -10.05851 -7.39088

H 6.08217 -9.08191 -5.18934

H 7.89256 10.54642 5.58825

H 7.50107 8.28997 6.41888

H 4.27924 10.31599 3.26172

H 5.72860 3.99896 5.85736

H 4.50682 2.36641 4.52350

H 2.33922 5.55478 2.61013

H 5.54150 11.59000 1.86790

H 5.93156 13.89371 1.00968

H 7.64748 15.02440 4.77385

H 7.23869 12.73420 5.65963

H 4.26937 1.47382 2.42887

H 3.06678 -0.16474 1.06489

H -0.44842 2.33091 1.17751

H 0.74586 3.94737 2.57165

H 7.93382 5.64901 5.71321

H 9.25499 4.85103 7.67648

H 5.97922 5.87917 10.27888

H 4.68307 6.66437 8.32359

H -10.49577 -6.47644 7.38030

H -8.36513 -7.38259 6.62260

H -8.86944 -2.49641 7.18491

H -4.33703 -6.93550 4.27852

H -2.81543 -5.62181 2.90030

H -4.41367 -2.00130 4.59212

H -10.30700 -2.22920 8.91672

H -12.49742 -1.29478 9.63776

H -14.54548 -4.39445 7.49544

H -12.36495 -5.32751 6.74066

H -2.64751 -4.25873 1.08975

H -1.13533 -2.94352 -0.31512

H -0.80799 0.01013 2.80986

H -2.28931 -1.32850 4.22546

H -4.85284 -7.24439 7.99695

H -4.46678 -9.71092 8.19328

H -6.57111 -10.30993 4.48221

H -6.94297 -7.87815 4.30347

H -2.28918 9.70142 -11.62321

H -3.78931 9.85408 -10.65860

H -2.21345 9.59355 -9.83827

H 5.18761 -10.16813 -10.99805

H 6.01329 -10.80781 -9.54434

H 4.35364 -10.13514 -9.41440

H 8.81880 4.48115 12.04713

H 7.80357 5.92385 11.74358

H 7.11434 4.27839 11.53999

H -5.37900 -13.39830 5.95227

H -6.79391 -12.39485 5.51105

H -5.25331 -12.22855 4.60354

SCF Done: E(RCAM-B3LYP) = -7570.17334334 A.U. after 1 cycles

Zero-point correction= 1.763571 (Hartree/Particle)

Thermal correction to Energy= 1.878692

Thermal correction to Enthalpy= 1.879636

Thermal correction to Gibbs Free Energy= 1.578034

Sum of electronic and zero-point Energies= -7568.409773

Sum of electronic and thermal Energies= -7568.294651

Sum of electronic and thermal Enthalpies= -7568.293707

Sum of electronic and thermal Free Energies= -7568.595309

6.5.2 Computed excitation energies of compound 5f (cam-B3LYP/def2svp SMD CH_2Cl_2 GD3BJ)

Excitation energies and oscillator strengths:

Excited State 1: Singlet-A 3.6687 eV 337.95 nm f=0.7013 <S**2>=0.000

498 -> 503 0.18559

498 -> 504 0.17064

498 -> 505 0.20692

499 -> 502 0.25310

499 -> 503 -0.14346

499 -> 504 0.24976

499 -> 505 0.11602

500 -> 502 0.20683

500 -> 504 0.24528

500 -> 505 0.10035

This state for optimization and/or second-order correction.

Total Energy, E(TD-HF/TD-DFT) = -7570.03852253

Copying the excited state density for this state as the 1-particle RhoCI density.

Excited State 2: Singlet-A 3.6698 eV 337.85 nm f=0.3200 <S**2>=0.000

498 -> 502 -0.14823

498 -> 503 0.23530

498 -> 505 0.16276

499 -> 503 -0.10845

499 -> 504 -0.14378

499 -> 505 -0.10518

501 -> 502 0.25209

501 -> 504 -0.30496

501 -> 505 0.27042

501 -> 506 -0.11734

Excited State 3: Singlet-A 3.6750 eV 337.37 nm f=0.5636 <S**2>=0.000

498 -> 502 0.10178

498 -> 503 -0.27053
499 -> 502 -0.14745
499 -> 505 0.25115
500 -> 502 0.21038
500 -> 503 0.26537
500 -> 505 -0.19533
501 -> 504 -0.16709
501 -> 505 0.12876

Excited State 4: Singlet-A 3.7023 eV 334.88 nm f=0.0566 <S**2>=0.000
498 -> 502 0.25689
498 -> 505 -0.10354
498 -> 506 -0.11330
499 -> 502 0.12076
499 -> 503 0.24590
499 -> 504 0.13179
500 -> 503 -0.19978
500 -> 504 0.12934
500 -> 505 0.23644
501 -> 504 -0.21224
501 -> 505 0.19179

Excited State 5: Singlet-A 4.2485 eV 291.83 nm f=0.6607 <S**2>=0.000
498 -> 510 0.10696
501 -> 502 0.10413
501 -> 504 -0.10060
501 -> 506 0.13214
501 -> 507 -0.13210
501 -> 510 0.48385
501 -> 526 -0.17030

Excited State 6: Singlet-A 4.2688 eV 290.45 nm f=0.5434 <S**2>=0.000

498 -> 511 0.12264

499 -> 508 0.10219

499 -> 509 0.11129

499 -> 511 0.25887

499 -> 512 0.19268

500 -> 507 0.15002

500 -> 511 0.30292

500 -> 512 -0.12919

500 -> 525 0.10576

Excited State 7: Singlet-A 4.2840 eV 289.41 nm f=0.5025 <S**2>=0.000

498 -> 511 0.10413

498 -> 512 -0.12723

498 -> 513 -0.15534

499 -> 511 0.17539

499 -> 512 -0.17893

499 -> 513 0.12433

500 -> 508 0.11684

500 -> 509 0.10460

500 -> 512 0.33402

Excited State 8: Singlet-A 4.3108 eV 287.61 nm f=0.2426 <S**2>=0.000

498 -> 502 0.10893

498 -> 513 0.39282

498 -> 524 0.11458

499 -> 511 0.12401

499 -> 513 -0.19435

500 -> 512 0.14073

501 -> 510 0.11167

Excited State   9:   Singlet-A   4.3451 eV 285.34 nm f=0.0159 <S**2>=0.000

501 -> 506   0.19044

501 -> 507   -0.22185

501 -> 509   0.19827

501 -> 510   -0.21765

501 -> 515   0.25070

501 -> 516   -0.18019

501 -> 519   0.10044

501 -> 520   -0.24362

501 -> 522   -0.17656

Excited State 10:   Singlet-A   4.3571 eV 284.55 nm f=0.0317 <S**2>=0.000

499 -> 507   -0.15644

499 -> 509   -0.12170

499 -> 511   0.17821

499 -> 514   0.13709

499 -> 516   0.14358

499 -> 521   -0.15360

499 -> 522   -0.10154

500 -> 507   -0.16126

500 -> 511   0.17303

500 -> 514   0.16251

500 -> 516   0.12191

500 -> 521   -0.10846

500 -> 522   -0.12764

6.5.3 Computed emission energies of compound 5f (cam-B3LYP/def2svp SMD CH_2Cl_2 GD3BJ)

Excitation energies and oscillator strengths:

Excited State 1: Singlet-A 2.7566 eV 449.77 nm f=0.2068 <S**2>=0.000

325 -> 326 0.61551

325 -> 327 0.13613

325 -> 330 0.14545

325 -> 334 0.10301

325 -> 338 -0.14472

325 -> 339 0.11583

This state for optimization and/or second-order correction.

Total Energy, E(TD-HF/TD-DFT) = -5183.75735975

Copying the excited state density for this state as the 1-particle RhoCI density.

Excited State 2: Singlet-A 3.7055 eV 334.59 nm f=0.2656 <S**2>=0.000

322 -> 327 0.21357

322 -> 329 0.34449

322 -> 332 0.14204

323 -> 327 -0.32488

323 -> 329 -0.14132

323 -> 332 -0.12264

324 -> 327 0.10707

324 -> 328 -0.20493

Excited State 3: Singlet-A 3.7122 eV 333.99 nm f=0.3403 <S**2>=0.000

322 -> 327 0.13638

322 -> 328 -0.19530

323 -> 328 -0.21654

323 -> 329 -0.10752

324 -> 327 -0.22176

324 -> 328 0.18742

324 -> 329 0.26387

324 -> 330 0.10511

324 -> 333 -0.11799

324 -> 336 0.11598

325 -> 330 0.16742

325 -> 333 0.10443

325 -> 334 0.12694

Excited State 4: Singlet-A 3.7183 eV 333.45 nm f=0.4370 <S**2>=0.000

322 -> 327 0.12096

322 -> 328 0.17869

322 -> 331 0.10639

323 -> 327 0.10177

323 -> 328 0.21733

323 -> 329 -0.16098

323 -> 331 0.12777

324 -> 327 0.11425

324 -> 328 0.15213

324 -> 329 -0.13163

324 -> 331 0.10048

325 -> 326 -0.13945

325 -> 330 0.25025

325 -> 331 -0.12359

325 -> 333 0.15463

325 -> 334 0.19017

325 -> 345 0.11765

Excited State 5: Singlet-A 3.7550 eV 330.18 nm f=0.0185 <S**2>=0.000

322 -> 327 -0.22805

323 -> 329 0.24549

324 -> 328 -0.25706

325 -> 326 -0.13815

325 -> 330 0.23783

325 -> 331 -0.11997

325 -> 333 0.14645

325 -> 334 0.17658

325 -> 345 0.12654

Excited State 6: Singlet-A 4.2176 eV 293.97 nm f=0.1557 <S**2>=0.000

325 -> 338 0.17523

325 -> 339 -0.19353

325 -> 344 -0.59004

6.6 Quantum chemical calculation data of tetrakis(4-(10-hexyl-7-(4-(trifluoromethyl)phenyl)-10*H*-phenothiazin-3-yl)phenyl)silane (5g)

6.6.1 Computed xyz-coordinates of compound 5g (cam-B3LYP/def2svp SMD CH_2Cl_2 GD3BJ)

0 1

C -7.87488 5.20009 -9.13103

C -9.07873 4.62465 -8.74517

C -9.23803 4.10028 -7.46477

C -8.20464 4.15670 -6.51703

C -7.00284 4.77589 -6.91301

C -6.83674 5.26469 -8.20598

S -5.72405 5.08371 -5.73015

N -8.36370 3.62685 -5.22253

C -5.97323 3.68455 -4.67718

C -7.26706 3.16660 -4.47940

C -7.40475 2.17838 -3.49647

C -6.31674 1.70877 -2.76457

C -5.02075 2.19163 -2.99285

C -4.88820 3.18642 -3.96834

C -9.70782 3.27282 -4.77180

C -10.22282 1.89959 -5.19384

C -3.83379 1.69873 -2.25159

C -3.64892 0.33333 -1.99032

C -2.52214 -0.11153 -1.31017

C -1.53844 0.78031 -0.85437

C -1.72697 2.14101 -1.12529

C -2.85103 2.59314 -1.81048

Si -0.06130 0.15217 0.12884

C -4.63257 -5.81586 10.86305

C -3.95344 -5.09362 11.83595

C -3.38858 -3.85586 11.53786

C -3.50603 -3.28652 10.26072

C -4.22889 -4.01551 9.29606

C -4.75900 -5.26900 9.58920

S -4.61249 -3.28970 7.72908

N -2.93586 -2.03606 9.95741

C -3.17509 -2.27858 7.52847

C -2.54866 -1.70539 8.65091

C -1.52882 -0.77989 8.39768

C -1.13390 -0.45251 7.10271

C -1.72842 -1.05415 5.98472

C -2.75213 -1.97372 6.24172

C -2.47112 -1.19120 11.05517

C -1.07213 -1.48416 11.59049

C -1.32350 -0.74842 4.59020

C 0.02486 -0.60928 4.23025

C 0.38340 -0.33623 2.91585

C -0.58032 -0.19186 1.90509

C -1.92420 -0.33124 2.27301

C -2.29028 -0.60456 3.58758

C 9.95361 7.97769 3.42465

C 10.30023 8.44326 2.16270

C 9.41222 8.33266 1.09539

C 8.13588 7.77343 1.26080

C 7.78642 7.34001 2.55486

C 8.69193 7.41929 3.60938

S 6.13212 6.82220 2.90810

N 7.23795 7.65915 0.18312

C 5.71590 6.12110 1.33840

C 6.24975 6.66478 0.15500

C 5.73661 6.17175 -1.05085

C 4.76359 5.17590 -1.08680

C 4.26262 4.60286 0.09035

C 4.77077 5.10485 1.29421

C 7.52944 8.38497 -1.05077

C 8.49141 7.70851 -2.02369

C 3.24226 3.52546 0.09284

C 3.30556 2.45266 -0.80648

C 2.34911 1.44373 -0.77793

C 1.28961 1.46284 0.13964

C 1.23757 2.53543 1.04177

C 2.19190 3.54678 1.01993

C 7.48914 -8.13662 -6.60289

C 7.04058 -9.34481 -6.08494

C 5.82083 -9.42291 -5.41709

C 4.99573 -8.29812 -5.26589

C 5.44820 -7.08899 -5.82973

C 6.68508 -7.00886 -6.46371

S 4.38866 -5.67349 -5.87963

N 3.76392 -8.37592 -4.58911

C 3.48379 -5.94748 -4.38479

C 3.20537 -7.25793 -3.95288

C 2.33474 -7.38828 -2.86378

C 1.78666 -6.28152 -2.21951

C 2.09936 -4.97509 -2.62007

C 2.96190 -4.84889 -3.71545

C 3.21899 -9.69576 -4.27949

C 3.74891 -10.36382 -3.01375

C 1.56007 -3.76845 -1.94540

C 1.14703 -2.66179 -2.69962

C 0.65807 -1.51730 -2.07989

C 0.56610 -1.42547 -0.68313

C 0.97783 -2.53734 0.06383

C 1.46400 -3.68695 -0.54988

O -6.56009 0.73713 -1.83478

C -6.63448 1.18353 -0.49341

O -0.12493 0.45936 6.96793

C -0.53470 1.77671 6.65079

O 4.33713 4.76132 -2.31714

C 3.10531 5.30848 -2.74955

O 0.95336 -6.52187 -1.16332

C -0.43027 -6.43293 -1.44924

H -7.74049 5.60019 -10.13759

H -9.91270 4.56532 -9.44778

H -10.19018 3.64067 -7.21278

H -5.88316 5.72281 -8.47848

H -8.36706 1.72962 -3.26620

H -3.89683 3.59122 -4.18145

H -9.71677 3.35317 -3.67616

H -10.39018 4.05439 -5.13270

H -10.26681 1.79018 -6.28578

H -9.59515 1.08447 -4.80941

H -11.24017 1.75547 -4.79936

H -4.39830 -0.38322 -2.32629

H -2.40703 -1.18494 -1.13566

H -0.98561 2.87168 -0.79292

H -2.97528 3.66291 -1.99227

H -5.06539 -6.79229 11.08792

H -3.84338 -5.49511 12.84558

H -2.84722 -3.33528 12.32352

H -5.29816 -5.80951 8.80776

H -0.99747 -0.28167 9.20383

H -3.24219 -2.46994 5.40167

H -2.52526 -0.14957 10.70969

H -3.20702 -1.27570 11.86652

H -0.98604 -2.50158 11.99507

H -0.30088 -1.37457 10.81619

H -0.83789 -0.77805 12.40160

H 0.79687 -0.71142 4.99272

H 1.44509 -0.23015 2.67631

H -2.71106 -0.21449 1.52405

H -3.34888 -0.68798 3.84259

H 10.65100 8.04965 4.26118

H 11.28093 8.89160 1.98974

H 9.73229 8.69148 0.12055

H 8.38817 7.05633 4.59402

H 6.08228 6.54171 -2.01200

H 4.41115 4.68682 2.23661

H 6.57009 8.57764 -1.55035

H 7.92033 9.37082 -0.76452

H 9.47824 7.53069 -1.57541

H 8.11373 6.73920 -2.37618

H 8.63232 8.35459 -2.90350

H 4.11512 2.41057 -1.53500

H 2.43933 0.61940 -1.48947

H 0.43246 2.59135 1.77939

H 2.11288 4.37631 1.72582

H 8.45031 -8.06696 -7.11531

H 7.64681 -10.24767 -6.18480

H 5.51783 -10.38412 -5.01049

H 7.00680 -6.04813 -6.87222

H 2.05386 -8.36084 -2.46930

H 3.23555 -3.85048 -4.06240

H 2.12713 -9.59206 -4.21644

H 3.40715 -10.33976 -5.14925

H 4.83304 -10.53484 -3.05657

H 3.54863 -9.76604 -2.11452

H 3.25867 -11.34075 -2.88461

H 1.19086 -2.70515 -3.78987

H 0.33095 -0.68258 -2.70584

H 0.91924 -2.51311 1.15463

H 1.77132 -4.53666 0.05941

H -6.80526 0.29943 0.13513

H -5.70108 1.67443 -0.17238

H -7.47192 1.88937 -0.35698

H 0.37361 2.38617 6.55411

H -1.09234 1.81480 5.70059

H -1.16753 2.19790 7.45096

H 2.88195 4.87223 -3.73217

H 2.28303 5.06511 -2.05671

H 3.17264 6.40562 -2.84939

H -0.96968 -6.62051 -0.51136

H -0.70731 -5.43474 -1.82620

H -0.72970 -7.18971 -2.19476

SCF Done: E(RCAM-B3LYP) = -5641.49249271 A.U. after 1 cycles

Zero-point correction= 1.369318 (Hartree/Particle)

Thermal correction to Energy= 1.455541

Thermal correction to Enthalpy= 1.456485

Thermal correction to Gibbs Free Energy= 1.228771

Sum of electronic and zero-point Energies= -5640.123174

Sum of electronic and thermal Energies= -5640.036952

Sum of electronic and thermal Enthalpies= -5640.036008

Sum of electronic and thermal Free Energies= -5640.263721

6.6.2 Computed excitation energies of compound 5g (cam-B3LYP/def2svp SMD CH_2Cl_2 GD3BJ)

Excitation energies and oscillator strengths:

Excited State 1: Singlet-A 3.7492 eV 330.70 nm f=0.3074 <S**2>=0.000

354 -> 359 -0.10617

354 -> 361 -0.12016

355 -> 361 -0.14244

356 -> 360 -0.15282

357 -> 358 0.28500

357 -> 359 0.32539

357 -> 361 0.27687

357 -> 362 0.11732

357 -> 363 0.12093

357 -> 364 -0.16607

357 -> 365 -0.13630

357 -> 374 -0.11104

This state for optimization and/or second-order correction.

Total Energy, E(TD-HF/TD-DFT) = -5641.35469490

Copying the excited state density for this state as the 1-particle RhoCI density.

Excited State 2: Singlet-A 3.7546 eV 330.22 nm f=0.3494 <S**2>=0.000

354 -> 359 -0.25013

354 -> 361 0.24342

354 -> 364 0.11205

355 -> 358 -0.21204

355 -> 359 0.24553

355 -> 362 -0.13857

356 -> 358 0.17042

356 -> 359 -0.12729

356 -> 360 0.21614

356 -> 363 0.13826

Excited State 3: Singlet-A 3.7550 eV 330.18 nm f=0.3390 <S**2>=0.000

354 -> 359 -0.11578

354 -> 360 0.27810

354 -> 363 0.11669

355 -> 358 -0.18734

355 -> 360 0.25629

355 -> 362 -0.12712

356 -> 358 -0.23498

356 -> 361 0.26707

356 -> 363 -0.10452

356 -> 365 -0.10025

357 -> 360 0.10514

Excited State 4: Singlet-A 3.7822 eV 327.81 nm f=0.0014 <S**2>=0.000

354 -> 358 -0.26147

354 -> 359 0.10845

354 -> 362 -0.16243

355 -> 359 -0.21160

355 -> 360 0.10089

355 -> 361 0.18748

355 -> 364 0.13910

356 -> 360 0.27379

356 -> 361 -0.11798

356 -> 363 0.15260

357 -> 359 0.14655

357 -> 361 0.18867

357 -> 365 -0.10099

Excited State 5: Singlet-A 4.3034 eV 288.11 nm f=0.5331 <S**2>=0.000

354 -> 363 -0.11945

354 -> 364 -0.17286

354 -> 365 0.17404

355 -> 362 0.15283

355 -> 363 -0.11021

355 -> 365 0.11799

355 -> 366 0.24262

355 -> 372 0.11589

356 -> 364 -0.13326

357 -> 364 -0.21397

357 -> 365 -0.15990

357 -> 374 0.11039

Excited State 6: Singlet-A 4.3054 eV 287.97 nm f=0.4298 <S**2>=0.000

354 -> 364 -0.23147

354 -> 366 -0.10468

355 -> 362 0.12168

355 -> 366 0.20914

356 -> 365 0.12418

357 -> 363 -0.18156

357 -> 364 0.19597

357 -> 365 0.28718

357 -> 374 -0.13209

Excited State 7: Singlet-A 4.3065 eV 287.90 nm f=0.4658 <S**2>=0.000

354 -> 363 -0.16425

354 -> 365 -0.15821

354 -> 366 0.12866

355 -> 363 -0.10501

355 -> 364 -0.19082

356 -> 358 -0.12481

356 -> 363 0.31828

356 -> 365 0.22068

356 -> 366 0.16040

356 -> 373 0.14598

Excited State 8: Singlet-A 4.3312 eV 286.26 nm f=0.0144 <S**2>=0.000

354 -> 358 -0.11894

354 -> 362 0.10281

354 -> 363 0.11055

354 -> 366 0.23617

355 -> 364 -0.24061

355 -> 365 0.11490

356 -> 363 -0.22769

356 -> 365 -0.12759

357 -> 364 0.11367

357 -> 365 0.22917

Excited State 9: Singlet-A 4.4765 eV 276.96 nm f=0.1520 <S**2>=0.000

354 -> 369 0.16131

356 -> 369 -0.19644

357 -> 369 -0.11574

357 -> 370 0.49995

357 -> 374 -0.13155

Excited State 10: Singlet-A 4.4779 eV 276.88 nm f=0.2309 <S**2>=0.000

354 -> 367 -0.20739

354 -> 368 -0.15004

354 -> 369 -0.14720

355 -> 368 -0.25521

356 -> 366 0.14347

356 -> 369 0.40726

356 -> 370 0.10099

356 -> 373 -0.11097

357 -> 370	0.11388

6.6.3 Computed emission energies of compound 5g (cam-B3LYP/def2svp SMD CH_2Cl_2 GD3BJ)

Excitation energies and oscillator strengths:

Excited State 1: Singlet-A 2.7996 eV 442.87 nm f=0.1517 <S**2>=0.000

357 -> 358 0.60705

357 -> 359 -0.15516

357 -> 363 -0.24289

This state for optimization and/or second-order correction.

Total Energy, E(TD-HF/TD-DFT) = -5641.32850051

Copying the excited state density for this state as the 1-particle RhoCI density.

Excited State 2: Singlet-A 3.7539 eV 330.28 nm f=0.2895 <S**2>=0.000

354 -> 359 -0.15214

354 -> 360 0.39551

354 -> 361 -0.19683

354 -> 362 0.11567

354 -> 364 -0.16186

355 -> 359 0.22582

355 -> 360 -0.13480

355 -> 361 0.11542

355 -> 362 -0.12737

356 -> 361 0.12475

Excited State 3: Singlet-A 3.7631 eV 329.47 nm f=0.2759 <S**2>=0.000

354 -> 361 0.19882

355 -> 360 0.16353

355 -> 361 0.30793

355 -> 365 -0.12074

356 -> 359 0.31651

356 -> 360 0.24635

356 -> 362 -0.14281

356 -> 364 -0.17766

Excited State   4:   Singlet-A   3.7756 eV 328.38 nm f=0.3007 <S**2>=0.000

354 -> 359   0.19530

354 -> 362   -0.10444

355 -> 359   0.20465

355 -> 360   0.17702

355 -> 361   -0.10364

355 -> 364   -0.12595

356 -> 361   0.29339

356 -> 365   -0.12271

357 -> 358   0.10993

357 -> 363   0.30026

357 -> 365   -0.10981

357 -> 377   -0.11856

Excited State   5:   Singlet-A   3.8129 eV 325.17 nm f=0.1388 <S**2>=0.000

354 -> 359   -0.13779

355 -> 360   -0.14108

355 -> 364   0.11003

356 -> 361   -0.17849

357 -> 358   0.16769

357 -> 363   0.43291

357 -> 365   -0.16040

357 -> 377   -0.19496

Excited State   6:   Singlet-A   4.1957 eV 295.50 nm f=0.0688 <S**2>=0.000

357 -> 370   -0.10007

357 -> 373   -0.14188

357 -> 374   -0.14896

357 -> 376   0.56796

357 -> 377   -0.21990

external emission: -5641.376278 - -5641.476606 = 0.100328 = 2.730052 eV 454.15 nm

6.7 Quantum chemical calculation data of tetrakis(4-(7-(4-chlorophenyl)-10-hexyl-10*H*-phenothiazin-3-yl)phenyl)silane (5h)

6.7.1 Computed xyz-coordinates of compound 5h (cam-B3LYP/def2svp SMD CH_2Cl_2 GD3BJ)

0 1

C -8.31129 -9.59504 -3.67776

C -7.63132 -10.12121 -4.77802

C -6.57577 -9.44135 -5.37461

C -6.15841 -8.18292 -4.91722

C -6.87058 -7.63763 -3.83146

C -7.89985 -8.33903 -3.21635

S -6.57625 -5.96963 -3.32109

N -5.09285 -7.49336 -5.51721

C -4.85103 -5.86339 -3.69885

C -4.32232 -6.56417 -4.80009

C -2.99415 -6.27870 -5.14849

C -2.23229 -5.36440 -4.42999

C -2.74449 -4.70495 -3.31067

C -4.07181 -4.98103 -2.96147

C -4.59818 -7.96063 -6.81054

C -3.58872 -9.10487 -6.77449

C -9.42270 -10.32756 -3.02345

C -9.65507 -10.20904 -1.64634

C -10.69485 -10.89238 -1.02625

C -11.52056 -11.71277 -1.78836

C -11.31525 -11.85280 -3.15731

C -10.27292 -11.16091 -3.76318

Cl -12.82725 -12.57432 -1.02007

C -1.92244 -3.75670 -2.52035

C -0.93622 -2.96996 -3.13204

C -0.15983 -2.09074 -2.38629

C -0.32934 -1.95826 -0.99944

C -1.32343 -2.73946 -0.39633

C -2.10349 -3.62085 -1.13801

Si 0.78114 -0.80894 -0.00460

C -5.97347 4.61134 9.92456

C -5.34073 3.95010 10.97921

C -4.58580 2.80166 10.76986

C -4.44401 2.23650 9.49407

C -5.11835 2.88136 8.43909

C -5.83850 4.05042 8.64898

S -5.19964 2.12686 6.84105

N -3.67948 1.07940 9.27775

C -3.62758 1.31538 6.83556

C -3.08499 0.81301 8.03397

C -1.93050 0.02503 7.92002

C -1.34361 -0.23500 6.68693

C -1.86135 0.29644 5.50371

C -3.01479 1.08271 5.61095

C -3.26004 0.29296 10.43581

C -1.99842 0.76543 11.15297

C -6.75682 5.85258 10.13770

C -6.82015 6.84507 9.15009

C -7.55119 8.01178 9.34196

C -8.23257 8.19890 10.54045

C -8.18909 7.23297 11.54092

C -7.45566 6.07057 11.33289

Cl -9.15114 9.65940 10.79145

C -1.22509 0.04222 4.18826

C -0.55330 -1.16118 3.93058

C 0.04786 -1.39422 2.69943

C -0.00130 -0.44396 1.66787

C -0.67266 0.75673 1.93196

C -1.27125 0.99748 3.16464

C -1.31383 9.76959 -7.58870

C -0.31839 10.59319 -7.05937

C 0.71969 10.07816 -6.29134

C 0.83320 8.70408 -6.03275

C -0.14926 7.87136 -6.60306

C -1.20604 8.39707 -7.33545

S 0.02518 6.11209 -6.54154

N 1.87907 8.18133 -5.25610

C 0.85710 5.96537 -4.98715

C 1.74658 6.96965 -4.55901

C 2.50199 6.69244 -3.40995

C 2.37192 5.49054 -2.72362

C 1.46244 4.51162 -3.12914

C 0.70327 4.78357 -4.27350

C 3.00820 9.05004 -4.93119

C 2.81993 9.97295 -3.73065

C -2.43638 10.31606 -8.38913

C -3.71027 9.73388 -8.33920

C -4.76721 10.24047 -9.08686

C -4.55581 11.34941 -9.90009

C -3.30288 11.95017 -9.97135

C -2.25561 11.43071 -9.21922

Cl -5.87498 11.99377 -10.84072

C 1.30613 3.23619 -2.38866

C 2.39378 2.64189 -1.73584

C 2.24635 1.44479 -1.04275

C 1.01041 0.78904 -0.97193

C -0.07421 1.38607 -1.63242

C 0.06686 2.58391 -2.32328

C 13.73468 -2.84147 1.34474

C 13.74564 -4.21806 1.11103

C 12.58263 -4.97683 1.17727

C 11.34625 -4.40257 1.50841

C 11.34537 -3.02083 1.78111

C 12.50373 -2.26166 1.67382

S 9.89766 -2.23916 2.43057

N 10.17070 -5.16642 1.57793

C 8.66165 -3.23121 1.64547

C 8.90501 -4.59154 1.37621

C 7.81731 -5.34438 0.90954

C 6.56587 -4.77306 0.71017

C 6.33643 -3.41553 0.94380

C 7.41757 -2.65881 1.41044

C 10.28488 -6.62323 1.56546

C 10.41021 -7.27549 0.19166

C 14.96937 -2.02457 1.25523

C 14.92743 -0.69571 0.81176

C 16.08092 0.07537 0.72550

C 17.30267 -0.48467 1.08515

C 17.37785 -1.80115 1.52905

C 16.21510 -2.55848 1.61208

Cl 18.75525 0.47382 0.97901

C 5.00636 -2.79929 0.71793

C 4.16042 -3.26092 -0.29865

C 2.91020 -2.68740 -0.50612

C 2.45139 -1.62778 0.28718

C 3.30233 -1.17093 1.30522

C 4.55234 -1.74084 1.51726

H -7.90204 -11.10336 -5.16877

H -6.06907 -9.91914 -6.20869

H -8.42012 -7.86814 -2.38010

H -2.52632 -6.78088 -5.99102

H -1.19939 -5.19436 -4.73806

H -4.53116 -4.46549 -2.11604

H -5.47221 -8.25292 -7.40854

H -4.16139 -7.09410 -7.32530

H -3.29507 -9.36350 -7.80317

H -2.67768 -8.83869 -6.22160

H -4.00155 -10.00826 -6.30554

H -9.00009 -9.58466 -1.03613

H -10.86006 -10.79529 0.04767

H -11.97322 -12.49267 -3.74689

H -10.13369 -11.26019 -4.84101

H -0.78723 -3.03076 -4.21182

H 0.58977 -1.48655 -2.90470

H -1.49608 -2.66757 0.68012

H -2.85347 -4.22789 -0.62719

H -5.40307 4.35118 11.99212

H -4.09409 2.35190 11.62828

H -6.34159 4.50926 7.79570

H -1.46212 -0.39466 8.80630

H -0.43771 -0.84278 6.66243

H -3.47893 1.49436 4.71278

H -4.10355 0.27354 11.13931

H -3.13018 -0.74393 10.09714

H -1.79606 0.10216 12.00765

H -1.11651 0.74788 10.49840

H -2.09795 1.78933 11.53799

H -6.27206 6.71711 8.21506

H -7.58596 8.77852 8.56672

H -8.73355 7.38630 12.47370

H -7.44530 5.30941 12.11487

H -0.51305 -1.93493 4.69957

H 0.56506 -2.34466 2.54194

H -0.72443 1.53320 1.16500

H -1.76413 1.95587 3.33932

H -0.36116 11.67241 -7.21565

H 1.44598 10.77693 -5.88467

H -1.93977 7.70643 -7.75552

H 3.20706 7.42456 -3.02501

H 2.97801 5.33559 -1.82955

H 0.00062 4.03805 -4.65057

H 3.88225 8.40419 -4.77274

H 3.23498 9.64293 -5.82757

H 3.73313 10.56887 -3.58146

H 1.98243 10.66982 -3.87064

H 2.62777 9.41400 -2.80481

H -3.89085 8.87689 -7.68794

H -5.75538 9.78152 -9.03249

H -3.14594 12.81396 -10.61879

H -1.27100 11.89456 -9.29855

H 3.37841 3.11031 -1.78877

H 3.12328 1.00992 -0.55718

H -1.05790 0.90914 -1.60849

H -0.80555 3.02753 -2.80701

H 14.67571 -4.71873 0.83639

H 12.65481 -6.03833 0.95583

H 12.44559 -1.19482 1.89833

H 7.93230 -6.40432 0.69890

H 5.75051 -5.41385 0.37043

H 7.29303 -1.59163 1.60337

H 11.15222 -6.88862 2.18515

H 9.40884 -7.02555 2.09219

H 10.49022 -8.36660 0.31164

H 9.54031 -7.06999 -0.44661

H 11.30081 -6.93033 -0.35072

H 13.97695 -0.25408 0.50735

H 16.03407 1.10628 0.37188

H 18.33979 -2.22788 1.81652

H 16.28106 -3.58273 1.98277

H 4.49029 -4.07264 -0.94991

H 2.28261 -3.07889 -1.31019

H 2.98093 -0.35724 1.96110

H 5.17606 -1.37197 2.33386

SCF Done: E(RCAM-B3LYP) = -7944.92525105 A.U. after 1 cycles

Zero-point correction= 1.531015 (Hartree/Particle)

Thermal correction to Energy= 1.629266

Thermal correction to Enthalpy= 1.630210

Thermal correction to Gibbs Free Energy= 1.371321

Sum of electronic and zero-point Energies= -7943.394236

Sum of electronic and thermal Energies= -7943.295985

Sum of electronic and thermal Enthalpies= -7943.295041

Sum of electronic and thermal Free Energies= -7943.553930

6.7.2 Computed excitation energies of compound 5h (cam-B3LYP/def2svp SMD CH_2Cl_2 GD3BJ)

Excitation energies and oscillator strengths:

Excited State 1: Singlet-A 3.5492 eV 349.33 nm f=0.4296 <S**2>=0.000

434 -> 439 -0.11231

434 -> 440 -0.14295

434 -> 441 -0.12066

435 -> 438 0.13269

435 -> 439 0.14834

435 -> 440 -0.10414

436 -> 438 0.10998

436 -> 439 0.13347

436 -> 441 0.12209

437 -> 438 0.26731

437 -> 439 0.29832

437 -> 440 0.22020

437 -> 441 0.23400

437 -> 463 -0.11054

This state for optimization and/or second-order correction.

Total Energy, E(TD-HF/TD-DFT) = -7944.79482026

Copying the excited state density for this state as the 1-particle RhoCI density.

Excited State 2: Singlet-A 3.5558 eV 348.68 nm f=0.5438 <S**2>=0.000

434 -> 439 -0.29464

434 -> 440 0.11672

434 -> 441 0.20309

435 -> 438 -0.30938

435 -> 440 0.31747

435 -> 464 0.11297

436 -> 441 0.20289

437 -> 438 0.12920

Excited State 3: Singlet-A 3.5612 eV 348.16 nm f=0.9259 <S**2>=0.000

434 -> 440 -0.22436

434 -> 441 0.21278

435 -> 439 0.17618

435 -> 441 -0.16567

436 -> 438 0.27437

436 -> 439 -0.21897

436 -> 440 0.17702

436 -> 441 -0.18128

436 -> 462 0.11218

437 -> 440 -0.15292

437 -> 441 0.12829

Excited State 4: Singlet-A 3.5883 eV 345.52 nm f=0.0278 <S**2>=0.000

434 -> 438 0.29690

434 -> 439 -0.11279

435 -> 439 0.17021

435 -> 440 -0.13149

435 -> 441 -0.21762

436 -> 440 -0.29566

436 -> 441 0.18670

437 -> 439 -0.22512

437 -> 441 -0.16221

Excited State 5: Singlet-A 4.2556 eV 291.35 nm f=0.6594 <S**2>=0.000

434 -> 447 0.13060

434 -> 451 -0.12877

436 -> 446 -0.10073

436 -> 447 -0.26829

436 -> 451 0.33072

436 -> 454 0.15143

437 -> 447 0.14136

437 -> 451 -0.13662

437 -> 454 -0.12018

Excited State 6: Singlet-A 4.2781 eV 289.81 nm f=0.4205 <S**2>=0.000

434 -> 454 -0.10230

435 -> 452 0.21083

435 -> 458 0.12978

436 -> 453 0.10953

437 -> 450 0.27540

437 -> 453 0.26115

437 -> 454 0.15322

437 -> 455 0.16512

Excited State 7: Singlet-A 4.2808 eV 289.63 nm f=0.5306 <S**2>=0.000

434 -> 448 -0.15991

434 -> 452 0.24509

434 -> 453 -0.13081

434 -> 457 0.15625

435 -> 446 0.17495

435 -> 449 0.14665

435 -> 451 0.12286

435 -> 452 0.11642

435 -> 453 0.12551

435 -> 454 -0.13248

435 -> 458 0.19419

436 -> 452 0.10482

436 -> 453 -0.12381

437 -> 450 -0.10251

437 -> 453 -0.12593

Excited State   8:   Singlet-A   4.2928 eV 288.82 nm f=0.1840 <S**2>=0.000

434 -> 446   -0.14534

434 -> 449   -0.17834

434 -> 453   -0.11415

434 -> 457   0.10975

434 -> 458   -0.16278

435 -> 448   0.21131

435 -> 449   -0.15268

435 -> 452   -0.11240

435 -> 457   -0.17638

435 -> 458   -0.14537

437 -> 450   0.16323

437 -> 455   0.11750

Excited State   9:   Singlet-A   4.2983 eV 288.45 nm f=0.0386 <S**2>=0.000

434 -> 443   0.11670

434 -> 444   -0.10255

434 -> 445   -0.14125

434 -> 448   -0.19248

434 -> 452   -0.12916

434 -> 457   0.15248

435 -> 442   -0.19388

435 -> 443   0.18809

435 -> 449   0.23192

435 -> 452   -0.16589

435 -> 454   0.11158

435 -> 456   0.14001

435 -> 458   0.11073

436 -> 448   -0.11590

Excited State 10:   Singlet-A   4.2996 eV 288.36 nm f=0.0558 <S**2>=0.000

434 -> 445	-0.11876
434 -> 447	-0.13218
434 -> 455	-0.10186
436 -> 442	0.13318
436 -> 443	0.19028
436 -> 445	0.12907
436 -> 446	-0.11094
436 -> 447	0.28005
436 -> 451	0.15782
436 -> 454	-0.11996
436 -> 455	0.19581
437 -> 443	-0.11705
437 -> 447	-0.14292

6.7.3 Computed emission energies of compound 5h (cam-B3LYP/def2svp SMD CH_2Cl_2 GD3BJ)

Excitation energies and oscillator strengths:

Excited State 1: Singlet-A 2.7148 eV 456.70 nm f=0.3163 <S**2>=0.000

437 -> 438 0.65555

437 -> 465 0.11834

This state for optimization and/or second-order correction.

Total Energy, E(TD-HF/TD-DFT) = -7944.74255677

Copying the excited state density for this state as the 1-particle RhoCI density.

Excited State 2: Singlet-A 3.5753 eV 346.78 nm f=0.4338 <S**2>=0.000

434 -> 440 0.21641

434 -> 441 -0.25969

435 -> 440 -0.23033

435 -> 441 0.14923

436 -> 439 0.39958

436 -> 441 0.22271

436 -> 463 0.10893

Excited State 3: Singlet-A 3.5852 eV 345.82 nm f=0.7626 <S**2>=0.000

434 -> 439 -0.10169

434 -> 440 -0.31355

434 -> 441 -0.18295

435 -> 439 0.31891

435 -> 441 -0.22210

436 -> 440 -0.23935

436 -> 441 0.20366

Excited State 4: Singlet-A 3.6127 eV 343.19 nm f=0.0685 <S**2>=0.000

434 -> 439 -0.32283

434 -> 441 0.15426

435 -> 440 0.35242

435 -> 441 0.20486

436 -> 440 -0.10929

436 -> 441 0.26471

Excited State 5: Singlet-A 3.8224 eV 324.36 nm f=0.4059 <S**2>=0.000

433 -> 438 -0.14424

437 -> 438 -0.12260

437 -> 446 0.45482

437 -> 447 0.20720

437 -> 453 0.21184

437 -> 457 -0.18303

437 -> 460 -0.21598

Excited State 6: Singlet-A 3.8857 eV 319.08 nm f=0.0651 <S**2>=0.000

437 -> 442 0.47607

437 -> 443 0.26661

437 -> 445 -0.13294

437 -> 447 -0.18126

437 -> 453 0.13453

437 -> 460 0.16351

437 -> 461 0.19639

corrected external emission excitation: -7944.813521 - -7944.911068 = 0.097547 = 2.654384 eV 467.09 nm

6.8 Quantum chemical calculation data of tetrakis(4-(7-(4-fluorophenyl)-10-hexyl-10*H*-phenothiazin-3-yl)phenyl)silane (5i)

6.8.1 Computed xyz-coordinates of compound 5i (cam-B3LYP/def2svp SMD CH_2Cl_2 GD3BJ)

0 1

C -8.41515 -9.53329 -3.50095

C -7.73781 -10.08023 -4.59261

C -6.67863 -9.41526 -5.20014

C -6.25417 -8.15240 -4.76231

C -6.96224 -7.58716 -3.68448

C -7.99604 -8.27296 -3.05903

S -6.65721 -5.91398 -3.19762

N -5.18517 -7.47718 -5.37388

C -4.93219 -5.82219 -3.58001

C -4.40883 -6.54198 -4.67166

C -3.07988 -6.26784 -5.02653

C -2.31279 -5.34564 -4.32387

C -2.82005 -4.66657 -3.21407

C -4.14764 -4.93205 -2.85777

C -4.69543 -7.96575 -6.66099

C -3.69128 -9.11411 -6.60992

C -9.53201 -10.24873 -2.83561

C -9.75007 -10.12293 -1.45627

C -10.79451 -10.79029 -0.82554

C -11.62818 -11.59496 -1.58641

C -11.44712 -11.74755 -2.95225

C -10.39983 -11.07100 -3.56822

F -12.63668 -12.24262 -0.98546

C -1.99335 -3.70766 -2.44169

C -1.00816 -2.93257 -3.06974

C -0.22888 -2.04130 -2.34167

C -0.39387 -1.88483 -0.95673

C -1.38621 -2.65525 -0.33714

C -2.16948 -3.54849 -1.06123

Si 0.71661 -0.71388 0.01270

C -1.56211 9.69551 -7.74600

C -0.57887 10.54379 -7.23330

C 0.47018 10.05903 -6.46014

C 0.60678 8.69189 -6.17802

C -0.36411 7.83397 -6.72981

C -1.43099 8.32969 -7.46887

S -0.16254 6.07895 -6.63577

N 1.66446 8.19954 -5.39632

C 0.67723 5.97425 -5.08217

C 1.55357 6.99941 -4.67659

C 2.31845 6.75415 -3.52640

C 2.20918 5.56325 -2.81749

C 1.31196 4.56413 -3.20037

C 0.54381 4.80396 -4.34592

C 2.78026 9.09230 -5.09203

C 2.58157 10.03392 -3.90774

C -2.69518 10.21002 -8.55406

C -3.96000 9.60860 -8.48874

C -5.02651 10.08396 -9.24417

C -4.82254 11.17648 -10.07259

C -3.58851 11.80109 -10.16570

C -2.53166 11.31170 -9.40574

F -5.84589 11.64139 -10.80357

C 1.17677 3.30014 -2.43649

C 2.27638 2.72924 -1.78276

C 2.14837 1.54165 -1.06959

C 0.92080 0.87282 -0.97860

C -0.17603 1.44696 -1.63918

C -0.05438 2.63501 -2.35024

C -6.02142 4.76342 9.92309

C -5.37112 4.12479 10.98094

C -4.60386 2.98253 10.78115

C -4.46541 2.40158 9.51230

C -5.15583 3.02376 8.45448

C -5.88972 4.18626 8.65451

S -5.23915 2.24767 6.86686

N -3.68828 1.25038 9.30536

C -3.65755 1.45491 6.86020

C -3.10017 0.97454 8.06099

C -1.93772 0.19801 7.94827

C -1.35745 -0.07179 6.71414

C -1.89003 0.43836 5.52814

C -3.05135 1.21315 5.63407

C -3.25028 0.48534 10.47060

C -1.98872 0.98265 11.17101

C -6.81972 5.99769 10.12593

C -6.89119 6.98214 9.13008

C -7.63626 8.14219 9.31200

C -8.31552 8.31813 10.50745

C -8.27068 7.37039 11.51808

C -7.52346 6.21455 11.31905

F -9.03519 9.43435 10.69117

C -1.26146 0.17343 4.21113

C -0.58145 -1.02739 3.96308

C 0.01159 -1.27087 2.73002

C -0.05400 -0.33401 1.68719

C -0.73327 0.86428 1.94171

C -1.32392 1.11544 3.17615

C 13.70111 -2.60026 1.28326

C 13.72378 -3.97796 1.05724

C 12.56924 -4.74853 1.13827

C 11.32980 -4.18517 1.47584

C 11.31681 -2.80198 1.73917

C 12.46682 -2.03168 1.61902

S 9.86531 -2.03038 2.39250

N 10.16241 -4.96111 1.56039

C 8.63421 -3.04118 1.62368

C 8.88962 -4.40124 1.36365

C 7.80588 -5.16901 0.91199

C 6.54720 -4.61207 0.71770

C 6.30563 -3.25496 0.94139

C 7.38263 -2.48343 1.39326

C 10.29225 -6.41646 1.55729

C 10.41412 -7.07718 0.18720

C 14.92733 -1.77106 1.17966

C 14.86895 -0.44867 0.71738

C 16.01422 0.33401 0.61822

C 17.23239 -0.21672 0.98450

C 17.33436 -1.51985 1.44600

C 16.17917 -2.28817 1.54202

F 18.34136 0.53086 0.89020

C 4.96757 -2.65440 0.72044

C 4.11721 -3.13612 -0.28309

C 2.85953 -2.57751 -0.48591

C 2.39718 -1.51322 0.29905

C 3.25254 -1.03620 1.30407

C 4.50996 -1.59132 1.51156

H -8.01437 -11.06705 -4.96765

H -6.17496 -9.90849 -6.02717

H -8.51364 -7.78662 -2.22992

H -2.61569 -6.78505 -5.86194

H -1.27991 -5.18447 -4.63676

H -4.60318 -4.40143 -2.01962

H -5.57207 -8.26297 -7.25268

H -4.25550 -7.10909 -7.18953

H -3.40079 -9.38953 -7.63516

H -2.77794 -8.84393 -6.06275

H -4.10751 -10.00834 -6.12667

H -9.08024 -9.50559 -0.85505

H -10.96400 -10.70042 0.24885

H -12.12924 -12.38304 -3.51970

H -10.26879 -11.17268 -4.64689

H -0.86283 -3.01181 -4.14880

H 0.51900 -1.44630 -2.87303

H -1.55528 -2.56479 0.73848

H -2.91851 -4.14595 -0.53780

H -0.63992 11.61926 -7.40831

H 1.18670 10.77615 -6.06828

H -2.15476 7.62003 -7.87445

H 3.01461 7.50337 -3.15843

H 2.82174 5.43354 -1.92380

H -0.14939 4.04135 -4.70583

H 3.66571 8.46416 -4.92554

H 2.99347 9.67251 -5.99997

H 3.48378 10.65037 -3.77601

H 1.72963 10.71147 -4.05518

H 2.40652 9.48908 -2.97018

H -4.12389 8.76198 -7.81981

H -6.01457 9.62354 -9.19112

H -3.46421 12.65365 -10.83563

H -1.55368 11.78788 -9.49403

H 3.25504 3.20810 -1.85137

H 3.03413 1.12455 -0.58445

H -1.15384 0.95912 -1.59952

H -0.93564 3.06071 -2.83404

H -5.42988 4.53936 11.98871

H -4.09985 2.55069 11.64171

H -6.40561 4.62686 7.79918

H -1.45780 -0.20470 8.83627

H -0.44507 -0.66985 6.69043

H -3.52694 1.60762 4.73416

H -4.08790 0.46563 11.18112

H -3.11051 -0.55459 10.14542

H -1.77138 0.33365 12.03294

H -1.11192 0.96684 10.50957

H -2.09754 2.01043 11.54293

H -6.33885 6.85156 8.19791

H -7.68903 8.91408 8.54218

H -8.82523 7.54051 12.44257

H -7.50418 5.45761 12.10498

H -0.52848 -1.79098 4.74142

H 0.53532 -2.21895 2.57998

H -0.79796 1.63040 1.16535

H -1.82363 2.07168 3.34302

H 14.65683 -4.47052 0.77786

H 12.65071 -5.81070 0.92315

H 12.39959 -0.96428 1.83839

H 7.92979 -6.22954 0.70948

H 5.73590 -5.26405 0.38982

H 7.24879 -1.41597 1.57826

H 11.16715 -6.66812 2.17209

H 9.42469 -6.82461 2.09352

H 10.50564 -8.16660 0.31409

H 9.53767 -6.88454 -0.44614

H 11.29735 -6.72720 -0.36404

H 13.91226 -0.02325 0.40935

H 15.97415 1.36140 0.25231

H 18.30980 -1.91603 1.73354

H 16.25423 -3.30687 1.92610

H 4.44933 -3.95206 -0.92789

H 2.22880 -2.98470 -1.27964

H 2.92879 -0.21815 1.95334

H 5.13700 -1.20686 2.31835

SCF Done: E(RCAM-B3LYP) = -6503.60346023 A.U. after 1 cycles

Zero-point correction= 1.536379 (Hartree/Particle)

Thermal correction to Energy= 1.633078

Thermal correction to Enthalpy= 1.634022

Thermal correction to Gibbs Free Energy= 1.379260

Sum of electronic and zero-point Energies= -6502.067082

Sum of electronic and thermal Energies= -6501.970382

Sum of electronic and thermal Enthalpies= -6501.969438

Sum of electronic and thermal Free Energies= -6502.224200

6.8.2 Computed excitation energies of compound 5i (cam-B3LYP/def2svp SMD CH_2Cl_2 GD3BJ)

Excitation energies and oscillator strengths:

Excited State 1: Singlet-A 3.5697 eV 347.32 nm f=0.4188 <S**2>=0.000

418 -> 424 -0.15082

418 -> 425 0.11661

419 -> 422 0.12472

419 -> 423 0.16515

420 -> 423 0.11182

420 -> 425 -0.11829

421 -> 422 0.27552

421 -> 423 0.27574

421 -> 424 0.26174

421 -> 425 -0.23614

421 -> 447 -0.10401

This state for optimization and/or second-order correction.

Total Energy, E(TD-HF/TD-DFT) = -6503.47227539

Copying the excited state density for this state as the 1-particle RhoCI density.

Excited State 2: Singlet-A 3.5773 eV 346.59 nm f=0.5033 <S**2>=0.000

418 -> 423 -0.29472

418 -> 425 -0.20533

419 -> 422 -0.29974

419 -> 424 0.32324

419 -> 426 0.10722

419 -> 448 0.10469

420 -> 425 -0.20175

421 -> 422 0.12232

Excited State 3: Singlet-A 3.5823 eV 346.11 nm f=0.8318 <S**2>=0.000

418 -> 424 -0.23208

418 -> 425 -0.22127

419 -> 423 0.15773

419 -> 425 0.18232

420 -> 422 0.28365

420 -> 423 -0.26046

420 -> 424 0.14412

420 -> 425 0.15592

420 -> 426 -0.10748

420 -> 446 0.10936

421 -> 424 -0.13334

421 -> 425 -0.12606

Excited State 4: Singlet-A 3.6099 eV 343.46 nm f=0.0190 <S**2>=0.000

418 -> 422 0.29057

418 -> 423 -0.10331

418 -> 426 -0.12249

419 -> 423 0.18817

419 -> 424 -0.12174

419 -> 425 0.20995

420 -> 424 -0.27793

420 -> 425 -0.20997

421 -> 423 -0.22153

421 -> 425 0.14038

Excited State 5: Singlet-A 4.2516 eV 291.61 nm f=0.6250 <S**2>=0.000

418 -> 431 0.10171

418 -> 435 -0.11856

420 -> 427 0.15843

420 -> 429 -0.10833

420 -> 430 -0.12859

420 -> 431 -0.21049

420 -> 435 0.32082

420 -> 436 0.11836

420 -> 438 0.15662

421 -> 435 -0.10685

Excited State 6: Singlet-A 4.2767 eV 289.90 nm f=0.4079 <S**2>=0.000

418 -> 436 0.15954

419 -> 436 0.26984

419 -> 442 0.15788

421 -> 434 0.17723

421 -> 437 0.23138

421 -> 439 0.14187

Excited State 7: Singlet-A 4.2781 eV 289.81 nm f=0.4698 <S**2>=0.000

418 -> 435 0.16543

418 -> 436 -0.20949

418 -> 437 0.10486

419 -> 426 -0.10291

419 -> 435 -0.13331

419 -> 437 -0.11770

419 -> 442 -0.16376

420 -> 437 0.15447

421 -> 434 0.13443

421 -> 437 0.22406

Excited State 8: Singlet-A 4.2986 eV 288.43 nm f=0.2044 <S**2>=0.000

418 -> 422 0.10022

418 -> 435 0.14381

418 -> 437 0.20006

418 -> 442 0.14520

419 -> 436 0.26433

419 -> 437 -0.10654

419 -> 441 0.10631
420 -> 442 0.10233
421 -> 434 -0.13357
421 -> 435 0.12881
421 -> 437 -0.14109
421 -> 439 -0.10756

Excited State 9: Singlet-A 4.3118 eV 287.55 nm f=0.0161 <S**2>=0.000
418 -> 441 0.10373
419 -> 432 0.14733
421 -> 428 -0.19643
421 -> 429 -0.15765
421 -> 430 0.16448
421 -> 433 -0.13850
421 -> 434 0.24787
421 -> 437 -0.12582
421 -> 439 0.13173
421 -> 440 0.15720
421 -> 441 -0.13127
421 -> 442 0.14600

Excited State 10: Singlet-A 4.3122 eV 287.52 nm f=0.0920 <S**2>=0.000
418 -> 427 0.10333
418 -> 432 0.19197
418 -> 441 0.13681
419 -> 426 -0.12108
419 -> 427 0.15534
419 -> 429 0.13217
419 -> 432 0.26590
419 -> 433 0.12709
419 -> 441 0.20582

421 -> 434	-0.11527

6.8.3 Computed emission energies of compound 5i (cam-B3LYP/def2svp SMD CH_2Cl_2 GD3BJ)

Excitation energies and oscillator strengths:

Excited State 1: Singlet-A 2.7323 eV 453.77 nm f=0.2908 <S**2>=0.000

421 -> 422 -0.64253

421 -> 423 -0.11264

421 -> 426 0.11869

421 -> 449 0.11324

This state for optimization and/or second-order correction.

Total Energy, E(TD-HF/TD-DFT) = -6503.42721792

Copying the excited state density for this state as the 1-particle RhoCI density.

Excited State 2: Singlet-A 3.5944 eV 344.94 nm f=0.4157 <S**2>=0.000

418 -> 424 0.19757

418 -> 425 -0.29413

419 -> 424 -0.20791

419 -> 425 0.14607

420 -> 423 0.39558

420 -> 425 0.19226

420 -> 427 -0.10991

420 -> 447 0.10235

Excited State 3: Singlet-A 3.6035 eV 344.07 nm f=0.6801 <S**2>=0.000

418 -> 423 0.11787

418 -> 424 0.31002

418 -> 425 0.15363

419 -> 423 -0.29648

419 -> 425 0.24509

419 -> 426 -0.10519

420 -> 424 0.23589

420 -> 425 -0.19569

Excited State 4: Singlet-A 3.6305 eV 341.50 nm f=0.0785 <S**2>=0.000

418 -> 423 -0.31143

418 -> 425 0.12355

418 -> 426 -0.10678

419 -> 424 0.36238

419 -> 425 0.17134

419 -> 428 -0.10410

420 -> 425 0.28588

Excited State 5: Singlet-A 3.8092 eV 325.49 nm f=0.3803 <S**2>=0.000

417 -> 422 0.12867

421 -> 422 0.14944

421 -> 426 0.20887

421 -> 427 0.20003

421 -> 430 -0.26785

421 -> 431 0.32977

421 -> 437 0.18360

421 -> 441 -0.18885

421 -> 444 -0.18017

421 -> 445 0.14285

Excited State 6: Singlet-A 3.9463 eV 314.18 nm f=0.0458 <S**2>=0.000

421 -> 426 0.33842

421 -> 427 0.25076

421 -> 429 -0.10142

421 -> 431 -0.35518

421 -> 444 0.25392

421 -> 445 0.19266

corrected external emission excitation: -6503.491041 - -6503.589232 = 0.098191 = 2.671923 eV 464.03 nm

6.9 Quantum chemical calculation data of tetrakis(4-(10-hexyl-7-phenyl-10*H*-phenothiazin-3-yl)phenyl)silane (5j)

6.9.1 Computed xyz-coordinates of compound 5j (cam-B3LYP/def2svp SMD CH_2Cl_2 GD3BJ)

0 1

C 6.54709 11.33534 -0.91919

C 6.29329 11.70368 -2.24196

C 5.80133 10.79317 -3.17043

C 5.55982 9.45371 -2.83170

C 5.85132 9.07592 -1.50696

C 6.30624 10.00006 -0.57473

S 5.79820 7.37266 -1.03204

N 5.06418 8.53130 -3.76790

C 4.49686 6.82428 -2.09770

C 4.33160 7.40114 -3.37218

C 3.40812 6.78181 -4.22729

C 2.67938 5.66663 -3.82964

C 2.81208 5.12442 -2.54980

C 3.73601 5.73494 -1.69320

C 5.05039 8.91067 -5.17884

C 3.85763 9.74482 -5.63789

C 7.05258 12.30862 0.08090

C 6.68966 12.20970 1.43155

C 7.16404 13.12228 2.36910

C 8.01111 14.15659 1.97700

C 8.37979 14.26769 0.63796

C 7.90701 13.35341 -0.29869

C 2.01225 3.95528 -2.11080

C 1.63257 2.95572 -3.01776

C 0.87731 1.86489 -2.60355

C 0.46506 1.72216 -1.26958

C 0.85489 2.71996 -0.36700

C 1.61054 3.81402 -0.77627

Si -0.60724 0.26580 -0.74706

C 2.60351 -3.55274 11.44931

C 1.42912 -3.16143 12.09536

C 0.53862 -2.26842 11.50984

C 0.78885 -1.69679 10.25377

C 1.99360 -2.06163 9.62269

C 2.85947 -2.98459 10.19580

S 2.51660 -1.22895 8.15192

N -0.10869 -0.79593 9.65757

C 0.93162 -0.90083 7.43760

C -0.17907 -0.64176 8.26397

C -1.35096 -0.21027 7.62504

C -1.41549 -0.05789 6.24470

C -0.32422 -0.35173 5.42434

C 0.84807 -0.78372 6.05607

C -1.17730 -0.22993 10.47778

C -2.43037 -1.08630 10.63711

C 3.54458 -4.52537 12.05831

C 4.26990 -5.42162 11.26071

C 5.15163 -6.33492 11.83103

C 5.32671 -6.37376 13.21270

C 4.61207 -5.48918 14.01779

C 3.73208 -4.57462 13.44679

C -0.39430 -0.21290 3.94943

C -1.19952 0.76726 3.35221

C -1.26837 0.89009 1.96951

C -0.53295 0.04813 1.12102

C 0.26921 -0.92928 1.72399

C 0.33824 -1.05945 3.10738

C 7.22845 -8.94620 -5.98276

C 6.37033 -10.03817 -5.83722

C 5.00991 -9.86968 -5.60487

C 4.42816 -8.59561 -5.53198

C 5.28883 -7.49677 -5.71767

C 6.65365 -7.67174 -5.90998

S 4.62797 -5.86292 -5.87362

N 3.05429 -8.42130 -5.29922

C 3.22603 -6.02355 -4.80629

C 2.55124 -7.25521 -4.70124

C 1.34695 -7.25365 -3.98205

C 0.84944 -6.09584 -3.39514

C 1.53889 -4.88382 -3.46967

C 2.74338 -4.87960 -4.18331

C 2.17379 -9.58132 -5.41618

C 2.07577 -10.47971 -4.18665

C 8.68483 -9.12122 -6.20922

C 9.61097 -8.20625 -5.68907

C 10.97648 -8.37073 -5.90203

C 11.44721 -9.45556 -6.63865

C 10.53833 -10.37349 -7.16083

C 9.17289 -10.20721 -6.94952

C 1.02211 -3.65076 -2.82771

C -0.35449 -3.41342 -2.72158

C -0.83732 -2.25558 -2.12034

C 0.02994 -1.28550 -1.60126

C 1.40761 -1.52633 -1.71462

C 1.89536 -2.68326 -2.31102

C -13.77707 0.01280 -1.36060

C -13.91644 0.33350 -2.71245

C -12.87186 0.89251 -3.44017

C -11.63525 1.18581 -2.84697

C -11.51085 0.89936 -1.47385

C -12.54555 0.30497 -0.76229

S -10.08585 1.43787 -0.57449

N -10.57756 1.74787 -3.58019

C -8.87795 1.31886 -1.86147

C -9.23833 1.56580 -3.20041

C -8.19077 1.63371 -4.13091

C -6.86492 1.45847 -3.75161

C -6.51380 1.17718 -2.42980

C -7.55493 1.10307 -1.49684

C -10.85801 2.27557 -4.91353

C -10.86881 1.25690 -6.04983

C -14.88019 -0.60866 -0.58599

C -14.61220 -1.52365 0.44191

C -15.64597 -2.10831 1.16741

C -16.97219 -1.79261 0.88001

C -17.25362 -0.88531 -0.13925

C -16.21934 -0.29915 -0.86278

C -5.10253 0.96998 -2.02387

C -4.06035 1.66606 -2.65020

C -2.73756 1.47219 -2.26621

C -2.39663 0.57708 -1.24358

C -3.44495 -0.11387 -0.61743

C -4.76860 0.07486 -0.99823

H 6.44900 12.73561 -2.56075

H 5.59771 11.15152 -4.17601

H 6.51736 9.65260 0.43840

H 3.23673 7.17181 -5.22699

H 1.96479 5.23637 -4.53312

H 3.90219 5.32791 -0.69397

H 5.98496 9.45082 -5.38234

H 5.10775 7.98525 -5.76750

H 3.95538 9.96109 -6.71261

H 2.90320 9.22292 -5.48504

H 3.79338 10.70370 -5.10602

H 6.01008 11.41768 1.75208

H 6.86225 13.02774 3.41479

H 8.38328 14.87327 2.71245

H 9.04927 15.06986 0.31886

H 8.22321 13.44153 -1.33984

H 1.95165 3.01975 -4.05982

H 0.61466 1.10114 -3.34059

H 0.56200 2.65099 0.68332

H 1.87569 4.58081 -0.04584

H 1.17512 -3.58514 13.06843

H -0.37192 -2.02770 12.05219

H 3.78031 -3.23229 9.66426

H -2.24492 0.00864 8.20307

H -2.35877 0.26770 5.80330

H 1.73948 -0.99606 5.46285

H -0.74899 -0.00773 11.46467

H -1.44273 0.74316 10.04250

H -3.15462 -0.55749 11.27523

H -2.91778 -1.29211 9.67454

H -2.21329 -2.05546 11.10618

H 4.12748 -5.41784 10.17828

H 5.70119 -7.02759 11.18940

H 6.01829 -7.09081 13.66055

H 4.74655 -5.50462 15.10185

H 3.19598 -3.87341 14.08923

H -1.76965 1.45632 3.97825

H -1.91192 1.66591 1.54576

H 0.84905 -1.61758 1.10444

H 0.95691 -1.84905 3.53822
H 6.76872 -11.05359 -5.87154
H 4.40184 -10.76053 -5.47058
H 7.27521 -6.78511 -6.04984
H 0.77745 -8.17130 -3.86064
H -0.08661 -6.15769 -2.83780
H 3.30446 -3.95026 -4.29803
H 1.17614 -9.20881 -5.68617
H 2.51709 -10.16682 -6.28008
H 1.39020 -11.31428 -4.39843
H 3.04822 -10.90610 -3.90585
H 1.69048 -9.94214 -3.30975
H 9.26006 -7.36284 -5.09127
H 11.67923 -7.64773 -5.48146
H 12.51885 -9.58517 -6.80553
H 10.89425 -11.22442 -7.74629
H 8.47203 -10.92286 -7.38359
H -1.06207 -4.13426 -3.13581
H -1.91829 -2.10462 -2.06780
H 2.12337 -0.79651 -1.32651
H 2.97401 -2.84434 -2.36099
H -14.85083 0.11450 -3.23194
H -13.03617 1.09120 -4.49590
H -12.39525 0.10509 0.30048
H -8.39911 1.81940 -5.18116
H -6.09331 1.51191 -4.52144
H -7.33049 0.91578 -0.44497
H -11.82629 2.79186 -4.86484
H -10.11510 3.05790 -5.11967
H -11.09054 1.76996 -6.99796
H -9.90121 0.74890 -6.16042

H -11.63018 0.47896 -5.90201

H -13.57924 -1.79796 0.66473

H -15.41233 -2.82310 1.95983

H -17.78377 -2.25209 1.44854

H -18.28911 -0.62406 -0.36946

H -16.45564 0.42585 -1.64399

H -4.28747 2.38966 -3.43551

H -1.95700 2.04509 -2.77249

H -3.22859 -0.82092 0.18796

H -5.55461 -0.49623 -0.50028

SCF Done: E(RCAM-B3LYP) = -6107.03632646 A.U. after 1 cycles

Zero-point correction= 1.568669 (Hartree/Particle)

Thermal correction to Energy= 1.662212

Thermal correction to Enthalpy= 1.663157

Thermal correction to Gibbs Free Energy= 1.416051

Sum of electronic and zero-point Energies= -6105.467658

Sum of electronic and thermal Energies= -6105.374114

Sum of electronic and thermal Enthalpies= -6105.373170

Sum of electronic and thermal Free Energies= -6105.620275

6.9.2 Computed excitation energies of compound 5j (cam-B3LYP/def2svp SMD CH_2Cl_2 GD3BJ)

Excitation energies and oscillator strengths:

Excited State 1: Singlet-A 3.5646 eV 347.82 nm f=0.6484 <S**2>=0.000

402 -> 407 0.12401

402 -> 408 -0.14944

402 -> 409 0.21264

404 -> 406 0.15974

404 -> 407 -0.17221

404 -> 408 -0.14939

405 -> 406 -0.26631

405 -> 407 0.29043

405 -> 408 -0.15746

405 -> 409 0.22370

This state for optimization and/or second-order correction.

Total Energy, E(TD-HF/TD-DFT) = -6106.90532984

Copying the excited state density for this state as the 1-particle RhoCI density.

Excited State 2: Singlet-A 3.5693 eV 347.36 nm f=0.6090 <S**2>=0.000

402 -> 407 -0.22707

402 -> 408 -0.25206

403 -> 406 0.15022

403 -> 408 -0.23179

404 -> 406 0.21729

404 -> 408 -0.12823

404 -> 409 0.29424

405 -> 406 0.17101

405 -> 408 0.10841

Excited State 3: Singlet-A 3.5729 eV 347.01 nm f=0.3845 <S**2>=0.000

402 -> 408 0.20830

402 -> 409 0.21590

403 -> 406 0.25019
403 -> 407 0.31117
403 -> 409 -0.14548
404 -> 406 -0.17347
404 -> 407 -0.10253
404 -> 408 -0.16719
404 -> 409 -0.11538
405 -> 408 -0.14229
405 -> 409 -0.16992

Excited State 4: Singlet-A 3.6017 eV 344.24 nm f=0.1407 <S**2>=0.000
402 -> 406 -0.29777
402 -> 410 0.11245
403 -> 407 0.19309
403 -> 409 -0.27647
404 -> 408 0.31021
405 -> 407 0.24418
405 -> 409 0.15714

Excited State 5: Singlet-A 4.2743 eV 290.07 nm f=0.4636 <S**2>=0.000
402 -> 420 0.20906
404 -> 420 -0.19457
405 -> 406 -0.11195
405 -> 410 -0.14733
405 -> 412 -0.10551
405 -> 414 -0.11506
405 -> 417 -0.13523
405 -> 420 0.30077
405 -> 421 0.15420
405 -> 422 0.21670

Excited State 6: Singlet-A 4.2792 eV 289.74 nm f=0.4763 <S**2>=0.000

402 -> 411	0.12590
402 -> 414	0.13525
402 -> 415	-0.14352
402 -> 416	0.10224
402 -> 419	-0.11320
402 -> 422	-0.19851
403 -> 419	-0.12528
404 -> 410	-0.11045
404 -> 416	0.10794
404 -> 419	-0.16797
404 -> 421	0.28836
405 -> 420	-0.15432

Excited State 7: Singlet-A 4.2806 eV 289.64 nm f=0.5631 <S**2>=0.000

402 -> 418	0.13838
402 -> 419	-0.19337
403 -> 412	0.11821
403 -> 414	0.13738
403 -> 416	0.11627
403 -> 418	-0.17604
403 -> 421	0.28645
403 -> 422	-0.16015
404 -> 415	-0.10302
404 -> 419	-0.15660
405 -> 420	0.11955

Excited State 8: Singlet-A 4.3013 eV 288.25 nm f=0.0597 <S**2>=0.000

402 -> 421	0.26287
403 -> 418	0.14890
403 -> 419	-0.20739

403 -> 420 -0.10157

404 -> 414 0.12684

404 -> 415 -0.12436

404 -> 419 -0.10142

404 -> 422 -0.18588

405 -> 420 0.21861

Excited State 9: Singlet-A 4.3154 eV 287.31 nm f=0.1076 <S**2>=0.000

402 -> 415 -0.15260

402 -> 417 0.12838

404 -> 416 0.16517

405 -> 410 -0.14374

405 -> 412 -0.15564

405 -> 413 -0.14920

405 -> 415 -0.22449

405 -> 416 -0.22871

405 -> 417 0.27807

405 -> 418 0.11542

405 -> 427 0.10502

Excited State 10: Singlet-A 4.3158 eV 287.28 nm f=0.0434 <S**2>=0.000

402 -> 411 -0.14869

402 -> 416 0.27709

403 -> 416 0.14831

404 -> 411 -0.14871

404 -> 413 -0.14445

404 -> 415 -0.18291

404 -> 416 0.25247

404 -> 423 0.10682

6.9.3 Computed emission energies of compound 5j (cam-B3LYP/def2svp SMD CH_2Cl_2 GD3BJ)

Excitation energies and oscillator strengths:

Excited State 1: Singlet-A 2.7252 eV 454.95 nm f=0.3168 <S**2>=0.000

405 -> 406 0.64456

405 -> 407 -0.11912

405 -> 410 0.11436

405 -> 433 0.11238

This state for optimization and/or second-order correction.

Total Energy, E(TD-HF/TD-DFT) = -6106.85624704

Copying the excited state density for this state as the 1-particle RhoCI density.

Excited State 2: Singlet-A 3.5875 eV 345.60 nm f=0.4864 <S**2>=0.000

402 -> 407 0.19868

402 -> 408 0.38834

402 -> 430 0.11095

403 -> 407 0.10782

403 -> 408 0.10143

404 -> 407 0.24569

404 -> 408 -0.18292

404 -> 409 0.28293

Excited State 3: Singlet-A 3.5898 eV 345.38 nm f=0.4567 <S**2>=0.000

402 -> 408 0.14888

402 -> 409 0.23888

403 -> 407 0.34384

403 -> 409 -0.31642

404 -> 407 -0.13045

404 -> 408 0.25545

404 -> 409 -0.11074

Excited State 4: Singlet-A 3.6185 eV 342.64 nm f=0.2390 <S**2>=0.000

402 -> 407 0.29095

402 -> 408 0.12860

403 -> 407 -0.18146

403 -> 408 0.16449

403 -> 409 0.30416

404 -> 408 0.26973

404 -> 409 -0.20842

Excited State 5: Singlet-A 3.7986 eV 326.40 nm f=0.3616 <S**2>=0.000

401 -> 406 -0.11888

405 -> 406 -0.16192

405 -> 410 0.30139

405 -> 411 0.10172

405 -> 414 0.22391

405 -> 415 0.36554

405 -> 417 -0.12752

405 -> 422 -0.16570

405 -> 428 -0.19607

Excited State 6: Singlet-A 3.9347 eV 315.10 nm f=0.0652 <S**2>=0.000

405 -> 410 -0.36672

405 -> 411 -0.21794

405 -> 413 0.13405

405 -> 414 0.13370

405 -> 415 0.20322

405 -> 417 -0.10896

405 -> 421 -0.29582

405 -> 422 0.12963

405 -> 428 -0.11468

405 -> 429 0.20073

corrected external emission excitation: -6106.924155 - -6107.022050 = 0.097895 = 2.663862 eV 465.43 nm

6.10 Quantum chemical calculation data of tetrakis(4-(10-hexyl-7-(p-tolyl)-10*H*-phenothiazin-3-yl)phenyl)silane (5k)

6.10.1 Computed xyz-coordinates of compound 5k (cam-B3LYP/def2svp SMD CH_2Cl_2 GD3BJ)

0 1

C -8.40192 -9.54789 -3.49057

C -7.72784 -10.08928 -4.58732

C -6.67365 -9.41964 -5.19867

C -6.25103 -8.15651 -4.76018

C -6.95622 -7.59591 -3.67826

C -7.98465 -8.28651 -3.04916

S -6.65371 -5.92267 -3.18934

N -5.18599 -7.47653 -5.37466

C -4.93023 -5.82605 -3.57735

C -4.40913 -6.54192 -4.67281

C -3.08193 -6.26384 -5.03151

C -2.31430 -5.34204 -4.32892

C -2.81907 -4.66701 -3.21550

C -4.14495 -4.93623 -2.85552

C -4.69969 -7.96132 -6.66434

C -3.69282 -9.10761 -6.61922

C -9.51129 -10.26932 -2.81948

C -9.73591 -10.13781 -1.44423

C -10.77698 -10.81693 -0.81716

C -11.63380 -11.65422 -1.53522

C -11.40851 -11.78691 -2.91054

C -10.37211 -11.10983 -3.54058

C -12.76372 -12.38695 -0.86655

C -1.99176 -3.70861 -2.44314

C -1.00793 -2.93200 -3.07151

C -0.22807 -2.04124 -2.34341

C -0.39140 -1.88629 -0.95810

C -1.38239 -2.65819 -0.33816

C -2.16589 -3.55125 -1.06218

Si 0.71910 -0.71657 0.01269

C 13.70173 -2.61688 1.28239

C 13.72249 -3.99335 1.04732

C 12.56759 -4.76399 1.12418

C 11.32884 -4.20239 1.46669

C 11.31705 -2.82113 1.73905

C 12.46749 -2.05077 1.62336

S 9.86563 -2.05288 2.39702

N 10.16052 -4.97839 1.54722

C 8.63414 -3.05770 1.62101

C 8.88849 -4.41660 1.35322

C 7.80409 -5.18086 0.89703

C 6.54610 -4.62161 0.70500

C 6.30579 -3.26544 0.93570

C 7.38322 -2.49754 1.39277

C 10.28961 -6.43362 1.53567

C 10.41058 -7.08685 0.16184

C 14.92773 -1.78712 1.18273

C 14.87217 -0.45617 0.75305

C 16.02463 0.31970 0.66311

C 17.27743 -0.20090 0.99540

C 17.33332 -1.53206 1.42500

C 16.18566 -2.30906 1.51806

C 18.52794 0.62753 0.89342

C 4.96860 -2.66237 0.71635

C 4.11916 -3.13795 -0.29092

C 2.86216 -2.57742 -0.49229

C 2.39943 -1.51704 0.29780

C 3.25391 -1.04602 1.30639

C 4.51072 -1.60317 1.51249

C -1.54775 9.68035 -7.76054

C -0.59421 10.54366 -7.21686

C 0.44535 10.07420 -6.42159

C 0.60273 8.70804 -6.14600

C -0.33752 7.83562 -6.72668

C -1.39559 8.31547 -7.48866

S -0.10511 6.08406 -6.63666

N 1.65142 8.23053 -5.34218

C 0.69725 5.98655 -5.06302

C 1.54575 7.02478 -4.63171

C 2.28847 6.78605 -3.46565

C 2.18311 5.58977 -2.76518

C 1.31123 4.57819 -3.17300

C 0.56613 4.81077 -4.33517

C 2.74279 9.14220 -5.00746

C 2.50013 10.07265 -3.82246

C -2.67011 10.17774 -8.59370

C -3.92036 9.54202 -8.58247

C -4.96987 10.00857 -9.36371

C -4.81973 11.12946 -10.18889

C -3.57483 11.76278 -10.19995

C -2.51971 11.29739 -9.41990

C -5.96458 11.62499 -11.02818

C 1.17763 3.30918 -2.41732

C 2.27151 2.74866 -1.74514

C 2.14422 1.55649 -1.03962

C 0.92302 0.87298 -0.97419

C -0.16796 1.43708 -1.65266

C -0.04684 2.62922 -2.35707

C -6.01820 4.77433 9.91225

C -5.36336 4.14137 10.97104

C -4.59467 2.99929 10.77494

C -4.45913 2.41199 9.50882

C -5.15374 3.02767 8.45019

C -5.88919 4.18988 8.64648

S -5.23894 2.24382 6.86627

N -3.68031 1.26065 9.30507

C -3.65508 1.45559 6.85933

C -3.09414 0.98108 8.06095

C -1.93030 0.20642 7.94873

C -1.35162 -0.06637 6.71450

C -1.88721 0.43878 5.52772

C -3.05032 1.21104 5.63304

C -3.23792 0.50205 10.47265

C -1.97554 1.00501 11.16765

C -6.81682 6.00891 10.11096

C -6.90629 6.98395 9.11078

C -7.65581 8.14186 9.29960

C -8.34385 8.37423 10.49265

C -8.25400 7.39945 11.49350

C -7.50890 6.24185 11.30851

C -9.15169 9.62410 10.70658

C -1.25950 0.17193 4.21070

C -0.57629 -1.02757 3.96497

C 0.01684 -1.27223 2.73215

C -0.05176 -0.33786 1.68732

C -0.73473 0.85889 1.93931

C -1.32550 1.11121 3.17342

H -8.00316 -11.07590 -4.96355

H -6.17246 -9.90945 -6.02928

H -8.49961 -7.80373 -2.21641

H -2.61952 -6.77773 -5.86996

H -1.28279 -5.17809 -4.64489

H -4.59882 -4.40890 -2.01440

H -5.57773 -8.25940 -7.25354

H -4.26343 -7.10270 -7.19282

H -3.40575 -9.38075 -7.64604

H -2.77791 -8.83639 -6.07520

H -4.10523 -10.00349 -6.13577

H -9.07457 -9.50904 -0.84460

H -10.92144 -10.69797 0.25976

H -12.06600 -12.42971 -3.50233

H -10.24035 -11.22178 -4.61867

H -12.76701 -12.21672 0.21912

H -13.73837 -12.05987 -1.26295

H -12.69452 -13.47208 -1.04162

H -0.86401 -3.00963 -4.15086

H 0.51877 -1.44529 -2.87515

H -1.55012 -2.56911 0.73779

H -2.91358 -4.14993 -0.53826

H 14.65490 -4.48478 0.76418

H 12.64827 -5.82484 0.90212

H 12.40122 -0.98480 1.84945

H 7.92710 -6.24044 0.68897

H 5.73433 -5.27099 0.37310

H 7.25027 -1.43098 1.58361

H 11.16478 -6.68914 2.14851

H 9.42210 -6.84459 2.06990

H 10.50210 -8.17698 0.28266

H 9.53378 -6.89068 -0.46992

H 11.29348 -6.73387 -0.38800

H 13.91393 -0.01919 0.46489

H 15.94770 1.35424 0.31873

H 18.29804 -1.96636 1.70134

H 16.26584 -3.33635 1.87894

H 18.30004 1.66810 0.62356

H 19.08029 0.63486 1.84606

H 19.21138 0.22243 0.12981

H 4.45152 -3.95059 -0.93974

H 2.23216 -2.98000 -1.28895

H 2.92986 -0.23142 1.95982

H 5.13700 -1.22349 2.32213

H -0.67272 11.61899 -7.38554

H 1.13771 10.80242 -6.00741

H -2.09517 7.59448 -7.91592

H 2.96341 7.54481 -3.07833

H 2.77724 5.46547 -1.85833

H -0.10576 4.03842 -4.71433

H 3.63638 8.53013 -4.82489

H 2.96469 9.73223 -5.90694

H 3.38651 10.70664 -3.66840

H 1.63763 10.73337 -3.98414

H 2.31672 9.51897 -2.89167

H -4.08137 8.67719 -7.93564

H -5.93423 9.49445 -9.32734

H -3.42294 12.63601 -10.83962

H -1.55502 11.80592 -9.47230

H -6.82591 11.90389 -10.40056

H -5.67809 12.50518 -11.62050

H -6.31445 10.84602 -11.72402

H 3.24539 3.23967 -1.79297

H 3.02571 1.14766 -0.53982

H -1.14065 0.93806 -1.63246

H -0.92366 3.04674 -2.85587

H -5.42006 4.56091 11.97682

H -4.08739 2.57261 11.63622

H -6.40846 4.62507 7.79046

H -1.44790 -0.19216 8.83727

H -0.43793 -0.66246 6.69123

H -3.52841 1.60129 4.73261

H -4.07366 0.48407 11.18543

H -3.09681 -0.53916 10.15209

H -1.75460 0.36033 12.03192

H -1.10042 0.98812 10.50404

H -2.08556 2.03421 11.53524

H -6.36566 6.84810 8.17196

H -7.70024 8.88654 8.50072

H -8.78775 7.54805 12.43622

H -7.47976 5.49425 12.10373

H -9.15693 10.25823 9.80908

H -10.19627 9.38456 10.96052

H -8.74784 10.22056 11.54029

H -0.52068 -1.78917 4.74508

H 0.54317 -2.21916 2.58397

H -0.80216 1.62288 1.16104

H -1.82795 2.06636 3.33831

SCF Done: E(RCAM-B3LYP) = -6264.10443204 A.U. after 1 cycles

Zero-point correction= 1.678257 (Hartree/Particle)

Thermal correction to Energy= 1.779226

Thermal correction to Enthalpy= 1.780170

Thermal correction to Gibbs Free Energy= 1.514457

Sum of electronic and zero-point Energies= -6262.426175

Sum of electronic and thermal Energies= -6262.325206

Sum of electronic and thermal Enthalpies= -6262.324262

Sum of electronic and thermal Free Energies= -6262.589975

6.10.2 Computed excitation energies of compound 5k (cam-B3LYP/def2svp SMD CH_2Cl_2 GD3BJ)

Excitation energies and oscillator strengths:

Excited State 1: Singlet-A 3.5678 eV 347.51 nm f=0.4457 <S**2>=0.000

418 -> 423 -0.10786

418 -> 424 -0.13753

418 -> 425 0.11659

419 -> 422 0.11277

419 -> 423 0.16055

420 -> 423 0.13822

420 -> 425 -0.16088

421 -> 422 0.26574

421 -> 423 0.28860

421 -> 424 0.22864

421 -> 425 -0.22330

This state for optimization and/or second-order correction.

Total Energy, E(TD-HF/TD-DFT) = -6263.97331733

Copying the excited state density for this state as the 1-particle RhoCI density.

Excited State 2: Singlet-A 3.5751 eV 346.80 nm f=0.5255 <S**2>=0.000

418 -> 423 -0.29137

418 -> 424 0.11153

418 -> 425 -0.21861

419 -> 422 -0.30196

419 -> 424 0.31649

419 -> 426 0.10897

419 -> 448 0.10471

420 -> 425 -0.18959

421 -> 422 0.10014

Excited State 3: Singlet-A 3.5793 eV 346.39 nm f=0.9037 <S**2>=0.000

418 -> 424 -0.23109

418 -> 425 -0.19928
419 -> 423 0.14531
419 -> 425 0.19817
420 -> 422 0.27618
420 -> 423 -0.23705
420 -> 424 0.16136
420 -> 425 0.14466
420 -> 426 -0.10522
420 -> 446 0.10723
421 -> 422 -0.11058
421 -> 424 -0.15551
421 -> 425 -0.14009

Excited State 4: Singlet-A 3.6081 eV 343.63 nm f=0.0281 <S**2>=0.000
418 -> 422 0.29034
418 -> 426 -0.12284
419 -> 423 0.18458
419 -> 424 -0.15855
419 -> 425 0.19463
420 -> 424 -0.26613
420 -> 425 -0.19246
421 -> 423 -0.21381
421 -> 425 0.17546

Excited State 5: Singlet-A 4.2460 eV 292.00 nm f=0.6109 <S**2>=0.000
418 -> 431 -0.15006
420 -> 427 0.14587
420 -> 429 -0.10533
420 -> 430 -0.13991
420 -> 431 0.34622
420 -> 434 0.14430

420 -> 435 -0.10309

421 -> 427 -0.10296

421 -> 430 0.11342

421 -> 431 -0.19181

Excited State 6: Singlet-A 4.2718 eV 290.24 nm f=0.3723 <S**2>=0.000

418 -> 435 -0.15515

418 -> 436 0.24467

419 -> 422 0.10721

419 -> 426 0.13831

419 -> 427 -0.12481

419 -> 430 0.10118

419 -> 433 -0.10660

419 -> 434 0.21275

419 -> 435 -0.12011

419 -> 437 0.18710

419 -> 438 0.20844

420 -> 436 0.10108

421 -> 437 0.13741

Excited State 7: Singlet-A 4.2730 eV 290.15 nm f=0.4498 <S**2>=0.000

418 -> 436 -0.15680

418 -> 438 0.15132

419 -> 436 0.13529

420 -> 436 -0.14662

420 -> 437 0.18136

421 -> 437 0.33342

421 -> 438 -0.11483

Excited State 8: Singlet-A 4.2932 eV 288.79 nm f=0.1850 <S**2>=0.000

418 -> 422 -0.10150

418 -> 426 -0.10848

418 -> 434 -0.14506

418 -> 437 -0.18454

418 -> 438 -0.12738

419 -> 434 -0.10057

419 -> 435 0.16365

419 -> 436 -0.23807

420 -> 430 -0.11035

420 -> 438 -0.15432

421 -> 437 0.22459

Excited State 9: Singlet-A 4.3119 eV 287.54 nm f=0.0913 <S**2>=0.000

418 -> 427 0.10780

418 -> 431 -0.12357

418 -> 432 0.13158

420 -> 427 -0.16355

420 -> 429 0.12171

420 -> 431 0.27831

420 -> 432 -0.18889

420 -> 434 -0.18005

420 -> 435 0.10814

420 -> 436 0.12592

421 -> 427 0.10306

421 -> 431 -0.16924

421 -> 432 0.10883

421 -> 434 0.12006

Excited State 10: Singlet-A 4.3127 eV 287.49 nm f=0.0959 <S**2>=0.000

418 -> 429 -0.12696

418 -> 432 -0.15980

418 -> 433 0.17040

418 -> 436	-0.13766
419 -> 426	0.12876
419 -> 427	-0.16552
419 -> 429	-0.11416
419 -> 432	-0.20623
419 -> 433	0.35164
419 -> 436	-0.11623
419 -> 443	0.13775
420 -> 432	-0.10748

6.10.3 Computed emission energies of compound 5k (cam-B3LYP/def2svp SMD CH_2Cl_2 GD3BJ)

Excitation energies and oscillator strengths:

Excited State 1: Singlet-A 2.7132 eV 456.96 nm f=0.3385 <S**2>=0.000

421 -> 422 0.63910

421 -> 423 0.12268

421 -> 426 0.13554

421 -> 449 0.11192

This state for optimization and/or second-order correction.

Total Energy, E(TD-HF/TD-DFT) = -6263.92254064

Copying the excited state density for this state as the 1-particle RhoCI density.

Excited State 2: Singlet-A 3.5899 eV 345.37 nm f=0.3391 <S**2>=0.000

418 -> 423 -0.15705

418 -> 424 -0.16544

418 -> 425 0.25504

419 -> 423 0.28948

419 -> 425 -0.18595

420 -> 423 0.23525

420 -> 424 -0.30034

420 -> 425 0.10174

Excited State 3: Singlet-A 3.6031 eV 344.11 nm f=0.6696 <S**2>=0.000

418 -> 423 -0.15203

418 -> 424 0.17500

418 -> 425 0.25901

419 -> 423 0.23929

419 -> 424 0.23688

420 -> 423 -0.20654

420 -> 424 0.23141

420 -> 425 -0.23132

Excited State   4:   Singlet-A   3.6334 eV 341.24 nm f=0.2825 <S**2>=0.000

418 -> 423   0.27601

418 -> 424   0.18507

418 -> 425   0.16823

418 -> 426   -0.10209

418 -> 446   0.10353

419 -> 424   0.14136

419 -> 425   0.35812

420 -> 424   -0.27204

Excited State   5:   Singlet-A   3.7678 eV 329.07 nm f=0.3260 <S**2>=0.000

417 -> 422   0.11351

421 -> 422   0.16997

421 -> 426   -0.32198

421 -> 427   0.15574

421 -> 430   -0.18954

421 -> 431   -0.25097

421 -> 432   -0.16680

421 -> 433   0.15113

421 -> 435   0.11509

421 -> 437   0.19534

421 -> 438   0.10652

421 -> 444   0.19329

Excited State   6:   Singlet-A   3.9521 eV 313.72 nm f=0.0804 <S**2>=0.000

421 -> 426   0.30202

421 -> 427   -0.21018

421 -> 429   -0.13080

421 -> 430   -0.14909

421 -> 431   -0.10881

421 -> 432   -0.12188

421 -> 437 0.34322

421 -> 438 -0.22398

421 -> 445 0.19224

corrected external emission excitation: -6263.992194 - -6264.089541 = 0.097347 = 2.648949 eV 468.05 nm

6.11 Quantum chemical calculation data of tetrakis(4-(10-hexyl-7-(4-methoxyphenyl)-10*H*-phenothiazin-3-yl)phenyl)silane (5l)

6.11.1 Computed xyz-coordinates of compound 5l (cam-B3LYP/def2svp SMD CH_2Cl_2 GD3BJ)

0 1

C -8.32965 -9.57573 -3.60365

C -7.64833 -10.11372 -4.69787

C -6.58803 -9.44410 -5.29884

C -6.16383 -8.18493 -4.85100

C -6.87359 -7.62868 -3.77026

C -7.90979 -8.31846 -3.15265

S -6.56646 -5.96038 -3.26740

N -5.09239 -7.50460 -5.45556

C -4.84056 -5.86789 -3.64554

C -4.31621 -6.57763 -4.74360

C -2.98647 -6.30056 -5.09396

C -2.21951 -5.38624 -4.38087

C -2.72753 -4.71826 -3.26469

C -4.05600 -4.98607 -2.91323

C -4.60084 -7.98323 -6.74536

C -3.59768 -9.13291 -6.70188

C -9.44871 -10.29403 -2.94664

C -9.68610 -10.16780 -1.56763

C -10.73021 -10.83661 -0.95095

C -11.58206 -11.66283 -1.69539

C -11.36344 -11.80322 -3.06825

C -10.30864 -11.12223 -3.67356

C -1.90120 -3.76844 -2.48082

C -0.91291 -2.98872 -3.09826

C -0.13436 -2.10588 -2.35916

C -0.30348 -1.96243 -0.97329

C -1.29872 -2.73750 -0.36425

C -2.08098 -3.62257 -1.09933

Si 0.80505 -0.80307 0.01196

C -5.93952 4.61185 9.94831

C -5.28740 3.96599 11.00131

C -4.52496 2.82129 10.79575

C -4.39055 2.24485 9.52469

C -5.08003 2.87428 8.47127

C -5.81127 4.03768 8.67757

S -5.16385 2.10743 6.87887

N -3.61696 1.09092 9.31106

C -3.58320 1.31271 6.86807

C -3.02816 0.82227 8.06619

C -1.86782 0.04302 7.94866

C -1.28661 -0.21858 6.71320

C -1.81590 0.30257 5.53048

C -2.97572 1.07910 5.64101

C -3.18246 0.31786 10.47193

C -1.91920 0.80598 11.17597

C -6.73396 5.84684 10.15726

C -6.82524 6.83163 9.15930

C -7.56434 7.98700 9.35053

C -8.24447 8.20286 10.55602

C -8.16701 7.23648 11.56215

C -7.42003 6.07853 11.35285

C -1.18503 0.04771 4.21260

C -0.50362 -1.15070 3.95662

C 0.09289 -1.38399 2.72325

C 0.02933 -0.43903 1.68766

C -0.65194 0.75651 1.94978

C -1.24582 0.99757 3.18465

C -1.44521 9.68157 -7.64749

C -0.48913 10.53633 -7.09433

C 0.55137 10.05560 -6.30674

C 0.70676 8.68630 -6.04683

C -0.23632 7.82288 -6.63565

C -1.29427 8.31337 -7.39124

S -0.00828 6.06982 -6.56492

N 1.75616 8.19710 -5.24979

C 0.79627 5.95304 -4.99371

C 1.64830 6.98431 -4.55239

C 2.39225 6.73052 -3.39019

C 2.28469 5.52688 -2.70281

C 1.40932 4.52224 -3.12022

C 0.66308 4.76969 -4.27862

C 2.84920 9.10299 -4.90568

C 2.60909 10.01986 -3.70960

C -2.56718 10.18901 -8.47414

C -3.82080 9.55476 -8.46938

C -4.87027 10.02444 -9.24130

C -4.70417 11.15439 -10.05256

C -3.46548 11.80055 -10.07222

C -2.41933 11.31427 -9.28999

C 1.27323 3.24559 -2.37800

C 2.36613 2.67558 -1.71223

C 2.23643 1.47669 -1.01863

C 1.01369 0.79539 -0.95941

C -0.07621 1.36875 -1.63190

C 0.04726 2.56775 -2.32413

C 13.78030 -2.75403 1.26777

C 13.79328 -4.13150 1.03644

C 12.63576 -4.89741 1.12164

C 11.40059 -4.32985 1.46646

C 11.39574 -2.94786 1.73355

C 12.54921 -2.18253 1.61190

S 9.94877 -2.17111 2.39167

N 10.22860 -5.10084 1.55329

C 8.71152 -3.17305 1.62092

C 8.95923 -4.53428 1.35823

C 7.87064 -5.29527 0.90632

C 6.61519 -4.73079 0.71306

C 6.38161 -3.37256 0.93849

C 7.46299 -2.60794 1.39173

C 10.35157 -6.55649 1.54767

C 10.46710 -7.21669 0.17662

C 15.00910 -1.93013 1.16122

C 14.95691 -0.59300 0.73298

C 16.10284 0.17806 0.63159

C 17.35329 -0.36360 0.95607

C 17.42804 -1.69127 1.38459

C 16.26498 -2.45291 1.48266

C 5.04718 -2.76411 0.71756

C 4.19664 -3.23704 -0.28998

C 2.94207 -2.67150 -0.49285

C 2.48297 -1.60884 0.29623

C 3.33852 -1.14063 1.30530

C 4.59304 -1.70242 1.51256

O -12.57262 -12.27448 -1.01400

C -13.46082 -13.11799 -1.70847

O -8.93925 9.35473 10.65667

C -9.63509 9.63141 11.84915

O -5.77823 11.54207 -10.77073

C -5.67215 12.67257 -11.60352

O 18.41665 0.45639 0.82781

C 19.69993 -0.03849 1.12980

H -7.92361 -11.09793 -5.08059

H -6.08284 -9.93132 -6.12859

H -8.42846 -7.83753 -2.32105

H -2.52172 -6.80950 -5.93415

H -1.18605 -5.22280 -4.69073

H -4.51235 -4.46348 -2.07048

H -5.47662 -8.27523 -7.34098

H -4.15913 -7.12298 -7.26666

H -3.30649 -9.40129 -7.72881

H -2.68455 -8.86758 -6.15199

H -4.01521 -10.03014 -6.22535

H -9.02642 -9.54595 -0.95906

H -10.90532 -10.74083 0.12230

H -12.01159 -12.43066 -3.67905

H -10.17078 -11.23071 -4.75124

H -0.76455 -3.05747 -4.17765

H 0.61615 -1.50696 -2.88239

H -1.47086 -2.65734 0.71170

H -2.83222 -4.22413 -0.58389

H -5.34160 4.37725 12.01069

H -4.02070 2.38442 11.65373

H -6.32727 4.48247 7.82450

H -1.39025 -0.36810 8.83409

H -0.37575 -0.81882 6.68614

H -3.44925 1.48177 4.74364

H -4.02038 0.29784 11.18216

H -3.04657 -0.72098 10.14136

H -1.70550 0.15183 12.03495

H -1.04188 0.78943 10.51524

H -2.02374 1.83245 11.55267

H -6.28901 6.69947 8.21750

H -7.62496 8.75226 8.57438

H -8.69052 7.36857 12.50841

H -7.39186 5.32849 12.14576

H -0.45201 -1.92045 4.72895

H 0.61781 -2.33043 2.56713

H -0.71544 1.52853 1.17916

H -1.74663 1.95214 3.35764

H -0.56617 11.61390 -7.24925

H 1.24565 10.77792 -5.88539

H -1.99533 7.59817 -7.82585

H 3.06981 7.48316 -2.99555

H 2.87989 5.39097 -1.79832

H -0.01158 4.00344 -4.66524

H 3.74219 8.48765 -4.73116

H 3.07102 9.70330 -5.79837

H 3.49671 10.65034 -3.54838

H 1.74768 10.68398 -3.86299

H 2.42508 9.45541 -2.78541

H -3.98439 8.68371 -7.83172

H -5.84451 9.53214 -9.22748

H -3.29888 12.67620 -10.69858

H -1.45441 11.82280 -9.33873

H 3.34113 3.16479 -1.75552

H 3.11722 1.06096 -0.52335

H -1.05007 0.87181 -1.61641

H -0.82893 2.99237 -2.81802

H 14.72199 -4.62787 0.74964

H 12.71139 -5.95937 0.90297

H 12.48722 -1.11564 1.83506

H 7.98852 -6.35625 0.70248

H 5.79990 -5.37747 0.38446

H 7.33504 -1.54006 1.57867

H 11.22701 -6.81298 2.15978

H 9.48347 -6.96167 2.08543

H 10.55485 -8.30658 0.30244

H 9.58963 -7.02040 -0.45417

H 11.35004 -6.86963 -0.37690

H 13.99857 -0.15054 0.45385

H 16.05686 1.21394 0.29018

H 18.38337 -2.14079 1.65314

H 16.34658 -3.48108 1.84063

H 4.52630 -4.05157 -0.93783

H 2.31109 -3.07198 -1.28977

H 3.01706 -0.32458 1.95824

H 5.22043 -1.32469 2.32226

H -14.17374 -13.50268 -0.96800

H -14.01741 -12.57175 -2.48856

H -12.93645 -13.96957 -2.17388

H -10.11616 10.60849 11.71355

H -10.41350 8.87686 12.05275

H -8.95498 9.68629 12.71587

H -6.65060 12.80334 -12.08315

H -5.43309 13.58315 -11.02861

H -4.90769 12.53328 -12.38649

H 20.40442 0.78452 0.95420

H 19.77818 -0.35556 2.18339

H 19.97385 -0.88697 0.48020

SCF Done: E(RCAM-B3LYP) = -6564.62604360 A.U. after 1 cycles

Zero-point correction= 1.700147 (Hartree/Particle)

Thermal correction to Energy= 1.803861

Thermal correction to Enthalpy= 1.804805

Thermal correction to Gibbs Free Energy= 1.534984

Sum of electronic and zero-point Energies= -6562.925897

Sum of electronic and thermal Energies= -6562.822183

Sum of electronic and thermal Enthalpies= -6562.821238

Sum of electronic and thermal Free Energies= -6563.091060

6.11.2 Computed excitation energies of compound 5l (cam-B3LYP/def2svp SMD CH_2Cl_2 GD3BJ)

Excitation energies and oscillator strengths:

Excited State 1: Singlet-A 3.5736 eV 346.95 nm f=0.4567 <S**2>=0.000

434 -> 440 0.13493

434 -> 441 0.11728

435 -> 441 -0.12070

436 -> 438 0.11622

436 -> 439 0.14688

437 -> 438 0.26853

437 -> 439 0.30591

437 -> 440 -0.20991

437 -> 441 -0.24999

437 -> 442 -0.10586

437 -> 444 -0.11397

This state for optimization and/or second-order correction.

Total Energy, E(TD-HF/TD-DFT) = -6564.49471769

Copying the excited state density for this state as the 1-particle RhoCI density.

Excited State 2: Singlet-A 3.5799 eV 346.33 nm f=0.5560 <S**2>=0.000

434 -> 439 0.28948

434 -> 441 0.20637

435 -> 438 -0.25897

435 -> 440 -0.27128

435 -> 442 0.11881

436 -> 438 0.14231

436 -> 440 0.15030

436 -> 441 0.19299

437 -> 438 -0.10723

Excited State 3: Singlet-A 3.5856 eV 345.79 nm f=0.9440 <S**2>=0.000

434 -> 440 0.23604

434 -> 441 -0.20748

435 -> 438 0.15103

435 -> 439 -0.25286

435 -> 440 -0.13326

436 -> 438 0.23945

436 -> 439 -0.10092

436 -> 440 -0.11705

436 -> 441 0.24583

436 -> 442 -0.11036

437 -> 440 0.12655

Excited State 4: Singlet-A 3.6147 eV 343.00 nm f=0.0209 <S**2>=0.000

434 -> 438 0.28270

434 -> 439 -0.10631

434 -> 442 -0.14600

435 -> 439 -0.15138

435 -> 441 -0.27670

436 -> 440 0.30747

436 -> 443 0.11979

437 -> 439 -0.20929

437 -> 441 0.14679

Excited State 5: Singlet-A 4.2391 eV 292.48 nm f=0.6385 <S**2>=0.000

434 -> 443 -0.11939

434 -> 451 -0.12670

435 -> 443 0.11100

435 -> 451 0.16546

436 -> 443 0.16767

436 -> 445 -0.12290

436 -> 446 -0.12467

436 -> 447 0.14398

436 -> 451 0.28975

Excited State 6: Singlet-A 4.2629 eV 290.84 nm f=0.3875 <S**2>=0.000

434 -> 445 -0.11243

434 -> 452 0.24811

435 -> 442 -0.13372

435 -> 443 0.10720

435 -> 451 -0.13007

435 -> 452 -0.13178

435 -> 453 -0.13614

435 -> 454 -0.18309

436 -> 452 0.24457

437 -> 453 0.13247

Excited State 7: Singlet-A 4.2643 eV 290.75 nm f=0.4760 <S**2>=0.000

434 -> 444 -0.11260

434 -> 451 0.12891

434 -> 452 -0.16209

434 -> 454 0.14958

435 -> 451 0.11313

435 -> 452 -0.13042

435 -> 453 0.14594

435 -> 454 0.10413

437 -> 444 0.10874

437 -> 445 0.11760

437 -> 446 -0.10216

437 -> 450 -0.12624

437 -> 453 0.32283

437 -> 454 -0.12230

Excited State 8: Singlet-A 4.2852 eV 289.33 nm f=0.1842 <S**2>=0.000

434 -> 438 -0.10311

434 -> 442 -0.12404

434 -> 451 -0.15694

434 -> 453 -0.20864

434 -> 454 -0.11218

435 -> 445 -0.11314

435 -> 452 0.26818

436 -> 443 0.11018

436 -> 452 -0.10595

436 -> 454 -0.15813

437 -> 444 0.10182

437 -> 451 -0.11020

437 -> 453 0.18270

437 -> 454 -0.12078

Excited State 9: Singlet-A 4.3066 eV 287.90 nm f=0.0874 <S**2>=0.000

434 -> 447 -0.19619

434 -> 448 -0.12341

435 -> 447 0.18763

435 -> 448 0.12669

436 -> 443 -0.12793

436 -> 445 0.10820

436 -> 447 0.38946

436 -> 448 0.12898

436 -> 455 -0.14679

437 -> 447 -0.15738

Excited State 10: Singlet-A 4.3073 eV 287.85 nm f=0.0362 <S**2>=0.000

434 -> 448 0.11188

434 -> 450 -0.13806

435 -> 448 -0.14554

435 -> 449	-0.14332
436 -> 448	0.17883
436 -> 449	0.14629
437 -> 444	0.14617
437 -> 445	0.11069
437 -> 446	-0.10843
437 -> 450	0.35226
437 -> 457	0.13529

6.11.3 Computed emission energies of compound 5l (cam-B3LYP/def2svp SMD CH_2Cl_2 GD3BJ)

Excitation energies and oscillator strengths:

Excited State 1: Singlet-A 2.7207 eV 455.71 nm f=0.3560 <S**2>=0.000

437 -> 438 0.63155

437 -> 439 0.11465

437 -> 442 -0.11062

437 -> 443 -0.14905

437 -> 465 0.10656

This state for optimization and/or second-order correction.

Total Energy, E(TD-HF/TD-DFT) = -6564.45026753

Copying the excited state density for this state as the 1-particle RhoCI density.

Excited State 2: Singlet-A 3.5957 eV 344.81 nm f=0.4548 <S**2>=0.000

434 -> 440 -0.14322

434 -> 441 0.23759

435 -> 440 0.12893

435 -> 441 -0.27320

436 -> 439 0.34797

436 -> 440 0.26443

436 -> 442 0.14863

436 -> 463 -0.10077

Excited State 3: Singlet-A 3.6034 eV 344.07 nm f=0.7937 <S**2>=0.000

434 -> 440 -0.28902

434 -> 441 -0.24978

434 -> 444 0.10581

435 -> 439 0.33225

435 -> 440 -0.14699

435 -> 442 0.13945

436 -> 440 0.16738

436 -> 441 -0.24677

Excited State 4: Singlet-A 3.6316 eV 341.40 nm f=0.0965 <S**2>=0.000

434 -> 439 -0.31083

434 -> 440 0.15130

434 -> 442 -0.14595

435 -> 440 0.25955

435 -> 441 0.24344

435 -> 444 -0.10237

436 -> 440 0.19629

436 -> 441 -0.22408

Excited State 5: Singlet-A 3.7670 eV 329.13 nm f=0.3242 <S**2>=0.000

437 -> 438 0.16190

437 -> 442 0.12052

437 -> 443 0.37949

437 -> 444 -0.12715

437 -> 445 -0.12986

437 -> 446 -0.19570

437 -> 447 -0.10831

437 -> 450 -0.15550

437 -> 451 -0.16682

437 -> 453 0.19050

437 -> 460 0.21820

Excited State 6: Singlet-A 3.9956 eV 310.30 nm f=0.0690 <S**2>=0.000

437 -> 442 -0.14383

437 -> 443 -0.24987

437 -> 450 -0.11620

437 -> 451 -0.11654

437 -> 453 0.40101

437 -> 454 0.25182

437 -> 460 0.13253

437 -> 461 -0.22768

corrected external emission excitation: -6564.514079 - -6564.611415 = 0.097336 = 2.648638 eV 468.11 nm

6.12 Quantum chemical calculation data of tetrakis(4-(10-hexyl-7-(5-methylthiophen-2-yl)-10*H*-phenothiazin-3-yl)phenyl)silane (5m)

6.12.1 Computed xyz-coordinates of compound 5m (cam-B3LYP/def2svp SMD CH_2Cl_2 GD3BJ)

0 1

C -8.99836 -9.13542 -3.32307

C -8.35721 -9.70852 -4.42373

C -7.28009 -9.08590 -5.04336

C -6.79556 -7.84506 -4.60571

C -7.46642 -7.25334 -3.51698

C -8.52307 -7.89252 -2.88341

S -7.08326 -5.59657 -3.02897

N -5.70644 -7.21404 -5.22801

C -5.36103 -5.57623 -3.43387

C -4.88240 -6.31483 -4.53335

C -3.54716 -6.09738 -4.90367

C -2.73290 -5.21100 -4.20838

C -3.19707 -4.51251 -3.09167

C -4.53058 -4.72154 -2.72002

C -5.25012 -7.72465 -6.51869

C -4.29713 -8.91574 -6.47186

C -10.12284 -9.78894 -2.64106

C -2.31842 -3.59348 -2.32795

C -1.29947 -2.87167 -2.96545

C -0.46638 -2.02353 -2.24533

C -0.61080 -1.85699 -0.85950

C -1.63886 -2.57124 -0.23058

C -2.47454 -3.42258 -0.94627

Si 0.58227 -0.76683 0.10544

C -10.60027 -9.56851 -1.37468

C -11.72022 -10.38616 -1.04237

C -12.09954 -11.22902 -2.05027

S -11.07315 -11.01519 -3.43287

C -13.21340 -12.22475 -2.06432

C -0.58078 9.66197 -7.86026

C 0.42453 10.45110 -7.29682

C 1.40083 9.90560 -6.47149

C 1.43606 8.53483 -6.17787

C 0.44226 7.73589 -6.77792

C -0.54815 8.28987 -7.57704

S 0.52627 5.97273 -6.66366

N 2.41909 7.98138 -5.34176

C 1.27211 5.82907 -5.06602

C 2.19404 6.79699 -4.62234

C 2.88279 6.51101 -3.43406

C 2.65607 5.33779 -2.72365

C 1.71048 4.40014 -3.14360

C 1.01987 4.67901 -4.32885

C 3.57241 8.80451 -4.98497

C 3.37441 9.76364 -3.81462

C -1.63485 10.22376 -8.71475

C 1.44692 3.15947 -2.37552

C 2.47200 2.51278 -1.67325

C 2.22288 1.34845 -0.95414

C 0.94322 0.78002 -0.90416

C -0.07849 1.43001 -1.61306

C 0.16468 2.59471 -2.33198

C -2.85853 9.68976 -9.02760

C -3.62978 10.51491 -9.89804

C -2.99935 11.67596 -10.25096

S -1.43212 11.75945 -9.51119

C -3.49430 12.77533 -11.13339

C -5.73060 5.49078 9.83812

C -5.13304 4.83113 10.91590

C -4.46502 3.62509 10.74738

C -4.36867 3.00293 9.49314

C -5.00327 3.65165 8.41674

C -5.64489 4.87213 8.58462

S -5.14150 2.84815 6.84694

N -3.68350 1.78979 9.31947

C -3.63098 1.92734 6.86946

C -3.11829 1.43385 8.08473

C -2.02400 0.56086 7.99696

C -1.46575 0.21146 6.77279

C -1.95143 0.73274 5.57165

C -3.04420 1.60419 5.65260

C -3.30087 1.02846 10.50654

C -2.00521 1.45439 11.19128

C -6.42399 6.77236 10.02257

C -1.34364 0.38173 4.26506

C -0.76561 -0.87772 4.05175

C -0.18896 -1.20151 2.82938

C -0.16977 -0.28910 1.76327

C -0.74879 0.96744 1.98270

C -1.32287 1.29846 3.20599

C -6.94850 7.30231 11.17339

C -7.54514 8.58361 10.98499

C -7.47763 9.03337 9.69523

S -6.66435 7.87290 8.69426

C -7.99251 10.31536 9.12638

C 13.33099 -3.76220 1.44420

C 13.23586 -5.13970 1.22694

C 12.02254 -5.80770 1.33022

C 10.83697 -5.13804 1.67022

C 10.94282 -3.75674 1.92123

C 12.15300 -3.08774 1.78877
S 9.56427 -2.86006 2.57227
N 9.60810 -5.81064 1.76484
C 8.25109 -3.76595 1.80828
C 8.38817 -5.14527 1.56160
C 7.24306 -5.82085 1.11455
C 6.03860 -5.15821 0.90917
C 5.91549 -3.78273 1.11742
C 7.05372 -3.10339 1.56745
C 9.61140 -7.27193 1.77376
C 9.67410 -7.95324 0.40955
C 14.61659 -3.06227 1.32349
C 4.63640 -3.06983 0.88059
C 3.75700 -3.48188 -0.12905
C 2.55570 -2.81597 -0.34873
C 2.18077 -1.70973 0.42486
C 3.06387 -1.30367 1.43688
C 4.26549 -1.96582 1.66095
C 15.88320 -3.57942 1.41613
C 16.90678 -2.60339 1.23449
C 16.42611 -1.34399 1.00390
S 14.69120 -1.35236 1.00147
C 17.19103 -0.08106 0.77570
H -8.68084 -10.68005 -4.80289
H -6.81207 -9.59563 -5.88137
H -9.01058 -7.38870 -2.04693
H -3.11506 -6.63218 -5.74527
H -1.69773 -5.09447 -4.53321
H -4.95335 -4.17409 -1.87556
H -6.14388 -7.98428 -7.10222
H -4.77793 -6.88893 -7.05283

H -4.03101 -9.21046 -7.49832

H -3.36652 -8.68248 -5.93689

H -4.74715 -9.78711 -5.97739

H -1.16753 -2.95925 -4.04555

H 0.30957 -1.47240 -2.78349

H -1.79367 -2.47072 0.84637

H -3.24906 -3.97910 -0.41489

H -10.15227 -8.85022 -0.68726

H -12.23020 -10.35525 -0.07811

H -13.75672 -12.18862 -1.10995

H -13.93237 -12.02234 -2.87377

H -12.84095 -13.25177 -2.20531

H 0.44409 11.52776 -7.47812

H 2.14144 10.57958 -6.04943

H -1.29266 7.62548 -8.01912

H 3.61038 7.21363 -3.03659

H 3.21282 5.17433 -1.79939

H 0.29445 3.96173 -4.71763

H 4.40841 8.12414 -4.77398

H 3.86516 9.36519 -5.88301

H 4.30592 10.32287 -3.63897

H 2.57519 10.49234 -4.00655

H 3.11825 9.23576 -2.88606

H 3.48719 2.91277 -1.70772

H 3.05347 0.86911 -0.43044

H -1.09300 1.02238 -1.60574

H -0.65985 3.08300 -2.85522

H -3.21015 8.73621 -8.63243

H -4.63017 10.25786 -10.25015

H -4.49292 12.52357 -11.51635

H -3.56843 13.73155 -10.59164

H -2.82920 12.93762 -11.99619

H -5.16172 5.27702 11.91123

H -4.00274 3.17427 11.62155

H -6.12058 5.33002 7.71431

H -1.58103 0.14265 8.89686

H -0.60651 -0.46111 6.76857

H -3.48350 2.01319 4.74080

H -4.13648 1.08769 11.21714

H -3.23625 -0.02725 10.21060

H -1.82802 0.81200 12.06723

H -1.13527 1.36390 10.52682

H -2.04228 2.49542 11.53970

H -0.78014 -1.62275 4.84953

H 0.25467 -2.19346 2.70743

H -0.74464 1.71650 1.18722

H -1.74158 2.29718 3.34447

H -6.92491 6.78145 12.13110

H -8.01895 9.15668 11.78370

H -8.46275 10.91180 9.92036

H -7.18540 10.91765 8.68022

H -8.74433 10.14089 8.34057

H 14.12234 -5.70849 0.94222

H 12.01189 -6.87516 1.12680

H 12.17657 -2.01564 1.99793

H 7.27514 -6.89030 0.92420

H 5.17464 -5.74036 0.58459

H 7.01342 -2.02650 1.74162

H 10.46072 -7.59403 2.39162

H 8.71178 -7.59775 2.31341

H 9.67559 -9.04517 0.54744

H 8.81435 -7.69547 -0.22349

H 10.58144 -7.68330 -0.14751

H 4.02230 -4.32808 -0.76572

H 1.89983 -3.17077 -1.14731

H 2.80699 -0.45604 2.07802

H 4.91561 -1.63248 2.47214

H 16.08123 -4.63073 1.62768

H 17.97409 -2.82633 1.27968

H 18.26939 -0.29099 0.79478

H 16.94937 0.37288 -0.19828

H 16.97675 0.67164 1.55081

SCF Done: E(RCAM-B3LYP) = -7547.07868913   A.U. after   1 cycles

Zero-point correction=   1.543990 (Hartree/Particle)

Thermal correction to Energy=   1.643520

Thermal correction to Enthalpy=   1.644464

Thermal correction to Gibbs Free Energy=   1.383542

Sum of electronic and zero-point Energies=   -7545.534699

Sum of electronic and thermal Energies=   -7545.435169

Sum of electronic and thermal Enthalpies=   -7545.434225

Sum of electronic and thermal Free Energies=   -7545.695148

6.12.2 Computed excitation energies of compound 5m (cam-B3LYP/def2svp SMD CH_2Cl_2 GD3BJ)

Excitation energies and oscillator strengths:

Excited State 1: Singlet-A 3.4872 eV 355.54 nm f=0.4436 <S**2>=0.000

422 -> 428 -0.18326

422 -> 429 -0.15301

424 -> 426 -0.11674

424 -> 427 -0.13683

424 -> 428 0.27237

424 -> 429 0.21939

425 -> 426 0.31605

425 -> 427 -0.17101

425 -> 428 -0.25655

This state for optimization and/or second-order correction.

Total Energy, E(TD-HF/TD-DFT) = -7546.95053556

Copying the excited state density for this state as the 1-particle RhoCI density.

Excited State 2: Singlet-A 3.4940 eV 354.85 nm f=0.7263 <S**2>=0.000

422 -> 427 0.29703

423 -> 426 0.10536

423 -> 429 0.19919

424 -> 426 0.28632

424 -> 427 -0.14363

424 -> 429 0.15655

425 -> 427 -0.27162

425 -> 428 0.14658

425 -> 429 0.17499

Excited State 3: Singlet-A 3.5014 eV 354.10 nm f=1.1341 <S**2>=0.000

422 -> 426 0.10024

422 -> 427 0.16070

422 -> 428 -0.16461

422 -> 429 0.27775

423 -> 426 0.27772

423 -> 427 0.18174

423 -> 428 0.25154

423 -> 429 -0.14671

424 -> 427 0.16992

424 -> 429 0.13442

425 -> 428 -0.11881

Excited State 4: Singlet-A 3.5278 eV 351.45 nm f=0.0367 <S**2>=0.000

422 -> 426 0.29456

422 -> 427 0.11381

423 -> 427 0.14498

423 -> 428 -0.23678

423 -> 429 0.26782

424 -> 427 0.21987

424 -> 428 0.16729

425 -> 428 -0.13061

425 -> 429 -0.20809

Excited State 5: Singlet-A 4.1991 eV 295.26 nm f=0.5088 <S**2>=0.000

422 -> 435 -0.22104

422 -> 437 0.11613

423 -> 435 0.39169

423 -> 436 -0.14450

424 -> 435 -0.16227

424 -> 438 0.11154

425 -> 437 0.15043

425 -> 438 -0.11181

Excited State 6: Singlet-A 4.2014 eV 295.10 nm f=0.2240 <S**2>=0.000

422 -> 435 -0.15334

422 -> 438 0.10396

422 -> 441 0.10670

423 -> 435 0.23337

424 -> 431 -0.11486

424 -> 437 0.21061

424 -> 438 -0.14655

425 -> 437 -0.19542

425 -> 438 0.19947

425 -> 439 -0.11858

425 -> 440 -0.12571

Excited State 7: Singlet-A 4.2073 eV 294.68 nm f=0.3425 <S**2>=0.000

422 -> 440 -0.17124

424 -> 432 -0.11480

424 -> 440 0.26804

424 -> 441 0.25320

425 -> 432 -0.11031

425 -> 433 0.11565

425 -> 440 0.29817

425 -> 441 0.25024

Excited State 8: Singlet-A 4.2123 eV 294.34 nm f=0.2204 <S**2>=0.000

422 -> 430 -0.11638

422 -> 432 0.11669

422 -> 433 0.10238

422 -> 436 -0.15147

422 -> 438 -0.10144

422 -> 439 0.14016

422 -> 440 -0.21897

422 -> 441 0.27145

423 -> 439 0.10097

423 -> 440 -0.19536

423 -> 441 0.19270

424 -> 440 -0.16203

425 -> 441 -0.10861

Excited State 9: Singlet-A 4.2617 eV 290.93 nm f=0.2370 <S**2>=0.000

422 -> 431 0.15481

422 -> 433 -0.11134

422 -> 434 0.11070

422 -> 437 -0.12493

422 -> 442 0.11160

423 -> 430 0.15736

423 -> 431 -0.19121

423 -> 432 -0.12552

423 -> 433 0.14793

423 -> 434 -0.14741

423 -> 435 0.15664

423 -> 437 0.22183

423 -> 438 0.11135

423 -> 441 0.11190

424 -> 437 -0.11314

Excited State 10: Singlet-A 4.2709 eV 290.30 nm f=0.2416 <S**2>=0.000

422 -> 432 -0.11118

422 -> 433 -0.11786

422 -> 436 0.19017

422 -> 437 -0.11352

423 -> 436 0.11264

424 -> 430 0.12263

424 -> 431 0.14324

424 -> 437	0.12312
424 -> 438	0.13122
424 -> 442	0.16493
425 -> 430	-0.14017
425 -> 431	-0.12389
425 -> 437	-0.14717
425 -> 438	-0.14963
425 -> 442	-0.13605

6.12.3 Computed emission energies of compound 5m (cam-B3LYP/def2svp SMD CH_2Cl_2 GD3BJ)

Excitation energies and oscillator strengths:

Excited State 1: Singlet-A 2.7004 eV 459.13 nm f=0.3832 <S**2>=0.000

425 -> 426 0.65400

425 -> 449 -0.10699

This state for optimization and/or second-order correction.

Total Energy, E(TD-HF/TD-DFT) = -7546.90179110

Copying the excited state density for this state as the 1-particle RhoCI density.

Excited State 2: Singlet-A 3.5198 eV 352.24 nm f=0.5677 <S**2>=0.000

422 -> 427 -0.10845

422 -> 428 -0.25496

423 -> 428 -0.11936

423 -> 429 -0.17914

424 -> 427 -0.30382

424 -> 428 0.36169

424 -> 429 -0.24105

Excited State 3: Singlet-A 3.5265 eV 351.57 nm f=0.8883 <S**2>=0.000

422 -> 428 0.20124

422 -> 429 0.37556

423 -> 427 0.38111

423 -> 428 0.18572

423 -> 429 -0.13248

423 -> 446 0.10104

424 -> 428 0.14435

Excited State 4: Singlet-A 3.5561 eV 348.66 nm f=0.0740 <S**2>=0.000

422 -> 427 0.35498

422 -> 428 0.10612

423 -> 428 0.17778

423 -> 429 0.34966

424 -> 428 0.25609

424 -> 429 -0.16795

Excited State 5: Singlet-A 3.8095 eV 325.46 nm f=0.4201 <S**2>=0.000

425 -> 426 -0.13249

425 -> 430 0.14388

425 -> 434 0.28139

425 -> 436 -0.34303

425 -> 437 0.16384

425 -> 438 0.10839

425 -> 441 -0.30213

425 -> 442 -0.16210

425 -> 445 -0.13385

Excited State 6: Singlet-A 3.8397 eV 322.90 nm f=0.1002 <S**2>=0.000

425 -> 430 0.48068

425 -> 431 0.27525

425 -> 433 -0.16511

425 -> 442 0.27051

425 -> 445 -0.12144

corrected external emission excitation: -7546.968199 - -7547.065376 = 0.097177 = 2.644313 eV 468.87 nm

6.13 Quantum chemical calculation data of tetrakis(4-(10,10'-dihexyl-10*H*,10'*H*-[3,3'-biphenothiazin]-7-yl)phenyl)silane (5n)

6.13.1 Computed xyz-coordinates of compound 5n (cam-B3LYP/def2svp SMD CH_2Cl_2 GD3BJ)

0 1

C -9.43722 5.75115 -6.71866

C -8.76883 6.95954 -6.92741

C -7.38061 7.03615 -6.91381

C -6.58225 5.90056 -6.71457

C -7.25734 4.67671 -6.54465

C -8.64495 4.61345 -6.52201

S -6.35484 3.15505 -6.51997

N -5.17967 5.97557 -6.69837

C -4.85720 3.72611 -5.77084

C -4.39905 5.03698 -6.00632

C -3.11844 5.35100 -5.52745

C -2.34414 4.41756 -4.84823

C -2.81458 3.12920 -4.58545

C -4.09371 2.81087 -5.05725

C -4.54328 7.20672 -7.16039

C -4.42956 8.33025 -6.13400

C -10.91659 5.66682 -6.70928

C -11.59766 4.73763 -5.91989

C -12.98678 4.67703 -5.89937

C -13.77352 5.52934 -6.68837

C -13.08318 6.43753 -7.51394

C -11.69645 6.51829 -7.50124

C -1.99832 2.13551 -3.84653

C -0.59989 2.13420 -3.94521

C 0.16431 1.19992 -3.25608

C -0.42968 0.22677 -2.43824

C -1.82761 0.23120 -2.34623

C -2.59720 1.16504 -3.03260

Si 0.63463 -0.99490 -1.47990

N -15.17686 5.47268 -6.67061

C -15.96509 6.59209 -6.99190

C -15.49010 7.60035 -7.85365

S -13.96096 7.39138 -8.71763

C -17.26282 6.74817 -6.48050

C -18.04181 7.85756 -6.80045

C -17.54390 8.86079 -7.62196

C -16.25893 8.72500 -8.14002

C -15.81777 4.33264 -6.02001

C -15.98127 4.42550 -4.50525

C -8.07613 -8.92579 4.37996

C -7.47031 -10.14662 4.07396

C -6.52016 -10.25611 3.06497

C -6.14083 -9.15217 2.28746

C -6.78204 -7.93150 2.57076

C -7.70257 -7.82069 3.60588

S -6.53438 -6.52814 1.52278

N -5.17762 -9.25817 1.26941

C -4.86408 -6.83618 1.02760

C -4.40122 -8.15770 0.87514

C -3.13167 -8.31646 0.29975

C -2.36314 -7.22394 -0.08515

C -2.80676 -5.91371 0.10608

C -4.07426 -5.74860 0.67719

C -4.76594 -10.59137 0.83615

C -3.69125 -11.27002 1.68108

C -9.07320 -8.80054 5.46921

C -10.11849 -7.87606 5.41206

C -11.06192 -7.77861 6.42887

C -10.99816 -8.58699 7.57324

C -9.92095 -9.49079 7.64657

C -9.00278 -9.60849 6.61068

C -1.97862 -4.74409 -0.27562

C -1.09984 -4.80272 -1.36504

C -0.31908 -3.70606 -1.71578

C -0.38287 -2.50394 -0.99911

C -1.26278 -2.45213 0.09281

C -2.04497 -3.54493 0.44777

N -11.94919 -8.49221 8.60251

C -12.24077 -9.58450 9.43875

C -11.28227 -10.58387 9.69802

S -9.61514 -10.38899 9.13967

C -13.49130 -9.72137 10.06120

C -13.77650 -10.80541 10.88793

C -12.83268 -11.80177 11.10245

C -11.58563 -11.68424 10.49490

C -12.85603 -7.34703 8.60857

C -14.09142 -7.45838 7.71890

C 4.98877 7.67284 7.44772

C 4.80581 6.88675 8.58758

C 4.63168 5.50986 8.50330

C 4.65502 4.83897 7.27202

C 4.87497 5.62851 6.12732

C 5.01262 7.00810 6.21557

S 5.14449 4.86344 4.55490

N 4.48177 3.44773 7.18312

C 4.11787 3.43656 4.75671

C 3.97017 2.84243 6.02515

C 3.29935 1.61132 6.06912

C 2.79351 1.01486 4.91980

C 2.90513 1.62587 3.66907

C 3.57458 2.85450 3.61848

C 4.55889 2.65595 8.40836

C 3.28101 2.57467 9.23836

C 5.15602 9.14309 7.52866

C 4.70882 9.99240 6.51402

C 4.88640 11.36957 6.58486

C 5.50099 11.97768 7.68955

C 5.91269 11.12320 8.72979

C 5.76711 9.74451 8.63535

C 2.34709 1.00517 2.44313

C 2.30681 -0.38819 2.29258

C 1.78253 -0.96752 1.14317

C 1.28167 -0.18545 0.09122

C 1.32098 1.20584 0.24942

C 1.84131 1.79095 1.39946

N 5.68535 13.36862 7.76389

C 6.72613 13.93498 8.52117

C 7.26092 13.27161 9.64310

S 6.50235 11.79580 10.25544

C 7.26994 15.18906 8.20165

C 8.30200 15.74639 8.95276

C 8.84336 15.06150 10.03310

C 8.31767 13.81652 10.36711

C 4.98794 14.21328 6.79766

C 5.65681 14.37056 5.43476

C 12.50415 -4.54289 -5.96029

C 12.32780 -4.16939 -7.29451

C 11.06319 -3.95183 -7.82980

C 9.90064 -4.11792 -7.06317

C 10.07872 -4.53318 -5.72975

C 11.34656 -4.71450 -5.19139

S 8.68164 -4.99129 -4.74553

N 8.62107 -3.89373 -7.59842

C 7.48491 -3.87848 -5.42351

C 7.53854 -3.52327 -6.78553

C 6.45120 -2.79324 -7.28817

C 5.38286 -2.42530 -6.47835

C 5.35243 -2.74219 -5.11871

C 6.43325 -3.47337 -4.61140

C 8.48707 -3.75072 -9.04609

C 8.76939 -2.36274 -9.61452

C 13.85089 -4.75081 -5.37810

C 14.92497 -5.20401 -6.14730

C 16.18214 -5.41485 -5.59191

C 16.44085 -5.16062 -4.23701

C 15.36673 -4.66576 -3.47283

C 14.10393 -4.49262 -4.02531

C 4.22903 -2.32530 -4.24499

C 2.92378 -2.21901 -4.74597

C 1.87538 -1.81579 -3.92758

C 2.07853 -1.50862 -2.57324

C 3.38463 -1.61787 -2.07889

C 4.43865 -2.01659 -2.89492

N 17.70800 -5.37307 -3.66988

C 17.86190 -5.66456 -2.30263

C 16.92969 -5.21132 -1.34877

S 15.64127 -4.09501 -1.82096

C 18.95432 -6.40622 -1.82732

C 19.10406 -6.69147 -0.47228

C 18.15637 -6.26774 0.45070

C 17.06464 -5.53124 -0.00067

C 18.84466 -5.58652 -4.56231

C 19.04085 -7.00806 -5.08289

H -9.33632 7.88026 -7.07327

H -6.92281 8.01169 -7.05578

H -9.11732 3.63816 -6.39001

H -2.70565 6.34564 -5.67378

H -1.35972 4.72307 -4.48991

H -4.49527 1.80715 -4.90580

H -3.54554 6.93958 -7.53438

H -5.10588 7.55690 -8.03641

H -3.94184 9.20080 -6.59840

H -5.41157 8.65328 -5.76252

H -3.83145 8.03570 -5.26104

H -11.04068 4.05757 -5.27303

H -13.45302 3.94765 -5.24219

H -11.21371 7.23900 -8.16395

H -0.10140 2.86050 -4.58980

H 1.25114 1.22561 -3.37215

H -2.33600 -0.50844 -1.72298

H -3.68289 1.14742 -2.91929

H -17.68553 5.99723 -5.81834

H -19.04897 7.93330 -6.38501

H -18.14525 9.73857 -7.86520

H -15.84642 9.49188 -8.79979

H -16.79914 4.19355 -6.49364

H -15.23359 3.43867 -6.27721

H -16.46596 3.50989 -4.13358

H -15.01663 4.52840 -3.99009

H -16.60288 5.28025 -4.20628

H -7.74433 -11.04611 4.62796

H -6.07761 -11.23261 2.88619

H -8.13997 -6.84107 3.80740

H -2.71580 -9.30943 0.15141

H -1.37709 -7.40890 -0.51414

H -4.48133 -4.74544 0.81722

H -5.66724 -11.21849 0.80378

H -4.42459 -10.50597 -0.20441

H -3.46593 -12.26090 1.25836

H -2.75669 -10.69333 1.70404

H -4.01169 -11.41292 2.72193

H -10.22951 -7.22988 4.53969

H -11.86595 -7.05728 6.30940

H -8.18508 -10.32326 6.72127

H -1.03484 -5.71538 -1.96050

H 0.35307 -3.79594 -2.57246

H -1.33348 -1.54164 0.69406

H -2.70104 -3.47221 1.31736

H -14.26595 -8.97487 9.90620

H -14.76080 -10.86732 11.35698

H -13.05631 -12.66014 11.73843

H -10.81897 -12.44562 10.65648

H -13.16127 -7.17677 9.65004

H -12.26912 -6.46361 8.32203

H -14.69416 -6.54258 7.81609

H -13.83043 -7.57760 6.65862

H -14.72691 -8.31033 7.99563

H 4.76380 7.35466 9.57252

H 4.46523 4.95961 9.42588

H 5.18678 7.57080 5.29645

H 3.15350 1.09851 7.01622

H 2.26992 0.06252 5.01747

H 3.71870 3.35660 2.65992

H 4.88390 1.64554 8.12492

H 5.37387 3.07264 9.01585

H 3.46464 1.95781 10.13119

H 2.94183 3.56311 9.57646

H 2.45222 2.11925 8.67968

H 4.19946 9.57993 5.64135

H 4.52483 11.97183 5.75549

H 6.13987 9.12714 9.45479

H 2.70801 -1.03004 3.07924

H 1.76965 -2.05823 1.06683

H 0.92808 1.85549 -0.53643

H 1.83371 2.87857 1.49482

H 6.89037 15.75222 7.35304

H 8.68909 16.72806 8.67126

H 9.66138 15.48800 10.61636

H 8.71460 13.26072 11.21982

H 3.97751 13.80104 6.67180

H 4.85129 15.19910 7.26255

H 5.04514 15.03118 4.80161

H 6.65907 14.81274 5.51356

H 5.76507 13.41036 4.91252

H 13.19608 -4.00784 -7.93530

H 10.99745 -3.63540 -8.86762

H 11.42588 -5.03945 -4.15221

H 6.42710 -2.48927 -8.33129

H 4.57215 -1.84506 -6.92187

H 6.44118 -3.77558 -3.56238

H 9.15539 -4.48799 -9.51118

H 7.46817 -4.06264 -9.31298

H 8.63609 -2.38222 -10.70688

H 8.09051 -1.60149 -9.20688

H 9.79500 -2.02917 -9.40677

H 14.78067 -5.43372 -7.20431

H 16.96732 -5.79459 -6.24040

H 13.30871 -4.10312 -3.38681

H 2.72164 -2.46920 -5.78921

H 0.87409 -1.74077 -4.36055

H 3.59459 -1.37506 -1.03451

H 5.44662 -2.06477 -2.47832

H 19.70822 -6.77504 -2.51782

H 19.97397 -7.26572 -0.14636

H 18.26235 -6.49999 1.51191

H 16.31043 -5.17413 0.70440

H 18.73167 -4.89262 -5.40682

H 19.74666 -5.25940 -4.02700

H 19.92259 -7.03619 -5.74111

H 19.20461 -7.72892 -4.27064

H 18.17751 -7.36172 -5.66240

SCF Done: E(RCAM-B3LYP) = -9152.93892440 A.U. after 1 cycles

Zero-point correction= 2.113434 (Hartree/Particle)

Thermal correction to Energy= 2.241759

Thermal correction to Enthalpy= 2.242704

Thermal correction to Gibbs Free Energy= 1.915873

Sum of electronic and zero-point Energies= -9150.825490

Sum of electronic and thermal Energies= -9150.697165

Sum of electronic and thermal Enthalpies= -9150.696221

Sum of electronic and thermal Free Energies= -9151.023051

6.13.2 Computed excitation energies of compound 5n (cam-B3LYP/def2svp SMD CH_2Cl_2 GD3BJ)

Excitation energies and oscillator strengths:

Excited State 1: Singlet-A 3.5073 eV 353.50 nm f=0.9737 <S**2>=0.000

557 -> 562 0.11412

557 -> 563 -0.13458

558 -> 563 -0.26094

559 -> 562 -0.24528

559 -> 563 0.26640

559 -> 565 -0.27336

559 -> 566 0.10703

560 -> 562 -0.13252

560 -> 563 0.13615

561 -> 564 -0.10366

This state for optimization and/or second-order correction.

Total Energy, E(TD-HF/TD-DFT) = -9152.81003298

Copying the excited state density for this state as the 1-particle RhoCI density.

Excited State 2: Singlet-A 3.5117 eV 353.06 nm f=0.6391 <S**2>=0.000

558 -> 562 0.18601

558 -> 563 0.26548

558 -> 565 0.25877

559 -> 562 0.11209

559 -> 563 0.19558

560 -> 562 -0.14642

561 -> 564 -0.22584

561 -> 565 0.10281

Excited State 3: Singlet-A 3.5162 eV 352.61 nm f=1.0087 <S**2>=0.000

554 -> 564 0.11424

560 -> 562 -0.17139

560 -> 564 0.30918

560 -> 565 0.16512

561 -> 562 0.20021

561 -> 564 0.22366

561 -> 565 -0.24080

561 -> 566 -0.10388

Excited State 4: Singlet-A 3.5431 eV 349.94 nm f=0.2549 <S**2>=0.000

558 -> 562 0.21281

558 -> 565 0.10062

558 -> 566 -0.11409

559 -> 563 0.23270

560 -> 562 0.12387

560 -> 564 -0.15965

560 -> 565 -0.25834

561 -> 564 0.26974

561 -> 565 -0.10985

Excited State 5: Singlet-A 3.7696 eV 328.91 nm f=0.0287 <S**2>=0.000

557 -> 562 -0.17589

557 -> 563 0.27249

557 -> 565 -0.18841

557 -> 566 0.16072

557 -> 567 0.18640

557 -> 569 -0.10204

557 -> 584 0.12222

558 -> 567 -0.11355

559 -> 566 0.13805

559 -> 567 0.20121

559 -> 569 -0.15248

559 -> 574 0.10908

Excited State 6: Singlet-A 3.7787 eV 328.11 nm f=0.0226 <S**2>=0.000

554 -> 562 0.14650

554 -> 564 -0.22979

554 -> 565 -0.18506

554 -> 566 -0.14771

554 -> 568 -0.16125

554 -> 585 -0.10026

555 -> 562 -0.10217

555 -> 566 0.10051

560 -> 566 0.16332

560 -> 568 0.23533

560 -> 569 0.14529

560 -> 571 -0.17105

Excited State 7: Singlet-A 3.7833 eV 327.71 nm f=0.0397 <S**2>=0.000

555 -> 564 -0.16036

555 -> 565 0.11873

555 -> 568 -0.11185

556 -> 562 0.13723

556 -> 564 0.21762

556 -> 565 -0.11829

556 -> 566 -0.13148

556 -> 568 0.13677

561 -> 566 -0.12253

561 -> 568 0.23065

561 -> 569 -0.19096

561 -> 570 0.13764

Excited State 8: Singlet-A 3.7893 eV 327.20 nm f=0.0632 <S**2>=0.000

555 -> 562 0.14279

555 -> 563 0.19807

555 -> 565 0.14790
555 -> 566 -0.12223
555 -> 567 0.13831
556 -> 563 0.15344
556 -> 565 0.15630
558 -> 566 0.14843
558 -> 567 -0.17954
558 -> 569 -0.14627
558 -> 570 -0.10196
558 -> 583 -0.12326
559 -> 567 -0.11935

Excited State 9: Singlet-A 4.2164 eV 294.05 nm f=0.3843 <S**2>=0.000
555 -> 582 -0.10788
555 -> 583 -0.13668
556 -> 583 -0.10676
558 -> 572 0.29250
558 -> 573 0.27865
558 -> 574 -0.20582
558 -> 576 0.15897
558 -> 582 -0.10247
559 -> 572 0.16640
559 -> 573 0.15979
559 -> 574 -0.10422

Excited State 10: Singlet-A 4.2392 eV 292.47 nm f=0.1971 <S**2>=0.000
554 -> 564 -0.11346
554 -> 585 0.15384
558 -> 571 0.11583
560 -> 571 0.42064
560 -> 572 -0.10810

560 -> 585 0.13225

6.13.3 Computed emission energies of compound 5n (cam-B3LYP/def2svp SMD CH_2Cl_2 GD3BJ)

Excited State 1: Singlet-A 2.7246 eV 455.05 nm f=0.4867 <S**2>=0.000

557 -> 562 0.12364

561 -> 562 0.63221

561 -> 563 0.10493

This state for optimization and/or second-order correction.

Total Energy, E(TD-HF/TD-DFT) = -9152.73823483

Copying the excited state density for this state as the 1-particle RhoCI density.

Excited State 2: Singlet-A 3.5288 eV 351.35 nm f=0.5828 <S**2>=0.000

555 -> 563 -0.11986

556 -> 564 0.11635

558 -> 563 -0.31293

558 -> 565 0.31293

558 -> 567 0.12383

560 -> 563 0.13682

560 -> 564 -0.25771

560 -> 565 0.14527

Excited State 3: Singlet-A 3.5360 eV 350.63 nm f=0.9991 <S**2>=0.000

554 -> 564 0.10194

555 -> 563 -0.10255

558 -> 563 -0.12369

558 -> 565 0.16121

559 -> 563 0.19314

559 -> 564 0.28426

559 -> 565 0.18261

560 -> 563 -0.14940

560 -> 564 0.22157

560 -> 565 -0.19726

Excited State 4: Singlet-A 3.5654 eV 347.74 nm f=0.3609 <S**2>=0.000

554 -> 563 0.11925

556 -> 564 0.10458

558 -> 563 0.22666

558 -> 565 -0.14130

559 -> 563 0.15567

559 -> 564 0.21776

559 -> 565 0.26364

560 -> 564 -0.24941

560 -> 565 0.11688

Excited State 5: Singlet-A 3.6239 eV 342.12 nm f=0.3147 <S**2>=0.000

557 -> 562 0.30829

557 -> 566 -0.31514

557 -> 567 -0.19578

557 -> 569 0.10304

557 -> 570 0.10797

557 -> 588 0.13747

561 -> 566 0.24887

561 -> 567 0.17016

561 -> 570 -0.14435

561 -> 572 0.10132

Excited State 6: Singlet-A 3.7880 eV 327.31 nm f=0.2143 <S**2>=0.000

554 -> 563 -0.10113

555 -> 565 0.11141

559 -> 568 0.16465

559 -> 569 0.11385

559 -> 571 -0.10202

561 -> 562 -0.10562

561 -> 566 -0.18852

561 -> 570	-0.23409
561 -> 571	-0.11756
561 -> 572	0.16508
561 -> 578	-0.10571
561 -> 589	-0.13469

corrected external emission excitation: -9152.827224 - -9152.925060 = 0.097836 = 2.662253 eV 465.71 nm

6.14 Quantum chemical calculation data of tetrakis(4-(10-hexyl-2-methyl-10*H*-phenothiazin-3-yl)phenyl)silane (5o)

6.14.1 Computed xyz-coordinates of compound 5o (cam-B3LYP/def2svp SMD CH_2Cl_2 GD3BJ)

0 1

C 8.32940 1.70756 9.50370

C 7.84415 2.86883 10.09092

C 6.54490 3.30348 9.83972

C 5.67614 2.57701 9.01039

C 6.17218 1.38108 8.45371

C 7.48366 0.97179 8.67820

S 5.09395 0.28708 7.57663

N 4.36449 3.01533 8.75491

C 3.99194 1.48115 6.87875

C 3.68972 2.66567 7.57268

C 2.67322 3.46734 7.03744

C 1.98753 3.15218 5.86038

C 2.33931 1.98781 5.15670

C 3.34204 1.17237 5.69207

C 3.81355 4.08640 9.58109

C 4.16934 5.51103 9.16423

C 1.67667 1.56934 3.89276

C 1.57462 2.43403 2.79522

C 0.97651 2.01529 1.61212

C 0.45156 0.72091 1.47489

C 0.56616 -0.14122 2.57260

C 1.16704 0.27266 3.75793

Si -0.40305 0.19778 -0.11867

C 0.88999 4.07639 5.40231

C -13.32025 1.86140 1.20599

C -13.41261 3.22784 0.97554

C -12.36851 3.91806 0.36441

C -11.20366 3.25996 -0.06020

C -11.14264 1.86732 0.14708

C -12.17349 1.18946 0.79195

S -9.82065 0.91604 -0.54314

N -10.14922 3.95668 -0.67720

C -8.53287 2.12522 -0.46002

C -8.82459 3.49167 -0.61464

C -7.73103 4.36034 -0.72119

C -6.40143 3.93256 -0.65613

C -6.13290 2.56831 -0.45083

C -7.21879 1.69062 -0.36006

C -10.38666 5.32873 -1.11867

C -10.23684 6.41335 -0.05502

C -4.75594 2.01432 -0.35862

C -3.80748 2.54400 0.52423

C -2.52976 1.99993 0.61135

C -2.14619 0.90684 -0.17670

C -3.10579 0.37135 -1.04877

C -4.38348 0.91273 -1.14008

C -5.31279 4.96046 -0.82147

C 9.41840 5.02174 -7.26012

C 9.05490 4.56531 -8.52041

C 7.75516 4.13253 -8.77224

C 6.76603 4.16435 -7.77697

C 7.14224 4.66581 -6.51471

C 8.45210 5.06157 -6.25868

S 5.92410 4.95018 -5.26399

N 5.45370 3.72788 -8.03152

C 4.79591 3.65025 -5.67025

C 4.62692 3.24199 -7.00473

C 3.59233 2.33293 -7.25913

C 2.74702 1.83314 -6.26353

C 2.95156 2.23289 -4.93157

C 3.99350 3.12860 -4.66579

C 5.05244 3.49769 -9.41699

C 5.42623 2.14240 -10.01206

C 2.13040 1.73985 -3.79413

C 0.73198 1.79913 -3.81331

C -0.01689 1.36034 -2.72583

C 0.59661 0.84443 -1.57686

C 1.99865 0.79728 -1.55923

C 2.75112 1.23471 -2.64390

C 1.67677 0.85203 -6.66434

C 2.95938 -12.37802 -1.15319

C 1.71635 -12.95540 -1.37714

C 0.54583 -12.25663 -1.09119

C 0.57808 -10.96296 -0.54751

C 1.84767 -10.40777 -0.28967

C 3.01367 -11.09615 -0.61286

S 1.99657 -8.88247 0.59372

N -0.60318 -10.25537 -0.26128

C 0.51417 -8.09302 0.04032

C -0.63453 -8.85076 -0.24643

C -1.81005 -8.13674 -0.51149

C -1.87902 -6.74011 -0.50097

C -0.71221 -6.00178 -0.23890

C 0.47049 -6.70648 0.01203

C -1.87159 -10.97995 -0.27272

C -2.53124 -11.16557 -1.63701

C -0.66857 -4.51535 -0.23220

C -1.56738 -3.75465 0.52688

C -1.49224 -2.36648 0.53934

C -0.51585 -1.67925 -0.19798

C 0.38068 -2.44579 -0.95308

C 0.30833 -3.83566 -0.96955

C -3.20095 -6.08914 -0.81323

H 9.35126 1.36992 9.68562

H 8.48184 3.46316 10.74885

H 6.21377 4.22842 10.30438

H 7.83393 0.04715 8.21355

H 2.37930 4.38042 7.54913

H 3.61854 0.25523 5.16746

H 2.72204 3.96327 9.59126

H 4.14227 3.90620 10.61361

H 3.70022 6.22341 9.85994

H 5.25299 5.68896 9.18036

H 3.81512 5.74682 8.15169

H 1.98746 3.44203 2.86121

H 0.92915 2.71528 0.77338

H 0.17926 -1.16104 2.50888

H 1.23443 -0.42011 4.59980

H 0.04827 3.52407 4.96195

H 0.51014 4.67083 6.24486

H 1.24599 4.78397 4.63730

H -14.12950 1.31776 1.69674

H -14.30280 3.78094 1.28273

H -12.47572 4.99014 0.22171

H -12.07932 0.11227 0.94788

H -7.89940 5.42353 -0.87251

H -7.03061 0.62443 -0.21664

H -9.70121 5.52814 -1.95338

H -11.39667 5.36404 -1.54900

H -10.43588 7.39776 -0.50536

H -10.93909 6.27667 0.77843

H -9.22515 6.43689 0.37181

H -4.07751 3.38338 1.16695

H -1.82130 2.43769 1.31864

H -2.85487 -0.48848 -1.67599

H -5.10487 0.48161 -1.83789

H -4.45141 4.55910 -1.37281

H -5.69125 5.83806 -1.36382

H -4.93583 5.31536 0.15050

H 10.43889 5.34898 -7.05323

H 9.78938 4.52914 -9.32787

H 7.51928 3.76493 -9.76738

H 8.70639 5.42859 -5.26155

H 3.42499 1.97744 -8.27259

H 4.15850 3.46274 -3.63916

H 3.96460 3.64056 -9.47009

H 5.48917 4.30335 -10.02301

H 5.06794 2.08879 -11.05142

H 6.51234 1.97921 -10.02210

H 4.97702 1.30721 -9.45805

H 0.22044 2.21576 -4.68229

H -1.10590 1.43403 -2.77623

H 2.52150 0.41157 -0.67978

H 3.84117 1.17634 -2.60457

H 1.53741 0.06675 -5.90867

H 0.70184 1.34627 -6.79877

H 1.93241 0.37075 -7.61848

H 3.87986 -12.91514 -1.38882

H 1.64193 -13.96256 -1.79282

H -0.40471 -12.73986 -1.30085

H 3.97775 -10.62252 -0.41342

H -2.72441 -8.67359 -0.75053

H 1.38436 -6.15017 0.23196

H -1.69423 -11.95989 0.19108

H -2.55654 -10.44984 0.40316

H -3.47296 -11.72219 -1.51504

H -2.76701 -10.20673 -2.11776

H -1.89480 -11.72977 -2.33194

H -2.32674 -4.25618 1.12878

H -2.21343 -1.80877 1.14315

H 1.15175 -1.95345 -1.55059

H 1.01438 -4.40586 -1.57743

H -3.07612 -5.17444 -1.40897

H -3.84843 -6.77888 -1.37219

H -3.74086 -5.80510 0.10373

SCF Done: E(RCAM-B3LYP) = -5340.98135782 A.U. after 1 cycles

Zero-point correction= 1.351177 (Hartree/Particle)

Thermal correction to Energy= 1.432544

Thermal correction to Enthalpy= 1.433488

Thermal correction to Gibbs Free Energy= 1.216987

Sum of electronic and zero-point Energies= -5339.630181

Sum of electronic and thermal Energies= -5339.548814

Sum of electronic and thermal Enthalpies= -5339.547869

Sum of electronic and thermal Free Energies= -5339.764370

6.14.2 Computed excitation energies of compound 5o (cam-B3LYP/def2svp SMD CH_2Cl_2 GD3BJ)

Excitation energies and oscillator strengths:

Excited State 1: Singlet-A 3.7644 eV 329.36 nm f=0.1794 <S**2>=0.000

338 -> 343 0.17228

338 -> 345 -0.12922

339 -> 342 -0.17185

339 -> 343 0.25145

339 -> 344 -0.15671

339 -> 345 -0.15917

339 -> 346 0.10293

340 -> 342 0.24357

340 -> 344 0.21589

340 -> 346 -0.10439

340 -> 348 0.13812

341 -> 343 -0.16147

341 -> 345 0.12074

This state for optimization and/or second-order correction.

Total Energy, E(TD-HF/TD-DFT) = -5340.84302048

Copying the excited state density for this state as the 1-particle RhoCI density.

Excited State 2: Singlet-A 3.7707 eV 328.81 nm f=0.1621 <S**2>=0.000

338 -> 344 -0.20778

339 -> 342 -0.18908

339 -> 343 0.19845

339 -> 344 -0.14119

339 -> 346 0.10879

340 -> 343 -0.23936

340 -> 345 0.25118

340 -> 348 -0.11225

341 -> 342 0.18648

341 -> 343 0.10620

341 -> 348 0.10329

Excited State 3: Singlet-A 3.7919 eV 326.97 nm f=0.2130 <S**2>=0.000

338 -> 342 -0.15375

338 -> 343 0.16101

338 -> 344 0.19538

338 -> 345 0.25529

338 -> 346 0.12211

338 -> 349 0.20132

339 -> 342 0.11094

339 -> 344 -0.18581

340 -> 342 0.11783

340 -> 344 -0.16611

341 -> 343 0.13668

341 -> 345 0.19944

341 -> 347 -0.12803

Excited State 4: Singlet-A 3.8040 eV 325.93 nm f=0.0460 <S**2>=0.000

338 -> 342 0.18256

338 -> 344 -0.15684

338 -> 346 -0.13385

338 -> 347 -0.10328

339 -> 345 0.19133

340 -> 343 0.18242

340 -> 345 0.11874

341 -> 342 0.11941

341 -> 343 0.14505

341 -> 344 -0.21746

341 -> 345 0.26566

341 -> 347 -0.22204

Excited State 5: Singlet-A 4.3558 eV 284.64 nm f=0.5401 <S**2>=0.000

338 -> 346 0.11930

338 -> 347 0.11733

338 -> 348 0.10686

338 -> 349 0.28698

338 -> 350 0.16305

338 -> 351 -0.14039

338 -> 355 -0.11231

338 -> 356 0.11415

339 -> 349 -0.13052

339 -> 351 0.13300

340 -> 347 -0.12974

340 -> 349 -0.11889

341 -> 347 -0.10823

Excited State 6: Singlet-A 4.3631 eV 284.17 nm f=0.2069 <S**2>=0.000

338 -> 347 0.11815

339 -> 346 -0.10961

339 -> 347 0.17592

339 -> 350 0.13766

339 -> 354 0.16568

339 -> 357 0.10558

339 -> 358 -0.10347

340 -> 350 -0.15445

341 -> 347 0.26320

341 -> 348 -0.13417

341 -> 350 -0.13049

341 -> 351 -0.10514

341 -> 358 -0.12777

Excited State 7: Singlet-A 4.3661 eV 283.97 nm f=0.2303 <S**2>=0.000

338 -> 348 0.14557
339 -> 350 -0.19679
339 -> 354 -0.11862
340 -> 348 0.30208
340 -> 349 -0.12782
340 -> 357 0.14910
341 -> 347 0.14098
341 -> 348 -0.19391
341 -> 358 -0.11213

Excited State 8: Singlet-A 4.3787 eV 283.16 nm f=0.1370 <S**2>=0.000
338 -> 348 0.15719
338 -> 349 -0.10511
338 -> 351 0.10627
339 -> 348 0.12919
339 -> 350 0.18379
340 -> 348 0.23679
340 -> 354 -0.12015
340 -> 358 0.13091
341 -> 347 -0.20632
341 -> 348 -0.12596
341 -> 349 0.11585
341 -> 357 -0.14056

Excited State 9: Singlet-A 4.4581 eV 278.11 nm f=0.2160 <S**2>=0.000
338 -> 349 -0.11896
338 -> 350 0.25835
338 -> 351 -0.15679
338 -> 352 0.10723
338 -> 354 -0.14139
338 -> 355 0.11897

339 -> 351 0.19285

340 -> 351 0.18805

341 -> 350 0.11546

341 -> 351 0.18801

341 -> 352 0.13461

341 -> 354 -0.23511

Excited State 10: Singlet-A 4.4592 eV 278.04 nm f=0.3089 <S**2>=0.000

338 -> 349 -0.11619

338 -> 350 0.16578

338 -> 351 -0.20611

338 -> 355 0.12998

339 -> 352 -0.20958

340 -> 350 -0.14759

340 -> 353 0.23890

341 -> 351 -0.21943

341 -> 354 0.24650

6.14.3 Computed emission energies of compound 5o (cam-B3LYP/def2svp SMD CH_2Cl_2 GD3BJ)

Excitation energies and oscillator strengths:

Excited State 1: Singlet-A 2.8299 eV 438.12 nm f=0.0861 <S**2>=0.000

341 -> 342 0.59418

341 -> 343 -0.17336

341 -> 346 0.13789

341 -> 347 -0.23109

341 -> 349 -0.11534

This state for optimization and/or second-order correction.

Total Energy, E(TD-HF/TD-DFT) = -5340.81007282

Copying the excited state density for this state as the 1-particle RhoCI density.

Excited State 2: Singlet-A 3.7820 eV 327.82 nm f=0.1399 <S**2>=0.000

339 -> 343 0.27043

339 -> 344 0.39831

339 -> 345 0.23986

339 -> 346 0.17319

339 -> 350 -0.10668

339 -> 351 -0.10075

339 -> 352 -0.16564

339 -> 353 -0.14301

340 -> 345 0.10730

Excited State 3: Singlet-A 3.8004 eV 326.24 nm f=0.1853 <S**2>=0.000

338 -> 343 0.22615

338 -> 345 -0.25923

338 -> 347 0.14225

338 -> 349 -0.15159

340 -> 343 -0.14954

340 -> 344 0.30394

340 -> 345 -0.24114

340 -> 346 -0.12631

340 -> 348 -0.24352

Excited State 4: Singlet-A 3.8106 eV 325.37 nm f=0.0961 <S**2>=0.000

338 -> 343 0.29497

338 -> 345 -0.27290

338 -> 346 0.13980

338 -> 347 0.17758

338 -> 349 -0.18262

339 -> 345 -0.12490

340 -> 344 -0.23298

340 -> 345 0.18906

340 -> 348 0.18743

Excited State 5: Singlet-A 3.8718 eV 320.22 nm f=0.3084 <S**2>=0.000

337 -> 342 0.10023

341 -> 342 0.21080

341 -> 346 -0.16306

341 -> 347 0.41461

341 -> 349 0.24257

341 -> 359 -0.15962

341 -> 361 -0.26758

Excited State 6: Singlet-A 4.1630 eV 297.82 nm f=0.0440 <S**2>=0.000

323 -> 342 -0.10291

341 -> 359 -0.18504

341 -> 360 -0.60396

341 -> 361 0.20173

corrected external emission excitation: -5340.862998 - -5340.965375 = 0.102377 = 2.785821 eV 445.05 nm

6.15 Quantum chemical calculation data of tetrakis(4-(10-hexyl-2-methoxy-10*H*-phenothiazin-3-yl)phenyl)silane (5p)

6.15.1 Computed xyz-coordinates of compound 5p (cam-B3LYP/def2svp SMD CH_2Cl_2 GD3BJ)

0 1

C -10.33358 -5.43726 -5.10980

C -10.24482 -4.94844 -6.40922

C -9.00984 -4.60818 -6.95623

C -7.82862 -4.78643 -6.22496

C -7.93142 -5.27582 -4.91012

C -9.17120 -5.58349 -4.35668

S -6.44028 -5.57527 -3.99764

N -6.56133 -4.46994 -6.75326

C -5.52282 -4.17321 -4.57979

C -5.64744 -3.79849 -5.92300

C -4.87063 -2.73290 -6.39306

C -3.97001 -2.07429 -5.55421

C -3.84652 -2.44337 -4.19751

C -4.64875 -3.49053 -3.74151

C -6.36647 -4.44239 -8.19827

C -5.05830 -5.09119 -8.62429

C -2.89199 -1.78058 -3.27312

C -2.75078 -0.38737 -3.22302

C -1.87998 0.20890 -2.31754

C -1.10896 -0.55377 -1.42951

C -1.25661 -1.94726 -1.48280

C -2.12728 -2.54905 -2.38508

Si 0.12025 0.23510 -0.24157

C 12.53876 -2.66388 -1.37711

C 12.62603 -2.69809 -2.76512

C 11.76858 -1.92939 -3.54870

C 10.82732 -1.07752 -2.95713

C 10.74427 -1.05591 -1.55309

C 11.58150 -1.85084 -0.77522

S 9.60745 0.06996 -0.78777

N 9.95753 -0.27262 -3.71918

C 8.28996 -0.09058 -1.96487

C 8.61100 -0.19567 -3.32357

C 7.56583 -0.25432 -4.25298

C 6.23312 -0.18160 -3.84397

C 5.90436 -0.08526 -2.47463

C 6.95996 -0.05412 -1.56184

C 10.32554 0.10239 -5.07983

C 10.01847 1.56002 -5.38774

C 4.50303 0.00483 -1.99141

C 3.48827 -0.82212 -2.49102

C 2.19402 -0.74672 -1.98729

C 1.85408 0.15559 -0.97025

C 2.87165 0.98744 -0.48109

C 4.16843 0.91313 -0.97765

C -4.91467 10.89711 4.82256

C -3.94844 11.80373 4.39859

C -3.21327 11.56626 3.23956

C -3.45676 10.43087 2.45662

C -4.43094 9.51766 2.89902

C -5.14039 9.74364 4.07529

S -4.79868 8.11141 1.88257

N -2.74527 10.16992 1.26872

C -3.14289 7.77672 1.34046

C -2.30396 8.85619 1.03728

C -1.02600 8.58613 0.53419

C -0.60458 7.27512 0.30574

C -1.44530 6.18336 0.61284

C -2.70166 6.47272 1.14841

C -2.12649 11.27139 0.53991

C -2.32526 11.16286 -0.96433

C -1.02959 4.77175 0.41366

C -0.36609 4.34789 -0.74702

C -0.02244 3.01316 -0.92262

C -0.32443 2.04156 0.04482

C -0.98206 2.47208 1.20329

C -1.32751 3.80815 1.38531

C -0.81459 -9.21657 8.38488

C -0.01260 -8.66572 9.37923

C 0.11368 -7.28422 9.50446

C -0.59281 -6.42048 8.65795

C -1.39426 -6.98894 7.65133

C -1.49165 -8.37044 7.51002

S -2.34694 -5.90965 6.61535

N -0.50381 -5.01859 8.76920

C -1.14787 -4.61098 6.46465

C -0.38394 -4.26833 7.58682

C 0.51024 -3.19647 7.48270

C 0.62522 -2.46677 6.29835

C -0.13369 -2.81611 5.16053

C -1.00308 -3.90197 5.27755

C -0.10740 -4.41848 10.03795

C -0.94025 -3.19598 10.39260

C -0.04934 -2.06544 3.88214

C 1.17726 -1.67354 3.32981

C 1.23018 -1.01026 2.10858

C 0.06747 -0.70500 1.38820

C -1.15711 -1.10181 1.94502

C -1.21613 -1.76511 3.16580

O -3.18106 -1.06978 -5.98797

C -3.22645 -0.67855 -7.33979

O 5.20683 -0.19632 -4.71925

C 5.46608 -0.24873 -6.10251

O 0.61980 6.98568 -0.18064

C 1.51587 8.02732 -0.48879

O 1.45083 -1.40659 6.18048

C 2.21467 -0.98673 7.28669

H -11.30179 -5.69640 -4.67723

H -11.14826 -4.81118 -7.00743

H -8.97012 -4.19668 -7.96440

H -9.21955 -5.95254 -3.32981

H -4.96761 -2.41856 -7.42820

H -4.58769 -3.78510 -2.69213

H -7.19024 -5.01041 -8.64390

H -6.44535 -3.41566 -8.59885

H -4.99254 -5.10678 -9.72255

H -4.17757 -4.55653 -8.24314

H -5.01117 -6.12800 -8.25919

H -3.33783 0.23629 -3.89675

H -1.80859 1.29921 -2.30218

H -0.67983 -2.58461 -0.80707

H -2.20745 -3.63797 -2.40918

H 13.20456 -3.27228 -0.76209

H 13.35930 -3.34341 -3.25378

H 11.83411 -1.99851 -4.63414

H 11.48622 -1.81936 0.31236

H 7.79878 -0.35321 -5.30895

H 6.73477 0.00195 -0.49520

H 11.40704 -0.04575 -5.17210

H 9.85715 -0.56475 -5.82576

H 10.37727 1.80936 -6.39759

H 8.94334 1.78267 -5.34955

H 10.52771 2.21671 -4.66667

H 3.71785 -1.53593 -3.28170

H 1.43233 -1.41166 -2.40194

H 2.65105 1.71971 0.30045

H 4.93386 1.58483 -0.58313

H -5.48589 11.07840 5.73501

H -3.74606 12.70440 4.98231

H -2.43898 12.27445 2.94586

H -5.88402 9.01157 4.39784

H -0.35085 9.41051 0.32427

H -3.37498 5.64899 1.39285

H -2.60834 12.19371 0.88213

H -1.05479 11.37112 0.78899

H -1.90263 12.05021 -1.45901

H -1.83754 10.27626 -1.39209

H -3.39787 11.11008 -1.20362

H -0.11865 5.07454 -1.52020

H 0.49838 2.72510 -1.84006

H -1.22500 1.75590 1.99193

H -1.82389 4.11158 2.30952

H -0.90820 -10.29923 8.28096

H 0.53975 -9.31501 10.06206

H 0.77378 -6.87773 10.27018

H -2.11486 -8.78173 6.71281

H 1.11921 -2.92704 8.34030

H -1.58974 -4.20565 4.40850

H -0.26433 -5.17519 10.81412

H 0.97113 -4.17851 10.05313

H -0.64761 -2.82345 11.38574

H -0.81182 -2.37415 9.67498

H -2.00831 -3.45849 10.42146

H 2.10172 -1.89925 3.86061

H 2.20759 -0.73173 1.70722

H -2.09178 -0.89018 1.41836

H -2.18765 -2.04673 3.57759

H -2.49269 0.12914 -7.45475

H -2.95349 -1.50787 -8.01337

H -4.22213 -0.29806 -7.62222

H 4.48998 -0.22549 -6.60301

H 6.06111 0.61678 -6.43828

H 5.98962 -1.17704 -6.38539

H 2.42691 7.55052 -0.87172

H 1.11108 8.69841 -1.26452

H 1.77237 8.62181 0.40370

H 2.78055 -0.10614 6.95767

H 1.57555 -0.70285 8.13921

H 2.92422 -1.76554 7.61217

SCF Done: E(RCAM-B3LYP) = -5641.51026665 A.U. after 1 cycles

Zero-point correction= 1.370475 (Hartree/Particle)

Thermal correction to Energy= 1.455823

Thermal correction to Enthalpy= 1.456767

Thermal correction to Gibbs Free Energy= 1.232718

Sum of electronic and zero-point Energies= -5640.139792

Sum of electronic and thermal Energies= -5640.054444

Sum of electronic and thermal Enthalpies= -5640.053500

Sum of electronic and thermal Free Energies= -5640.277549

6.15.2 Computed excitation energies of compound 5p (cam-B3LYP/def2svp SMD CH_2Cl_2 GD3BJ)

Excitation energies and oscillator strengths:

Excited State 1: Singlet-A 3.9959 eV 310.28 nm f=0.5420 <S**2>=0.000

354 -> 359 -0.22588

354 -> 361 0.15332

355 -> 358 0.24327

355 -> 359 -0.23288

355 -> 361 0.24623

355 -> 370 -0.10473

356 -> 358 0.23406

356 -> 359 -0.21175

357 -> 360 -0.12053

This state for optimization and/or second-order correction.

Total Energy, E(TD-HF/TD-DFT) = -5641.36342154

Copying the excited state density for this state as the 1-particle RhoCI density.

Excited State 2: Singlet-A 4.0232 eV 308.17 nm f=0.4586 <S**2>=0.000

354 -> 360 -0.21495

354 -> 361 0.10721

355 -> 358 -0.14523

355 -> 359 -0.15818

356 -> 359 0.14913

356 -> 360 0.24743

357 -> 358 -0.22841

357 -> 360 -0.18234

357 -> 361 0.26670

Excited State 3: Singlet-A 4.0238 eV 308.13 nm f=0.6382 <S**2>=0.000

354 -> 359 0.22318

354 -> 361 0.24013

355 -> 358 -0.13808

355 -> 359 -0.17861

355 -> 361 -0.13681

356 -> 358 0.17294

356 -> 360 -0.21631

357 -> 358 0.20122

357 -> 360 -0.18185

357 -> 361 -0.14553

Excited State 4: Singlet-A 4.0714 eV 304.52 nm f=0.0495 <S**2>=0.000

354 -> 358 0.26786

354 -> 359 0.10326

354 -> 362 -0.12342

355 -> 359 -0.22365

355 -> 360 0.11168

356 -> 361 0.28214

357 -> 359 0.12569

357 -> 360 0.28925

357 -> 364 0.11011

Excited State 5: Singlet-A 4.4515 eV 278.52 nm f=0.4063 <S**2>=0.000

354 -> 363 0.19992

354 -> 367 -0.22006

354 -> 371 0.10276

355 -> 363 -0.17675

355 -> 367 0.20781

356 -> 365 0.10801

356 -> 368 0.16947

357 -> 364 -0.15219

357 -> 368 0.11429

357 -> 369 0.14069

357 -> 372 -0.10467

Excited State 6: Singlet-A 4.4519 eV 278.50 nm f=0.2978 <S**2>=0.000

354 -> 364 0.13882

354 -> 369 -0.13968

356 -> 364 -0.18737

356 -> 369 0.19480

356 -> 372 -0.10881

357 -> 358 -0.10195

357 -> 365 0.23418

357 -> 368 0.24220

357 -> 369 -0.18215

357 -> 373 -0.14625

Excited State 7: Singlet-A 4.4649 eV 277.69 nm f=0.3048 <S**2>=0.000

354 -> 363 0.11050

354 -> 365 0.11183

354 -> 367 -0.14146

354 -> 368 0.13205

354 -> 370 0.10912

355 -> 367 0.11322

355 -> 369 -0.11715

355 -> 370 0.15459

356 -> 368 -0.15477

357 -> 364 0.15286

357 -> 367 -0.10347

357 -> 369 -0.16726

Excited State 8: Singlet-A 4.4902 eV 276.12 nm f=0.1001 <S**2>=0.000

354 -> 366 0.11090

354 -> 368 -0.11051

354 -> 370 0.19573

354 -> 373 0.10055
355 -> 366 0.13643
355 -> 367 -0.10065
355 -> 370 0.29201
355 -> 374 -0.13174
356 -> 370 0.23405

Excited State 9: Singlet-A 4.6024 eV 269.39 nm f=0.1525 <S**2>=0.000
349 -> 366 0.12363
354 -> 365 0.12487
354 -> 366 0.20516
354 -> 370 -0.13230
355 -> 365 0.11573
355 -> 366 0.32649
355 -> 370 -0.18925
356 -> 366 0.25646
356 -> 370 -0.14243
357 -> 364 0.11387
357 -> 369 0.11378

Excited State 10: Singlet-A 4.6031 eV 269.35 nm f=0.1274 <S**2>=0.000
354 -> 363 0.27485
354 -> 367 0.23564
355 -> 363 -0.24162
355 -> 367 -0.20576
356 -> 364 -0.12572
356 -> 365 0.12039
356 -> 368 -0.15374
357 -> 364 -0.18323
357 -> 368 -0.13108

6.15.3 Computed emission energies of compound 5p (cam-B3LYP/def2svp SMD CH_2Cl_2 GD3BJ)

Excitation energies and oscillator strengths:

Excited State 1: Singlet-A 2.8188 eV 439.84 nm f=0.1870 <S**2>=0.000

357 -> 358 0.62050

357 -> 359 -0.13370

357 -> 364 -0.23970

This state for optimization and/or second-order correction.

Total Energy, E(TD-HF/TD-DFT) = -5641.34291929

Copying the excited state density for this state as the 1-particle RhoCI density.

Excited State 2: Singlet-A 3.7392 eV 331.58 nm f=0.3350 <S**2>=0.000

354 -> 359 0.20669

354 -> 360 0.37060

354 -> 361 -0.15076

354 -> 363 0.19236

355 -> 359 0.14739

356 -> 359 -0.18436

356 -> 360 0.18863

356 -> 361 0.22599

356 -> 362 0.10899

Excited State 3: Singlet-A 3.7418 eV 331.35 nm f=0.3035 <S**2>=0.000

354 -> 361 0.18523

355 -> 359 -0.25244

355 -> 361 -0.34383

355 -> 362 0.10838

355 -> 365 -0.14486

356 -> 359 -0.21871

356 -> 360 0.25809

356 -> 362 0.12793

356 -> 366 -0.10449

Excited State 4: Singlet-A 3.7624 eV 329.53 nm f=0.3068 <S**2>=0.000

354 -> 359 -0.20778

354 -> 360 -0.12288

354 -> 363 -0.10272

355 -> 359 0.20344

355 -> 360 -0.14265

355 -> 361 0.22583

355 -> 362 -0.11368

356 -> 360 0.20135

356 -> 361 0.21386

356 -> 365 0.10002

357 -> 364 0.25675

357 -> 377 -0.10253

Excited State 5: Singlet-A 3.8158 eV 324.93 nm f=0.2212 <S**2>=0.000

349 -> 358 0.12807

354 -> 359 -0.10519

356 -> 360 0.10557

356 -> 361 0.10401

357 -> 358 -0.18561

357 -> 363 -0.12992

357 -> 364 -0.47315

357 -> 377 0.22518

Excited State 6: Singlet-A 4.1521 eV 298.60 nm f=0.0309 <S**2>=0.000

357 -> 370 0.13088

357 -> 371 -0.35700

357 -> 372 0.26511

357 -> 373 0.24323

357 -> 374 -0.22916

357 -> 375 0.29299

357 -> 376 -0.13740

corrected external emission excitation: -5641.391125 - -5641.492575 = 0.101450 = 2.760600 eV 449.12 nm

6.16 Tetrakis(4-(10-hexyl-2-(methylthio)-10*H*-phenothiazin-3-yl)phenyl)silane (5q)

6.16.1 Computed xyz-coordinates of compound 5q (cam-B3LYP/def2svp SMD CH_2Cl_2 GD3BJ)

0 1

C -8.00283 4.67358 -9.20895

C -9.21292 4.23969 -8.68275

C -9.31672 3.87793 -7.34179

C -8.21682 3.96199 -6.47437

C -7.00665 4.43684 -7.01611

C -6.89914 4.76203 -8.36535

S -5.62424 4.77155 -5.96330

N -8.31850 3.59842 -5.11869

C -5.89698 3.52722 -4.73922

C -7.20516 3.13885 -4.39935

C -7.34221 2.29545 -3.28905

C -6.24446 1.81688 -2.56927

C -4.93883 2.15619 -2.95981

C -4.80265 3.02827 -4.04436

C -9.64792 3.39974 -4.54612

C -10.27467 2.02487 -4.76082

C -3.71653 1.65716 -2.27637

C -3.47777 0.28619 -2.12211

C -2.33632 -0.16245 -1.46907

C -1.39250 0.73422 -0.94376

C -1.62573 2.10232 -1.12761

C -2.76617 2.55753 -1.78428

Si 0.07509 0.10006 0.04990

C -3.91531 -6.04152 10.87302

C -3.46468 -5.14444 11.83298

C -3.15111 -3.83191 11.48841

C -3.30197 -3.36482 10.17382

C -3.79308 -4.27768 9.22032

C -4.06990 -5.59832 9.56253

S -4.20885 -3.73183 7.58909

N -2.98646 -2.03885 9.82289

C -3.00293 -2.45386 7.40634

C -2.58777 -1.70290 8.52048

C -1.78275 -0.58616 8.26068

C -1.36388 -0.24499 6.97203

C -1.72749 -1.04235 5.87452

C -2.56353 -2.13424 6.12812

C -2.76491 -1.06720 10.89121

C -1.36508 -1.04257 11.49886

C -1.28974 -0.78019 4.47801

C 0.06698 -0.67386 4.14789

C 0.45731 -0.41781 2.83935

C -0.48501 -0.26403 1.80963

C -1.83803 -0.39133 2.14608

C -2.23496 -0.64794 3.45605

C 10.37797 7.53343 3.22364

C 10.53285 8.23629 2.03567

C 9.49868 8.29661 1.10469

C 8.26321 7.67504 1.34234

C 8.11125 6.99619 2.56710

C 9.16061 6.90786 3.47755

S 6.53229 6.35850 3.04868

N 7.21632 7.73566 0.40386

C 5.89232 5.95718 1.45178

C 6.24038 6.73036 0.32986

C 5.54117 6.46792 -0.85552

C 4.57387 5.46401 -0.95143

C 4.28193 4.65402 0.15782

C 4.95296 4.93909 1.35111

C 7.32160 8.68744 -0.69964

C 8.12589 8.22665 -1.91185

C 3.29896 3.53880 0.13507

C 3.40800 2.48829 -0.78272

C 2.47832 1.45409 -0.78918

C 1.40706 1.42824 0.11484

C 1.31947 2.47300 1.04596

C 2.24786 3.50917 1.05821

C 7.91460 -8.66226 -5.64194

C 7.18309 -9.82143 -5.41688

C 5.83065 -9.75655 -5.09069

C 5.15775 -8.52853 -5.00259

C 5.90568 -7.36520 -5.26839

C 7.26561 -7.43360 -5.55792

S 5.10340 -5.79199 -5.38431

N 3.79131 -8.46066 -4.67392

C 3.80535 -6.05230 -4.21430

C 3.24639 -7.33139 -4.04440

C 2.10175 -7.41601 -3.24112

C 1.55104 -6.30396 -2.59899

C 2.15545 -5.04253 -2.72366

C 3.27707 -4.95063 -3.55351

C 3.00234 -9.68986 -4.70630

C 3.06172 -10.56325 -3.45619

C 1.66274 -3.81621 -2.04209

C 1.35135 -2.67328 -2.78776

C 0.88247 -1.52253 -2.16238

C 0.71956 -1.46539 -0.77040

C 1.04944 -2.60988 -0.03155

C 1.51291 -3.76514 -0.65228

S -6.61232 0.74817 -1.18435

C -5.91824 1.68135 0.20597

S -0.33443 1.20978 6.83747

C -1.31885 2.27140 5.74707

S 3.78971 5.27324 -2.54604

C 2.04746 5.57698 -2.14865

S 0.09040 -6.60094 -1.61279

C -1.13831 -5.56124 -2.44619

H -7.91336 4.94590 -10.26208

H -10.09697 4.16582 -9.31966

H -10.27830 3.52567 -6.97712

H -5.93751 5.11065 -8.74907

H -8.32841 2.00050 -2.94222

H -3.80093 3.32790 -4.35985

H -9.57679 3.61350 -3.47085

H -10.30234 4.17820 -4.96090

H -10.40300 1.78893 -5.82594

H -9.67118 1.22051 -4.31888

H -11.26786 1.99906 -4.28737

H -4.19622 -0.43354 -2.51574

H -2.17962 -1.23984 -1.36743

H -0.91104 2.83515 -0.74512

H -2.93305 3.63092 -1.89958

H -4.14984 -7.07467 11.13540

H -3.33882 -5.46269 12.87006

H -2.78183 -3.16991 12.26731

H -4.43319 -6.27904 8.78915

H -1.47189 0.06732 9.07062

H -2.88155 -2.76213 5.29305

H -3.01417 -0.07571 10.48921

H -3.50967 -1.26567 11.67372

H -1.09190 -2.00602 11.95014

H -0.59413 -0.80021 10.75484

H -1.32340 -0.27638 12.28785

H 0.82149 -0.78577 4.92712

H 1.52487 -0.32958 2.61973

H -2.60628 -0.27643 1.37776

H -3.29832 -0.72359 3.69445

H 11.18995 7.47225 3.95043

H 11.47488 8.74145 1.81187

H 9.67116 8.84081 0.17976

H 9.00694 6.35741 4.40863

H 5.72519 7.06393 -1.74465

H 4.73323 4.33958 2.23714

H 6.29952 8.94759 -1.00688

H 7.75349 9.61199 -0.29332

H 9.16919 7.99840 -1.65512

H 7.69975 7.32667 -2.37567

H 8.13185 9.02461 -2.66983

H 4.23081 2.48142 -1.49839

H 2.59829 0.64761 -1.51650

H 0.50810 2.48720 1.77851

H 2.14776 4.31896 1.78453

H 8.97769 -8.70705 -5.88501

H 7.66470 -10.79943 -5.48171

H 5.30023 -10.68646 -4.90288

H 7.81378 -6.50661 -5.74142

H 1.59341 -8.36664 -3.10777

H 3.75842 -3.97946 -3.68702

H 1.96140 -9.40507 -4.91228

H 3.33178 -10.26637 -5.58130

H 4.08267 -10.90243 -3.23456

H 2.69195 -10.03775 -2.56539

H 2.43445 -11.45525 -3.60497

H 1.45505 -2.69598 -3.87496

H 0.62433 -0.65818 -2.78033

H 0.93939 -2.60775 1.05539

H 1.75706 -4.64196 -0.05166

H -6.21319 1.13934 1.11573

H -4.82222 1.72902 0.16270

H -6.33687 2.69633 0.24483

H -0.80176 3.24091 5.71672

H -1.37803 1.86991 4.72697

H -2.32941 2.41938 6.15184

H 1.51540 5.60999 -3.10994

H 1.62007 4.77260 -1.53578

H 1.92441 6.54311 -1.64034

H -2.10117 -5.77571 -1.96122

H -0.91543 -4.49221 -2.33220

H -1.21099 -5.81824 -3.51187

SCF Done: E(RCAM-B3LYP) = -6933.27130123 A.U. after 1 cycles

Zero-point correction= 1.354914 (Hartree/Particle)

Thermal correction to Energy= 1.443572

Thermal correction to Enthalpy= 1.444516

Thermal correction to Gibbs Free Energy= 1.210965

Sum of electronic and zero-point Energies= -6931.916387

Sum of electronic and thermal Energies= -6931.827729

Sum of electronic and thermal Enthalpies= -6931.826785

Sum of electronic and thermal Free Energies= -6932.060336

6.16.2 Computed excitation energies of compound 5q (cam-B3LYP/def2svp SMD CH_2Cl_2 GD3BJ)

Excitation energies and oscillator strengths:

Excited State 1: Singlet-A 3.7177 eV 333.50 nm f=0.1231 <S**2>=0.000

370 -> 375 -0.15221

370 -> 376 -0.15919

370 -> 377 0.15871

371 -> 375 0.11603

371 -> 376 -0.16808

372 -> 374 -0.28335

372 -> 376 0.30360

372 -> 377 -0.24136

372 -> 378 0.16264

372 -> 383 -0.16178

This state for optimization and/or second-order correction.

Total Energy, E(TD-HF/TD-DFT) = -6933.13455924

Copying the excited state density for this state as the 1-particle RhoCI density.

Excited State 2: Singlet-A 3.7198 eV 333.31 nm f=0.1249 <S**2>=0.000

370 -> 374 0.14678

370 -> 375 0.33912

370 -> 377 -0.19296

370 -> 379 -0.11929

370 -> 384 -0.11551

371 -> 374 -0.22244

371 -> 376 -0.28095

371 -> 378 0.10743

371 -> 380 -0.11863

372 -> 377 -0.13887

Excited State 3: Singlet-A 3.7229 eV 333.04 nm f=0.1049 <S**2>=0.000

370 -> 374 -0.14281

370 -> 375 -0.17729

371 -> 376 -0.16087

371 -> 377 -0.26085

372 -> 376 -0.18560

373 -> 374 -0.21916

373 -> 375 0.25758

373 -> 377 0.21832

373 -> 378 0.15742

373 -> 384 -0.10288

Excited State 4: Singlet-A 3.7334 eV 332.09 nm f=0.0112 <S**2>=0.000

370 -> 374 0.16823

370 -> 375 0.14484

371 -> 374 0.16407

371 -> 375 -0.18000

371 -> 378 -0.10662

372 -> 376 0.18842

372 -> 377 -0.12597

373 -> 374 -0.12711

373 -> 375 0.20628

373 -> 376 0.14377

373 -> 377 0.30104

373 -> 378 0.11989

373 -> 385 0.10511

Excited State 5: Singlet-A 4.2922 eV 288.86 nm f=0.2529 <S**2>=0.000

370 -> 379 -0.13220

370 -> 380 0.10873

370 -> 381 -0.14689

371 -> 379 0.10336

371 -> 380 0.11647

371 -> 381 -0.11635
372 -> 378 -0.24845
372 -> 380 -0.12845
372 -> 381 0.36002
372 -> 382 -0.19345
372 -> 383 -0.12422

Excited State 6: Singlet-A 4.2941 eV 288.73 nm f=0.2803 <S**2>=0.000
370 -> 379 0.43259
370 -> 381 0.10212
370 -> 389 -0.12673
371 -> 380 0.26404
371 -> 382 -0.14289
372 -> 379 0.10266
373 -> 380 0.11633
373 -> 381 0.12783

Excited State 7: Singlet-A 4.2959 eV 288.61 nm f=0.2330 <S**2>=0.000
370 -> 380 0.10876
371 -> 379 0.10470
371 -> 380 0.31220
371 -> 381 0.17449
371 -> 390 0.10500
372 -> 380 0.16333
373 -> 378 -0.18762
373 -> 379 0.14698
373 -> 381 -0.22047
373 -> 382 -0.23549

Excited State 8: Singlet-A 4.3039 eV 288.07 nm f=0.0396 <S**2>=0.000
370 -> 379 -0.21190

370 -> 382 -0.12122
371 -> 379 0.16688
371 -> 380 0.13347
371 -> 382 -0.14188
372 -> 380 0.13591
372 -> 381 -0.17665
373 -> 378 0.15804
373 -> 380 0.15752
373 -> 381 0.25529
373 -> 382 0.15236

Excited State 9: Singlet-A 4.4895 eV 276.17 nm f=0.2727 <S**2>=0.000
370 -> 385 -0.10053
371 -> 383 -0.12534
371 -> 387 0.10888
372 -> 383 0.36343
372 -> 385 0.17257
372 -> 387 -0.24909
372 -> 388 0.19973
373 -> 384 -0.11957
373 -> 385 0.14833
373 -> 388 -0.10141

Excited State 10: Singlet-A 4.4913 eV 276.05 nm f=0.2420 <S**2>=0.000
370 -> 386 -0.10381
371 -> 383 -0.14271
371 -> 385 0.18984
371 -> 386 -0.16326
371 -> 390 0.14270
372 -> 385 0.13679
373 -> 383 0.10718

373 -> 384	0.25385
373 -> 385	-0.18726
373 -> 386	-0.16654
373 -> 388	0.22465
373 -> 389	-0.11365

6.16.3 Computed emission energies of compound 5q (cam-B3LYP/def2svp SMD CH_2Cl_2 GD3BJ)

Excitation energies and oscillator strengths:

Excited State 1: Singlet-A 2.7303 eV 454.11 nm f=0.0535 <S**2>=0.000

373 -> 374 0.63373

373 -> 375 0.11021

373 -> 381 -0.15010

373 -> 382 0.14082

This state for optimization and/or second-order correction.

Total Energy, E(TD-HF/TD-DFT) = -6933.10028830

Copying the excited state density for this state as the 1-particle RhoCI density.

Excited State 2: Singlet-A 3.7580 eV 329.92 nm f=0.1030 <S**2>=0.000

370 -> 376 -0.12146

370 -> 377 -0.28074

371 -> 375 0.38064

371 -> 377 0.25446

371 -> 378 -0.14925

371 -> 384 -0.15514

372 -> 375 -0.10240

372 -> 376 -0.13679

Excited State 3: Singlet-A 3.7600 eV 329.75 nm f=0.0708 <S**2>=0.000

370 -> 375 0.14402

370 -> 376 -0.26960

370 -> 377 -0.20123

370 -> 378 -0.10486

370 -> 383 0.12250

371 -> 376 -0.11404

371 -> 377 -0.13781

372 -> 375 0.18231

372 -> 376 0.31098

372 -> 377 -0.23091

372 -> 379 -0.12689

372 -> 383 -0.10301

372 -> 385 -0.10426

Excited State 4: Singlet-A 3.7671 eV 329.12 nm f=0.0738 <S**2>=0.000

370 -> 375 -0.22751

370 -> 376 0.21663

370 -> 377 0.11926

370 -> 378 0.13592

371 -> 375 0.12065

371 -> 376 0.12224

371 -> 377 0.25310

372 -> 375 0.12863

372 -> 376 0.26473

372 -> 377 -0.19622

372 -> 379 -0.11205

373 -> 382 -0.11076

Excited State 5: Singlet-A 3.8050 eV 325.85 nm f=0.2260 <S**2>=0.000

369 -> 374 -0.17721

373 -> 374 0.13171

373 -> 379 -0.10641

373 -> 381 0.27158

373 -> 382 -0.47481

373 -> 388 -0.13310

373 -> 389 -0.19471

373 -> 393 0.13369

Excited State 6: Singlet-A 4.1955 eV 295.52 nm f=0.1196 <S**2>=0.000

373 -> 388 0.13090

373 -> 389 0.26719

373 -> 390 -0.26604

373 -> 391 0.19063

373 -> 393 0.47751

external emission: -6933.153068 - -6933.250538 = 0.097470 = 2.652307 eV 467.46 nm

6.17 Quantum chemical calculation data of tetrakis(4-(10-hexyl-10*H*-phenothiazin-1-yl)phenyl)silane (5r)

6.17.1 Computed xyz-coordinates of compound 5r (cam-B3LYP/def2svp SMD CH_2Cl_2 GD3BJ)

0 1

C -2.14703 -9.37545 -0.30344

C -1.44792 -8.22035 -0.64550

C -2.09834 -6.99027 -0.69675

C -3.46943 -6.90153 -0.43311

C -4.16983 -8.07483 -0.11259

C -3.51009 -9.29831 -0.02652

S -5.93320 -7.97872 0.06841

N -4.15273 -5.67544 -0.48036

C -6.03564 -6.39747 0.86844

C -5.11854 -5.39677 0.50578

C -5.18066 -4.15186 1.16763

C -6.23255 -3.90828 2.05914

C -7.16134 -4.89339 2.37171

C -7.04042 -6.15624 1.80072

C -3.86439 -4.73328 -1.55821

C -4.11705 -5.33498 -2.93201

C -4.12921 -3.11489 0.99120

C -4.46073 -1.78893 0.69747

C -3.46851 -0.82382 0.54720

C -2.11321 -1.14540 0.69185

C -1.79212 -2.47318 1.01544

C -2.77766 -3.44187 1.15826

Si -0.74086 0.11009 0.40989

C -8.26368 5.30677 -2.61168

C -7.66446 4.08011 -2.33546

C -6.72118 3.96844 -1.31740

C -6.38218 5.08048 -0.53916

C -7.00816 6.30692 -0.81075

C -7.92513 6.42452 -1.85236

S -6.68657 7.67373 0.27525

N -5.43748 4.99606 0.49687

C -4.96390 7.37418 0.58154

C -4.51333 6.04573 0.66173

C -3.13847 5.81088 0.87698

C -2.29571 6.89881 1.13546

C -2.76590 8.20578 1.09753

C -4.09965 8.44623 0.78355

C -5.45728 3.84395 1.39432

C -6.78949 3.69654 2.11321

C -2.54940 4.44859 0.78134

C -2.78273 3.65289 -0.34769

C -2.21986 2.38731 -0.44965

C -1.41126 1.85997 0.57091

C -1.16535 2.67243 1.68409

C -1.72082 3.94543 1.78787

C 7.17431 3.14517 -3.43320

C 6.28243 2.96812 -2.37779

C 5.38057 1.90724 -2.38728

C 5.37560 0.98952 -3.44304

C 6.28744 1.16810 -4.49482

C 7.16667 2.24839 -4.49949

S 6.36565 -0.07445 -5.76119

N 4.48371 -0.09443 -3.47304

C 4.63639 -0.46438 -5.86640

C 3.86560 -0.43101 -4.69315

C 2.48323 -0.70220 -4.78394

C 1.95178 -1.13082 -6.00496

C 2.73915 -1.20658 -7.14830

C 4.07895 -0.83642 -7.08674

C 4.26417 -0.87166 -2.25615

C 5.54321 -1.51621 -1.74456

C 1.58583 -0.49065 -3.61784

C 1.58294 0.74436 -2.95736

C 0.80639 0.93638 -1.82329

C 0.01091 -0.09537 -1.30084

C -0.02584 -1.30640 -2.00125

C 0.74403 -1.50141 -3.14641

C 8.91333 0.58116 1.10314

C 7.72315 0.97838 0.49877

C 6.54136 1.03266 1.23392

C 6.53792 0.71800 2.59735

C 7.74823 0.34428 3.20200

C 8.92146 0.25395 2.45699

S 7.76985 0.10202 4.95984

N 5.36300 0.76867 3.36655

C 6.18004 -0.66480 5.14492

C 5.12564 -0.24440 4.31649

C 3.87012 -0.87731 4.44929

C 3.68339 -1.80327 5.48352

C 4.72537 -2.17511 6.32367

C 5.98979 -1.62864 6.13058

C 4.43919 1.88608 3.19055

C 5.10037 3.22811 3.46441

C 2.75896 -0.63750 3.49070

C 2.98900 -0.68873 2.11181

C 1.95238 -0.47661 1.21095

C 0.64549 -0.21226 1.64496

C 0.41667 -0.19941 3.02895

C 1.45253 -0.40252 3.93570

H -1.63460 -10.33808 -0.25245

H -0.37908 -8.27137 -0.86422

H -1.54032 -6.08787 -0.95018

H -4.07311 -10.19569 0.23949

H -6.28609 -2.93556 2.55203

H -7.96146 -4.68903 3.08564

H -7.73345 -6.95743 2.06600

H -4.51968 -3.86705 -1.41648

H -2.83346 -4.34963 -1.48860

H -3.44836 -6.18397 -3.13602

H -5.15499 -5.69150 -3.01309

H -3.94516 -4.57790 -3.71224

H -5.50951 -1.51279 0.56689

H -3.76508 0.20015 0.30878

H -0.74782 -2.76145 1.16300

H -2.49827 -4.46745 1.40687

H -8.99568 5.39679 -3.41665

H -7.92353 3.19848 -2.92577

H -6.24460 3.00782 -1.11763

H -8.38921 7.39256 -2.05392

H -1.23687 6.70806 1.32108

H -2.08676 9.04051 1.28097

H -4.47945 9.46725 0.70683

H -5.20356 2.91494 0.85804

H -4.66217 3.99355 2.13264

H -6.74534 2.85321 2.81912

H -7.02885 4.61150 2.67584

H -7.61368 3.50300 1.41117

H -3.41001 4.03438 -1.15552

H -2.41420 1.79998 -1.35146

H -0.52937 2.31249 2.49557

H -1.52203 4.55139 2.67469

H 7.87607 3.98144 -3.43033

H 6.28029 3.66660 -1.53822

H 4.67424 1.78282 -1.56538

H 7.86005 2.37450 -5.33401

H 0.88588 -1.36132 -6.06065

H 2.30214 -1.52321 -8.09724

H 4.70190 -0.84717 -7.98365

H 3.80424 -0.25166 -1.46976

H 3.53312 -1.65203 -2.49201

H 5.33093 -2.11898 -0.84823

H 5.97975 -2.17288 -2.51217

H 6.29728 -0.76539 -1.46916

H 2.21394 1.55432 -3.32805

H 0.84308 1.90574 -1.31851

H -0.64341 -2.13049 -1.63532

H 0.72438 -2.46711 -3.65681

H 9.83680 0.52775 0.52345

H 7.70568 1.24066 -0.56062

H 5.61148 1.32915 0.74695

H 9.84741 -0.05416 2.94769

H 2.70575 -2.27663 5.59236

H 4.56176 -2.91508 7.10937

H 6.83270 -1.93770 6.75237

H 3.98819 1.87642 2.18487

H 3.61234 1.73816 3.89400

H 4.36073 4.03826 3.37467

H 5.52325 3.25114 4.48009

H 5.91269 3.43566 2.75255

H 3.99429 -0.89468 1.74033

H 2.17302 -0.52027 0.14306

H -0.59042 -0.02207 3.41672

H 1.24786 -0.36440 5.00789

SCF Done: E(RCAM-B3LYP) = -5183.91581811 A.U. after 1 cycles

Zero-point correction= 1.237956 (Hartree/Particle)

Thermal correction to Energy= 1.312919

Thermal correction to Enthalpy= 1.313864

Thermal correction to Gibbs Free Energy= 1.115706

Sum of electronic and zero-point Energies= -5182.677862

Sum of electronic and thermal Energies= -5182.602899

Sum of electronic and thermal Enthalpies= -5182.601954

Sum of electronic and thermal Free Energies= -5182.800112

6.17.2 Computed excitation energies of compound 5r (cam-B3LYP/def2svp SMD CH_2Cl_2 GD3BJ)

Excitation energies and oscillator strengths:

Excited State 1: Singlet-A 4.1266 eV 300.45 nm f=0.1095 <S**2>=0.000

322 -> 326 -0.17836

322 -> 327 -0.11342

322 -> 328 0.13819

322 -> 334 -0.12191

323 -> 327 -0.11022

325 -> 326 0.31268

325 -> 327 0.23648

325 -> 328 -0.17552

325 -> 329 0.12916

325 -> 331 -0.19808

325 -> 334 0.17598

This state for optimization and/or second-order correction.

Total Energy, E(TD-HF/TD-DFT) = -5183.76416755

Copying the excited state density for this state as the 1-particle RhoCI density.

Excited State 2: Singlet-A 4.1586 eV 298.14 nm f=0.1111 <S**2>=0.000

323 -> 327 -0.14650

323 -> 329 0.23454

323 -> 332 0.20510

323 -> 333 0.10993

324 -> 326 0.24600

324 -> 327 -0.23456

324 -> 328 0.15165

324 -> 329 0.12201

324 -> 333 0.31353

325 -> 327 -0.13079

325 -> 329 0.11150

325 -> 333 0.11398

Excited State 3: Singlet-A 4.1643 eV 297.73 nm f=0.0947 <S**2>=0.000

323 -> 326 0.26101

323 -> 328 0.21383

323 -> 329 -0.15492

323 -> 332 -0.24023

323 -> 333 0.20637

324 -> 327 -0.10574

324 -> 329 0.25618

324 -> 332 0.26286

Excited State 4: Singlet-A 4.1799 eV 296.62 nm f=0.0577 <S**2>=0.000

322 -> 327 -0.24430

322 -> 328 -0.27265

322 -> 329 -0.19092

322 -> 331 0.27318

322 -> 334 0.29298

325 -> 328 -0.15983

325 -> 334 0.16788

Excited State 5: Singlet-A 4.2793 eV 289.73 nm f=0.0244 <S**2>=0.000

322 -> 326 -0.10929

322 -> 331 -0.16421

322 -> 334 0.20470

323 -> 331 -0.14255

323 -> 334 0.10390

325 -> 326 0.18557

325 -> 327 0.13219

325 -> 328 -0.10868

325 -> 331 0.36500

325 -> 334 -0.27041

Excited State 6: Singlet-A 4.2988 eV 288.42 nm f=0.0557 <S**2>=0.000

323 -> 329 -0.21259

323 -> 332 0.35526

324 -> 326 -0.19591

324 -> 327 0.12243

324 -> 328 -0.13609

324 -> 333 0.33988

325 -> 332 0.12359

Excited State 7: Singlet-A 4.3011 eV 288.26 nm f=0.0531 <S**2>=0.000

323 -> 326 -0.18710

323 -> 328 -0.14796

323 -> 332 -0.17046

323 -> 333 0.26469

324 -> 327 0.14178

324 -> 329 -0.22284

324 -> 332 0.29612

324 -> 333 0.19377

325 -> 333 0.14867

Excited State 8: Singlet-A 4.3091 eV 287.73 nm f=0.0296 <S**2>=0.000

322 -> 327 0.22014

322 -> 328 0.23869

322 -> 329 0.15590

322 -> 330 0.18150

322 -> 331 0.25308

322 -> 334 0.28193

325 -> 328 0.15440

325 -> 334 0.17097

Excited State 9: Singlet-A 4.5909 eV 270.07 nm f=0.0715 <S**2>=0.000

322 -> 330 0.10141

322 -> 331 -0.15015

322 -> 335 0.35752

322 -> 336 -0.26959

322 -> 338 0.19275

322 -> 341 0.12936

323 -> 338 -0.10666

325 -> 335 0.14082

325 -> 338 0.24459

Excited State 10: Singlet-A 4.6118 eV 268.84 nm f=0.0967 <S**2>=0.000

322 -> 331 0.10426

322 -> 335 -0.12393

322 -> 336 -0.12070

322 -> 338 -0.26068

323 -> 336 -0.13939

323 -> 338 -0.10334

324 -> 336 -0.10344

325 -> 335 -0.11995

325 -> 336 0.39804

325 -> 337 -0.12540

325 -> 338 0.17675

6.17.3 Computed emission energies of compound 5r (cam-B3LYP/def2svp SMD CH_2Cl_2 GD3BJ)

Excitation energies and oscillator strengths:

Excited State 1: Singlet-A 2.6448 eV 468.78 nm f=0.0278 <S**2>=0.000

325 -> 326 0.59526

325 -> 327 -0.11697

325 -> 330 -0.32983

This state for optimization and/or second-order correction.

Total Energy, E(TD-HF/TD-DFT) = -5183.74371789

Copying the excited state density for this state as the 1-particle RhoCI density.

Excited State 2: Singlet-A 3.4649 eV 357.83 nm f=0.0817 <S**2>=0.000

325 -> 326 0.32202

325 -> 330 0.57885

325 -> 348 -0.12488

Excited State 3: Singlet-A 4.1702 eV 297.31 nm f=0.0916 <S**2>=0.000

322 -> 327 -0.17370

322 -> 328 0.13988

322 -> 329 -0.21306

322 -> 332 0.19279

322 -> 333 0.15847

323 -> 327 0.29903

323 -> 328 0.20716

323 -> 329 -0.15135

323 -> 332 0.24960

323 -> 333 -0.22398

Excited State 4: Singlet-A 4.1725 eV 297.14 nm f=0.0760 <S**2>=0.000

322 -> 327 0.18919

322 -> 329 0.10449

322 -> 333 -0.16658

323 -> 328 0.11603

323 -> 329 -0.11378

323 -> 332 0.14948

324 -> 328 0.34690

324 -> 329 0.26562

324 -> 334 0.30648

Excited State 5: Singlet-A 4.1741 eV 297.03 nm f=0.0446 <S**2>=0.000

321 -> 326 -0.17659

322 -> 327 -0.28428

322 -> 329 -0.16186

322 -> 332 0.11035

322 -> 333 0.25384

323 -> 328 -0.14839

323 -> 329 0.14848

323 -> 332 -0.19494

324 -> 328 0.21829

324 -> 329 0.17092

324 -> 334 0.19577

Excited State 6: Singlet-A 4.2076 eV 294.66 nm f=0.0961 <S**2>=0.000

321 -> 326 -0.35949

321 -> 330 0.14686

325 -> 331 0.11096

325 -> 337 0.14523

325 -> 343 0.36825

325 -> 344 0.28146

corrected external emission excitation: -5183.792370 - -5183.887906 = 0.095536 = 2.599666 eV 476.92 nm

6.18 Quantum chemical calculation data of tetrakis(10-hexyl-10*H*-phenothiazin-3-yl)silane (6)

6.18.1 Computed xyz-coordinates of compound 6 (cam-B3LYP/def2svp SMD CH_2Cl_2 GD3BJ)

0 1

C -3.03125 7.34264 -3.35615

C -1.77070 7.90169 -3.52260

C -0.63085 7.10129 -3.51192

C -0.71532 5.70893 -3.35905

C -2.00351 5.15470 -3.22808

C -3.13650 5.96319 -3.20204

S -2.22592 3.40051 -3.23959

N 0.43575 4.89835 -3.34778

C -0.71180 2.92010 -2.46175

C 0.46338 3.66974 -2.67169

C 1.65916 3.11940 -2.18616

C 1.67654 1.90520 -1.50545

C 0.50680 1.18577 -1.25066

C -0.68680 1.72758 -1.74909

Si 0.48667 -0.40290 -0.24918

C 1.70393 5.48490 -3.77538

C 2.47137 6.27452 -2.71852

C -5.02958 3.11816 6.04441

C -4.70847 2.11101 6.94530

C -3.59651 1.29840 6.73689

C -2.75247 1.48200 5.63094

C -3.07225 2.53080 4.74672

C -4.20553 3.31590 4.94000

S -1.95911 2.97225 3.44558

N -1.62807 0.66160 5.41835

C -1.28630 1.37374 3.09676

C -1.11596 0.43460 4.13236

C -0.39604 -0.72933 3.81820

C 0.10197 -0.94979 2.53839

C -0.11258 -0.04312 1.49478

C -0.82077 1.12242 1.81074

C -1.18407 -0.20613 6.50694

C -1.90553 -1.54465 6.63878

C -6.94187 -5.57541 -1.17172

C -6.79407 -5.61016 -2.55231

C -5.59857 -5.21729 -3.14936

C -4.50030 -4.79891 -2.38275

C -4.65483 -4.80485 -0.98279

C -5.86415 -5.16275 -0.39322

S -3.26693 -4.49819 0.06936

N -3.29176 -4.40064 -2.98511

C -2.37698 -3.36657 -0.95855

C -2.42165 -3.49494 -2.36018

C -1.54815 -2.67987 -3.09817

C -0.69595 -1.77713 -2.47101

C -0.67825 -1.61976 -1.08099

C -1.54812 -2.43327 -0.34534

C -3.09121 -4.69444 -4.40238

C -3.70068 -3.70442 -5.39150

C 9.43359 -0.14360 1.21191

C 9.64667 -1.23445 0.37889

C 8.58571 -2.04675 -0.01421

C 7.27709 -1.80951 0.43339

C 7.08463 -0.72097 1.30629

C 8.14239 0.10963 1.66626

S 5.51769 -0.46908 2.08636

N 6.20358 -2.62963 0.03750

C 4.46326 -1.08110 0.80479

C 4.88862 -2.14443 -0.01713

C 3.92950 -2.70203 -0.87595

C 2.62767 -2.21154 -0.92764

C 2.21649 -1.12749 -0.14894

C 3.17161 -0.57621 0.71729

C 6.50233 -3.90543 -0.60943

C 6.77150 -3.85589 -2.11108

H -3.92665 7.96677 -3.35031

H -1.65754 8.98019 -3.65195

H 0.33652 7.58314 -3.62578

H -4.11572 5.49436 -3.08087

H 2.60308 3.63768 -2.33336

H 2.64044 1.52622 -1.15809

H -1.63275 1.19792 -1.60567

H 2.33003 4.66703 -4.15697

H 1.49226 6.12695 -4.64107

H 3.40478 6.65913 -3.15698

H 1.89755 7.13244 -2.34320

H 2.73733 5.65570 -1.85092

H -5.90709 3.74922 6.19617

H -5.33297 1.93604 7.82397

H -3.39474 0.51027 7.45743

H -4.42658 4.10816 4.22103

H -0.21291 -1.48513 4.57742

H 0.66649 -1.86878 2.35995

H -0.99980 1.88074 1.04459

H -0.10692 -0.37460 6.37295

H -1.28122 0.36416 7.44064

H -1.48493 -2.10346 7.48864

H -2.98207 -1.42018 6.81754

H -1.79478 -2.16606 5.73995

H -7.88091 -5.86931 -0.69934

H -7.62033 -5.93520 -3.18820

H -5.53336 -5.24074 -4.23386

H -5.94834 -5.13682 0.69565

H -1.52658 -2.73671 -4.18332

H -0.04186 -1.16839 -3.10088

H -1.57759 -2.36012 0.74463

H -2.00831 -4.76968 -4.57056

H -3.49197 -5.70050 -4.58681

H -4.79192 -3.63363 -5.28989

H -3.29593 -2.69092 -5.26714

H -3.47849 -4.03213 -6.41847

H 10.25943 0.50365 1.51250

H 10.64989 -1.46126 0.01178

H 8.79482 -2.87753 -0.68288

H 7.94738 0.95330 2.33232

H 4.18863 -3.53707 -1.52150

H 1.92362 -2.69496 -1.60886

H 2.90265 0.25706 1.37258

H 7.36624 -4.34167 -0.08971

H 5.66039 -4.58058 -0.40449

H 6.96857 -4.87425 -2.47919

H 5.91712 -3.45492 -2.67289

H 7.64351 -3.23514 -2.35728

SCF Done: E(RCAM-B3LYP) = -4260.81875283 A.U. after 1 cycles

Zero-point correction= 0.913031 (Hartree/Particle)

Thermal correction to Energy= 0.969069

Thermal correction to Enthalpy= 0.970013

Thermal correction to Gibbs Free Energy= 0.813517

Sum of electronic and zero-point Energies= -4259.905722

Sum of electronic and thermal Energies= -4259.849684

Sum of electronic and thermal Enthalpies= -4259.848740

Sum of electronic and thermal Free Energies= -4260.005236

6.18.2 Computed excitation energies of compound 6 (cam-B3LYP/def2svp SMD CH_2Cl_2 GD3BJ)

Excitation energies and oscillator strengths:

Excited State 1: Singlet-A 3.7807 eV 327.94 nm f=0.0597 <S**2>=0.000

242 -> 249 -0.18074

243 -> 246 0.25829

243 -> 248 -0.12829

244 -> 249 0.20654

245 -> 246 0.35678

245 -> 247 0.18619

245 -> 248 0.11637

245 -> 249 0.28030

245 -> 250 -0.13120

This state for optimization and/or second-order correction.

Total Energy, E(TD-HF/TD-DFT) = -4260.67981305

Copying the excited state density for this state as the 1-particle RhoCI density.

Excited State 2: Singlet-A 3.7958 eV 326.63 nm f=0.1024 <S**2>=0.000

242 -> 246 -0.12937

242 -> 247 -0.18650

242 -> 248 0.13644

243 -> 246 -0.32274

243 -> 248 0.12621

243 -> 249 0.33436

243 -> 250 0.10731

244 -> 246 0.20010

244 -> 248 -0.17538

245 -> 246 0.14458

245 -> 248 0.13639

245 -> 249 0.10912

Excited State 3: Singlet-A 3.8129 eV 325.17 nm f=0.1072 <S**2>=0.000

242 -> 247 0.27329

242 -> 248 0.25671

242 -> 249 -0.16036

243 -> 248 -0.18952

244 -> 246 -0.12743

244 -> 247 0.33849

244 -> 248 -0.21280

245 -> 248 0.22927

245 -> 249 -0.11574

Excited State 4: Singlet-A 3.8403 eV 322.85 nm f=0.0085 <S**2>=0.000

242 -> 246 -0.17898

242 -> 247 0.29833

242 -> 248 -0.12309

242 -> 250 0.12105

243 -> 248 0.24443

244 -> 247 0.28566

244 -> 248 0.21496

244 -> 249 -0.17551

245 -> 248 -0.17167

245 -> 249 0.18133

Excited State 5: Singlet-A 4.3661 eV 283.97 nm f=0.2689 <S**2>=0.000

242 -> 251 -0.10816

242 -> 257 0.26733

243 -> 246 0.10387

243 -> 250 0.10450

243 -> 257 -0.12977

244 -> 246 0.27434

244 -> 250 0.30507

244 -> 254 0.10954

244 -> 256 -0.16670

245 -> 246 -0.12854

245 -> 250 -0.12285

245 -> 257 0.14281

Excited State 6: Singlet-A 4.4008 eV 281.73 nm f=0.1088 <S**2>=0.000

242 -> 246 0.25957

242 -> 250 0.29731

242 -> 254 0.10360

242 -> 256 -0.13173

243 -> 257 0.12175

244 -> 251 -0.16545

244 -> 255 -0.10157

244 -> 257 0.27216

245 -> 246 0.10209

245 -> 250 0.10800

245 -> 257 -0.11517

Excited State 7: Singlet-A 4.4273 eV 280.05 nm f=0.1109 <S**2>=0.000

241 -> 246 -0.10551

242 -> 252 -0.11566

243 -> 250 0.21724

243 -> 251 0.12375

243 -> 255 0.11627

243 -> 256 0.20293

244 -> 251 -0.11270

244 -> 252 0.12417

245 -> 250 0.21763

245 -> 251 -0.12782

245 -> 252 0.20788

245 -> 256 0.25894

Excited State 8: Singlet-A 4.4377 eV 279.39 nm f=0.0919 <S**2>=0.000

242 -> 250 0.11388

242 -> 256 0.12246

243 -> 249 0.10623

243 -> 250 0.18520

243 -> 251 0.25363

243 -> 252 -0.19842

243 -> 255 0.16512

243 -> 256 0.12664

244 -> 250 -0.15672

244 -> 256 -0.13222

245 -> 250 -0.16143

245 -> 251 0.10117

245 -> 252 -0.19163

245 -> 256 -0.15027

Excited State 9: Singlet-A 4.4947 eV 275.85 nm f=0.1794 <S**2>=0.000

242 -> 253 0.10399

242 -> 255 -0.14967

243 -> 254 -0.20674

244 -> 252 0.16691

244 -> 255 0.12380

245 -> 252 0.23444

245 -> 254 -0.19222

245 -> 255 0.34503

245 -> 256 -0.17740

Excited State 10: Singlet-A 4.4995 eV 275.55 nm f=0.2847 <S**2>=0.000

242 -> 252 -0.19233

242 -> 254 -0.24067

243 -> 253	0.29291
243 -> 254	-0.23284
243 -> 255	-0.15355
243 -> 256	-0.10818
244 -> 251	-0.23773
244 -> 252	-0.13393
244 -> 255	0.13198

6.18.3 Computed emission energies of compound 6 (cam-B3LYP/def2svp SMD CH_2Cl_2 GD3BJ)

Excitation energies and oscillator strengths:

Excited State 1: Singlet-A 2.8141 eV 440.58 nm f=0.0142 <S**2>=0.000

245 -> 246 0.65779

245 -> 247 -0.16897

245 -> 249 0.13821

This state for optimization and/or second-order correction.

Total Energy, E(TD-HF/TD-DFT) = -4260.66340207

Copying the excited state density for this state as the 1-particle RhoCI density.

Excited State 2: Singlet-A 3.8019 eV 326.11 nm f=0.0790 <S**2>=0.000

242 -> 246 -0.10643

242 -> 248 0.21869

242 -> 249 0.25116

243 -> 246 -0.20361

243 -> 247 -0.36885

243 -> 248 0.20281

243 -> 249 0.22602

243 -> 250 0.13250

244 -> 247 0.19801

Excited State 3: Singlet-A 3.8218 eV 324.41 nm f=0.0680 <S**2>=0.000

242 -> 247 0.33034

242 -> 249 0.28031

243 -> 249 -0.26557

244 -> 247 0.23449

244 -> 248 0.36180

Excited State 4: Singlet-A 3.8422 eV 322.69 nm f=0.0255 <S**2>=0.000

242 -> 247 -0.23925

242 -> 248 -0.21898

242 -> 250 0.10622

243 -> 248 0.31158

244 -> 248 0.28359

244 -> 249 -0.37788

Excited State 5: Singlet-A 3.9917 eV 310.60 nm f=0.1900 <S**2>=0.000

241 -> 246 0.15262

245 -> 250 -0.20076

245 -> 251 -0.20579

245 -> 252 -0.10859

245 -> 253 -0.16621

245 -> 254 0.10042

245 -> 256 -0.53400

Excited State 6: Singlet-A 4.1545 eV 298.44 nm f=0.0307 <S**2>=0.000

230 -> 246 -0.12733

245 -> 250 0.11413

245 -> 255 -0.13918

245 -> 256 -0.11885

245 -> 257 0.61564

corrected external emission excitation: -4260.699869 - -4260.802089 = 0.102220 = 2.781544 eV 445.74 nm

6.19 Quantum chemical calculation data of tetrakis(5-(10-hexyl-10*H*-phenothiazin-3-yl)thiophen-2-yl)silane (7)

6.19.1 Computed xyz-coordinates of compound 7 (cam-B3LYP/def2svp SMD CH_2Cl_2 GD3BJ)

0 1

Si -0.05897 0.39035 -1.09572

C -0.84490 -0.22075 0.47833

C 0.87533 1.95314 -0.70745

C 1.07808 -0.91453 -1.78403

C -1.37056 0.73452 -2.36899

S 2.77141 -0.94583 -1.42837

C 3.02013 -2.42596 -2.29589

C 1.83557 -2.86084 -2.84401

C 0.74033 -2.00452 -2.55365

C -1.24077 0.80110 -3.73758

C -2.44397 1.14640 -4.40895

C -3.50122 1.34520 -3.55100

S -3.00123 1.09032 -1.91157

S -1.12737 -1.90420 0.76209

C -1.93509 -1.60879 2.26701

C -1.96432 -0.25943 2.53502

C -1.34750 0.52207 1.52225

C 0.91660 3.11023 -1.44967

C 1.76943 4.10874 -0.90734

C 2.38657 3.71681 0.25775

S 1.90569 2.10465 0.67748

C 8.63378 -8.69567 -4.52693

C 9.53653 -8.25618 -3.56712

C 9.38157 -7.01111 -2.96250

C 8.33002 -6.15200 -3.31610

C 7.44574 -6.59734 -4.31768

C 7.58354 -7.85801 -4.89189

S 6.22437 -5.50378 -4.98191

N 8.17086 -4.89411 -2.70612

C 5.88868 -4.54244 -3.53580

C 6.91523 -4.27807 -2.60661

C 6.62164 -3.35610 -1.59078

C 5.37576 -2.74884 -1.49429

C 4.34971 -3.04145 -2.39560

C 4.63626 -3.95676 -3.41709

C 9.27534 -4.35767 -1.91382

C 3.32605 4.47708 1.09170

C 3.55976 4.15893 2.43142

C 4.43157 4.90639 3.21339

C 5.13150 6.00470 2.69188

C 4.92521 6.29559 1.32799

C 4.03019 5.56517 0.55929

S 5.93176 7.50148 0.51516

N 6.00533 6.76709 3.47974

C 6.19172 8.60967 1.86914

C 6.27699 8.11629 3.18541

C 6.65479 9.02443 4.18624

C 6.92268 10.35906 3.89174

C 6.79888 10.83766 2.59364

C 6.42446 9.95275 1.58641

C 6.40005 6.24503 4.78631

C 5.42612 6.49394 5.93471

C -2.46233 -2.71105 3.08172

C -2.76329 -3.96128 2.53659

C -3.28617 -4.98623 3.31535

C -3.52053 -4.82152 4.68879

C -3.17711 -3.57152 5.24256

C -2.68487 -2.54077 4.45451

S -3.20734 -3.33798 6.99544

N -4.05370 -5.85089 5.47755

C -4.55514 -4.40956 7.39979

C -4.78252 -5.58014 6.65053

C -5.75746 -6.46792 7.13061

C -6.47694 -6.19721 8.29204

C -6.26529 -5.02075 8.99959

C -5.30293 -4.12643 8.53933

C -4.10973 -7.19943 4.91762

C -5.30600 -7.50820 4.02179

C -11.88093 3.00201 -1.83592

C -12.23127 2.65430 -3.13424

C -11.25948 2.53218 -4.12447

C -9.90481 2.77849 -3.85437

C -9.57180 3.16596 -2.54199

C -10.54197 3.25005 -1.54716

S -7.93042 3.70212 -2.15800

N -8.92072 2.65531 -4.85314

C -7.03074 2.68395 -3.28986

C -7.58918 2.34836 -4.53905

C -6.74196 1.70492 -5.45493

C -5.42588 1.39359 -5.13874

C -4.88879 1.69275 -3.88398

C -5.72463 2.34151 -2.96651

C -9.34972 2.55195 -6.24631

C -9.74628 1.15972 -6.73014

C 9.36361 -4.83217 -0.46595

H 1.74907 -3.77755 -3.42786

H -0.27739 -2.19226 -2.90044

H -0.29791 0.60927 -4.25290

H -2.52587 1.26045 -5.49031

H -2.42964 0.16237 3.42638

H -1.28211 1.61085 1.56067

H 0.33797 3.24542 -2.36507

H 1.90564 5.09540 -1.35106

H 8.74331 -9.67596 -4.99415

H 10.37411 -8.88904 -3.26610

H 10.09960 -6.71500 -2.20244

H 6.86636 -8.17281 -5.65346

H 7.36962 -3.10140 -0.84488

H 5.20395 -2.04879 -0.67404

H 3.87763 -4.19306 -4.16517

H 10.20576 -4.60265 -2.44380

H 9.19535 -3.26241 -1.94207

H 3.03365 3.32371 2.89841

H 4.55180 4.62009 4.25465

H 3.90869 5.82991 -0.49245

H 6.74515 8.69738 5.21881

H 7.22070 11.02891 4.70113

H 6.99487 11.88567 2.36016

H 6.33140 10.29899 0.55446

H 6.57147 5.16617 4.66916

H 7.38032 6.67773 5.02816

H 4.44498 6.03430 5.75455

H 5.83585 6.06110 6.85997

H 5.25645 7.56470 6.11048

H -2.61579 -4.14538 1.47036

H -3.51943 -5.92729 2.82461

H -2.43271 -1.59327 4.93383

H -5.96958 -7.39086 6.59731

H -7.22107 -6.92008 8.63315

H -6.83578 -4.79786 9.90299

H -5.10644 -3.19924 9.08268

H -3.17583 -7.36158 4.36235

H -4.08217 -7.90417 5.75980

H -5.34314 -6.85500 3.13960

H -5.23764 -8.54818 3.66807

H -6.26137 -7.39476 4.55147

H -12.63695 3.08612 -1.05321

H -13.27477 2.46014 -3.39127

H -11.57553 2.23853 -5.12199

H -10.23957 3.53730 -0.53742

H -7.10815 1.42310 -6.43840

H -4.81700 0.87783 -5.88295

H -5.34238 2.62632 -1.98356

H -10.18933 3.24710 -6.38203

H -8.53494 2.94529 -6.86938

H -10.58975 0.74676 -6.16069

H -10.04801 1.21433 -7.78720

H -8.91903 0.44112 -6.65378

H 9.49135 -5.92049 -0.39134

H 10.22946 -4.35728 0.02001

H 8.46792 -4.56919 0.11274

SCF Done: E(RCAM-B3LYP) = -6466.92100416 A.U. after 1 cycles

Zero-point correction= 1.106443 (Hartree/Particle)

Thermal correction to Energy= 1.180054

Thermal correction to Enthalpy= 1.180998

Thermal correction to Gibbs Free Energy= 0.980144

Sum of electronic and zero-point Energies= -6465.814561

Sum of electronic and thermal Energies= -6465.740950

Sum of electronic and thermal Enthalpies= -6465.740006

Sum of electronic and thermal Free Energies= -6465.940860

6.19.2 Computed excitation energies of compound 7 (cam-B3LYP/def2svp SMD CH_2Cl_2 GD3BJ)

Excitation energies and oscillator strengths:

Excited State 1: Singlet-A 3.5489 eV 349.36 nm f=0.4576 <S**2>=0.000

326 -> 330 0.11642

326 -> 331 0.17411

326 -> 333 0.24481

327 -> 330 -0.29546

328 -> 331 -0.24158

328 -> 333 -0.17748

329 -> 330 0.30811

This state for optimization and/or second-order correction.

Total Energy, E(TD-HF/TD-DFT) = -6466.79058544

Copying the excited state density for this state as the 1-particle RhoCI density.

Excited State 2: Singlet-A 3.5573 eV 348.53 nm f=0.6603 <S**2>=0.000

326 -> 330 0.18408

326 -> 331 0.13340

327 -> 330 -0.13506

327 -> 332 0.23073

327 -> 333 -0.29430

328 -> 330 -0.18129

329 -> 331 0.32176

329 -> 332 0.20105

Excited State 3: Singlet-A 3.5666 eV 347.62 nm f=0.7467 <S**2>=0.000

326 -> 330 0.10856

326 -> 331 -0.17726

326 -> 332 0.23658

326 -> 333 0.15132

326 -> 334 -0.10252

327 -> 331 -0.18423

328 -> 330 -0.17416

328 -> 332 0.22976

328 -> 333 0.26744

328 -> 334 -0.11614

329 -> 331 0.10215

329 -> 332 -0.24253

Excited State 4: Singlet-A 3.6324 eV 341.33 nm f=0.0521 <S**2>=0.000

326 -> 330 0.21901

326 -> 332 -0.18387

326 -> 334 0.11505

327 -> 331 -0.25578

327 -> 332 -0.15145

328 -> 330 -0.17582

328 -> 331 0.13356

328 -> 332 -0.21697

329 -> 333 0.29513

Excited State 5: Singlet-A 4.1798 eV 296.63 nm f=0.4261 <S**2>=0.000

326 -> 334 0.33897

326 -> 335 0.12541

326 -> 337 -0.11954

328 -> 332 0.10024

328 -> 334 0.39393

329 -> 334 -0.21342

Excited State 6: Singlet-A 4.1990 eV 295.27 nm f=0.5030 <S**2>=0.000

326 -> 334 0.10187

326 -> 336 -0.12127

326 -> 337 0.19446

327 -> 335 0.25264

327 -> 337 0.14481

328 -> 336 0.27674

328 -> 337 -0.22105

329 -> 335 -0.17082

329 -> 336 0.29104

Excited State 7: Singlet-A 4.2037 eV 294.94 nm f=0.5424 <S**2>=0.000

326 -> 335 0.27320

327 -> 335 -0.11483

327 -> 336 0.17400

327 -> 337 0.34313

328 -> 335 -0.13041

329 -> 335 0.23785

329 -> 336 0.22718

329 -> 337 0.11274

Excited State 8: Singlet-A 4.2361 eV 292.69 nm f=0.0501 <S**2>=0.000

326 -> 334 0.14346

326 -> 335 -0.18887

326 -> 336 0.13383

327 -> 335 0.19172

327 -> 336 0.28380

328 -> 334 0.12856

328 -> 335 0.20559

328 -> 336 -0.12178

329 -> 337 0.33459

Excited State 9: Singlet-A 4.3963 eV 282.02 nm f=0.3625 <S**2>=0.000

322 -> 333 0.14592

323 -> 331 -0.11675

324 -> 330 -0.16899

325 -> 330 0.22921

325 -> 331 -0.14304

326 -> 339 0.10713

326 -> 341 0.12436

327 -> 338 -0.14057

327 -> 339 -0.14155

327 -> 340 -0.10141

328 -> 338 0.12529

328 -> 339 -0.12288

328 -> 340 0.15607

329 -> 338 0.24967

Excited State 10: Singlet-A 4.4097 eV 281.16 nm f=0.6686 <S**2>=0.000

322 -> 331 0.11895

324 -> 330 -0.17810

324 -> 332 0.11187

324 -> 333 -0.15799

325 -> 331 0.16450

325 -> 332 0.13235

326 -> 338 0.10408

326 -> 339 0.20324

327 -> 338 -0.20455

327 -> 340 0.18192

327 -> 341 -0.16319

329 -> 339 0.26113

6.19.3 Computed emission energies of compound 7 (cam-B3LYP/def2svp SMD CH_2Cl_2 GD3BJ)

Excitation energies and oscillator strengths:

Excited State 1: Singlet-A 2.6870 eV 461.41 nm f=0.3386 <S**2>=0.000

329 -> 330 0.64288

329 -> 337 -0.10066

329 -> 339 0.10647

329 -> 340 0.13070

329 -> 344 0.11403

This state for optimization and/or second-order correction.

Total Energy, E(TD-HF/TD-DFT) = -6466.76082352

Copying the excited state density for this state as the 1-particle RhoCI density.

Excited State 2: Singlet-A 3.5718 eV 347.12 nm f=0.3331 <S**2>=0.000

326 -> 331 0.12739

326 -> 333 -0.26976

327 -> 332 -0.24501

327 -> 333 -0.18398

328 -> 331 0.43016

328 -> 332 -0.14189

328 -> 336 0.10245

Excited State 3: Singlet-A 3.5869 eV 345.66 nm f=0.6309 <S**2>=0.000

326 -> 331 -0.19992

326 -> 332 -0.30452

326 -> 333 0.10102

326 -> 334 0.12081

327 -> 331 -0.20101

327 -> 332 0.12756

327 -> 333 -0.32063

328 -> 332 0.22131

328 -> 333 0.15498

Excited State   4:   Singlet-A   3.6299 eV 341.57 nm f=0.3423 <S**2>=0.000

326 -> 331   -0.15555

326 -> 332   0.27574

326 -> 333   -0.15793

326 -> 334   -0.12941

327 -> 331   -0.24485

327 -> 332   -0.21995

328 -> 331   -0.10110

328 -> 333   0.27633

329 -> 340   -0.13158

Excited State   5:   Singlet-A   3.7383 eV 331.66 nm f=0.2307 <S**2>=0.000

321 -> 330   -0.10252

329 -> 330   -0.17145

329 -> 337   -0.26728

329 -> 338   0.15444

329 -> 339   0.30109

329 -> 340   0.41314

329 -> 344   -0.10184

Excited State   6:   Singlet-A   4.0385 eV 307.00 nm f=0.7255 <S**2>=0.000

325 -> 330   0.54461

329 -> 343   -0.13878

329 -> 344   0.28353

corrected external emission excitation: -6466.813452 - -6466.906560 = 0.093108 = 2.533599 eV 489.36 nm

6.20 Quantum chemical calculation data of tetrakis(4-(5-(10-hexyl-10*H*-phenothiazin-3-yl)thiophen-2-yl)phenyl)silane (8)

6.20.1 Computed xyz-coordinates of compound 8 (cam-B3LYP/def2svp SMD CH_2Cl_2 GD3BJ)

0 1

Si -0.11070 0.21459 -0.26171

C -1.12802 1.60729 -1.01557

C -1.27962 -1.00362 0.56968

C 0.87580 -0.66455 -1.60276

C 1.09590 0.91630 1.00042

C 2.20368 0.16649 1.42263

C 3.08614 0.65146 2.37945

C 2.90359 1.91782 2.95427

C 1.80178 2.67555 2.53098

C 0.92061 2.18191 1.57692

C 2.05832 -0.10219 -2.10639

C 2.78405 -0.72164 -3.11634

C 2.36184 -1.94351 -3.66002

C 1.18595 -2.51647 -3.15474

C 0.46031 -1.88565 -2.15168

C -2.23090 2.14028 -0.33185

C -2.96373 3.20041 -0.85154

C -2.62778 3.76754 -2.08926

C -1.53453 3.23146 -2.78475

C -0.80114 2.17750 -2.25380

C -0.97846 -1.60384 1.79998

C -1.82624 -2.54007 2.37925

C -3.02471 -2.90967 1.75199

C -3.33756 -2.30658 0.52502

C -2.48142 -1.37629 -0.05093

C 3.11884 -2.61566 -4.72429

C -3.91696 -3.89708 2.37370

C -3.38733 4.89127 -2.65302

C 3.82958 2.44380 3.96645

S -5.02043 5.21774 -2.15801

C -5.14792 6.59403 -3.20862

C -3.97939 6.76847 -3.91017

C -2.98501 5.80482 -3.59672

C 3.73647 3.60780 4.69083

C 4.81767 3.80576 5.58841

C 5.74673 2.79394 5.55490

S 5.27430 1.58174 4.40587

S 4.77920 -2.21469 -5.04420

C 4.89166 -3.36652 -6.33837

C 3.69767 -4.02899 -6.49187

C 2.69668 -3.60491 -5.57907

C -3.93529 -4.33236 3.67659

C -4.93636 -5.30534 3.93419

C -5.68877 -5.61911 2.82814

S -5.14571 -4.71073 1.45231

C -13.40585 8.63745 -1.28804

C -13.51884 9.35651 -2.47106

C -12.38655 9.69769 -3.20690

C -11.09871 9.35111 -2.77028

C -10.99989 8.65476 -1.55013

C -12.13696 8.28414 -0.83764

S -9.41906 8.36306 -0.81195

N -9.95281 9.69357 -3.51173

C -8.44707 8.19875 -2.28007

C -8.77286 8.93965 -3.43278

C -7.85076 8.89604 -4.49029

C -6.68381 8.14738 -4.41251

C -6.38046 7.38769 -3.27913

C -7.29401 7.42744 -2.21818

C -10.08574 10.66967 -4.59098

C -6.79558 -6.57636 2.71854

C -7.76790 -6.48354 1.72002

C -8.82266 -7.38535 1.64856

C -8.95249 -8.44194 2.56227

C -7.94647 -8.55569 3.54331

C -6.91253 -7.63425 3.63032

S -7.88054 -9.98075 4.58862

N -10.01798 -9.35127 2.49477

C -9.61158 -10.32193 4.71258

C -10.47166 -10.04423 3.63244

C -11.79532 -10.49857 3.73579

C -12.24218 -11.18400 4.86284

C -11.39030 -11.41790 5.93471

C -10.07328 -10.97483 5.85212

C -10.85236 -9.35666 1.29515

C -11.97279 -8.32127 1.24743

C 6.13797 -3.54604 -7.09225

C 6.40444 -4.73862 -7.77079

C 7.58552 -4.92013 -8.47815

C 8.56044 -3.91335 -8.56094

C 8.27148 -2.70035 -7.90577

C 7.10344 -2.53458 -7.17327

S 9.31503 -1.29163 -8.13719

N 9.75578 -4.09910 -9.27058

C 10.86612 -2.11781 -8.33816

C 10.92525 -3.38553 -8.94900

C 12.19984 -3.89994 -9.23224

C 13.35628 -3.19318 -8.91134

C 13.28074 -1.96064 -8.27550

C 12.02565 -1.43251 -7.98635

C 9.86863 -5.26056 -10.14998

C 10.26944 -6.57324 -9.48236

C 11.85975 5.52459 11.12479

C 12.76876 4.61075 10.60731

C 12.34191 3.57109 9.78472

C 10.98538 3.39620 9.47039

C 10.07301 4.30907 10.03431

C 10.50926 5.36793 10.82587

S 8.33095 4.04664 9.87645

N 10.55222 2.34759 8.63772

C 8.30351 3.26770 8.28911

C 9.37299 2.44296 7.88475

C 9.18859 1.71117 6.70207

C 8.02008 1.80981 5.95676

C 6.97866 2.65795 6.34045

C 7.15171 3.38726 7.52475

C 11.49386 1.27622 8.32083

C -10.56301 10.12926 -5.93635

C 12.45974 1.54799 7.17085

H 2.39064 -0.82248 0.99592

H 3.93074 0.02801 2.68134

H 1.63158 3.66967 2.94661

H 0.07845 2.80801 1.27287

H 2.42898 0.84454 -1.70455

H 3.68974 -0.23978 -3.49171

H 0.84197 -3.47781 -3.53949

H -0.44673 -2.36945 -1.78195

H -2.52983 1.72379 0.63373

H -3.80374 3.59923 -0.27813

H -1.26491 3.63342 -3.76281

H 0.03964 1.78505 -2.83056

H -0.05581 -1.34252 2.32345

H -1.54577 -2.99942 3.32849

H -4.27126 -2.55565 0.01558

H -2.76754 -0.92580 -1.00507

H -3.82762 7.58445 -4.61703

H -1.98779 5.80371 -4.03735

H 2.91182 4.31249 4.58639

H 4.91329 4.68123 6.23081

H 3.53314 -4.78683 -7.25809

H 1.68299 -4.00576 -5.57079

H -3.26064 -3.94833 4.44179

H -5.11222 -5.74345 4.91687

H -14.29096 8.35638 -0.71458

H -14.50105 9.65321 -2.84509

H -12.52272 10.24411 -4.13660

H -12.01731 7.73031 0.09638

H -8.04046 9.44726 -5.40729

H -6.01109 8.13748 -5.27142

H -7.08944 6.87536 -1.29796

H -10.77101 11.45312 -4.23982

H -9.10966 11.15931 -4.71118

H -7.72856 -5.67354 0.98865

H -9.55866 -7.24433 0.86159

H -6.15629 -7.76965 4.40564

H -12.50129 -10.31982 2.92916

H -13.27893 -11.52544 4.89686

H -11.73912 -11.94309 6.82564

H -9.37769 -11.15592 6.67481

H -10.18505 -9.22326 0.43283

H -11.27231 -10.36648 1.19323

H -12.51785 -8.41826 0.29617

H -12.69570 -8.45261 2.06384

H -11.59117 -7.29337 1.31292

H 5.68994 -5.56201 -7.72778

H 7.74303 -5.87899 -8.96439

H 6.93038 -1.57174 -6.68700

H 12.30644 -4.86935 -9.71194

H 14.32690 -3.62939 -9.15685

H 14.18417 -1.40879 -8.00991

H 11.93587 -0.45898 -7.49887

H 8.90298 -5.38051 -10.65956

H 10.59065 -5.00605 -10.93753

H 10.32665 -7.36673 -10.24293

H 11.24959 -6.50653 -8.99130

H 9.54425 -6.88963 -8.72033

H 12.19058 6.34881 11.75920

H 13.83367 4.70423 10.83070

H 13.08980 2.89015 9.38696

H 9.77076 6.06393 11.23049

H 9.96878 1.04934 6.33590

H 7.94245 1.22328 5.03894

H 6.35356 4.03871 7.88475

H 12.05670 1.05337 9.23719

H 10.90392 0.37450 8.10766

H -10.63021 10.95745 -6.65814

H -9.87683 9.37804 -6.35019

H -11.55449 9.66181 -5.86765

H 13.11696 0.67601 7.03284

H 11.93591 1.72750 6.22226

H 13.09635 2.42240 7.36183

SCF Done: E(RCAM-B3LYP) = -7390.01182993   A.U. after   1 cycles

Zero-point correction=   1.434043 (Hartree/Particle)

Thermal correction to Energy= 1.526485

Thermal correction to Enthalpy= 1.527429

Thermal correction to Gibbs Free Energy= 1.279376

Sum of electronic and zero-point Energies= -7388.577787

Sum of electronic and thermal Energies= -7388.485345

Sum of electronic and thermal Enthalpies= -7388.484401

Sum of electronic and thermal Free Energies= -7388.732454

6.20.2 Computed excitation energies of compound 8 (cam-B3LYP/def2svp SMD CH_2Cl_2 GD3BJ)

Excitation energies and oscillator strengths:

Excited State 1: Singlet-A 3.3981 eV 364.86 nm f=1.2989 <S**2>=0.000

405 -> 410 -0.12164

406 -> 411 0.16402

408 -> 411 0.15520

409 -> 410 0.40838

409 -> 411 0.31753

409 -> 413 0.16296

409 -> 414 -0.11844

409 -> 415 0.11354

409 -> 417 -0.12682

This state for optimization and/or second-order correction.

Total Energy, E(TD-HF/TD-DFT) = -7389.88695093

Copying the excited state density for this state as the 1-particle RhoCI density.

Excited State 2: Singlet-A 3.4327 eV 361.18 nm f=1.8314 <S**2>=0.000

406 -> 412 0.13323

406 -> 413 -0.23784

407 -> 412 -0.22899

408 -> 410 -0.25339

408 -> 411 0.31635

408 -> 413 -0.17096

408 -> 414 0.13535

408 -> 415 0.13453

409 -> 411 -0.12496

409 -> 413 0.10205

Excited State 3: Singlet-A 3.4446 eV 359.94 nm f=1.0022 <S**2>=0.000

406 -> 412 0.33569

406 -> 413 0.13362

406 -> 416 0.16451

407 -> 410 -0.24408

407 -> 411 0.12599

407 -> 413 0.30572

407 -> 414 0.13135

407 -> 417 -0.11248

408 -> 412 -0.20572

Excited State 4: Singlet-A 3.5012 eV 354.12 nm f=0.0640 <S**2>=0.000

406 -> 410 -0.25160

406 -> 411 0.12690

406 -> 413 0.15461

406 -> 414 0.15803

407 -> 412 0.33339

407 -> 416 0.18289

408 -> 413 -0.26726

408 -> 415 0.10347

408 -> 417 0.10171

409 -> 411 -0.19597

Excited State 5: Singlet-A 3.9668 eV 312.55 nm f=0.6818 <S**2>=0.000

402 -> 411 0.16209

403 -> 411 0.13001

405 -> 410 0.29390

405 -> 411 0.18803

405 -> 413 0.11287

406 -> 415 0.11884

408 -> 415 0.21166

409 -> 414 -0.15839

409 -> 415 0.12644

409 -> 417 -0.23007

409 -> 418 -0.20000

Excited State 6: Singlet-A 3.9766 eV 311.79 nm f=1.2019 <S**2>=0.000

402 -> 413 0.19056

403 -> 410 0.15594

403 -> 411 -0.15136

403 -> 412 -0.10968

404 -> 411 -0.11477

404 -> 412 0.14026

405 -> 411 0.13061

406 -> 415 -0.12660

406 -> 417 -0.19016

407 -> 416 0.18700

408 -> 414 -0.18651

408 -> 415 -0.21534

408 -> 418 -0.15547

409 -> 415 0.16298

Excited State 7: Singlet-A 3.9921 eV 310.57 nm f=0.7051 <S**2>=0.000

402 -> 412 0.20403

403 -> 412 -0.12907

403 -> 413 -0.12587

404 -> 410 -0.16581

404 -> 412 -0.12824

404 -> 413 0.16708

406 -> 416 0.27911

407 -> 414 0.16638

407 -> 417 -0.24497

407 -> 418 0.15326

408 -> 416 -0.14494

Excited State 8: Singlet-A 4.0701 eV 304.62 nm f=0.0319 <S**2>=0.000

402 -> 410 -0.15373

403 -> 413 -0.13179

404 -> 412 0.18560

405 -> 411 -0.14812

406 -> 414 0.14426

406 -> 417 -0.11032

406 -> 418 0.17534

407 -> 412 -0.10572

407 -> 416 0.23429

407 -> 420 -0.11840

408 -> 415 0.14022

408 -> 417 0.17144

409 -> 415 -0.12832

409 -> 417 0.10840

Excited State 9: Singlet-A 4.2294 eV 293.15 nm f=0.0064 <S**2>=0.000

405 -> 410 -0.19410

405 -> 411 -0.16104

406 -> 419 0.14525

409 -> 418 -0.18346

409 -> 419 0.42132

409 -> 420 -0.16149

409 -> 422 0.19239

Excited State 10: Singlet-A 4.2467 eV 291.95 nm f=0.0281 <S**2>=0.000

402 -> 412 0.11295

404 -> 410 -0.14717

404 -> 412 -0.16878

406 -> 419 0.12417

406 -> 420 0.20751

406 -> 421	-0.15695
407 -> 418	-0.14247
407 -> 419	-0.16533
407 -> 420	-0.15587
407 -> 421	0.20600
407 -> 422	0.10112
408 -> 418	-0.10945
408 -> 419	-0.14656
408 -> 422	0.17548

6.20.3 Computed emission energies of compound 8 (cam-B3LYP/def2svp SMD CH_2Cl_2 GD3BJ)

Excitation energies and oscillator strengths:

Excited State 1: Singlet-A 2.6255 eV 472.24 nm f=1.1852 <S**2>=0.000

405 -> 410 0.10735

409 -> 410 -0.65400

This state for optimization and/or second-order correction.

Total Energy, E(TD-HF/TD-DFT) = -7389.84010241

Copying the excited state density for this state as the 1-particle RhoCI density.

Excited State 2: Singlet-A 3.4635 eV 357.97 nm f=1.4282 <S**2>=0.000

406 -> 413 -0.21023

407 -> 412 0.19239

408 -> 411 0.39639

408 -> 413 0.32691

408 -> 414 -0.12229

408 -> 415 0.13185

408 -> 416 0.16559

Excited State 3: Singlet-A 3.4703 eV 357.27 nm f=0.7838 <S**2>=0.000

406 -> 412 -0.40679

406 -> 415 -0.15208

406 -> 416 0.12853

407 -> 411 -0.29506

407 -> 413 0.29855

407 -> 414 0.13071

407 -> 417 0.15218

Excited State 4: Singlet-A 3.4860 eV 355.66 nm f=0.9945 <S**2>=0.000

405 -> 410 0.42400

406 -> 411 -0.19221

406 -> 413 0.15734

407 -> 412 -0.26039

408 -> 413 0.12821

409 -> 414 0.10084

409 -> 417 -0.11206

409 -> 418 -0.14343

Excited State 5: Singlet-A 3.5783 eV 346.49 nm f=0.0803 <S**2>=0.000

405 -> 410 0.35684

406 -> 411 0.20732

406 -> 413 -0.14244

406 -> 414 -0.11685

406 -> 417 -0.12312

407 -> 412 0.25591

407 -> 415 0.12766

407 -> 416 -0.11143

408 -> 411 -0.12064

408 -> 413 -0.20142

408 -> 416 -0.11366

409 -> 418 -0.11661

Excited State 6: Singlet-A 3.8447 eV 322.48 nm f=0.0013 <S**2>=0.000

401 -> 410 0.10860

405 -> 410 0.27864

409 -> 410 0.12983

409 -> 418 0.15333

409 -> 426 -0.14737

409 -> 428 0.51269

7. Crystallographic data

7.1 X-ray structure determinations

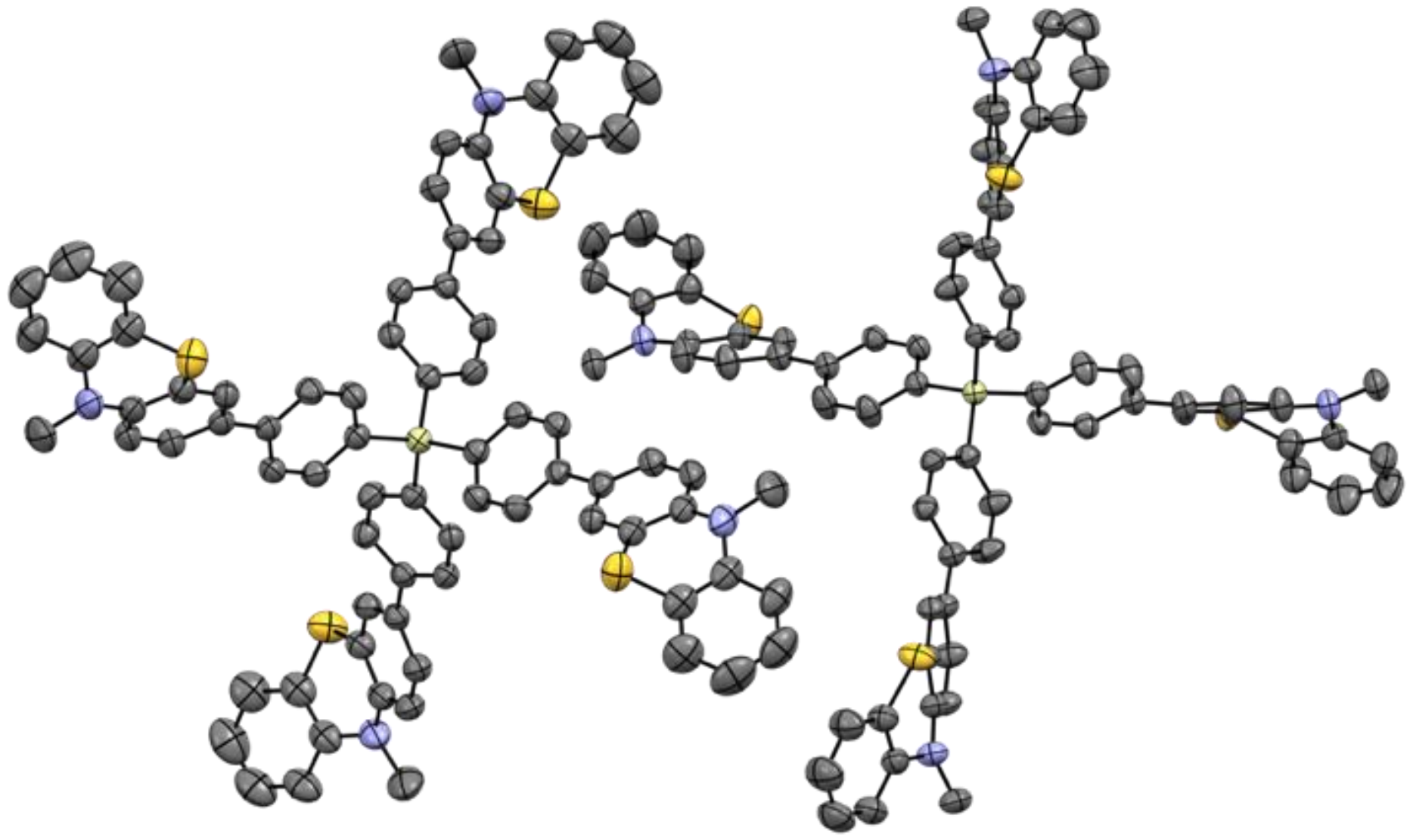

Figure S103. Molecular structure of **5a**. Thermal ellipsoids are shown with 50% probability. Hydrogens are omitted for clarity.

Table S3. Crystal data and structure refinement for **5a**.

Empirical formula	$C_{76}H_{56}N_4S_4Si$ (CCDC-No 2483488)
Formula weight	1181.57
Temperature/K	298.88(10)
Crystal system	tetragonal
Space group	$I4_1/a$
a/Å	29.1639(3)
b/Å	29.1639(3)
c/Å	14.4520(2)
α/°	90
β/°	90
γ/°	90
Volume/Å^3	12291.9(3)
Z	8
ρ_{calc}g/cm^3	1.277
μ/mm^{-1}	1.979
F(000)	4944.0
Crystal size/mm^3	0.42 × 0.13 × 0.11
Radiation	Cu Kα (λ = 1.54184)
2Θ range for data collection/°	6.062 to 152.604

Index ranges	$-33 \le h \le 34$, $-34 \le k \le 34$, $-7 \le l \le 18$
Reflections collected	20817
Independent reflections	6093 [R_{int} = 0.0366, R_{sigma} = 0.0377]
Data/restraints/parameters	6093/0/386
Goodness-of-fit on F^2	1.071
Final R indexes [I>=2σ (I)]	R_1 = 0.0409, wR_2 = 0.1151
Final R indexes [all data]	R_1 = 0.0472, wR_2 = 0.1196
Largest diff. peak/hole / e $Å^{-3}$	0.35/–0.32

7.2 Hirshfeld analysis

The supramolecular packing interactions were analyzed for compound **5a** by Hirshfeld surfaces using the Crystal Explorer V21.3 program,[41] as described in the literature.[42-44]

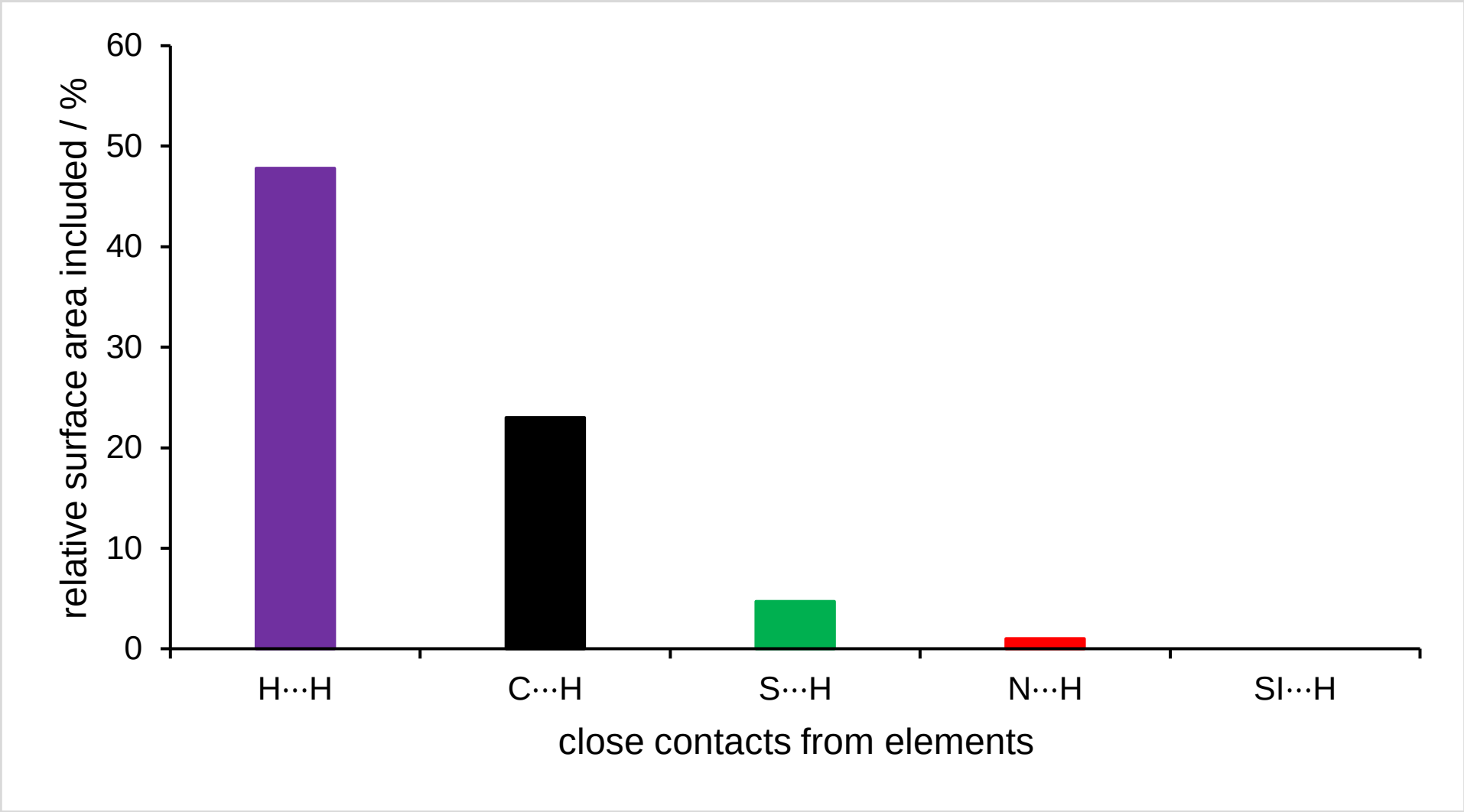

Figure S104. Percentage contributions to the Hirshfeld surface area for the various close intermolecular contacts for **5a**.

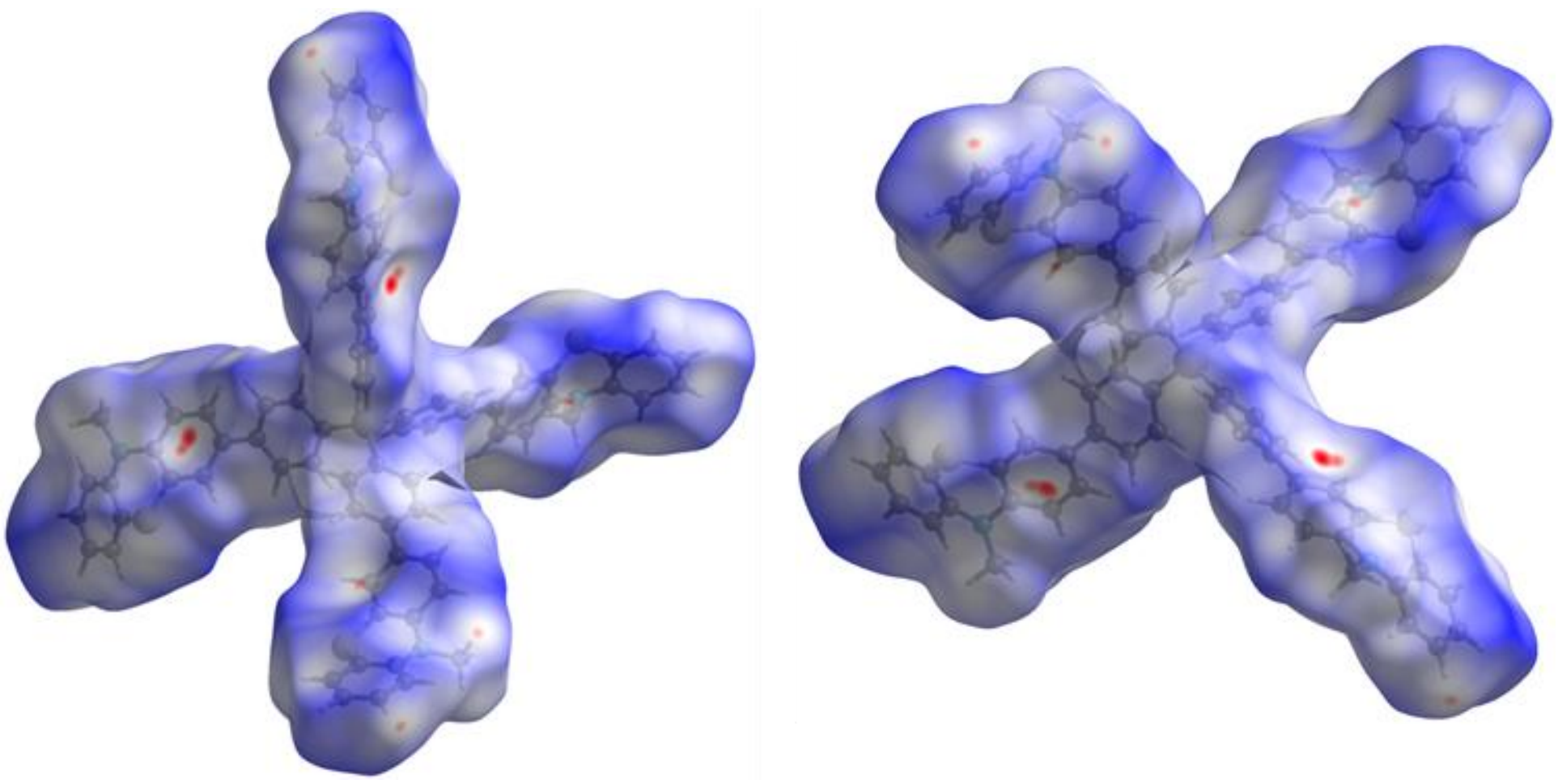

Figure S105. Hirshfeld surface of **5a** mapped with the d_{norm} property. The red spots represent the closest contacts and blue the most distant contacts. A breakdown of the d_{norm} property is shown below.

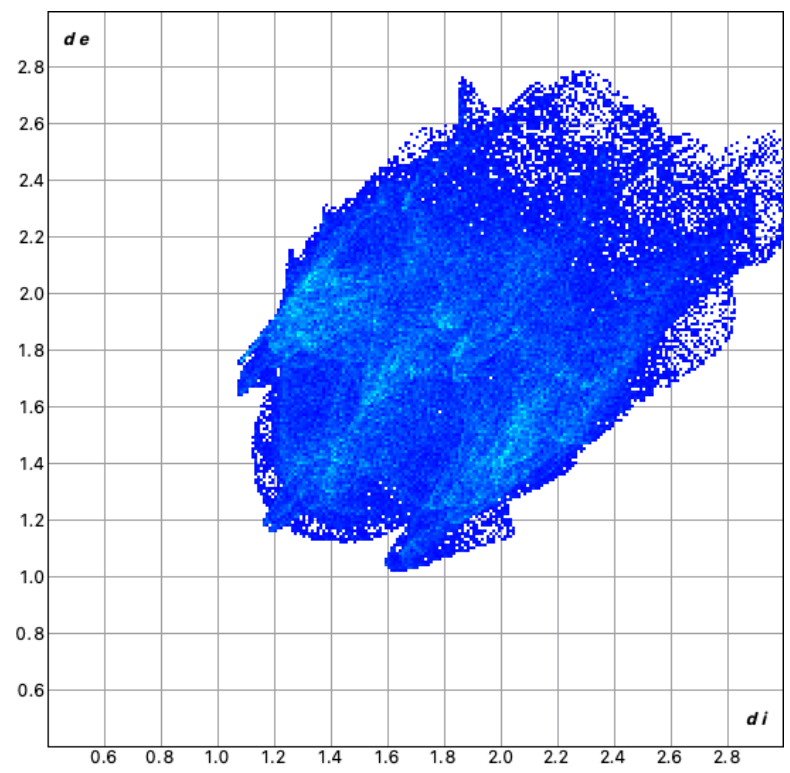

Figure S106. Fingerprint plot for **5a** (d_i and d_e are the distances from the surface to the nearest atom interior and exterior to the surface, respectively). A breakdown of the plot into the contributions from Si···H, S···H, N···H, C···H, H···H close intermolecular contacts is given below.

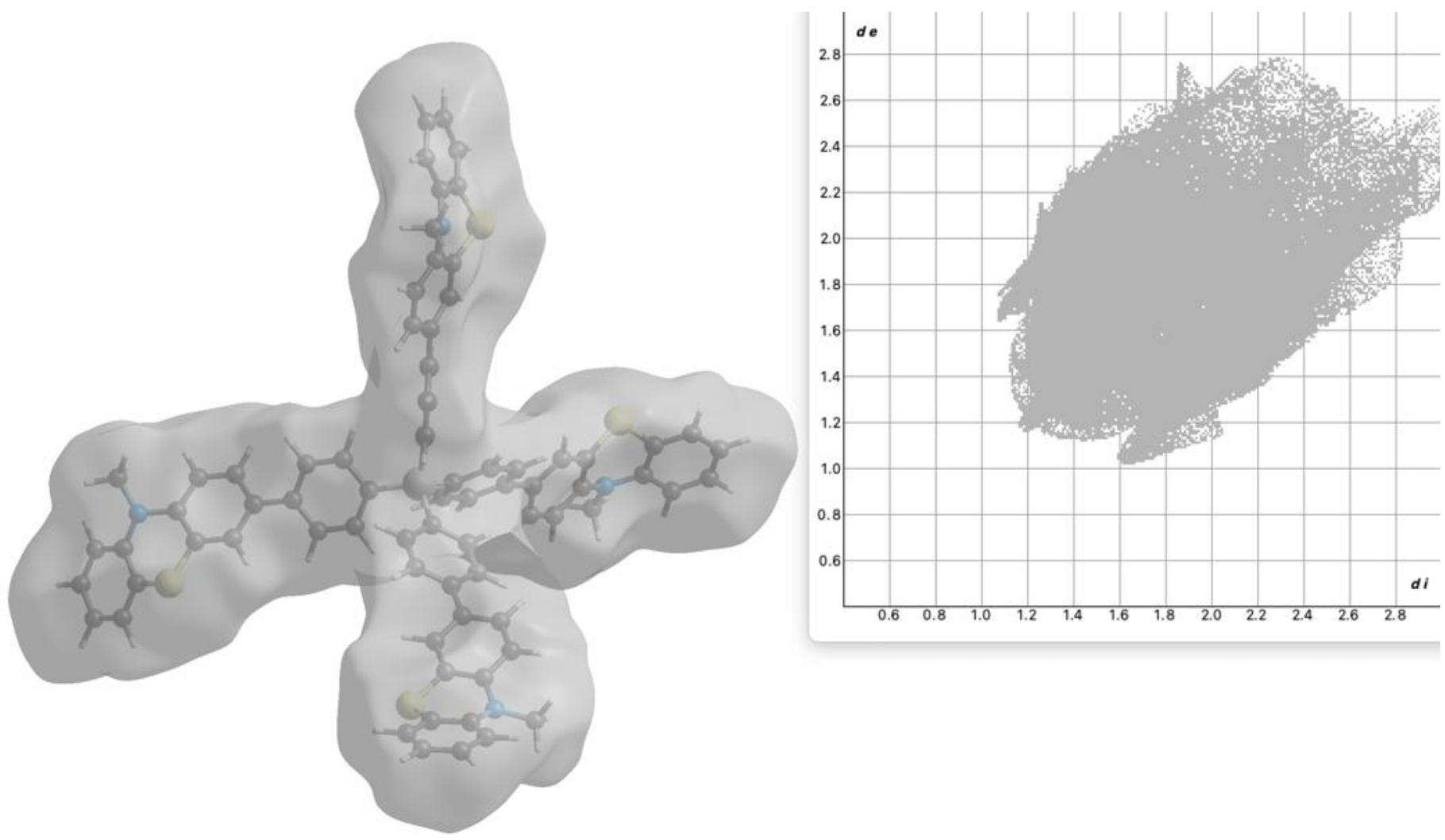

Figure S107. Fingerprint plot contributions from Si···H close intermolecular contacts for **5a**. Relative surface area included: 0.0%.

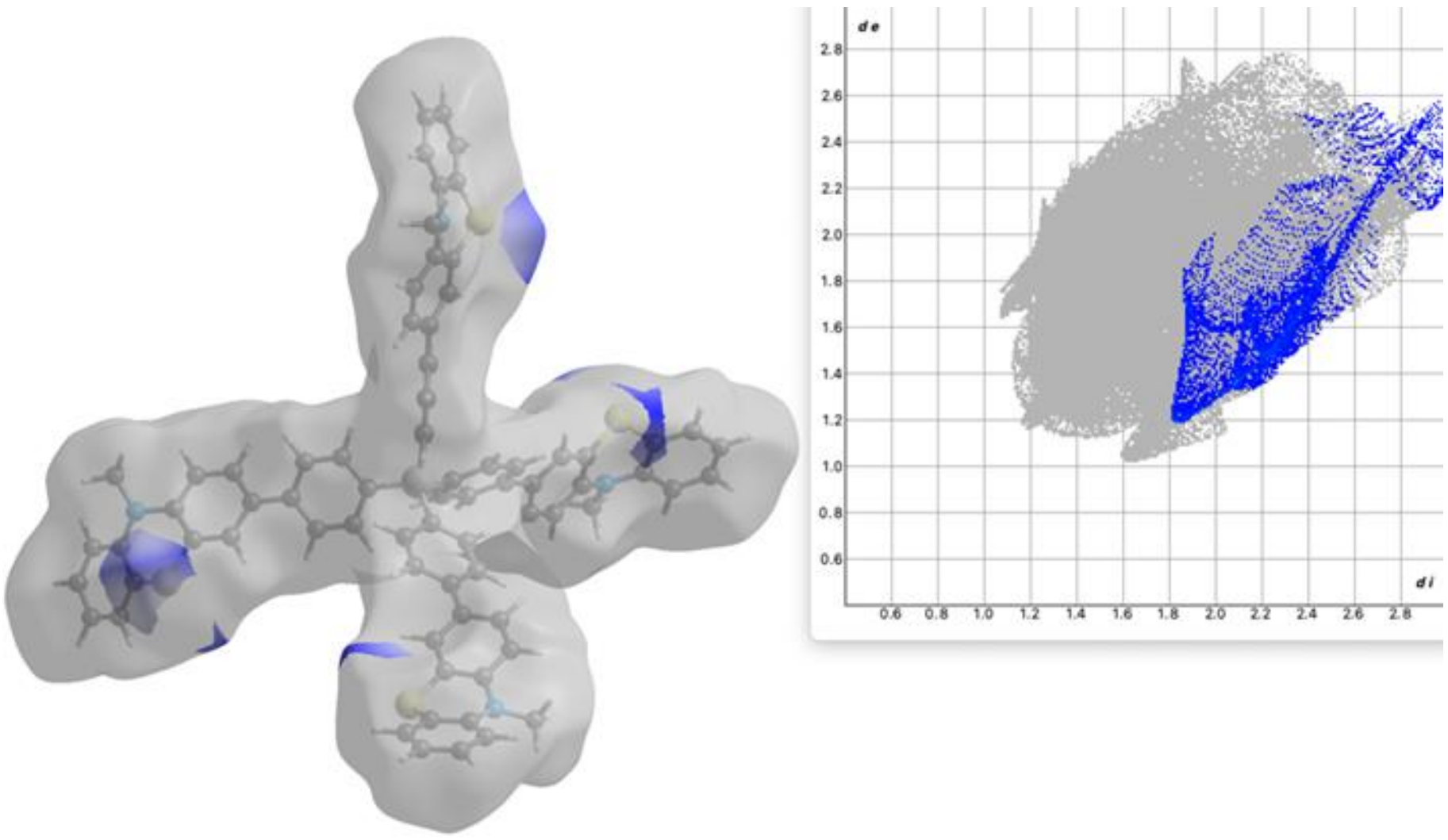

Figure S108. Fingerprint plot contributions from S···H close intermolecular contacts for **5a**. Relative surface area included: 4.7%.

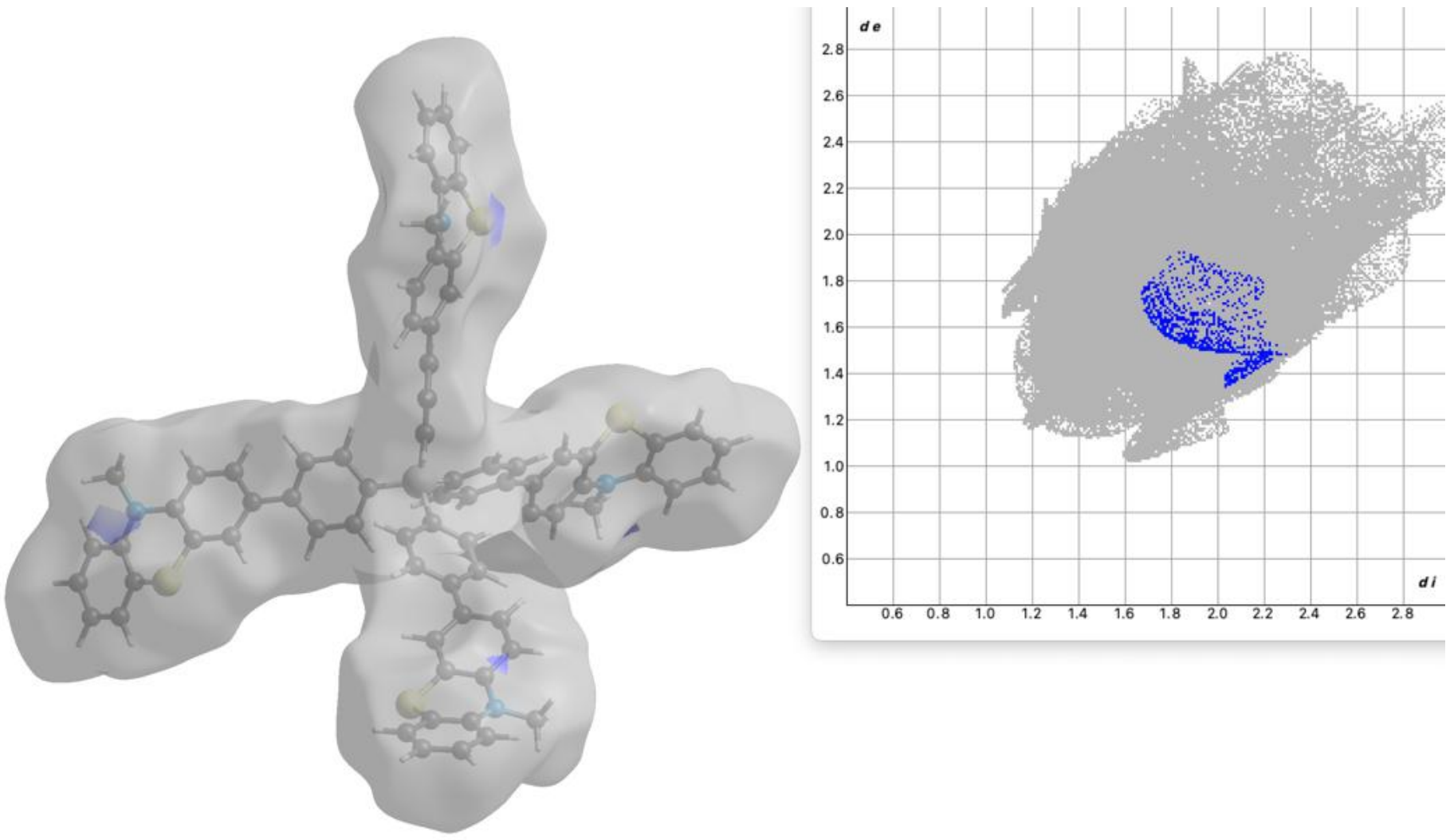

Figure S109. Fingerprint plot contributions from N···H close intermolecular contacts for **5a**. Relative surface area included: 0.8%.

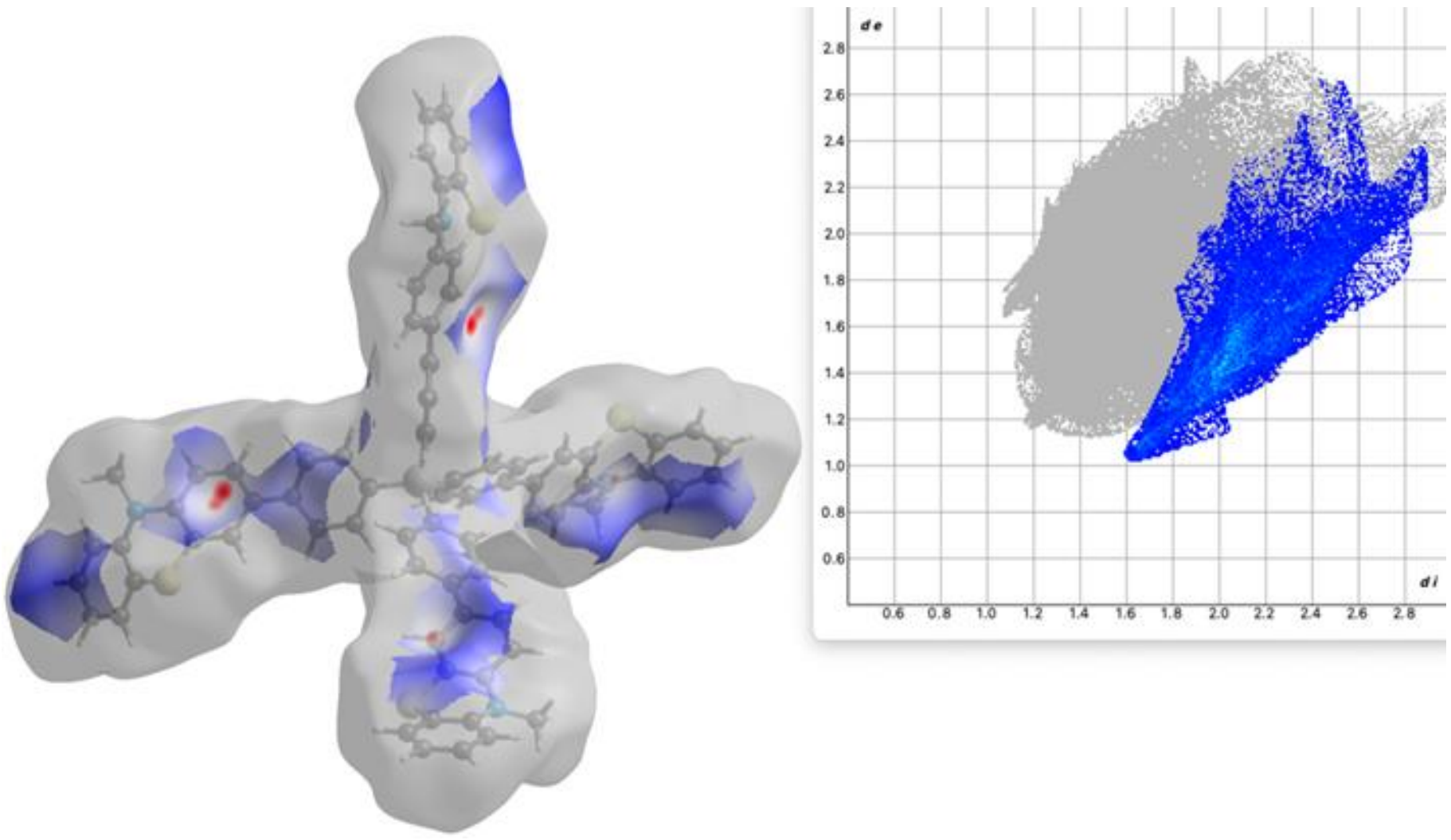

Figure S110. Fingerprint plot contributions from C···H close intermolecular contacts for **5a**. Relative surface area included: 22.8%.

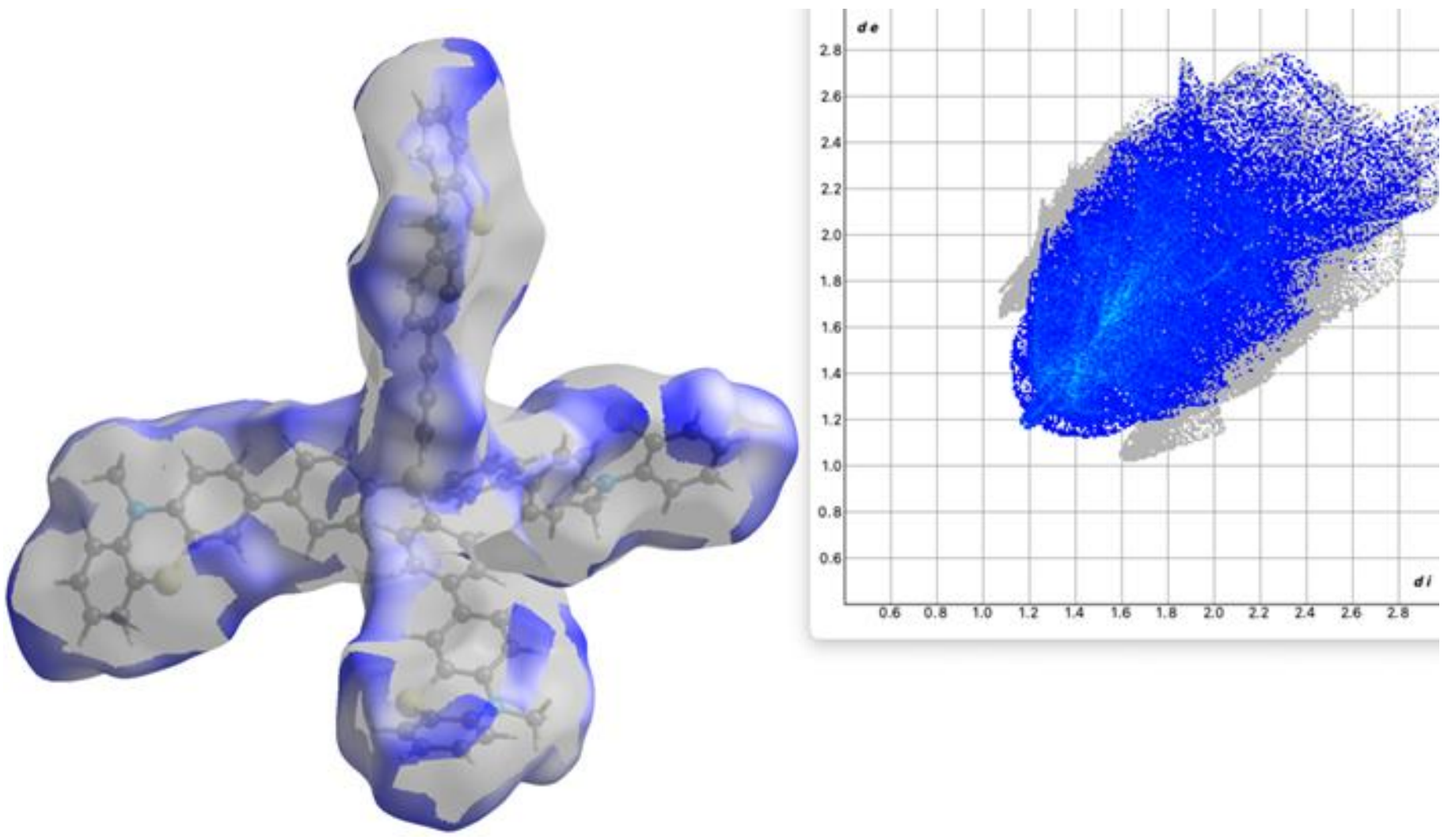

Figure S111. Fingerprint plot contributions from H···H close intermolecular contacts for **5a**. Relative surface area included: 47.8%.

8. Literature

[14] S.-Y. Liu, J. W. Jung, C.-Z. Li, J. Huang, J. Zhang, H. Chen, A. K.-Y. Jen, *Three-dimensional molecular donors combined with polymeric acceptors for high performance fullerene-free organic photovoltaic devices*, *J. Mater. Chem. A* **2015**, *3*, 22162-22169.

[29] M. Sailer, R.-A. Gropeanu, T. J. J. Müller, *Practical synthesis of iodo phenothiazines. A facile access to electrophore building blocks*, *J. Org. Chem.* **2003**, *68*, 7509-7512.

[30] M. Sailer, A. W. Franz, T. J. J. Müller, *Synthesis and electronic properties of monodisperse oligophenothiazines*, *Chem. Eur. J.* **2008**, *14*, 2602-2614.

[32] I. A. Wright, M. K. Etherington, A. S. Batsanov, A. P. Monkman, M. R. Bryce, *Oxidation State Tuning of Room Temperature Phosphorescence and Delayed Fluorescence in Phenothiazine and Phenothiazine-5,5-dioxide Dimers*, *Chem. Eur. J.* **2023**, *29*, e202300428.

[33] A. Hallberg, A. R. Martin, *A New Synthesis of 1-Chlorophenothiazine*, *Synth. Commun.* **1983**, *13*, 467-470.

[34] Y. S. Yang, H. D. Kim, J.-H. Ryu, K. K. Kim, S. S. Park, K.-S. Ahn, J. H. Kim, *Effects of anchoring groups in multi-anchoring organic dyes with thiophene bridge for dye-sensitized solar cells*, *Synth. Met.* **2011**, *161*, 850-855.

[35] C. S. Krämer, K. Zeitler, T. J. J. Müller, *First synthesis and electronic properties of (hetero)aryl bridged and directly linked redox active phenothiazinyl dyads and triads*, *Tetrahedron Lett.* **2001**, *42*, 8619-8624.

[36] L. Levi, S. Scheuren, T. J. J. Müller, *A Novel N-Benzylation of Phenothiazine with Benzyl Alcohols Activated by n-Propylphosphonic Acid Anhydride (T3P®)*, *Synthesis* **2014**, *46*, 3059-3066.

[37] M. Stephan, B. Stute, E. von Lieres, T. J. J. Müller, *Consecutive Three-Component Synthesis of Phenothiazine-Based Merocyanines – Bayesian Optimization, Electronic properties, and DSSC Characteristics*, *Eur. J. Org. Chem.* **2022**, *2022*, e202200163.

[38] Deposition Number CCDC 2483488 contains the supplementary crystallographic data for this paper. These data are provided free of charge by the joint Cambridge Crystallographic Data Centre and Fachinformationszentrum Karlsruhe Access Structures service.

[41] P. R. Spackman, M. J. Turner, J. J. McKinnon, S. K. Wolff, D. J. Grimwood, D. Jayatilaka, M. A. Spackman, *CrystalExplorer: a program for Hirshfeld surface analysis, visualization and quantitative analysis of molecular crystals*, *J. Appl. Crystallogr.* **2021**, *54*, 1006-1011.

[42] J. J. McKinnon, D. Jayatilaka, M. A. Spackman, *Towards quantitative analysis of intermolecular interactions with Hirshfeld surfaces*, *Chem. Commun.* **2007**, 3814-3816.

[43] J. J. McKinnon, M. A. Spackman, A. S. Mitchell, *Novel tools for visualizing and exploring intermolecular interactions in molecular crystals*, *Acta Crystallogr., Sect. B: Struct. Sci.* **2004**, *60*, 627-668.

[44] M. A. Spackman, J. J. McKinnon, *Fingerprinting intermolecular interactions in molecular crystals*, *CrystEngComm* **2002**, *4*, 378-392.

[51] Gaussian 16, M. J. Frisch, G. W. Trucks, H. B. Schlegel, G. E. Scuseria, M. A. Robb, J. R. Cheeseman, G. Scalmani, V. Barone, G. A. Petersson, H. Nakatsuji, X. Li, M. Caricato, A. V. Marenich, J. Bloino, B. G. Janesko, R. Gomperts, B. Mennucci, H. P. Hratchian, J. V. Ortiz, A. F. Izmaylov, J. L. Sonnenberg, D. Williams-Young, F. Ding, F. Lipparini, F. Egidi, J. Goings, B. Peng, A. Petrone, T. Henderson, D. Ranasinghe, V. G. Zakrzewski, J. Gao, N. Rega, G. Zheng, W. Liang, M. Hada, M. Ehara, K. Toyota, R. Fukuda, J. Hasegawa, M. Ishida, T. Nakajima, Y. Honda, O. Kitao, H. Nakai, T. Vreven, K. Throssell, J. A. Montgomery, Jr., J. E. Peralta, F. Ogliaro, M. J. Bearpark, J. J. Heyd, E. N. Brothers, K. N. Kudin, V. N. Staroverov, T. A. Keith, R. Kobayashi, J. Normand, K. Raghavachari, A. P. Rendell, J. C. Burant, S. S. Iyengar, J. Tomasi, M. Cossi, J. M. Millam, M. Klene, C. Adamo, R. Cammi, J. W. Ochterski, R. L. Martin, K.

Morokuma, O. Farkas, J. B. Foresman, D. J. Fox, Gaussian, Inc., Wallingford, CT, **2016**.

[54] G. M. Sheldrick, *SHELXT – integrated space-group and crystal-structure determination*, *Acta Crystallogr., Sect. A: Found. Adv.* **2015**, *71*, 3-8.

[55] O. V. Dolomanov, L. J. Bourhis, R. J. Gildea, J. A. K. Howard, H. Puschmann, *OLEX2: a complete structure solution, refinement and analysis program*, *J. Appl. Crystallogr.* **2009**, *42*, 339-341.

[56] G. M. Sheldrick, *Crystal structure refinement with SHELXL*, *Acta Crystallogr., Sect. C: Struct. Chem.* **2015**, *71*, 3-8.

[57] I. Noviandri, K. N. Brown, D. S. Fleming, P. T. Gulyas, P. A. Lay, A. F. Masters, L. Phillips, *The Decamethylferrocenium/Decamethylferrocene Redox Couple: A Superior Redox Standard to the Ferrocenium/Ferrocene Redox Couple for Studying Solvent Effects on the Thermodynamics of Electron Transfer*, *J. Phys. Chem. B* **1999**, *103*, 6713-6722.

[58] R. R. Gagné, C. A. Koval, G. C. Lisensky, *Ferrocene as an internal standard for electrochemical measurements*, *Inorg. Chem.* **1980**, *19*, 2854-2855.

[59] G. Gritzner, J. Kůta, *Recommendations on reporting electrode potentials in nonaqueous solvents*, *Electrochim. Acta* **1984**, *29*, 869-873.

[60] C. Dostert, C. Wanstrath, W. Frank, T. J. J. Müller, *4H-dithieno[2,3-b:3',2'-e][1,4]thiazines – synthesis and electronic properties of a novel class of electron rich redox systems*, *Chem. Commun.* **2012**, *48*, 7271-7273.

[61] Gaussian 09, M. J. Frisch, G. W. Trucks, H. B. Schlegel, G. E. Scuseria, M. A. Robb, J. R. Cheeseman, G. Scalmani, V. Barone, G. A. Petersson, H. Nakatsuji, X. Li, M. Caricato, A. Marenich, J. Bloino, B. G. Janesko, R. Gomperts, B. Mennucci, H. P. Hratchian, J. V. Ortiz, A. F. Izmaylov, J. L. Sonnenberg, D. Williams-Young, F. Ding, F. Lipparini, F. Egidi, J. Goings, B. Peng, A. Petrone, T. Henderson, D. Ranasinghe, V. G. Zakrzewski, J. Gao, N. Rega, G. Zheng, W. Liang, M. Hada, M. Ehara, K. Toyota, R. Fukuda, J. Hasegawa, M. Ishida, T. Nakajima, Y. Honda, O. Kitao, H. Nakai, T. Vreven, K. Throssell, J. A. Montgomery, Jr., J. E. Peralta, F. Ogliaro, M. Bearpark, J. J. Heyd, E. Brothers, K. N. Kudin, V. N. Staroverov, T. Keith, R. Kobayashi, J. Normand, K. Raghavachari, A. Rendell, J. C. Burant, S. S. Iyengar, J. Tomasi, M. Cossi, J. M. Millam, M. Klene, C. Adamo, R. Cammi, J. W. Ochterski, R. L. Martin, K. Morokuma, O. Farkas, J. B. Foresman, D. J. Fox, Gaussian, Inc., Wallingford, CT, **2009**.

[62] C. S. Krämer, T. J. Zimmermann, M. Sailer, T. J. J. Müller, *Syntheses of phenothiazinylboronic acid derivatives – Suitable starting points for the construction of redox active materials*, *Synthesis* **2002**, *2002*, 1163-1170.

[63] N. Bucci, T. J. J. Müller, *First syntheses and electronic properties of (oligo)phenothiazine–C60 dyads*, *Tetrahedron Lett.* **2006**, *47*, 8323-8327.
